# A postoperative recurrence prediction model for intrahepatic cholangiocarcinoma based on multi-omics analysis of adjacent-to-tumor tissues

**DOI:** 10.3389/fonc.2026.1753415

**Published:** 2026-03-25

**Authors:** Rongyu Ping, Qianfu Zhao, Wenhao Ma, Huqiang Wang, Weiren Liu, Dong Yang

**Affiliations:** 1 State Key Laboratory of Medical Proteomics, National Center for Protein Sciences (Beijing), Beijing, China; 2 The Second Medical Center & National Clinical Research Center for Geriatric Diseases, Chinese PLA General Hospital, Beijing, China; 3 Department of Hepatobiliary Cancer Surgery and Transplantation, Liver Cancer Institute, Zhongshan Hospital, Fudan University, Key Laboratory of Carcinogenesis and Cancer Invasion of Ministry of Education, Shanghai, China; 4 College of Life Sciences, Hebei University, Baoding, Hebei, China

**Keywords:** adjacent-to-tumor tissues, intrahepatic cholangiocarcinoma, molecular subtype, prediction model, proteomics

## Abstract

**Background:**

The patients with intrahepatic cholangiocarcinoma (iCCA) are highly susceptible to recurrence after radical resection, while predicting recurrence remains challenging. Adjacent-to-tumor tissues (ATTs), as the main microenvironment for postoperative recurrence, exhibited superior predictive value for recurrence compared with tumor tissues. However, the postoperative recurrence prediction model based on iCCA ATTs characteristics has not been studied. This study aims to construct recurrence prediction model based on iCCA ATTs and discover possible beneficial postoperative treatment options.

**Methods:**

Consensus clustering was employed to classify the proteome of 116 iCCA ATTs. Multivariate Cox regression was used to construct recurrence prediction model. Tissue microarray containing 88 iCCA ATTs (another independent cohort) and immunohistochemistry were used for validating the expression of target proteins.

**Results:**

We classified iCCA ATTs into two subtypes (S1 and S2) based on 116 iCCA patients’ proteomic data, where S1 exhibited higher recurrence rates and immune scores than those of S2. We constructed a multivariate Cox regression model based on four molecules (ENO3, HSPA13, POSTN, PTBP3). The expression of POSTN was an independent prognostic factor for recurrence of iCCA. The high-risk group for recurrence exhibited a poorer response to immunotherapy but was more sensitive to certain chemotherapy and targeted therapies.

**Conclusions:**

We obtained novel molecular subtyping and constructed a postoperative recurrence prediction model based on iCCA ATTs, offering novel perspectives for tumorigenesis and providing some references for postoperative treatment options.

## 1 Introduction

Intrahepatic cholangiocarcinoma (iCCA) is the second most common primary malignant tumor of the liver, accounting for 10-20%, and its incidence has been increasing year by year over the past 40 years (1). Even after radical resection, iCCA is still very prone to recurrence and metastasis (2). The five-year survival rate of iCCA is 9% (3), which is much worse than that of hepatocellular carcinoma (HCC). Adjacent-to-tumor tissues (ATTs) have generally been analyzed as control samples in previous cancer research (4–7). With the increasing understanding, ATTs are considered to be an intermediate state between normal and cancerous tissues through a comprehensive transcriptome analysis (8). There are genomic instability and early-stage cancer evolution in ATTs of cancer at spatial transcriptome level (9). Moreover, gene expression profile of ATTs was highly correlated with the survival of HCC patients (10–13). Strikingly, ATTs exhibited superior predictive value for clinical outcomes and recurrence compared to tumor tissues in both lung adenocarcinoma and colorectal cancer patients (14, 15). However, there remains a lack of recurrence prediction models based on multi-omics data derived from ATTs in iCCA.

Proteomics has played a pivotal and unique role in molecular subtyping of both tumors or ATTs (6, 13, 16, 17), offering novel insights and strategies for precise classification, personalized treatment, therapeutic monitoring, and prognostic assessment. While proteomic analysis of ATTs shows promise for developing recurrence predictive models, systematic proteomic study of iCCA ATTs is notably absent. A novel and efficient proteomic-based recurrence risk prediction model is urgently needed to guide postoperative treatment of iCCA.

**Figure 1:**
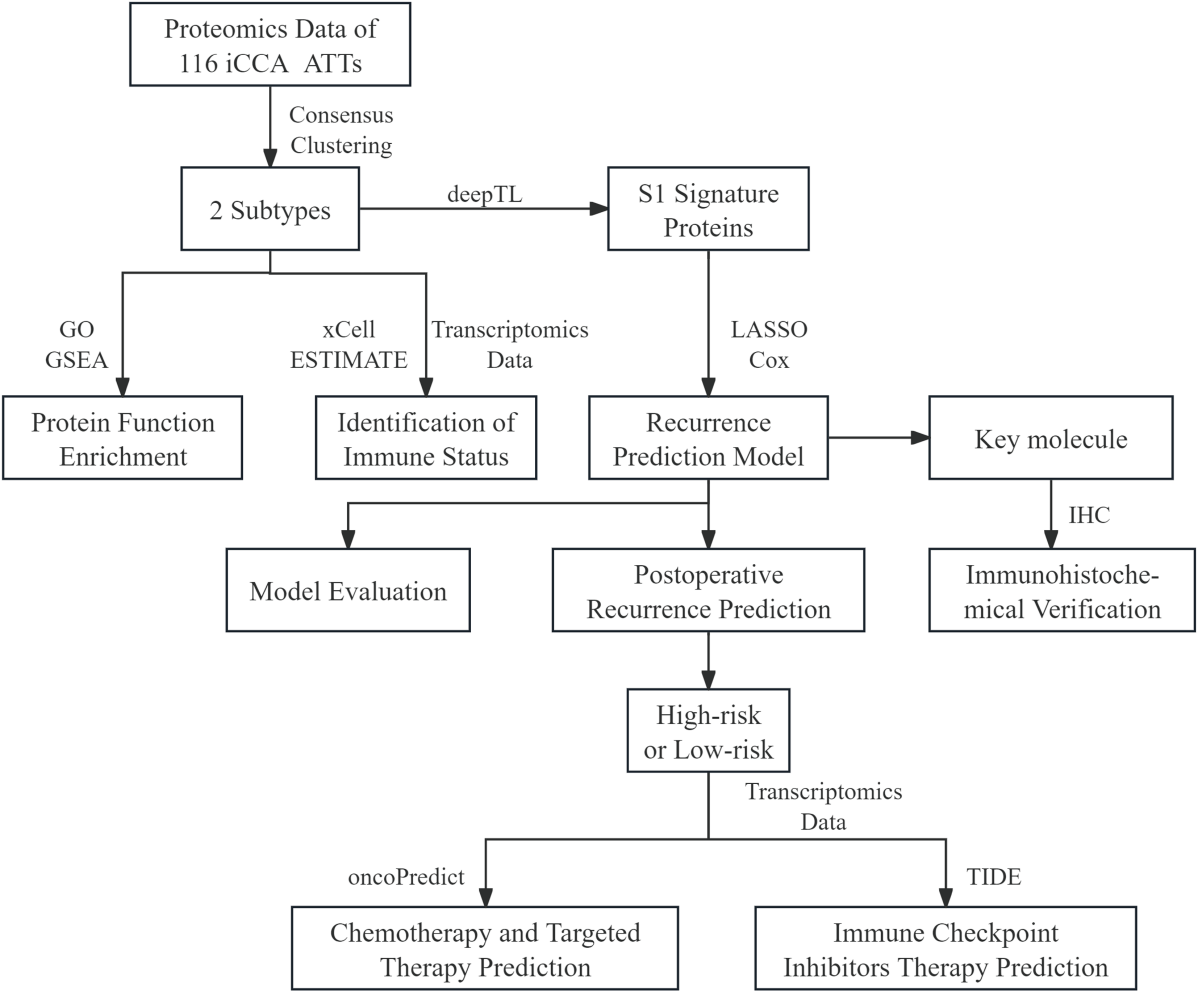
Outline of the study design. A dataset containing gene expression and proteomics data from 116 intrahepatic cholangiocarcinoma patients and relevant clinical information was utilized for molecular classification and subtype characterization. We conducted protein functional enrichment analysis and immune status identification based on the two subtypes. We screened for signature proteins of the S1 by using deepTL and employed LASSO and Cox regression to construct a recurrence prediction model. The model was evaluated, and immunohistochemical validation was performed on the key molecule POSTN. We utilized transcriptomic data to predict drug treatment responses based on the group derived from the predictive model. S1, subtype 1; GO, gene ontology; GSEA, gene set enrichment analysis; POSTN, periostin; LASSO, least absolute shrinkage and selection operator; IHC, immunohistochemistry; TIDE, tumor immune dysfunction and exclusion.

In this study, we conducted molecular subtyping at proteomic level and identified the immune status of different subtypes of iCCA ATTs. Furthermore, we constructed, evaluated and validated a multi-factor Cox regression prediction model for recurrence of iCCA. Finally, we conducted drug response prediction based on the risk stratification determined by the predictive model (**Figure 1**).

## 2 Materials and methods

### 2.1 Patient cohort collection

We collected tissues from 116 iCCA patients who underwent liver resection prior to receiving any anticancer therapy and matched adjacent liver tissues. Each patient received a pathological diagnosis from the department of pathology at Zhongshan Hospital (Shanghai, China), which was confirmed as iCCA and follow-up ended in December 2022. All tissue samples were collected according to standardized protocols. All patients included in the study signed informed consent forms allowing the use of their data and tissue samples. The Ethics Committee of Zhongshan Hospital reviewed and approved the use of all human tissues involved in the study (B2021-611). The baseline clinicopathological features of the 116 iCCA patients are presented in **Supplementary Table S1**.

### 2.2 Transcriptomic and proteomic sequencing, quality control and quantification

The transcriptome and proteome quantification of the ATT samples of the 116 iCCA patients were performed using high-throughput RNA-sequencing (RNA-seq) and mass spectrometry (MS) based shotgun proteomics approaches, respectively. Principal component analysis (PCA) was performed using R software (version 4.0.4) to evaluate batch effects in the proteomics dataset associated with the following two variables: batch identifier and sample type (tumor versus non-tumor) (6).The methods for RNA-seq and MS data generating, processing and quality control were the same as those used in the analysis of the corresponding tumor samples (16). We used the intensity-based absolute quantification (iBAQ) values for further proteomics analysis (**Supplementary Table 2**), with a quantile normalization and log2 transformation. We selected proteins that were expressed in at least 50% of the samples for further analysis, with missing values filled in as 0 (18, 19).

### 2.3 Classification of molecular subtypes and survival analysis

Molecular classification was conducted through consensus clustering (20), with a subsample size of 1,000 and a sampling proportion of 0.8. Partitioning around medoids (PAM) algorithm and euclidean distance were used for clustering. The optimal K value was 2. Kaplan-Meier curves and log-rank tests were used for estimating survival. The endpoint of the study was overall survival (OS). OS was defined as the interval between the date of diagnosis and the date of patient death or the last follow-up. Disease-free survival (DFS) was defined as the interval between the date of surgery and the date of disease recurrence or death for any reason or the last follow-up (whichever occurs first).

### 2.4 Differential expressed proteins and function enrichment analysis

Differential expressed protein (DEP) analysis was performed using limma (21), with the selection criterion being an absolute logFC value greater than 1 and a P-adjusted value less than 0.05. The over/under-representation analysis was performed based on the hypergeometric distribution model, and the *P* values were corrected using the Benjamini-Hochberg method. The GO annotation file was downloaded from https://geneontology.org/, version (date-generated 2023-11-16). KEGG pathway annotation was obtained from the website of DAVID (22) (https://davidbioinformatics.nih.gov/). The GSEA analysis (23) was conducted using the clusterProfiler R package, using org.Hs.eg.db for gene annotation.

### 2.5 Immune infiltration analysis and drug reaction prediction

Estimation of the relative abundance of different types of immune cells in tissue samples was performed using the xCell (24). The ESTIMATE (25) score was used to evaluate the immune or stromal score of the samples. OncoPredict (26) was used to predict the response to chemotherapy and targeted therapy, and the TIDE (27) score was used to predict the response to immune checkpoint inhibitors.

### 2.6 Signature protein selection and development of recurrence prediction model

The permutation-based feature importance test (PermFIT) in the deepTL package was proposed to help explain individual features in complex frameworks, including support vector machine (SVM) and deep neural networks (DNN). Deep learning algorithm PermFIT (28) was used for subtype-specific signature proteins selection. Here, we used the differential genes set of S1 as the input matrix and used the PermFIT-DNN and PermFIT-SVM algorithms to identify 38 and 52 signature proteins, respectively. Given the presence of multicollinearity among variables or when the number of variables exceeds the sample size, it was necessary to first conduct variable selection using Least Absolute Shrinkage and Selection Operator (LASSO) regression, followed by the construction of a Cox regression model to assess prognostic implications (29, 30). Signature proteins selected by PermFIT-DNN were subsequently subjected to rigorous variable selection using LASSO logistic regression (glmnet R package) (31), with penalty parameters optimized through iterative contour regression analysis. After 10-fold cross-validation, the optimal lambda (λ.min) was selected. When coefficient was not equal to 0, the number of feature proteins in the model was determined by the number of the most common proteins. The selected variables were first included in univariate Cox regression analysis (32, 33), and then the variables with *P* values less than 0.05 were included in multivariate Cox regression analysis. The multivariate Cox proportional hazards regression was performed using stepwise backward selection of variables (34).

### 2.7 Validation of the recurrence prediction model

We used the ‘caret’ package for 10-fold cross-validation to validate the model and obtain the optimal parameters. In terms of performance evaluation, we used the time-dependent area under the curve AUC curve to evaluate the discriminatory ability of the prediction model, and used the calibration curve to evaluate the accuracy of the prediction model. To measure the clinical utility and discriminatory ability of the model, we also used the decision curve analysis (DCA) curve (35) to evaluate the detection efficacy of the prediction model compared with the clinical TNM staging.

### 2.8 Tissue microarray experiment and immunohistochemical validation

A TMA containing 88 iCCA ATTs samples (another independent cohort) was established (**Supplementary Table 3**). Immunohistochemical (IHC) staining was conducted with IHC reagents from Fuzhou Maixin Biotech Co., Ltd. (Fuzhou, Fujian, China). In brief, TMA underwent deparaffinization and rehydration prior to antigen retrieval. Subsequently, it was blocked using a solution containing 3% hydrogen peroxide and 1× Animal-Free Blocking Solution. Then, the sections were incubated with primary antibodies (POSTN, 19899-1-AP, Proteintech Group, Rosemont, IL, USA) overnight at 4°C. The next day, the sections were incubated with the secondary antibody at 37°C for 1 h, and 3,3 N-diaminobenzidine tetrahydrochloride (DAB) staining was conducted. Finally, they were counterstained with hematoxylin for 2 min.

Using the PANNORAMIC 1000 slice scanner (3DHISTECH Ltd., Budapest, Hungary), the tissue sections were scanned and imaged. CaseViewer analysis software (3DHISTECH Ltd.) was used to calculate the immunoreactive-score (IRS) of the target area. IRS was a combination of the intensity (0 to 3 points) and proportion of positive cells (0 to 4 points). The IRS was calculated by multiplying two parameters: Staining Intensity (SI), Scored on a scale of 0-3, 0: No staining, 1: Weak staining, 2: Moderate staining, 3: Strong staining; Percentage of Positive Cells (PP), scored on a scale of 0-4, 0: 0% positive cells, 1: 0<PP ≤ 10%, 2: 10<PP ≤ 50%, 3: 50<PP ≤ 80%, 4: 80<PP ≤ 100%. Final IRS = SI × PP, resulting in a total score ranging from 0 to 12. The median IRS was defined as the threshold value to distinguish the low and high POSTN expression groups.

## 3 Results

### 3.1 Molecular subtyping and functional feature analysis of iCCA ATTs

We collected a total of 116 iCCA patients’ tumor samples and paired ATT samples. The patients had never received any anti-cancer therapy (such as radiation, immunotherapy or chemotherapy) before surgery. Prior to MaxQuant database searching, we assessed batch effects between tumor tissues and paired adjacent-to-tumor tissues through PCA. We focused primarily on the first two principal components in the PCA (**Supplementary Figure 1A**). The analysis results demonstrated that batch effects had a negligible impact on inter-sample variation, whereas sample type showed significant effects. Cumulative recurrence was observed in 61 patients (52.6%) by the end of December 2022, and overall clinical information was shown in **Supplementary Table 1**. RNA sequencing and proteomic profiling were performed on tumor tissues and ATTs from 116 iCCA patients.

**Figure 2:**
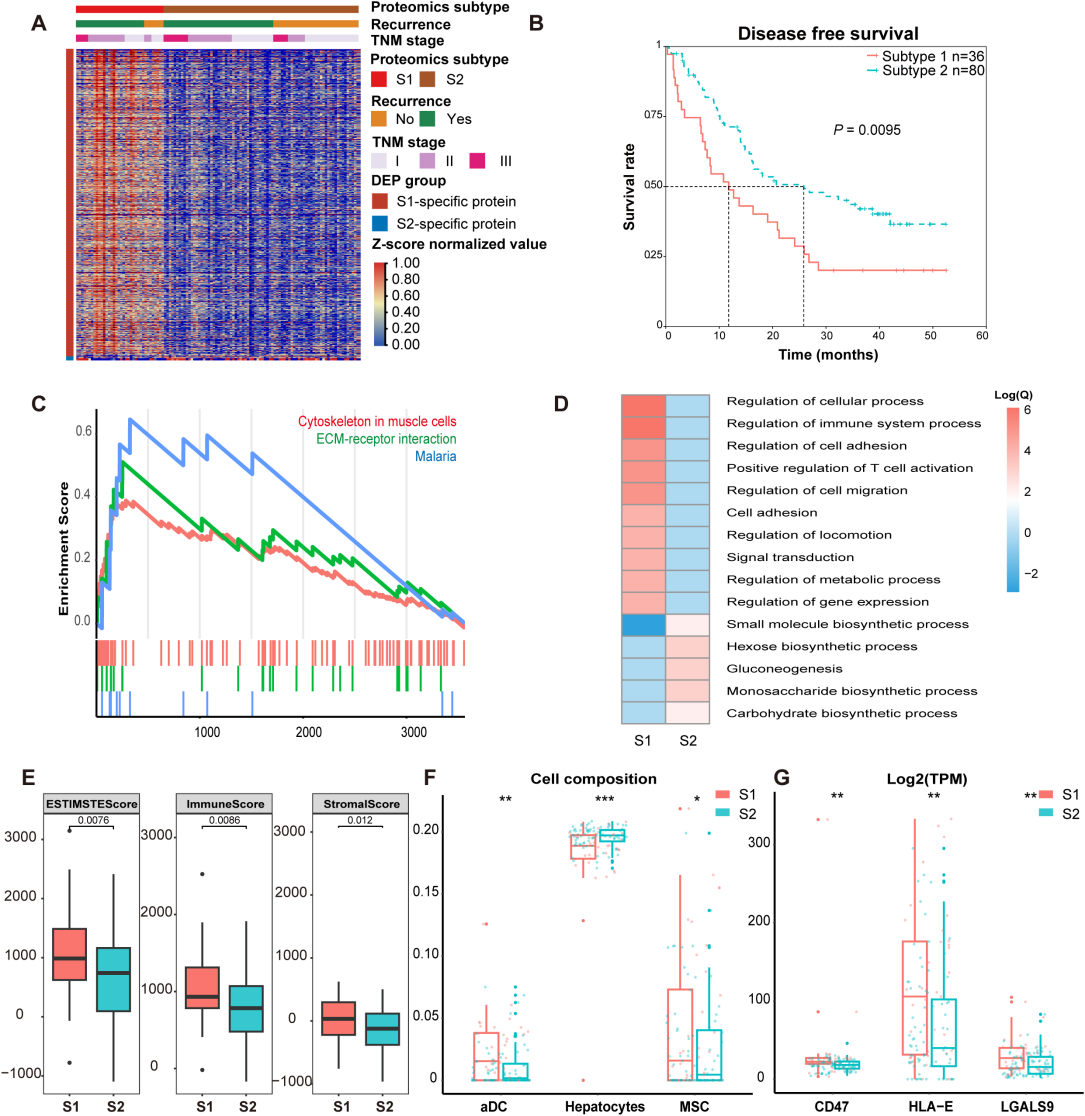
The distinct features of the two ATT subtypes. **(A)** Heatmap of clinical stages and differential protein expression levels (Z-score normalized) across different subtypes. **(B)** This part presented the Kaplan-Meier survival curves, illustrating the probability of survival over time for different patient groups, with disease-free survival (DFS) as the primary endpoint of interest (log-rank test). **(C)** GSEA analysis of differentially expressed proteins among ATT subtypes. **(D)** Heatmap of GO functional enrichment terms for differentially expressed proteins among ATT subtypes. **(E)** Comparison of ESTIMATE scores among ATT subtypes (Wilcoxon rank-sum test). **(F)** Determination of relative cellular composition differences among ATT subtypes using xCell (Wilcoxon rank-sum test). **(G)** Differential transcriptional expression levels (Log2(TPM)) of immune-related genes among ATT subtypes (Wilcoxon rank-sum test). TPM, transcripts per million. *P < 0.05, **P < 0.01, ***P < 0.001.

We used the proteomic data of iCCA ATTs to perform consensus clustering analysis (**Supplementary Figures 1B–D**). The iCCA ATTs were divided into two subtypes that 36 cases were classified into subtype 1 and 80 cases into subtype 2 (subtype 1 and subtype 2 were abbreviated as S1 and S2, respectively) (**Figure 2A**). By comparing with classical proteomic subtypes of tumor tissues, we observed that tumor subtypes and adjacent non-tumor tissue subtypes did not correspond one-to-one (**Supplementary Figure 1E**). Instead, a subset of cases within the tumor subtypes clustered into a distinct adjacent tissue subtype, suggesting that the adjacent non-tumor tissues harbor important early alterations that influence patient prognosis, particularly recurrence. Through comparative analysis of clinical prognostic information, we found that the S1 had a recurrence rate of 78%, while the S2 had a recurrence rate of 56%, with a statistically significant difference between them (Chi-square test, *P* = 0.026358). However, no statistically significant difference was observed in the composition of TNM stages between the subtypes (Fisher’s exact test, *P* = 0.168). These findings suggested that ATTs has provided important prognostic information, particularly regarding recurrence. The Kaplan-Meier curve showed that the prognosis of subtype S1 was worse than subtype S2 (log-Rank test, *P* = 0.014) (**Figure 2B**, **Supplementary Figure 1F**). Proteomic profiling revealed 970 upregulated and 13 downregulated proteins in S1 compared with their expression in S2 (**Supplementary Figure 1G**; **Supplementary Table 4**).

GO enrichment analysis revealed that the upregulated proteins of S1 were primarily enriched in key regulatory processes, including the regulation of nitrogen compound metabolism, development, stimulus response, signal transduction, and multicellular organismal processes; Conversely, the downregulated proteins of S1 were associated with small molecules biosynthesis, glucose metabolism, extracellular exosomes, chromatin structural organization (**Supplementary Table 5**). KEGG pathway enrichment analysis showed that the upregulated proteins in S1 were predominantly associated with immune-related pathways, including antigen processing and presentation, the NOD-like receptor signaling pathway, the B cell receptor signaling pathway, the chemokine signaling pathway, and regulation of the actin cytoskeleton. In contrast, the downregulated proteins remained enriched in metabolic pathways, particularly glycolysis/gluconeogenesis (**Figure 2D**). These implied that S1 harbored the molecular foundation for transformation from ATTs to tumor, whereas S2 demonstrated functional features closer to normal liver tissues. Meanwhile, via GSEA analysis based on the differential proteins of subtypes (**Figure 2C**), we found that extracellular matrix receptor interaction pathway played the most important role in tumor cell proliferation, survival, and invasive capacity (36, 37).

In summary, we identified the subtype S1 of iCCA ATTs associated with poor prognosis and recurrence. At the same time, we found that the S1 was more enriched in the regulatory process based on functional enrichment analysis, suggesting the changes in S1 exhibited some tumorigenic transformation.

### 3.2 Comparison of immune status of iCCA ATT subtypes

We explored the immune scores and stromal scores among different iCCA ATT subtypes. The immune scores and stromal scores of S1 were higher than those of S2, while no differences in immune or stromal scores were found in the paired tumor tissues (**Figure 2E**; **Supplementary Table 6**). We investigated the relative abundance of immune cells in different iCCA ATT subtypes (**Figure 2F**). We found that there were differences in the composition of immune cells among the iCCA ATT subtypes. The proportion of activated dendritic cells (aDC), plasmacytoid dendritic cells (pDC) and mesenchymal stem cells (MSC) in S1 were higher than those in S2, while the proportion of hepatocyte in S2 was higher than that in S1(**Figures 1H**, **2F**; **Supplementary Table 7**). However, we did not find differences in the relative abundance of CD4^+^ or CD8^+^ T cells between S1 and S2. This result suggested that S1 ATTs were in more active immune state. It suggested that the subtype of dendritic cells may regulate T cell function to promote the formation of liver tumor cells. Our findings suggested that the changes of dendritic cells in S1 may serve an immunomodulatory role by regulating T cells function, ultimately promoting tumor relapse (38).

We further compared the immune checkpoint related genes among different subtypes and found there were differences in three genes: CD47, HLA-E and LGALS9. The expression level of these genes in S1 were much higher than those in S2 (**Figure 2G**). In particular, the gene expression level of HLA-E was higher in S1 than in S2. Recent studies have found that HLA-E and CD94-NKG2A have the strongest immune interaction between circulating tumor cells (CTC) and natural killer cells (39). Mechanistically, the observed HLA-E overexpression in S1 may enhance CTC-NK cell interaction, leading the impairment of immune surveillance capacity.

### 3.3 Screening of subtype signatured proteins and construction of recurrence prediction model

**Figure 3:**
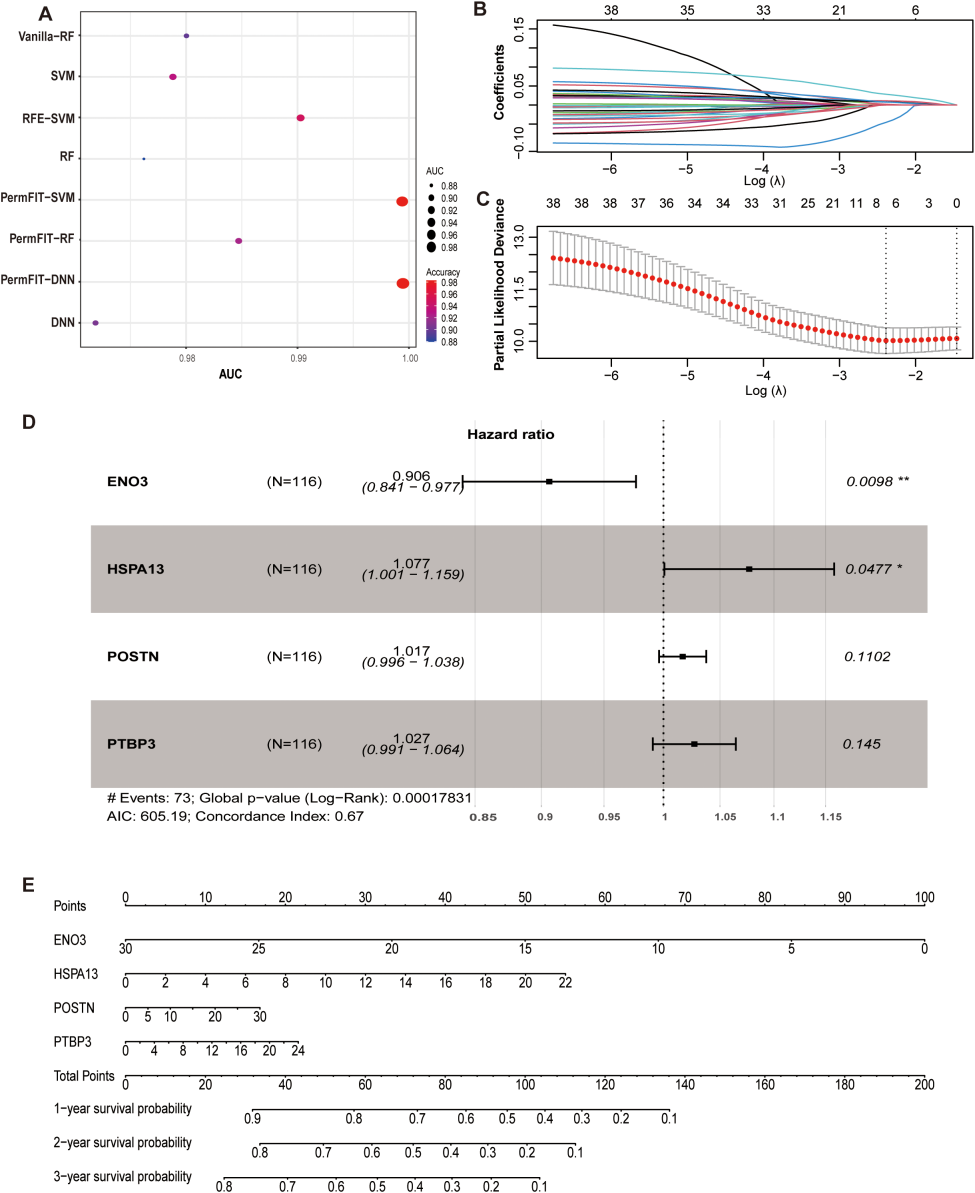
Signature protein screening and construction of recurrence prediction model. **(A)** Signature protein screening for ATT S1 was conducted using PermFIT in conjunction with Deep Neural Networks (DNN), Support Vector Machines (SVM), and Random Forest (RF) algorithms. The Area Under the Curve (AUC) and accuracy (ACC) metrics are shown. **(B)** LASSO regression coefficient path plot using the least angle regression (LARS) method. The upper x-axis represents the number of feature proteins at different λ values, while the lower x-axis represents the logarithm of the penalty coefficient λ. The weight coefficients of certain features rapidly decrease to zero as λ gradually increasing. Key variables are selected by controlling the value of λ. **(C)** Cross-validation curve for LASSO regression. The y-axis represented the cross-validation error (smaller errors indicated better fitting). It can be observed that eight feature proteins were selected when the error was minimized in this study. **(D)** Forest plot of a multivariate Cox regression model was constructed based on four protein molecules. When the hazard ratio (HR) and its 95% confidence interval (CI) for a variable do not intersect the dashed line, it is considered that the variable has a statistically significant impact on patient recurrence outcomes. HR < 1 indicated that the variable was a protective factor, while HR > 1 indicating risk factors. The molecule ENO3 was shown to be a protective factor, whereas the molecule HSPA13 was a risk factor. **(E)** Based on the contributions of the four protein molecules to recurrence outcomes in the model, scores were assigned to each protein’s values, which were then summed to obtain a total score. The predicted probability of recurrence events for an individual was calculated through a functional transformation relationship between the total score and the probability of recurrence events occurring. *P < 0.05, **P < 0.01, #, Hazard ratio.

We used the deepTL tool to test eight methods of screening subtype signature proteins. The algorithm combination of PermFIT-DNN had the best performance with an accuracy of 0.98 and an AUC of 0.99. We selected 38 proteins screened by the PermFIT-DNN algorithm as the S1-signatured proteins, and these 38 proteins were composed of proteins with high and low expression in S1 ATTs (**Figure 3A**; **Supplementary Table 8**).

In order to make the prediction model simpler and more effective, we firstly used LASSO regression to reduce the number of signature proteins which are important for iCCA tumor recurrence. By cross-verifying the curve, we took the value of λ.min as the number of variables included in the equation (**Figure 3B**). A total of 8 proteins were included, namely ENO3, GMIP, HSPA13, MANBA, PLOD2, POSTN, PTBP3, and TBC1D5 (**Figure 3C**; **Supplementary Table S9**).

Next, the stepwise regression method was used to construct the multivariate Cox proportional risk regression model. As the result, there are four proteins involved in the model, including ENO3 (HR, 0.91; 95% CI, 0.84-0.98), HSPA13 (HR, 1.08; 95% CI, 1.00-1.16), POSTN (HR, 1.02; 95% CI, 1.00-1.04) and PTBP3 (HR, 1.03; 95% CI, 0.99-1.06) (**Figure 3D**). After determining the coefficients, we used the following formula for this four-protein model to calculate the risk score (**Figure 3E**):

Risk score= 0.0740 × PA_HSPA13_ + 0.0166 × PA_POSTN_ + 0.0266 × PA_PTBP3_ − 0.0985 × PA_ENO3_, where PA means protein abundance.

### 3.4 Evaluation and validation of recurrence prediction model

**Figure 4:**
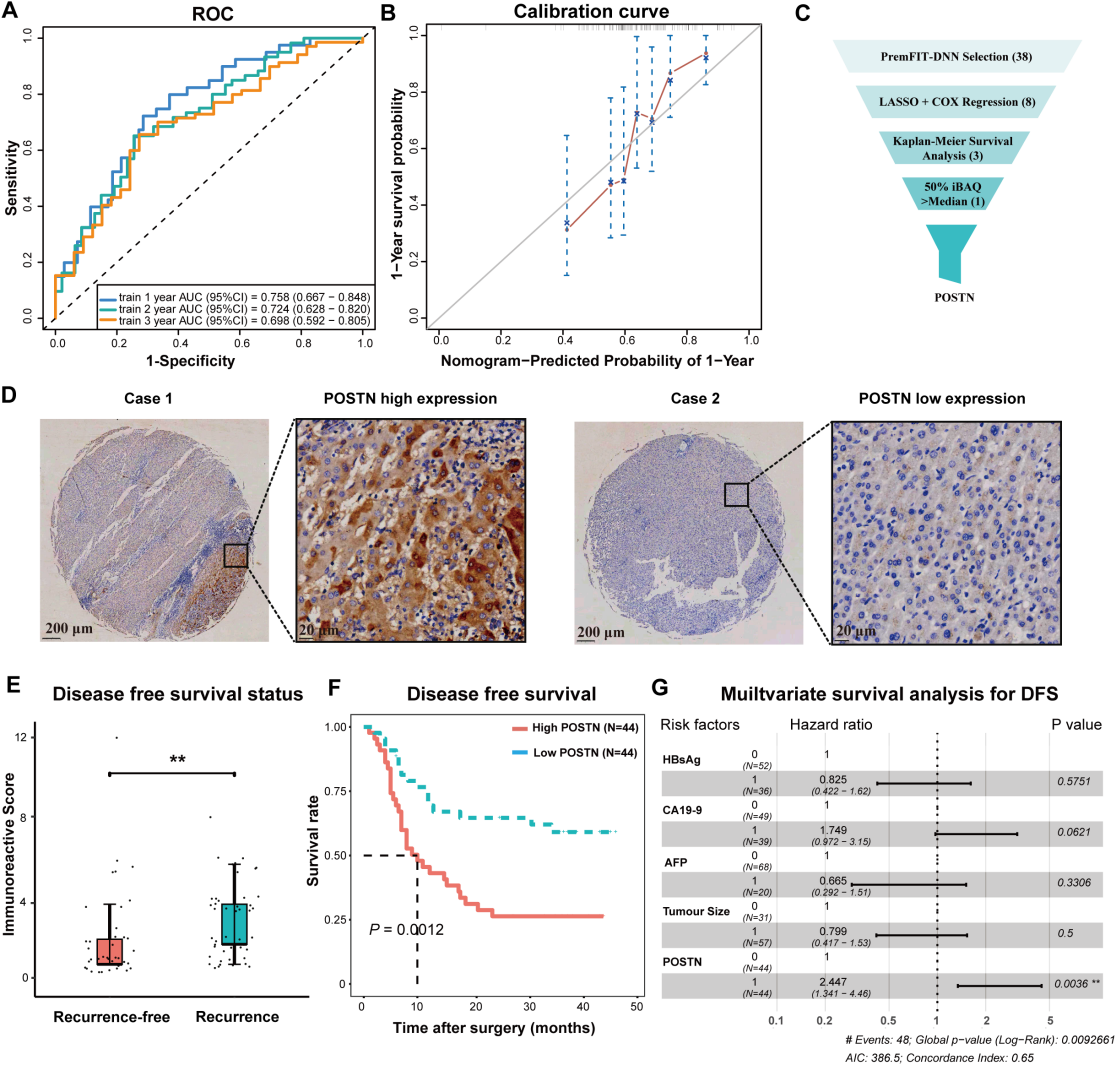
Evaluation of the recurrence prediction model and immunohistochemical validation of key molecule. **(A)** The area under the receiver operating characteristic (ROC) curve (AUC) was used to assess the discriminatory ability of the recurrence prediction model. **(B)** The calibration curve, presented as a scatter plot of actual recurrence incidence versus predicted recurrence incidence, illustrates the model’s calibration accuracy. The closer the curve is to the diagonal line, the higher the model’s calibration degree. This figure depicted the calibration curve for predicting post-surgery recurrence after one year. **(C)** Screening process for key molecules including iBAQ values selection and univariate survival curve analysis. **(D)** This part displayed immunohistochemical staining of the POSTN molecule. Case 1 represented high expression of POSTN on ATT tissue microarrays, while Case 2 depicted low expression of POSTN on ATT tissue microarrays. Scale bar was 200μm&2μm. **(E)** Comparison of immunohistochemical scores of POSTN with respect to recurrence outcomes. **(F)** Survival curve analysis was conducted for recurrence outcomes between the high-expression and low-expression groups of POSTN. **(G)** Multivariate survival analysis was performed for recurrence outcomes between the high-expression and low-expression groups of POSTN. **P < 0.01, #, Hazard ratio.

We evaluated the performance of the prediction model in terms of both differentiation and calibration. We tested the model’s consistency index (C-index), which is 0.67, to measure the differentiation of the prediction model. We also plotted the area under the receiver operating curve (AUC) to compare the performance of the predictive model. The AUC for one-year recurrence prediction reached 0.758 (95% CI, 0.667-0.848), the AUC for two-years recurrence prediction reached 0.724 (95% CI, 0.628-0.820) and the AUC for the three-years was 0.689 (95% CI, 0.592-0.805) (**Figure 4A**). To evaluate the generalization ability of the relapse prediction model, we performed an internal 10-fold cross-validation. The mean value of AUC reached 0.7177(range: 0.6746-0.7854) and the mean value of C-index reached 0.6749(range: 0.6447-0.7142).

In addition, we used calibration curves to measure the ability to predict the calibration degree of the model. The calibration curve showed that the survival results predicted by the model are in good agreement with the actual survival results, which confirming the good fitting degree of the predicted model (**Figure 4B**; **Supplementary Figures 3A, B**). We also performed decision curve analysis (DCA) to further compare the clinical benefit of the predictive model with tumor node metastasis classification (**Supplementary Figure 3C**). Receiver operating curve (ROC) and decision curve analysis (DCA) showed that the predictive model had better predictive ability and higher clinical application value than the traditional TNM classification.

### 3.5 Immunohistochemical validation of POSTN

As mentioned above, we constructed a recurrence prediction model containing ENO3, HSPA13, POSTN and PTBP3. Furthermore, we conducted internal validation and evaluation. To further verify the role of the molecules which made up the prediction model, we attempted immunohistochemical validation on iCCA ATTs samples. We used univariate survival analysis to select molecules with *P* value less than 0.05, resulting in a total of 189 molecules. We further selected molecules with higher expression levels in the proteomic data cohort. Ultimately, we decided to conduct immunohistochemical validation of the POSTN molecule in tissue microarray, to investigate its expression level in iCCA ATTs samples and its relationship with survival and recurrence (**Figure 4C**). This molecule, also identified as the peritendinous extracellular matrix protein, demonstrates significant associations with tumor metastatic potential and prognosis.

We observed heterogeneous POSTN expression in this tissue microarray which was composed of 88 iCCA ATT samples (**Figure 4D**). We utilized the immunoreactive score (IRS) for semi-quantitative immunohistochemical evaluation, and discovered that the scores of POSTN molecules differed between recurrent patients and non-recurrent patients, with a higher IRS of POSTN molecules in recurrent patients (**Figure 4E**). Simultaneously, we also observed that the IRS of POSTN molecules was higher in deceased patients than in surviving patients (**Supplementary Figure 3D**). These indicated that POSTN molecules played a certain role in predicting the recurrence of iCCA patients.

We classified the tissue microarray samples into high-expression and low-expression groups based on the average optical density (AOD) and conducted the analysis of survival curves (**Figure 4F**; **Supplementary Figure 3E**). The prognosis of tissue microarray samples with high expression of POSTN was poorer than those of low expression. In the multivariate survival analysis, the expression of POSTN was an independent prognostic factor for survival and recurrence in iCCA patients (**Figure 4G**; **Supplementary Figure 3F**). This finding also verified the validity of our four-protein recurrence prediction model from another perspective.

### 3.6 Drug reaction prediction based on recurrence prediction model

**Figure 5:**
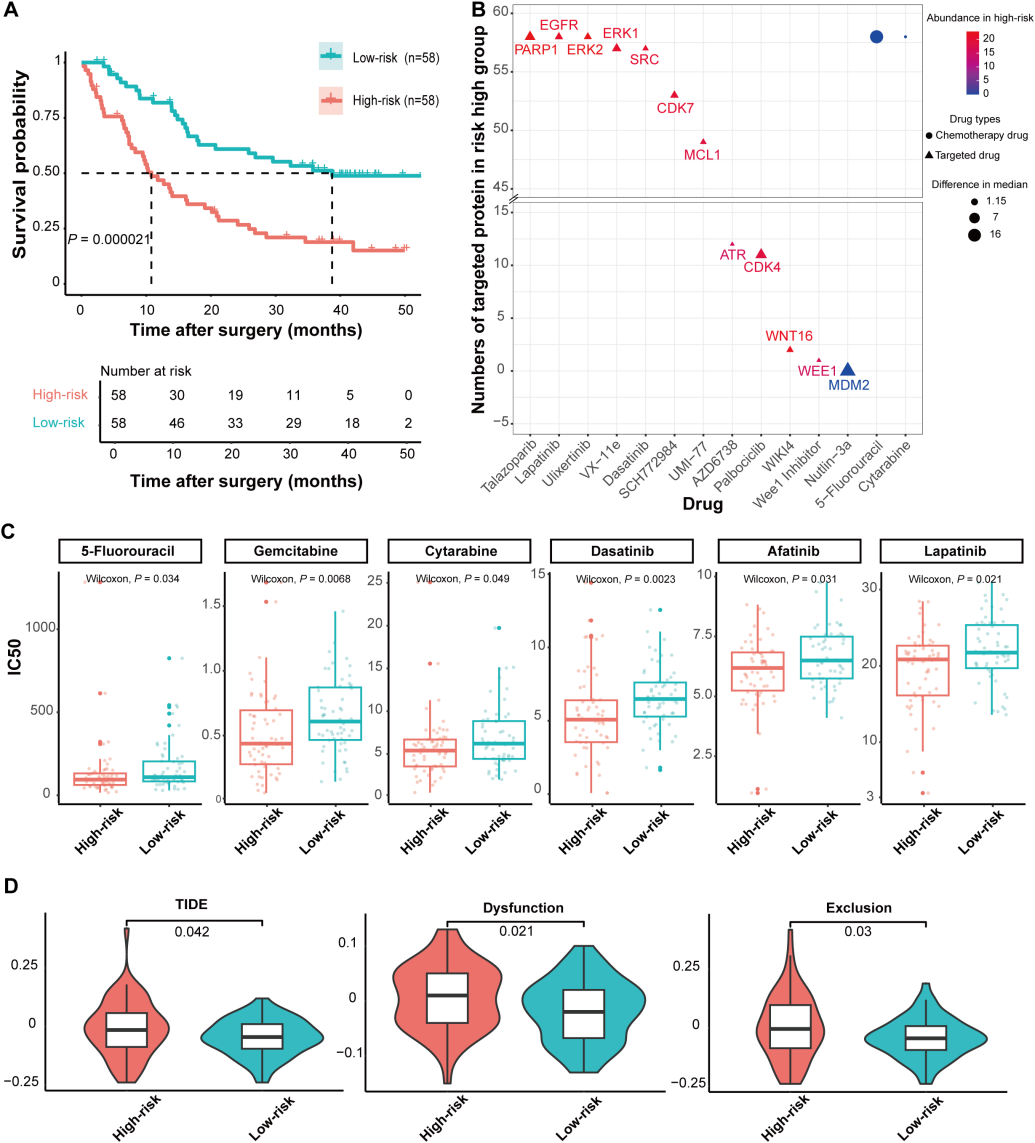
Prediction of drug treatment response based on risk groups from the predictive model. **(A)** Utilizing the risk prediction values output by the predictive model, ATT cohort samples were stratified into high-recurrence and low-recurrence risk groups based on the median risk prediction value. Survival curve analysis revealed that samples in the high-recurrence risk group were more prone to recurrence. **(B)** The x-axis represented the types of targeted and chemotherapeutic drugs, while the y-axis indicated the number of samples with drug targets in the high-risk group. The size of the graphical elements denoted the absolute value of the median difference in IC50 values between the high-risk and low-risk groups. All drugs displayed in the figure exhibit lower IC50 values in the high-recurrence risk group compared to the low-recurrence risk group. IC50, half maximal inhibitory concentration. **(C)** Representative drugs showing significant IC50 differences between the high-recurrence and low-recurrence risk groups are presented (Wilcoxon rank-sum test). **(D)** Prediction of immunotherapy response and comparison of T cell dysfunction and immune exclusion scores between the high-recurrence and low-recurrence risk groups (Wilcoxon rank-sum test).

In addition, we stratified 116 samples into high-risk and low-risk group by the median risk score which was derived from recurrence predictive model. Survival analysis between the high-risk and low-risk groups showed that the prognosis of iCCA patients deteriorated as the risk value increased, with a significant difference in prognosis (p < 0.0001) (**Figure 5A**). Patients in the low-risk group had a better prognosis than those in the high-risk group.

We further investigated whether there were differences in the response to chemotherapy, targeted therapy, and immune checkpoint treatment between the two risk groups. We predicted the response to 198 drugs including chemotherapy drugs and targeted therapy drugs by the transcriptome matrix of iCCA ATTs. Our analysis revealed that 35 drugs showed significant differences in response between the high-risk and low-risk group (**Figure 5B**). Unexpectedly, 34 drugs exhibited lower IC50 values in the high-risk group compared to the low-risk group, suggesting that samples in the high-risk group were more sensitive to these treatments. Additionally, we identified lapatinib, talazoparib, and dasatinib as potential targeted therapy options for postoperative recurrence of iCCA by integrating the expression levels of the target proteins of these targeted therapies in the high-risk group (**Figure 5C**; **Supplementary Table 10**). Chemotherapeutic agents such as 5-fluorouracil, gemcitabine, and cytarabine were also identified as potential chemotherapy options for postoperative recurrence of iCCA.

We predicted the response to immune checkpoint inhibitor therapy in the two groups and found that the tumor immune dysfunction and exclusion (TIDE) scores of high-risk groups were higher than those of low-risk groups which suggested that samples in the high-risk groups are less likely to respond to immune checkpoint inhibitor therapy than those in the low-risk groups (**Figure 5D**, **Supplementary Table 11**). The T cell dysfunction and T cell exclusion scores exhibited a similar distribution to the TIDE score. This indicated that the high-risk groups also harbored two mechanisms that counteract the efficacy of immune checkpoint inhibitors. These findings suggested that samples in the high-risk groups may be more sensitive to chemotherapy and targeted therapies but have a poor response to immune checkpoint inhibitor therapy, providing a basis and reference for treatment selection in patients with postoperative recurrence.

## 4 Discussion

In recent years, molecular classification has gained increasing attention and widespread application in oncological research and clinical practice, constituting a pivotal component of precision medicine. Transitioning from histological to molecular classification systems has fundamentally facilitated precision medicine in oncology (40). However, there are still many difficulties in clinical prediction on postoperative recurrence based solely on tumor tissue omics analysis. Traditional perspectives often regard “adjacent non-tumor tissue” merely as a negative control for surgical margins. However, modern tumor biology views adjacent-to-tumor tissues as dynamic, heterogeneous ecosystems that played multifaceted roles in tumor recurrence. The ATTs provide the macroscopic tumor microenvironment for postoperative recurrence. The relationship between adjacent-to-tumor tissues and tumor recurrence fundamentally involves dynamic host-tumor interactions. Elucidating this process is crucial for developing strategies to prevent and control tumor recurrence. Accordingly, utilizing ATTs expression to predict response to systemic therapy may hold theoretical promise. Moreover, there are many uncharacterized biological alterations that influence patient prognosis and recurrence in ATTs, which providing a theoretical basis for predicting postoperative recurrence (41, 42). Therefore, analyzing the proteomics data from iCCA ATTs may yield favorable results. We found that the subtype S1 had a higher rate of recurrence than S2, suggesting ATTs played a vital role in the prediction of recurrence after tumor resection. We speculate that ATTs provided growth environment for tumor recurrence. The results that samples of S1 exhibiting higher immune and stromal scores suggested that there may be some early immune activities that potentially influenced tumor recurrence in the samples of S1. In contrast, no such differences in immune and stromal scores were found in the tumor samples paired with the iCCA ATTs. This also highlighted that certain changes have existed in the ATTs that are not present in the tumor tissues.

The immune cells played a key role in tumor microenvironment (TME) (43). Immune cells can recognize and destroy newborn tumor cells during cancer immune monitoring under normal circumstances. But this may be affected by different factors during cancer immune reprogramming. The fact that immune cells acted as guardians (anti-tumor immunity) or bystanders or supporters of tumors (pro-tumor immunity) made them a “double-edged sword” in TME (44). We found differences in immune scores between the ATTs subtypes not in the paired tumor groups suggesting that ATTs immune activity may be relatively more active. The subtype-specific variations in dendritic cells (DC) abundance implied a potential role of DC-mediated antigen presentation in ATTs in tumor relapse (45). In our study, we observed significant differences in immune status among ATTs subtypes, with the recurrence-associated S1 exhibiting a tendency toward immune infiltration. Although traditional perspectives have suggested that immune infiltration predicts better therapeutic outcomes, this conclusion has largely been drawn from studies focusing on tumor tissues. Furthermore, our study revealed increased expression of immune checkpoint-related genes in the S1 adjacent tissue subtype. HLA-E binds to the inhibitory receptor NKG2A on the surface of NK cells and T cells, directly suppressing the cytotoxic functions of these cells (39). CD47 interacts with SIRPα on macrophages, transmitting a signal that inhibits macrophage-mediated phagocytosis of tumor cells (46, 47). LGALS9 binds to receptors such as Tim-3 on T cells, ultimately inducing CD8+ T cell exhaustion while enhancing the suppressive activity of regulatory T cells (48). The elevated expression of these three genes collectively establishes and maintains an immunosuppressive tumor microenvironment by targeting multiple critical mechanisms: inhibiting cytotoxic immune cells (NK cells, T cells), blocking innate immune clearance (macrophages), and inducing immune cell exhaustion. This further supported that the immune infiltration observed in the S1 adjacent tissue subtype may represent a pro-tumor phenotype.

The POSTN gene, also known as periostin, was located on chromosome 13q13.3 and encoded a protein belonging to the Gla domain-containing family (49), whose members were typically involved in the regulation of the extracellular matrix (ECM) and cell-cell interactions. POSTN protein was expressed in various tissues, with particularly high expression in connective tissues and certain tumors. In relevant immunohistochemical experiments, the expression level of this molecule was not high in normal liver tissues. However, in our omics analysis, we found that the expression level of POSTN in the iCCA ATTs of S1 was more than two-fold higher than those in S2. As a component of the extracellular matrix, POSTN protein contributed to maintaining tissue structure and strength. It can enhance cell-cell adhesion and promoted cell migration, playing crucial roles in wound healing and embryonic development. POSTN protein can initiate intracellular signaling pathways, influencing cell growth, differentiation, and survival by binding to multiple receptors. High expression of POSTN was associated with increased tumor aggressiveness and metastatic potential in various cancers such as lung cancer, breast cancer, and colon cancer (50). POSTN protein can bind to integrin receptors, activating intracellular signaling pathways such as the FAK and ERK pathways, which were vital for cell migration and survival (50). POSTN from preoperative serum can serve as an independent biomarker reflecting the prognosis of liver cancer patients (51, 52). In our study, we also found that POSTN can serve as an independent prognostic and recurrence biomarker for iCCA. However, the underlying mechanisms, such as whether the carboxylation status of POSTN affected its action on target tissues, remained unclear and warrant further investigation.

Clinical predictive models can be used to inform an individual’s diagnosis or prognosis, and provided more evidence-based information for decision-making by physicians, patients, and healthcare policymakers (53). Currently, the preventive postoperative treatment for iCCA patients primarily involves attempts with various therapeutic modalities including chemotherapy, targeted therapy, and immunotherapy. There is no clear guidance provided by existing guidelines. We tried to establish ATT-based predictive model for early interventions in high-risk recurrence patients.

It was found that ENO3 was involved in the Wnt/β-catenin signaling pathway. As a glycolytic enzyme, it can inhibit tumor growth and metastasis in hepatocellular carcinoma by suppressing the Wnt/β-catenin pathway (54, 55). HSPA13 participates as a novel component of the TNF-α receptor complex, promoting cell survival and inflammatory responses while inhibiting cell death through binding to RIP1 and TNFR1 (56–58). POSTN is involved in multiple pathways, including TGF-β, PI3K/Akt, Wnt, NF-κB, and MAPK. As a secreted extracellular matrix protein, it can bind to integrins and activate multiple signaling pathways, playing roles in diseases such as cancer and inflammation (59–62). PTBP3 participates in RNA metabolism and regulatory pathways, functioning as an RNA-binding protein involved in mRNA splicing, stability regulation, and translational control, thereby promoting tumor progression (63, 64). Both POSTN and ENO3 may be associated with the Wnt signaling pathway. ENO3 has been confirmed as an inhibitor of the Wnt/β-catenin signaling pathway, whereas POSTN has been reported to activate multiple signaling pathways including Wnt. This suggests that these two molecules may play opposing roles in the regulation of the Wnt pathway. Within the hepatocellular carcinoma microenvironment, low expression of ENO3 may fail to effectively inhibit the Wnt pathway, while high expression of POSTN continuously activates this pathway. Their combined effect leads to excessive activation of Wnt signaling, thereby accelerating hepatocellular carcinoma progression. This implied that they may functionally antagonize each other, although not through direct molecular interaction.

Furthermore, we found that samples in the high-risk group tended to be more sensitive to certain chemotherapy and targeted therapies but less responsive to immune checkpoint inhibitors, which offering a valuable reference for the treatment of postoperative patients. Additionally, we identified targeted therapies such as lapatinib, talazoparib, and ulixertinib which have not yet been applied in iCCA treatment. Our results provided a theoretical basis and potential direction for their off-label use in this setting. The construction of predictive model based on signature proteins had played a certain role in suggesting the recurrence of tumor, so that early intervention can be carried out. Of course, if dynamic monitoring of patients after tumor resection can be carried out in the future, it would be an expanded application of precision medicine.

Overall, we identified molecular subtypes associated with poor prognosis through proteomic analysis of iCCA ATTs, offering novel perspectives for oncology research. We constructed a multivariate Cox regression prediction model based on subtype-specific signature proteins, providing a basis for recurrence prediction. The predictive model provided drug recommendation for postoperative recurrent patients.

## 5 Conclusions

We identified molecular subtypes associated with poor prognosis through proteomic analysis of iCCA ATTs, offering novel perspectives for oncology research. We constructed a multivariate Cox regression prediction model based on subtype-specific signature proteins, providing a basis for recurrence prediction. The predictive model provided drug recommendation for postoperative recurrent patients.

## Data Availability

The datasets presented in this study can be found in online repositories. The names of the repository/repositories and accession number(s) can be found in the article/**Supplementary Material**.
