# Supplementary material for: A postoperative recurrence prediction model for intrahepatic cholangiocarcinoma based on multi-omics analysis of adjacent-to-tumor tissues
Source: Front Oncol. 2026 Mar 25;16:1753415. doi: 10.3389/fonc.2026.1753415 (PMC13057434; doi:10.3389/fonc.2026.1753415)
Supplement: Supplementary file 2 [file DataSheet2.docx]

**A postoperative recurrence prediction model for intrahepatic cholangiocarcinoma based on multi-omics analysis of adjacent-to-tumor tissues**

**Supplementary Figures**


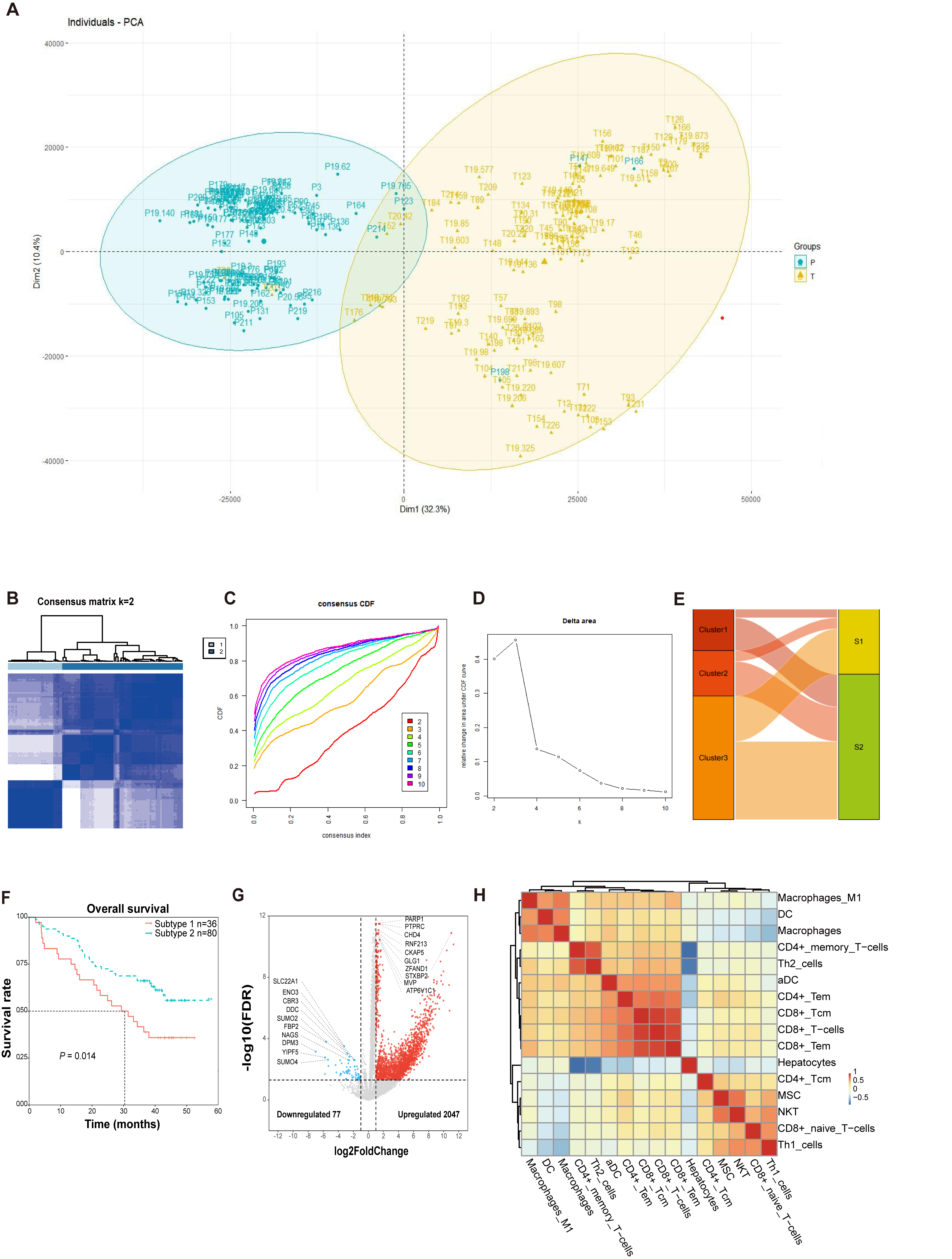


**Fig. S1. The supplemental results of the distinct features of the two ATT subtypes (related to Fig. 2 in the main text).**

(**A**) Principal component analysis (PCA) of tumor (T) and paired adjacent-to-tumor (P) samples. (**B**)Consensus matrix of consensus clustering when K=2. This matrix visualized the stability and agreement among clustering results when the number of clusters (K) was set to 2, indicating the consistency of sample assignments across multiple iterations of the clustering algorithm. (**C**) Consensus cumulative distribution function (CDF) of consensus clustering. (**D**) Delta area of consensus CDF. (**E**) Illustration of molecular subtype concordance between tumor (left panel) and adjacent-to-tumor samples (right panel). (**F**) Survival curves with overall survival (OS) as the observation endpoint. (**G**) This volcano plot displayed the differentially expressed proteins between ATTs subtypes, with the x-axis representing the log2 fold change in expression and the y-axis representing the -log10 transformed false discovery rate (FDR), highlighting proteins with significant expression differences. (**H**) Correlation analysis of immune cells.

**
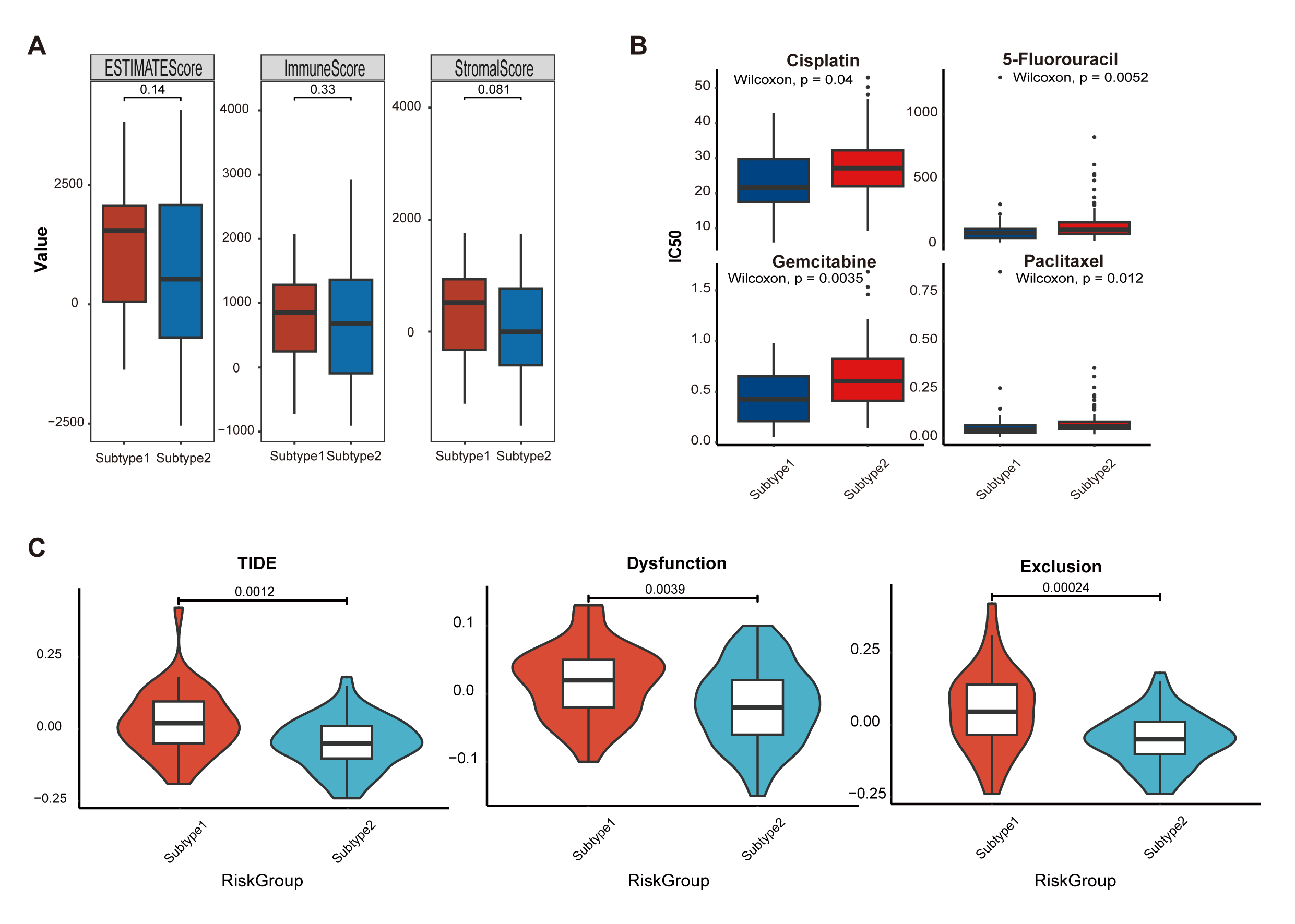
**

**Fig. S2. ESTIMATE scores between ATTs subtypes paired tumor samples and drug response across ATTs subtypes.**

**(A**) Comparison of immune scores across ATTs subtypes paired tumor samples. (**B**) Comparison of predicted IC50 concentrations for drugs across ATTs subtypes. (**C**) Prediction of immunotherapy response across ATTs subtypes.

**
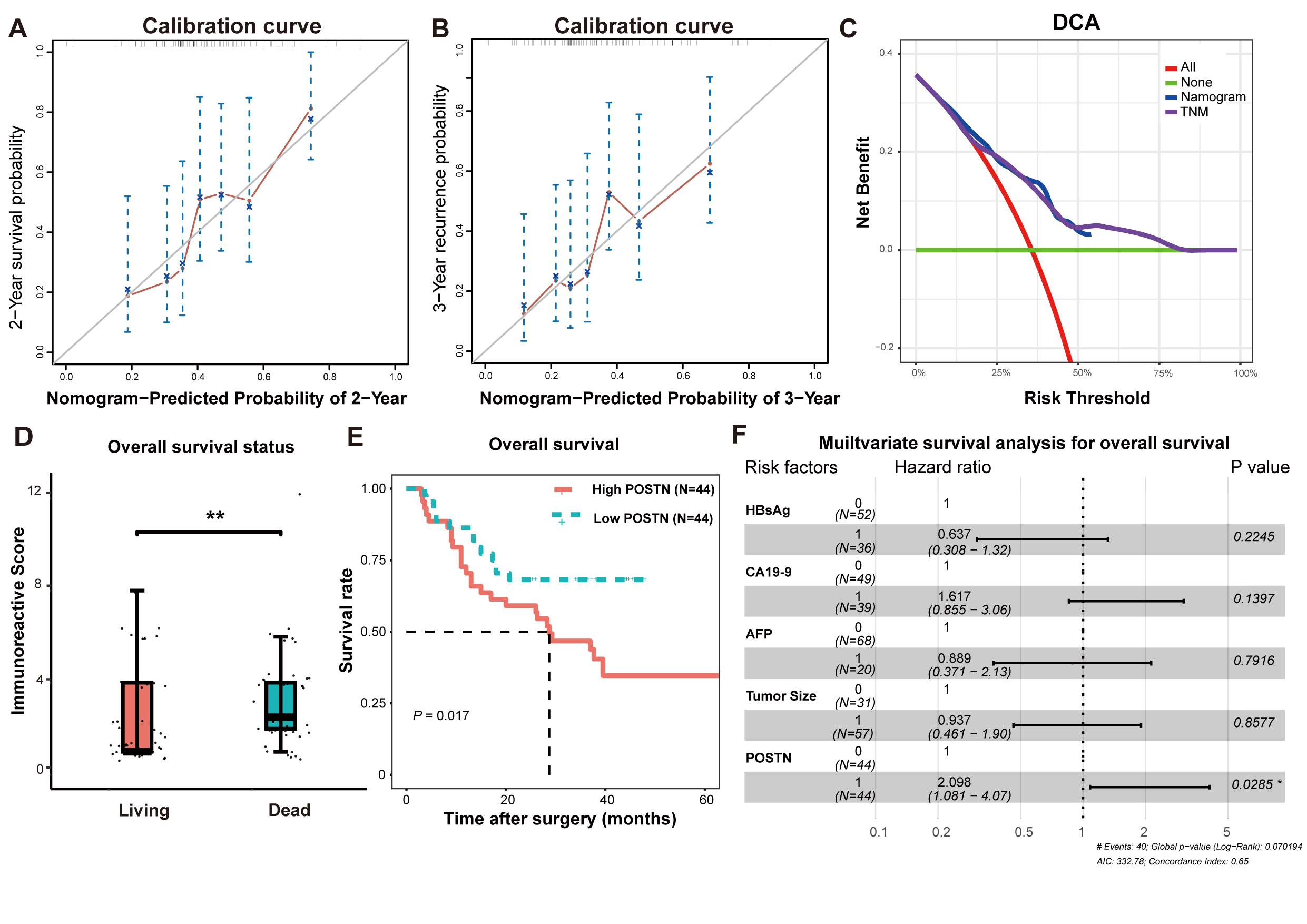
**

**Fig. S3. Evaluation of the recurrence prediction model and immunohistochemical validation with overall survival as the endpoint.**

(**A**) This figure depicted the calibration curve for predicting post-surgery recurrence after two years. (**B**) This figure depicted the calibration curve for predicting post-surgery recurrence after three years. (**C**) Decision curve analysis (DCA) curve for observing net benefit of interventions based on model prediction. (**D**) Comparison of immunohistochemical scores of POSTN with respect to overall survival outcomes. (**E**) Survival curve analysis was conducted for overall survival outcomes between the high-expression and low-expression groups of POSTN. (**F**) Multivariate survival analysis was performed for overall survival outcomes between the high- expression and low-expression groups of POSTN.

**Supplementary Table S1. The baseline clinicopathological features of the 116 iCCA patients.**

**Supplementary Table S2. The filtered proteome expression matrix of iCCA ATTs samples.**

**Supplementary Table S3. Clinical characteristics of iCCA in the tissue microarray (TMA) experiment.**

**Supplementary Table S4. Differentially expressed proteins (DEPs) across iCCA ATTs subtypes.**

**Supplementary Table S5. Results of GO enrichment analysis between ATTs subtypes DEPs.**

**Supplementary Table S6. ESTIMATE scores in iCCA ATTs samples.**

**Supplementary Table S7. xCell scores in iCCA ATTs samples.**

**Supplementary Table S8. Modeling input matrix based on signature proteins of S1.**

**Supplementary Table S9. Hazard Ratios (HR) from Univariate, Multivariable, and Backward Stepwise Cox Regression Models.**

**Supplementary Table S10. Targeted molecules expression profiles in iCCA ATTs samples.**

**Supplementary Table S11. TIDE scores in iCCA ATTs samples.**
