# Supplementary material for: A postoperative recurrence prediction model for intrahepatic cholangiocarcinoma based on multi-omics analysis of adjacent-to-tumor tissues
Source: Front Oncol. 2026 Mar 25;16:1753415. doi: 10.3389/fonc.2026.1753415 (PMC13057434; doi:10.3389/fonc.2026.1753415)
Supplement: Supplementary file 1 [file DataSheet1.pdf]

Supplementary Table S1. The baseline clinicopathological features of the 116 iCCA patients

ATTs subtype	ID	Sex	Age	Date of surgery	Last follow-up	Date of death	Date of recurrence	Recurrence	TTR	Osstatus	OS	RFSsta	RFS	T	N	M	TNM8th
2	T101	female	50	2018/11/23		2019/4/28		0	5.2	1	5.2	1	5.2	T2	N1	M0	III B
2	T102	male	68	2018/11/29	2022/12/19			0	49.36666	0	49.36666	0	49.36666	T2	N0	M0	II
2	T103	male	67	2018/12/3	2022/12/19		2020/5/22	1	17.86666667	0	49.2333	1	17.86666667	T2	N0	M0	II
2	T104	male	52	2018/12/3		2019/9/15		1	3.4	1	9.533333333	1	3.4	T2	N0	M0	II
1	T105	male	49	2018/12/5		2019/4/15		1	2.3	1	4.366666667	1	2.3	T2	N0	M0	II
2	T106	male	54	2018/12/5		2020/3/15	2019/9/15	1	9.466666667	1	15.53333333	1	9.466666667	T2	N1	M0	III B
1	T108	male	44	2018/12/13	2022/9/20		2019/8/19	1	8.3	0	45.9	1	8.3	T2	N0	M0	II
2	T110	female	60	2018/12/18	2022/12/13		2020/4/15	1	16.13333333	0	48.5333	1	16.13333333	T2	N0	M0	II
2	T117	female	68	2018/12/24	2022/12/13		2021/12/1	1	35.7666	0	48.3333	1	35.7666	T2	N0	M0	II
1	T118	female	66	2018/12/26	2022/12/13			0	48.2666	0	48.2666	0	48.2666	T2	N0	M0	II
2	T119	male	63	2019/1/4		2020/4/15		1	9.866667	1	15.56666667	1	9.866667	T1a	N1	M0	III B
2	T12	female	65	2017/10/27		2021/8/15	2018/5/2	1	6.233333333	1	42.26666	1	6.233333333	T2	N0	M0	II
1	T121	male	71	2019/1/30		2022/3/15	2020/2/15	1	12.7	1	38	1	12.7	T2	N0	M0	II
1	T123	male	65	2019/2/14		2019/6/15		1	1.4	1	4.033333333	1	1.4	T2	N0	M0	II
1	T126	female	56	2019/2/18		2021/7/15	2020/11/15	1	21.2	1	29.2666	1	21.2	T2	N0	M0	II
2	T129	female	54	2019/2/22	2022/12/13		2020/3/15	1	15.63333333	0	46.3333	1	15.63333333	T1b	N1	M0	III B
2	T130	female	70	2019/2/20		2021/12/15	2019/11/15	1	8.933333333	1	34.3	1	8.933333333	T1b	N0	M0	I B
1	T131	male	51	2019/2/27		2021/3/15	2020/11/15	1	20.9	1	24.9	1	20.9	T1b	N0	M0	I B
1	T134	male	30	2019/3/6		2019/7/1	2019/3/15	1	0.3	1	3.9	1	0.3	T2	N0	M0	II
1	T136	female	62	2019/3/8		2019/5/10		0	2.1	1	2.1	1	2.1	T1a	N0	M0	I A
2	T140	male	72	2019/3/15	2022/12/13			0	45.6333	0	45.6333	0	45.6333	T1a	N0	M0	I A
1	T147	female	59	2019/3/28	2022/12/13		2020/3/15	1	11.76666667	0	45.2	1	11.76666667	T2	N0	M0	II
2	T148	male	48	2019/3/28	2022/12/13			0	45.2	0	45.2	0	45.2	T1a	N0	M0	I A
2	T150	male	56	2019/4/8	2022/12/13		2019/11/15	1	7.366666667	0	44.8333	1	7.366666667	T2	N0	M0	II
2	T152	male	67	2019/5/10	2022/12/13		2022/7/15	1	38.733	0	43.7666	1	38.733	T2	N0	M0	II
1	T153	male	55	2019/4/16	2022/12/13			0	44.5666	0	44.5666	0	44.5666	T2	N0	M0	II
1	T154	male	62	2019/4/17		2020/8/9	2019/8/2	1	3.5	1	16	1	3.5	T2	N0	M0	II
1	T156	male	76	2019/4/22		2019/8/15		1	1.4	1	3.833333333	1	1.4	T2	N1	M0	III B
1	T158	male	69	2019/4/24		2020/12/30	2020/3/15	1	8.466667	1	20.53333	1	8.466667	T1a	N1	M0	III B
1	T159	female	62	2019/4/26		2021/1/30	2020/3/15	1	10.8	1	21.5	1	10.8	T2	N0	M0	II
2	T162	male	72	2019/5/8	2022/12/16		2021/7/15	1	25.83333333	0	43.9333	1	25.83333333	T1a	N0	M0	I A
1	T164	female	78	2019/5/8		2021/3/15	2019/12/15	1	7.366666667	1	22.5666	1	7.366666667	T2	N0	M0	II
1	T166	male	63	2019/5/10		2020/1/31		1	1.533333	1	8.866666667	1	1.533333	T2	N0	M0	II
1	T167	female	72	2019/5/10		2020/2/14		1	6.6	1	9.333333333	1	6.6	T1a	N1	M0	III B
1	T173	female	59	2019/6/3	2022/12/13			1	2.2	0	42.9666	1	2.2	T2	N0	M0	II
2	T176	male	65	2019/6/12	2022/12/13			0	42.666	0	42.666	0	42.666	T1a	N0	M0	I A
2	T177	female	71	2019/6/13	2022/12/15			0	42.7	0	42.7	0	42.7	T2	N0	M0	II
1	T179	male	69	2019/6/19		2022/6/15	2019/9/15	1	2.9333	1	36.4	1	2.9333	T2	N1	M0	III B
1	T181	male	59	2019/6/24		2020/9/3		1	6.466667	1	14.56666667	1	6.466667	T2	N1	M0	III B
2	T182	male	69	2019/6/25		2021/3/15	2020/7/15	1	13.9	1	20.9666	1	13.9	T2	N1	M0	III B
2	T184	male	62	2019/7/1	2022/12/13			0	42.0333	0	42.0333	0	42.0333	T2	N0	M0	II
1	T187	male	52	2019/7/12	2022/12/13		2021/11/15	1	28.5666	0	41.666	1	8.666667	T2	N0	M0	II
1	T19.136	female	71	2019/4/26	2022/12/13		2021/6/15	1	25.9	0	44.2333	1	25.9	T1a	N0	M0	I A
1	T19.140	male	78	2019/4/28		2022/1/15	2020/5/15	1	26.8	1	33.1	1	26.8	T2	N0	M0	II
2	T19.144	female	55	2019/5/6	2022/12/13			0	43.9	0	43.9	0	43.9	T1a	N0	M0	I A
2	T19.17	male	57	2019/1/16	2022/12/13			1	32.3	0	47.5666	1	32.3	T1a	N0	M0	I A
1	T19.177	female	76	2019/5/28	2022/12/13			0	43.1666	0	43.1666	0	43.1666	T2	N0	M0	II
2	T19.206	female	70	2019/6/10		2021/1/15	2019/10/15	1	4.233333333	1	19.13333333	1	4.233333333	T1b	N0	M0	I B
2	T19.217	female	72	2019/6/12	2022/12/15		2022/4/15	1	34.6	0	42.7333	0	34.6	T2	N0	M0	II
2	T19.220	female	75	2019/6/12		2022/8/20	2021/11/15	1	29.5666	1	38.8333	1	29.5666	T1a	N0	M0	I A
2	T19.3	male	61	2019/1/3		2020/6/15	2019/7/3	1	6.033333333	1	17.63333333	1	6.033333333	T2	N0	M0	II
2	T19.325	female	60	2019/7/4	2022/12/13			0	41.9333	0	41.9333	0	41.9333	T1a	N0	M0	I A
2	T19.413	female	51	2019/7/24	2022/12/13		2020/2/15	1	6.866666667	0	41.2666	1	6.866666667	T1b	N0	M0	I B
2	T19.511	male	75	2019/8/15	2022/12/13			0	40.5333	0	40.5333	0	40.5333	T2	N0	M0	II
2	T19.557	female	69	2019/8/28	2022/12/13			0	40.1	0	40.1	0	40.1	T1a	N0	M0	I A
2	T19.603	male	66	2019/9/10	2022/12/19			0	39.86666	0	39.86666	0	39.86666	T1a	N0	M0	I A
2	T19.607	male	64	2019/9/11	2022/12/13			0	39.633	0	39.633	0	39.633	T1a	N0	M0	I A
2	T19.608	female	65	2019/9/12	2022/12/13			0	39.6	0	39.6	0	39.6	T2	N0	M0	II
2	T19.62	female	62	2019/3/4	2022/12/13		2022/8/15	1	42	0	46	1	42	T2	N0	M0	II
1	T19.642	male	75	2019/9/23		2020/2/15	2019/11/15	1	1.766666667	1	4.833333333	1	1.766666667	T2	N0	M0	II
2	T19.649	female	61	2019/9/25	2022/12/20		2020/10/15	1	13.53333333	0	39.4	1	13.53333333	T2	N0	M0	II
2	T19.689	male	69	2019/10/11	2022/12/13			0	38.6333	0	38.6333	0	38.6333	T1b	N1	M0	III B
2	T19.699	female	85	2019/10/14	2022/12/19			0	38.7333	0	38.7333	0	38.7333	T1b	N0	M0	I B
1	T19.705	male	65	2019/10/15		2022/5/15		0	31.4333	1	31.4333	0	31.4333	T1b	N0	M0	I B
2	T19.752	male	61	2019/10/25		2022/1/15	2021/2/15	1	14	1	27.1	1	14	T1a	N1	M0	III B
2	T19.793	female	70	2019/11/11	2022/12/13		2021/3/15	1	16.333	0	37.6	1	16.333	T1b	N0	M0	I B
1	T19.85	male	51	2019/3/20	2022/12/13		2020/10/12	1	19.0666	0	45.4666	1	19.0666	T1a	N0	M0	I A
2	T19.873	female	45	2019/12/4	2022/12/13		2020/11/15	1	13.93333333	0	36.8333	1	13.93333333	T2	N0	M0	II
2	T19.893	male	49	2019/12/11		2020/3/15		0	3.166666667	1	3.166666667	1	3.166666667	T2	N1	M0	III B
2	T19.98	male	57	2019/4/1	2022/12/13			0	45.0666	0	45.0666	0	45.0666	T1a	N0	M0	I A
2	T190	male	73	2019/7/19		2020/11/10	2019/12/31	1	5.5	1	16.03333333	1	5.5	T2	N0	M0	II
2	T191	male	65	2019/7/22	2022/12/13		2020/11/14	1	16.03333	0	41.3333	1	16.03333	T1a	N0	M0	I A
2	T192	female	76	2019/7/24	2022/12/13			0	41.2666	0	41.2666	0	41.2666	T1a	N0	M0	I A
2	T193	male	57	2019/7/25	2022/12/14			0	41.2666	0	41.2666	0	41.2666	T1b	N0	M0	I B
2	T195	female	53	2019/7/26		2021/8/28	2020/10/1	1	14.4333	1	25.4666	1	14.4333	T2	N0	M0	II
2	T196	female	62	2019/7/26	2022/12/13			0	41.2	0	41.2	0	41.2	T1a	N0	M0	I A
2	T197	male	68	2019/8/1		2022/11/15	2021/10/15	1	26.8666	1	40.0666	1	26.8666	T1b	N0	M0	I B
1	T198	male	77	2019/8/1	2022/12/13		2021/4/15	1	16.33333333	0	41	1	16.33333333	T1a	N0	M0	I A
2	T2	male	52	2017/9/14		2019/5/15		0	20.26666667	1	20.26666667	1	20.26666667	T1b	N0	M0	I B
2	T20.31	female	70	2020/1/14	2022/12/13			0	35.4666	0	35.4666	0	35.4666	T1a	N0	M0	I A
2	T20.42	male	45	2020/1/16	2022/12/13			0	35.4	0	35.4	0	35.4	T1a	N0	M0	I A
2	T20.56	male	56	2020/3/11	2022/10/26		2020/6/16	1	3.1666666	0	31.966	1	3.1666666	T1a	N0	M0	I A
2	T200	male	64	2019/8/5		2022/9/27	2020/7/1	1	11.0333	1	38.3	1	11.0333	T1b	N1	M0	III B
2	T203	male	65	2019/8/7	2022/12/13			0	40.8	0	40.8	0	40.8	T1a	N0	M0	I A
2	T209	male	69	2019/8/28		2021/7/15	2019/9/15	1	0.6	1	22.9	1	0.6	T1a	N1	M0	III B
2	T211	male	37	2019/9/27		2021/3/15	2020/12/15	1	14.83333333	1	15.23333333	1	14.83333333	T1b	N0	M0	I B
1	T214	female	65	2019/11/20		2022/9/15	2021/1/5	1	13.73333333	1	34.333	1	13.73333333	T1b	N0	M0	I B
2	T216	male	61	2019/11/27	2022/12/19			0	37.2666	0	37.2666	0	37.2666	T1b	N0	M0	I B
1	T219	male	72	2019/12/2	2022/12/13			0	36.9	0	36.9	0	36.9	T1b	N0	M0	I B
2	T220	male	70	2019/12/3		2021/5/15	2020/10/6	1	10.26666667	1	17.6333	1	10.26666667	T1b	N0	M0	I B
2	T222	female	70	2019/12/9	2022/12/13		2020/4/15	1	4.266666667	0	36.6666	1	4.266666667	T1b	N0	M0	I B
2	T224	female	63	2019/12/16	2022/12/13			0	36.4333	0	36.4333	0	36.4333	T1b	N1	M0	III B
2	T225	female	47	2019/12/20	2022/12/13			0	36.3	0	36.3	0	36.3	T1a	N1	M0	III B
2	T226	male	54	2019/12/20	2021/12/14		2020/9/28	1	10.13333333	0	36.3333	1	10.13333333	T1a	N0	M0	I A
2	T231	female	67	2020/1/8	2022/10/20			0	33.8666	1	33.8666	0	33.8666	T1b	N0	M0	I B
2	T232	male	47	2020/1/9		2021/6/1	2020/1/15	1	1.03333333	1	16.9666	1	1.03333333	T2	N1	M0	III B
2	T234	female	42	2020/1/15		2020/9/26	2020/4/27	1	3.4	1	8.5	1	3.4	T1a	N1	M0	III B
2	T235	female	66	2020/1/17		2021/8/23	2020/8/15	1	7.2	1	19.46666	1	7.2	T2	N0	M0	II
2	T3	female	71	2017/9/14		2017/12/15		1(因)	3.066666667	1	3.066666667	1	3.066666667	T2	N1	M0	III B
2	T4	male	70	2017/9/15		2017/11/15		0	2.033333333	1	2.033333333	1	2.033333333	T2	N1	M0	III B
2	T45	male	46	2018/3/6		2019/7/15		1	9	1	16.53333333	1	9	T2	N0	M0	II
2	T46	male	58	2018/3/7		2018/7/15		0	4.333333333	1	4.333333333	1	4.333333333	T2	N0	M0	II
2	T51	male	75	2018/3/21	2022/12/19		2019/9/15	1	18.1	0	57.8	1	18.1	T1b	N0	M0	I B
2	T52	female	78	2018/3/21		2019/2/9		0	10.83333333	1	10.83333333	1	10.83333333	T1a	N0	M0	I A
2	T55	female	77	2018/3/26		2019/5/1		0	13.36666666	1	13.36666666	1	13.36666666	T2	N0	M0	II
2	T57	female	39	2018/4/8	2022/12/13		2019/8/15	1	16.46666667	0	57	1	16.46666667	T1a	N0	M0	I A
1	T68	female	79	2018/8/21	2022/12/13			0	52.5	0	52.5	0	52.5	T1a	N0	M0	I A
2	T71	male	63	2018/8/23	2022/12/13			0	52.43333	0	52.43333	0	52.43333	T1b	N0	M0	I B
1	T86	male	64	2018/10/12		2019/11/15	2019/5/7	1	6.9	1	13.3	1	6.9	T1a	N0	M0	I A
2	T89	female	68	2018/10/19	2022/12/19		2020/7/1	1	20.7	0	50.73333	1	20.7	T1a	N0	M0	I A
1	T90	female	67	2018/10/22		2020/12/15	2020/10/15	1	24.13333333	1	26.16666667	1	24.13333333	T1a	N0	M0	I A
1	T93	female	70	2018/10/26		2020/1/24	2019/6/15	1	7.733333333	1	15.16666667	1	7.733333333	T1a	N0	M0	I A
2	T95	male	70	2018/10/29		2022/5/15	2019/8/15	1	9.666666667	1	43.133	1	9.666666667	T2	N0	M0	II
2	T97	male	60	2018/11/5		2021/2/9	2020/7/1	1	20.133	1	27.5	1	20.133	T2	N0	M0	II
1	T98	male	62	2018/11/5	2022/12/13			0	49.9666	0	49.9666	0	49.9666	T1a	N0	M0	I A
2	T99	female	65	2018/11/16	2022/12/13			0	49.6	0	49.6	0	49.6	T1a	N0	M0	I A

Abbreviations: TTR: time to recurrence; OS: overall survival; RFS: recurrence-free survival; TNM: tumor node metastasis;

Supplementary Table S4. Differentially expressed proteins (DEPs) across iCCA ATTs subtypes

Uniprot ID	log2FC	AveExpr	F	P.Value	adj.P.Val
A0A0C4DH67	1.068082	21.56629668	13.06842143	0.00050315	0.001328684
A0A0C4DH69	1.124644714	21.6153033	10.88956403	0.001608867	0.00372004
A5D8V6	1.530121446	18.43887055	38.37315809	4.51994E-08	4.33451E-07
A5YKK6	1.057417772	18.33500301	43.00548339	1.55661E-09	2.73161E-08
A6NCE7	1.035142765	19.9628449	13.49699132	0.000559244	0.001454375
A6NHR9	2.021708439	15.59433193	56.42152663	1.87972E-11	8.81729E-10
A6NI72	1.738645255	17.99888677	27.08301131	1.54394E-06	8.48389E-06
A6NNZ2	1.094887981	23.63300589	32.48889666	1.70178E-07	1.3223E-06
A8MVU1	1.370990467	18.05658561	18.96854214	3.55379E-05	0.000127723
B0I1T2	2.024055247	16.01449294	29.08792201	7.39709E-07	4.53992E-06
O00139	1.075797674	16.0340973	8.078484043	0.00639471	0.012464477
O00154	1.215481184	19.9515728	33.21794796	7.28279E-08	6.47551E-07
O00160	1.517224514	18.93346154	48.09878337	2.39867E-10	6.63903E-09
O00178	1.64868216	18.36389947	47.34510514	3.32945E-10	8.30144E-09
O00182	1.277711495	20.88110067	36.36125629	1.97127E-08	2.18042E-07
O00267	1.204045887	18.91687527	61.4583339	2.25095E-12	1.87079E-10
O00391	1.368274138	17.80034844	11.25130162	0.00115807	0.00275652
O00425	1.112030078	16.62759622	5.637724945	0.021045229	0.035534607
O00462	1.601490796	17.08236215	43.73630347	2.4513E-09	3.98603E-08
O00469	1.805677871	17.55332671	36.11806981	2.85302E-08	2.96912E-07
O00471	1.166537185	18.58187616	29.28988643	3.50256E-07	2.40635E-06
O00534	1.408207687	17.80679495	18.80773253	3.35943E-05	0.000121614
O00560	1.227480951	21.37622125	34.16496088	4.76228E-08	4.53782E-07
O00629	1.038939238	21.03196684	23.4339317	3.96307E-06	1.90942E-05
O00757	-1.157428397	25.01709728	13.83020356	0.000310047	0.000863745
O14497	1.169022548	16.2093066	25.19836545	2.10469E-06	1.10964E-05
O14498	1.60113738	18.55388176	15.52028129	0.000265063	0.000754945
O14545	1.157702705	16.88389242	25.46741057	3.06703E-06	1.52688E-05
O14617	1.014988627	20.25953826	53.27349811	3.69497E-11	1.51443E-09
O14656	1.010077089	20.14649459	22.0814032	7.31532E-06	3.23779E-05
O14672	1.030676216	20.03148271	32.58900346	8.66829E-08	7.45274E-07
O14732	1.365835591	18.94225874	9.647909523	0.002635311	0.005703327
O14776	1.253983028	19.38498079	37.50734121	1.23822E-08	1.49085E-07
O14786	1.650124018	19.12912255	59.19838808	4.80847E-12	3.12759E-10
O14787	1.256534041	18.16642325	28.10578431	5.50568E-07	3.53498E-06
O14907	1.294553047	21.24611093	33.95999825	6.92594E-08	6.21362E-07
O14908	1.20596557	18.74721969	28.30017598	5.53994E-07	3.55316E-06
O14974	1.278729386	19.69153046	58.86872668	5.376E-12	3.39818E-10
O14981	1.293184435	16.02828076	32.770822	9.78894E-08	8.22711E-07
O15042	1.275066842	19.38338418	46.25585095	4.56483E-10	1.05061E-08
O15085	1.09893975	14.94883099	17.36555171	8.59662E-05	0.000277766
O15117	1.686520097	17.4355107	37.87022651	3.72586E-08	3.72212E-07
O15121	1.15588418	19.49263164	11.02969274	0.00199703	0.004477417
O15212	1.016067435	21.72887164	33.70636751	5.6241E-08	5.21777E-07
O15231	1.004636843	16.67723899	11.27401271	0.001310399	0.003079901
O15269	1.123581346	18.9063174	22.41959247	6.4186E-06	2.89006E-05
O15294	1.300168488	18.20954564	42.19768712	2.25781E-09	3.76344E-08
O15355	1.447144826	19.21810991	33.32295714	6.87272E-08	6.18442E-07
O15357	1.252899041	15.9497706	18.83092972	4.24897E-05	0.000149476
O15400	1.038843288	22.40826314	45.98147474	5.04796E-10	1.13989E-08
O15427	1.208873861	17.94160238	13.96902179	0.000367204	0.001005653
O15455	1.057449512	17.2124692	8.827548951	0.003895081	0.008015187
O15460	1.854647463	16.88628611	20.43599012	2.91011E-05	0.000107627
O15498	1.334961763	20.25769937	39.25340195	6.30724E-09	8.63674E-08
O43148	1.316476833	17.76896688	33.65047914	6.57158E-08	5.94208E-07
O43149	1.271863282	17.05205666	26.7049554	1.00038E-06	5.88041E-06
O43164	1.098477804	16.40460897	15.39850925	0.000190039	0.000562688
O43172	1.065631662	19.26618632	38.81446905	7.61536E-09	1.01043E-07
O43252	1.61645215	18.10542728	47.82367844	2.72406E-10	7.34269E-09
O43294	1.234334574	20.49295669	34.8157059	3.62018E-08	3.63476E-07
O43314	1.150106529	16.80684387	24.0436222	3.42109E-06	1.68492E-05
O43464	1.084503448	21.35984474	11.81201118	0.000816072	0.002030259

O43493	1.096996171	18.91254038	9.09197283	0.003294925	0.006952338
O43752	1.202554749	17.73207778	10.76319129	0.001804537	0.004107398
O43815	1.282708477	18.3522646	27.05525856	8.93524E-07	5.33617E-06
O60218	1.532244832	21.96029834	9.14594405	0.003081522	0.006564553
O60264	1.516436637	17.23628951	43.78458	1.38969E-09	2.51236E-08
O60341	1.132662481	17.74530069	32.62631436	9.36449E-08	7.94853E-07
O60488	1.498380373	18.99322553	14.20617979	0.000270041	0.000766205
O60496	1.308918668	18.28638818	29.51938993	4.20051E-07	2.81792E-06
O60568	1.171870233	20.15816783	44.92481253	7.44927E-10	1.53712E-08
O60603	1.294715277	15.61973757	9.152436867	0.003892114	0.008011837
O60645	1.043348977	19.06130906	21.67561278	8.6693E-06	3.76759E-05
O60711	1.232675001	18.40506355	13.12175499	0.000558996	0.001454362
O60831	1.211208017	20.45525084	17.88945198	6.48143E-05	0.000216675
O60934	1.085453645	16.6130127	9.944611202	0.002507018	0.005459241
O60936	1.224624801	21.78860724	40.33228369	4.26263E-09	6.26722E-08
O75044	1.230139565	15.91406098	11.85762497	0.001011629	0.002445017
O75113	1.875595336	15.20239723	27.47133133	1.15017E-06	6.61789E-06
O75165	1.33412341	19.36105566	67.73974554	2.8735E-13	5.11246E-11
O75323	1.070847916	19.50501791	19.18979845	2.88061E-05	0.000106668
O75391	1.054062041	19.32605182	20.367625	1.61252E-05	6.45438E-05
O75410	1.57271841	17.01573133	29.84399437	3.44751E-07	2.37672E-06
O75475	1.090014187	19.79146763	27.10118351	8.28762E-07	4.98423E-06
O75487	1.80788746	17.41635541	32.36114821	1.92872E-07	1.46279E-06
O75503	1.009627897	18.13949895	23.76907694	4.93921E-06	2.29296E-05
O75558	1.268560185	18.02138134	10.15104011	0.002076657	0.004635104
O75563	1.020920224	17.79861838	14.77129155	0.000248535	0.000711768
O75569	1.227030238	20.71103644	40.21968686	4.54418E-09	6.55238E-08
O75594	1.02150032	19.83878282	11.66217637	0.000894876	0.002195546
O75694	1.213944809	19.06994085	50.94722316	8.39824E-11	2.83927E-09
O75794	1.077028215	16.96122483	21.09468833	2.03014E-05	7.85168E-05
O75828	-1.310503882	22.9232081	11.65774359	0.000879855	0.002169366
O75843	1.018954436	17.24164349	6.636504975	0.012827372	0.022906295
O75882	1.014193929	17.93408979	28.0787522	5.70107E-07	3.62928E-06
O75886	1.153329722	19.57282615	45.90924698	6.08141E-10	1.28924E-08
O75934	1.002876561	20.8626603	31.40999237	1.39673E-07	1.11277E-06
O75976	1.200327213	17.38275495	23.60477667	3.81265E-06	1.84736E-05
O75995	1.604226942	18.85182331	16.81422734	0.000155645	0.000472782
O76021	1.019403458	20.68723985	48.19063053	2.25698E-10	6.3111E-09
O94776	1.137298931	19.07560249	38.32985336	9.0022E-09	1.15352E-07
O94804	1.073363357	18.24196461	25.25522189	1.90357E-06	1.01978E-05
O94888	1.243354079	17.97385756	19.54515529	2.52593E-05	9.4885E-05
O94901	1.109082053	17.69460672	17.50888603	6.00034E-05	0.00020297
O94915	1.081724097	14.43952493	11.46582223	0.001102575	0.00264018
O94919	1.421496383	19.02908877	35.34958369	3.03535E-08	3.1102E-07
O94925	1.216848651	18.48691241	18.67913491	3.55675E-05	0.000127753
O95049	1.026191698	17.60903445	8.475095323	0.004424096	0.008968087
O95155	1.412895808	15.92999415	32.80722072	1.40299E-07	1.11494E-06
O95251	1.005350266	16.38987604	9.549167696	0.003165434	0.006714625
O95302	1.531490915	18.42241938	45.36698825	1.34884E-09	2.46834E-08
O95319	1.485591885	18.03730994	22.74425314	1.3703E-05	5.58195E-05
O95352	1.093790002	19.89971383	48.00602476	2.4804E-10	6.77557E-09
O95379	1.478644505	19.19489235	26.3655338	1.91459E-06	1.02203E-05
O95425	1.236359637	16.72744546	29.74686779	2.86646E-07	2.02514E-06
O95466	1.579150849	16.93124591	18.88950279	4.42292E-05	0.000155126
O95544	1.078023794	17.96017131	20.4188026	2.04998E-05	7.90914E-05
O95630	1.19989648	18.8364775	32.23778169	1.16683E-07	9.55171E-07
O95684	1.255861108	17.96078905	26.8754609	1.09088E-06	6.32539E-06
O95749	1.001399418	18.08044749	23.95028788	4.15325E-06	1.98665E-05
O95758	1.28020133	18.85629618	19.03488105	3.29563E-05	0.000119809
O95817	1.272606128	18.99290981	36.98791036	1.54365E-08	1.78669E-07
O95819	1.181010905	16.55406082	20.56988474	1.699E-05	6.71963E-05
O95858	1.504445394	18.73845358	12.45104248	0.001116908	0.002670227
O95994	1.419589903	21.42775616	5.373152609	0.023721686	0.039354191
O96019	1.037687689	20.17103319	22.57479738	5.90036E-06	2.68909E-05
P00488	1.861227607	19.84478216	44.87111142	8.40848E-10	1.68282E-08

P00746	1.514079021	18.7832513	32.5874189	2.10735E-07	1.57041E-06
P01033	1.947875828	19.8485216	16.63826354	0.000140317	0.000431256
P01137	1.481092154	18.22262098	6.454010265	0.015996341	0.027753582
P01703	1.204081724	22.20683862	18.06464858	5.57083E-05	0.000190599
P01833	1.435710697	18.32411602	6.829872734	0.011066928	0.020147398
P01889	1.037898156	22.82778463	31.02938942	1.63075E-07	1.27466E-06
P01903	1.188901993	24.57142439	48.07846576	2.35048E-10	6.542E-09
P01920	1.19563599	20.17708909	19.98577602	1.93706E-05	7.53666E-05
P02741	1.349835618	21.814196	8.785028793	0.003841042	0.007920329
P02745	1.033095818	19.92685944	21.05692666	1.21094E-05	5.00432E-05
P02750	1.083539578	21.40648836	25.50009496	1.63154E-06	8.90798E-06
P02788	1.064911994	23.74895314	21.81385695	8.03632E-06	3.52045E-05
P04062	1.48827446	18.48009873	32.42112242	1.44708E-07	1.14693E-06
P04083	1.121573816	25.11103658	66.87097729	3.8033E-13	5.55096E-11
P04216	1.965728821	22.57458858	52.13840964	5.50752E-11	2.05873E-09
P04233	1.071154155	21.87509114	26.81016587	9.46794E-07	5.62623E-06
P04275	1.473644417	17.84510415	25.57245314	1.59774E-06	8.74512E-06
P05107	1.533485606	20.80360613	55.01312432	2.01504E-11	9.3473E-10
P05164	1.116643398	23.26863998	28.12145467	5.40692E-07	3.48653E-06
P05543	1.186911098	19.56780381	26.11039655	1.28517E-06	7.27572E-06
P05997	1.559429087	17.48417719	9.081756235	0.003537023	0.007392786
P06280	1.469251226	18.23115022	24.70985362	3.17394E-06	1.56966E-05
P06681	1.031152038	20.47552665	37.46974746	1.25645E-08	1.50674E-07
P06756	1.357456725	20.13464693	62.54842968	1.56643E-12	1.45448E-10
P07093	1.293813317	17.1793571	17.53377295	7.37566E-05	0.000242818
P07942	2.105936341	18.31973214	34.44291585	4.41142E-08	4.25773E-07
P07948	1.447005051	19.72054042	47.82422004	2.80134E-10	7.51713E-09
P08138	1.242928243	20.11728128	23.9916046	3.19932E-06	1.5796E-05
P08185	1.03480288	20.78752314	23.22509866	4.3753E-06	2.07957E-05
P08237	1.006807508	19.55043313	11.35951217	0.001015598	0.002452517
P08243	3.688449409	16.72030471	17.93178426	0.000511628	0.001346935
P08567	1.270901643	21.20113246	29.38085735	3.24686E-07	2.26448E-06
P08575	1.478453066	20.74343726	85.13998512	1.36998E-15	2.73266E-12
P08631	1.572614367	18.1543455	29.90752877	3.25172E-07	2.26523E-06
P09012	1.238407694	21.25251742	29.00528466	3.83842E-07	2.59538E-06
P09211	1.082726468	26.32270822	59.57476579	4.23451E-12	2.85554E-10
P09486	1.467509209	19.41531465	19.88066458	2.35321E-05	8.92935E-05
P09758	1.265463684	19.24286181	8.850735727	0.004411805	0.008949234
P09874	1.005408458	22.23619622	88.50984164	5.1384E-16	2.73266E-12
P09913	1.270856178	19.83459111	13.43167684	0.000374831	0.001023728
P09917	2.143877673	17.54169726	36.49987574	2.17432E-08	2.36997E-07
P0DJI8	1.280235748	21.97870414	6.701945	0.011332061	0.020567501
P0DOX2	1.0351178	23.09139864	14.41165461	0.000233694	0.000673941
P11215	1.559520435	19.45301573	36.48348805	1.84615E-08	2.05724E-07
P11413	1.228825755	21.74229654	47.18047828	3.25656E-10	8.15367E-09
P12318	1.371359708	19.26887718	11.78947346	0.001522224	0.003541597
P12429	1.431382099	21.61814813	29.77216987	2.72903E-07	1.94642E-06
P12931	1.151487223	19.26312655	14.14080646	0.000269583	0.000765632
P13611	2.264723273	18.33643981	25.0471742	2.81052E-06	1.42527E-05
P13674	1.332112796	21.86974183	25.09474224	1.9395E-06	1.03348E-05
P13747	1.068562325	19.11929911	12.06222474	0.000768137	0.001923682
P13796	1.02737079	25.64651576	67.7434507	2.87007E-13	5.11246E-11
P13929	-1.135431754	25.78503752	17.83212628	4.80161E-05	0.000166374
P14598	1.238884947	18.01486873	17.79526719	5.11124E-05	0.000175982
P14618	1.033394913	25.43296756	45.28031028	6.5332E-10	1.36695E-08
P14780	1.068194594	18.94521338	10.64252182	0.001455852	0.003400398
P15153	1.077523519	22.43834356	6.308527928	0.013444618	0.023869633
P15291	1.103115966	18.63791925	22.10222823	1.00558E-05	4.26162E-05
P15498	1.576878514	16.73031625	37.0715872	2.61693E-08	2.79139E-07
P16284	1.005656299	19.10616805	28.22796857	5.17217E-07	3.36416E-06
P16333	1.048469109	19.11722822	25.45537854	1.76762E-06	9.57888E-06
P16422	1.514484437	18.52162564	6.850695009	0.011589787	0.020971662
P16885	1.726418492	17.48682531	42.65487254	2.15733E-09	3.62625E-08
P16949	1.378113319	21.13299213	28.50593687	5.61792E-07	3.58779E-06
P16989	1.333064758	19.22480328	20.36135393	2.30653E-05	8.76335E-05

P17066	-1.634854328	23.55143951	11.08236897	0.001177946	0.002800488
P17213	1.46750109	19.67300185	16.27184659	0.000115913	0.000364201
P17405	1.019751983	17.88325112	10.75455602	0.001492487	0.003477415
P17480	1.045840832	19.76252013	33.12485668	7.09533E-08	6.35011E-07
P17655	1.278670886	22.5867157	63.09705318	1.30629E-12	1.34773E-10
P17900	1.083760456	21.7904624	31.45499729	1.39068E-07	1.10958E-06
P17931	1.175716131	23.13201495	22.91010638	5.01635E-06	2.32516E-05
P18031	1.309906776	19.3497953	42.4342695	2.28062E-09	3.77562E-08
P18065	1.344530643	19.58161674	8.239501517	0.005421933	0.010750446
P18084	1.514552413	17.9902136	28.12732684	6.64512E-07	4.12066E-06
P18283	1.287027388	22.54060687	21.59397007	8.91833E-06	3.85602E-05
P18428	1.449285769	21.95184809	45.88957153	5.22118E-10	1.16553E-08
P19320	1.2893086	17.24944845	17.94535933	4.93326E-05	0.000170443
P19367	1.159141991	20.33444255	32.29330075	9.76613E-08	8.21949E-07
P19447	1.208938801	14.93597972	12.94286169	0.000748665	0.001883149
P19474	1.030382957	19.68638008	40.91340768	3.34652E-09	5.14796E-08
P19623	1.336081484	20.95075909	54.15918086	2.71141E-11	1.16727E-09
P19784	1.030338221	20.69916431	45.67753045	5.64427E-10	1.22374E-08
P19838	1.011084259	19.73002539	26.56404168	1.03918E-06	6.0668E-06
P19878	1.865259866	18.22364541	59.65592332	5.68861E-12	3.50934E-10
P20036	1.02686378	22.420327	23.51536709	3.89231E-06	1.88139E-05
P20292	1.46306292	21.51523503	17.01597828	0.000126987	0.000394339
P20585	1.110175475	15.86844023	31.89266366	1.63166E-07	1.27466E-06
P20591	1.231444847	20.09283911	10.74237285	0.001402543	0.003287434
P20592	1.287251856	16.2650521	11.85609073	0.001028801	0.002481516
P20701	1.382961834	16.71549424	27.00623484	1.07818E-06	6.2639E-06
P20702	2.018064846	16.75594432	18.58420222	6.28779E-05	0.000211382
P20774	1.080414549	23.2001465	22.89934064	4.99879E-06	2.31882E-05
P20908	1.830628419	16.84437921	25.31553221	3.18569E-06	1.57417E-05
P20933	1.056556333	19.83922596	16.58681926	8.94727E-05	0.000288472
P20936	1.266466833	16.3627017	20.48689739	1.69333E-05	6.70606E-05
P21266	1.026125166	23.26888843	30.16259985	2.32451E-07	1.70993E-06
P21283	1.141816808	21.11672154	75.33917628	2.61777E-14	1.20498E-11
P21333	1.015293231	24.73998003	48.22721404	2.22731E-10	6.29531E-09
P22352	1.325826991	21.8145816	39.46989352	5.92297E-09	8.23458E-08
P22681	1.321806175	16.07044796	13.92594732	0.00039893	0.001081781
P22894	1.390061811	18.48045426	13.85639659	0.000318191	0.000884641
P23219	1.009416629	17.53872482	10.23801227	0.001958805	0.00440518
P23229	1.57946251	17.81852085	39.98221405	5.19067E-09	7.30846E-08
P23258	1.106722284	19.92258198	41.89237218	2.31063E-09	3.79858E-08
P23368	1.295301322	21.22190398	52.06626482	5.64956E-11	2.07415E-09
P23381	1.133460012	23.44165983	59.14593999	4.89451E-12	3.14933E-10
P23469	1.920470136	16.00905279	12.40080701	0.001252718	0.002961779
P23743	1.408142947	16.62245991	11.30835	0.001588799	0.003679324
P24158	1.228447091	23.53823483	31.29768932	1.46198E-07	1.15721E-06
P24821	1.357894914	21.83319321	27.81877721	6.13504E-07	3.84823E-06
P25098	1.198927953	17.15557817	29.16372823	4.37742E-07	2.90727E-06
P25205	1.87089762	16.80497119	33.47092623	8.35694E-08	7.23703E-07
P26006	2.669120463	16.50186035	10.70917729	0.002572855	0.005582294
P26447	1.361776474	23.88712092	43.69680587	1.1749E-09	2.21785E-08
P27708	1.150002459	19.30566021	50.67678063	9.24654E-11	3.02952E-09
P28068	1.266313832	19.16597192	13.40486923	0.000607328	0.001565137
P28161	1.133885045	20.87448477	17.59247111	5.35628E-05	0.000183679
P28340	1.202496577	14.7668608	6.356157616	0.014695992	0.02579666
P28676	1.507095209	21.78919588	46.38656023	4.35149E-10	1.02517E-08
P28907	1.197853312	18.86932834	22.41208018	8.85304E-06	3.83888E-05
P29466	1.606779106	20.38079816	48.38623891	2.10287E-10	6.07903E-09
P30511	1.005533503	17.87208118	12.86840643	0.000555058	0.001446003
P30876	1.334141752	17.94496183	47.7102943	2.91863E-10	7.76227E-09
P31483	1.457306833	16.86470769	12.05139576	0.000892456	0.002191405
P31949	1.113514646	26.08797655	28.75026834	4.16276E-07	2.79887E-06
P32121	1.034497831	18.12627295	15.05484684	0.000189558	0.000561543
P32456	1.586955139	18.21691028	25.78791332	1.93041E-06	1.02955E-05
P32942	1.730131479	17.33453792	23.07331777	1.25059E-05	5.14317E-05
P33241	1.062718286	22.43257725	27.75540955	6.29972E-07	3.93092E-06

P33316	1.318794572	20.22405091	57.96150427	7.59074E-12	4.1651E-10
P33991	1.214832152	16.78516702	8.200912486	0.005636675	0.011128295
P33992	1.607513368	16.74116339	15.66832584	0.000200361	0.000589459
P33993	1.368288868	17.41469146	9.959628385	0.002276474	0.005030435
P34910	1.272486144	18.31255052	25.3720864	2.93545E-06	1.46994E-05
P35052	1.459456222	17.53951595	16.21460236	0.000175525	0.000523599
P35237	1.406056009	21.80032889	16.60568206	8.42477E-05	0.000273246
P35442	1.751513315	19.36239355	12.24137395	0.000767611	0.001923527
P35475	1.112312873	16.81918754	13.19995731	0.000634459	0.001625953
P35610	1.461693493	17.04365257	7.17673729	0.010439591	0.019168614
P36222	1.77166466	18.04021153	13.97224012	0.000524007	0.001373481
P36954	1.037923617	18.98566778	13.96105755	0.000312622	0.000870107
P39059	1.380056343	17.35386439	10.5100938	0.001943472	0.004377017
P39748	1.414579402	18.86073492	40.93035056	3.79891E-09	5.71172E-08
P40121	1.132244391	22.17710891	12.75782481	0.000517688	0.00136169
P40123	1.112768824	19.39034428	21.71183432	8.87895E-06	3.84536E-05
P40199	1.018244405	19.39900397	6.273143971	0.014957229	0.026178432
P41218	1.449797417	20.37978137	36.8864719	1.63563E-08	1.86522E-07
P41240	1.059127393	21.52507806	51.4188697	7.10354E-11	2.50045E-09
P42224	1.113746198	23.77741065	55.66243738	1.60958E-11	7.89485E-10
P42226	1.021918182	19.98029507	34.3634584	4.26119E-08	4.15293E-07
P42229	1.407903309	16.74164422	16.91831881	9.42311E-05	0.000301539
P42331	1.391007943	18.86324873	46.57114369	4.28848E-10	1.01432E-08
P42768	1.263612983	18.3651105	21.47044131	1.10994E-05	4.6285E-05
P42898	1.311203003	16.73244243	30.63788544	5.63495E-07	3.59484E-06
P43121	1.150023488	18.05071226	19.36054444	2.65118E-05	9.89687E-05
P43246	1.572977379	18.70505464	25.72018812	1.51584E-06	8.33712E-06
P43304	1.653604203	18.94023957	52.99423362	4.20822E-11	1.66768E-09
P43353	1.123209867	16.84306624	8.011101918	0.007055249	0.013605739
P43405	1.330315647	17.45526944	15.93218083	0.000135894	0.000418953
P45973	1.43059467	20.16571866	45.29096244	7.21197E-10	1.50371E-08
P46063	1.196453855	20.73720563	47.91815136	2.49102E-10	6.77557E-09
P46821	1.860198753	16.57309827	23.39686261	4.64431E-06	2.17631E-05
P46937	1.225809212	19.73440541	27.33645961	7.76475E-07	4.71719E-06
P48426	1.003988392	21.06823554	63.86255531	1.01487E-12	1.18774E-10
P48595	2.039376584	17.25147064	9.841171348	0.00312431	0.006632094
P48634	1.168942195	15.54988037	15.37349117	0.000158553	0.000479909
P48681	1.507364742	17.51293332	35.71724817	2.67571E-08	2.82888E-07
P48723	1.33567636	18.41520105	56.13227119	1.83739E-11	8.72613E-10
P48730	1.316723484	17.38416614	14.54724826	0.000318287	0.000884641
P48741	-1.852203405	23.49647404	12.2003836	0.000703535	0.001786908
P48960	1.317061015	19.29028591	26.72290618	1.01502E-06	5.94125E-06
P49137	1.009980359	18.38524815	16.70640926	9.40888E-05	0.000301245
P49590	1.125080981	18.08831887	24.58716702	2.91707E-06	1.46441E-05
P49662	1.055770228	18.49102041	18.87326411	3.39073E-05	0.000122451
P49736	2.19035442	16.61436879	29.05643645	9.3108E-07	5.53835E-06
P49747	1.514675824	17.48865255	9.201622912	0.003553952	0.007420394
P49754	1.624567716	16.65603025	56.50759908	2.97864E-11	1.24645E-09
P49902	1.155616398	20.85977743	40.23258769	4.33611E-09	6.35963E-08
P49915	1.102549918	20.38414529	52.59876021	4.68281E-11	1.80787E-09
P49916	1.283608453	16.65860988	26.76462459	9.97439E-07	5.87468E-06
P49961	1.187808092	19.07122417	21.83356936	1.22584E-05	5.05545E-05
P50452	1.3737901	17.79256764	12.02031197	0.001302616	0.00306522
P50453	1.223301375	22.30456539	59.33695785	4.58847E-12	3.0173E-10
P50454	1.218606364	23.75325588	65.56591658	5.80998E-13	7.72599E-11
P50479	1.907442598	18.36499381	5.86563044	0.023100738	0.038526983
P50749	1.538913966	17.30045718	8.784015637	0.005055707	0.010087814
P51159	1.116918951	20.71857252	28.94322518	4.20049E-07	2.81792E-06
P51911	1.27608773	21.06929439	9.545269788	0.002617255	0.005668352
P52292	2.774123355	17.31919726	18.34189451	0.000187333	0.000556053
P52435	1.142505748	19.09875004	14.92147984	0.000226869	0.000657744
P52566	1.279271577	24.02298065	69.18364298	1.89743E-13	4.44721E-11
P52630	1.113636665	20.20195762	46.261342	4.55566E-10	1.05061E-08
P52701	1.449607467	15.62446398	30.399895	3.64527E-07	2.48161E-06
P52788	1.150662202	21.77075318	52.55295785	4.7589E-11	1.82409E-09

P52948	1.052746131	18.4009798	36.91253616	1.56106E-08	1.79643E-07
P53004	1.130332847	22.17585759	61.32465955	2.35366E-12	1.8779E-10
P53634	1.013128563	22.68475771	47.45206413	2.95017E-10	7.77702E-09
P54289	1.322669689	19.0189575	27.05214773	8.64724E-07	5.17969E-06
P54652	1.69325329	19.06930477	33.08757289	9.13912E-08	7.7793E-07
P54709	1.509381731	19.04066276	31.74873702	1.79542E-07	1.38095E-06
P54922	1.348530659	17.80672743	42.66242101	9.40547E-09	1.20261E-07
P55011	1.392378865	17.02135033	11.49262382	0.001112312	0.002660764
P55039	1.127573478	20.12549244	36.70620342	1.72278E-08	1.94879E-07
P55058	1.127447641	17.53217728	15.48267962	0.000169896	0.000509093
P55160	2.003049825	17.53277744	61.70826658	2.34202E-12	1.8779E-10
P55209	1.204197847	22.1389189	44.95887024	7.35611E-10	1.52315E-08
P55809	1.78511849	17.81187689	34.74289473	1.16852E-07	9.55253E-07
P56182	1.164085086	17.38121841	5.032767121	0.02935474	0.04743688
P57678	1.219013847	15.08993031	7.68471586	0.008513024	0.016029559
P57740	1.338840975	18.31498924	33.58457616	5.99793E-08	5.4781E-07
P58107	1.54183072	15.66074544	17.58629284	5.65131E-05	0.000192692
P60709	1.450177828	28.47899835	5.780861274	0.01776032	0.030487021
P60903	1.282481439	24.9013542	49.44615302	1.43565E-10	4.33885E-09
P61018	1.026959543	19.03603763	16.72915313	8.33838E-05	0.000271179
P61289	1.591518306	20.70507	63.75343231	1.05198E-12	1.18774E-10
P61769	1.429793471	24.47258925	20.08922522	1.74793E-05	6.89493E-05
P61964	1.00453441	19.65463873	25.24092485	2.1689E-06	1.13649E-05
P62310	1.247076664	23.58977926	23.78228489	3.43848E-06	1.69131E-05
P62736	1.665400277	25.68072172	9.739366944	0.002273179	0.00502501
P62820	1.022906241	23.45866676	9.015525908	0.0032697	0.006903983
P63267	1.626328668	25.71710559	5.689131251	0.019676145	0.033425562
P63313	1.380033555	25.48557039	36.27859007	2.0728E-08	2.2759E-07
P69849	1.826806133	21.17277634	20.80258638	1.26464E-05	5.18972E-05
P78324	1.147202963	19.19178699	27.58311525	6.84694E-07	4.22393E-06
P78346	1.186718586	18.68216598	22.20633101	7.63367E-06	3.36624E-05
P78536	1.035107403	16.35587305	6.240710138	0.015766114	0.027425705
P80188	1.339437925	22.84448917	24.82119424	2.18025E-06	1.14144E-05
P80511	1.353400787	22.3255337	16.47663008	9.30701E-05	0.000298783
P98082	1.281588281	19.04741703	41.34109309	3.04051E-09	4.76294E-08
P98095	1.865493063	18.846093	44.63438878	1.01991E-09	1.98153E-08
P98171	1.304616638	17.55772296	18.56642	3.88148E-05	0.000137762
P98175	1.104320876	17.91111619	25.27190656	1.97281E-06	1.0475E-05
P98179	1.333961914	21.71157319	46.87570951	4.54941E-10	1.05061E-08
P98194	1.021025035	16.79082213	9.524853919	0.002998023	0.006403989
Q00403	1.217792135	18.12463995	17.28136586	6.91863E-05	0.000229369
Q00653	1.197544296	17.99770723	16.46969978	9.05634E-05	0.000291361
Q01432	1.959846222	16.29855181	39.35946167	2.98225E-08	3.0875E-07
Q01469	1.381622057	23.36244873	45.4581895	6.11873E-10	1.28924E-08
Q01813	2.021287633	19.98147816	23.54219802	3.78153E-06	1.83807E-05
Q01968	1.089986934	15.98549964	7.508790386	0.008993578	0.016786516
Q01970	1.303667737	17.35650358	33.38303332	6.49971E-08	5.90201E-07
Q02108	1.380212529	17.26885098	25.78982766	2.31056E-06	1.19947E-05
Q02153	1.157420858	18.39794105	25.01054966	2.04945E-06	1.08338E-05
Q02809	1.613108204	19.09926486	62.72649682	1.47667E-12	1.44945E-10
Q02880	1.038100182	18.34423576	18.87456663	2.99348E-05	0.000110234
Q03001	1.452980935	15.41774007	31.83953658	1.20746E-07	9.84392E-07
Q03169	1.741414677	18.69768166	54.38157456	2.68403E-11	1.16386E-09
Q03518	1.647945701	20.15043312	51.40444959	7.59457E-11	2.6422E-09
Q03519	1.548461862	19.41777311	38.21895277	9.57857E-09	1.21476E-07
Q05209	1.414976014	20.62292003	6.199836506	0.014183643	0.025022086
Q05397	1.059191109	18.3722143	28.50802005	4.65812E-07	3.06647E-06
Q05519	1.376497017	20.38344529	42.23384042	2.03176E-09	3.43504E-08
Q05655	1.54543335	17.23530757	35.84896451	3.02891E-08	3.1102E-07
Q05707	1.178579125	23.80014327	37.02699942	1.49288E-08	1.73801E-07
Q06033	1.244850103	19.85187427	28.30229416	5.13419E-07	3.35038E-06
Q06828	2.005368501	20.05023739	20.85495907	1.91258E-05	7.47861E-05
Q07866	1.135486877	18.97815369	19.88666247	1.91308E-05	7.47861E-05
Q08209	1.117921859	20.34501099	16.44871477	9.10071E-05	0.000292631
Q08380	1.029337684	23.21974779	50.78305171	8.90326E-11	2.95984E-09

Q08623	1.028248951	21.33039913	21.95895677	7.77915E-06	3.4178E-05
Q08945	1.217683515	20.08155659	68.27608675	2.41848E-13	4.82406E-11
Q08AF3	1.852868899	16.85243984	33.40631274	1.09447E-07	9.04598E-07
Q0JRZ9	1.165731556	19.07042223	29.27135227	3.3968E-07	2.35318E-06
Q0VD83	1.506165277	16.7256079	18.33181411	4.68216E-05	0.000162991
Q0ZGT2	1.147631286	17.83694783	16.30377854	0.000104746	0.00033222
Q10570	1.020243904	17.3985693	26.48495459	1.09738E-06	6.35082E-06
Q12765	1.64362412	19.92733538	51.71399503	6.59966E-11	2.36481E-09
Q12769	1.094957728	18.79673524	39.70603053	5.30245E-09	7.44832E-08
Q12788	1.049757306	16.83074724	22.73053208	6.16266E-06	2.7874E-05
Q12802	1.330257323	17.09660049	47.01746782	3.74802E-10	9.11714E-09
Q12805	2.070723726	20.09980526	40.01755072	5.12169E-09	7.22834E-08
Q12849	1.156198493	18.9107684	23.42944986	4.07507E-06	1.95238E-05
Q12888	1.881319662	16.43581482	42.80742626	1.98791E-09	3.37945E-08
Q12913	1.063044839	16.5218488	19.98891644	2.06378E-05	7.95726E-05
Q12929	1.408301644	18.66769878	38.7345751	8.17385E-09	1.075E-07
Q12933	1.337394597	16.46329479	9.195720292	0.004205826	0.008572092
Q12955	1.486054761	16.26270221	26.27546451	1.22467E-06	6.99166E-06
Q12972	1.096251552	19.13555785	21.59977436	9.24775E-06	3.9898E-05
Q12979	2.73590977	16.40381295	27.47773084	3.93433E-06	1.89881E-05
Q13043	1.782457259	18.56108117	45.23469631	7.76626E-10	1.57537E-08
Q13094	1.423014386	18.37837127	24.39275185	3.50997E-06	1.7202E-05
Q13107	1.194831425	17.38816226	32.96959626	8.30567E-08	7.20306E-07
Q13153	1.008636047	18.71246759	20.54427427	1.4919E-05	6.02849E-05
Q13206	1.280263531	15.01936568	13.11389528	0.000786808	0.00196587
Q13277	1.136653585	18.15765263	11.72362841	0.001530442	0.003557951
Q13287	1.195476793	20.01835483	34.47860146	4.27669E-08	4.16125E-07
Q13303	1.496479626	18.34683359	12.62494786	0.000832957	0.00206565
Q13315	1.255533662	14.97723854	19.24610751	2.78828E-05	0.00010357
Q13325	1.479453363	18.35310006	44.79880099	9.34964E-10	1.84648E-08
Q13439	1.199816238	16.83045015	40.20915564	4.46707E-09	6.48809E-08
Q13443	1.479580756	16.03908656	7.382093656	0.011403968	0.020679196
Q13459	1.22343589	16.5788023	31.87101933	1.24673E-07	1.0109E-06
Q13488	1.463427185	18.59152153	49.16860659	1.58616E-10	4.6988E-09
Q13503	1.504069342	17.91221029	15.5198162	0.000374799	0.001023728
Q13509	1.113914999	19.56433623	12.58756565	0.000589061	0.001523968
Q13523	1.073395682	17.22879781	20.63414691	1.39188E-05	5.65657E-05
Q13546	1.012060885	19.49703989	45.76044223	5.76415E-10	1.24522E-08
Q13614	1.822748065	16.1753192	15.14970685	0.000701283	0.0017827
Q13636	1.515039356	18.91745905	20.18795245	2.44261E-05	9.21599E-05
Q13643	1.899357218	19.08735909	35.22455585	5.2968E-08	4.98366E-07
Q13795	1.250033582	18.51118359	32.12931735	1.94562E-07	1.47375E-06
Q13951	1.40851322	19.60419106	42.31127178	2.64722E-09	4.28134E-08
Q13976	1.103224114	17.58438605	12.41846578	0.000755092	0.00189533
Q14008	1.352684637	18.04044341	76.95240853	1.59405E-14	9.53877E-12
Q14112	1.322715816	19.79927611	25.74104206	1.47259E-06	8.14416E-06
Q14116	1.423252856	19.94563645	30.97708146	2.02246E-07	1.52041E-06
Q14137	1.224047467	17.54664836	26.58653592	1.21218E-06	6.92809E-06
Q14149	1.167007447	15.35646275	15.14121705	0.000335859	0.000929163
Q14155	1.092039535	17.86888453	24.15339739	3.13283E-06	1.55061E-05
Q14185	1.42953005	15.87437864	30.00078273	2.94001E-07	2.06977E-06
Q14192	1.269900062	19.95529564	19.8945541	2.00165E-05	7.74767E-05
Q14257	1.268114739	20.35789708	34.02125657	5.04175E-08	4.76616E-07
Q14410	1.082891001	18.49754328	22.41924945	7.6675E-06	3.37867E-05
Q14451	1.386455239	16.35154098	9.192553108	0.003954843	0.008124195
Q14562	1.533235183	14.85009726	24.58578661	4.80254E-06	2.2487E-05
Q14566	1.375171076	16.6628102	9.001112849	0.004028586	0.00825867
Q14669	1.147519506	17.50714017	33.02472901	7.38573E-08	6.52825E-07
Q14671	1.253247691	16.76748622	25.96491279	2.32878E-06	1.20653E-05
Q14676	1.140206674	14.4637815	9.111554784	0.003561094	0.007432713
Q14690	1.206594503	16.64244108	20.86342639	1.24875E-05	5.13927E-05
Q14692	1.303162369	15.41237219	19.59041159	2.97496E-05	0.000109687
Q14746	1.138779544	17.49765907	25.4756221	1.94337E-06	1.03462E-05
Q14764	1.502270274	23.12249777	71.48904005	8.70311E-14	2.8933E-11
Q14766	1.461948202	17.20485784	21.51337762	1.04781E-05	4.41868E-05

Q14767	1.965015047	17.29614399	25.4465968	2.64645E-06	1.35123E-05
Q14790	1.504966818	18.90573833	44.78891614	8.44489E-10	1.68447E-08
Q14839	1.403817833	18.85597927	85.33957197	1.29208E-15	2.73266E-12
Q14919	1.167300512	19.91230149	21.13099324	1.13535E-05	4.7213E-05
Q14956	1.206871362	20.60888351	15.91883214	0.000124119	0.000386436
Q14997	1.312156308	16.66183975	27.05042113	8.75078E-07	5.23647E-06
Q14CX7	1.176564942	17.12166965	9.574561669	0.002535849	0.005511994
Q15007	1.107824319	17.93305345	24.61944074	2.7766E-06	1.41206E-05
Q15020	1.140329891	19.26663676	56.43786404	1.23223E-11	6.30227E-10
Q15042	1.100532109	18.811997	37.19101056	1.40042E-08	1.64963E-07
Q15061	1.257488999	16.8274801	30.29560009	2.88292E-07	2.03436E-06
Q15063	2.03749978	23.00218163	22.93588198	1.08733E-05	4.55459E-05
Q15080	1.820507261	17.69121678	42.57739689	3.79399E-09	5.71172E-08
Q15102	1.23834477	21.72233622	45.5578393	5.89834E-10	1.26229E-08
Q15113	1.308605218	18.76645706	27.38340167	9.98581E-07	5.87562E-06
Q15120	1.128674337	17.93715254	24.94163423	2.54977E-06	1.30856E-05
Q15121	1.211455133	23.14399974	40.97949957	3.26362E-09	5.05945E-08
Q15154	1.086729307	16.15809671	15.40447497	0.000157126	0.000476556
Q15155	-2.121189028	17.40218309	8.35486771	0.004834837	0.009711871
Q15208	1.179623061	18.05968082	18.77389199	3.69272E-05	0.000132081
Q15269	1.28473632	16.64500841	31.5118263	1.82055E-07	1.39848E-06
Q15286	1.047246739	21.69762342	48.29523933	2.17319E-10	6.19916E-09
Q15386	1.043019701	17.21321405	22.16349415	7.23447E-06	3.20675E-05
Q15397	1.24945408	16.99219056	26.85618082	1.33856E-06	7.49957E-06
Q15427	1.120978274	20.84631677	47.25800923	3.43585E-10	8.46095E-09
Q15542	1.160859601	15.80619432	13.2834426	0.000639081	0.001637098
Q15582	1.091279046	24.49814703	53.58108213	3.31778E-11	1.37872E-09
Q15642	1.860709205	17.59210963	36.15709139	2.6368E-08	2.8026E-07
Q15643	1.150773456	15.72715037	23.42099566	4.36638E-06	2.07698E-05
Q15648	1.241255414	14.57844837	13.15963479	0.000531964	0.001392509
Q15751	1.145238697	12.48133638	9.145494646	0.003936342	0.008088966
Q15758	1.156258219	17.23087862	12.005618	0.00091781	0.002239876
Q15813	1.14561831	19.24780505	35.78341568	2.43002E-08	2.60596E-07
Q15833	1.074781842	20.76511407	77.1522468	1.49949E-14	9.53877E-12
Q15904	1.236966795	18.1175664	18.95853522	3.93075E-05	0.000139346
Q16204	1.261213295	18.75880073	30.28056496	2.33662E-07	1.71352E-06
Q16270	1.581371659	21.01383923	42.56180293	1.79615E-09	3.10641E-08
Q16363	1.581270354	16.95784713	18.74184119	3.29754E-05	0.000119809
Q16512	1.569964715	16.95188562	38.85288735	1.25926E-08	1.50674E-07
Q16513	1.114512937	17.62678976	21.47706064	9.4593E-06	4.05186E-05
Q16643	1.910497995	19.24881711	49.73576429	1.50306E-10	4.49714E-09
Q16647	1.852095497	19.09721004	37.19087666	1.45325E-08	1.69774E-07
Q16666	1.237089825	20.3561751	43.36011498	1.3321E-09	2.44519E-08
Q16695	-1.979403088	24.65282906	7.014635917	0.009809577	0.01811185
Q16787	2.416980011	15.41742691	13.6201336	0.000686129	0.001747146
Q2M2I8	1.005668318	17.33329669	14.1709061	0.000311025	0.000866064
Q2NL82	1.077692609	17.06605706	19.30305952	2.96021E-05	0.00010921
Q2TB90	1.871806475	19.22468749	9.870133475	0.002217255	0.004923211
Q3B8N2	1.634121253	20.93078948	32.52786142	3.40158E-07	2.35318E-06
Q3KQU3	2.058983699	16.85962247	26.57868008	6.88749E-06	3.07114E-05
Q3KQV9	1.357174209	16.50884212	28.83777586	5.31811E-07	3.44038E-06
Q3SXM5	1.091240129	17.71540975	28.56120499	5.93852E-07	3.74965E-06
Q3YEC7	1.140365718	17.68826884	21.65878764	9.48529E-06	4.06008E-05
Q460N5	1.115491451	18.67807441	43.52890285	1.25078E-09	2.32444E-08
Q4J6C6	1.065405953	15.66587778	15.07918579	0.000263858	0.00075187
Q52LJ0	1.034749412	21.24678272	62.99680076	1.35031E-12	1.36954E-10
Q53EL6	1.042480255	21.33791257	69.57127066	1.59833E-13	3.98516E-11
Q53EP0	1.33211256	17.63546474	37.68415417	1.3538E-08	1.60737E-07
Q53FA7	1.355317921	20.87893816	33.78491302	5.45074E-08	5.10441E-07
Q53GG5	2.174681471	19.80627847	44.7487479	2.26852E-09	3.77078E-08
Q53GS9	1.197117884	19.59279975	36.35267141	1.94322E-08	2.15338E-07
Q562E7	1.131775342	16.94308506	25.35323935	1.77128E-06	9.58348E-06
Q5H9R7	1.26413959	18.9220087	40.95403509	3.44073E-09	5.27931E-08
Q5HYK7	1.658247191	16.80460858	13.92920598	0.000480343	0.001278067
Q5JPE7	1.827047224	21.17272594	20.80132866	1.26534E-05	5.18972E-05

Q5JSL3	1.222699109	14.5282924	7.717010554	0.00774359	0.01473375
Q5JTH9	1.586599041	16.6580861	38.88346472	1.17875E-08	1.42786E-07
Q5JU69	1.053627327	18.54534095	26.71927181	1.19512E-06	6.85017E-06
Q5MIZ7	1.109688834	16.52732709	19.2012506	2.86612E-05	0.000106197
Q5PRF9	1.11213646	16.4925547	9.255999728	0.004379086	0.008888891
Q5SRE5	1.179120733	15.27290818	17.44600251	6.62189E-05	0.000220631
Q5SW79	1.135289402	14.92531164	15.97755536	0.000137385	0.000423115
Q5SY16	1.006643121	17.10181627	9.07371413	0.003715332	0.007711602
Q5TEJ8	1.644649895	17.33196582	32.88268434	1.16217E-07	9.54937E-07
Q5TFQ8	1.109995004	19.22348637	23.37611507	4.45084E-06	2.10544E-05
Q5TZA2	-1.059242075	18.16874639	12.40714201	0.000618579	0.00158866
Q5UIP0	1.868394348	14.03395486	7.61028424	0.010532472	0.01931545
Q5VIR6	1.057584113	17.02012937	12.00560157	0.00080165	0.001999615
Q5VT52	1.133484508	16.20288276	18.73496366	3.72385E-05	0.000132877
Q5VTL8	1.010282408	17.35037543	14.60917509	0.000269337	0.000765294
Q5VTR2	1.284636381	16.56779249	33.3587051	8.22557E-08	7.14395E-07
Q5VV41	1.027804566	16.02423356	5.67695796	0.021497403	0.036165437
Q5VW32	1.024954592	20.86235471	44.61238558	8.36194E-10	1.67912E-08
Q5VYS8	1.321683484	16.02121976	19.53049704	2.65771E-05	9.91503E-05
Q5VZ89	1.163407937	17.20766945	19.98229915	1.82117E-05	7.15555E-05
Q5VZK9	1.601918097	15.23499237	18.12597599	6.42223E-05	0.000215057
Q63HN8	1.339116017	19.25403585	79.84389471	6.62119E-15	7.12123E-12
Q66K74	1.027152073	20.37689682	42.23340021	2.03209E-09	3.43504E-08
Q68CZ2	1.0340229	20.32157231	62.6643077	1.50741E-12	1.44945E-10
Q68DQ2	1.095512995	13.0493049	8.240067794	0.005748485	0.011322889
Q69YN4	1.271906168	14.24258723	19.89555477	2.2072E-05	8.45576E-05
Q6DKI2	1.633740194	20.93073503	32.52920381	3.40003E-07	2.35318E-06
Q6DKJ4	1.317195205	18.75135033	25.68857535	1.56835E-06	8.61012E-06
Q6IN85	1.468471026	17.62373129	62.03720126	2.49845E-12	1.94165E-10
Q6NUK1	1.368676691	19.38788384	27.46750927	7.10656E-07	4.37057E-06
Q6NUQ1	1.086493312	16.83047381	17.53603144	6.26678E-05	0.000211032
Q6NXT2	-1.474584871	24.99316521	6.627672619	0.012299842	0.02210278
Q6NYC1	1.125531307	16.98857837	9.085180564	0.004417951	0.008958665
Q6NYC8	1.104679832	18.46434881	21.61600127	9.18246E-06	3.96449E-05
Q6NZY4	1.1292185	17.58513133	19.578239	2.27018E-05	8.66375E-05
Q6P1J9	1.313192121	16.93363923	22.73237471	6.15782E-06	2.78732E-05
Q6P1M0	1.005419754	18.33154701	19.73096622	2.12128E-05	8.16319E-05
Q6P1N0	1.153753173	18.26516336	26.19934519	1.2378E-06	7.04085E-06
Q6P2E9	1.259974783	18.92405801	36.49228785	1.8398E-08	2.05399E-07
Q6P4A8	1.625101047	19.6011896	39.10560302	8.45063E-09	1.10211E-07
Q6P9B6	1.678512575	17.39341376	43.98650528	1.89603E-09	3.26029E-08
Q6P9B9	1.04903173	16.11900184	14.84286732	0.00024713	0.000708928
Q6PCB0	1.510537834	17.97004671	14.50346147	0.000292277	0.000819198
Q6PCE3	1.449629255	17.94499459	31.16315091	1.78899E-07	1.37778E-06
Q6PD74	1.072228876	17.13682951	14.11001727	0.000505723	0.001333148
Q6PI78	1.015504002	18.63669036	21.72403128	1.72758E-05	6.81915E-05
Q6PIU2	1.377257164	21.15665084	49.3611746	1.48012E-10	4.45078E-09
Q6QNY0	1.034697072	18.79449744	22.84667778	5.38163E-06	2.47911E-05
Q6UX06	1.535663556	19.15773489	7.307740197	0.008428705	0.015890792
Q6VY07	1.509178016	17.77744213	30.0535206	3.17271E-07	2.21535E-06
Q6WCQ1	1.151111745	18.95261801	33.35469095	6.57362E-08	5.94208E-07
Q6YHK3	1.398711819	15.86566238	9.813379415	0.002901292	0.0062227
Q6ZNJ1	1.522675116	14.24549775	5.702020493	0.022199509	0.037200185
Q6ZVM7	1.438375436	17.30638741	29.41171347	4.46141E-07	2.95322E-06
Q709C8	1.089809661	18.48096723	48.55435074	1.979E-10	5.77675E-09
Q70J99	1.381799836	16.16436547	12.19418627	0.000818994	0.002035241
Q70UQ0	1.237066308	19.67014588	21.38176552	1.13026E-05	4.70337E-05
Q7KZ85	1.332944872	17.23016527	41.45279542	2.7279E-09	4.36465E-08
Q7KZI7	1.022477321	17.59100277	25.91800386	1.4236E-06	7.89509E-06
Q7L2E3	1.232939804	16.98510456	29.96034522	2.55851E-07	1.8446E-06
Q7L2J0	1.10307211	16.56999783	23.01258075	6.54025E-06	2.936E-05
Q7L5N7	1.17977808	17.73270617	16.62129934	9.62897E-05	0.000307469
Q7L9L4	1.054702539	21.04227444	26.72816512	1.4838E-06	8.1986E-06
Q7LBC6	1.389039136	15.50011065	19.19313965	3.6484E-05	0.000130653
Q7LG56	1.114477053	20.41289201	38.55981057	8.91928E-09	1.14781E-07

Q7Z3B4	1.15324443	19.43306449	23.63347335	3.6989E-06	1.80393E-05
Q7Z3K3	1.100123748	15.42284596	13.31723647	0.000467855	0.00124817
Q7Z417	1.113079575	18.54311107	17.96321598	4.68105E-05	0.000162991
Q7Z460	1.410591281	17.35895927	43.70912549	1.28886E-09	2.38041E-08
Q7Z478	1.039451362	17.49430892	19.73662611	2.03246E-05	7.85168E-05
Q7Z4H8	1.259470611	19.74744589	46.0053718	5.00389E-10	1.13421E-08
Q7Z4S6	1.05505213	16.44109717	22.8766112	5.45986E-06	2.50936E-05
Q7Z5K2	1.189342139	16.2700592	30.27201895	2.86056E-07	2.02336E-06
Q7Z5L7	1.464618554	17.79990442	5.332895289	0.025296749	0.041575322
Q7Z5L9	1.07708368	18.790615	20.69929173	1.32387E-05	5.40753E-05
Q7Z5R6	1.142737267	19.50077201	47.53446762	2.94191E-10	7.77702E-09
Q7Z7G0	1.636938047	16.83723086	10.10543222	0.00343832	0.007224335
Q86SZ2	1.084977884	19.44895246	28.99831672	4.22067E-07	2.82195E-06
Q86UX7	1.19623018	21.56728778	68.34715719	2.36395E-13	4.82406E-11
Q86WV6	1.38623784	18.62129954	16.51722424	9.94687E-05	0.000316439
Q86X10	1.538931329	17.06265878	38.44249969	8.96171E-09	1.15079E-07
Q86X55	1.153756214	19.60126889	28.80540788	4.32935E-07	2.87853E-06
Q86XA9	1.192223899	14.48539242	22.30585673	8.13555E-06	3.55351E-05
Q86XP3	1.206973824	19.62543845	58.17072214	6.81338E-12	3.89138E-10
Q86YV0	1.693918802	15.64644697	21.9040833	1.10641E-05	4.62681E-05
Q8IU81	1.152622951	16.27611133	12.89823028	0.000628427	0.001611186
Q8IUI8	1.175388645	18.64350988	31.22978596	1.61355E-07	1.26381E-06
Q8IUR7	1.250694523	16.82709179	22.40188699	6.698E-06	3.00006E-05
Q8IUX7	1.602740961	19.7388305	19.27286408	2.58339E-05	9.68212E-05
Q8IVD9	1.015994585	18.43322945	22.32234669	6.87061E-06	3.0659E-05
Q8IVT2	1.827981943	17.38836739	6.007217875	0.023200487	0.038671787
Q8IWB7	1.017245199	20.03320479	34.12144764	4.84519E-08	4.60217E-07
Q8IWJ2	1.306715718	15.52601838	42.01054447	2.95476E-09	4.65297E-08
Q8IWV7	1.09330542	16.025838	15.220343	0.000175714	0.000523904
Q8IWV8	1.246836016	16.22797025	30.24805511	2.62149E-07	1.87868E-06
Q8IWX8	1.045763356	19.10110165	33.26405768	6.71081E-08	6.04781E-07
Q8IWY9	1.004034925	14.73316196	15.03912141	0.000238068	0.000684903
Q8IXB1	1.217593621	18.1787117	26.89031674	9.5719E-07	5.67673E-06
Q8IXT5	1.061585208	15.54261731	11.41055094	0.001190314	0.002827644
Q8IY21	1.240003919	16.83312727	21.28315787	1.07924E-05	4.52889E-05
Q8IY67	1.237410798	17.4585183	21.16700523	1.68444E-05	6.69194E-05
Q8IYI6	1.042650949	18.36451991	24.43659876	2.5949E-06	1.32717E-05
Q8IYS1	1.10270839	16.95048987	9.981027397	0.002498745	0.005453132
Q8IYS2	1.006099535	18.51025003	14.71158695	0.000235433	0.0006783
Q8IYU8	1.142772177	16.91882073	10.37545893	0.002672881	0.005780456
Q8IZ07	1.65358478	17.01282022	34.34789614	5.50139E-08	5.1438E-07
Q8IZE3	1.062697911	17.06458404	20.36410473	1.76922E-05	6.96972E-05
Q8IZH2	1.370516404	15.89419352	30.89511247	2.16037E-07	1.60393E-06
Q8IZL8	1.552714922	18.64587863	61.38464041	2.40149E-12	1.89086E-10
Q8IZP0	1.009910045	21.10220419	36.85430328	1.59696E-08	1.83421E-07
Q8N122	1.148795038	16.71649015	21.47021161	9.63359E-06	4.1084E-05
Q8N129	1.286086644	19.23071594	34.28609238	5.5249E-08	5.1525E-07
Q8N1F7	1.189017729	19.48283034	49.63561165	1.34133E-10	4.07438E-09
Q8N1F8	1.29023073	16.00295692	20.80785574	2.27399E-05	8.67274E-05
Q8N1I0	1.478873407	16.67991707	17.98160737	4.78987E-05	0.000166064
Q8N2S1	1.624826071	17.82180677	33.00198339	7.56845E-08	6.67005E-07
Q8N392	1.255867898	16.85926121	15.0924307	0.000215562	0.000630154
Q8N3D4	1.356948992	16.94213024	37.34236012	1.39665E-08	1.64844E-07
Q8N3P4	1.039392194	15.11790612	10.16784542	0.002291946	0.005059021
Q8N3U4	1.056897446	17.11887043	18.41342045	3.8214E-05	0.000135953
Q8N5X7	1.085169759	17.41881397	7.867983858	0.006913966	0.013367746
Q8N6M0	1.345901532	17.7907698	30.63385076	2.81919E-07	1.99645E-06
Q8N6T3	1.245670606	19.31550597	34.99170644	4.12496E-08	4.05983E-07
Q8N8S7	1.401723994	18.19660491	30.29741262	2.49332E-07	1.80849E-06
Q8NB90	1.174981728	16.18880946	16.88316516	8.38401E-05	0.000272333
Q8NBJ5	1.01587598	20.52493688	49.84975083	1.24229E-10	3.87179E-09
Q8NBN3	1.24631794	17.27415932	21.95464327	1.167E-05	4.84619E-05
Q8NBZ7	1.089859194	17.05227199	8.300444557	0.006064278	0.011878442
Q8NCN5	1.331507861	17.74634273	27.55327665	7.63079E-07	4.65471E-06
Q8ND30	1.164880855	18.64766907	22.22163238	6.77245E-06	3.02887E-05

Q8NE86	1.154411808	18.70582812	20.24530459	1.78398E-05	7.01861E-05
Q8NEB9	1.246873626	16.89842412	35.09845932	4.58617E-08	4.39098E-07
Q8NEJ9	1.007074127	17.7892741	13.11550139	0.000605092	0.001560046
Q8NEU8	1.174117739	18.05029377	28.78370361	4.31266E-07	2.87063E-06
Q8NF37	1.156540058	17.63989371	12.59678856	0.000713153	0.001805207
Q8NF50	1.991213657	15.43909421	45.41207477	1.60815E-09	2.81379E-08
Q8NFH4	1.539943322	18.91608311	62.60898721	6.26116E-12	3.67321E-10
Q8NFV4	1.097538684	20.76911361	30.29626068	2.22956E-07	1.65324E-06
Q8NHP8	1.553430773	19.04427818	35.4682001	3.17486E-08	3.23652E-07
Q8NHV1	1.062979641	18.34183421	22.22475169	7.72409E-06	3.4011E-05
Q8NI08	1.292087554	15.68143456	30.3857116	6.37249E-07	3.96805E-06
Q8NI27	1.052379946	17.36247688	22.80639074	5.24778E-06	2.41931E-05
Q8NI36	1.423547427	16.88429911	36.93362774	1.80402E-08	2.02159E-07
Q8TAG9	1.079205456	16.65521579	23.80113991	3.95908E-06	1.90904E-05
Q8TD55	1.369535529	20.19111924	49.80867815	1.26069E-10	3.90879E-09
Q8TDD1	1.145880422	16.27283753	22.72943308	6.73075E-06	3.01248E-05
Q8TDJ6	1.544913848	14.64558911	10.21481673	0.002181872	0.004855456
Q8TDN6	1.116969711	17.82087894	19.43126032	2.74918E-05	0.000102308
Q8TDQ7	1.154798055	17.28586746	9.7571417	0.002876329	0.006178017
Q8TDW0	1.186182707	14.66312354	7.844309403	0.007561144	0.014446323
Q8TDZ2	1.628559577	17.06043753	12.25842333	0.000837291	0.002074678
Q8TEQ6	1.201598715	17.03326254	24.56435052	2.45658E-06	1.26617E-05
Q8TF05	1.381198513	17.66531861	30.00181959	2.51536E-07	1.81786E-06
Q8TF09	1.018732817	23.00818334	39.44264766	1.07788E-08	1.33818E-07
Q8WTW3	1.052694931	17.18307889	8.577857363	0.004160564	0.008488515
Q8WU39	1.99271977	21.17342577	34.20719609	5.00996E-08	4.74361E-07
Q8WU76	1.208541082	18.37525958	27.76136905	6.66194E-07	4.12255E-06
Q8WUF5	1.831755704	16.47490394	15.33219859	0.000364955	0.001000865
Q8WUM0	1.350548902	18.63724843	60.0429736	3.75983E-12	2.58607E-10
Q8WUP2	1.734956819	18.73644983	22.78549658	1.57624E-05	6.32609E-05
Q8WVV9	1.223039178	17.62737481	28.0305734	6.71344E-07	4.14584E-06
Q8WVY7	1.645450807	19.42375236	44.58043459	9.85579E-10	1.92108E-08
Q8WWP7	1.155018052	19.62969761	40.15049409	4.97871E-09	7.05986E-08
Q8WWX9	1.253616303	19.33701084	14.96941067	0.000253447	0.000724274
Q8WWY3	1.021880531	19.83351341	24.96910866	2.08598E-06	1.10172E-05
Q8WX93	1.751229197	18.6191935	43.90764293	1.19788E-09	2.24705E-08
Q8WXF1	1.241289174	19.62431637	30.16102031	2.32601E-07	1.70993E-06
Q8WXH0	1.661389457	16.99617792	63.79768577	1.03676E-12	1.18774E-10
Q8WXI9	1.317285094	17.6039381	26.31284471	1.7492E-06	9.48976E-06
Q8WXX5	1.123145456	19.39536751	22.88064789	5.40012E-06	2.48381E-05
Q8WYP5	1.030750985	15.25105827	8.204151656	0.005295082	0.010530333
Q92484	1.110121081	18.54277514	24.53314212	2.78264E-06	1.41233E-05
Q92542	1.111884283	19.51565621	34.08352623	4.83983E-08	4.60217E-07
Q92599	1.082313557	20.88929374	25.12939659	1.91099E-06	1.02102E-05
Q92608	1.865843511	17.48695652	51.05429126	9.44866E-11	3.02952E-09
Q92609	1.306912803	17.56820465	26.98451745	1.01569E-06	5.94125E-06
Q92615	1.066978842	16.941075	24.98028004	2.9328E-06	1.46984E-05
Q92619	1.532686007	17.88119682	45.2080118	8.05903E-10	1.62923E-08
Q92621	1.868721758	18.38951228	54.85374103	2.12958E-11	9.72779E-10
Q92626	2.405645254	17.77892115	47.04138237	4.16343E-10	9.92589E-09
Q92665	1.05117357	19.0184442	19.18095464	2.72797E-05	0.000101616
Q92743	2.153799312	17.39682938	17.7363644	0.000141019	0.000432971
Q92769	1.354115236	19.07338481	21.86459855	9.28549E-06	4.0032E-05
Q92783	1.120440968	19.98587492	38.28931497	9.50574E-09	1.21284E-07
Q92785	1.162895175	17.17928122	35.80127566	3.5995E-08	3.62617E-07
Q92797	1.046930282	17.75840322	41.99633422	2.32398E-09	3.81006E-08
Q92835	2.0657267	16.39758932	46.21290936	1.18053E-09	2.22148E-08
Q92844	1.083110627	17.38842203	20.77418617	2.39623E-05	9.06387E-05
Q92878	1.054160016	19.23371147	52.79884365	4.36465E-11	1.7183E-09
Q92888	1.083571919	20.08658811	61.3903252	2.30262E-12	1.8779E-10
Q92896	1.269453305	20.01602634	75.73960381	2.31359E-14	1.15371E-11
Q92974	1.741339834	16.68385314	33.45947712	9.12834E-08	7.7793E-07
Q93062	1.152791171	19.84096455	31.84214761	1.30085E-07	1.04768E-06
Q93084	1.877767856	16.3123066	38.43855082	2.72763E-08	2.87869E-07
Q969G3	1.171190116	18.33540602	28.76295697	5.14194E-07	3.35178E-06

Q969S3	1.349839388	17.03563289	29.52811008	5.31189E-07	3.44008E-06
Q969S9	1.076892347	16.98315732	11.82576482	0.000850843	0.002103904
Q969X6	1.142658005	16.38549562	14.96447396	0.000313355	0.000871741
Q96A72	1.143242247	21.42659258	17.43053658	5.95845E-05	0.000201785
Q96AX1	1.537398099	17.39241594	34.20337325	5.59615E-08	5.20498E-07
Q96AY3	1.775346426	17.7620696	33.07699479	8.84101E-08	7.59033E-07
Q96AZ6	1.97316643	18.34281989	8.810896056	0.00624243	0.01219561
Q96B97	1.341412639	18.21213388	28.0298445	6.03545E-07	3.7977E-06
Q96BJ3	1.018270372	19.59103738	24.89206833	2.11509E-06	1.11415E-05
Q96BX8	1.198071666	17.88757083	8.855376009	0.005547694	0.010963475
Q96C90	1.568303401	20.85832056	29.68759938	3.3858E-07	2.35042E-06
Q96CG8	2.088169192	18.67202312	5.507616129	0.030742836	0.049254386
Q96CV9	1.024608147	19.24524759	25.27920494	1.81005E-06	9.76633E-06
Q96CW5	1.06193633	16.39704129	11.25114089	0.001191767	0.002829974
Q96CX2	1.101193338	22.81497034	56.90282356	1.05047E-11	5.41896E-10
Q96D15	2.145103427	19.46424659	67.50073943	3.74701E-13	5.55096E-11
Q96D96	1.228087568	19.31826564	16.65940005	0.000152206	0.000464693
Q96DZ1	1.042998139	19.30139624	39.42347563	6.15372E-09	8.46525E-08
Q96F07	1.196751788	17.76581086	23.17823171	4.58204E-06	2.15318E-05
Q96F15	1.297053169	18.71178311	27.1761101	1.06836E-06	6.21892E-06
Q96FX7	1.211618283	17.32694062	25.01009418	3.26089E-06	1.60867E-05
Q96HP0	1.323686712	14.17739587	7.231054439	0.009432218	0.017534139
Q96HQ2	1.300305834	18.53446645	16.74640499	9.87537E-05	0.000314498
Q96HW7	1.005891588	15.28097052	11.77874365	0.00087642	0.002161787
Q96JB2	1.360505273	17.90026132	37.77040073	1.16117E-08	1.41805E-07
Q96JC1	1.549707234	15.84426707	66.59811613	7.07646E-13	9.00969E-11
Q96JG6	1.243473281	17.17053249	48.38441083	2.50263E-10	6.77636E-09
Q96JH7	1.20659149	18.36870482	12.75081243	0.000519438	0.001365092
Q96JJ3	1.542232112	17.51457214	35.75207505	4.47735E-08	4.30747E-07
Q96JJ7	1.347690755	18.66847871	30.37926992	2.60808E-07	1.87356E-06
Q96JM3	1.175121438	15.98309125	10.4155561	0.001733243	0.003966129
Q96KP1	1.353459701	18.98799278	43.95875769	1.09163E-09	2.09369E-08
Q96LD4	1.850137299	17.64436221	43.64670063	1.67629E-09	2.92446E-08
Q96ME1	1.915281744	15.41149815	6.295434661	0.017404891	0.029928411
Q96ME7	1.746567734	17.39038489	36.47417195	3.25654E-08	3.31414E-07
Q96N67	1.0442863	15.82493667	16.68068622	8.38753E-05	0.000272333
Q96NT0	1.051497021	18.59712141	9.660969619	0.002765167	0.005960648
Q96P11	1.13387245	15.5617246	7.023053534	0.012291479	0.022101025
Q96P48	1.538291567	17.79991366	60.97639545	2.75143E-12	2.05807E-10
Q96PP8	1.06473574	17.46534394	8.861826449	0.003867236	0.00796885
Q96PP9	1.160297366	18.00105676	12.68393529	0.000550499	0.001436628
Q96PU8	1.082692604	20.58114799	32.75102074	8.12094E-08	7.07361E-07
Q96Q05	1.010366307	16.16109763	9.768908803	0.00250577	0.005459241
Q96Q15	1.056915543	14.19016553	7.655823863	0.00694634	0.013408117
Q96RE7	1.014913832	16.64936126	7.049341235	0.011650646	0.021062678
Q96RN5	1.234028883	15.66776589	9.424632747	0.003304548	0.006970186
Q96RQ1	1.229461196	19.13955101	30.20683138	2.51157E-07	1.81732E-06
Q96RQ9	2.260307065	17.16340505	12.50924516	0.001142131	0.002723998
Q96RS6	1.281957213	18.11404339	26.72376125	1.22698E-06	6.99259E-06
Q96RT1	1.004163787	17.76145372	40.03763668	4.76862E-09	6.82665E-08
Q96RU3	1.230410024	19.02385027	32.56991033	8.99917E-08	7.71505E-07
Q96S52	1.263003974	18.81672535	32.81691222	9.28203E-08	7.88972E-07
Q96S59	1.129002952	16.96745367	8.604210064	0.00502254	0.010030011
Q96SL4	1.111161492	18.40843213	11.03942063	0.001659454	0.003813431
Q96ST3	1.468792591	16.70607535	28.58558236	5.20729E-07	3.38332E-06
Q96T51	1.027867301	19.56557506	33.64678009	5.67028E-08	5.24435E-07
Q96T60	1.366795608	17.78589479	29.35135967	3.46076E-07	2.38065E-06
Q96T76	1.161108316	18.75323185	41.40295953	2.84113E-09	4.50964E-08
Q96TA1	1.277451027	21.69986555	62.02588746	1.86322E-12	1.6641E-10
Q99439	1.308159233	23.00517182	50.61055616	9.46725E-11	3.02952E-09
Q99598	1.043401874	21.24419904	46.04253249	4.93613E-10	1.12311E-08
Q99622	1.627868226	19.92771777	68.1490664	4.14175E-13	5.76378E-11
Q99715	1.803965418	18.64915013	20.16435363	1.69044E-05	6.69908E-05
Q99828	1.42504757	18.49366987	19.64865348	3.09667E-05	0.000113506
Q99873	1.034480624	21.78536927	58.84089286	5.42693E-12	3.39818E-10

Q99880	-2.896069109	27.85093155	7.523589443	0.007896555	0.014986674
Q99996	1.17345605	15.19982788	30.58324051	2.0661E-07	1.54351E-06
Q9BQ67	1.041655086	18.76121688	20.1391874	1.83887E-05	7.22034E-05
Q9BQA9	1.002963939	21.46831909	25.84070064	1.44041E-06	7.97356E-06
Q9BR61	1.035726067	17.17447483	19.97450573	4.67487E-05	0.000162943
Q9BRR6	1.08870689	20.8629044	54.4186208	2.47719E-11	1.09804E-09
Q9BS40	1.769209271	22.52679197	55.75896604	1.66957E-11	8.12253E-10
Q9BSQ5	1.033343657	16.95749224	10.78590131	0.001620538	0.003740199
Q9BT22	1.213612449	19.2408902	37.10743075	1.5591E-08	1.79643E-07
Q9BTC0	1.176646869	15.24004273	27.67442576	8.22179E-07	4.9596E-06
Q9BTE3	1.217431842	17.26665166	35.23776424	4.25509E-08	4.15293E-07
Q9BTU6	1.269580224	18.79944709	36.55398467	1.7959E-08	2.01626E-07
Q9BTW9	1.05906899	17.90012203	18.40296445	3.70472E-05	0.000132353
Q9BTY2	1.14039059	19.71384019	25.78405129	1.50637E-06	8.29267E-06
Q9BUF5	1.48976734	23.01076511	14.86057257	0.000189108	0.000560485
Q9BVJ6	1.378202469	16.22359141	19.96326698	2.29494E-05	8.73041E-05
Q9BVL2	1.121716838	18.78461437	22.5208377	7.18148E-06	3.18562E-05
Q9BVM2	1.160540454	17.16904569	8.688175164	0.005026892	0.010033664
Q9BVP2	1.204257594	17.45271807	23.7115019	4.3057E-06	2.05138E-05
Q9BW27	1.039379037	18.46697858	20.28822432	1.62214E-05	6.46695E-05
Q9BW30	1.062398156	19.17598713	12.52304281	0.000648499	0.001655553
Q9BXD5	1.159578895	20.61091264	28.4511188	4.76943E-07	3.12942E-06
Q9BXJ9	1.022895793	19.18994929	21.42879972	9.59073E-06	4.09562E-05
Q9BXN1	1.060130145	21.41400803	11.88580868	0.000798244	0.001991948
Q9BY32	1.276151518	21.80142073	35.68147449	2.76317E-08	2.89576E-07
Q9BYG3	1.067128996	18.61528806	21.4737969	1.18196E-05	4.90151E-05
Q9BZ29	1.092270706	14.3848288	6.469461256	0.013774954	0.02438016
Q9BZF1	1.303100285	17.93793972	40.04876317	4.84912E-09	6.90883E-08
Q9BZF9	1.371135576	17.03278725	23.22029837	4.53988E-06	2.13742E-05
Q9BZG1	1.400341122	17.87663813	16.37775905	0.000132328	0.000408802
Q9BZQ8	1.364267037	17.78942777	18.8319281	3.32358E-05	0.000120535
Q9BZV1	1.142026936	20.12085778	31.90973687	1.14044E-07	9.39996E-07
Q9BZZ2	1.226947825	18.8925664	26.45187622	1.13719E-06	6.55582E-06
Q9C005	1.412578001	21.59798887	19.27404108	2.63536E-05	9.84394E-05
Q9GZL7	1.570807963	17.6121436	36.41979	2.92502E-08	3.03351E-07
Q9GZM3	1.142364079	19.09859705	14.91198953	0.000227831	0.000658938
Q9GZN8	1.177743478	19.19567916	14.09830308	0.000321436	0.000892564
Q9GZQ8	1.033749479	19.96235685	13.4563266	0.000568965	0.001475807
Q9GZR7	1.214924433	15.78469602	10.73663862	0.001549372	0.003600561
Q9H0A0	1.005464619	18.78957226	17.77104276	4.93716E-05	0.000170479
Q9H0D6	1.229939166	19.18548897	45.46338339	6.10704E-10	1.28924E-08
Q9H0L4	1.245816344	17.11180003	8.808633306	0.003956718	0.008125257
Q9H0Q0	1.249061062	18.70931711	25.52528008	2.21244E-06	1.15627E-05
Q9H0S4	1.474584275	17.34114351	31.1566686	1.88323E-07	1.43828E-06
Q9H1A4	1.171214913	15.26554836	16.76465439	0.000115072	0.000362418
Q9H1A7	1.142117904	19.09895149	14.91481091	0.000227545	0.000658428
Q9H1C4	1.715155823	19.03593602	80.60184186	2.0344E-14	1.10671E-11
Q9H270	1.442828706	17.37730583	34.94042801	3.97112E-08	3.9343E-07
Q9H299	1.139178811	24.2835367	19.02029954	2.78653E-05	0.00010357
Q9H2M9	1.072469583	18.71270766	40.93843664	3.31487E-09	5.11243E-08
Q9H2P0	1.500446035	15.91847605	21.47599085	1.56246E-05	6.27923E-05
Q9H2U1	1.085797488	17.13294967	26.28286286	1.33975E-06	7.49957E-06
Q9H2V7	1.192058	19.68839851	42.63135196	1.83232E-09	3.15983E-08
Q9H3H3	1.059057418	18.96387553	25.47212435	1.81365E-06	9.76633E-06
Q9H3K6	1.230376862	21.88456414	43.12060594	1.45683E-09	2.60229E-08
Q9H3S7	1.042794879	19.04783038	48.40781621	2.08654E-10	6.0611E-09
Q9H3U1	1.026918491	19.00313837	23.33998879	4.16284E-06	1.98965E-05
Q9H425	1.304015101	17.85521318	11.20700603	0.001733856	0.003966129
Q9H4A6	1.236254395	20.94530938	41.28620316	2.96921E-09	4.66346E-08
Q9H4G0	1.178380522	18.06386377	15.98704174	0.000120311	0.000376144
Q9H583	1.194458419	16.51815888	14.09754975	0.000277259	0.000782234
Q9H5N1	1.190240922	17.72519631	28.73692685	4.51101E-07	2.98275E-06
Q9H6T3	1.024882973	17.20507464	22.23585888	8.28316E-06	3.61271E-05
Q9H6Y2	1.203738313	16.41351763	14.35214058	0.000459023	0.001225701
Q9H7D7	1.273075069	18.21829529	32.54529019	9.51904E-08	8.04547E-07

Q9H832	1.271958323	19.79794521	35.44270049	3.03293E-08	3.1102E-07
Q9H9A5	1.101481926	17.05932259	21.91586775	9.58802E-06	4.09562E-05
Q9H9H4	1.357465695	17.78199162	12.42816117	0.000988813	0.002398482
Q9HAV4	1.414438962	17.00606011	31.58518574	1.3984E-07	1.11277E-06
Q9HB40	1.029040627	20.85199134	26.01228683	1.31242E-06	7.38809E-06
Q9HBI0	1.770657724	18.14246133	40.76551667	9.8034E-09	1.22984E-07
Q9HCS7	1.209279955	15.69767756	23.05447874	7.364E-06	3.25452E-05
Q9HD26	1.104368584	18.23872388	17.54801237	6.28081E-05	0.000211288
Q9HD45	1.015240589	21.0230265	24.02822721	3.09269E-06	1.53455E-05
Q9NPY3	1.495751001	17.31848377	38.7479293	2.77547E-08	2.90357E-07
Q9NQ88	1.143084252	19.24869855	24.50372716	2.81732E-06	1.42751E-05
Q9NQP4	1.137962864	22.41210237	36.67168643	1.77821E-08	2.00015E-07
Q9NR09	1.298676584	15.41313571	35.85243871	2.49275E-08	2.66368E-07
Q9NR12	1.553747109	19.80753238	33.74332741	5.72213E-08	5.28414E-07
Q9NR30	1.470349931	18.31410559	46.23780427	5.10979E-10	1.14521E-08
Q9NR99	3.002733411	15.33162181	5.295147543	0.030086902	0.048407542
Q9NRA0	-1.319001785	19.77205791	6.704722991	0.011098885	0.020199431
Q9NRH3	1.106635834	19.92285142	41.91574573	2.29036E-09	3.77562E-08
Q9NS15	1.478622212	15.15067931	18.82401047	4.16115E-05	0.000146905
Q9NTI5	1.566064921	17.91357289	29.9143531	2.574E-07	1.85353E-06
Q9NTZ6	1.026087447	19.31159728	20.44998141	1.46883E-05	5.94285E-05
Q9NUB1	1.556522934	17.12471114	12.04812557	0.000920755	0.002246146
Q9NUQ9	1.23307569	22.52356351	54.10358367	2.76447E-11	1.18161E-09
Q9NUY8	1.126990471	18.39489214	26.63289854	1.05403E-06	6.14749E-06
Q9NV70	1.137959616	18.16897847	24.35438186	2.71301E-06	1.38167E-05
Q9NV88	1.278229571	17.24688242	17.50036872	8.22691E-05	0.000268136
Q9NVQ4	1.002516463	17.97060387	14.86047149	0.000326972	0.000907083
Q9NW15	1.110465321	18.11328043	17.68458487	5.82303E-05	0.000197758
Q9NW82	1.203153379	16.46954684	11.16293727	0.001925206	0.004342937
Q9NX55	1.040459665	20.5583701	28.09902769	5.94029E-07	3.74965E-06
Q9NXF1	1.065986643	16.89378819	20.13889885	1.91667E-05	7.47951E-05
Q9NXN4	1.126625894	16.48204886	14.84875672	0.000283287	0.000796612
Q9NYB0	1.385477544	18.05147995	33.28098691	1.02082E-07	8.50776E-07
Q9NYY8	1.076465417	16.62605133	21.48303649	1.35336E-05	5.52046E-05
Q9NZD2	1.035663107	19.81451871	27.39458806	7.57865E-07	4.62761E-06
Q9NZM1	2.067970763	17.75660866	37.79796242	1.17137E-08	1.42469E-07
Q9NZM3	1.318193359	17.09933623	25.36189147	1.96557E-06	1.04458E-05
Q9NZU5	1.683490748	18.96033296	38.20452851	1.13468E-08	1.39438E-07
Q9P086	1.004533233	18.5414008	25.75028019	2.18834E-06	1.14467E-05
Q9P0J1	1.706631535	17.37223936	31.80325579	1.8719E-07	1.43608E-06
Q9P107	1.385801569	15.98791142	21.44351588	1.61084E-05	6.45197E-05
Q9P253	1.474785922	17.78781125	41.02453938	3.27804E-09	5.06869E-08
Q9P258	1.574248437	18.95948552	28.72083815	4.60094E-07	3.03551E-06
Q9P260	1.063695626	17.80329223	28.36321798	5.00607E-07	3.27391E-06
Q9P2A4	1.125886243	19.10825268	32.87437071	7.96509E-08	6.96829E-07
Q9P2I0	1.124397876	17.44612154	14.59181481	0.000219746	0.000640196
Q9UBB4	1.064386822	20.12705749	46.69231066	3.8912E-10	9.38909E-09
Q9UBB6	1.234050882	16.01403513	9.932835424	0.002444918	0.005349319
Q9UBB9	1.178759387	16.19118182	15.13107835	0.000210102	0.000615393
Q9UBC2	1.143580648	19.51780217	55.891203	1.4874E-11	7.54287E-10
Q9UBE0	1.221298262	20.79457363	53.10181052	3.92421E-11	1.58665E-09
Q9UBF2	1.511706813	18.37829951	47.67889875	3.03695E-10	7.89638E-09
Q9UBF8	1.175524439	15.43400088	8.945848279	0.004814557	0.009677632
Q9UBG0	1.723501376	17.12908144	35.32703869	4.11348E-08	4.0552E-07
Q9UBL3	1.045924065	17.31468649	17.34292028	8.5723E-05	0.000277368
Q9UBT2	1.105851702	20.79249906	67.49357777	3.11063E-13	5.11246E-11
Q9UBU8	-1.295683654	20.66515173	5.533086857	0.022352233	0.037414199
Q9UBW5	1.573122547	18.73487168	30.31685498	2.33466E-07	1.71352E-06
Q9UBX5	1.069088125	19.73790332	16.02992272	0.000115431	0.000363357
Q9UFG5	1.174837901	19.78653989	22.81608425	8.38013E-06	3.65235E-05
Q9UG63	1.121481788	17.10265768	15.96854518	0.000135759	0.000418753
Q9UGI8	1.169350081	20.43941184	27.20717086	7.92668E-07	4.8058E-06
Q9UGM6	1.484899612	18.86202052	35.00998129	6.55146E-08	5.93999E-07
Q9UGT4	1.805375037	19.61762199	50.6647669	1.0833E-10	3.42989E-09
Q9UGV2	1.300543375	18.68880171	27.49533563	8.9724E-07	5.35303E-06

Q9UH62	1.098650209	19.99387167	23.67632263	3.59938E-06	1.7597E-05
Q9UH65	1.357579941	18.96179733	39.34133013	6.22172E-09	8.53917E-08
Q9UH99	1.065409112	21.36484531	54.9416516	2.06561E-11	9.50816E-10
Q9UHD2	1.251940738	18.1747875	15.33289766	0.000153426	0.000467705
Q9UHR4	1.001777107	18.32250532	21.69885842	8.78512E-06	3.81496E-05
Q9UHY1	1.306809786	19.57778499	43.95203173	1.14932E-09	2.18334E-08
Q9UI08	1.444379241	19.86073209	50.53486748	1.00208E-10	3.18961E-09
Q9UI26	1.383940282	17.26223159	35.56305663	2.74303E-08	2.88476E-07
Q9UID3	1.13504704	18.71083576	31.6802276	1.30656E-07	1.05086E-06
Q9UIG0	1.592537507	15.44395721	24.22152206	3.6165E-06	1.76662E-05
Q9UIQ6	1.382755556	17.38924995	33.22900942	7.37109E-08	6.52581E-07
Q9UJY5	1.29491307	18.85179837	24.98892469	2.2719E-06	1.18432E-05
Q9UK41	1.158210615	19.70640541	25.12141599	2.33695E-06	1.20971E-05
Q9UK55	1.395449625	18.65500286	31.91892807	1.36922E-07	1.09832E-06
Q9UK76	1.037919729	20.64033689	15.37754186	0.000178075	0.000530414
Q9UKK3	2.433260213	17.03673547	61.18812726	3.0244E-12	2.19164E-10
Q9UKM7	1.01110684	17.62631025	25.11580416	2.34238E-06	1.21148E-05
Q9UKU9	1.669740744	16.74871771	10.58398219	0.002406852	0.005271816
Q9UL26	1.074766957	19.00837511	33.2979656	9.92098E-08	8.31473E-07
Q9UL45	1.169008304	19.63553604	18.02063754	6.52843E-05	0.000217897
Q9ULD2	1.233138077	14.88888921	10.09372454	0.002194535	0.004878194
Q9ULH1	1.53293244	15.78013382	23.17837723	7.95108E-06	3.49078E-05
Q9ULK4	1.118106074	14.60598998	18.03494505	5.32826E-05	0.000182857
Q9ULU4	-1.598518757	17.63096709	7.0376236	0.010520368	0.019299166
Q9ULZ3	1.127888168	22.95916024	54.30548382	2.57669E-11	1.13374E-09
Q9UM07	1.840712779	18.09179564	15.04514691	0.000242591	0.00069691
Q9UM22	1.393478233	19.81147636	22.55510765	6.51074E-06	2.92495E-05
Q9UM54	1.001413746	20.41766209	46.27431819	4.53405E-10	1.05061E-08
Q9UMS6	1.483537245	18.32644671	28.12738126	5.72532E-07	3.64084E-06
Q9UMZ2	1.121800137	15.94335264	14.69001116	0.000282919	0.00079595
Q9UNF1	1.526996629	17.13079051	25.70193487	2.15886E-06	1.13222E-05
Q9UP83	1.014266981	17.9574764	14.51893289	0.000227391	0.000658315
Q9UPN7	1.233511615	18.26206823	32.19331668	1.11293E-07	9.18587E-07
Q9UPT5	1.286456691	19.19276326	41.9176312	2.28873E-09	3.77562E-08
Q9UPU7	1.293967165	16.61813114	32.46430414	1.049E-07	8.71837E-07
Q9Y217	1.192829459	16.91657829	31.55733401	1.78851E-07	1.37778E-06
Q9Y221	1.442541557	18.76845155	15.77222402	0.000269152	0.000765132
Q9Y287	1.005837356	18.87799895	16.45483606	9.83975E-05	0.000313531
Q9Y2D4	1.483097457	17.2126838	36.43028908	2.65107E-08	2.81277E-07
Q9Y2E5	1.151586088	18.82887804	20.30758792	1.60828E-05	6.44604E-05
Q9Y2G5	1.061233815	18.79037391	20.70744663	1.39958E-05	5.67804E-05
Q9Y2I8	1.166709124	18.73944265	26.90521095	9.19685E-07	5.47601E-06
Q9Y2J2	1.069768895	18.76509639	21.24911017	1.03812E-05	4.39019E-05
Q9Y2J8	2.047880452	16.79822184	16.79086716	0.000187734	0.000556689
Q9Y2L1	1.174425699	18.03641735	40.29501876	4.41606E-09	6.46105E-08
Q9Y2L5	1.07159346	16.4260708	26.45355742	1.18925E-06	6.82966E-06
Q9Y2T2	1.115074718	20.29674204	38.466096	8.54069E-09	1.10972E-07
Q9Y2V7	1.127060869	17.63114838	15.39110279	0.000166459	0.0005008
Q9Y2X7	1.196090679	16.59471619	14.54359253	0.000275036	0.000778163
Q9Y303	1.151813385	19.16239643	37.47781443	1.3003E-08	1.54691E-07
Q9Y312	1.001288487	18.46195893	15.92333138	0.000129967	0.00040234
Q9Y333	1.0143682	22.03678879	38.7709674	7.59323E-09	1.00973E-07
Q9Y3E0	1.063798995	21.7631345	25.82472723	1.49661E-06	8.25409E-06
Q9Y3L3	1.473067185	17.89112607	13.09131168	0.000482765	0.001281663
Q9Y3P9	1.31568611	17.10834312	30.69460605	1.97479E-07	1.49018E-06
Q9Y3R5	1.16955795	15.61033428	17.05614052	7.18545E-05	0.000237294
Q9Y4B6	1.124922771	16.26320907	25.0595345	2.31052E-06	1.19947E-05
Q9Y4E1	1.362218479	16.83012466	13.19417903	0.000451555	0.001207915
Q9Y4E6	1.114231822	15.83775429	29.589071	3.27297E-07	2.27473E-06
Q9Y4G6	1.022128225	17.31091571	19.28225689	2.49161E-05	9.37133E-05
Q9Y4H4	1.272595557	19.34138118	15.50898135	0.000167911	0.000504402
Q9Y4K0	2.178000912	15.65514497	7.485455401	0.011574349	0.020950062
Q9Y4K1	1.14654647	15.32414729	12.0721759	0.000997636	0.002416944
Q9Y4Y9	1.116305563	23.51960574	42.65450482	1.90472E-09	3.26586E-08
Q9Y520	1.012588508	17.91970771	30.48183205	2.03946E-07	1.52934E-06

Q9Y5B0	1.007914588	15.86392798	10.12726925	0.002244624	0.004974752
Q9Y5B9	1.142231702	19.23268817	55.67662357	1.60171E-11	7.89485E-10
Q9Y5J1	1.416028634	15.28524389	17.04887131	9.82872E-05	0.000313346
Q9Y5Q8	1.221320095	16.36312804	28.31785836	5.64864E-07	3.59973E-06
Q9Y5Q9	1.240056456	15.67334834	23.46875626	6.23666E-06	2.81449E-05
Q9Y613	1.087182695	15.83148737	9.800348044	0.002561258	0.005560236
Q9Y625	1.308662948	18.54331692	36.53720672	2.05967E-08	2.26564E-07
Q9Y666	1.16116265	16.92340531	17.34650812	7.6743E-05	0.000251771
Q9Y6D6	1.249519045	17.69338942	40.45588867	3.98237E-09	5.89863E-08
Q9Y6K5	1.201171273	19.53242591	24.7467071	2.27206E-06	1.18432E-05
Q9Y6K9	1.031996721	18.48372417	16.75103225	9.22403E-05	0.000296437

Supplementary Table S3. Clinical characteristics in the tissue microarray (TMA) experiment

ID	Age (years)	Sex	HBsAg	CA19-9 (U/mL)	AFP (ng/mL)	Tumor size (cm)	OS (months)	OS Status	DFS (months)	DFS Status
1	49	female	positive	58.6	10	2	26.0	No	26.0	No
2	51	female	negative	21.3	3.1	8	48.0	No	12.0	Yes
3	53	male	positive	0.6	226.8	7	37.0	Yes	5.0	Yes
4	60	male	negative	241.9	2.7	3	47.0	No	18.0	Yes
5	50	female	positive	60.6	3.4	5	47.0	No	47.0	No
6	42	male	positive	0.6	1.7	7	47.0	No	35.0	Yes
7	72	female	positive	21.6	2.1	6	46.0	No	46.0	No
8	68	female	negative	16.1	1.4	4.5	46.0	No	46.0	No
9	73	male	positive	15.2	1.8	2.8	46.0	No	46.0	No
10	47	male	positive	2.6	1	8	45.5	No	45.5	No
11	63	female	negative	91.1	2.3	10	44.0	No	6.0	Yes
12	63	female	positive	19.1	20.2	7	44.0	No	44.0	No
13	72	female	negative	22	1.5	6.5	9.0	Yes	4.5	Yes
14	60	male	negative	5.6	1.1	4	28.7	Yes	8.0	Yes
15	61	female	negative	885.7	2.5	7	44.7	No	44.7	No
16	51	male	positive	15.1	76.1	5	44.0	No	44.0	No
17	43	male	negative	3,741	957.7	6	13.5	Yes	6.5	Yes
18	62	female	positive	31.7	0.9	2.5	26.3	Yes	2.5	Yes
19	74	male	negative	7.3	7.5	6	44.3	No	44.3	No
20	47	female	negative	34.2	3.7	3	44.7	No	44.7	No
21	47	male	negative	1.2	1.5	6	44.0	No	44.0	No
22	61	female	negative	2,839	1.1	10	44.0	No	44.0	No
23	72	male	negative	11.5	3.1	4	20.7	Yes	10.0	Yes
24	28	male	negative	1,000	1.1	15	5.7	Yes	3.0	Yes
25	56	female	positive	349.4	1000	6	29.3	Yes	18.0	Yes
26	64	female	positive	16.7	1.3	5.5	26.0	Yes	8.0	Yes
27	61	female	negative	20.1	2.7	9	43.5	No	19.0	Yes
28	69	male	negative	40	43.5	9	42.0	No	42.0	No
29	42	female	positive	262.8	3.3	9	20.0	Yes	10.0	Yes
30	66	male	negative	35	3.4	9	27.7	No	27.7	No
31	53	female	positive	3,759	5	7	4.5	Yes	3.0	Yes
32	38	female	positive	5,112	2.8	9	9.0	Yes	4.0	Yes
33	42	female	positive	498.9	71.5	8	9.3	Yes	5.0	Yes
34	57	female	negative	8.3	2.3	11.5	37.7	Yes	15.0	Yes
35	62	male	negative	10,000	2.8	8.5	3.7	Yes	3.7	No
36	61	male	negative	68.2	1.2	9	3.7	Yes	2.0	Yes
37	77	male	negative	10,000	2.3	6.5	4.0	Yes	4.0	No
38	53	male	positive	24.8	3.5	2.5	39.5	Yes	5.5	Yes
39	71	female	positive	21	710.8	2	28.0	No	28.0	No
40	44	female	positive	12.1	4.4	2.5	13.0	Yes	9.0	Yes
41	56	female	negative	6.8	1.9	15	3.3	Yes	2.0	Yes
42	32	female	negative	119.6	1.7	9	39.0	No	24.0	Yes
43	42	female	positive	226	9	10	6.0	Yes	6.0	No
44	76	male	negative	101.1	4.4	9	39.5	No	39.5	No
45	68	male	positive	16.5	422.3	8	39.7	No	39.7	No
46	64	female	negative	9.5	1.6	4.5	12.0	Yes	7.0	Yes
47	50	female	positive	46.7	10.2	3.5	27.0	No	21.3	Yes
48	48	female	positive	16.3	2128	3.5	37.3	No	37.3	No
49	62	male	negative	10.1	1.4	6.5	17.0	Yes	7.0	Yes
50	67	male	negative	814.1	5.4	4	14.5	Yes	7.0	Yes
51	70	female	negative	44.8	2	5.5	28.3	Yes	15.5	Yes
52	70	male	positive	47.2	2.5	3	64.3	No	12.3	Yes
53	63	male	negative	10,000	1.7	12	39.3	No	39.3	No
54	63	female	negative	12.8	2.8	2.5	38.7	No	17.7	Yes
55	58	male	positive	33.8	4.9	2.5	39.0	No	39.0	No
56	56	male	positive	70.9	1.8	9	38.3	No	5.0	Yes
57	56	male	negative	21.5	5.3	5	38.0	No	38.0	No
58	39	female	positive	256.6	444.5	3.5	38.0	No	38.0	No
59	67	female	negative	11.7	2.1	4	38.0	No	38.0	No
60	60	female	negative	1.6	1.6	4.5	5.5	Yes	5.5	No
61	56	male	negative	305.1	417.3	4	13.5	Yes	6.5	Yes
62	74	female	negative	21.6	2.7	8	25.3	No	25.3	No
63	62	female	negative	81.8	3	5.5	37.0	No	37.0	No
64	58	male	negative	8.1	2.5	7	37.0	No	37.0	No
65	42	female	positive	8.2	153.6	4	36.3	No	13.0	Yes
66	67	male	negative	7	6	10	11.0	Yes	8.0	Yes
67	55	female	positive	80.9	5	3.5	36.5	No	36.5	No
68	64	male	positive	500	1.5	6	11.0	Yes	7.0	Yes
69	47	female	positive	12.5	3.4	3	36.5	No	36.5	No
70	54	female	positive	47.9	2.7	9	36.0	No	36.0	No
71	72	female	negative	233.5	2.2	5	15.0	Yes	12.0	Yes
72	48	female	positive	27.6	2.4	4	35.7	No	35.7	No
73	54	female	negative	16.2	3.8	5	3.0	Yes	1.0	Yes
74	53	female	negative	100.5	2.1	5	4.0	Yes	4.0	Yes
75	50	male	positive	8.1	214.8	7	35.5	No	35.5	No
76	71	female	negative	3,190	2.9	5	15.0	Yes	11.0	Yes
77	71	female	negative	125.3	1.5	5	11.0	Yes	5.0	Yes
78	39	female	negative	17.6	4.6	3	8.3	Yes	4.0	Yes
79	60	female	positive	25.8	2.8	7.5	13.0	Yes	6.5	Yes
80	70	male	negative	11.6	1.5	12	34.3	No	34.3	No
81	78	female	negative	385.2	4.1	4	17.3	Yes	8.3	Yes
82	62	female	negative	4,178	2.7	6	34.3	No	31.3	Yes

83	58	female	negative	171.2	3	4	18.0	Yes	6.0	Yes
84	77	female	negative	23.8	3	4	8.7	Yes	4.0	Yes
85	57	male	negative	24.4	56.2	6	18.0	Yes	12.0	Yes
86	57	male	positive	24.6	7.7	4	34.0	No	34.0	No
87	75	male	negative	17.5	2.6	5	34.0	No	34.0	No
88	73	female	negative	14.1	2.3	6.5	33.7	No	33.7	No

Supplementary Table S5. Results of GO enrichment analysis between ATTs subtypes DEPs.

Name	Category	GO term	M	N	UP_m	UP_n	UP_expect	UP_pval	UP_q_value	O/U	UP_Log(Q)	UP_Fold_enrichment	DOWN_m	DOWN_n	DOWN_expect	DOWN_pval	DOWN_q_value	O/U	DOWN_Log(Q)	DOWN_Fold_enrichment
GO:0043230	Cellular component	extracellular organelle	5589	1390	861	197	214.1331186	0.076186148	7.62E-02	-	-1.11812	0.92	11	7	2.7357309	0.007277858	1.46E-02	++	1.83697	2.5587
GO:0065010	Cellular component	extracellular membrane-bounded organelle	5589	1390	861	197	214.1331186	0.076186148	7.62E-02	-	-1.11812	0.92	11	7	2.7357309	0.007277858	1.46E-02	++	1.83697	2.5587
GO:0070062	Cellular component	extracellular exosome	5589	1384	861	194	213.208803	0.053220785	5.32E-02	-	-1.27392	0.9099	11	7	2.72392199	0.007093704	1.42E-02	++	1.8481	2.5698
GO:1903561	Cellular component	extracellular vesicle	5589	1390	861	197	214.1331186	0.076186148	7.62E-02	-	-1.11812	0.92	11	7	2.7357309	0.007277858	1.46E-02	++	1.83697	2.5587
GO:0000786	Cellular component	nucleosome	5589	48	861	2	7.39452496	0.015035199	1.50E-02	--	-1.82289	0.2705	11	4	0.094471283	1.51E-06	3.03E-06	++	5.51923	42.3409
GO:0005198	Molecular function	structural molecule activity	5589	414	861	61	63.77777778	0.378407257	3.78E-01	-	-0.42204	0.9564	11	4	0.814814815	0.006438229	1.29E-02	++	1.8902	4.9091
GO:0032993	Cellular component	protein-DNA complex	5589	75	861	11	11.55394525	0.507669014	5.08E-01	-	-0.29442	0.9521	11	4	0.147611379	9.19E-06	1.84E-05	++	4.73543	27.0982
GO:0044815	Cellular component	DNA packaging complex	5589	65	861	7	10.01341922	0.195041546	1.95E-01	-	-0.70987	0.6991	11	4	0.127929862	5.17E-06	1.03E-05	++	4.98516	31.2671
GO:0030527	Molecular function	structural constituent of chromatin	5589	40	861	0	6.162104133	0.001209594	1.21E-03	--	-2.91736	0	11	3	0.078726069	5.39E-05	1.08E-04	++	3.96764	38.1068
GO:0044283	Biological process	small molecule biosynthetic process	5589	279	861	27	42.98067633	0.002871484	5.74E-03	--	-2.24086	0.6282	11	3	0.549114332	0.015059285	1.51E-02	++	1.8222	5.4633
GO:0046982	Molecular function	protein heterodimerization activity	5589	117	861	11	18.02415459	0.039638555	3.96E-02	--	-1.40188	0.6103	11	3	0.230273752	0.001305079	2.61E-03	++	2.58333	13.028
GO:0006094	Biological process	gluconeogenesis	5589	33	861	2	5.08373591	0.097465264	9.75E-02	-	-1.01115	0.3934	11	2	0.064949007	0.001798747	3.60E-03	++	2.444	30.7934
GO:0006458	Biological process	'de novo' protein folding	5589	35	861	5	5.391841116	0.541875135	5.42E-01	-	-0.2661	0.9273	11	2	0.06888531	0.002022646	4.05E-03	++	2.39305	29.0338
GO:0008150	Biological process	biological_process	5589	126	861	14	19.41062802	0.10696844	1.07E-01	-	-0.97074	0.7213	11	2	0.247987118	0.024277299	4.86E-02	++	1.31377	8.0649
GO:0008180	Cellular component	COP9 signalosome	5589	26	861	2	4.005367687	0.212524221	2.13E-01	-	-0.67259	0.4993	11	2	0.051171945	0.001115542	2.23E-03	++	2.65148	39.0839
GO:0016051	Biological process	carbohydrate biosynthetic process	5589	63	861	6	9.70531401	0.127144741	1.27E-01	-	-0.8957	0.6182	11	2	0.123993559	0.006442291	1.29E-02	++	1.88993	16.1299
GO:0019319	Biological process	hexose biosynthetic process	5589	35	861	2	5.391841116	0.076999356	7.70E-02	-	-1.11351	0.3709	11	2	0.06888531	0.002022646	4.05E-03	++	2.39305	29.0338
GO:0031072	Molecular function	heat shock protein binding	5589	74	861	11	11.39989265	0.527565854	5.28E-01	-	-0.27772	0.9649	11	2	0.145643228	0.008805022	1.76E-02	++	1.75424	13.7322
GO:0042026	Biological process	protein refolding	5589	20	861	3	3.081052067	0.614908571	6.15E-01	-	-0.21119	0.9737	11	2	0.039363035	0.000656381	1.31E-03	++	2.88181	50.8091
GO:0044183	Molecular function	protein folding chaperone	5589	50	861	5	7.702630166	0.195994973	1.96E-01	-	-0.70776	0.6491	11	2	0.098407586	0.004097697	8.20E-03	++	2.08643	20.3236
GO:0046364	Biological process	monosaccharide biosynthetic process	5589	39	861	3	6.00805153	0.128008948	1.28E-01	-	-0.89276	0.4993	11	2	0.076757917	0.002508157	5.02E-03	++	2.29962	26.0559
GO:0061077	Biological process	chaperone-mediated protein folding	5589	63	861	8	9.70531401	0.348625845	3.49E-01	-	-0.45764	0.8243	11	2	0.123993559	0.006442291	1.29E-02	++	1.88993	16.1299
GO:0072562	Cellular component	blood microparticle	5589	121	861	6	18.640365	0.000276165	5.52E-04	--	-3.2578	0.3219	11	2	0.238146359	0.022501853	2.25E-02	++	1.64778	8.3982
GO:0140662	Molecular function	ATP-dependent protein folding chaperone	5589	31	861	3	4.775630703	0.274511574	2.75E-01	-	-0.56144	0.6282	11	2	0.061012704	0.001587532	3.18E-03	++	2.49825	32.7801
GO:0000015	Cellular component	phosphopyruvate hydratase complex	5589	3	861	0	0.46215781	0.605323604	6.05E-01	-	-0.21801	0	11	1	0.005904455	0.005893895	1.18E-02	++	1.92857	169.3636
GO:0000253	Molecular function	3-keto sterol reductase activity	5589	4	861	0	0.616210413	0.512021845	5.12E-01	-	-0.29071	0	11	1	0.007872607	0.007851497	1.57E-02	++	1.80402	127.0227
GO:0001727	Molecular function	lipid kinase activity	5589	2	861	0	0.308105207	0.715603676	7.16E-01	-	-0.14533	0	11	1	0.003936303	0.003932781	7.87E-03	++	2.10427	254.0455
GO:0003376	Biological process	sphingosine-1-phosphate receptor signaling pathw	5589	5	861	0	0.770263017	0.433087054	4.33E-01	-	-0.36342	0	11	1	0.009840759	0.009805594	1.96E-02	++	1.7075	101.6182
GO:0004090	Molecular function	carbonyl reductase (NADPH) activity	5589	7	861	0	1.078368223	0.309817605	3.10E-01	-	-0.50889	0	11	1	0.013777062	0.013703296	2.74E-02	++	1.56214	72.5844
GO:0004634	Molecular function	phosphopyruvate hydratase activity	5589	3	861	0	0.46215781	0.605323604	6.05E-01	-	-0.21801	0	11	1	0.005904455	0.005893895	1.18E-02	++	1.92857	169.3636
GO:0004930	Molecular function	G protein-coupled receptor activity	5589	11	861	1	1.694578637	0.47664105	4.77E-01	-	-0.32181	0.5901	11	1	0.021649669	0.021456887	4.29E-02	++	1.3674	46.1901
GO:0005985	Biological process	sucrose metabolic process	5589	3	861	0	0.46215781	0.605323604	6.05E-01	-	-0.21801	0	11	1	0.005904455	0.005893895	1.18E-02	++	1.92857	169.3636
GO:0005986	Biological process	sucrose biosynthetic process	5589	2	861	0	0.308105207	0.715603676	7.16E-01	-	-0.14533	0	11	1	0.003936303	0.003932781	7.87E-03	++	2.10427	254.0455
GO:0006000	Biological process	fructose metabolic process	5589	12	861	0	1.84863124	0.134024735	1.34E-01	-	-0.87282	0	11	1	0.023617821	0.023386606	4.68E-02	++	1.33	42.3409
GO:0006668	Biological process	sphinganine-1-phosphate metabolic process	5589	2	861	0	0.308105207	0.715603676	7.16E-01	-	-0.14533	0	11	1	0.003936303	0.003932781	7.87E-03	++	2.10427	254.0455
GO:0006669	Biological process	sphinganine-1-phosphate biosynthetic process	5589	1	861	0	0.154052603	0.845947397	8.46E-01	-	-0.07266	0	11	1	0.001968152	0.001968152	3.94E-03	++	2.40491	508.0909
GO:0008481	Molecular function	sphinganine kinase activity	5589	1	861	0	0.154052603	0.845947397	8.46E-01	-	-0.07266	0	11	1	0.001968152	0.001968152	3.94E-03	++	2.40491	508.0909
GO:0008753	Molecular function	NADPH dehydrogenase (quinone) activity	5589	2	861	0	0.308105207	0.715603676	7.16E-01	-	-0.14533	0	11	1	0.003936303	0.003932781	7.87E-03	++	2.10427	254.0455
GO:0009111	Biological process	vitamin catabolic process	5589	7	861	0	1.078368223	0.309817605	3.10E-01	-	-0.50889	0	11	1	0.013777062	0.013703296	2.74E-02	++	1.56214	72.5844
GO:0010155	Biological process	regulation of proton transport	5589	9	861	1	1.38647343	0.585423111	5.85E-01	-	-0.23253	0.7213	11	1	0.017713366	0.017587045	3.52E-02	++	1.45378	56.4545
GO:0010286	Biological process	heat acclimation	5589	5	861	0	0.770263017	0.433087054	4.33E-01	-	-0.36342	0	11	1	0.009840759	0.009805594	1.96E-02	++	1.7075	101.6182
GO:0010457	Biological process	centriole-centriole cohesion	5589	2	861	0	0.308105207	0.715603676	7.16E-01	-	-0.14533	0	11	1	0.003936303	0.003932781	7.87E-03	++	2.10427	254.0455
GO:0010669	Biological process	epithelial structure maintenance	5589	7	861	0	1.078368223	0.309817605	3.10E-01	-	-0.50889	0	11	1	0.013777062	0.013703296	2.74E-02	++	1.56214	72.5844
GO:0017050	Molecular function	D-erythro-sphingosine kinase activity	5589	1	861	0	0.154052603	0.845947397	8.46E-01	-	-0.07266	0	11	1	0.001968152	0.001968152	3.94E-03	++	2.40491	508.0909
GO:0032053	Biological process	ciliary basal body organization	5589	1	861	0	0.154052603	0.845947397	8.46E-01	-	-0.07266	0	11	1	0.001968152	0.001968152	3.94E-03	++	2.40491	508.0909
GO:0033005	Biological process	positive regulation of mast cell activation	5589	7	861	1	1.078368223	0.690182395	6.90E-01	-	-0.16104	0.9273	11	1	0.013777062	0.013703296	2.74E-02	++	1.56214	72.5844
GO:0035253	Cellular component	ciliary rootlet	5589	3	861	0	0.46215781	0.605323604	6.05E-01	-	-0.21801	0	11	1	0.005904455	0.005893895	1.18E-02	++	1.92857	169.3636
GO:0038036	Molecular function	sphingosine-1-phosphate receptor activity	5589	1	861	0	0.154052603	0.845947397	8.46E-01	-	-0.07266	0	11	1	0.001968152	0.001968152	3.94E-03	++	2.40491	508.0909
GO:0042132	Molecular function	fructose 1,6-bisphosphate 1-phosphatase activity	5589	2	861	0	0.308105207	0.715603676	7.16E-01	-	-0.14533	0	11	1	0.003936303	0.003932781	7.87E-03	++	2.10427	254.0455
GO:0042363	Biological process	fat-soluble vitamin catabolic process	5589	5	861	0	0.770263017	0.433087054	4.33E-01	-	-0.36342	0	11	1	0.009840759	0.009805594	1.96E-02	++	1.7075	101.6182
GO:0042373	Biological process	vitamin K metabolic process	5589	9	861	0	1.38647343	0.221605348	2.22E-01	-	-0.65442	0	11	1	0.017713366	0.017587045	3.52E-02	++	1.45378	56.4545
GO:0042374	Biological process	phylloquinone metabolic process	5589	4	861	0	0.616210413	0.512021845	5.12E-01	-	-0.29071	0	11	1	0.007872607	0.007851497	1.57E-02	++	1.80402	127.0227
GO:0042376	Biological process	phylloquinone catabolic process	5589	4	861	0	0.616210413	0.512021845	5.12E-01	-	-0.29071	0	11	1	0.007872607	0.007851497	1.57E-02	++	1.80402	127.0227
GO:0042377	Biological process	vitamin K catabolic process	5589	4	861	0	0.616210413	0.512021845	5.12E-01	-	-0.29071	0	11	1	0.007872607	0.007851497	1.57E-02	++	1.80402	127.0227
GO:0045125	Molecular function	bioactive lipid receptor activity	5589	1	861	0	0.154052603	0.845947397	8.46E-01	-	-0.07266	0	11	1	0.001968152	0.001968152	3.94E-03	++	2.40491	508.0909
GO:0045494	Biological process	photoreceptor cell maintenance	5589	3	861	0	0.46215781	0.605323604	6.05E-01	-	-0.21801	0	11	1	0.005904455	0.005893895	1.18E-02	++	1.92857	169.3636
GO:0045724	Biological process	positive regulation of cilium assembly	5589	9	861	0	1.38647343	0.221605348	2.22E-01	-	-0.65442	0	11	1	0.017713366	0.017587045	3.52E-02	++	1.45378	56.4545
GO:0070370	Biological process	cellular heat acclimation	5589	5	861	0	0.770263017	0.433087054	4.33E-01	-	-0.36342	0	11	1	0.009840759	0.009805594	1.96E-02	++	1.7075	101.6182
GO:0070402	Molecular function	NADPH binding	5589	11	861	1	1.694578637	0.47664105	4.77E-01	-	-0.32181	0.5901	11	1	0.021649669	0.021456887	4.29E-02	++	1.3674	46.1901
GO:0090036	Biological process	regulation of protein kinase C signaling	5589	4	861	0	0.616210413	0.512021845	5.12E-01	-	-0.29071	0	11	1	0.007872607	0.007851497	1.57E-02	++	1.80402	127.0227
GO:0090037	Biological process	positive regulation of protein kinase C signaling	5589	1	861	0	0.154052603	0.845947397	8.46E-01	-	-0.07266	0	11	1	0.001968152	0.001968152	3.94E-03	++	2.40491	508.0909
GO:0090153	Biological process	regulation of sphingolipid biosynthetic process	5589	7	861	1	1.078368223	0.690182395	6.90E-01	-	-0.16104	0.9273	11	1	0.013777062	0.013703296	2.74E-02	++	1.56214	72.5844
GO:0090520	Biological process	sphingolipid mediated signaling pathway	5589	5	861	0	0.770263017	0.433087054	4.33E-01	-	-0.36342	0	11	1	0.009840759	0.009805594	1.96E-02	++	1.7075	101.6182
GO:0097729	Cellular component	9+2 motile cilium	5589	12	861	1	1.84863124	0.427589196	4.28E-01	-	-0.36897	0.5409	11	1	0.023617821	0.023386606	4.68E-02	++	1.33	42.3409
GO:0120219	Cellular component	subapical part of cell	5589	1	861	0	0.154052603	0.845947397	8.46E-01	-	-0.07266	0	11	1	0.001968152	0.001968152	3.94E-03	++	2.40491	508.0909
GO:1901662	Biological process	quinone catabolic process	5589	5	861	0	0.770263017	0.433087054	4.33E-01	-	-0.36342	0	11	1	0.009840759	0.009805594	1.96E-02	++	1.7075	101.6182
GO:1903564	Biological process	regulation of protein localization to cilium	5589	5	861	0	0.770263017	0.433087054	4.33E-01	-	-0.36342	0	11	1	0.009840759	0.009805594	1.96E-02	++	1.7075	101.6182
GO:1903566	Biological process	positive regulation of protein localization to cilium	5589	3	861	0	0.46215781	0.605323604	6.05E-01	-	-0.21801	0	11	1	0.005904455	0.005893895	1.18E-02	++	1.92857	169.3636
GO:1904627	Biological process	response to phorbol 13-acetate 12-myristate	5589	3	861	0	0.46215781	0.605323604	6.05E-01	-	-0.21801	0	11	1	0.005904455	0.005893895	1.18E-02	++	1.92857	169.3636
GO:1904628	Biological process	cellular response to phorbol 13-acetate 12-myristat	5589	3	861	0	0.46215781	0.605323604	6.05E-01	-	-0.21801	0	11	1	0.005904455	0.005893895	1.18E-02	++	1.92857	169.3636
GO:1904732	Biological process	regulation of electron transfer activity	5589	4	861	0	0.616210413	0.512021845	5.12E-01	-	-0.29071	0	11	1	0.007872607	0.007851497	1.57E-02	++	1.80402	127.0227
GO:1904959	Biological process	regulation of cytochrome-c oxidase activity	5589	4	861	0	0.616210413	0.512021845	5.12E-01	-	-0.29071	0	11	1	0.007872607	0.007851497	1.57E-02	++	1.80402	127.0227
GO:1905038	Biological process	regulation of membrane lipid metabolic process	5589	7	861	1	1.078368223	0.690182395	6.90E-01	-	-0.16104	0.9273	11	1	0.013777062	0.013703296	2.74E-02	++	1.56214	72.5844
GO:2000303	Biological process	regulation of ceramide biosynthetic process	5589	7	861	1	1.078368223	0.690182395	6.90E-01	-	-0.16104	0.9273	11	1	0.013777062	0.013703296	2.74E-02	++	1.56214	72.5844

NOTE

**M**	**The total number of expressed proteins**
**N**	**The total number of differentially expressed proteins**
**UP_m**	**Total number of over-representation proteins**
**UP_n**	**Number of this term of over-representation proteins**
**UP_expect**	**expected over-representation protein numbers according to backgrour**
**UP_q_value**	**Adjusted Pvalue**
**O/U**	**Over/under-representation grade**
**UP_Log(Q)**	**The over- or under- representation strengths are represented by -log(C**
**DOWN_m**	**Total number of under-representation proteins**
**DOWN_n**	**Number of this term of under-representation proteins**
**DOWN_expect**	**expected under-representation protein numbers according to backgrou**
**DOWN_q_value**	**Adjusted Pvalue**
**O/U**	**Over/under-representation grade**
**DOWN_Log(Q)**	**The over- or under- representation strengths are represented by -log(C**

Supplementary Table S6. ESTIMATE scores in iCCA ATTs samples

Sample	StromalScore	ImmuneScore	ESTIMATEScore
P105	-234.22363	537.9875928	303.7639632
P108	-16.4022667	828.601249	812.1989823
P118	-475.126952	408.9758786	-66.15107319
P121	-180.601485	888.9255758	708.3240909
P123	595.1211423	1898.004874	2493.126016
P126	553.3581722	1804.526828	2357.885
P131	20.07682347	875.0177877	895.0946112
P134	17.17248775	930.0600137	947.2325014
P136	507.8720185	1667.411525	2175.283543
P147	-359.57006	940.2972235	580.727164
P153	165.4914729	1213.990338	1379.48181
P154	-217.976252	430.1856906	212.2094389
P156	-34.7116833	746.6560543	711.944371
P158	277.685438	1174.756006	1452.441444
P159	-759.027175	-18.71565531	-777.7428299
P164	298.3129034	846.8465434	1145.159447
P166	138.6052362	1224.339604	1362.94484
P167	4.896304341	874.5671016	879.4634059
P173	336.0640623	1365.285107	1701.349169
P179	218.6538511	1363.31497	1581.968821
P181	96.17141897	902.0392418	998.2106608
P187	505.7830709	1296.453703	1802.236774
P19.136	621.9468545	2524.815072	3146.761926
P19.140	-354.204759	764.274132	410.0693731
P19.177	-500.589879	790.86508	290.2752014
P19.642	-252.730884	1578.223324	1325.49244
P19.705	-364.073759	593.1048135	229.0310543
P19.85	-170.279792	806.0208186	635.7410263
P198	293.8721718	1071.202429	1365.074601
P214	404.9578203	1516.433974	1921.391794
P219	159.8539623	931.9273033	1091.781266
P68	-514.70811	631.0808293	116.3727191
P86	43.42211747	931.5330168	974.9551343
P90	366.4756448	1089.557225	1456.03287
P93	225.1989489	1534.388224	1759.587173
P98	-19.3385469	745.5217497	726.1832028
P101	267.1318223	1307.579038	1574.71086
P119	-104.31249	639.0453363	534.7328468
P129	277.9848055	1280.984101	1558.968906
P182	182.0087582	1016.992772	1199.00153
P184	161.6355517	989.670307	1151.305859
P190	54.04481222	727.7925883	781.8374005
P19.217	-604.74788	-138.2052157	-742.9530958
P19.511	-829.034212	-169.7357568	-998.7699689
P195	-249.704233	565.6702752	315.9660423
P19.608	-223.606986	558.3638177	334.756832
P19.649	-378.235592	493.1236085	114.8880165
P19.689	-773.789112	57.92730086	-715.8618112
P19.873	-69.0003583	1026.405522	957.4051634
P19.893	14.70304351	614.4913485	629.194392
P200	-282.197692	328.1339048	45.93621304
P209	-38.8834714	800.6212549	761.7377835
P224	112.8040882	723.4969312	836.3010194
P225	107.5776186	865.0847293	972.6623479
P232	84.83855321	767.7230119	852.5615651
P234	204.687633	1296.449044	1501.136677
P235	252.3209319	1108.854962	1361.175894
P3	402.4694257	1630.328825	2032.798251
P4	99.03798465	1440.106854	1539.144839
P19.325	-226.933413	999.1743211	772.240908
P19.607	-671.909783	439.5359739	-232.373809
P19.793	40.33564331	1279.651485	1319.987128
P20.31	34.56807559	1013.146148	1047.714223
P20.42	-137.932362	594.1415618	456.2092001
P20.56	-407.720351	560.3325212	152.6121707
P216	374.0756406	1347.265912	1721.341553
P220	-38.7318743	528.498797	489.7669228
P226	39.66665034	831.9444396	871.6110899
P130	-26.5054483	970.222463	943.7170147
P140	-649.596672	144.7020645	-504.8946075
P148	-424.599751	316.7702694	-107.8294815
P162	-85.4014679	861.3320598	775.9305919
P176	-7.72899416	822.4520311	814.7230369
P19.144	-382.107736	543.1537349	161.0459986
P191	-498.45228	554.6679177	56.21563802
P192	-176.399077	355.064009	178.6649318
P19.98	-394.20877	501.2008219	106.9920518
P2	-244.051427	279.9313691	35.87994181
P51	112.4723484	1105.62794	1218.100288
P52	500.7447948	1912.926015	2413.67081
P57	-617.090061	-93.23127464	-710.3213356
P71	-37.0506118	1165.977213	1128.926601
P89	-177.063302	954.9672378	777.9039358
P99	-472.625653	318.0713408	-154.5543127
P19.206	-502.661239	425.4945495	-77.16668904
P19.220	-655.815353	21.41411105	-634.4012416
P193	131.236624	1282.801269	1414.037893
P19.413	-222.803153	160.7727502	-62.0304024
P19.557	-191.305045	629.3156964	438.0106514

P19.603	-294.638077	689.9567263	395.3186497
P196	-137.435849	888.0593818	750.6235325
P197	223.3521181	1439.082942	1662.43506
P203	288.4545206	1261.650304	1550.104825
P211	343.4264553	554.9151908	898.3416461
P222	214.8573637	1003.200868	1218.058231
P231	217.1251868	1250.642518	1467.767705
P102	-299.91319	239.9909417	-59.92224787
P103	121.6172837	976.1708078	1097.788092
P104	182.5967804	1310.134032	1492.730813
P106	-101.248028	820.0372333	718.7892051
P110	-296.159091	1028.738672	732.5795809
P117	-441.350229	204.5478819	-236.8023473
P12	-111.090447	1057.824501	946.7340539
P150	-682.754321	-14.7852829	-697.5396039
P152	-898.005032	-105.688649	-1003.693681
P177	-696.214966	233.6092405	-462.6057256
P19.17	-165.31344	867.5028984	702.1894588
P19.3	-339.686824	698.8747733	359.1879498
P19.62	-380.129631	1171.552363	791.4227317
P19.699	218.5779073	1230.851398	1449.429305
P19.752	-964.491921	-132.025831	-1096.517752
P45	-177.931492	732.6266599	554.6951679
P46	235.4516932	1199.818797	1435.27049
P55	-436.410948	933.4130531	497.0021048
P95	-362.955035	581.9130139	218.9579793
P97	295.7995364	1540.687143	1836.48668

NOTE

**StromalScore**	**A computational metric reflecting stromal cell abundance within tumor tissue, derived from gene expression signatures of extracellular matrix and fibroblast-related genes.**
**ImmuneScore**	**A quantitative measure of immune cell infiltration in tumors, calculated using expression profiles of immune-specific genes.**
**ESTIMATEScore**	**A combined score integrating StromalScore and ImmuneScore to infer overall tumor purity, with higher values indicating lower tumor purity.**

Supplementary Table S7. xCell scores in iCCA ATTs samples

sample	aDC	Adipocytes	Astrocytes	B-cells	Basophils	CD4+ memory T-cells	CD4+ naive T-cells	CD4+ T-cells	CD4+ Tcm	CD4+ Tem	CD8+ naive T-cells	CD8+ T-cells	CD8+ Tcm	CD8+ Tem	cDC	Chondrocytes	Class-switched memory B-cells	CLP	CMP	DC	Endothelial cells	Eosinophils	Epithelial cells	Erythrocytes
P105	0.002031765	0.08454964	0	1.17232986213196e-18	0.002964366	6.36207184467533e-19	0	6.75205328303598e-18	1.28404239905167e-18	0.00234115	0.006591905	0.000686705	0.010281638	0.005583349	0.004140199	4.90867823181148e-18	1.03006475469564e-18	0.012193979	9.45240524295636e-20	2.15769238230729e-20	0.022136548	1.50795298949176e-19	0.044418938	0
P108	0.014351836	0.062061677	0	0	2.7527384925733e-18	1.66888676380049e-18	0.006688176	3.36707730027662e-18	0	0.001949371	2.39259195751026e-21	0	0.01843522	8.10916128891283e-19	0.045821839	3.30057005539851e-18	9.5806293560443e-19	0.004108958	1.38863983667982e-19	0.000404387	0.032551625	2.76191159087378e-19	0.004973321	0
P118	0	0.044061729	1.18019916250159e-17	0	0.032636474	5.96652934670723e-19	0	3.89859834733393e-18	0.002669108	5.11224407253932e-19	0.000213934	0	1.07671680533722e-18	7.83240673699475e-18	0	0	0	0.018406443	0	8.495891050025e-19	0.014029624	1.07137733587181e-19	0.046075773	0
P121	0.046120705	0.05029192	0	0	0.008150181	1.52184432451026e-18	0.009697485	0	0.012930425	0.006515976	0.014064175	0.020600448	0.007803686	0.012690016	0.016054885	0	0.00114636	0.003278037	0.005889126	1.69054829320979e-20	0.014040981	0.001768058	0.040377938	0
P123	0.033405532	0.021107329	0	5.113869769075e-19	0	0.005609252	0.034414643	0	0	0.042179906	9.88038928381561e-20	0.015749473	0.035068681	0	0.177480183	0.005693026	0	0.001784137	0	0.005128122	0.053185331	0.005691441	0.082666928	0
P126	0.046830758	0.046019192	1.42077231609611e-18	0	8.07961403097849e-20	8.59265425694372e-20	0.019210401	0	9.94155713104646e-19	0.048042751	1.88553208804011e-19	0.024833836	0.058027601	0.011843844	0.117006437	2.54334169069297e-18	2.73066882335131e-18	0	0	0.005760632	0.101990802	0.000253023	2.77143337057637e-19	1.59848786772263e-20
P131	0.006134874	0.067215815	6.06616393695198e-18	0	0.000129647	1.27003071875222e-19	0	2.22490593072889e-18	0	0	0	2.13769766648401e-19	0	0	0.069243087	0	0	0.005534489	7.85981346175028e-19	4.46530553220637e-20	0.034316328	0	3.52966976631044e-19	4.53704946342653e-20
P134	0.017971411	0.047849375	0	3.94387436795634e-19	0.023086614	1.9058386355031e-19	3.8893827991428e-20	9.97197639131882e-19	3.16221219176206e-20	4.34068366411522e-05	0.003757466	0	0	0.000163779	1.90278322520063e-19	4.18434088141666e-19	0	0.015082879	0.008882195	0	0.028624282	0	0.021075435	0
P136	0.036577687	0.063758408	2.26333637998804e-18	2.56567777822004e-18	2.19685858583934e-18	1.76394122768904e-22	0	1.32066534540907e-18	3.5786200114375e-18	0.031987049	0.00124367	0.018746848	0.044557259	0.012719953	0.06476322	4.13017121511632e-18	0	0.017214452	0	0.012247464	0.027690567	3.68074881597091e-19	0.017333598	0
P147	0.039160896	0	0.015454771	0.015010619	2.41115969236274e-18	0.011921773	0	0	0.001925051	0.011625958	0.000216224	0.002413301	0.009410411	0	0	0	0	0.049541761	6.92122832300501e-19	2.4802324638412e-20	6.66728674893474e-19	0	0.341567509	0
P153	4.37248458587256e-18	0.081351159	0	4.17511373123665e-19	0.003834082	1.17662262067006e-19	2.68558684314244e-20	7.21275123956597e-18	6.42320681607141e-18	0	0	0	0	0	0.069196488	3.22913102440756e-18	0	1.61236307638435e-20	7.20329696096112e-05	0.000680985	0.052002085	0.003422093	0	0
P154	4.6061489673542e-18	0.081235261	0	0	0.018969392	0	2.05596816987866e-18	0	0	0	2.76632333750261e-21	0	0.001197972	1.40370850438554e-18	0.044154874	1.27243294059685e-17	0	0.000457361	0.000664989	0	0.024099591	1.15559218002542e-18	0	0
P156	9.3910066389621e-19	0.065396323	0	4.61300178785496e-18	0	3.59923761685232e-19	1.1413306715828e-18	9.3967890199878e-19	4.10001130093434e-19	0	2.41494325871597e-19	4.71089854672175e-19	0	1.45124088862747e-18	0.079505717	5.2880499899148e-18	3.42411615124135e-18	0.004861609	0	0.002822293	0.035042826	3.57448439605816e-21	0.008008655	0
P158	0.00925936	0.079211429	2.67022671152884e-18	0	0	0	0	0	3.40588826106389e-18	1.80305669455533e-18	0	0	0.002181238	0	0.109504606	0	0	0.00140601	0	0.006423103	0.0395316	0	0.006592486	0
P159	5.98059667232692e-18	0.056092556	0	3.49976965596225e-18	0.033643523	0	3.46278374818157e-18	0	0	9.34445418772118e-18	0.000710885	5.1740457803779e-18	3.19234532225835e-18	0	1.016363055861e-18	0	8.06256123951683e-05	0.017237526	4.76842461435534e-19	0	0	0	0.021204389	0
P164	0.031152855	0.046958227	0	0	3.06830980147681e-18	3.99434925198e-18	0	9.35109554742739e-19	0	0.033796248	0.008232533	0.014842176	0.015293576	0	0.026058855	0.022479576	0	0.00798443	0	0	0.064399719	0	0.086870841	0
P166	0.043411752	0.0659128	0	0	0.047138377	0	0.01070557	0	1.99867259476936e-19	0.023490489	0.006376219	0.03098494	0.054218271	0.01195761	0.009221143	0.005583176	9.72473415101388e-19	0.009969262	3.23225024144311e-19	2.55008183059134e-19	0.021997976	3.37806420702318e-19	0.074342851	0
P167	2.94259049719588e-18	0.077000215	7.47865611681086e-18	3.59530394357098e-19	9.1517597658967e-18	3.57894337690839e-18	0	0	0	0	0	6.24334458123633e-19	3.48447080348661e-19	0	0.086083703	3.83673492211402e-18	1.52950134128629e-18	0.000953753	0.003100683	0.002809947	0.036878655	0.000289881	5.73127366527206e-19	0
P173	0.028269705	0.080707109	0	0	3.76452104028521e-18	2.0185241495353e-18	0.006868732	2.46700766156703e-18	0	0	2.47938261087096e-21	0	0.004251974	3.72104400097731e-19	0.108629931	3.84883359186134e-18	0	1.42707801692417e-18	0.000703777	0.007477854	0.072133608	0.005070059	3.15894378747456e-19	1.09653929662404e-20
P179	0.026618098	0.046563811	1.25638524015449e-18	0	3.11629950734459e-18	0	0.012602676	2.66736326845301e-18	1.1839746585228e-19	0	0	0.00475214	0.032336957	0.002697922	0.105208928	6.92645151955833e-19	2.81586632002801e-18	0	4.73345233604429e-20	0.005773695	0.051795724	7.22911593014332e-19	0.000217643	0
P181	1.09532404444333e-18	0.056796847	8.95197129680758e-18	0	8.14815897612563e-19	1.38120537816027e-18	2.3965921557933e-18	5.17773613764241e-18	0	4.89037824234192e-18	1.12546288500975e-20	0	1.37270534546135e-19	4.22362287152314e-19	0.172200218	0	2.4340520637829e-18	0	4.55597597560187e-21	0.004259303	0.038138044	0	0	0
P187	0.016415181	0.045944292	1.40181469652075e-18	0	0	7.70819166373597e-19	0.00878599	1.60682967319917e-18	4.55073359210276e-18	0.006173597	1.35725343328715e-19	0	0.013478128	0.000124582	0.103598583	0	0	1.00303426620771e-18	0.004124138	0.003586827	0.082364228	1.00568494676952e-19	1.94829374970976e-18	8.88321879350498e-21
P19.136	0.125826445	0.050665315	1.55503843956977e-17	0.014096041	0.04196215	0.008969574	0.012851662	0	0	0.079246645	0.011134196	0.224519124	0.264916343	0.099945981	0.098793313	0	1.64587080856529e-18	0.014640081	0	0.010915933	0.01274958	0	9.32440499409294e-05	0
P19.140	0.010815298	0.064412397	0	0	0.040829173	0	0.003558621	8.22719706025135e-18	0.024209424	0.00874957	0.003667414	2.21743380649329e-23	0.005066951	0.004501863	0.003042719	1.57437016331915e-18	0.001954688	1.78670778912436e-20	0.002231351	4.77086534003599e-19	0.003655091	8.31706627446436e-19	0.026760784	0
P19.177	0	0.079022691	0	0	0.097160661	0	0	0	0.002384006	0	0.012039294	2.64559588652997e-21	0.012333073	0.01013703	3.00274958105501e-20	1.20877210982525e-18	0.009225623	0.005021582	0.002985711	8.68099929149282e-19	0.005686133	1.03730342236188e-18	0.024747627	0
P19.642	0.03709528	0.062532627	0	0.24170922	1.69365215669773e-18	0	0.018237318	1.46573382450429e-18	0	0.022121811	0.018301641	0.085949534	0.09415832	0.01414756	0.062400615	0	0.021066727	0.016521896	3.97667382551674e-20	0.001846685	0.007951301	0	0.022457678	0.000122128
P19.705	0.013346477	0.069063888	0	0	6.76252654651808e-18	2.49102666252959e-19	2.2035127935524e-18	0	0	0.026834181	0.008354726	0.012042294	0.023786864	2.04568007841553e-18	0.059170321	0	0	0.020580383	7.83224346027251e-18	0.004803826	0	1.03077077641701e-19	0.073729922	0
P19.85	0.042582006	0.10557257	0	0	0	0	0.001057081	4.4163620959885e-18	0	0.002948688	0.006364511	0.002822578	0.008595598	0.004043209	0.035015517	0	0	0.005829809	2.15315618291191e-19	0.003062202	0.004559697	4.28553630356552e-19	0.002914031	0
P198	0.037828114	0.08050822	2.34854308032181e-17	0	0	5.76298278887411e-21	0	5.99644378604934e-18	0	0	0	0	0.029719474	1.20296740129453e-19	0.082532973	0	3.70318250863982e-18	0.002922099	4.21901671120475e-20	0.00178781	0.041901616	0	0.004190518	0
P214	0	0.063076757	2.58518088417246e-19	0	4.80268803138953e-18	3.10653205280876e-19	0.017691982	5.88115299083694e-18	1.18479601042711e-18	2.63232871663321e-19	0	0.002222055	0.019252188	1.44817243884159e-20	0.204810415	1.62824941692361e-18	1.0799313936184e-18	0	0.015683784	0.009198365	0.07055166	0.003823821	0	1.49270758513656e-20
P219	0.008412359	0.073246735	0	0	0	3.27955376366037e-18	0	1.32927458910746e-17	0	5.25731979690352e-18	0	2.64330944261879e-18	0	8.58398316653052e-18	0.048424321	3.49297705253503e-18	0	5.02687136541593e-20	1.18569053162662e-18	0	0.056574158	0	4.77694125048701e-20	0
P68	0.042522828	0.069679778	0	0	0.017864711	0	2.99344895613258e-19	2.46050794399107e-18	0.000496104	1.38095330329575e-18	0.00350518	0.005497572	0.008517822	0.00046325	0.016128502	4.09073320407715e-18	0	0.010898603	6.47970150249463e-19	0.002105169	3.6027553515583e-21	0	0.009926852	0
P86	0.060654635	0.030132459	0	0	0	0	0	9.79076478419387e-18	0	4.74487392515278e-19	5.69720467731837e-20	3.39796727902146e-18	1.28337361582087e-18	0	0.07805884	0	1.97356823455052e-17	0.012600673	8.28502289184107e-18	0.003924096	0.023479698	0	0.040628411	0
P90	0.049456014	0.078600619	3.510052508698e-18	0	0	1.89766414298348e-18	0	2.55526695565877e-17	0	4.59526464667198e-18	0.001630652	3.82469693836877e-19	0.013826266	0.00440023	0.049538002	0	2.67630089608425e-17	0.014963153	3.48162636988845e-21	0.001949421	0.024226073	8.3957945964953e-19	0.017605331	0
P93	2.06119476875941e-19	0.049023817	1.96934485394004e-17	0	0	0	0.007023211	9.96977264582339e-19	1.12985610195434e-18	0	0.002801317	0.019090556	0.049066292	0.012240538	0.117463499	0	1.77347202658669e-18	0.003058872	5.54556950106552e-19	0.005509619	0.040641676	0	1.61749523870921e-20	0
P98	0	0.088105231	0	0	0	1.3733108036752e-18	0.001500456	1.85360114801976e-18	1.40899264145818e-18	5.48695610844463e-18	0.001116057	1.19850261595322e-19	0.007865854	0.001560872	0.072575935	0	0	0	0	0	0.025927538	1.14335825143477e-18	3.45176824278439e-20	7.41509022061598e-20
P101	3.88027532130699e-19	0.070507672	1.13372438384298e-17	0	1.67310269922498e-17	0	0.001598635	5.08241752172746e-18	0	0.005070343	0	0	0.01717171	0.000226006	0.104742149	4.14343875764887e-18	2.78015085498506e-18	0.001920321	4.37493665250971e-19	0.005354464	0.031566755	0.002524423	0.005413021	0
P119	0	0.056605863	4.98007099718099e-18	0	0	0	0	1.05978128717967e-18	0	8.57827051907953e-18	0	7.4638284977635e-18	1.14314305617609e-19	0	0.070061065	0	0	0	0.003523938	2.02574661227861e-21	0.034446813	1.27964107383397e-19	0	0
P129	8.95549725293124e-18	0.062308747	0	0	0.001768823	0	8.97911431502927e-22	4.05959384928511e-18	0	2.79395151624241e-18	4.52011241085198e-20	0	0.013685934	0.000846029	0.264671454	0	0	0	1.04089085831022e-18	0.024193909	0.065874295	0.002908693	7.65245875404287e-19	8.92881424319641e-21
P182	0.036164012	0.063353912	3.97025399339096e-18	8.75921620757043e-19	2.67936935736233e-18	0	0	0	0	1.22863665316758e-18	0.000186975	6.48008136067479e-18	0.002457912	0	0.071172548	5.67966305279618e-18	0	0	4.85027078317044e-19	0.003876624	0.059300417	0	0	2.52281968314887e-21
P184	0.005575046	0.042742963	0	0	0	4.33476558249056e-18	0	0	0	2.52175803232866e-18	1.82987118051742e-19	0	3.66656872713178e-18	1.12604899378889e-18	0.058498274	0	1.93088153287042e-17	0	0	0	0.028057472	0	2.39368238906096e-21	7.09377627031652e-23
P190	0	0.054535985	4.93878884820959e-18	0	4.88609561878448e-20	0	0	1.22777309382307e-17	0	6.92315113508527e-18	3.43358288645856e-20	8.64696308621386e-19	1.91189171097149e-18	0	0.040831457	2.31279254181342e-18	0	0.001258186	0	0	0.056301737	0.002234025	0	1.37812658664611e-19
P19.217	4.026223956792e-18	0.054077332	8.06316890475459e-18	0	0	1.07033384868577e-18	0	1.9899984622077e-18	0	0	0.00035583	3.71897868112217e-18	0	0	0.012472259	0	0	0.011822078	0	0	0	0	0.022430227	0
P19.511	3.25198468242146e-18	0.059699914	3.31722054691736e-18	0	0.007081141	0	5.13124825033371e-18	2.42822342924884e-18	0	2.9407016064294e-18	0.011970781	0	0	8.58486823913316e-18	1.52038150271799e-19	4.13841479299602e-18	0.002936175	0.011295945	0	0	0	0	0.023136203	0
P195	0.006212286	0.073600074	0	6.41354204979798e-19	0.039732355	0	5.60373367670612e-19	1.01578824207694e-18	0.005115896	0.002576124	0.003686477	4.96459052446473e-19	0.008305227	3.49772593364372e-18	0.028586384	1.97699528319192e-18	0	0	0	0	6.11517856793546e-20	0	0.009142521	0
P19.608	0.007836177	0.060098125	5.45455272970815e-18	1.41549308480025e-18	0.000884145	1.28218518346976e-19	3.06450829951386e-18	0	1.0573151224835e-19	0.003014109	0.013541619	0	0.004337638	2.16238783832957e-19	0.01627803	1.93870832918216e-18	0	0.019009519	0	8.04839986800132e-21	0.03087658	8.25761722422216e-21	0.022841229	0.00071034
P19.649	0.005279257	0.081491089	4.24620399885832e-18	0	0.003477786	1.48696922155629e-19	0	1.67630951796703e-18	0.001000464	0.000739042	0.004477112	0.002357466	0.011640288	0.003653146	0.024083602	0	0	0.003190376	0	0	0.007251328	0.001344286	0.002809192	0
P19.689	3.90759040542607e-18	0.05073957	7.27540703345854e-18	1.27300255118365e-18	0.023381573	0	0	5.92285163273108e-18	3.32065194876544e-19	2.28452909768615e-18	0.016164474	1.46275287948451e-18	2.35772504418562e-18	6.69228183828033e-18	0.002681314	1.75639277075778e-18	0	0.02254685	1.53135504585274e-19	1.01154662250833e-19	0	0	0.00852019	0.000308879
P19.873	1.11211426292943e-20	0.059065582	1.33433338780501e-17	0	0.033219353	0	0	6.86151194609113e-18	4.60735284724916e-18	3.91673527012475e-19	0.000998113	3.44842446086914e-19	0.014568808	0	0.083720848	0	2.59707146715062e-18	0.000776134	0	0.003469296	0.026791593	0	8.40635013374419e-20	1.90137667154469e-20
P19.893	0.010362253	0.087874787	6.61936646572077e-18	0	0	8.80919055217182e-19	3.03091249286785e-18	2.27288428994965e-18	0	3.042663572238e-18	0	5.06511176401929e-19	0	2.21127248269315e-18	0.077440075	0	8.65071921871415e-18	4.13774772254339e-21	0	0.000923878	0.025172819	7.46937302160059e-19	0.00178305	4.45501780070505e-22
P200	0	0.049721066	6.29810272490059e-18	0	0	0	0	0	1.60919551109877e-18	0	0	4.23998507507413e-19	0	0	0.020913641	0	6.48071224687846e-18	0.002204606	1.35495277568623e-21	2.16544318823409e-19	0.021995137	1.15054484392818e-18	0	6.37790560702347e-21
P209	0.007965205	0.068819007	8.81132119381958e-20	7.28284774830199e-19	7.21944761167392e-18	0	0	2.92079634000708e-18	4.40272769145593e-18	1.08651400170538e-17	7.26132276916187e-24	0	0.003968322	1.6949900970971e-20	0.088045186	4.22160831691446e-18	0	5.41262564111436e-20	0.005690192	0.003283701	0.034596515	0	9.04383404615385e-19	0
P224	0.001715299	0.0528382	0	0	0	5.03134480614578e-20	5.19623873758062e-18	8.18362495950306e-18	0	0	1.63577018666674e-19	9.95041012520956e-18	2.38330682780574e-18	6.17242499165673e-18	0.023311463	1.96720154024963e-17	0	0.000462865	1.40052951642577e-20	3.24567162830825e-20	0.032169373	0	0	9.24225314642436e-21
P225	1.08604350529409e-18	0.043004601	1.38578295588289e-18	0	0	0	0	1.78041099986195e-18	3.71818252357045e-18	0	1.37623479626685e-20	0	3.80628371719121e-19	0	0.097252482	0	0	0	0	0	0.034318332	2.32663376517724e-18	0	3.01769604319954e-21
P232	0.005903486	0.049058895	0	0	3.12853481547034e-19	0	2.58090333360834e-18	5.84643964069452e-18	0	1.55977220325235e-19	0	2.95236624927485e-18	1.59207659784915e-19	2.13620079571419e-18	0.097960075	0	0	1.11591820169929e-19	2.99086056109651e-19	0.001408087	0.048278027	0	1.00822480127555e-18	1.27738388157555e-20
P234	0.0212327	0.04837904	7.08099531696882e-18	0	1.31774767786126e-18	0	0.002936331	0	0	0	0.000448421	0.011687209	0.017843309	0	0.109124922	9.620025190501e-18	3.0201819322983e-19	0.003590921	3.14036314637503e-19	0.008228594	0.02980911	3.50912609515743e-19	0	7.38478361237749e-21
P235	0	0.074406398	8.67914000444143e-18	0	0	0	0.001340861	2.53380051889645e-18	0	9.4696745559534e-20	5.42265729636355e-19	2.32540582158957e-18	0.004283146	7.04443404054617e-19	0.142417866	4.58003482361827e-18	0	8.6244988646517e-05	1.20641962057336e-19	0.005117085	0.032469373	0	2.36270812965805e-20	0
P3	0.06848419	0.042317747	0	0	0.013269526	5.74994286832375e-19	0.002475853	0	0	0.005670381	2.31305030491514e-20	0.006837181	0.027817761	0.000731669	0.109948938	1.16251835480588e-19	1.11336078741337e-17	0.009325753	3.16238876044621e-18	0.007026389	0.056017066	0.003518193	0.004596486	2.88700588961658e-21
P4	0.002070669	0.072132935	8.9898087911868e-18	1.244929129457e-18	0.003278974	0	1.32419164926841e-18	1.19634614129457e-18	0	0	0	6.96345324096682e-19	0	3.4607487850567e-19	0.067189864	6.92643258497219e-18	2.54286904448136e-18	0.000829368	3.36352759379096e-19	0.000987761	0.027706833	8.36571932375985e-21	5.7278041893894e-05	0
P19.325	0.015135137	0.080179184	0	0.02647442	0.007197329	0	0.012371312	0	3.09976204789669e-21	0.038453549	0.006594571	0.048801518	0.054208172	0.035459081	0.012235156	0	0.007838726	0.006718861	0	1.30144318217905e-20	0.006818908	2.16858768359853e-19	0.011003172	0
P19.607	0.008542831	0.054297738	0	0.004798455	0.021219029	6.9841075788013e-20	0	2.04763529207119e-18	0	0.007265234	0.009032774	2.60525855650841e-20	0.006651723	3.00997783162957e-18	0.020194362	0	0.004716343	0.006977656	0	0.002295378	8.6727040598063e-22	5.521807651535e-19	0.0357833	1.13123957356755e-19
P19.793	0.014269101	0.063393482	0	0	0.010702595	2.1112374107131e-19	0	0	5.09470655112076e-19	0.000606467	0	0.016358201	0.034082364	0.003004717	0.090700093	0	0	0.004256698	0	0.005944026	0.024294548	0.002697517	8.83981926894735e-21	0
P20.31	1.38245636260652e-17	0.065123998	7.31387102276652e-19	0	0	1.28058834530734e-18	0	0	2.07081631796661e-18	0	0	2.27512458220859e-18	0.011931093	0.002394361	0.113202404	0	0	0.00132125	0	0.002290111	0.043419375	7.56778815481325e-21	6.89371656438026e-22	2.44505726788607e-19
P20.42	0.001660427	0.101787441	2.09257003942418e-18	2.62750735447912e-18	0.007738572	4.56886823751284e-20	4.59161818142602e-23	0	4.34201877788108e-18	7.09579867944333e-19	0.000566352	3.64750464683387e-19	0.016363388	0	0.024647793	0	8.80940361878338e-19	4.13975718992351e-22	0	0	0.036221028	0	0.003896116	4.98675602223098e-20
P20.56	0.012736038	0.03873991	0	0	0	1.02373928481433e-18	0	1.83120952734989e-18	0	0.013946055	0.0113201	0.000688791	0	1.33874003863222e-19	0.004197759	6.17345332552899e-20	0.003275959	0.020425078	4.72355024747898e-19	1.09082825597315e-20	0	0.002276565	0.051613701	0
P216	0.005085991	0.057848202	1.79203567610155e-18	1.29286425969662e-18	4.08419427344643e-18	1.94324852345244e-18	0.009548346	3.30201747553418e-18	8.82543423288241e-19	0.001234109	3.19375995287504e-19	3.09622847463225e-19	0.0123947	2.45904101041287e-18	0.106385871	6.10342202427323e-18	0	0.000636792	0.005027154	0.007271283	0.09188898	0.006620284	2.57166630140265e-19	0
P220	4.52729749491881e-18	0.089463027	0	0	1.37759506577544e-18	0	0	4.52614084696345e-18	0	5.6136536662064e-18	0.003200466	2.75958256274458e-18	0.00045658	0	0.019401698	0	0	0.003339869	0	1.00758258634702e-18	0.047890972	4.33731251453718e-19	0.015774067	0
P226	5.03261721930521e-21	0.067711999	1.71883539342792e-18	1.766357980582e-18	1.20112184253143e-17	0	1.14862089544333e-20	2.87837695976487e-18	3.51567968010924e-18	2.99355348964757e-18	6.55608228834478e-19	2.27662260133852e-18	0.009751533	1.8172816446512e-18	0.073733975	3.23882417277696e-18	3.64159474700918e-18	3.22781019582616e-19	3.94785470178369e-21	0.001193646	0.031761216	4.33645514902203e-18	0.005142528	0
P130	0.011983228	0.069492159	3.72400667476407e-18	0	0	5.08117220603051e-20	0	2.59709931985001e-18	1.66845877934911e-18	0	0.004319086	0	0.010353667	0.004420932	0.081264483	8.31062535737777e-19	2.12935099129507e-18	0.005996003	0.000117188	0.005772008	0.021274516	5.60778668077355e-19	0.008356937	0
P140	0	0.084737448	0	0	0.065101992	0	7.72374725465749e-19	8.5297769431033e-18	0	3.69280857716377e-18	0.009208097	0	5.14233476936191e-18	6.72399010411025e-18	0.011295651	2.5685777584606e-17	0.002080609	0.000183948	0	0	0.001828641	0	0.041842149	0
P148	0.000362575	0.081707757	2.82770031023715e-17	6.58451840714605e-20	0.051749429	1.21396594704578e-18	0	0	3.00841683888162e-21	0	5.07536713805454e-21	0	4.97386208992831e-18	2.2954600267151e-17	0.014492824	0	0	0.017245958	1.13066059849292e-18	7.98822216661667e-19	0.002432444	1.0601639548254e-18	0.005750714	0
P162	4.91964947327154e-19	0.073173386	0	9.27079317147078e-19	2.22769669013611e-19	0	0.000753197	3.35129585931289e-18	2.87812570907606e-19	0	0.001541935	0.001674076	0.019936511	0.003880438	0.050066659	0	0	0.000340019	0.007364127	0.001581219	0.017744265	0	0.000809324	0
P176	5.26865493751573e-21	0.066689786	2.12683985558393e-17	0	8.80769240977742e-19	3.13272364382224e-18	4.79766361691655e-18	2.17354121476621e-18	0	3.44635197092382e-18	0.002639459	0	0.008161257	4.0016545276208e-19	0.063749725	8.11577096142713e-18	0	0.00406149	1.24304085423492e-20	0.000970775	0.023899404	0	3.27327461111658e-19	1.601881380682e-21
P19.144	0	0.073714413	0	2.15417383258719e-18	0.03607789	5.62928514052339e-19	2.19742184475e-18	3.63094128056223e-18	0	0	0.015229358	0	5.73977650407733e-19	0.001909323	0.002171157	0	6.09864728770048e-19	0.009512667	0.000887252	1.29669362233276e-18	0.005544357	2.10480500659388e-18	0.017686604	8.53299948363406e-21
P191	0.00599674	0.078704411	0	6.44540927176607e-18	0	0	1.47271096042311e-18	0	1.11679727499726e-18	0.002164532	0.009299995	0.006321922	0.012948883	0.003259401	0.047722025	0	0	0.007456256	0	0.005083798	0.002346942	2.65552696863948e-19	0.003630065	5.94301914074352e-22
P192	0.007801927	0.07114479	2.80490299687254e-18	1.40482053302147e-18	4.15637810179229e-19	2.90000899215905e-19	0	0	0	0	0.001459559	0	5.22181497663672e-19	0	0.038295286	6.69893879172075e-18	2.10933420056405e-18	0.005333322	0.002205439	0.000462251	0.010103681	2.11341737033128e-19	0.00342513	0
P19.98	0	0.075324214	6.70103241925685e-18	0	0.004301099	1.64867584613223e-18	0	1.12157467058644e-17	0	0	0.009191525	0	0	0	0.022634028	8.27096697078849e-18	4.79339606303706e-18	0.011448948	1.9225521866338e-21	4.38399646126923e-20	0.006291495	0	0.006109848	3.9690582595046e-21
P2	0.024330557	0.08109138	4.00366347121668e-19	0	1.08099266952018e-17	9.28281458531067e-19	0	1.20672921887539e-18	0	0	0.000636624	2.1126213800803e-18	0	4.99910708417554e-18	0.002432381	0	0	0.011218156	2.48550596468011e-18	3.32930037713329e-19	0.018827789	4.45617453420397e-20	0.012657959	2.83069060791546e-05
P51	0.037498798	0.106682611	1.19980638472352e-17	3.07633398158273e-18	0	0	0.001929047	6.25410634388193e-18	0	0	0.001008676	0	0.007093804	0.007662199	0.072532954	0	9.20772953009637e-18	0.000757365	0	0.003494329	0.028670622	1.27479127046027e-18	2.39018592133636e-05	0.00029899
P52	0.075116937	0.062689598	0	1.52450350170003e-18	0	9.98418307832243e-22	0.003034268	2.51235694296363e-18	0	0.032947657	0	0.046883001	0.049166685	0.029236383	0.187602387	2.60451561855381e-18	0	0.008102558	1.02867544370287e-18	0.030578137	0.043396068	0.007204617	1.17117576254231e-19	0
P57	5.05075010730581e-18	0.07526546	0	7.0248770258757e-18	0.007432832	3.15433329744247e-19	0	6.7972752725784e-18	0	0	0.003679694	0	8.28707294951672e-18	0	0.006228941	0	2.12729672971366e-18	0.003688659	1.67957600252751e-19	1.92982767308536e-19	0.005176218	4.19098679940957e-19	0.000560742	0
P71	2.16171953167395e-18	0.060923976	0	0	0.017043069	0	0	0	2.34255554843376e-18	1.00758374661835e-18	0.003025556	1.7504843301354e-18	0.009192543	0	0.056803201	0	0	4.74559998405627e-20	0	0	0.033185664	4.42962237559757e-20	1.70899530081816e-19	0
P89	0.022797696	0.066291356	0	0	0	0	0	7.36419914014289e-19	2.64350431455644e-18	0.011354665	0.004134622	0.002891814	0.029386385	0.007412425	0.074854438	0	1.93075552549895e-18	0.017377161	0	0.00631718	0.024119334	0	0.009611154	0
P99	0	0.067782913	0	2.28346426195621e-18	8.46202811277262e-19	0	3.49121590380433e-18	2.16710029707421e-18	3.19569884826401e-19	0	0.013317038	3.8426074812789e-19	0.000842265	1.29038958023024e-18	0.012759334	0	0	0.004421822	1.33586376066332e-20	8.35172008452083e-19	0.00440193	3.76672689534688e-19	0.008358665	0
P19.206	5.1098076971372e-18	0.052451623	0	1.50514683657967e-18	0.003234094	0	0	2.27740074499837e-18	0	0.012355929	0.003410873	0.002328932	0.013954139	0.003734339	0.013127893	0	2.70192030094915e-21	0.011697235	2.37126166554207e-19	0	0.001104208	1.28975427924138e-18	0.008043544	7.97805273044417e-20
P19.220	0	0.070973886	8.75298639743705e-19	4.83656789442851e-18	0.003142827	0	3.70685017865845e-19	2.113580065699e-18	0	3.32636793914385e-19	0.004003462	0	3.30622392610087e-18	2.81091048105339e-18	0.003777127	0	0	0.016653691	1.07595490887443e-18	1.32756222325112e-18	0.000649444	0	0.026667696	1.48068507156338e-21
P193	0.03529655	0.049138092	1.9730676085425e-17	0	0	5.04889815975347e-19	0.008500611	0	1.89575066850654e-18	0.005272341	0.00282463	0.013482936	0.033268173	0.000999015	0.075424913	0	2.46179971190493e-19	0	2.28103360900133e-18	0.004452479	0.042227583	0.001555769	6.24776809754395e-20	4.99218208248999e-21
P19.413	0	0.063605853	0	0	2.20979306000175e-18	2.42241249961656e-18	1.05129348498171e-18	5.9065186668116e-19	0	0	0	2.27867204183202e-18	3.60071402700261e-18	0	0.039828883	5.88758257292849e-18	0	0.009047668	1.4589235021683e-18	0	0.02265958	7.8502049088002e-19	0.005656799	1.32616434840474e-20
P19.557	0.013028549	0.087507225	1.29511646752715e-17	1.04688754826772e-18	0.016417454	0	4.92970676424086e-18	2.49183009214145e-18	0	1.44620285799712e-20	0.010739971	1.70419244543227e-18	0.008305404	2.44643106504347e-18	0.029496793	0	0	0.010760698	0	0.001646282	0.004997887	2.12028029883029e-21	0.032894489	0
P19.603	0.016443405	0.054469231	0	9.97976306191133e-19	0.040008158	0	4.42647876257213e-21	4.34538885898281e-18	0	0.009987996	0.005410039	0.002561247	0.018722981	7.86294231487884e-18	0.023955838	0	1.73695163545496e-18	0.005205692	3.84234651750357e-19	2.72653735486322e-19	0.011047293	0	0.015776306	0
P196	0	0.059866632	0	1.86149809845456e-19	0	1.35706768791664e-19	0	9.58904188590044e-18	0	4.76860519368796e-18	0.001271538	2.08867653475237e-18	0.009419892	6.43041087905347e-19	0.113944025	5.31868424936168e-18	0	0.017000945	4.16026233085598e-19	0.009102435	0.012421387	3.78506910433928e-22	0.077735914	5.91118538744418e-21
P197	0.021544744	0.061126679	0	2.17130235723778e-18	0.005162589	1.4490394623907e-20	0.004188017	1.20038335505311e-18	3.36953739984193e-19	0.01508905	2.32761483293884e-19	2.05365708352031e-18	0.004988946	0.00501724	0.087934237	0	8.64664308961706e-19	0	0	0.006010368	0.043997676	0.002229721	0	0
P203	0.001497809	0.06025141	4.77323212784273e-18	0	0	1.92626783904056e-18	0	5.80053672622625e-18	2.75813366973107e-18	0.00695771	0.004514932	0	0.008518555	0.003357778	0.120187348	0	1.89004979552536e-18	0.005974651	3.17992647885923e-19	0.009742881	0.095391563	0.001040927	0.001876863	0.00026124
P211	2.21257893706257e-19	0.062010156	0	0	2.99156921148752e-17	6.39844002700105e-19	1.08013384723759e-17	0	2.27945133447007e-17	1.70695105705231e-18	0	0	0	1.15910709743004e-17	0.059716452	0	0	0	1.37501266403077e-18	1.47157235263857e-17	0.066540317	6.76297327779667e-18	2.16886719808835e-18	4.04846296189022e-21
P222	0.015218586	0.058248728	3.19271194001476e-18	0	0	1.23285694313879e-18	2.69701058013152e-18	1.35676723724764e-18	1.17690585251146e-18	0	0	6.83299175534856e-18	0.00475948	6.8151701514863e-21	0.078891218	0	1.20861656104857e-17	9.06475655664761e-20	0	0.003860715	0.042358056	1.87499113135908e-19	1.4758651063291e-19	1.68061798253352e-19
P231	0.062604529	0.060238899	3.0604452921077e-18	0	0.013986868	2.17570249197614e-20	0.002802269	0	4.68299457387258e-19	0.017738346	7.92708220174623e-21	0.007758334	0.025135716	0	0.099130037	1.65555458524226e-18	2.39948439262574e-19	0.005524709	0	0.010063633	0.040457308	7.24520378330068e-20	2.22883089676139e-19	0
P102	0.004602986	0.069285933	5.89683757203868e-18	0	0	0	2.81315941704992e-18	6.2058296981412e-18	2.10746875129251e-19	0	0.000156092	3.43433173983056e-18	0	0	0.037468698	0	2.20815406014341e-19	0.006846044	0.000502261	0.000592544	0.022492715	0.001459449	0.009211404	0
P103	1.22026498571489e-18	0.081094427	2.54729687111152e-18	7.47306226924788e-19	0	0	0.003448648	2.12062424114114e-18	0.007245581	0.001063152	0.00188812	0	0.001272757	0.00544665	0.0582135	0	1.23799276764456e-18	0	0.005584265	0	0.041229063	0.006885078	1.85486970974299e-19	1.07959425741363e-22
P104	0.009734718	0.067738129	0	1.09080751132312e-19	0.022134904	3.58575162684949e-19	2.55239085341606e-22	2.5894547003825e-18	1.92366618751051e-19	4.3715361151192e-19	6.18793911798864e-20	0.004327536	0.002330856	8.88600048626162e-19	0.089169794	1.45417341446922e-19	0	0	0	0.003231347	0.049742097	0.007762594	0	0
P106	0.00181376	0.089917231	1.27994911862609e-18	4.13024879194642e-19	4.81194585551427e-18	9.25544243015998e-19	8.81805703250169e-19	0	0	8.07570483963474e-19	0.001242506	1.17075015798248e-20	0.00295192	9.32682587620179e-20	0.064223476	4.07440316444406e-18	0	0.001870678	1.41485583645386e-19	0.000765076	0.040088307	0.001694852	0.002549666	1.69512331751353e-21
P110	0.004336719	0.070871319	0	1.01064040207326e-18	0.045689429	0	0.001949816	0	4.82802973975216e-20	0.011366769	0.018210446	0.022144404	0.031084226	0.003798506	0.042070148	0	0	0.01229074	3.03455065140108e-19	0.001060612	0.011024484	0	0.003032273	0
P117	1.29071700759607e-20	0.073175214	0	1.70664745485994e-18	0.01014725	0	0	2.02200744093086e-18	0.00628586	0.006417316	0.008238783	0	0	5.39401993249809e-19	0.016777177	3.67976685825133e-18	0	0.013699657	0.00123582	0	0.009975902	1.33767483285317e-20	0.027596431	2.431412785102e-20
P12	0.025847469	0.053084219	4.31663258507569e-18	0.048014145	0.043544837	0	0.006955935	0	0.024177764	0.028632964	0.007456892	0.014701397	0.018444037	0.001436841	0.041102776	0	0.017387828	0.00470293	0.003914807	0.003051429	0.032923349	0	0.011359962	0
P150	4.09869939043736e-18	0.054879746	0	0	0.036504898	0	0	0	0	8.36802238959552e-19	0.006379148	0	3.74119133289892e-19	0	0	0	0	0.003976666	5.73620472234849e-05	0	0.00793983	0	0.020669721	0
P152	6.62618936436005e-18	0.062118053	0	2.5253957997868e-19	0.054842805	3.83405097581738e-19	0	5.11580086227033e-18	0.025567256	0	0.016695152	0	8.58626598032095e-18	7.86734218742569e-19	0	1.01346240197976e-17	0.01272578	2.17709755526991e-19	0.000842977	0	0.000133254	0	0.01153126	0
P177	0	0.072899508	0	0	0.037141597	0	0	0	0.017336527	0.005943338	0.015607015	0.005084967	0.001505691	0.001177593	2.12377157675601e-19	4.59694410331945e-18	0.009197387	0.00548374	0	7.61629396276367e-19	0	0	0.028836617	3.20877492442397e-20
P19.17	0	0.084128237	0	0	0	1.1470334856438e-18	0.000279136	0	5.98121517467145e-19	0.004799135	0	0	0.008574164	0.000476652	0.053854573	2.20322569779904e-18	0	0	0.000518737	0.001474386	0.03925412	0.002898045	0.000174359	0
P19.3	0	0.065643906	0	0	0.029257668	6.16789131284025e-19	3.98833488589149e-21	0	2.99146888941732e-20	0	0.007689068	0	0.012624671	0	0.026478876	0	0	0.001918476	0.006995841	1.7509022516902e-19	0.01953414	5.24631814068936e-19	0	6.31099221901889e-21
P19.62	0.028601791	0.056692228	7.34907940713761e-18	0.040256946	0.044245956	5.11301095501218e-20	0.021666556	0.011950283	0.062452239	0.042106729	0.012976962	0.078897134	0.039526603	0.018047827	0.005680137	0	0.011263039	0.00323894	0	5.36286665022568e-20	0	2.35621613346837e-20	0.008753239	2.36083237485159e-20
P19.699	3.66801007548698e-18	0.058725492	0	0	0	0	0	0	0	0	0	4.62417554878534e-19	0.019142852	0.00141125	0.167205098	6.62522833990964e-18	0	0.001960358	[illegible]	0.003795088	0.092296353	0.002109898	0.016586067	0
P19.752	2.17990892152486e-18	0.082071932	0	0.02242926	0	0	0	0	0.00104966	0.005082779	0.009978952	0.000356718	4.91500108913363e-19	0	7.95499512457149e-19	4.72657672161991e-20	0.014729955	0.010848206	1.27025364984042e-18	5.43207646052247e-19	[illegible]	0	0.012387706	3.53329847953778e-20
P45	1.55949854003444e-19	0.101000467	0	1.67549710991199e-18	3.2396186496515e-18	7.09883232615546e-19	0	0	0	0.005962253	0.002898322	0.007742504	0.014318438	0.006178541	0.025137183	0	2.10441929645725e-18	0.00549898	5.60008747258625e-19	0	0.009029536	2.05950861019643e-19	0	0
P46	0.018424443	0.051253985	0	0	0.028109179	6.33717676833742e-19	0	0	1.32066132885702e-18	4.93938069522672e-18	0	0	0.004510804	1.46868416998574e-19	0.071286188	1.27850998963572e-18	9.13736910368342e-19	3.51007047356444e-19	0.000631556	2.53240122559149e-20	0.045024926	0.001513457	0.00012545	6.34243012435421e-20
P55	0.053799229	0.057173141	5.63530831790844e-18	0.004294829	0.042678413	0	0.00578766	4.33356004055383e-18	0.058711879	0.014545363	0.00973129	4.79791351243531e-21	3.08201861638831e-19	0.017187493	0.005987042	2.51881584836259e-18	0.020105499	0.000609925	0.00734866	1.46202746996972e-21	0	0.003715343	0.01370438	5.60116784371853e-06
P95	0.021288206	0.065499896	0	2.22458397115113e-19	0.007988516	2.25620373770064e-19	6.99712708080926e-20	0	0	0.033931553	0.007668151	0.014932156	0.013183087	0.01725012	0.000622067	0	0.009833206	0.010761212	0.001736509	3.14360974818251e-20	0.005027153	0	0.039126036	0
P97	0.030750189	0.067644632	1.95466403624317e-18	0	2.8515610597615e-19	1.78656822398179e-18	0.011071878	4.37386186959175e-19	0	0.004936306	0.000329289	0.019436091	0.029688456	0.00519834	0.115714101	0	0	0	0	0.008198778	0.055850124	0.005072672	2.91044177387156e-19	8.87694815145707e-21

NOTE

**aDC**	**Activated dendritic cells**
**CD4+ Tcm**	**CD4+ central memory T-cells**
**CD4+ Tem**	**CD4+ effector memory T-cells**
**CD8+ naive**	**CD8+ naive T-cells**
**CD8+ Tcm**	**CD8+ central memory T-cells**
**CD8+ Tem**	**CD8+ effector memory T-cells**
**cDC**	**Conventional dendritic cells**
**CLP**	**Common lymphoid progenitors**
**CMP**	**Common myeloid progenitors**
**DC**	**Dendritic cells**
**GMP**	**Granulocyte-macrophage progenitors**
**HSC**	**Hematopoietic stem cells**
**iDC**	**Immature dendritic cells**
**ly Endothel**	**Lymphatic endothelial cells**
**MEP**	**Megakaryocyte–erythroid progenitors**
**MPP**	**Multipotent rogenitors**
**MSC**	**Mesenchymal stem cells**
**mv Endoth**	**Microvascular endothelial cells**
**NKT**	**Natural killer T-cells**
**pDC**	**Plasmacytoid dendritic cells**
**Tgd cells**	**Gamma delta T-cells**
**Th1 cells**	**Type 1 T-helper cells**
**Th2 cells**	**Type 2 T-helper cells**
**Tregs**	**Regulatory T-cells**

Fibroblasts	GMP	Hepatocytes	HSC	iDC	Keratinocytes	ly Endothelial cells	Macrophages	Macrophages M1	Macrophages M2	Mast cells	Megakaryocytes	Melanocytes	Memory B-cells	MEP	Mesangial cells	Monocytes	MPP	MSC	mv Endothelial cells	Myocytes	naive B-cells	Neurons	Neutrophils
2.48389094813163e-18	0.008382566	0.187814441	0.187692575	0	0.001337166	0.015185222	0	1.05806656311711e-18	0.002319367	8.61666757641635e-21	0.012466401	0.002700788	1.68805835147403e-18	0.040584044	1.48050710408154e-18	0	0	0.127700299	0.014962576	0.008084809	3.03112637494431e-18	4.69036028388591e-20	4.56997855441141e-21
2.62108597821733e-19	0.02021101	0.189000592	0.319527185	0.039404965	0.000103811	0.012912995	4.41509605449044e-19	0	0.018706102	5.62710044683628e-19	0.010765725	0.002337645	0	0.019882083	0	0	5.00125204321348e-19	0.087404769	0.014476603	0.003682234	0	3.91698577303316e-21	4.22284758186083e-19
3.64918334322341e-18	6.20441443424174e-19	0.18094315	0.114507789	0	0.001288774	0.008208553	4.80802313008483e-20	0	0.007550365	1.70356513371444e-20	0.003821901	0.000624097	4.26012639574664e-18	0.03053167	0.013801591	0	6.82091089266576e-19	0.05498712	0.006576301	0.006462097	6.73826430080408e-20	0.000144358	0.002058223
0.020544755	0.008902795	0.18143301	0.195108952	0.005233294	0.000833413	0.010482427	7.82295810212318e-19	0	0.005220712	1.56032477171107e-18	0.003524961	0.000707951	0	0.014778413	0.006836525	0	0	0.165335456	0.007395803	0.007295915	1.51054954432712e-20	0.00057637	2.42361157408836e-19
0.079142992	0.047164299	0.128526255	0.359843766	0.056671052	0.001456133	0.022299357	0.007252704	5.19363252005816e-20	0.024063832	0	0.012026608	0	0	2.05953706243068e-19	0.056371339	1.18644499777456e-19	7.29090807642293e-19	0.035453101	0.032398771	0.003576846	2.08606964285487e-18	2.52960124265186e-05	0
0.028201586	0.030388384	0.185948994	0.379349518	0.117456928	0	0.047705667	0.007527794	0	0.034982382	0	0.018409051	0.001026325	0	0.000685389	0.005138994	0.01885103	0	0.068403156	0.062284576	0.00097387	0	0	0.004690922
2.41197007565719e-17	0.039730711	0.198128097	0.279410385	0.041684969	0	0.012691001	0.003814851	2.21390132306765e-18	0.025316855	6.14025309973118e-20	0.008703154	0.001067492	6.5552991484073e-19	0.014648446	4.11219049975577e-18	0.005893467	7.68778095719765e-19	0.011172193	0.021877655	4.88601751415943e-20	0	1.25542887031728e-20	0.003886692
0.021699638	0	0.168748796	0.144703212	0	1.28116552609212e-21	0.01555468	0	0	0.010103487	0	0.009294183	0.001266065	3.16178064200682e-18	0.012798226	0.00608347	0.01946309	0	0.154701435	0.023501852	0.005245357	0	0	0.00415489
3.92117097956175e-18	0.03895586	0.178544662	0.284860842	0.110219634	0.000199864	0.005042775	0.032522958	0.005948414	0.064395891	7.49391168447139e-21	0.008856005	0.001308998	0	0.012310747	0.006369215	0.019339371	5.28695058459696e-19	0.038622434	0.013336131	0.001398049	4.92856348852214e-19	5.97348562846421e-20	0.001105769
0	0	1.36084402555915e-18	0	0.045038141	0.058419162	3.80386630932456e-20	0	0	0	0.001386448	3.64990384983801e-06	3.19157929333179e-18	7.06303701693176e-20	0.006517819	0.150677689	6.63465352067166e-19	0	0	8.79718974221334e-19	0	0.001177183	5.19480754626762e-22	8.65472785407231e-05
1.3076019990839e-17	0.044650136	0.196232188	0.246203952	0.073269726	0	0.018967717	0.01267734	7.27224011610617e-19	0.040579039	0.001866387	0.010231524	0.000348501	2.98613442557519e-18	0.010498036	1.11383240498915e-19	0.019889432	1.21268237066102e-17	0.006008688	0.034395305	0	0	2.53046898429919e-20	0.004795655
2.49367034894317e-17	0.008456082	0.208115429	0.27980586	0.027684886	1.4702225783182e-20	0.005494483	0	5.51051159720931e-18	0.010818624	5.94619064384044e-19	0.011139662	0	2.08191909986995e-17	0.024209295	0	1.94287183601181e-18	7.68303487143631e-19	0	0.012131792	0.000472096	1.2660294163084e-18	0	0
0	0.021182165	0.195412122	0.174864403	0.105501073	0.001361008	0.010533204	0.008019145	0	0.027985569	1.4243810456738e-19	0.009176313	0	2.98124812860856e-18	0.015071902	3.3130100515347e-19	0.00755698	3.30178966055288e-18	1.08345878840531e-17	0.0264745	0	0	5.28859640716814e-21	0
0	0.036066431	0.195760843	0.342206762	0.125902981	0.000125858	0.012542526	0.011725923	0.000487796	0.039995315	4.54072980991835e-18	0.007141548	5.44798710141231e-19	0	0.008968293	0.011247199	0	7.57195061607583e-20	9.30282033371425e-18	0.025746606	0	1.71920114729577e-18	6.21075367745287e-20	0.000239334
7.03599427075734e-18	0	0.185286998	0.086601048	0	0	0	1.2565969382044e-19	2.96514357728011e-18	0.00064212	0	0.003697871	0.000477183	0	0.039323773	0.000564513	6.98365238323089e-18	0	0.023448009	5.66487041281108e-19	0.002205673	0	1.27639573737095e-21	0.001402935
0.128727975	0	0.163838935	0.270956633	0.013987513	0.002498687	0.027769226	7.98564406304658e-19	2.59905990501886e-18	0.009583372	0	0.006986871	0.000288009	0	0.011628475	0.049443136	0	7.30173761073175e-19	0.129381583	0.033014211	0.007643278	0	0.000557896	0
0.04947854	0	0.167314705	0.208266173	0	0.00153838	0.015473294	0	1.17579945052524e-18	0.010425954	0	0.010117451	0.003332443	0	0.019407425	0.01815595	0	0	0.155314422	0.016602279	0.009719363	2.06106855955081e-19	0.001507718	7.98205394680796e-20
0	0.02875023	0.199392861	0.263598906	0.100533911	0	0.008697821	0.006465356	1.17221086510419e-18	0.030485218	8.43273003440507e-19	0.009940874	5.63682038016328e-18	1.28363041506511e-17	0.019011395	4.03147583583329e-19	2.13185472319458e-20	7.15070118446621e-18	6.48652744555863e-18	0.02403583	0	4.88062368904096e-18	1.12454801384527e-19	0.000233635
9.01156130746915e-18	0.047162902	0.195159437	0.268608617	0.146543755	0	0.022490214	0.007892039	9.97511016642251e-19	0.036757751	8.71078883871478e-19	0.013364575	0.001115309	6.58531259502517e-18	0	0.0052637	0.016341954	5.56410921801059e-19	7.80721024234397e-20	0.045910538	8.49967463816827e-05	3.04730922647258e-18	0	0.002128607
0	0.037205638	0.18674003	0.318538107	0.130326372	0.000138427	0.020472327	0.009534178	0	0.032081862	3.73078940512431e-18	0.012367683	0.00033081	0	0.004568661	0	0.002253207	4.75910053020322e-19	0.004366328	0.036075835	0	0	2.74921504463392e-21	0
2.90622383550593e-17	0.039197232	0.206479918	0.258347285	0.183750748	0.000103613	0.009532797	0.010281046	0	0.037403386	0	0.008639242	0	0	0.008661288	2.57071570333799e-18	0.002067131	6.98137911566702e-18	9.31944844096652e-18	0.028492567	0	2.44899335596213e-18	1.80329046596205e-20	0
0.026874431	0.057755813	0.188763323	0.405177074	0.102235454	0	0.033955434	0.012036498	0	0.035279313	0	0.017288632	0.001083441	0	8.75157664028607e-05	5.64472880435691e-20	0.001668262	5.79637163635643e-19	0.025852878	0.046398477	9.58904811522732e-20	4.0015908575682e-19	3.80655840436992e-20	2.6063981811276e-20
0.013827567	0.017481852	0.162883481	0.252101577	0.012936044	9.85470592539277e-20	0.001747335	0.028846443	0.003895984	0.04939235	0	0.006912759	0.002174154	0	0.009019745	0.009964851	0.013923118	0	0.101009671	0.009506064	0	0	0	5.85090846152286e-20
0.006348916	0.013797127	0.175212609	0.195681142	5.3703368822305e-18	0	0.007250749	0	0	0.01293627	5.93116620764679e-20	3.25065187923855e-20	0.001371372	5.60442720799713e-19	0.030020033	1.38996351986905e-18	0	0	0.218703061	0.005799453	0.011516821	0.000315724	0.002186574	0
1.35163649265015e-17	0	0.182987555	0.125231897	1.79301805452371e-18	0	0	1.08095087970511e-18	0	0.011227894	0	0.004001955	0.000569678	1.30413826615135e-18	0.036924049	2.67767791843856e-19	1.70444336492677e-17	0	0.108672564	0.004035511	0.004417058	0	0.000736023	2.15995938810674e-18
0	0.015906506	0.17307182	0.16335051	0.004018085	0.001300466	0.002836913	9.67075455304568e-19	0	0.004012929	0	0.003777662	0.000866902	0.125277936	0.035531959	0.009298989	1.08527401718945e-18	3.38380468148631e-19	0.021888402	0.004423291	5.23405407731045e-19	0.053134469	0.000690081	4.69647594777589e-19
0.005675006	9.99249560747346e-18	0.178156386	0.092372922	0.013574264	0.003775311	3.02004548697989e-18	0	2.45994042758801e-18	0.003728792	0	0.002613252	0.000405815	0	0.024377697	0.027898401	7.11094665589646e-18	0	0.000148442	6.09457030517362e-18	0	0	0.000529705	0
1.54815801170491e-17	0.032397072	0.197171702	0.205631219	0.037145602	0.000229126	0.000727191	0.00813298	0	0.018875676	0	0.00875374	0.001527983	3.31304984170564e-18	0.025113783	6.12486903588122e-21	0	0	0.01846899	0.002139794	0.001308772	6.84591374523566e-19	0	0
3.77797087864196e-18	0.03061573	0.198894322	0.327055257	0.031586033	3.02414928936646e-05	0.007548595	0.010693474	0	0.03234023	0	0.010727703	7.35244909990085e-19	0	0.019313439	1.50895701196644e-20	4.39516122559323e-18	0	8.51902399281135e-18	0.028283674	0.001794266	6.40167701463314e-18	0	0
0	0.092586395	0.198103129	0.368134431	0.230365974	0	0.029266414	0.01027998	0	0.029631946	1.29461085681878e-18	0.016405969	0.000711387	0	0	1.39014512114977e-19	0.013105232	4.93015354084357e-19	0.022916419	0.042698836	2.36946272841261e-19	0	0	8.69757366701733e-21
0	0.030729315	0.198396076	0.346326256	0.057026625	2.2245368355605e-05	0.027816228	0.004686601	0	0.02571233	1.89319554254077e-18	0.013670472	0.001030054	0	0.018186998	9.16264725704866e-19	7.38411220337327e-19	6.68158559991499e-19	0.013246001	0.038170546	0.002451898	4.43784593317688e-18	7.61265876730215e-20	0
7.28498750820192e-18	0.002709562	0.193655181	0.086152777	0.006344781	0.000660139	2.80093397805903e-19	0	0	0.013551042	0	0.002796619	0	0	0.024952316	0	0.001227747	5.17148437149525e-19	1.98416285731557e-17	9.64762733845223e-19	0	1.09594705666331e-19	0	0.001715658
1.45386166732604e-17	0.026591925	0.190674035	0.28826617	0.062279233	0.006944258	0	0.006522451	5.02298166029971e-19	0.029690296	0	0.008322352	8.50274654031672e-20	0	0.016051028	0.000929124	5.95120341561693e-19	3.90123450574243e-19	7.47196866579804e-19	0.014442362	4.18087341461184e-20	0	0	8.10617057505949e-19
0.008694738	0.024118295	0.191202019	0.347012832	0.023235404	0.000922634	0.005969507	0.005523922	0	0.025146886	1.08986424742562e-18	0.009155819	0.001365451	1.11244458014641e-17	0.021510055	0.010868572	1.72960536291456e-17	3.97984856461196e-20	0.004479863	0.009503167	0.001753172	5.65735689635089e-18	6.58124830258962e-21	0
0	0.025156562	0.202840864	0.359287091	0.11786316	0.000123482	0.009155168	0.01159717	0	0.036013533	3.11797597408663e-19	0.011506214	4.98705398662053e-19	1.29430080662526e-17	0.017985267	4.01843352381777e-19	0.007444505	0	1.32733793383473e-17	0.028905318	1.26525593442451e-19	0	2.70772015959055e-21	0.003326229
2.98005253488285e-17	0.034542642	0.204424176	0.288046547	0.064464732	4.2447004945864e-05	0.004560448	0	2.62273409865773e-19	0.019327126	4.72821098052396e-18	0.009074663	0.000511905	1.72522619557911e-18	0.015085117	6.32698308326443e-18	0	6.11172243433389e-19	0.00217351	0.013721114	0	1.39837215206472e-19	0	8.42513185039363e-20
2.26340120707362e-17	0.032172173	0.19502639	0.267508376	0.111497875	0	0.013773145	0.025953499	0.000466106	0.050442316	0	0.012642166	0.000983214	1.74697204298491e-18	0.011585844	0.006028069	0.011678794	4.65236627518927e-19	0.019359722	0.021636784	1.1861180262477e-20	1.82357853529483e-18	1.32134298326138e-19	0.000510809
2.06590288743488e-17	0.024962485	0.201530284	0.277471398	0.095799098	0.000171028	0.008212201	0	0	0.026241939	0	0.005982868	0	7.68515906027353e-18	0.024509931	9.74612359553601e-19	6.53638108051829e-20	0	0	0.018419506	0	8.51578154149227e-18	0	0.004652493
1.45049334002975e-17	0.047991027	0.200711074	0.303691536	0.437710197	0	0.021620441	0.003758353	1.41450145440014e-18	0.034946717	0	0.010698564	0	2.5754812242025e-18	0.014795632	0	0.008660817	2.55049057625802e-20	5.75968681040164e-18	0.043690116	2.62934930670989e-19	0	0	0.0086297
1.62079919611612e-18	0.039758129	0.20520738	0.261316535	0.026094057	6.94529732650913e-05	0.022144006	0.014161129	8.7137580356938e-19	0.035420455	2.78519396115936e-19	0.010073643	0	2.00608099434552e-18	0.020160789	0	0.007119974	2.35135111951959e-19	1.09724859379454e-17	0.03994303	0.000750978	0	2.11052976554561e-21	0.001606032
0.013789798	0.022531297	0.197509955	0.422850043	0.04348837	4.42194560006209e-05	0.003056163	0.005340813	0	0.024447318	0	0.006724165	0.001422347	0	0.009854606	0	1.8927540476815e-17	4.91138338860136e-19	0.012650291	0.014773881	0	8.56260606384928e-18	5.15668923963205e-20	1.1781406186487e-18
0	0.018477055	0.19969266	0.32255642	0.063697639	2.11411592246796e-05	0.022949828	0	3.66397605719887e-18	0.025876877	1.87418533778065e-18	0.012675365	0.000637871	0	0.015648392	0	0.001257567	6.85180703286333e-19	0.019542393	0.038751654	1.82973020397266e-20	2.21292885184384e-17	4.24629610832634e-22	0.007289735
2.29496039356582e-17	0	0.203379115	0.131262813	3.37507176381782e-18	0.000881084	0	0	0	0.001571232	2.56128808922594e-18	0.003532427	0	7.74109014405955e-18	0.021375965	1.59097427082593e-18	0	0	5.68923878637021e-18	1.04312835854298e-17	0.001677064	6.62476621211827e-18	0	6.74511128089924e-19
9.82922210436581e-18	0	0.197879744	0.134415349	1.68495383846386e-19	0.001007144	2.10536563647799e-18	3.15209161196431e-18	3.8498718231679e-19	2.39508907883999e-05	0	0.000565059	0	0	0.042466678	1.59501788382428e-18	0	1.67966184200734e-18	1.41391963847824e-17	3.12826368471393e-19	0.008329141	0	0.001319168	0.000927816
0	0	0.206360159	0.145396218	0.023370104	0	0	1.17103026299912e-18	2.5726109828367e-19	0.013116307	0	0.003496306	0.001516047	1.31832604729968e-17	0.01046199	0	9.10768534059961e-21	9.48710998202467e-18	0.059462856	1.94123567134815e-18	0	6.33870715527546e-18	0	0.002949219
0.014736402	0.017536657	0.17907658	0.257506605	0	0.000447854	0.010039374	0	0	0.013097499	6.87201559142798e-20	0.008357351	0.001527839	0	0.035792986	0	0	6.27465583217971e-19	0.052923304	0.01874286	0.006907897	0	0	0
0	0.024008607	0.198033466	0.221471961	0.007752427	0	0.000928126	8.06020952996188e-20	0	0.012627143	0	0.0079962	0.000435823	0	0.014722523	0	1.13839740325444e-19	6.14421036359637e-19	0	0.003155821	4.54057423938973e-05	0	0	0.000590648
1.20266514146053e-17	0.006113357	0.193401862	0.157605746	0	0	1.85686554452465e-18	2.01668656270898e-18	0	0.005092509	9.05784635946254e-19	0	0	1.1862027758575e-17	0.031479277	0	0	3.91372448344472e-19	3.54776107759299e-18	5.45117505429404e-18	0.008519238	0	0.00056024	0
1.2633809383009e-17	0.026976099	0.20436187	0.273667064	0.087107626	0	0.00930872	0.004904472	5.65532281861844e-19	0.020782784	0	0.009141032	0	0	0.014422182	9.20281261463005e-20	0.012226851	5.13358761139367e-19	5.52350593338769e-18	0.017769958	0.000133848	3.56845243250001e-18	0	0.006072025
4.445066330979e-18	0.032790014	0.200732169	0.222389022	0.054774591	0.000229317	0.005992951	0.005868672	0	0.030835998	0	0.010084234	2.8108465135936e-18	0	0.009488476	5.06377419880668e-19	2.96778694129612e-20	3.26350694190099e-19	0	0.01300113	0.012535699	3.23197759637993e-18	0	0
3.03318243248369e-17	0.013757279	0.205510716	0.252421564	0.004060787	5.26420882843357e-05	0.005073222	0.001898934	0	0.021339	0	0.010389338	3.06573631298874e-19	1.03367900863132e-17	0.012155116	0	6.77822971173443e-18	4.07656313116948e-19	5.7097688733669e-19	0.016989931	0.000562178	0	0	8.01799847589446e-19
0	0.04082958	0.201199329	0.293202854	0.090600677	2.37956088699689e-05	0.009390955	0.009954354	4.01906999000032e-18	0.03256875	2.96445695938354e-18	0.008713319	1.91940776923007e-18	7.36064491458663e-18	0.026773235	2.51323765565294e-18	1.61784999582511e-17	0	0	0.021753754	0.00257859	6.54368650174798e-19	9.72176854194282e-20	0
3.8354199436225e-17	0.031698839	0.203877402	0.288107056	0.00504976	0	0.009628184	0.008642771	0	0.023802169	2.00452248408308e-18	0.013070165	0	6.2594060319749e-18	0.006739312	2.15678705725026e-18	0.00325761	3.55167237710481e-19	0	0.019045966	6.61970476966994e-20	0	2.08242257162924e-21	0.002359159
0	0.029900961	0.202350989	0.31897516	0.050747408	0	0.010450527	0.008365015	5.22244786232383e-19	0.034785707	0	0.011831586	0	1.60913003404301e-17	0.0143035	0	4.04674392743134e-19	6.3038955176967e-20	0	0.021004378	0	0	0	0.001272203
0	0.038988695	0.201371504	0.272960748	0.054752519	0	0.015530377	0.004365235	0	0.025100379	1.74979666516958e-18	0.012177469	0	1.80538438872178e-18	0.001936236	2.92157940272491e-18	0.011694185	2.93822870779058e-19	9.65935573369722e-18	0.027056448	0.004608434	5.78419865182571e-18	0	0.006731997
4.2819962908039e-17	0.028291939	0.206906955	0.337079855	0.041275134	0	0.006688755	0.010695424	3.35376869507613e-18	0.032943514	0	0.012410464	0	9.35288905811085e-18	0.001507207	2.15080338111461e-18	0.018826259	1.63093389524567e-19	1.22333552658794e-17	0.012663318	1.20304599077553e-20	0	2.66057489244762e-20	0.007801938
0	0.022961674	0.202020057	0.316449915	0.162008865	1.23233414440355e-05	0.014533035	0.004156269	0	0.032113213	2.54016938226803e-18	0.009580198	0	0	0.008383974	0.000723503	0	4.03509636347945e-19	4.74478619486313e-18	0.02050102	5.22147056578936e-20	7.48679247174091e-18	0	2.02935974996494e-19
0	0.029995846	0.189327929	0.311418128	0.033495234	1.29767082872184e-19	0.014502817	0.020045223	0.000251129	0.050593582	0.00094405	0.008570448	5.30828407822405e-21	0	0.002027614	0.008311542	0.035511363	5.11052160975203e-19	0	0.034561562	1.06186226367168e-20	1.95999966139368e-18	1.64602910938022e-19	0.016431427
5.56942746619493e-18	0.005247313	0.200432037	0.190376761	0.093698686	8.70236716381929e-05	0.010865749	0.006478198	0	0.040852799	0	0.01015789	9.01114436580441e-20	0	0.004240133	0.002294941	0.083519196	4.3233524013582e-19	0	0.020550931	0.002195824	3.97493879264527e-19	3.03178645736513e-20	0.078415968
0.024323986	0.004049043	0.189747939	0.186342533	0.023065645	0	0.007201709	0	0	0.008840635	8.48026666752957e-20	0.013222345	0.002245959	0.000320864	0.023341268	2.17662935165177e-18	1.29723000184246e-18	0	0.108278807	0.005049169	5.36054922563202e-23	0.007986321	0	0
2.62811781905354e-18	0	0.196516781	0.12341777	0.012625328	0.000957252	6.02640438229266e-19	1.84314758719654e-18	7.14169421747733e-18	0.003311249	8.57113023841794e-20	0.002340293	0.001440182	1.45615468643908e-19	0.03230735	0	0	1.41285434245979e-17	0.030768884	0	0.000356047	0	0.000783676	2.0846535830959e-20
0	0.014499755	0.196067425	0.176904036	0.13622446	9.50974684192233e-20	0.005006785	0.01093824	0	0.0394286	0	0.005399605	0.00075432	0	0.007164092	0	0.016439971	0	0.035872416	0.021917713	1.08961225258269e-19	2.30891753031958e-19	0	0.001961387
2.0663115864729e-17	0.021124072	0.201915944	0.325756453	0.079979746	0.000100361	0.018700775	0.005808875	1.39555231461297e-19	0.02870517	0	0.009155409	0	6.38642145580119e-18	0.021267061	0	0	1.81260133922365e-19	0	0.029591841	0.001245079	0	4.72099059070992e-20	0.001297798
0	0.016507072	0.199944392	0.229298971	0	0	0.014519018	0	0	0.019776361	1.85218887060225e-19	0.009573937	0.000818728	0	0.02571776	0	2.33937589774905e-18	5.91046100907536e-19	0.02455848	0.021238497	0.001440931	3.0885755881994e-18	0	0
2.96380589118467e-19	0.003786555	0.170818442	0.078655382	0.004955294	0.001568154	0.000215004	0.00088338	0	0.018533652	0	0.002836491	0.000902261	0	0.02104283	0.018387952	0.00076319	2.83206255905028e-18	0.083877186	0.00086707	0.000932463	0.000929307	0.000278759	0
0.004781806	0.060608598	0.181455123	0.306578839	0.175165651	6.76739405153109e-21	0.044907959	0.009019559	0	0.030253312	1.2470790635014e-20	0.019857716	0.001427442	0	0.004028042	0	0.005923357	5.84680356825155e-19	0.038225629	0.056224968	5.45626461357433e-20	5.92065654913808e-20	0	0
0.009182606	0.012316626	0.197890541	0.307627455	1.99249101060074e-21	0.000514679	0.023724276	0	0	0.012484221	1.7294966251476e-18	0.013066905	0.001897386	0	0.031644009	0.002897643	0	7.4133103042288e-19	0.003776064	0.026961112	0.003468889	8.84450078007423e-18	0.000221679	2.86279894475077e-19
6.30472400286431e-18	0.031699297	0.191254507	0.255477697	0.057156857	0.00027996	0.013517072	0.007825357	1.16353000441751e-18	0.03592047	1.34409999082609e-18	0.009677486	0.000692963	0	0.022466245	3.21514737431929e-19	1.77883128324911e-18	4.44532936566094e-19	0.0156116	0.023140146	0.001827373	7.82305462828009e-18	0	6.85405712191495e-19
0	0.028420292	0.20065192	0.285447529	0.096462132	0.001225898	0.00707923	0.003324156	0	0.020819825	5.34993563587958e-18	0.00949557	3.37500210612289e-20	3.9678328087048e-18	0.018953237	3.21094452169305e-18	0	3.26711027185648e-19	1.60336045224247e-19	0.014984171	6.86846308940465e-05	0	1.64515303420713e-20	1.94124458723711e-18
0	0	0.18882547	0.147393489	0.029232611	0	0.001467766	0	0	2.05470777207485e-19	0	3.10906363557247e-20	0.002611806	0	0.035579145	0	7.85266044580528e-18	1.36498381521969e-17	0.090857428	0	0.013671176	0	0.000160993	0
0	2.28047492364912e-18	0.193546835	0.286024799	3.28936467937139e-18	1.50681721853575e-19	0.00253153	0	0	0.00389428	0	0.005924166	0.001139766	0	0.039156847	4.85557886573285e-18	0	1.511518988876e-17	0.047583873	4.08143542557366e-20	0.003250666	0	0.000471801	0
4.79926936408318e-18	0.023856426	0.201430329	0.208821452	0.032718733	0.000555112	0.005337042	0.003440629	0	0.027000521	9.98024395768944e-19	0.007684132	0.000563414	4.25213686677704e-18	0.027134296	0	1.02322573706323e-19	0	2.85576136144168e-19	0.013752053	0.001498649	0	1.63278961904619e-23	8.52452747379415e-23
0	0.033269797	0.20547042	0.330464958	0.06117949	0.000290155	0.002994745	0.005219307	0	0.024566743	8.90060699920311e-19	0.013879292	5.3555167240242e-18	2.39012143316467e-17	0.02283966	1.17993781522068e-17	2.77950538792166e-19	1.39898347799202e-17	0	0.013292688	0.001117538	3.38385484518738e-18	0	2.13149077578789e-21
0	0.008284006	0.189394217	0.282526744	0	8.34905242774091e-20	4.91828958654751e-20	1.60229751309536e-18	0	0.005250075	0	0.006072605	0.003372812	1.02674843494527e-17	0.044172796	2.51763145554814e-18	4.6066790915423e-18	0	0.053504567	0	0.003218152	1.05133032930543e-18	0.001449123	1.63603710380831e-19
8.35619564372097e-18	0.010857427	0.201079137	0.206275193	0.038412417	0.000764186	6.21519588950424e-19	6.02882326086502e-19	0	0.008880436	9.60924057905215e-19	0.005409808	3.52749365496903e-19	1.01640570383919e-17	0.031677607	2.83487408845658e-18	0	0	0	7.3675518749879e-19	0.001700038	2.87493449359716e-18	1.23175699950204e-20	0
0	0.009960062	0.205368294	0.25966795	0.013238931	0.000274065	0.001083349	1.64730005933959e-18	2.64210603610442e-19	0.008599662	0	0.008270314	0.001542927	0	0.02781382	0	0	6.91675699224381e-19	0.015778967	0.003772546	0.001667804	0	4.07533922572316e-05	0
2.86452863240012e-17	0.010896374	0.204121904	0.288732812	0.013800047	0.000748775	1.3549239909464e-20	1.08702476990868e-18	6.12298580012635e-18	0.00776369	0	0.007916849	0	0	0.035228162	7.40374667311286e-19	1.18786706154164e-17	4.9172746037287e-19	0	0.002012683	0.001396324	0	0	0.000278164
0.016730137	0.00994491	0.197343953	0.222676311	0	0.000327879	0.00790893	2.25363392967696e-19	5.34582821690435e-19	0.009881305	0	0.013670133	0.001381827	0	0.023532272	0	3.43891414311386e-19	7.49696086032325e-18	0.008863108	0.007922854	0.000222327	0	0	2.41642799072872e-19
0	0.036596625	0.196882297	0.304496499	0.000340816	0.000223446	0.004395224	0.011525701	0	0.026918086	1.62588690246433e-18	0.012158906	0.001468358	7.84998162107824e-18	0.026647436	0	2.73545993717857e-05	5.68381851614322e-19	0.033416218	0.021624626	0.000688506	0	6.33751636392872e-21	7.34776583843094e-19
5.4755735645789e-18	0.048021081	0.179560817	0.24785474	0.251972353	0	0.013589527	0.048522082	0.008483048	0.080888668	0.00571825	0.009159467	0.002094047	2.06835823925821e-18	0.000638414	0.00248526	0.023165388	2.60856441505425e-19	0	0.02822376	0	0	0	1.7778611570008e-20
0	0.002985877	0.206536241	0.241381888	0	0	9.05394290340128e-20	7.93731920837385e-18	1.51993028165601e-18	0.007003319	0	0.008364585	0.000783317	3.69212259932436e-18	0.02345798	9.52182781660117e-19	0	8.82210526770926e-19	0.019681232	1.8869792024669e-19	0.00025716	2.07892025057567e-18	1.22076033453001e-05	0.001171183
3.86925222566879e-17	0.040908649	0.205230223	0.331302636	0.085965101	1.29609474787177e-19	0.008185575	0.003236675	3.94948620692421e-19	0.024439321	8.85945522100594e-19	0.010661533	3.33018886236812e-19	9.52986860571785e-18	0.02131714	0	0.00816206	5.32709810489599e-19	0	0.021146903	0.000423611	5.22961792564668e-18	0	0.007318374
0	0.013940373	0.194577578	0.360540802	0.065452706	0.00084969	0.005245328	2.32955097837883e-20	5.90515054979514e-18	0.012393648	1.78364479880631e-18	0.014881582	0.00019184	2.08665747599779e-17	0.037722214	0	2.76103258920456e-18	8.26826204249337e-19	0.04146184	0.010883976	0.000552092	4.26304597992188e-18	1.6333963627305e-23	0
0	0.024232631	0.205999419	0.224287457	0.009278211	0.000620119	3.1709160182095e-19	0	1.46447112623176e-18	0.006515865	9.63563454994185e-19	0.009747733	0.000358427	1.26786430931162e-18	0.034372645	1.52531008955886e-18	0	6.96021125692446e-19	0.005299481	7.89902520331326e-19	0.000132412	2.06243689801723e-18	0	0
1.96732993139081e-17	0	0.197545034	0.137850203	0	1.78047358380684e-20	0	1.20710583416813e-18	1.18654384126763e-17	0.010182539	2.97749921933889e-20	0.005500371	0.000718895	0	0.028075586	0	0	0	0.019802391	2.85157278504914e-19	0	0	0	2.87037626212694e-19
1.60570881612244e-17	8.54617012864358e-19	0.195975802	0.101654506	0	0.000494924	0	0	0	0.004845967	0	0.005707841	5.36994758682435e-19	8.15429178666784e-18	0.035783423	0	0	0	0	8.73570210008183e-19	0.000620843	0	0.000327727	0.000118458
0	0.040869949	0.196388183	0.314455823	0.0210195	8.96015577950423e-22	0.010093528	0.01476459	0	0.042123828	2.5424124030807e-18	0.008450289	0	0	0.006202579	4.09249366185213e-18	0.002937549	2.57682042871781e-19	0	0.027058197	1.89887665129709e-19	1.99447152239346e-18	0	0.002001129
0	0.008909599	0.201598352	0.306639626	0.008617345	0.000168428	0.006612472	2.66892228017768e-18	9.11687821954164e-19	0.007537511	2.17205037664726e-19	0.007613647	0.001513242	0	0.026831925	0	0	7.29968536035467e-19	0.023419342	0.009821873	0	0	0	0
4.47767938633346e-18	0.014135067	0.18848973	0.24464073	0.001814071	0.000537814	0.002023559	1.25671194187353e-19	3.43877133976914e-18	0.0091811	9.75572319429193e-19	0.006589582	0.001476164	2.84365818142458e-18	0.03259354	1.20045499009107e-18	0	9.56805321705721e-19	0.085467187	4.26703366635339e-20	0.007357172	2.60816171658425e-19	0.001469588	1.47693243882426e-05
0	1.27689576006124e-18	0.195338552	0.205303777	1.05167799741818e-18	0	0.002094427	3.36584278625693e-18	5.68477246275684e-18	0.006458451	0	0.009235577	0.0024935	0	0.020522278	0	0	0	0.082939686	0.007124441	0.006936265	0	0.000460585	1.1565843088548e-18
1.35233231742466e-17	0.007567344	0.196056092	0.269957512	0.026575721	0.005730489	0.000882694	0	6.99819341010493e-19	0.016671181	2.62434128025016e-19	0.018809395	0	0	0.038902214	0.013911291	5.20886619253691e-19	0	0	0.008431464	0	0	0	0.000690618
6.0000132190654e-18	0.046245389	0.194963447	0.22354976	0.089689353	0	0.007179709	0.013778753	2.20299040225826e-19	0.043756321	0.000452782	0.008090296	1.68725608096201e-19	0	0.005447768	2.44738146497383e-18	0.028579785	8.68514133823191e-18	1.22986202580845e-17	0.033686782	0.000227715	1.02987958980387e-18	0	0.008378431
9.99493876089688e-18	0.019797689	0.185987137	0.413519308	0.160867805	0	0.040384646	0.008616802	0	0.033011135	0	0.01033117	0.001296467	0	0.03333388	1.51953678278909e-19	0.005005948	7.14413334837752e-19	0.046289799	0.063712059	0.000384647	5.54563976982794e-19	5.53026675792698e-20	0.00619669
1.17133140283877e-18	0.025738556	0.194788574	0.453549626	0.080411186	0	0.016411409	0.001494508	0	0.024201237	7.80531236014913e-18	0.01690723	0	4.01969344850307e-17	0.011450191	0	8.03735124528079e-18	2.98722425167746e-19	4.37356124479344e-17	0.031715053	0.002562384	4.35741670090903e-18	4.03742158108112e-20	8.23060678522404e-17
3.51905319333499e-17	0.026501104	0.203105753	0.310639697	0.088745187	0	0.017364456	0.013071319	0.000324689	0.028421411	1.78248454723491e-18	0.014206632	4.49202308074382e-19	5.41241378488493e-18	0.01173697	0	0.003378293	1.43416165808171e-19	0	0.024183484	4.16159891254628e-20	6.21829167637215e-18	9.08775310658392e-20	0.001891534
2.29619170002354e-18	0.028158586	0.194960291	0.28228082	0.091589037	0	0.016391534	0.004219187	0	0.02435512	3.66506487274481e-20	0.010874848	0.001489739	0	0.004084795	3.65091488568351e-18	0.005310459	6.24165138992434e-19	0.039889429	0.026393905	0.000910414	8.14584683530602e-19	0	0.001599648
0.031029357	4.86101603139855e-24	0.192756576	0.224090082	0.032279054	0.000282329	0.0117784	0	0	0.00499617	0	0.008541592	0.001792259	2.32004215804436e-18	0.026352899	7.32285631565883e-19	0	5.18966173736995e-19	0.072717359	0.008711434	0.003554668	0	0	0
0.003952791	0.044467084	0.195991978	0.274074911	0.096351592	0	0.020406409	0.003682315	1.93594961863778e-18	0.024274877	3.56737446205948e-19	0.007750613	0.001264566	0	0.006778945	2.81198604781279e-19	0.001063779	4.0803897838644e-19	0.042755178	0.026754113	0.003938937	0	4.34382597231284e-20	0.000776544
1.72325137793713e-18	0.027780957	0.197309434	0.259228622	0.133747835	0	0.018451169	0.003994792	7.61915297331976e-19	0.030510551	0	0.0091522	0.001091689	1.6933407721828e-18	0.00367951	0.00287433	0.036876698	6.8000663355822e-19	0.007117376	0.035023624	0	2.31577316068169e-19	1.35745833872389e-19	0.01546932
3.40568389419508e-18	0.022551496	0.197131151	0.217500399	0.09208845	0.000115095	0.019124033	0.005794694	0	0.023688297	0	0.011630499	2.45128622273813e-19	6.44014832844826e-19	0.017539917	0	0.010163788	5.36063680072575e-18	1.57590482595459e-19	0.032741922	0	0	0	0.003009998
1.9585924573621e-17	0.013866985	0.187426333	0.229923021	0.072799031	4.10104921425014e-20	0.003212563	0	0	0.011654056	2.51733785704059e-19	0.008990062	0	0	0.032761599	1.43992709231758e-18	3.24062217584551e-18	7.21341142169174e-18	0	0.006651687	0.006083594	0	0.000231642	4.47099717836655e-21
3.64420463845118e-18	2.25273600663477e-18	0.191348641	0.180634497	0	0.00080458	0.005945501	0	9.22651110262831e-19	0.006192159	0	0.002282176	0.001405736	2.7141888837623e-18	0.028178291	5.25682589825467e-19	0	7.620805147324e-19	0.107086753	0.000506866	0.004760268	7.2763804491018e-19	0.000850593	0
0.012755945	0.020469474	0.176651413	0.226768945	0.087787288	2.62199593283875e-19	0.021429605	4.23778486600452e-19	0	0.014528914	3.35134771544324e-19	0.006539805	0.002025537	0.0166004	0.027572448	3.45268703008922e-18	9.32229427273063e-19	1.09838175697869e-18	0.19900053	0.026854368	0.009797285	0.017911907	0	0
5.41842757086943e-18	5.43001286115774e-19	0.200983252	0.228450479	0	0.000313474	0.001420932	0	6.50121074406341e-18	0.004968871	5.8533790454478e-19	0.008761248	0.002179192	0	0.038098533	0	0	9.56122988680717e-19	0.03383959	0.001027073	0.002714421	5.91708894699277e-18	0.000503967	0
9.02615310238668e-18	8.87241219005595e-20	0.190883328	0.104621676	9.1905538526236e-20	1.00812746401736e-19	0	0	1.84905668295257e-17	0.003882248	7.79549927977101e-20	0.001213052	0.001394627	0	0.030758585	3.40489674654716e-18	2.60460869028178e-18	1.50155423133939e-17	0.10695085	3.2682191216804e-22	0.010311283	8.31031049925821e-19	0.000802518	0
2.15093989750407e-18	0	0.185970904	0.12048312	2.54318623078392e-19	0.00021322	0	0	6.88714790566694e-19	0.000579706	5.85276736389868e-19	0.000751476	0.001465201	0	0.040800518	2.58753460128353e-19	2.2691512511243e-18	0	0.051756697	2.70764735151721e-18	0.00963524	1.34395203227016e-18	0.001334566	1.54333483971546e-18
6.18510543038876e-18	0.025578634	0.196767106	0.207423578	0.045926804	0.000175214	0.011875011	0.005609312	0	0.029160107	3.4160738998442e-21	0.009374736	0.000540944	1.26899252143781e-17	0.029401529	2.38263561360578e-19	0.006784712	5.57842507624963e-18	0.008536967	0.025929415	0.002033371	0	5.43771487951324e-20	0.00436962
0	0.012729934	0.202341936	0.255487447	0.0344764	0	0.010383706	5.24066263534345e-19	3.8008282550914e-18	0.015311648	1.99290809911011e-18	0.008462151	0.000390744	4.66647694967759e-18	0.031067472	0	0	7.57021299851474e-18	5.92318758312319e-20	0.013293433	2.66975010089988e-19	0	0	8.65752301667481e-20
2.58647677461013e-18	1.73250336971073e-18	0.175615702	0.021461459	1.39683267121227e-18	5.46440941127075e-19	6.749211863412e-19	2.5367571726439e-18	0	0.004009844	0	0	0.000191727	1.97492225257854e-18	0	0.003011761	0	4.09016687122435e-19	0.108156561	0	0.001955896	0.010464347	0	1.15036621554648e-19
2.50527677735964e-17	0.036022886	0.195222555	0.370478706	0.122829318	0.000635754	0.043518389	0.013389521	1.46001337297124e-05	0.044253857	2.2814259199521e-20	0.013651423	0	9.2221718428789e-18	0.028867849	0.002085617	2.19742853394386e-20	0	0	0.064417615	5.8809872184548e-19	0	0	0.002523739
1.02032382549742e-17	0	0.190180817	0.020001153	6.63292945273465e-18	0.001530859	2.97205009860792e-18	3.60230773307051e-19	0	0.001120713	0	0.000464525	0	0.011545637	0.030202641	5.56121740259187e-18	9.84913383605756e-18	0	1.42967019530273e-18	0	0.003408568	8.67431483853877e-06	0	4.10470993952669e-19
5.64780759714454e-18	0.008753158	0.208609457	0.20757998	0.012604963	1.92296671565933e-05	0.001940208	0.000840038	0	0.013118218	2.0922146417419e-18	0.007970629	0	5.3855900748092e-18	0.015970087	0	0	9.38934323469245e-18	2.02697609549216e-19	0.006974484	0.003625385	2.85639494865467e-18	3.27437889789236e-20	0
0	0.024780677	0.200693473	0.262435462	0.016288655	0	0.014451534	0.005991855	0	0.022641481	1.67938417760989e-19	0.013186647	0.000867045	0	0.007510609	0	0.010659236	6.17317755719642e-19	0.011676312	0.025807231	9.31807565260004e-20	0	0	0.002880808
0	0.000745157	0.180459876	0.12717651	3.01334627914782e-19	0	0.003251162	3.74675547151186e-18	9.04190669675304e-19	0.014738329	4.92035182960614e-19	0.001948388	9.10275536426789e-07	8.47282978209799e-18	0.002481728	0.001182738	0.002156084	0	0.108082278	0.004721816	0.003135997	0.002034128	0.000753526	0
0.013523713	0.004305086	0.182992716	0.117179899	4.46406180445743e-18	0.000382654	0.004343552	0	7.4199350290314e-19	0.008251407	7.83668303599984e-20	0.00784074	0.002081276	0	0.028480422	9.8040071045994e-19	1.87215272647956e-18	0	0.13676091	0.003495752	0.003527357	7.7166257599547e-19	0	4.85897732401874e-20
3.34691526416412e-19	0.041344276	0.194292773	0.294383005	0.116370769	0	0.021464236	0.009292096	0	0.037244748	7.61051171311808e-21	0.007642275	0.001481472	2.55511644198524e-18	0.010817039	0	0.026384453	4.80716371085438e-19	0.00358491	0.039676478	2.10270843750181e-19	0	4.30438851351541e-21	0.00629616

NK cells	NKT	Osteoblast	pDC	Pericytes	Plasma cells	Platelets	Preadipocytes	pro B-cells	Sebocytes	Skeletal muscle	Smooth muscle	Tgd cells	Th1 cells	Th2 cells	Tregs	ImmuneScore	StromaScore	MicroenvironmentScore
0	0.011650872	0.038939802	6.82477639400749e-18	0.037829978	0.004503714	0.001083779	0	9.37671814086821e-18	0.000755725	0.002242491	0.145820044	5.26262965192082e-18	0.048242382	3.4486391699794e-19	6.60698091984987e-19	0.000457803	0.053343094	0.053800897
0	0.008854795	0.061016883	2.81826742406824e-18	0.081506426	0.004564163	7.31913516842651e-19	0.012887402	0	1.05857953127694e-05	6.74326779301053e-19	0.132930007	5.37824402883256e-19	0.015204813	0.001415872	0	0.000269591	0.047306651	0.047576242
0	0.029293314	0.029199972	2.84374203266362e-18	0.018345976	1.75051248582849e-19	2.52865377575579e-19	0	1.88989757460461e-18	0.001102646	0.001874845	0.137619273	8.73290485215286e-18	0.035835004	7.25512619546205e-18	0	0.001372149	0.029045677	0.030417825
4.15120861255935e-18	0.056779975	0.020077875	5.84561373754571e-18	0.067458302	0.001668029	4.8189355728708e-19	0	1.91780097637273e-17	0.000580614	1.13075977339581e-19	0.051492423	0	0.034479988	4.93207777182325e-18	3.85506815993395e-18	0.014912337	0.042438828	0.057351165
0	0	0.011822734	0	0.033150385	1.08073463487362e-20	2.16106645974791e-19	4.91238253781573e-18	1.78812970627515e-19	0.002814524	2.89735937333063e-18	0.14523923	0	1.4291462632145e-18	1.05233720700901e-19	0	0.022547826	0.076717826	0.099265652
0	0	0.045512811	5.9886562868063e-18	0.09138432	0	0	0.003535425	3.03795362933043e-18	0	0	0.103889006	0	0.017653933	0.015261396	6.07409882097996e-18	0.041278158	0.08810579	0.129383948
0	0.009206676	0.068824237	0	0.060870784	0	1.37383866536263e-19	0.026424132	0	0	0	0.139571225	9.87906034132934e-19	0.011760352	2.57525754535604e-18	2.97724501798344e-20	0.00906334	0.050766072	0.059829412
0	0.018470103	0.030517744	0	0.059917795	0	7.60082345378783e-19	0	0	1.23271464722337e-05	1.59565819122297e-19	0.144201345	1.23909365784689e-18	0.043990204	0	4.02546404550826e-18	0.015745321	0.049086647	0.064831968
0	0.009971882	0.046683696	9.09340369969631e-18	0.037811185	1.7893878215929e-18	0	4.55841445054695e-18	0	0.0006245	0	0.17803247	0	0.009537745	0.048203806	0.009122978	0.055974939	0.045724487	0.101699427
0	0.009415097	0.098011664	0.003762916	0	0	0	4.895291528325e-18	0	0.019953434	0	0.299418906	0	0.035501713	0.177741185	7.70777669905224e-18	0.012597944	3.33364337446737e-19	0.012597944
0	0	0.049009034	0	0.059217697	0.000344974	1.69966529650528e-20	0.019101717	0	0	0	0.082883865	6.92017539532198e-19	0.021250735	2.86696536608515e-18	0.00259724	0.028887928	0.066676622	0.09556455
1.68830423692874e-19	4.57994394774291e-18	0.073671865	0	0.073415718	0	2.47216989779895e-18	0.022641667	5.02114770974036e-18	0	0	0.124092015	0	0.020931134	0	0	2.57460900274277e-18	0.052667426	0.052667426
1.33080066346877e-18	0	0.081499938	3.81517219261438e-18	0.032162519	0	0.003922925	0.053999353	8.26599120103736e-18	0	0	0.133451354	0	0.012054136	4.17532574946601e-20	0.001964023	0.012265612	0.050219575	0.062485186
9.97663147159646e-20	0	0.060481469	1.43309752010386e-17	0.064907486	0	0	0.025622504	0	6.66594679163337e-05	0	0.107803887	8.71716291342701e-20	0	0.001507926	0.003239768	0.012258907	0.059371514	0.071630421
2.45410884071583e-19	0.019632309	0.045154197	0	0	0.003524488	0	0.024457134	0.001438288	0.00035694	0.001319863	0.088101178	1.45491505047666e-17	0.066156319	0	0.00354823	0.00093529	0.028046278	0.028981568
1.35514310119943e-17	0.046894154	0.027472303	0	0.065755805	0	0	2.57563866281816e-18	0	0.00243612	1.94724444659066e-19	0.061657312	4.01222497245989e-18	0.016687777	1.74490638473915e-18	6.72760658573384e-18	0.009894784	0.120042961	0.129937745
1.47067086469378e-18	0.110289604	0.018479586	7.3659850609195e-18	0.022982508	0.009114482	5.98925461268491e-18	0	0	0.00245066	0	0.143033772	1.45916239228904e-17	0.079674	3.44417435253244e-18	0	0.020656627	0.068694658	0.089351285
0	0	0.070625465	0	0.045394748	4.76370582814669e-21	1.74248483596096e-22	0.01503049	6.86314675570431e-18	0	0	0.098866489	7.44595690759578e-19	0.019048854	0	0.0040635	0.006532547	0.056939435	0.063471982
0	0.006038385	0.041564175	1.29884067740725e-17	0.090414525	0	0.000296911	0.007065973	0	0	1.80412153293344e-19	0.110711475	2.66347569908934e-19	0.004953371	9.65891332073533e-19	0	0.025940342	0.076420358	0.102360701
1.06211373973113e-17	0	0.071160905	8.343964286211e-18	0.092442202	5.34994390556107e-20	1.72538024486736e-20	0.038540846	0	0.000114301	3.01983505051107e-19	0.116945743	0	0.00269124	0.012574016	0.004445023	0.01487548	0.049179768	0.064055248
7.16007438613854e-18	9.48098980331114e-19	0.073868185	6.21843266006189e-18	0.046541477	8.25660756692724e-19	2.33704625145371e-18	0.024516489	2.84765956390808e-17	0	2.67895163394179e-18	0.124512182	0	0.008791242	0	0	0.011071653	0.047467446	0.058539099
1.28138657057741e-17	2.89565634475167e-19	0.056220664	1.30508251054246e-17	0.100135281	0	6.8097425424826e-19	0.022893765	0	2.08037245314116e-20	4.42221827636066e-19	0.150701734	1.0336209977026e-18	0.010811889	0.003533846	0	0.011527724	0.077591475	0.0891192
0	0.038044245	0.017646202	0.002028397	0.043204364	0.007801301	9.56299214208733e-20	0	0	4.44317586371668e-20	0	0.118285576	0	0.018023917	0.117921369	0	0.194867106	0.038621231	0.233488337
0	0.073829583	0	0.000473883	0.075305949	0.005482418	1.62203358979071e-18	1.21299600419236e-17	1.73321885009792e-18	1.12071708833994e-20	0.002618934	0.014329293	0	0.054932254	7.49092884524582e-18	1.1379206795909e-17	6.39688270541061e-18	0.037208202	0.037208202
0	0.055011912	0.029984025	4.6372486329823e-18	0.006181691	0.001045168	0	0	0	0.000445701	0	0.096135865	0	0.089300748	0	0	1.47955952429848e-17	0.042354412	0.042354412
0	0.014276205	0.057098511	0.014214181	3.73028652495256e-18	0.012492215	0	0	0.020355285	0.000125136	3.21979119920764e-19	0.162844148	0	0.035783884	0.043717387	0	0.219670292	0.035241964	0.254912256
9.49704581545039e-19	0.034433129	0.069282856	0	1.11586677660148e-17	0.001496544	1.26422444960422e-18	0	2.87339359481827e-19	0.003246978	0	0.220421653	7.69726363193746e-18	0.025379918	0.007372838	4.34583078627325e-18	0.011230747	0.037369447	0.048600194
0	0.005925941	0.083828729	0	0.017029559	0.003085125	0.001773878	0.035141909	2.30189656482573e-18	0	5.280740838945e-19	0.172935035	3.85726593313696e-18	0.027979998	0.004708681	4.41016283906701e-18	0.009345174	0.055066133	0.064411307
0	6.16380783956458e-19	0.08331962	0	0.041666568	0	0	6.13095250731775e-19	0	0	0	0.208115417	0	0.015739766	0.010744721	3.84599515834303e-18	0.008320856	0.061204918	0.069525774
0	1.38588221755828e-19	0.065579633	0	0.081200872	0	1.82376860435968e-20	0.019730111	3.09317725213142e-18	1.55277785876609e-20	1.88737296527183e-19	0.153287554	0	0.008277915	9.22608264372791e-19	2.05510406119625e-19	0.025752968	0.066814209	0.092567177
0	0	0.070259006	1.08289793814386e-17	0.076787907	2.99298749200445e-18	0	0.052199401	6.95526788630389e-18	0	0	0.138327516	0	0.007839476	6.2230273061017e-19	0	0.003124401	0.064910446	0.068034847
0	0.013298571	0.080223558	1.30017379612169e-18	9.06753235220064e-18	0.013355723	0.004774179	2.47791529947459e-20	9.18363516882407e-18	2.04796042882808e-20	0	0.169239769	0	0.042689514	0	0	0.007030764	0.034839889	0.041870653
1.10603028329637e-17	0	0.06732226	1.09317279924616e-17	0.042415728	0	0	0.03482464	1.02339653974322e-17	0.000687294	3.07336334094534e-19	0.157058172	0	0.008292591	0	0	0.006964364	0.026806078	0.033770443
0	0	0.069134884	1.3506528472017e-17	0.026969891	0	0	0.018110165	0	0.001384052	6.87908902639336e-19	0.222389855	5.71636223643363e-18	0.014940269	0.007399209	2.3847481988435e-18	0.004982229	0.055760715	0.060742943
3.27350601151476e-18	0	0.04652628	1.44583062403268e-18	0.025026861	1.62480647214672e-18	3.26557152364422e-18	0.001278275	0	0	6.23894916437427e-18	0.191451629	6.1130449688683e-19	0.029219706	0.023461212	2.64185378784516e-20	0.031312052	0.044832747	0.076144799
0	0	0.05677418	0	0.044806727	0	3.09792993580828e-21	0.057722059	5.80441166369658e-19	6.1925763203359e-21	1.88301182158394e-19	0.16370134	0	0.003292313	5.78968692748691e-20	0	5.2861813067185e-18	0.057016385	0.057016385
0	0.001895762	0.065613104	0	0.015772872	3.79820308413858e-19	0	0.026555391	8.58976563870099e-18	7.41501340701463e-21	0	0.068622405	0	0.000327246	0.020919772	0	0.030681326	0.051037213	0.081718539
0	9.3971421922619e-18	0.078600411	8.43258762953861e-18	0.054242435	7.69670446179355e-19	0	0.05634053	5.67720901143827e-18	0	6.14491143147566e-18	0.115398467	1.13924018169262e-17	0.005792101	4.69118362943743e-19	3.11238729218177e-19	0.003101662	0.045526338	0.048628
5.13747911299755e-18	0	0.080004165	2.75594069698935e-18	0.083686008	0	0	0.008420074	4.2634034157462e-18	0	0	0.138539346	0	0.014963131	0.000182583	0	0.032100982	0.064091521	0.096192502
0	2.83414781696568e-18	0.056674636	8.37536296145787e-18	0.050860032	4.24180284185145e-19	0	0.01321144	3.60774513770241e-18	1.35525271560688e-20	2.07916233170023e-18	0.10986294	0	0.012696468	0.00500803	0	0.017842506	0.061327165	0.079169671
1.28124845405472e-17	5.87583171982813e-18	0.062877142	0	0.082437555	2.08000951202951e-18	0	0.041982472	9.71785894129016e-18	0	3.79048349464767e-20	0.104201244	0	0.002626028	0	0	0.003560542	0.042295117	0.045855659
0	0	0.080123014	1.39279689150011e-17	0.082089376	0	4.17006622060597e-18	0.04080717	1.04746462897981e-17	0	0	0.096563423	0	0.01100262	0	1.2010886318626e-18	0.007187551	0.055418861	0.062606412
5.00792704386239e-18	0	0.092129318	0	0	0	1.75888990376004e-18	0.006690357	3.18866367345263e-19	0.000384541	2.11190683070852e-18	0.216166365	5.7967239618988e-18	0.02864418	0	6.16661423885175e-19	9.30180226967208e-18	0.027038666	0.027038666
7.19415622689179e-19	0.03288485	0.059990508	2.86550862303683e-18	4.47496468197112e-18	0.002750239	3.64014753335302e-19	0.003572222	0	0.001069875	0	0.199971695	1.21250407899449e-17	0.067856441	9.32390917037174e-18	3.98354660929114e-20	0.000618544	0.029849957	0.030468501
0	0.001267332	0.063472682	4.01581847475891e-18	0.036822521	0.001801394	0	0.034517289	3.07166903014867e-18	0	5.35301445503635e-19	0.154432474	6.18727077347864e-18	0.031527346	3.08497121493421e-18	0.005146662	0.001966146	0.036800037	0.038766183
1.30127992597619e-17	0.01367302	0.050507914	4.34948384094813e-18	0.028647182	2.78102721668285e-19	0	3.896609317259e-19	0	0.003871926	0	0.18157314	0	0.038917367	6.18384968611465e-18	6.99370124286038e-19	9.67554567837909e-18	0.052855554	0.052855554
2.57368198404112e-18	0.000484488	0.086910783	0	0.059492887	1.34639516694065e-20	0.000689474	0.028741473	0	1.49457198621752e-21	0	0.11387298	3.50884828967839e-19	0.03299212	0.013794188	0.003803406	0.0028616	0.044371209	0.047232808
2.11813299852437e-18	0	0.022579347	3.36563302929443e-18	2.12077151858544e-17	0	0	0.007910021	5.07864851305841e-19	0.000311881	5.80677947849729e-22	0.181770313	6.2785019992963e-18	0.0426029	4.00923307199471e-18	0	9.20024398455312e-18	0.025369785	0.025369785
0	2.97263253247983e-19	0.073086526	8.55130782544628e-18	0.030189278	0	0	0.032124292	0	0	0	0.144660731	9.93129037374447e-19	0.009515961	3.33013355001019e-20	0	0.017781762	0.042928587	0.06071035
1.1838315888074e-17	0	0.075576994	1.50625104884322e-17	0.041631452	0	0	0.052880556	8.05112499029348e-18	0	0	0.143355043	0	0.007048644	0	0	0.004528367	0.056523803	0.06105217
9.76687301310332e-18	3.05482246782508e-18	0.086402572	0	0.043639966	0	0	0.021967113	3.19875277262768e-17	0	7.1325984940389e-19	0.12140864	4.39194340031344e-18	0.016616595	0	0	0.001265956	0.035858102	0.037124058
4.91921617181424e-18	2.50564128768811e-18	0.075099047	4.41280420767607e-18	0.029758568	1.47300666755247e-18	6.70061704374559e-21	0.04880301	1.58902426322945e-17	0	0	0.124224448	2.67783138488921e-18	0.018052957	0	8.53643943594942e-20	0.00882537	0.051707761	0.06053313
0	1.27029452046768e-17	0.056066763	0	0.065193619	0	4.62720299317378e-19	0.013461349	2.64131300138897e-17	5.21209023622921e-21	1.68209513758357e-18	0.133441576	4.17034787622018e-18	0.007758924	7.3610411093096e-19	2.87895993798569e-18	0.00950636	0.042503786	0.052010146
0	0	0.078200107	0	0.045719742	0	0	0.019376313	9.21858216475824e-18	0	6.14515508277142e-18	0.128816666	1.18930732708543e-18	0.008114838	0.005498581	0	0.006424812	0.038661466	0.045086279
5.84445628917104e-18	7.23173421492339e-18	0.080725424	0	0.051403436	3.94082985189129e-18	0	0.057597728	0	0	4.05173369209767e-18	0.11551557	2.95792939894856e-18	2.83485197822635e-22	0	0	0.016133003	0.048668461	0.064801464
2.48210631818972e-18	0	0.061079497	0	0.089841384	0	0	0.026282554	3.05121177445735e-18	5.08228780978076e-20	2.91144038645539e-18	0.130282534	4.77035944193411e-19	0.010122369	0.002170854	0.004894766	0.038159616	0.039094075	0.077253691
0	5.20129308562957e-19	0.058907235	2.89998025499572e-18	0.075300291	0	1.89278485779567e-18	0.02622139	5.08274288184273e-18	2.71050543121376e-20	0	0.154461226	6.14015795394793e-19	0.006083907	0	0.002607416	0.006182236	0.053437886	0.059620121
0	2.92164253015303e-19	0.063066498	6.98337174213127e-18	0.054757482	0	0	0.032585583	0	0	0	0.117855332	0	0.013067333	0.006839381	0.003284802	0.060209217	0.049167406	0.109376623
0	1.0069350018679e-17	0.052403966	0	0.054357903	9.24283990453504e-19	9.18578448208138e-19	0.058026971	2.14066552093929e-18	0	6.17929143822891e-19	0.036134301	1.173677687356e-19	0.007060682	2.67444351065947e-20	4.4697078848779e-21	0.112934082	0.049919884	0.162853967
1.88449611127495e-18	0.025208872	0.046552731	2.38347456424768e-18	0.096885745	0.014963705	0.003327073	0.012956017	0	3.00131331368736e-05	8.7243395478057e-21	0.095160957	0	0.051785548	0.005814004	0	0.050183959	0.055661039	0.105844998
8.93243180971348e-18	0.026306979	0.084756836	9.84446542254852e-18	0	0.010070572	1.80300847055427e-18	0.021703697	8.85498828495717e-18	0.000728203	1.40650096775634e-19	0.157659429	0	0.030226247	0.022628939	3.59606865506203e-18	0.004729222	0.027148869	0.031878091
3.09924535675653e-18	0.009998304	0.058872199	0	0.021084019	0.000565423	0.002964438	0.018313534	4.6267943529658e-18	4.45819886334679e-20	9.09183507769194e-19	0.102352961	0	0.016309037	0.006542562	0.016579816	0.036226228	0.043844015	0.080070243
0	7.03652206933348e-19	0.064214314	1.03477822060567e-17	0.070955611	4.5793042601461e-20	0	0.01664722	2.44877588297514e-17	0	0	0.13634417	0	0.036307681	0	0	0.006264523	0.054271687	0.060536209
1.43646724234385e-19	0	0.088820295	3.24788576878089e-18	0.07489579	0.004401102	0	0.004125398	0	0	8.25749438585319e-20	0.130963559	0	0.030569209	0	2.07534941139997e-18	3.77366621880411e-18	0.069004235	0.069004235
4.21004983341104e-18	0.065789849	0.044192029	0	3.56788313964218e-18	0.002735024	2.88948678918079e-20	2.10598416171713e-18	0	0.001677919	0	0.109341992	2.72709578578275e-18	0.035028918	0.000323254	0.003612033	0.003074618	0.019369955	0.022444573
0	0	0.063296971	1.67449240414439e-18	0.070643772	0.001922248	0.001638689	0.021312224	0	0	0	0.148746027	0	0.0122763	3.76983161158048e-19	4.51143694007379e-20	0.019222989	0.077259494	0.096482483
2.55986398907737e-18	0	0.07198431	0	0.062417241	1.81694690379473e-18	5.56545722248027e-18	0.019455203	1.36331987488011e-17	9.77052574813367e-05	2.9999543170987e-19	0.184566805	0	0.019290713	0	0	8.86845183747254e-18	0.073268303	0.073268303
0	3.58678630069565e-18	0.07884957	1.28160290572654e-17	0.061884412	2.59228761069016e-20	0	0.025847132	0	0.000267196	0	0.204902563	2.65689801923491e-18	0.017767826	0.008129441	0	0.006012669	0.049736607	0.055749276
0	0	0.063922514	0	0.072689176	0.000942025	3.38043954609849e-19	0.016789079	0	0.000320087	2.02321661671491e-18	0.185275329	0	0.016684565	0.012696806	0	0.006064109	0.045383337	0.051447446
0	0.057535136	0.04031648	4.17392381777108e-18	0.000517774	0.00428429	0	4.66124492379475e-18	7.52342363761362e-19	0.001246428	0.001723366	0.117951237	1.28527060766463e-17	0.06378197	1.98975979505274e-18	2.16784961789463e-18	1.09216249259391e-17	0.043283045	0.043283045
0	0.016307734	0.041165934	4.92057283167865e-18	1.64371272337003e-18	0	0	0	0	2.84912321161277e-19	0	0.179319665	4.84829256549559e-18	0.048515385	8.13952063087877e-19	0	1.28322090370568e-18	0.042070101	0.042070101
0	4.90123756454218e-20	0.069416923	0	0.050001832	0.003737711	0.004317766	0	8.45542820481904e-18	0.000119333	0	0.180232533	5.7088552285736e-20	0.031081517	0.004066918	1.00934088065296e-18	0.004463949	0.049922774	0.049922774
0	0	0.080329985	0	0.054476902	3.63093902779155e-19	0	0.010084644	0	0	3.20481713850013e-18	0.187770301	0	0.01338326	0.003052666	0.001959615	0.004126721	0.045294595	0.049421317
1.10950438389803e-17	0.070332162	0.033497854	1.34348617526847e-17	0.02467402	0.000965735	0	0	0	0	3.25425397646085e-18	0.199884575	9.81071744745917e-18	0.059503893	0	1.29475957908589e-18	1.77694919307165e-17	0.039629385	0.039629385
8.43833498755171e-18	2.55791397647497e-19	0.084633872	0	0.009295624	0.001059174	5.51638518013194e-22	0.010338519	9.34094486499476e-18	0	2.50435050392978e-18	0.187809202	0	0.033013325	0.018667333	1.26918923960653e-18	0.007603814	0.040525676	0.04812949
2.40122701796049e-18	0	0.073971074	1.15712255168234e-17	0.023538598	5.39329111299362e-21	0	0.002660105	0	3.391743659605e-20	0	0.220363455	3.44366879107376e-18	0.025573123	0	0	0.000308167	0.040624236	0.040932403
2.15464559619117e-18	2.15266269253572e-18	0.078126737	2.01424078437845e-18	0.010023696	5.41928666611975e-19	0	0.009416106	0	0.000179002	1.62169038127679e-18	0.179166146	0	0.020757151	0.006773433	3.69082347913658e-18	0.000185443	0.040807854	0.040993297
0	0	0.051294909	0	0.031367809	0	0	0.023043706	1.22440126792488e-18	0	4.65753276706037e-20	0.139897953	9.59081764083104e-19	0.021973549	2.30495300831778e-22	0	3.005159992242e-18	0.058324653	0.058324653
1.03484495261135e-18	4.4581670831125e-21	0.073452118	4.10428772100144e-18	0.070942239	0	0	0.004738842	0	8.26708284758099e-05	0	0.114755253	0	0.013463161	0.004120716	0	0.01003159	0.067676616	0.077708206
2.37414237347497e-18	0.016599167	0.036465821	0	0.039429829	6.66086143156916e-19	4.74818750595398e-19	0.016049441	0	2.37028047106271e-20	0	0.126341387	1.14347736441548e-18	0	0.050064738	0.005011984	0.108047649	0.053042833	0.161090483
0	2.4665753541813e-19	0.06528698	0	0.04030233	4.59076386107857e-19	1.9132971050215e-18	0.024820966	2.58948540308492e-18	0	6.30682355348353e-20	0.13895919	0	0.021399936	5.34866654421818e-19	7.7530055739418e-19	0.000780789	0.040220839	0.041001628
0	0	0.062985312	0	0.050988624	4.23433193317236e-18	0	0.008975797	1.61332556881265e-17	0	9.75109566556142e-19	0.106698982	6.74411557569821e-18	0.02884638	7.66554165418539e-21	0	0.012478072	0.04705482	0.059532893
0	0	0.049075724	4.48279670089816e-18	0.023153033	0	7.045831562439e-19	0	0	0	2.856372095077e-19	0.20939393	5.58759069649331e-18	0.044112705	0	3.40270356461808e-18	0.006139329	0.045205345	0.051344675
0	0	0.063410591	0	0.019178192	3.26691583488623e-21	0.001451497	0	0	0	0	0.1546035	0	0.032467163	0.00078988	0	4.67348897342618e-18	0.036092422	0.036092422
5.37588048886418e-18	0.039420046	0.097914518	3.53992188411536e-19	0	0.001866762	1.04816453742797e-19	0.001660142	1.98540232428787e-17	3.04516477658343e-20	0	0.236127136	1.80393132799124e-18	0.043121046	0.009388458	0	0.001552622	0.026777916	0.028330537
3.68739089568246e-18	0.023723283	0.091303455	7.53387696515475e-19	0	0.004753578	0.002258508	0.005897289	5.99661843972095e-18	0	0	0.20270069	3.16189885885033e-18	0.052693647	1.95248879258574e-18	0	7.89719276040043e-05	0.035811665	0.035890637
0	0	0.057335736	0	0.082739234	0	0	0.017128056	0	6.04645829432352e-21	0	0.114186968	3.02245391835119e-19	0.015819934	0.007146489	1.16972818716888e-20	0.026129635	0.045682838	0.071812473
1.02435036964727e-18	3.50167719151835e-19	0.078868748	0	0.068205273	0	0	0.029883369	1.04857806554474e-17	4.16136902745376e-20	0	0.198499227	0	0.022012735	0.001823294	0	5.04321472458858e-18	0.043132717	0.043132717
8.51553540386656e-18	0.041662424	0.012759731	0	0.020705132	0.002949917	0	0	0	0.000181632	1.14153421904781e-18	0.168242335	7.13012105759646e-18	0.049006824	0	0	0.001107368	0.046252556	0.047359924
1.39028797827065e-17	0.030047683	0.071611845	0	0.003042925	8.54717577741169e-19	8.01053916385027e-20	6.08375576247126e-18	9.27090725586662e-18	0	7.84683889927759e-19	0.216233503	0	0.035038252	0.007920883	2.23678681153083e-18	0.001707498	0.032758262	0.03446576
2.72848020478649e-18	2.60014013278736e-19	0.064540367	4.92504120873486e-18	0.032064564	0	5.10970722530767e-19	0	2.18786961097346e-17	0.003735009	3.14167142512009e-18	0.154910984	1.29340027186596e-17	0.012968062	0.006123749	0	0.006528702	0.036144009	0.042672711
5.78922809928468e-19	0.007641868	0.078125425	0	0.049994869	0.003875345	0	0.038406933	0	7.09440275438703e-20	1.04744072886264e-18	0.143618816	0	0.006975011	0.012464784	6.42591998093966e-20	0.039619893	0.052562177	0.09218207
1.41567663643044e-17	0.012255911	0.049135707	1.51984131762657e-17	0.103106776	0	4.68494008981506e-18	0.010932649	0	6.78906331006609e-20	2.44986916019295e-20	0.113413394	6.15724022849852e-18	0.016398543	0.007371261	7.01356822220987e-19	0.020402166	0.077821487	0.098223652
0	5.98545274132292e-19	0.069063046	0	0.07037258	0	9.22880807663816e-18	0.028972485	1.18314505161771e-17	0	1.39448289450828e-18	0.188383785	7.35141502138187e-18	2.42581974054106e-20	0	8.54234096802548e-18	0.000996338	0.064275237	0.065271575
0	0	0.074095643	2.57412223640129e-18	0.071850365	7.59260037927773e-19	0	1.913828876496e-20	2.27665305704114e-17	0	0	0.175973087	0	0.013749695	0.005462671	0.000149915	0.014801241	0.050303392	0.065104633
2.73111335012187e-18	0.001149121	0.067167144	0	0.071868146	3.58147994080596e-20	0.000355576	0.026832837	8.8672638633532e-18	0	0.000528092	0.120778872	0	0.019913343	0	1.35525271560688e-20	0.01930084	0.050348103	0.069648944
0	0.002403173	0.074796126	3.44438069552906e-18	0.065162001	2.23831988691804e-20	7.45438567113337e-19	0.033150941	0	1.7391960393665e-20	0	0.113672026	8.71880316534488e-19	0.024663106	1.64003309576679e-18	0	0.001367995	0.061404003	0.062771998
0	0	0.04789285	2.89789440515287e-18	0.086558201	0	9.40088794551547e-19	0.035302957	0	1.42219115376771e-21	0.001150498	0.072985743	0	0.015854909	0	0.000469823	0.00827181	0.06313814	0.071409951
5.91890190115189e-18	0.002886376	0.042506326	0	0.067408811	0.0004404	1.16173592367307e-19	0.019859807	0	0	9.41664279889663e-21	0.05308326	0	0.00237742	1.24631935477545e-18	0.01463524	0.047774858	0.058740113	0.106514971
9.85471084108062e-19	0.00311045	0.073644299	0	0.075539641	0	0.003841654	0.020167213	0	0	1.2692120294902e-18	0.078891028	0	0.018790367	0	0.001182802	0.014285605	0.065002769	0.079288374
0	0.00364458	0.040984573	0	1.67042182630328e-17	0.004026207	0.003976285	3.01392797975167e-18	0	2.07387217030298e-20	9.45975150518255e-19	0.200532801	5.37275603551965e-18	0.054777172	0.000349893	3.89733515157671e-18	0.015470011	0.040947902	0.056417913
3.13001909856188e-18	0.016641197	0.046282275	0	8.2183299332921e-19	0.000911407	8.87952903456622e-20	2.72416674052431e-18	1.59163034731905e-17	0.000874953	2.22838795579361e-20	0.120969277	4.05583835214249e-18	0.048857123	4.12890337155696e-18	1.15812222933811e-18	4.58136716178747e-18	0.041575558	0.041575558
0	0.069057229	0	0	0.023969191	0.00647254	2.87436112186068e-19	0	0.008910723	0	0.009555144	0.05395437	0	0.084327465	0	7.00978170137219e-19	0.043844647	0.049381757	0.093226404
1.02959407038351e-17	1.89121119325076e-19	0.064256968	8.64755633475562e-18	0.029988676	1.08181855249353e-19	4.32056160355978e-18	0.031911553	5.38603108062684e-18	0.000254924	0.001139559	0.135206912	1.04774082776983e-17	0.051692165	4.72294371292157e-18	2.53221057479647e-18	7.25418573891991e-18	0.031409788	0.031409788
3.38057598361003e-18	0.03692086	0.022542056	0	0	0.002481077	0	0.000490056	0	0	0.00868794	0.010072216	0	0.0754503	1.73726334528367e-17	3.27831784813712e-18	7.62098673929236e-18	0.031125654	0.031125654
0	0.036726742	0.028389094	1.82981952329872e-18	5.39057370901703e-18	3.66741553711304e-20	1.0888005823901e-18	0.001942049	8.40521163078757e-18	0.000690449	0.003975107	0.154840869	2.20793647581727e-18	0.064887463	0	1.12145876998919e-18	0.003389978	0.036449754	0.039839732
0	0	0.087918717	5.53002174642053e-18	0.06719284	0.000577863	6.32338421216409e-20	0.037878417	0	0	1.01687366740405e-18	0.094794927	0	0.028542787	7.3194608201542e-20	0	0.014090716	0.061691179	0.075781895
9.54479046449402e-19	0	0.081925471	8.15746665039254e-18	0.011182998	0	0	0	0	0	3.2052386309666e-19	0.140582505	1.85703299276499e-18	0.033168636	0.013549585	0.001512198	2.88350045233238e-18	0.042589023	0.042589023
0	0.033235028	0	1.30362780532196e-18	4.2329154756823e-18	0.012883338	0.001677486	0	0.006690843	5.01580885802565e-22	4.66472805309938e-05	0.028760588	3.07334560172377e-18	0.032509279	0	0.017130911	0.087402909	0.028346114	0.115749023
0	0	0.0620091	1.39618229088358e-17	0.078502329	0	0	0	1.04040510593759e-17	1.91271509511293e-05	1.85531699707916e-18	0.107737172	2.2826517326433e-18	0.03483709	2.37599693450789e-18	3.76843525577541e-18	0.014545497	0.075510922	0.09005642
2.05069839062553e-18	0.01536776	0.077653935	4.14220382352572e-19	8.99234142910602e-18	0.009195494	0.000888709	4.54711856154808e-18	0.001299895	0.000580799	0	0.171821779	3.65900387097904e-18	0.051799678	0.000556892	0	0.015190652	0.041035966	0.056226618
0	1.95864565316873e-20	0.075442124	0	0.025807128	0	0.005430739	0.033838834	0	0	1.52076575332067e-19	0.125899262	0	0.01791346	0.014129777	0	0.005721695	0.055015002	0.060736696
1.09800675210481e-17	0	0.048472685	0	0.070242988	8.09993485456632e-19	0	0.029783391	0	2.77598680010456e-20	1.09206815205263e-20	0.099309491	7.50205339424395e-19	0.011429293	1.60311754282084e-18	0.009654794	0.014030237	0.048139456	0.062169693
2.14087471872891e-18	0.042797725	0	1.05439292117066e-19	0.017314411	0.007013492	0	1.91619361841801e-18	0	0	0	0	0	0.038064424	0	0	0.006777504	0.028586571	0.035364075
0	0.03721447	0.057220856	6.25927902732853e-18	0.030653946	5.18214362403938e-22	0	0	0	0.001880203	0.00126745	0.075334024	0	0.043321914	0.01439495	0	0.00995477	0.042025381	0.051980152
1.64446112051197e-18	0.000845697	0.043740043	0	0.079316604	0.001995878	4.15213434383963e-20	0.03353044	4.10814341656418e-18	0	2.7427538082162e-20	0.102460636	9.80199367270777e-19	0.006331323	1.02006937232326e-18	0.009439524	0.049786833	0.061747378	0.111534211

Supplementary Table S8. Modeling input matrix based on signature proteins of S1.

Sample	Recurrence	TTR_day	ABCF2	ARID1A	BOP1	BRIX1	CBFB	COG2	DBN1	DDX24	DHX8	ENO3	EPDR1
P101	0	156	17.59526801	15.84512184	16.83357337	17.03691116	20.37047662	17.88095106	19.29551525	0	0	26.50516682	19.60362983
P102	0	1481	14.67303099	0	17.71603274	16.82467414	16.65355613	16.50234264	18.91860251	14.93725542	0	25.2323057	18.57772222
P103	1	536	0	13.23836302	18.11385377	0	18.97929898	16.21107991	19.86518208	14.11075105	15.11143778	26.34511326	0
P104	1	102	16.33137398	18.09868205	16.92280142	15.49021486	20.36300601	17.25606166	19.74418968	12.91391695	0	25.95285014	20.31344708
P105	1	69	18.37299652	17.22511474	17.85993109	18.5505972	19.85842201	18.84209304	19.88911606	21.10423182	16.50652251	24.72576019	21.23185262
P106	1	284	0	16.73586518	16.4301465	15.46063612	16.53251009	17.0877982	19.37663851	15.17455677	0	26.97719508	19.8864231
P108	1	249	18.7797079	17.35333698	17.9687775	17.85382512	20.85085247	19.50539781	18.95208755	16.90084013	13.92658653	24.19045552	21.35841536
P110	1	484	16.99147614	15.94765939	14.854726	0	20.16791038	17.10698251	17.74815594	16.18409891	14.04516893	28.04157692	19.81006218
P117	1	1073	18.47142834	16.22268381	15.16292755	0	20.53905441	17.60747685	18.64927085	14.17402467	14.12357454	27.50765711	0
P118	0	1448	17.64877192	18.04352578	19.46828651	18.09866656	21.20312951	18.99759352	20.80202282	16.66739066	16.0030414	25.36586833	22.0443058
P119	1	296	15.72083766	0	17.7972687	17.72407076	19.53302067	0	17.02571373	0	0	26.7822817	16.32614144
P12	1	187	0	16.35807733	18.06767462	0	20.09422972	18.88956357	19.8976084	16.40389811	15.65172814	24.7654872	18.4444104
P121	1	381	17.98035787	18.39636274	17.45581263	18.03283889	21.83969085	19.08243757	20.93252509	15.68968215	16.83250794	24.20522529	22.78413644
P123	1	42	18.22073097	19.05519173	18.3295913	19.31302816	22.04518312	18.70299085	22.61662807	17.71985234	18.08686019	25.27130432	21.65382893
P126	1	636	18.20436831	18.64489925	19.70905117	18.67046831	21.0178407	18.08638648	21.60131288	17.32209664	16.54660656	25.11026342	21.57791435
P129	1	469	15.61954189	15.52410032	18.66205061	17.72317464	18.29030248	16.70369428	19.07786917	0	14.81212686	26.35664537	18.44725471
P130	1	268	15.9035004	0	17.5616895	18.66073649	19.75712797	18.53968583	19.63843469	0	0	27.03595404	20.57210313
P131	1	627	17.53608272	13.81872854	16.455292	18.28059296	19.63914359	17.19416416	21.19084202	12.80471948	15.20330222	26.17091006	22.1213207
P134	1	9	18.35629316	18.36862251	18.78106286	18.39029298	21.0209859	19.74112133	21.82619481	17.47753905	15.78218278	22.86285296	22.53976842
P136	0	63	16.78289014	16.33418345	18.04075188	0	20.38995711	18.89333639	21.12411452	0	0	25.95476485	17.43508654
P140	0	1369	0	14.53654102	18.47521616	0	20.45640512	17.51219114	20.76766672	0	0	25.31263467	16.00344227
P147	1	353	21.78891155	19.215786	21.33640255	22.33358652	22.61211044	21.45951988	23.65667183	19.1166447	18.95663398	17.06718808	21.66948959
P148	0	1356	14.31787928	13.37163487	15.18825821	0	0	0	19.65741012	0	14.98102789	26.52777228	20.37248093
P150	1	221	0	15.59084333	17.79566179	0	19.13434777	16.60120134	19.44501828	0	0	26.50053038	21.22183112
P152	1	1162	14.47650777	15.79379227	0	17.91642341	18.38409421	16.24175856	18.98103128	13.28818596	12.95523438	26.92557085	20.00429596
P153	0	1337	16.72764826	17.48759121	17.8613983	16.27504068	20.44469625	18.61251183	19.01156991	16.22150079	16.06698065	27.38993402	19.27296523
P154	1	105	16.83826951	16.04071674	19.42340129	18.39484193	19.25454649	15.46150678	18.62013764	16.17040283	16.69329187	27.35805972	21.3693461
P156	1	42	19.1119836	16.91135045	19.77486686	18.97707345	20.05229366	19.0246714	18.99622078	0	12.31635283	25.32373963	19.46241813
P158	1	254	17.81419787	15.22026708	19.02800631	16.44588786	18.96868404	18.8395653	20.1627264	15.18602084	16.73820208	25.07922984	20.33851762
P159	1	324	18.71637907	15.3259126	19.19385876	14.80768697	19.54136493	17.81814673	19.29999558	0	0	25.39960167	20.75823676
P162	1	775	0	17.80787077	18.48600733	0	19.22898436	14.12933428	19.94753761	0	0	26.64644179	16.46076936
P164	1	221	0	17.68606938	16.61664236	18.58387594	20.31536064	17.80659558	21.62708131	15.67589792	16.44084753	22.04508945	21.12777455
P166	1	46	20.37407241	17.78727441	20.62218763	20.91338088	22.56887274	20.89565622	24.53624942	17.44079312	15.17280974	0	23.74152432
P167	1	198	16.59054999	16.47449385	18.02178441	16.8019991	20.16659164	17.0158366	17.76646606	15.2352053	16.63863451	27.05124874	17.81909242
P173	1	66	17.96806312	18.25658115	19.14544468	19.15539991	20.45891341	17.79232558	19.21354684	20.35499744	16.0420655	24.47586907	21.22490892
P176	0	1280	14.22840036	0	17.71349531	16.37980344	19.08153052	18.11903886	18.44932208	0	0	26.64279235	0
P177	0	1281	14.95716886	17.74007923	17.73816732	16.01235632	19.688547	18.22778936	18.44902855	0	14.38378008	27.54267654	19.48394677
P179	1	88	17.70130313	15.05460819	17.83060196	17.20370054	20.7277338	17.27238979	20.43925029	15.01138238	0	22.78466124	22.17775447
P181	1	194	16.99737321	16.39773467	16.17918217	18.52849132	19.53528816	16.5002271	19.07165147	16.25649824	13.30927069	26.86605129	19.70403725
P182	1	417	16.57356432	15.71158321	17.23872448	0	17.42704473	0	20.26247341	16.70172641	0	26.52737174	20.95132652
P184	0	1261	16.63266542	14.54579288	18.32161437	0	20.53554971	16.36405812	18.61709694	16.13159523	16.8390537	25.59540583	20.47452432
P187	1	857	17.510638	17.17612094	17.08122864	18.39525763	19.50979061	17.7404674	19.91885842	16.60758035	14.54793699	25.62295543	21.0378241
P19.136	1	777	18.07407737	18.00310871	19.82588971	17.988833	22.22321439	19.39201721	20.92106328	16.78565854	16.0803041	24.82250621	17.1354274
P19.140	1	804	17.23912336	16.32244568	18.85595014	17.52546321	20.55749714	18.12997999	19.58718371	14.39629654	15.32297314	23.98772417	21.11019212
P19.144	0	1317	0	16.81331302	16.25437294	16.24814928	19.29265772	17.26246302	18.71021948	0	0	26.74837159	17.99832987
P19.17	1	969	15.59428141	15.58183584	17.0757825	18.67699697	18.61495303	16.23505076	17.54796818	16.45577932	0	26.63576883	21.22248286
P19.177	0	1295	16.6931411	16.98939023	18.94325013	18.60921683	20.20789064	17.53926865	19.88979629	15.80313254	15.07532455	26.58809059	18.13797295
P19.206	1	127	18.7763414	15.63969796	16.64408567	17.54798525	20.49887317	17.8923327	20.49741862	15.79030858	0	26.59495955	20.52281044
P19.217	1	1038	14.81710007	16.98187307	15.66335414	0	20.59629297	16.30059095	19.22701524	0	0	27.3984128	17.83693591
P19.220	1	887	16.25748141	15.08868932	0	16.49488838	18.28446187	16.5725866	19.81900203	0	14.97956673	26.87258166	0
P19.3	1	181	0	13.61269242	0	17.74336598	0	0	0	12.93397413	15.18994304	26.2406279	0
P19.325	0	1258	16.0791383	16.37116203	17.0287478	17.18555682	19.45344359	17.96077764	18.29538396	16.59489442	14.28037096	25.12601238	19.33809456
P19.413	1	206	17.40135724	16.22413802	16.03089647	18.03387937	17.93849988	17.48903639	20.19484322	14.64844993	13.3906429	26.69374257	18.56737371
P19.511	0	1216	17.38545962	0	16.67274765	14.96922054	18.12757205	16.0553367	19.19413004	15.53940865	0	25.62679222	19.75196756
P19.557	0	1203	16.79296141	15.68681316	16.69335019	18.22411362	17.61945973	0	18.40372545	0	14.76096951	27.04978531	18.1329201
P19.603	0	1196	18.64363638	16.64512929	17.50383698	18.88046292	18.47963896	0	16.34597575	15.12672251	14.26622519	25.855774	20.43574269
P19.607	0	1189	16.3321063	16.47556309	17.35561541	18.33744559	19.66418525	17.00410276	16.85032167	0	0	25.82670327	18.45921959
P19.608	0	1188	17.14858975	16.05869374	17.75866984	18.79476491	19.53751621	14.03654001	16.48694155	0	0	25.649589	20.30628366
P19.62	1	1260	0	16.15036186	18.29297832	0	20.14175018	0	20.10171459	17.0468924	13.15686207	25.24636917	20.9793199
P19.642	1	53	17.36206989	16.77283755	18.43583772	19.5877645	20.11552643	18.06139573	19.78666204	15.93248585	14.33975466	25.49358233	21.44519603
P19.649	1	406	17.48905377	17.32123119	17.43365262	18.581798	0	16.13599724	0	0	16.21295143	26.64686781	17.15679734
P19.689	0	1159	0	16.50586548	16.58836626	16.61534537	0	17.8269936	20.6890075	15.48811258	0	25.15329311	17.68673053
P19.699	0	1162	15.42190546	13.39339885	0	17.48437508	18.21100679	15.98446022	16.3605158	15.89376229	0	25.10698099	20.12416684
P19.705	0	943	17.75064658	16.89969377	15.07092764	18.68546404	20.66034042	16.59580268	21.45051759	16.09216956	0	25.06379332	21.96981578
P19.752	1	420	16.58761885	14.20612849	18.15203639	0	0	16.04160359	0	15.54286922	0	26.35431969	20.25422442
P19.793	1	490	18.0901857	16.51069764	17.93593942	18.83540455	19.34264346	18.22357484	19.29805662	16.80458738	0	25.96869724	20.44797956
P19.85	1	572	16.16125791	15.3573854	19.47918293	18.86854748	20.40912272	17.51642375	19.27419301	13.57036531	0	27.472786	20.7608208
P19.873	1	418	16.0610677	17.27612817	0	17.1699572	18.89459724	15.76332487	0	0	0	26.83472131	18.02030242
P19.893	0	95	17.3819165	15.71349204	17.78706639	14.96739894	19.02989369	16.59445725	0	0	14.16428949	26.93280463	20.19663504
P19.98	0	1352	0	16.66116935	18.42919591	0	0	16.98874149	19.64418696	0	0	25.30642673	20.15301132
P190	1	165	0	16.48489845	15.74217084	17.45799958	18.1923977	17.46469955	17.5811815	0	0	25.32702207	20.28215742
P191	1	481	0	0	18.27185187	0	17.88853552	0	18.4294347	0	0	26.39641813	0
P192	0	1238	0	15.54164849	17.43258948	16.78179312	19.63528751	17.41847658	18.89119948	11.64263021	0	26.24141991	16.34141279
P193	0	1238	0	15.96828507	17.98778677	18.21423561	20.9777501	17.21119361	19.34110295	0	0	25.58443715	16.2006952
P195	1	433	14.55888073	15.60024018	0	18.15831561	17.15336075	0	16.52871407	13.17762974	12.13332752	26.30430897	20.22224355
P196	0	1236	15.99694796	15.5432924	17.75236717	16.56246415	0	16.45745996	15.22345119	0	0	26.24974207	0
P197	1	806	0	13.36041213	16.91002789	15.16702061	0	0	20.29931476	14.94042212	0	24.58636765	20.24368211
P198	1	490	19.02048205	17.12652003	18.5502712	18.98779461	22.03707665	20.76149961	23.65205154	16.6210526	17.15085569	22.02659057	22.5392909
P2	0	608	18.19116917	16.11555898	18.09605976	16.8696736	19.7375856	17.03556862	18.88734733	14.91223533	15.09916392	24.77591599	18.46393826
P20.31	0	1064	0	14.85514642	16.17419179	19.27665905	19.42060026	17.59899125	18.15997014	14.41172378	12.97936523	27.69970521	20.51613529
P20.42	0	1062	15.86220666	13.73255618	0	16.7987962	19.46691346	17.29866826	18.61121651	13.65416197	0	26.7974098	20.45432662
P20.56	1	95	16.94548105	0	17.27354067	17.69056287	17.20096634	16.41514201	17.07723366	0	0	24.69494768	19.42507353
P200	1	331	18.44305717	15.341139	0	0	20.64548017	17.71150143	19.62812315	18.82605879	0	25.36320331	19.56809709
P203	0	1224	0	0	16.83440588	18.22102419	18.94273969	17.63327418	16.56132503	16.32926017	13.09945777	26.4727557	17.93425904
P209	1	18	0	14.86620577	17.60657153	0	19.27472966	16.59733775	18.49465388	0	0	26.65595044	18.4780321
P211	1	445	16.30552071	16.26842927	16.98756061	17.46072103	20.04104449	17.13520738	20.16782847	16.65385196	0	26.18963543	0
P214	1	412	17.78942216	17.21604108	15.14821498	20.3018005	19.73834466	18.31891908	22.60550781	15.85601554	13.75774434	25.56593452	20.1667277
P216	0	1118	0	15.74675269	16.70513036	16.65638061	18.27730974	18.8746932	20.04394483	12.40900453	14.40672641	26.44297291	19.88809982
P219	0	1107	18.12690171	16.85919913	19.72013572	20.94606207	19.993767	18.48955817	21.31224498	12.57465567	15.62676323	26.28738436	20.37578896
P220	1	308	17.02323267	16.58687238	15.53368204	0	20.28627539	17.97236725	19.25960167	12.99108011	14.28856726	25.98713551	18.45600325
P222	1	128	18.60180735	16.16207899	17.57477298	19.30900417	19.27283082	18.00696937	17.4839474	0	14.47071917	26.5848628	0
P224	0	1093	0	15.02388479	16.44503915	0	19.87425069	0	16.64134402	15.93724345	0	26.33030487	19.69235994
P225	0	1089	14.80428001	16.44797557	16.21211355	0	18.79453875	18.27393783	16.58988198	16.88975106	12.86044914	26.890209	17.16972916
P226	1	304	0	0	16.25054212	17.87073463	17.71266496	17.04634285	17.28321189	0	0	23.70044442	0
P231	0	1016	0	17.31404175	14.90073466	18.29398388	18.50446405	17.10350397	18.68567577	16.53568046	12.95631604	24.58173747	18.66604868
P232	1	31	19.15467427	0	17.8495131	17.02585392	19.24408535	17.11074377	18.94860449	0	15.16023076	26.80841575	18.01523963
P234	1	102	17.25632326	16.55885538	0	16.24327533	19.88586721	0	19.06873567	0	0	26.25069425	19.9881944
P235	1	216	18.35357645	16.64183689	0	17.34726646	0	17.73145488	19.36951301	0	13.53427487	27.26094707	20.0167521
P3	1	92	0	16.49623302	0	17.01562053	20.10034224	15.43160692	19.34714297	17.0134406	15.36193132	24.48096739	20.04280901
P4	0	61	15.90464278	15.1753541	0	17.21111642	19.00644637	0	18.85881554	0	0	26.32293592	18.73895825
P45	1	270	0	15.02388479	17.76239297	16.50938105	15.78766994	16.39240828	18.406786	15.57908896	11.42672042	25.64792628	0
P46	0	130	0	16.45846439	17.05101991	16.27074871	19.43513251	17.76639907	18.85017984	14.04881992	11.42441544	22.76931107	18.4374415
P51	1	543	0	0	0	0	17.03525602	0	17.15442691	0	0	27.23322166	20.08604048
P52	0	325	0	15.23150255	18.953887	18.12170566	19.51792566	17.89555935	19.22135131	13.22673642	0	25.03917635	0
P55	0	401	17.52762943	16.74404666	15.87681367	18.15650851	19.96475062	18.20604176	18.16031626	16.94791284	14.24854751	26.35243037	19.47353093
P57	1	494	18.76151811	16.9836248	17.8154381	19.04365288	19.28734232	15.73523105	18.54848365	0	14.92884759	25.62486439	0
P68	0	1575	17.30530248	16.0459217	17.63804888	19.29470543	19.95479186	15.65236463	20.21643217	18.00890476	15.9078536	26.89527025	20.89035848
P71	0	1573	15.71151484	15.50356826	16.44775478	18.29316193	0	17.59429615	18.55353913	16.96017018	13.67796824	26.17905761	19.07551931
P86	1	207	16.96613239	16.93038098	18.41158227	18.42609834	20.45450219	17.33006132	20.17987278	18.45523878	16.22381996	24.37975658	18.22246764
P89	1	621	16.97799906	13.35826736	14.56215542	0	0	17.59215235	18.09248894	16.69773365	15.69853161	26.68647815	20.48606527
P90	1	724	18.11618489	17.68433508	18.55780301	18.18665461	20.5586526	17.80995504	18.20206885	17.97817902	14.05085031	23.78054168	19.57631024
P93	1	232	15.42415526	15.89781938	16.58653774	18.73583954	20.29310828	17.01618104	20.40648041	16.62149736	15.86027463	26.61908404	20.06126523
P95	1	290	0	16.1542616	17.02671887	16.88721983	20.05165974	17.62824161	18.97184695	15.76896798	13.78619885	24.54522233	21.05003449
P97	1	604	0	15.43977365	17.14826826	17.42161874	20.37011166	17.2757855	15.69002347	14.62762358	0	26.77548263	21.08299754
P98	0	1499	15.43945218	16.67247876	18.78525161	17.96115276	19.67982543	18.4009637	20.68964447	16.58104393	0	25.52694045	21.00273892
P99	0	1488	0	0	0	18.87320292	0	0	18.59755917	14.68401438	13.75274634	27.02122693	21.69615723

EVI2B	G6PD	GMIP	GPNMB	HDAC2	HSPA13	IMPA2	LAMB1	LPCAT1	MANBA	MEAK7	MX2	NIPSNAP2	PDK3
18.54309405	21.81967536	15.84896988	22.69013745	17.51572984	18.3715685	18.02971465	18.09315167	0	14.7967557	0	15.78954462	20.40411178	16.39894443
0	21.15791647	0	19.76163536	17.17691133	18.64747241	0	18.42224469	17.53298664	0	0	0	0	17.53329206
0	21.27639243	0	19.73128231	0	17.91032677	18.09377775	19.55318844	0	16.72566015	13.06878577	16.88570246	18.21868673	18.82350192
18.59063272	21.8050319	15.32917827	17.73156855	20.58460345	18.97905717	19.0012034	18.48396048	17.23501161	0	17.97444657	15.31612499	20.7127321	18.16528541
18.43780594	21.9207389	15.39754507	20.49554291	20.15049559	20.38495342	23.29191966	18.90583533	18.79044714	19.33533825	17.66104085	16.83139473	16.85334265	18.39964656
0	21.23576659	16.07757124	20.39381666	18.73003483	17.69093093	18.99159194	15.71862266	0	16.11718604	0	0	17.94866946	17.47061366
17.9650476	21.5155234	17.32398483	18.75491141	21.87512155	20.0067995	18.73180974	17.48496593	17.33634366	19.45733637	18.00558442	0	17.32588142	20.15540796
19.42134647	22.55162408	0	19.54116441	17.27755928	18.13763004	20.68959625	14.53505873	18.94406455	16.92228877	17.00026679	16.84018936	19.06354632	17.41206065
17.40114437	22.13718357	0	20.65729754	17.44835595	16.1126104	16.07930801	17.09282423	0	17.84628247	0	0	18.81321095	18.99924436
17.88249364	22.85571248	15.9655862	22.42595716	19.12613072	19.83823538	19.26896645	20.68540696	18.03728593	19.13630626	17.29584889	15.08333063	21.20736773	19.37567258
0	20.84057475	15.58229258	19.24432027	17.95485678	18.13086249	0	18.15701826	0	17.60588363	0	0	19.12995875	17.3308382
0	22.05474126	0	19.31327746	0	19.74116286	19.6085004	19.40635141	16.7095563	16.66624357	15.90648398	15.96887274	19.5281571	16.94585375
18.96308544	23.13029396	17.90245332	22.05886777	22.18062372	19.78900508	19.4284305	20.4450114	18.38728402	18.80384795	18.00483185	0	21.80779637	18.1439778
20.2452103	24.14772549	18.57214168	22.81845969	22.60466158	19.79505396	18.12637731	22.27851847	17.87812697	18.45551718	19.78418323	17.98248287	21.10668127	19.55963794
18.78330591	23.25375408	18.19015349	21.50720983	20.54381602	19.79308095	19.91385384	19.75920372	17.91089261	16.38021477	18.47322383	17.68220877	21.48673296	17.62821856
0	21.93993996	0	21.42316337	19.94759917	18.36145099	16.17794677	18.30260492	0	15.46272217	19.20473351	0	19.69567945	18.64911859
18.30178399	21.96094287	14.88822419	20.57370769	18.42095951	18.69973921	0	20.35593084	18.70768326	17.48575644	0	14.71859852	19.69590847	17.84621946
19.14024433	22.72824622	13.98516938	21.80037825	16.9795757	18.90074949	18.36578089	20.42893526	17.11682893	17.67851638	18.50281047	14.68810386	20.66782281	17.94945402
20.38089489	22.90294994	15.40953145	19.73217749	19.04597134	20.0851931	21.94412447	19.90520004	16.65369681	19.97203451	17.95323509	15.69214946	20.75690684	19.80002469
19.76346319	22.76936153	16.65875159	24.84172329	18.61693663	19.18299046	0	20.10119631	18.38807557	18.45676773	17.32444434	15.62780913	19.30295593	18.82559686
19.1246871	20.78227811	0	20.10005664	17.1621853	17.99151666	0	19.29104564	0	0	15.98291037	17.64876424	16.82695363	16.37217566
21.03467488	25.55192912	19.15535966	23.20464244	23.71861647	19.51295153	19.85701066	23.5682219	22.71655703	20.02411821	20.95566783	19.76839177	22.74367105	18.78150566
18.69080542	20.64429211	0	19.94718372	0	17.96564803	17.98945144	18.06612777	0	0	0	14.50904096	17.2888709	18.3764935
17.46578229	21.79238337	0	19.54429956	17.36025519	17.72600945	18.36849064	16.47698021	15.86389634	17.64633136	17.39547649	14.69857491	19.5770093	19.12802082
16.47914001	21.32872244	0	0	19.48651659	17.59114228	19.01166966	17.64502309	15.79046034	17.19792396	18.24756887	13.97716975	20.09418706	0
17.75417052	21.21849098	16.09881355	21.08259561	20.73992764	19.2774068	23.21510702	17.74302589	17.41236746	18.57230181	16.11428575	16.9950655	18.31643959	17.18100916
19.07885323	21.66237281	16.87145483	20.32555095	20.87823807	18.62827414	15.33825055	17.00589192	18.22960016	17.23928523	18.39132205	0	19.01990004	18.91450049
19.75885873	21.64417898	0	0	18.96366699	19.51612283	0	16.73827932	0	18.72263753	18.94267174	16.17177742	18.83465865	20.45476067
18.23140782	22.15291218	15.41596248	0	18.91950526	18.50213574	0	20.46990041	17.2299186	17.0115948	17.72607917	14.70289045	21.40713665	18.61067941
17.49085501	22.89012536	16.02138559	20.99248003	18.57849394	19.78629544	0	18.76722085	18.5184195	18.1652844	17.48504173	18.69789639	21.22959269	17.39467438
17.69088377	20.77628708	15.0953137	20.22750638	20.0901619	0	0	18.35226917	0	15.380905	15.74692088	15.60959724	0	0
18.86236184	23.18079148	16.78074396	23.05052205	20.5840893	19.42991061	0	20.7838889	19.67860793	16.23988063	18.71307191	18.97382417	22.29470476	17.89815886
22.67069128	26.60783649	20.56875349	21.62514216	19.85365482	19.02429152	0	23.19379413	22.50452517	19.08915037	21.77567963	18.18445988	22.14192047	19.94583584
18.29902428	21.29050166	0	20.16081019	19.28318579	19.53199292	17.82840455	17.93461783	17.85822761	15.91308773	18.69359601	0	19.38842475	19.93558612
19.84429227	22.05251547	14.25940792	20.20910438	20.17572569	19.63072738	20.21884784	18.43962431	17.22952335	19.24484075	17.9629465	15.35030945	21.25783498	19.28957219
0	20.96849277	0	20.13172236	18.40215996	0	15.50631946	17.07018248	15.61927141	16.11974342	16.05718036	0	17.33047609	16.21277464
17.92581595	22.20269347	0	19.85354071	20.92941937	16.76600464	0	16.40151503	0	17.90086773	18.6747804	0	19.93717316	17.83758511
18.94568338	21.9525811	17.80010811	21.15380019	18.85864637	19.54952031	22.49644176	19.28254568	16.41274037	0	18.34474887	0	20.16766769	19.28881198
17.30492969	21.21389543	0	19.9431354	19.3223656	19.03557556	19.86883105	18.01452048	16.25942679	15.87587808	17.37166157	16.69607638	18.44074504	18.33429482
0	20.8762241	15.81199739	0	20.62396671	18.48312955	0	19.48779363	0	16.61434878	13.94664893	14.85946869	19.39233773	17.7917297
0	21.24140658	14.29359877	21.05482279	0	17.98276192	19.24437107	18.33960076	15.17024395	0	0	0	19.46157535	18.86545703
17.75581479	21.48716847	0	20.54352091	19.15194345	19.61368279	20.0994792	19.02885827	17.09436927	17.40575194	17.79901065	14.54069862	18.53721836	19.25989482
20.29302218	23.2391364	18.34963958	22.74720462	20.58209798	19.14057434	17.54781211	20.08739146	19.12388573	17.55152689	18.64433318	17.76553756	20.87069681	19.38496406
17.47992577	21.35381144	14.90443616	19.15938581	20.12854318	19.51712238	22.40198148	19.82001598	17.15828983	18.10916717	18.32215993	14.12382883	19.33594621	18.08740431
17.72286809	20.97775901	0	20.2178186	0	18.08602677	0	19.65156402	16.64185492	0	17.80459366	14.33732701	19.30828407	17.16875903
0	21.83391596	0	21.10397997	15.03075799	16.42395302	23.93952156	15.79956663	17.0374733	17.40469182	18.13134863	0	0	16.27609715
21.73636534	21.34181776	0	19.35124115	20.20646809	19.03722617	18.45236372	16.42398322	16.56687376	18.16923724	18.54376391	17.06475561	20.73466652	18.74881738
0	22.64897751	14.21495972	20.09541471	16.72379525	18.54672931	20.02042993	19.1850798	0	17.05820532	18.15540764	0	18.28193716	17.10490626
18.37429585	21.41917039	14.27115382	20.48066775	0	19.24669591	18.24440437	16.22642758	0	0	18.43856197	0	18.69317448	14.47909021
0	21.4126806	0	19.39389328	17.73606144	16.8931339	17.69969	19.53468074	0	16.52704449	17.79260332	14.50587694	19.14163594	18.29224164
0	19.72474626	13.24288683	18.10903493	0	17.54859632	0	14.51800608	0	15.98742705	15.62022344	0	0	18.48509223
17.15107426	21.62697265	16.17902512	0	20.06074099	18.31974083	17.34880218	17.2867593	16.60846416	17.33062915	16.47064023	0	17.76154438	16.48772465
17.38546165	21.57752984	0	0	17.58761915	17.89616922	0	15.40338269	0	0	15.29147938	0	19.82286466	16.68588917
18.33545234	23.01714815	0	0	19.16738287	0	17.8938297	17.54643746	16.50336853	16.38597678	16.28872794	0	19.34490916	16.84398471
0	21.75951935	0	19.81446351	17.53231628	17.50461459	17.82884481	15.30714628	0	16.86492754	0	0	20.01418985	18.76570286
17.62753356	21.70315761	0	20.48950157	20.48181702	18.01341923	0	19.23499486	16.03801297	16.3683339	17.8965704	0	17.46094429	0
18.31305333	21.71923113	14.02511755	21.89898868	0	18.73525064	18.98300726	19.1379096	17.80887384	17.97846838	19.09508219	0	17.65125236	17.40853682
0	21.16976813	0	0	17.61629271	16.80212112	0	16.57509463	17.129304	0	14.09859661	0	16.76309729	17.27938434
17.11413601	22.06625395	17.2618097	22.02792742	0	17.36327139	18.55229528	20.08413742	19.02634455	16.15355657	0	0	0	17.20645777
19.43614976	22.5086163	0	21.25257011	21.89455117	18.14422975	18.69430493	18.26472245	18.09536059	18.00515899	19.04844188	19.20003209	21.03117457	17.25240117
16.9281197	21.13886454	0	0	20.02558073	18.30517235	0	14.60064704	0	17.87445494	0	0	19.73050108	17.76926113
17.99436344	21.50619985	14.52024991	0	0	18.30678653	0	18.78226544	18.26218795	0	15.98078489	0	21.77051067	16.43483334
0	20.99882468	0	21.05787971	19.58103429	16.94262624	0	17.98229231	16.65425108	16.05634348	16.45003969	16.07093756	0	0
18.87206278	22.39086727	16.23685002	22.27103992	17.63729953	17.47668453	17.92392547	21.52316177	16.48723511	18.87802926	17.75970587	17.30864089	19.20765989	0
0	20.20620036	15.79141997	20.98427757	17.37135269	18.53580654	0	14.75853459	0	18.10722333	16.0050187	0	18.2830191	14.3331362
16.19255643	22.56578404	0	21.86068454	18.78005122	18.03636174	19.90878183	19.88425281	16.93601968	16.11687695	15.94773948	0	20.77451483	0
0	22.95579542	16.51439068	21.19648915	19.27850867	19.93252268	19.17139343	18.13033576	0	18.64584282	18.45260338	0	18.60185649	18.98245533
17.25129065	20.95578715	14.97696961	19.92958473	19.76275974	14.80948216	0	0	0	0	0	0	19.24272395	19.0704577
16.6692878	20.48759057	0	20.75477176	18.51282813	17.24347882	0	18.85238192	16.5862407	16.03060672	17.05418092	15.14235259	19.73815796	16.19129557
18.33614543	22.00798863	0	0	19.1142979	17.08131191	18.29695345	20.92043924	18.59641072	14.82078965	16.78391284	0	19.4854718	19.79727772
0	22.17329976	0	19.94855465	18.5619875	18.68760466	19.9840726	18.50655647	0	0	17.57658569	0	0	17.52355467
0	19.90431541	0	18.77601624	0	15.59338205	0	18.20153506	0	0	16.63091482	0	18.48948928	0
0	20.73732986	0	20.76739576	19.2172548	17.31001235	16.38468529	18.85832019	0	15.69422011	16.81755896	0	19.67503655	0
18.7468376	21.20127563	0	19.65267199	0	0	0	14.10833956	0	15.65407736	17.62085281	0	18.48154308	18.95721271
15.94990955	21.84866418	0	21.72157978	18.84004891	17.66851697	19.05910058	17.19424065	16.10464718	15.50739662	18.49126849	0	20.06921301	0
0	19.51153821	0	0	18.88347309	0	17.95810036	18.14881194	17.29493822	0	16.09571717	0	18.15377631	0
19.9902381	20.49779568	0	18.04922064	17.5169872	0	18.89686183	17.41932219	0	13.91648363	17.21332736	18.25567865	0	0
19.1444711	24.30903889	17.93804762	22.98769905	20.23377905	19.95445898	0	22.61468823	19.63491677	20.14515176	19.20341035	17.40022672	21.23942277	18.9671964
16.92780217	22.0774212	0	0	17.47490773	19.3827527	20.7139325	18.24883887	0	16.09571717	15.07682591	0	19.15528084	0
16.42712212	21.20778109	13.64896368	21.21466209	19.93083802	18.81776173	18.60722146	17.23954956	0	18.05440926	17.36487236	0	19.60821823	16.61411115
0	21.55889887	14.56939549	21.1436232	14.29078581	19.20804301	15.73884086	13.38465368	0	0	0	0	0	17.6019767
18.05060511	22.54731023	16.27559094	23.75408516	18.19156144	17.21993908	14.86179511	17.93582419	0	0	0	0	20.12219438	18.44782215
16.29657705	21.12368077	15.96949489	20.33758501	21.50015414	18.68287653	19.73967511	17.03013061	0	14.58926029	14.17792443	0	19.42761736	19.46854283
0	20.82144643	14.14761006	19.95124172	0	0	18.63706279	0	0	0	16.63095041	0	17.98155481	0
18.14762412	20.0171568	15.65873875	19.62963962	18.96527851	18.9514841	18.01516081	17.6716631	0	17.15299181	0	0	19.24004317	18.22879441
16.28355961	20.91086096	0	19.60460535	18.86730232	17.23770967	18.68015951	19.56729245	0	0	17.55858544	14.99051028	0	17.11022937
19.75432488	23.69016502	17.54368922	21.97754388	19.31945547	19.72643431	19.33039414	22.07704372	20.69287364	18.22921307	19.7572757	18.44380092	19.9707161	18.10653362
18.97993617	21.83352491	15.85244444	18.48732034	17.73762668	19.65575512	0	19.53205082	0	0	17.73964783	17.00659137	18.63493105	16.21817749
0	22.59849425	0	21.60396132	19.10765074	19.94667268	17.90810986	21.42037873	19.71869551	17.03825557	19.01227145	16.43770314	20.21323432	18.84270486
0	21.54317462	0	20.0980696	18.34260142	17.82588124	17.54019006	19.30433973	0	16.70139032	16.08907249	15.40801595	0	18.73121385
0	21.01440315	16.11321091	0	18.0267882	17.47494778	17.11130611	12.79155202	0	16.40527221	17.11593765	0	19.56919452	0
0	19.54660579	14.7195171	18.81814154	17.6276333	17.9646607	17.47958563	15.26841133	0	14.07981042	0	18.62827849	0	15.0974821
0	20.31184896	0	20.14046979	18.30289859	18.71982586	18.02888113	15.37311182	0	14.25762845	0	16.82800515	19.40297722	17.2685623
0	20.65098812	0	19.56685547	17.73768855	17.77349923	24.20209519	0	0	16.2825994	16.50137485	0	17.73977154	0
0	21.88381496	16.4840978	15.54423275	19.47793713	0	0	18.21048346	0	15.63481409	15.818283	15.125978	17.65245516	0
18.73985127	20.93988411	0	18.20719461	17.650664	18.25119459	18.91915834	16.69470248	0	15.54185807	15.6638801	0	19.95751926	18.31367134
18.69544979	21.20642162	14.05547808	19.31352693	0	18.6430201	0	15.88121118	0	0	16.44050868	0	0	16.61884163
18.46566201	21.17481382	14.77488054	22.16644175	0	18.71452087	0	18.1128711	16.85506472	17.62187768	16.70323348	13.77365163	0	0
19.49628425	22.61338382	17.00643183	22.59536227	18.49713232	18.64387839	18.61154696	17.06297546	16.97624029	15.78540781	16.28485813	16.51117631	20.2419564	17.63733529
16.28405076	22.80097663	17.09650787	20.52180912	17.72970833	18.53016394	18.41384676	18.28103803	15.60976804	17.01224623	0	0	20.29623254	16.55548666
0	20.8584373	15.67563293	20.54626868	0	19.26744371	18.34268604	15.40602743	16.69278944	0	0	15.42509009	17.9921599	18.1156139
17.44774806	20.99203086	16.32000272	19.18880471	18.29060384	18.32056693	0	17.58932593	0	0	16.78837085	13.95631178	0	17.19937493
0	20.7984693	16.3904801	18.71808057	0	15.39092312	0	0	0	0	0	0	0	0
0	21.59215163	15.21125205	25.28287889	17.88244495	18.30112753	18.80746334	14.80082686	17.80118831	18.58009491	0	18.66746964	20.457066	0
17.17961155	21.28738336	16.86164406	19.44569955	20.80243348	18.83609654	0	18.54980928	17.10437507	0	15.33918811	0	19.06332856	17.86797065
0	21.33867931	15.44470462	19.90678636	18.88130204	18.82253242	0	16.36633688	0	17.90526024	16.62451889	0	19.4111125	19.05164041
18.19157338	22.20309486	17.74333169	20.77655216	21.68528604	19.09292661	23.45673234	21.03387699	0	18.21051054	17.91812437	19.70200156	20.23419351	17.26443046
0	22.16014011	0	21.51342654	17.26604865	16.45563528	0	20.29790738	16.56170135	16.55203397	17.61984006	13.55892704	19.05983105	18.60493559
0	21.98471575	17.10096048	23.45912857	19.01697939	18.94124241	0	20.48191184	0	15.38298093	17.80393794	17.40855052	21.5037816	18.06196572
0	21.80728635	15.93660545	21.32302331	21.24205804	16.33916347	18.55733295	19.02957893	0	16.65649591	17.41377493	0	17.83181562	15.40201008
18.70233403	22.00067176	17.43242367	24.25974846	19.46892844	18.19689772	23.96769277	19.14706301	18.4403929	17.65913922	16.78390556	17.36849217	19.84000677	18.78611895
18.26223282	22.23409939	16.52736264	20.39514404	19.20009313	18.27424019	0	19.22692462	18.74549547	17.61329985	19.52869244	15.14891398	19.87972242	17.33057831
17.79967517	21.66030299	14.553009	18.20519614	0	18.18720475	0	19.23087825	17.43568947	15.44192765	16.38081277	0	20.58588011	16.39595461
16.81747076	21.07143545	17.04249145	0	20.88830885	18.6533281	18.37629918	18.59362085	0	16.59808073	18.83794012	14.4557522	18.35414808	18.1935907
17.84398917	22.29152284	14.27852632	19.75812521	19.38687227	18.80728883	18.53910839	19.73921234	16.95864235	17.31957979	19.63129043	16.53552462	19.89450024	18.10757185
0	21.73337411	0	19.29500642	0	18.3770177	15.99900654	20.43899413	19.48188193	0	17.55506364	15.12172067	20.60648668	17.39028972

PLOD2	POGZ	POSTN	PPP1R14B	PRAF2	PTBP3	RASA1	SMPD1	TBC1D5	TEX10	TRIP11	UBXN7	WTAP
16.50285724	13.79601867	20.37658477	0	0	19.78737491	0	18.20780468	16.80390113	17.38838393	14.3421507	0	18.17431989
16.9209217	0	22.42058663	20.74757818	21.09014817	0	13.15362984	19.00914469	17.04547614	16.82213614	12.39680117	19.11849271	18.22997976
16.16430033	14.44171213	0	20.8569229	19.23125483	19.32110763	0	17.43129706	16.06820587	14.78040704	17.00033495	17.48898272	17.85131557
15.54583035	0	0	20.73569136	20.99797913	18.1776841	16.55817957	0	18.20948522	17.3665298	15.32494038	16.96467738	18.61460056
18.06839304	16.7772052	0	21.31350205	21.08612425	19.45362136	16.36960541	20.33246651	19.39130796	18.30021829	16.75265652	19.7010028	17.87100148
18.34063446	13.93208948	22.09414925	19.73941836	20.23643348	18.85642949	0	18.6547609	17.36966483	16.49576072	14.67741922	16.40652284	15.99383078
18.11236227	17.55853316	23.37779706	21.44498928	21.34531816	20.32681527	17.95018741	20.06400189	18.01673901	17.39719745	16.03262327	20.08118742	19.68293291
0	16.32594858	21.65961196	19.5063563	0	20.20244692	17.20650071	17.29655487	17.84663581	17.29042528	16.53671802	19.31026244	18.74577243
16.15244439	13.39307767	21.99351125	18.15482274	0	19.43577579	18.02302647	18.19117433	18.10787934	16.51713961	0	18.57658324	18.64124607
17.90154722	17.12351131	23.27669654	19.25705473	20.98847645	20.8264495	16.72779275	19.95216974	18.63398092	18.03894297	15.16070553	19.72054251	17.68317032
16.22311922	0	23.86924881	17.78894632	0	17.77646523	0	17.22625218	17.03789037	15.93178815	15.12654047	0	0
18.3792904	0	21.44875062	0	0	19.2333976	0	16.5120781	16.03667323	0	16.19555588	18.88789488	16.58860778
16.64286471	15.29217567	23.23587158	21.50155211	22.19002137	20.02333185	17.69352884	19.20419817	18.16636809	18.65914186	16.7986914	20.26945594	19.14404553
20.62549273	18.09391178	24.68140985	22.52270272	22.57539258	20.75436976	17.94906914	19.90988038	20.12221332	20.17852885	17.12519629	20.27712945	20.6991574
18.21269153	15.03226024	23.96794234	22.41275136	21.80549186	20.33042294	16.84408312	19.31562099	19.36077812	18.57877888	17.51762349	20.18291416	19.45518738
16.00951137	15.21349194	23.40591774	21.74358525	21.74970868	0	17.71745248	0	17.85230571	0	16.34449447	0	16.88003753
18.61793661	16.55564126	0	22.68040565	21.2092414	19.55179603	15.57275192	0	17.06932373	17.39554868	17.45480152	17.32006089	17.72056001
20.21660262	16.33910345	27.39013427	22.84313471	20.75724025	18.25543625	15.63505791	17.8833924	16.40001496	16.7067232	15.77169835	16.03815211	18.09664654
19.62426091	17.11055108	22.11150958	20.41671519	22.46598644	20.08929915	18.11324688	21.4034253	19.85621384	18.42598596	17.48636068	20.06505031	19.71611277
19.67537816	13.41305101	0	22.81954004	20.19510023	19.20479771	16.95306488	19.37699373	18.17900236	16.68591329	16.09134449	19.49917964	17.81779473
14.02648437	14.71647449	0	21.30924859	19.01392785	18.29564257	16.9179375	0	17.46664953	0	14.38530333	19.57114338	17.49840074
22.49548125	19.76957301	28.6262134	23.55387482	22.47618335	22.99128832	17.75302873	0	20.74656957	20.46997819	18.20526947	21.30926217	21.95073778
18.33201371	15.02717877	0	18.35380008	0	0	14.63545456	0	0	0	16.39792641	16.90546448	17.8769164
18.55573264	15.13333171	23.25146433	16.57248464	0	18.85838802	15.97349354	16.45289978	18.28406372	0	15.26644766	18.81161261	18.35290847
16.89198653	0	0	19.26268111	0	19.0482106	17.31939883	19.20391546	18.18505444	16.35972818	14.73729973	18.24231098	0
18.37578501	14.63545456	21.9906664	21.24863825	21.14385234	18.71882647	17.75769904	18.25348889	18.79954917	15.77066309	16.61795358	0	18.32110738
17.2802536	14.52442462	22.56025832	21.13282711	20.39864491	18.15071801	13.51250529	15.24092377	18.99069473	16.89633504	15.68569235	15.66014909	0
17.22351672	14.37029127	23.45705236	22.08977642	0	18.91424915	15.87681367	19.2923249	19.8652804	15.32607144	17.00953965	18.75992955	19.20681167
16.81905493	15.71663864	23.2789939	21.98173118	20.99533177	0	16.49058657	18.53080087	18.79779942	15.80402575	16.32298513	18.08973997	18.08287317
19.03574899	0	22.38278874	20.96825096	21.22212453	19.62670356	17.65493327	0	19.29106196	17.52154052	17.102353	18.51350665	19.57453954
17.28087248	16.74195509	0	21.93915885	19.50102682	0	17.02332006	0	0	15.94824775	13.99529549	16.57195293	17.83270971
19.23596954	13.5602947	23.30988654	22.78739125	22.81828411	20.82999839	17.81158159	18.2971343	20.91901203	18.47175295	18.5186296	19.26992176	20.07536164
23.78131221	17.25891512	28.85436857	24.60493992	17.74409978	22.38476289	19.70792699	0	20.14770852	19.60748339	17.93719728	18.44605934	20.67243971
19.01476229	11.96343175	24.16976442	23.13298782	0	20.46500983	17.94909796	19.14439221	18.35369345	16.55698455	18.25031962	17.45265269	16.85430794
17.19686678	15.81530332	0	22.40371309	22.2077173	20.1940465	16.51432386	19.30816319	18.00346214	18.21229064	18.85828739	17.69441054	19.26682283
0	0	0	20.82050101	18.02282997	19.4507584	15.79902218	16.84131055	0	0	14.7294698	16.38571564	18.25821436
16.80622624	0	0	21.92899164	21.18964652	0	14.68622532	17.46061581	18.60051121	16.56850854	16.42053273	0	18.97214824
17.27003213	16.93665269	24.44246735	22.78276236	21.30203881	20.28752604	17.03880739	18.27794237	16.26940373	16.65215454	16.10549353	16.72176435	18.63013564
17.42507928	16.2027835	22.46012725	20.79067487	21.00555777	18.58731085	15.8249192	17.75040866	18.70039052	17.31099	14.86412177	18.89008903	17.47219017
17.50609039	14.00792847	0	20.85888021	19.88749151	13.84194561	14.4152549	0	16.11891044	13.46593691	17.46773517	15.57901091	17.62366672
0	13.9952311	0	20.2452172	21.31212384	18.56599175	15.78620747	0	16.6751894	14.4906999	14.45704815	16.12171212	0
18.4427323	17.15431255	0	18.73028154	20.47840523	19.61998479	17.49346035	16.05477081	16.94702384	15.56027244	14.79413627	17.17149496	0
17.51555949	16.77878558	0	22.73998959	20.11189659	18.74073684	17.17939476	17.89947025	16.37824621	17.79085775	16.73672305	16.94673307	18.58904484
18.97076921	13.26138109	23.51817761	19.2968837	21.13832254	19.82418633	17.8636668	18.29061915	18.18118232	16.86230396	16.97775335	18.82193418	17.12935961
0	15.52405603	0	21.29755658	19.4152293	0	0	0	16.43832868	19.19892689	16.23554083	0	17.01015701
16.81683615	15.14368323	0	16.4674264	0	0	14.5823702	0	17.22112876	16.68071818	16.95014392	15.41804533	0
16.82188	16.19507289	0	22.36347912	20.76432681	19.88387789	17.18170652	18.40378608	18.95890163	16.57187934	16.89193803	17.51632238	18.72491782
17.41889668	16.07298509	0	20.94896926	21.06838437	17.31551684	17.82730422	0	18.05878159	18.34038022	12.98567058	0	18.14173011
17.02226491	16.15851223	22.67385411	20.57190586	20.55614019	18.47274362	17.5901594	14.26234867	19.88455538	16.45203019	15.34258159	18.5045258	17.87615208
16.48383424	14.55419807	0	21.15494299	0	18.721656	15.84195535	0	0	17.08464108	14.54604059	0	17.66701949
0	0	22.5152118	0	0	0	16.21556882	16.7758828	15.81605974	15.17835361	14.61900979	0	16.30494673
17.20141762	0	0	20.63141397	19.82577264	19.31114836	0	18.90508969	18.87005871	17.31119376	14.20403417	0	17.99213448
0	0	0	0	19.37471115	18.03968672	0	18.77915564	16.79285622	16.92362633	14.78618742	19.30874994	18.36156076
16.13417695	13.72345461	22.3133635	18.93003882	0	0	15.79576532	0	17.46338072	16.73973817	15.55709046	15.66089072	0
0	0	0	20.80660108	0	18.576384	0	17.18633586	16.91278695	15.58838104	14.13654535	0	17.4554207
18.91890525	16.21126497	0	18.42349256	0	19.58392452	17.55634013	16.31838141	14.8778607	17.3424173	15.56602868	16.26530239	14.98571021
17.37207648	15.30274023	0	19.71136537	20.49264092	20.06176621	0	17.04231425	14.66659844	16.61672759	0	17.85988061	17.40191178
16.126496	0	21.86938457	21.68254641	19.47464731	18.9820673	15.99048731	17.84981853	0	15.31869652	14.27747023	16.77918349	17.81905227
17.52935029	13.86645068	22.51912843	21.40067034	21.14178572	17.47243855	16.13408081	0	17.67897927	15.08956961	15.60382785	18.44972701	19.32176722
17.69130894	16.52408803	20.6901911	22.02946279	21.54309176	20.39197041	13.89997216	20.40199469	18.96287982	16.58672543	15.38008199	18.2610234	18.84788508
18.68322747	0	21.5449778	20.23270944	17.34594107	18.52954099	14.7234604	19.01884198	17.55481623	0	14.79890499	0	16.22903547
15.59873823	14.85338344	23.18810097	21.01130657	21.30146189	16.8949397	16.69590487	18.42318482	17.81902972	16.17491498	15.9917466	16.24447193	18.5179645
0	14.19785823	0	20.19803989	0	17.73226142	14.10535175	16.41958764	15.2483342	14.13985805	15.46467838	18.58668737	0
19.5381489	16.26819899	24.24347175	22.64508639	0	17.9325821	22.05110029	0	17.46056328	17.62212246	16.58103093	21.84023999	16.3354741
18.37608142	13.87046328	22.35947633	20.40137515	0	19.32622295	0	18.86274016	0	0	15.20384692	17.17346857	18.15417712
0	0	0	21.44689039	0	15.13501367	17.10670485	0	14.62584321	17.63974114	16.70578919	0	15.57811156
17.89579435	15.47537637	23.23341603	21.1495134	18.91225607	19.50236476	17.04567882	18.65233107	17.05835215	17.50848411	16.81403428	18.04895756	17.20220509
0	12.15929495	20.81361171	0	0	19.18423963	0	18.79961816	19.21017256	0	12.77186574	16.76701277	16.686278
17.78088263	0	21.24120002	21.36388348	0	0	0	18.23502087	0	0	14.72384945	17.20424445	18.12486153
19.58717585	0	26.10312957	23.62371738	21.63370824	0	15.56317941	17.96873844	0	17.62477773	14.30126594	17.59710546	0
14.64327031	0	0	20.21591155	0	0	14.38049656	16.55173526	18.29765685	16.65153947	14.03155931	15.49572535	18.00362078
14.65323948	0	0	0	0	16.89756766	14.64310269	15.50100282	17.5056099	0	0	16.98247221	16.90125222
14.99354098	16.08538999	0	21.48183271	20.78154311	18.87836812	14.94989275	0	15.56066356	14.86434434	12.81074743	18.43121582	15.9357268
0	16.39826087	0	22.16672784	17.13806228	18.26693904	0	0	16.70660472	15.177163	0	17.8093882	16.50219657
15.96469787	0	0	19.49247433	20.08128026	0	16.44948723	16.70168236	18.10950579	16.4997696	13.76332456	14.75436688	0
16.6132781	0	0	20.18528177	0	19.49914614	14.10381561	0	0	15.5647617	0	0	0
16.53748932	0	0	22.45219434	20.37925108	18.02654035	14.39174718	0	16.74933957	0	14.79756909	15.81927535	16.34100379
21.03120215	18.3015151	0	22.54932491	23.71175588	19.8317837	19.5087785	0	17.94816757	19.26400536	15.15408145	20.08823755	19.62908222
17.04910771	0	0	20.88274976	19.62416854	18.90374497	16.16601498	17.96257443	16.74463805	16.54964006	14.81303703	17.52521047	18.18050521
17.20304067	0	0	0	0	17.24411858	16.7346117	16.76626464	0	16.44721742	17.45351628	14.82965316	17.3356537
16.80453027	0	0	15.93853689	0	0	0	12.84092036	18.07225202	17.77541247	15.83813328	14.52890294	17.37391697
17.0694964	15.02845361	20.67602185	20.0839618	0	19.78747969	16.26230488	18.35588569	15.6562012	15.57697746	13.22482449	0	16.59503502
18.56509954	15.69292344	0	20.23518213	18.58645815	19.81210332	15.68894057	0	17.52225097	0	16.94045127	18.50223323	18.66476362
16.83166841	14.41617707	0	20.17180954	18.64494306	0	14.31084817	0	0	17.22798846	0	17.56126067	17.34382579
16.7422605	0	21.95871434	21.25489954	0	17.35727965	0	20.30699121	18.38684274	0	14.46245678	0	0
19.76502109	15.5848619	0	19.3424976	21.96734657	0	16.87058722	15.49573811	18.61965209	17.4892203	16.08570421	18.96358285	17.69071682
22.08013534	17.28901942	0	22.61452509	21.75165098	20.16810721	18.42524784	17.10518965	18.55411967	18.45678873	14.94080902	18.90386696	17.75264366
17.00899825	0	0	21.4575388	19.81482835	0	0	17.32671399	17.88718589	16.45174969	15.97396698	18.18036849	19.3927797
19.50145571	16.84748524	26.79808142	22.24684271	21.98986252	21.51231663	18.02191924	16.2870624	18.18634553	18.65261404	14.04577836	20.4293493	18.97655138
17.8121186	16.16394485	22.68145256	19.81673339	19.01286214	17.98030503	15.7331667	18.08410287	0	17.40296796	16.91107915	0	17.42100345
16.86892761	14.65557518	0	0	20.20404865	0	15.36080838	16.62284252	15.8588673	17.57267992	15.71162062	18.61403567	17.68985064
0	13.41552611	20.86376173	19.55323372	19.1358514	18.62625367	16.16652482	18.78859523	15.62776958	0	15.51960464	16.80203813	17.16940671
0	13.99463927	20.2334675	19.80370354	0	0	0	0	17.26132522	15.53386051	15.32922812	17.66495642	17.08345212
0	0	0	20.63201397	0	18.44964278	15.30123714	17.22694996	15.08371722	17.99806155	16.6705182	0	18.61168591
16.46031579	0	22.07499163	0	0	18.22589557	16.47208696	0	17.18633972	16.33655059	0	16.10584396	16.48962631
14.29219704	15.01119256	20.56621815	19.61916525	0	17.64481548	16.29075463	13.88435304	18.13844621	16.10534012	14.74523321	0	0
17.4482196	0	21.68900438	20.90028572	20.14879138	15.90905212	14.66699307	16.9647701	16.79991111	15.55205527	0	16.56173216	18.35375279
19.03999933	13.78726634	24.63950402	19.23987972	0	20.32157939	18.58161663	18.47825898	18.2375424	15.80501303	14.76072753	18.60803322	16.8651808
13.89024797	13.58068808	21.40534012	20.63516936	0	18.25415312	15.59316972	19.11493278	17.21930731	15.73624669	15.35236339	17.29291716	0
17.54494546	0	20.75151043	0	16.76166027	17.84600722	15.3123367	19.19807306	16.69475177	0	15.02087921	18.6696494	16.78614991
15.7602573	0	0	0	20.18106886	18.99479085	16.46636633	18.81814154	14.14988642	16.37415867	15.87655029	16.68721154	17.16940671
15.60806815	0	0	0	0	0	15.66924565	0	18.06503376	0	16.27386841	18.94626387	16.85080087
0	0	0	20.44873156	0	18.24431609	15.28661977	16.52594014	13.72181592	15.28058581	0	17.42973467	16.1395921
14.57220774	0	0	0	19.71426494	19.40036522	0	18.76457493	16.57839633	17.95800403	15.41701022	18.30317515	18.70067371
15.07377176	0	0	16.95552122	18.67690954	0	17.04678001	17.97953331	17.34214334	18.05270104	15.70312584	19.03464925	18.10689928
16.71972438	16.59546237	0	20.81168337	0	18.36285646	15.51813879	17.26938225	17.71834554	16.43131848	16.30578546	18.53384615	16.55475365
17.46355568	0	0	20.90999523	0	14.43029314	16.65745844	16.84157727	18.10796879	16.48673286	17.00958407	18.30311763	19.18869258
18.05414018	16.80274268	0	17.49959318	19.25209432	0	15.85615478	18.52447098	18.18597538	17.18625651	16.41052436	17.95076571	18.56157132
19.44818729	14.54324972	26.34030794	21.96889812	20.46461239	18.99255981	17.86668841	19.52306124	16.2880696	17.71876363	14.53007833	18.78857101	18.7133083
18.79108724	15.32679671	0	20.52965301	0	0	0	0	0	17.70391381	0	19.12590823	19.98235021
16.56962783	16.40199661	0	21.27492547	0	19.80876237	17.39731982	18.88203738	16.31954664	17.47031317	16.04672834	18.08165939	17.87941426
18.62830562	16.10324234	24.17221533	22.68854174	21.23526115	18.78307521	14.86514329	16.99406104	18.19475731	17.80376014	16.24283747	18.18946218	0
17.11214454	14.27758461	22.1474812	21.05750466	0	18.41820096	15.90910556	18.96451086	17.75416632	16.81311742	16.6634661	20.45659573	18.34792674
17.66093961	15.76950268	19.95854119	21.62605704	21.632976	18.35072017	16.87299537	16.15226513	17.26210438	15.4968191	15.3536563	18.05433018	17.76641764
17.92215984	16.83878178	25.50607621	22.40798704	19.10645998	17.89366891	13.96736997	17.72427151	18.50259773	16.66983549	17.1750866	18.8671862	17.16225175
16.67105535	0	24.74773879	22.74170122	20.94703042	13.60278807	13.69415982	16.85556205	17.84347306	18.0065036	17.29305002	18.25688958	16.43946756

Supplementary Table S9. Hazard Ratios (HR) from Univariate, Multivariable, and Backward Stepwise Cox Regression Models

name	desc	stats	id	HR (univariable)	HR (multivariable)	HR (final)
ENO3	Mean ± SD	25.6 ± 2.8	ENO3	0.88 (0.82-0.94, p<.001)	0.92 (0.85-0.99, p=.033)	0.91 (0.84-0.98, p=.010)
GMIP	Mean ± SD	9.5 ± 8.0	GMIP	1.04 (1.01-1.08, p=.006)	1.02 (0.98-1.05, p=.320)	
HSPA13	Mean ± SD	17.1 ± 4.8	HSPA13	1.10 (1.02-1.19, p=.019)	1.06 (0.99-1.14, p=.107)	1.08 (1.00-1.16, p=.048)
MANBA	Mean ± SD	13.3 ± 7.3	MANBA	1.04 (1.00-1.08, p=.056)		
PLOD2	Mean ± SD	15.3 ± 6.1	PLOD2	1.05 (1.00-1.10, p=.053)		
POSTN	Mean ± SD	11.9 ± 11.6	POSTN	1.03 (1.01-1.05, p=.009)	1.01 (0.99-1.04, p=.181)	1.02 (1.00-1.04, p=.110)
PTBP3	Mean ± SD	15.0 ± 7.8	PTBP3	1.04 (1.01-1.08, p=.019)	1.02 (0.98-1.06, p=.280)	1.03 (0.99-1.06, p=.145)
TBC1D5	Mean ± SD	15.6 ± 5.7	TBC1D5	1.06 (1.01-1.12, p=.023)	1.02 (0.97-1.08, p=.408)	

n=116, events=73, Likelihood ratio test=25.19 on 8 df(p=.001)

Supplementary Table S10. Targeted molecules expression profiles in iCCA ATTs samples

Sample	EGFR	PARP1	ERK2	ERK1	SRC	CDK7	MCL1	CDK6	ATR	CDK4	CDK1	WEE1	WNT16	PARP2
P179	2170260.756	5737131.369	6486594.563	742079.0793	1090253.317	2021770.937	368695.9854	100146.9174	NA	NA	NA	NA	NA	NA
P97	2151346.636	4468898.332	3616512.92	1054489.989	465818.1109	795723.6949	NA	129596.7819	135404.2511	NA	NA	NA	NA	NA
P103	1085501.919	2427620.775	3634916.005	968992.9815	1917391.347	630874.8254	1390810.627	211261.0963	NA	NA	NA	NA	NA	NA
P104	2430720.711	5736809.71	8423234.509	1007440.4	58881.69559	1362344.886	366165.2764	232325.6698	NA	NA	NA	NA	NA	NA
P105	2304013.524	8747052.464	6060066.367	586629.0024	1473343.479	NA	175522.8494	139287.2627	NA	856834.6507	NA	NA	NA	NA
P131	1544676.611	5344348.526	6957410.597	1227787.132	306530.757	927102.1465	402676.729	NA	NA	NA	NA	NA	1828420.433	NA
P136	1350405.498	3786309.183	10809689.73	2098120.868	498436.9171	752888.6708	214871.1148	NA	NA	NA	NA	NA	NA	NA
P140	722119.8086	3711180.803	3293151.149	1640294.337	NA	1819694.519	44774.01514	NA	NA	NA	467427.6167	NA	NA	NA
P148	1292466.212	2385532.366	3461565.829	668872.447	149654.9509	673143.6385	NA	613940.2785	NA	NA	NA	NA	NA	NA
P152	2631129.679	4683981.288	5912342.919	244950.3503	354262.6016	1413935.582	NA	490670.6267	NA	NA	NA	NA	NA	NA
P153	3240096.058	5460412.521	3984723.307	727832.8895	1140082.401	838008.9947	199020.7039	84071.46402	NA	NA	NA	NA	NA	NA
P154	2224492.261	6199107.774	6789550.745	495595.1136	1631517.537	2355059.351	232926.2264	741502.879	NA	NA	NA	NA	NA	NA
P162	683412.9281	2663852.409	5179849.396	1577767.128	79211.89615	911042.7233	64112.12719	NA	NA	NA	NA	NA	NA	NA
P164	2062667.084	11287542.57	11272799.85	2186591.843	1032080.509	103762.6262	494709.0885	515881.3717	NA	NA	NA	NA	NA	NA
P159	2332883.081	7443134.981	6214415.118	NA	288681.6535	NA	1184799.02	310160.5855	NA	NA	NA	NA	NA	NA
P166	1118472.558	13881402.94	23267078.07	3437954.077	2541810.478	189845.1415	NA	66232.46651	4288.519287	1036918.873	75640.21741	NA	NA	NA
P173	2894262.128	9866670.664	8529459.833	1235929.265	1098196.16	NA	399848.645	382787.351	2047.325355	NA	NA	NA	NA	NA
P177	1308922.67	6617063.308	9696384.478	821368.6335	819479.2476	NA	220106.0622	432188.5982	21262.53642	NA	NA	NA	NA	NA
P184	2158247.238	5027882.219	5380741.495	923802.3573	881133.0082	910649.6231	71138.74513	601346.6398	NA	NA	NA	NA	NA	NA
P187	2489734.532	5283315.449	7936013.469	1428937.052	525118.001	1349590.516	66110.76473	1134083.005	NA	NA	NA	NA	NA	NA
P19.136	1274344.632	8354246.187	11431560.24	3517070.972	2062416.963	1206757.797	NA	NA	NA	NA	NA	NA	NA	NA
P19.144	1593422.961	3832071.372	6104743.062	1580630.038	NA	1621205.746	139315.1259	184137.2134	NA	NA	NA	NA	NA	NA
P19.17	1734391.256	4502226.431	4325218.308	1159405.853	44741.76432	999551.1892	335032.0433	NA	NA	NA	NA	NA	NA	NA
P19.177	2157770.971	6224824.574	10726789.21	709378.9985	989459.9707	NA	423924.5407	439567.1247	58106.08104	NA	NA	NA	NA	NA
P19.206	1605624.961	4969466.702	3863782.896	1005664.499	1304856.991	1654689.678	158418.5083	282045.8992	NA	NA	NA	NA	NA	NA
P19.220	1975134.043	2640890.002	3896792.156	1596959.058	819075.6223	1494709.459	80856.72296	942331.663	NA	NA	NA	NA	NA	NA
P19.325	1397825.327	4305207.159	5923892.175	1561911.658	1340414.944	66731.57225	NA	254765.0627	NA	725316.9033	NA	NA	NA	NA
P19.413	1696565.274	3186317.928	5333954.251	1415130.579	292357.9903	44560.76436	588814.3773	230275.2461	NA	NA	NA	8827.970445	NA	NA
P19.557	1479995.058	3370162.69	5034194.871	1564499.042	387152.5302	NA	1004817.954	139463.1631	NA	NA	NA	NA	NA	NA
P19.603	2023158.125	6204608.735	6632353.31	1603079.968	1284481.881	1355863.289	63740.39927	91423.35894	NA	534247.6296	NA	NA	NA	NA
P19.607	3073896.18	5819029.534	4107915.245	1137286.594	1683818.64	1370250.339	347715.9493	NA	NA	81984.55238	NA	NA	NA	NA
P19.699	1456692.096	2715472.724	4830058.468	607180.9792	1334310.776	1353440.756	21832.86654	NA	NA	NA	NA	NA	NA	NA
P19.705	1474500.487	6629774.661	7441727.783	1888409.167	1752987.771	2405584.511	473112.2828	245329.9093	NA	NA	NA	NA	NA	NA
P19.793	2907889.548	4709296.335	7241085.619	1789729.352	800109.1725	675069.3499	394826.3427	260291.3676	NA	NA	NA	NA	NA	NA
P19.85	2042020.132	8816691.921	7808773.516	2244514.398	2487075.526	963125.9377	122194.8198	163739.3793	NA	NA	NA	NA	NA	NA
P190	2167536.048	4405827.09	3732512.195	1485823.323	210994.8615	1110429.171	NA	609136.4433	NA	NA	NA	NA	NA	NA
P195	1674660.634	2307818.665	6595700.838	542276.8854	174478.3106	123511.5018	NA	NA	NA	NA	NA	NA	NA	NA
P198	1328917.079	13793678.83	14969263.85	2455412.965	1240314.536	815790.6861	205509.1337	NA	NA	NA	NA	NA	NA	NA
P19.98	2028285.372	4649459.804	4684903.611	683305.29	225974.0648	NA	398908	161582.1726	NA	NA	NA	NA	NA	NA
P93	2476853.368	6313817.7	8632038.868	1701369.943	264334.0899	619557.6957	558632.2943	2145086.95	NA	NA	NA	NA	NA	NA
P98	1988908.573	6213294.493	7456603.05	232042.5824	524878.5718	501764.3115	533985.6882	1170544.555	NA	NA	NA	NA	NA	NA
P99	2376861.798	3880232.216	7003986.291	179088.131	463326.2953	NA	NA	1155499.921	NA	NA	NA	NA	NA	NA
P130	1473093.881	3829158.105	15865834.32	1163935.625	1395386.228	1339364.381	277313.3042	25025.46145	NA	NA	NA	NA	NA	NA
P20.31	1426130.214	3679823.961	6745078.927	1332358.105	752369.422	1585407.141	55250.38195	229931.9273	NA	NA	NA	NA	NA	NA
P2	2091039.931	4636872.06	6293165.012	985630.7705	409388.2689	328571.3505	845866.3011	773057.749	NA	NA	NA	NA	NA	NA
P51	996584.9955	2006527.05	3808030.432	NA	784132.7399	365187.7357	783096.2071	NA	NA	NA	NA	NA	NA	NA
P20.42	1395946.717	3926465.785	5182632.859	1164188.9	272128.434	1370332.183	303258.6376	255877.1558	NA	NA	NA	NA	NA	NA
P52	1393215.982	3945978.02	5812449.152	645489.7704	669277.8421	823578.9648	162982.8542	127547.9542	63125.50107	NA	NA	NA	NA	NA
P12	1498485.017	4791319.334	5450323.24	1473721.438	1152269.383	1221728.838	413909.5045	403561.6371	1456.823836	NA	NA	NA	NA	NA
P19.3	1521860.15	3138655.925	2557018.173	200938.1212	879243.9173	706530.9812	NA	NA	NA	NA	NA	11378.09515	NA	NA
P20.56	1227166.5	2900032.648	6259576.507	2003331.455	1177905.499	1254145.181	107019.6103	NA	NA	NA	NA	NA	NA	NA
P101	1852386.184	4670537.985	6002182.945	1908119.341	969952.7687	379132.1298	170293.9844	113801.1419	NA	NA	42506.59275	NA	NA	NA
P134	2750266.335	12331712.93	10023353.98	2169824.449	1598689.096	770268.9748	566400.4453	557943.8467	NA	110197.0247	NA	NA	NA	NA
P147	1126879.272	57351382.16	21404684.08	9165587.129	14128201.41	541091.1957	75847.62992	1383762.652	31323.29572	325505.3341	8564306.687	52495.24284	NA	NA
P4	1817094.904	3378949.118	4662246.056	2037289.618	868781.3621	557709.9948	594902.8651	NA	26526.6957	NA	803856.0366	NA	NA	NA
P110	2248122.69	7434882.457	6671327.895	401745.6551	2272919.378	876695.2876	104990.7783	190996.564	NA	157713.0729	1287975.673	NA	NA	NA
P117	1576147.847	5370699.347	7376834.101	688177.128	1668138.594	1950993.977	319282.4657	237830.9842	NA	NA	NA	NA	NA	NA
P118	2904343.202	8045006.059	8636099.573	498772.6247	1232593.616	1697394.753	836274.4897	713456.2396	NA	NA	NA	NA	NA	NA
P19.217	3216003.802	4651738.039	7601921.982	2179927.056	1748763.407	832312.55	47631.94524	235064.3656	NA	NA	1131258.387	NA	NA	NA
P19.511	2832350.493	2696117.252	4254125.979	1628316.872	839475.0716	1393676.464	39688.70079	53124.2376	NA	NA	NA	NA	NA	NA
P19.689	1368045.051	3828100.949	8230202.563	1490870.653	1308941.656	NA	868402.7577	261826.3109	NA	NA	NA	NA	NA	NA
P235	2341455.291	6283611.665	8617625.464	1328125.106	606944.4088	325786.2874	157282.3754	702080.4769	NA	NA	NA	NA	NA	NA
P108	2214782.392	8803605.916	5429309.738	496439.1679	1081158.803	104725.6183	92049.10047	580626.218	NA	NA	NA	NA	NA	NA
P121	1915088.405	10527776.61	12686617.66	3945783.824	289971.6127	988943.6866	59627.41309	850881.3187	NA	NA	NA	NA	NA	NA
P123	974274.4411	14072868.87	15375095.5	2369208.149	2913191.686	2123843.456	98926.48269	506504.8163	356427.6928	641043.5491	NA	NA	NA	NA
P126	2141909.331	11359871.59	9900936.271	4398539.69	3188095.888	1848083.481	578885.9741	637649.4386	54262.64765	378729.107	NA	NA	NA	NA
P19.642	1563658.198	5943569.14	10095417.91	2795525.223	516072.3668	497510.8691	613075.5614	256471.2368	46801.38439	172691.8259	NA	NA	NA	NA
P19.649	2192033.699	5148800.326	5406777.034	2011344.248	NA	1205682.708	207290.4026	NA	NA	NA	NA	NA	NA	NA
P19.873	2214955.534	3856003.008	5002929.345	1029983.279	206590.5923	930361.202	NA	96541.08233	NA	NA	NA	NA	NA	NA
P3	1364272.645	6139493.94	7346813.609	2715321.241	135175.8983	965473.6582	467499.3079	NA	40768.1754	NA	NA	NA	NA	NA
P106	2132869.102	4878038.59	4129049.887	1495850.43	301876.5679	625813.4898	NA	NA	NA	NA	NA	NA	NA	NA
P150	2374045.452	4967754.725	5946221.175	645494.2565	753344.0861	1485429.17	114410.7003	571007.9666	135246.6663	NA	NA	NA	NA	NA
P129	2094430.987	5812697.649	6092084.908	1156687.874	597011.3499	112433.0645	673378.348	259585.158	NA	NA	NA	NA	NA	NA
P156	3788776.639	6488926.468	9757266.846	145373.9733	792906.3125	180546.1094	1072104.512	360447.2944	NA	NA	207862.6432	NA	NA	NA
P158	3158506.279	6764436.24	5144794.372	1004385.17	306151.1287	NA	476485.9675	318339.7248	211634.1667	165737.473	NA	NA	NA	NA
P119	2403845.874	2874704.676	4729538.025	1651231.836	1558939.99	1562894.133	47570.86008	NA	NA	NA	NA	NA	NA	NA
P167	4174826.123	5263556.411	5428465.193	152453.9923	412841.9519	88117.82324	538405.4185	592751.3156	NA	NA	NA	NA	NA	NA
P181	3594803.253	4818934.979	4634622.436	256460.8573	1745211.524	1140900.463	19372.93813	301989.1972	NA	NA	NA	NA	NA	NA
P19.140	2296190.704	7071149.586	6088454.426	571189.6904	1510450.184	1426576.291	NA	507902.7115	NA	NA	NA	NA	NA	NA
P19.62	2305930.98	4444699.01	7486928.486	2758896.143	1107915.249	457562.903	168355.2898	444206.446	NA	559729.0572	NA	NA	NA	NA
P209	2407561.526	2657563.574	5540414.157	1968420.656	559450.3505	1023281.005	147657.3273	NA	NA	NA	NA	NA	NA	NA
P224	1618598.561	4282067.527	4902192.603	301054.2881	1658837.335	1845152.003	271081.3006	NA	NA	NA	NA	NA	NA	NA
P225	1716075.034	3090766.436	6351099.129	1162375.478	749216.1794	1118518.886	139373.3149	200283.6001	NA	NA	NA	NA	NA	NA
P232	1638050.333	4179549.546	4731262.807	2064692.354	166229.6078	1297405.734	66529.11213	106661.4723	NA	NA	NA	NA	NA	NA
P234	1638474.944	3135058.462	5171743.902	1146661.488	394623.0745	1094613.126	NA	121629.7108	NA	NA	NA	NA	NA	NA
P19.608	1763362.676	5623184.898	5674396.346	594524.8892	339682.8847	225663.5706	320100.216	278050.7697	NA	NA	NA	NA	NA	NA
P19.752	1722383.161	3427276.73	5176010.446	238409.501	488374.2326	1386491.695	NA	326511.2349	NA	NA	NA	NA	NA	NA
P19.893	1214056.527	4251889.624	6252302.036	452807.7745	915509.3686	113413.2261	202930.2291	210897.3106	NA	NA	501541.3474	NA	NA	NA
P176	1750134.247	2310114.289	3518221.962	452902.5697	71939.32083	398856.7375	110452.7238	NA	NA	NA	NA	NA	NA	NA
P191	1002062.647	1651088.272	1867050.48	564798.8146	NA	NA	NA	NA	NA	NA	NA	NA	NA	NA
P192	1596525.618	2890053.363	4358994.48	404598.9429	57712.25968	1946766.306	210743.2111	503160.2305	NA	NA	NA	NA	NA	NA
P193	922440.5948	3863640.226	3970731.171	938636.9173	185268.7427	NA	87542.22828	NA	NA	NA	NA	NA	NA	NA
P182	1777808.096	4355235.22	6459100.345	128722.5293	2124676.57	437219.5104	254671.6594	NA	NA	NA	NA	NA	NA	NA
P200	2692337.011	4919622.293	2390385.484	1820948.443	1977757.185	NA	184056.734	NA	NA	NA	NA	NA	NA	NA
P196	626623.14	3098137.244	2204624.626	379107.851	20633.51034	622202.6166	NA	NA	NA	NA	NA	NA	NA	NA
P197	572823.1021	2430521.758	5506255.594	223912.4215	107796.0508	NA	68627.06699	NA	NA	NA	NA	NA	NA	NA
P203	1213515.662	4059673.878	4836418.209	1060894.021	35385.88321	1276228.551	152478.0816	NA	NA	NA	NA	NA	NA	NA
P211	2256458.692	4791173.452	3656350.061	288807.2609	642714.2575	1481780.026	537495.1428	268267.1498	NA	NA	NA	NA	NA	NA
P214	1762835.66	6531853.261	5677414.166	1308060.944	622498.4974	1429933.004	240480.0008	278580.8607	NA	NA	216595.1789	NA	NA	NA
P216	1388782.549	5404045.427	4628832.445	689960.8714	98226.21351	1209359.271	214897.4335	60860.83721	NA	NA	61313.0518	NA	NA	NA
P219	1407717.184	6958212.027	5010535.082	1026571.898	992349.2744	1628461.858	396272.3303	NA	NA	NA	NA	NA	NA	NA
P220	1536948.694	4165624.916	5159002.907	1089967.567	1388233.891	1397353.086	373871.9138	234568.9746	NA	NA	NA	NA	NA	NA
P222	1425245.781	3781281.131	3937281.287	719673.0723	206318.665	281842.5492	144053.9932	NA	NA	NA	NA	NA	NA	NA
P226	800578.1988	3583435.929	4011423.694	1378409.227	1829537.142	532020.8794	236260.4791	NA	NA	NA	NA	NA	NA	NA
P231	1542354.398	4694538.543	6296691.512	1634999.037	114064.7634	618492.2304	240274.121	703593.4451	NA	NA	NA	NA	NA	NA
P45	2358033.44	4075343.867	2094805.969	1113730.5	109801.9745	NA	222654.6123	513390.3742	NA	NA	NA	NA	NA	NA
P46	1252938.329	2995005.7	2763740.218	1027602.132	1570203.641	582069.5925	NA	143718.4991	NA	NA	NA	NA	NA	NA

P55	1927692.486	4293192.5	5121802.8	1964798.86	1306734.766	694894.4182	46208.93561	305703.9736	NA	NA	NA	NA	NA	NA
P57	1765316.872	4004109.158	5607146.066	508614.6234	105014.5799	531239.3975	371193.0645	36143.6584	NA	NA	NA	NA	NA	NA
P68	2916633.571	8870609.385	6290546.767	1224537.987	1728296.794	1472936.329	174955.0128	52410.13803	NA	NA	NA	NA	NA	NA
P71	2191938.486	7588256.586	7944270.863	1870558.357	1927349.999	847737.2184	139048.444	400005.1739	NA	NA	89550.17193	NA	NA	NA
P86	1962264.901	7748344.74	7151777.721	2263448.699	2483336.414	1419703.762	664942.2534	1243130.067	NA	126635.1529	NA	NA	7091237.192	NA
P89	2248681.998	5759967.622	6048359.514	1792512.589	2327553.142	1341300.985	NA	190465.2002	74626.92235	NA	NA	NA	NA	NA
P90	2321946.967	7290674.437	6283123.601	523788.7724	1871747.606	1171862.637	539808.8141	221616.6873	NA	320714.1605	150540.2418	NA	NA	NA
P95	613062.4995	4921875.688	3365808.61	863039.6869	187822.856	361290.967	NA	220062.9162	21786.99128	NA	NA	NA	NA	NA
P102	979938.3432	3967878.196	3999141.421	20467.23574	711369.9884	686131.5255	215637.7309	NA	NA	NA	28824.27811	NA	NA	NA

Supplementary Table S11. TIDE scores in iCCA ATTs samples

Patient	No benefits	Responder	TIDE	IFNG	MSI Expr Sig	Merck18	CD274	CD8	CTL.flag	Dysfunction	Exclusion	MDSC	CAF	TAM M2
P164	FALSE	FALSE	0.42	-1.08	1	2.85	-0.92	1.46	FALSE	0.07	0.42	0.02	0.04	0
P19.705	FALSE	FALSE	0.18	3.79	0	-8.44	-0.87	-0.99	FALSE	0.13	0.18	0.01	0.03	-0.01
P20.56	FALSE	FALSE	0.18	-3.04	0	-7.13	-0.06	-1.52	FALSE	0.07	0.18	0.01	0.03	-0.01
P95	FALSE	FALSE	0.15	-1.73	0	-1.63	-0.86	-0.44	FALSE	0.08	0.15	0.01	0.03	-0.02
P86	FALSE	FALSE	0.15	15.63	1	5.95	1.15	-1.55	FALSE	-0.03	0.15	0.02	-0.02	0.02
P187	FALSE	FALSE	0.14	2	0	9.94	1.1	0.77	FALSE	0.05	0.14	0.01	0	0.01
P121	FALSE	FALSE	0.14	-7.79	0	-4.82	-0.66	-1.12	FALSE	0.05	0.14	0.01	-0.01	0.02
P105	FALSE	FALSE	0.13	-5.86	0	-8.62	-0.78	-1.18	FALSE	0.05	0.13	0	0.02	0
P126	FALSE	FALSE	0.13	-3.93	0	7.53	0.52	6.06	TRUE	0.13	0.07	0	0.01	-0.01
P158	FALSE	FALSE	0.12	-1.9	0.16	10.12	0.58	-0.08	FALSE	0.01	0.12	0	0.01	0
P134	FALSE	FALSE	0.11	-7.86	0	-7.41	-0.79	-1.41	FALSE	0.06	0.11	0.01	0.03	-0.01
P3	FALSE	FALSE	0.1	15.23	0	17.48	1.25	1.9	TRUE	0.1	0.06	0.01	0.02	-0.01
P19.62	FALSE	FALSE	0.1	-5.68	0	-5.39	-0.56	-0.6	FALSE	0.03	0.1	0.01	0	0.01
P118	FALSE	FALSE	0.09	-8.46	0	-7.47	-0.87	-2.89	FALSE	0.06	0.09	0	0.01	0
P19.325	FALSE	FALSE	0.08	-5.29	0.58	-3.51	-0.74	0.77	TRUE	0.08	0.12	0	0.01	0.02
P195	FALSE	FALSE	0.07	-5.2	1	-8.25	-0.21	0.02	FALSE	-0.07	0.07	0	0.01	0.01
P203	FALSE	FALSE	0.06	-7.25	0	0.58	0.6	0.97	FALSE	-0.01	0.06	0	-0.03	0.03
P52	FALSE	FALSE	0.06	4.63	0	30.07	0.79	4.26	TRUE	0.06	0.05	-0.01	0.02	0
P108	FALSE	FALSE	0.06	-0.82	1	5.63	-0.29	1.07	FALSE	0.02	0.06	0.01	-0.01	0.01
P19.642	FALSE	FALSE	0.06	4.88	0.04	8.23	-0.26	5.72	TRUE	0.06	0.14	0.01	0	0.01
P19.752	FALSE	FALSE	0.05	-3.61	1	-7.89	-0.82	-2.61	FALSE	-0.03	0.05	-0.01	0	0.01
P19.144	FALSE	FALSE	0.05	-8.88	0	-9.82	-0.81	-2.57	FALSE	-0.01	0.05	0	0	0.02
P103	FALSE	FALSE	0.05	-6.62	1	-0.27	0.56	0.27	FALSE	-0.02	0.05	0	-0.01	0.02
P214	FALSE	FALSE	0.05	8.8	0.03	6.87	0.09	1.11	TRUE	0.05	0	0	-0.01	0.01
P19.603	FALSE	FALSE	0.04	-5.37	0	-9.26	-0.86	-1.84	FALSE	0.02	0.04	0	0	0.02
P19.136	FALSE	FALSE	0.04	68.97	0	103.23	0.46	54.2	TRUE	0.04	0.1	0	0	0.01
P19.793	FALSE	FALSE	0.03	0.6	1	7.97	0.35	2.16	TRUE	0.03	-0.06	-0.01	0.01	-0.01
P173	FALSE	FALSE	0.03	1.79	0	9.3	0.6	2.36	TRUE	0.03	-0.03	-0.01	0	0.01
P19.140	FALSE	FALSE	0.03	-10.5	0	-9.85	-1	-2.74	FALSE	0.01	0.03	0	-0.01	0.02
P102	FALSE	FALSE	0.03	1.17	0	-11.04	-0.63	-1.58	FALSE	0.1	0.03	0.01	0.02	-0.03
P184	FALSE	FALSE	0.03	2.08	1	-1.06	0.18	-1.42	FALSE	-0.03	0.03	0	-0.01	0.02
P159	FALSE	FALSE	0.03	-7.31	0	-8.12	-0.9	-3.08	FALSE	0.11	0.03	0	0.04	-0.04
P231	FALSE	FALSE	0.03	-2.63	1	7.82	0.47	1.74	TRUE	0.03	0.11	0.01	0.01	0
P12	FALSE	FALSE	0.02	-9.67	0	-0.86	-0.84	-2.23	FALSE	0.07	0.02	0	0.02	-0.01
P136	FALSE	FALSE	0.02	21.55	0	8.12	0.49	4.92	TRUE	0.02	0.19	0	0.02	0.01
P166	FALSE	FALSE	0.02	-0.74	0	2.32	-0.66	-0.42	FALSE	0.04	0.02	0	0	0.01
P148	FALSE	FALSE	0.01	-8.98	0.91	-8.16	-0.65	-2.89	FALSE	-0.02	0.01	0	0	0
P19.217	FALSE	FALSE	0.01	-5.43	1	-6.54	-0.85	-2.81	FALSE	-0.09	0.01	0	-0.02	0.03
P19.608	FALSE	FALSE	0.01	-7.75	0	-10.36	0.1	-1.55	FALSE	0.06	0.01	0.02	0.02	-0.04
P179	FALSE	FALSE	0.01	6.61	1	-1.53	1.2	3.61	TRUE	0.01	0.09	0	0	0.01
P197	FALSE	FALSE	0.01	0.41	0	8.67	0.5	-1.68	FALSE	0.06	0.01	0	0.01	-0.01
P97	FALSE	FALSE	0.01	-0.49	0	-3.72	0.7	3.36	TRUE	0.01	-0.04	-0.01	-0.01	0.02
P19.206	FALSE	FALSE	0.01	-8.87	0	-8.41	-0.88	-2.41	FALSE	0.07	0.01	0	0.02	-0.01
P196	FALSE	FALSE	0	-4.72	1	-7.68	-0.58	0.81	FALSE	-0.09	0	-0.01	-0.03	0.04
P131	FALSE	FALSE	0	-3.56	0.93	-2.41	0.36	-0.49	FALSE	0.03	0	0.01	0.01	-0.02
P19.98	FALSE	TRUE	0	-8.47	0.91	-8.03	-0.61	-1.94	FALSE	-0.02	0	-0.01	0	0.01
P2	FALSE	TRUE	-0.01	-6.33	0	-11.09	0.3	-2.37	FALSE	0.02	-0.01	0.01	0.02	-0.02
P55	FALSE	TRUE	-0.01	-9.69	0	-9.82	-0.83	-2.76	FALSE	0.02	-0.01	0	-0.01	0.01
P19.85	FALSE	TRUE	-0.02	5.57	1	8	-0.28	-0.52	FALSE	-0.06	-0.02	0	0.01	-0.02
P140	FALSE	TRUE	-0.02	-9.87	0	-9.45	-0.98	-2.52	FALSE	0.03	-0.02	-0.01	-0.01	0.02
P235	FALSE	TRUE	-0.02	-3.22	1	-0.85	0.34	0.27	FALSE	-0.06	-0.02	0	-0.01	0.01
P190	FALSE	TRUE	-0.02	-6.83	1	-4.8	-0.19	-1.07	FALSE	-0.04	-0.02	0	-0.03	0.02
P110	FALSE	TRUE	-0.02	-6.77	1	-6.16	-0.89	-1.68	FALSE	-0.01	-0.02	0	0	0
P93	FALSE	TRUE	-0.02	-3.91	0	4.44	0.06	1.61	TRUE	-0.02	-0.24	-0.01	0.01	-0.03
P19.177	FALSE	TRUE	-0.02	-8.31	0	-5.48	-0.98	-1.69	FALSE	0.02	-0.02	-0.01	0	0
P104	FALSE	TRUE	-0.03	-1.29	1	-3.58	3.06	1.2	FALSE	-0.01	-0.03	0	-0.01	0.01
P192	FALSE	TRUE	-0.03	-7.92	1	-9.52	-0.59	-2.27	FALSE	-0.08	-0.03	0	-0.01	0.01
P68	FALSE	TRUE	-0.03	31.18	1	7.4	-0.29	-0.9	FALSE	-0.02	-0.03	0.01	0	-0.01
P19.699	FALSE	TRUE	-0.03	-0.2	1	-7.61	-0.05	-0.21	FALSE	-0.05	-0.03	-0.02	-0.03	0.04
P19.607	FALSE	TRUE	-0.03	-4.58	0.01	-6.3	-0.67	-1.91	FALSE	0.01	-0.03	-0.01	-0.02	0.02
P119	FALSE	TRUE	-0.03	-5.12	1	-9.69	-0.13	-1.14	FALSE	-0.15	-0.03	-0.01	-0.01	0.02
P101	FALSE	TRUE	-0.04	11.53	1	9.92	1.05	2.54	FALSE	-0.04	-0.04	-0.01	0	0.01
P51	FALSE	TRUE	-0.04	-0.48	0.95	5.04	0.32	-1.23	FALSE	-0.03	-0.04	-0.01	-0.03	0.03
P19.557	FALSE	TRUE	-0.04	-8.87	0	-9.83	-0.91	-2.54	FALSE	-0.02	-0.04	0.01	-0.01	0
P123	FALSE	TRUE	-0.05	34.02	1	23.32	1.6	6.44	TRUE	-0.05	0.2	0.01	-0.01	0.03
P220	FALSE	TRUE	-0.05	-0.94	1	-8.43	-0.66	-1	FALSE	-0.04	-0.05	-0.01	-0.02	0.02
P181	FALSE	TRUE	-0.05	-4.14	0	4.17	0.03	-1.09	FALSE	-0.02	-0.05	-0.01	0	0
P90	FALSE	TRUE	-0.05	1.53	1	0.23	-0.31	1.02	TRUE	-0.05	0.31	0.01	0.02	0.01
P182	FALSE	TRUE	-0.05	-2.8	0	0.13	0.79	-0.28	FALSE	0.01	-0.05	-0.01	-0.01	0.01
P216	FALSE	TRUE	-0.06	-1.26	0	-3.85	1.01	-0.27	FALSE	0.06	-0.06	0.01	0.01	-0.03
P46	FALSE	TRUE	-0.06	-3.03	0	11.01	-0.15	0.72	TRUE	-0.06	-0.02	-0.01	-0.02	0.02
P177	FALSE	TRUE	-0.06	-10.2	0	-10.61	-0.77	-2.4	FALSE	-0.03	-0.06	0	-0.02	0.02
P152	FALSE	TRUE	-0.06	-9.86	0	-10.64	-0.9	-2.82	FALSE	0.02	-0.06	0	-0.01	0
P198	FALSE	TRUE	-0.06	1.87	1	7.41	0.45	-0.03	FALSE	-0.04	-0.06	-0.01	-0.02	0.03
P130	FALSE	TRUE	-0.06	14.98	1	16.29	0.73	-0.68	FALSE	-0.08	-0.06	-0.01	-0.02	0.02
P98	FALSE	TRUE	-0.06	-3.6	1	-8.34	0.01	-0.59	FALSE	-0.07	-0.06	-0.01	-0.01	0.01
P117	FALSE	TRUE	-0.07	-5.57	0.02	-10.78	-0.92	-2.76	FALSE	0.01	-0.07	0	0	0
P19.3	FALSE	TRUE	-0.07	-6.77	1	-3.32	-0.27	-0.9	FALSE	-0.11	-0.07	0	-0.04	0.03
P19.893	FALSE	TRUE	-0.07	0.02	1	-7.33	0.22	-1.52	FALSE	-0.09	-0.07	0	0	-0.01

P19.511	FALSE	TRUE	-0.07	-9.54	0	-10.66	-0.8	-2.98	FALSE	0	-0.07	-0.01	0	0
P129	FALSE	TRUE	-0.07	39.45	0	10.96	0.53	-0.25	FALSE	0.07	-0.07	0	0.01	-0.02
P193	FALSE	TRUE	-0.08	7.68	1	5.11	1.01	3.76	FALSE	-0.06	-0.08	-0.01	-0.02	0.01
P150	FALSE	TRUE	-0.08	-8.32	0	-12	-0.81	-2.85	FALSE	-0.03	-0.08	-0.01	0	0
P211	FALSE	TRUE	-0.08	-2.39	0	4.99	1.09	-2.53	FALSE	0.02	-0.08	0	0.01	-0.02
P200	FALSE	TRUE	-0.08	-5.72	0	-6.19	-0.37	-1.76	FALSE	0.05	-0.08	0	0.01	-0.02
P106	FALSE	TRUE	-0.08	-6.1	1	0.24	0.45	-0.76	FALSE	0.07	-0.08	0.01	0.02	-0.04
P232	FALSE	TRUE	-0.09	-2.09	0	-7.68	0.74	-1.34	FALSE	0.02	-0.09	0	0.01	-0.03
P45	FALSE	TRUE	-0.1	-5.52	0	1.43	-0.28	-0.64	FALSE	0.03	-0.1	0	0.01	-0.03
P234	FALSE	TRUE	-0.1	1.21	1	0.72	0.75	0.32	FALSE	-0.05	-0.1	-0.01	-0.01	0
P147	FALSE	TRUE	-0.1	67.47	1	88.62	0.9	4.94	TRUE	-0.1	0.26	0.03	-0.01	0.02
P153	FALSE	TRUE	-0.1	9.65	0	-6.79	1.57	-1.37	FALSE	-0.01	-0.1	-0.01	0	0
P20.31	FALSE	TRUE	-0.1	-4.02	1	-0.08	0.22	-0.3	FALSE	-0.03	-0.1	-0.02	0	0
P154	FALSE	TRUE	-0.11	0.11	1	-9.69	-0.28	-0.85	FALSE	-0.04	-0.11	-0.01	-0.01	0
P162	FALSE	TRUE	-0.11	-3.99	1	2.43	-0.62	-0.46	FALSE	-0.07	-0.11	-0.02	-0.02	0.01
P191	FALSE	TRUE	-0.12	7.98	1	-4.98	-0.33	-0.23	FALSE	-0.09	-0.12	0	-0.01	-0.01
P89	FALSE	TRUE	-0.12	3.68	0.08	-2.77	-0.64	-0.05	FALSE	0	-0.12	0	-0.02	0
P219	FALSE	TRUE	-0.13	-5.21	1	-7.39	0.23	-0.83	FALSE	-0.02	-0.13	0	0	-0.01
P57	FALSE	TRUE	-0.13	-7.87	1	-9.55	-0.44	-2.35	FALSE	-0.13	-0.13	-0.01	-0.02	0
P225	FALSE	TRUE	-0.13	6.49	1	-6.72	0.53	-0.9	FALSE	-0.12	-0.13	-0.02	-0.02	0.02
P19.413	FALSE	TRUE	-0.13	-7.9	1	-10.07	-0.72	-2.48	FALSE	-0.13	-0.13	0	-0.02	-0.01
P20.42	FALSE	TRUE	-0.15	0.6	1	-1.87	-0.37	-0.12	FALSE	-0.08	-0.15	-0.02	-0.02	0.01
P156	FALSE	TRUE	-0.15	-5.56	0	-0.72	0.65	-1.79	FALSE	0.04	-0.15	0	0.01	-0.03
P99	FALSE	TRUE	-0.15	-5.8	1	-7.42	-0.8	-1.8	FALSE	-0.08	-0.15	-0.01	-0.01	-0.01
P176	FALSE	TRUE	-0.15	-5.63	1	-1.08	0.05	-0.26	FALSE	-0.08	-0.15	-0.01	-0.02	0.01
P222	FALSE	TRUE	-0.16	-1.17	0	9.41	0.67	-0.31	FALSE	-0.02	-0.16	-0.01	-0.01	0
P19.17	FALSE	TRUE	-0.17	-4.53	1	7.27	0.04	-0.67	FALSE	-0.08	-0.17	-0.02	-0.02	0.01
P19.689	FALSE	TRUE	-0.17	-9.02	0	-12.34	-0.57	-2.9	FALSE	-0.03	-0.17	0	-0.02	-0.01
P4	FALSE	TRUE	-0.17	1.41	1	-5.32	3.15	0.03	FALSE	0	-0.17	-0.01	0.01	-0.03
P71	FALSE	TRUE	-0.18	-0.15	1	5.13	-0.35	-1.54	FALSE	-0.08	-0.18	-0.01	-0.02	0
P224	FALSE	TRUE	-0.18	10.66	0	-2.62	0.91	-1.89	FALSE	0.01	-0.18	-0.01	0	-0.02
P167	FALSE	TRUE	-0.19	-4.51	1	-2.02	0.22	-0.98	FALSE	0.06	-0.19	-0.01	0.03	-0.04
P19.649	FALSE	TRUE	-0.2	2.15	1	-7.02	-0.37	-0.47	FALSE	-0.01	-0.2	0.01	0.02	-0.06
P19.220	FALSE	TRUE	-0.22	-9.37	0	-10.83	-0.86	-2.92	FALSE	0.02	-0.22	0	0.01	-0.04
P226	FALSE	TRUE	-0.23	-5.44	1	-3.36	-0.37	-0.32	FALSE	-0.05	-0.23	-0.01	0	-0.03
P209	FALSE	TRUE	-0.24	-5.01	1	0.62	0.39	-0.46	FALSE	-0.06	-0.24	-0.01	0	-0.02
P19.873	FALSE	TRUE	-0.24	8.48	1	-2.65	1.64	0.12	FALSE	-0.04	-0.24	0	0	-0.03

NOTE

**No benefits**	**Binary prediction of non-response to immune checkpoint inhibitors (ICB therapy)**
**Responder**	**Binary prediction of positive response to ICB therapy**
**TIDE**	**Composite score reflecting tumor immune escape potential (higher values indicate lower treatment response likelihood)**
**IFNG**	**Expression level of interferon-gamma signaling pathway genes**
**MSI Expr Sig**	**Microsatellite instability (MSI) associated gene expression signature**
**Merck18**	**18-gene immunotherapy response prediction signature (Merck validation panel)**
**CD274**	**PD-L1 (immune checkpoint ligand) gene expression level**
**CD8**	**Quantification of CD8+ T-cell infiltration in tumor microenvironment**
**CTL.flag**	**Binary indicator of cytotoxic T lymphocyte (CTL) functional activity**
**Dysfunction**	**T-cell dysfunction score (higher = stronger immunosuppression)**
**Exclusion**	**T-cell exclusion score (higher = poorer immune cell infiltration)**
**MDSC**	**Myeloid-derived suppressor cell abundance (tumor immune suppression)**
**CAF**	**Cancer-associated fibroblast infiltration level (stroma-mediated resistance)**
**TAM M2**	**M2-polarized tumor-associated macrophage infiltration (pro-tumor immune modulation)**

Supplementary Table S2. The filtered proteome expression matrix of iCCA ATTs samples

Uniprot ID	P179	P97	P103	P104	P105	P131	P136	P140	P148	P152	P153	P154	P162	P164	P159	P166	P173	P177
A0A024RBG1	1066264.205	1708393.977	1744952.749	3355005.362	944755.7575	1932807.02	627769.6708	1714415.13	823786.7692	1684651	1687722.614	3197752.79	1575775.548	0	329707.1369	948244.0283	0	0
A0A075B6K0	0	0	0	0	0	4898346.38	0	16890036.79	8363611.477	4928563.162	5330427.118	3442314.075	4988801.748	0	0	1771409.392	0	0
A0A075B6K5	336912.0554	0	3112971.103	5646075.927	10001555.72	9602605.225	21153841.03	6125398.375	817289.0096	7624703.274	1308473.694	5509906.814	6318321.336	18216609.82	11979504.23	32213928.02	5063875.627	8190116.324
A0A075B6P5	0	53169034.53	37877741.08	86034639.53	622285.6594	50869652.88	0	65074505.9	35430150.57	28856399.06	33312109.38	22282209.42	49052116.69	155028166.1	62416773.37	296758051.3	53085194.11	57202280.85
A0A075B6R9	0	5970194.509	3181086.054	2377105.014	11549950.16	2833392.514	0	0	2329328.519	3333934.341	1240701.527	1841671.749	7622569.853	24108292.51	9069589.627	37097834.72	6460578.044	5517560.884
A0A075B6S2	0	0	38076411.01	85486827.57	627793.7023	50627495.31	29531569.82	64461938.13	35339694.66	28443588.17	0	0	49479712.17	0	0	0	0	0
A0A075B6S5	7080784.24	2464530.152	1303232.079	3078361.484	8287613.423	2766946.863	0	3930999.902	982154.0988	4882995.091	3048150.415	3871140.875	780125.9565	10049795.94	7045990.327	25834302.14	5973301.45	3948012.478
A0A075B6S6	0	0	37773970.58	85199980.41	627078.8269	50748054.68	0	64666192.01	35707544.06	28679858.85	0	0	49197034.36	0	0	0	0	0
A0A087WW87	0	53030105.87	37500212.51	84124577.34	625671.6538	50488452.1	0	65714618.95	35517088.52	28796560.19	33244280.81	22245197.67	48341714.84	156719886.1	62089943.55	301680363.7	53322392.97	57349347.63
A0A0A0MRZ7	0	0	37415229.27	85771656.4	626354.907	50995569.18	29666567.37	64864392.35	35609046.56	28505190.38	0	0	49330738.45	0	0	0	0	0
A0A0A0MRZ8	666359.5842	6678039.688	0	14460843.43	2843832.009	11372476.61	12419544.4	9854224.784	0	7925185.922	6382899.899	5266063.546	12243303.02	167133.7537	0	436735.474	55452.21492	0
A0A0A0MS15	1432375.797	0	2102150.349	1570825.469	4177548.297	1353039.974	4808408.154	763676.4956	0	901626.13	746229.7235	1092866.555	433709.5106	3850527.426	2015947.474	13653974.83	1770787.068	1578510.824
A0A0B4J1V0	1025395.262	707870.2611	1985447.282	745519.6546	0	0	0	5578444.948	0	0	1422925.204	2926390.767	3031309.762	0	0	0	0	0
A0A0B4J1X5	0	944643.0253	0	1242352.114	2279305.063	1611673.991	1176510.401	281050.4178	0	2554928.005	2922135.276	668806.6797	0	12097972.7	2425941.473	11516465.11	0	0
A0A0B4J1Y9	0	3816073.289	1987791.11	2150947.684	3947333.673	2872794.894	5030940.742	1155560.548	4323358.737	3143280.613	0	647469.1466	692123.337	2698500.466	753630.9346	7127661.006	1065925.637	674505.0923
A0A0B4J2D5	100141043.5	57160695.76	35543235.1	78562215.04	78773082.47	94067206.82	60377271.73	77852364.23	109136695.8	119635845.4	141028144.3	105324129.3	50059811.79	50775550.23	79445109.09	27479828.59	29241670.81	29619385.52
A0A0B4J2H0	3005500.417	0	1806232.751	0	0	1516128.779	0	4660876.771	0	3176274.252	1832080.495	951316.5718	2818316.783	1330839.802	0	0	3490448.551	2311604.227
A0A0C4DH29	0	1006687.184	1815103.673	0	145673.6185	1517828.121	0	4667234.956	0	3168841.22	0	0	2834295.913	0	0	1653844.432	0	0
A0A0C4DH31	0	2946601.248	2871056.019	3306473.119	63943.74782	3026524.476	1399423.554	5674520.476	1556757.33	5388305.993	2652817.137	1583885.69	3524364.298	1195735.335	2310775.513	22999652.7	2527808.002	349571.3734
A0A0C4DH38	1704523.314	1252156.706	0	3806939.613	3046285.98	1085730.151	3258256.922	1093221.135	0	4705903.979	7133941.901	111015.1167	0	5288832.137	4824504.121	28285068.09	6074988.049	6374948.58
A0A0C4DH41	0	0	7311588.276	10900828.22	8536973.224	19505011.99	0	32856334.35	15113909.07	15419961.56	18680381.85	11243645.91	24349574.12	0	4129446.773	13151181.57	0	0
A0A0C4DH55	0	25704395.72	141186.3066	19505694.81	4360680.652	22260827.85	16731839.17	11445672.98	0	6414375.656	15281557.12	10491961.17	1846067.964	0	1560220.498	12787605.67	0	0
A0A0C4DH67	0	0	1304691.26	3072110.817	8265579.237	2763901.508	3781711.029	3936694.343	983413.9236	4894087.404	0	0	778128.5429	10062252.87	7055133.129	25879500.26	0	0
A0A0C4DH68	0	5978435.363	3185087.236	2379705.907	11534886.3	2836806.142	0	0	2332292.763	3330208.061	1241989.426	1839542.351	7634215.213	24150933.99	9058564.378	37174413.91	6452505.223	5524248.991
A0A0C4DH69	0	0	1301730.237	3075186.668	8276596.471	2769941.234	0	3925042.896	984556.4314	4888766.302	0	0	779191.5498	10074609.22	7063441.084	25919020.15	0	0
A0A0C4DH72	5319411.656	8426961.801	1015032.845	0	0	4001902.729	10558982.19	6690837.277	1906826.618	5400916.29	6996166.833	3045566.544	8354846.038	20927845.36	4073370.436	55997387.85	10237440.58	6655547.956
A0A0C4DH73	5312665.528	8438829.025	1012621.185	0	0	3997335.418	10573048.17	6680576.313	1909163.821	5407401.682	7004314.132	3048817.743	8380036.614	20964477.68	4077953.789	56137959.47	10250661.9	6646727.227
A0A1B0GUS4	50024730.62	0	32299146.04	0	0	27192738.86	44994718.4	35477813.05	40198997.95	42627279.26	41265818.66	38841477.82	44043685.57	75758641.37	35454725.22	127289527.6	63338096.4	46077593.08
A0AV96	881469.1871	953759.5579	171004.8047	1062321.546	556111.5183	439314.3678	1320396.676	575384.2967	839352.2439	1466818.422	1386092.703	1055356.207	992710.1928	674006.6115	953646.0631	3792362.838	1001021.854	1214503.193
A0AVT1	3965454.524	3702584.392	2530524.859	3651644.162	4034853.774	3069002.129	3897030.818	1658747.715	1952853.297	4197640.193	4819082.136	4065149.945	2350160.293	2799347.4	5156115.516	10313116.57	5390959.859	4393332.351
A0FGR8	1743941.262	1601151.441	1373665.575	1537880.887	2047811.258	1629483.736	1334456.447	1080909.368	800716.457	1550785.297	2079777.644	1420437.816	605537.1491	2531603.783	1797988.759	2297008.264	1451112.399	1428206.302
A0MZ66	4899655.622	3453253.573	2852888.961	4411584.129	6447246.805	4934119.215	10407708.56	4287944.895	4443124.392	3533715.859	6870490.171	4199423.785	4083916.103	5616823.426	7380234.558	3080104.844	5425679.598	4958345.674
A1L0T0	22302861.05	22387457.06	17121287.76	22034790.7	29881773.63	13770121.66	5036993.826	3726533.636	13944891.75	27590138.24	24502793.39	28053257.82	6576421.669	16715268.95	26589173.81	16087464.46	35451565.59	17547745.1
A1L390	0	33574.32996	49073.35445	28772.00966	58630.89348	24013.063	0	0	13187.45883	47239.75898	98560.61218	158284.0756	0	213509.7363	189357.7233	259816.6143	132434.3138	283427.0723

O43175	57633633.46	74435434.62	94963493.62	112025401.2	109036272.2	95134192.71	24762257.19	100111068.9	102083981.3	143556262.1	105175203.9	149425128	94074985.24	[illegible]
O43181	9131289.148	9025329.669	7857911.052	5507466.558	13204982.4	20966146.14	13550522.98	11482846.17	18157398.41	15924213.43	11142775.56	21386580.09	11402781.44	[illegible]
O43236	450746.8342	457113.9922	319292.1632	601684.45	456968.4919	220423.9492	297990.0311	103805.5652	255590.5035	294980.6749	57889.41914	581172.3501	61524.82728	[illegible]
O43237	4011312.147	2919136.029	2624682.084	4116356.173	4212766.676	2168875.761	3130334.546	1758175.082	2539118.439	2375250.969	3586772.112	2461551.077	1229294.652	[illegible]
O43242	7679462.606	9919364.934	9175072.3	10044213.48	9370861.944	7141137.059	7524149.945	5336854.358	7678706.923	9635972.563	9639415.408	11319015.86	7354659.235	[illegible]
O43251	197588.1913	108205.0601	0	0	0	264631.5963	0	0	0	416929.538	649360.0078	0	133968.2601	[illegible]
O43252	361549.4763	164361.5009	0	340744.6527	928851.4456	406084.0598	293707.1847	181745.1156	323949.7567	215568.1495	328678.9454	292814.3174	348965.0715	[illegible]
O43264	1652145.927	899160.6998	771959.8668	884475.7423	1056787.443	582237.7246	940865.3705	724032.3393	446259.3145	1055034.624	1468340.503	1195245.664	64284.08934	[illegible]
O43272	116783.1274	195386.362	470378.4132	1040136.073	262461.045	0	0	46335.37683	265348.2459	588079.4996	935572.328	606203.67	195062.2861	[illegible]
O43290	1120066.041	599096.9722	826032.0241	1505286.229	420209.391	2049291.937	1337362.029	789595.6382	1298492.745	1126485.053	1382049.057	1855423.171	1097275.382	[illegible]
O43292	0	0	0	0	0	0	0	0	0	0	187961.7501	0	0	[illegible]
O43293	0	0	0	0	0	0	0	0	0	0	0	24368.82883	0	[illegible]
O43294	2241635.851	756383.4532	2115276.968	2588039.443	2769306.608	3223279.185	792262.9177	1266505.04	696676.6064	1087356.065	934654.8556	1921751.34	280912.1501	[illegible]
O43299	0	87971.19946	38050.38983	16367.58879	0	19266.042	0	0	0	50072.98831	22498.92553	36332.02833	26842.64623	[illegible]
O43301	102296.2804	0	0	58123.72662	229963.8484	205390.1208	121408.4547	147997.6399	0	433864.8246	89846.47422	79797.7966	0	[illegible]
O43310	0	86412.26142	0	0	0	116803.2	0	0	103021.461	0	76858.87525	80116.05194	0	[illegible]
O43312	691472.496	393589.6914	1734568.942	812009.55	1180221.405	230175.3427	242072.4333	149550.3807	287219.8681	720869.6612	289863.7673	1269918.646	0	[illegible]
O43314	87411.58538	0	0	127068.9272	166792.8915	157852.4462	21676.64462	0	100323.5696	296496.095	109952.9465	355454.5646	0	[illegible]
O43318	0	0	62316.37801	169987.975	143517.5909	0	80202.72635	151873.3125	76984.10497	98077.64325	193751.7679	37538.44607	104622.8492	[illegible]
O43324	9206072.304	7248792.613	2099662.504	3815975.585	4819273.122	5252473.407	4672709.356	4388816.47	4015488.237	3463225.373	5442510.299	4102335.91	2607896.143	[illegible]
O43390	14034024.64	5626796.05	3427683.131	9890761.961	8350941.453	5410199.201	15366958.23	9164227.299	8440364.527	4445043.95	9589983.025	10827458.2	9308089.954	[illegible]
O43395	0	0	0	255651.4876	145471.8878	479176.2041	510840.6593	849188.9454	392924.0416	237081.3804	397753.8183	668209.0389	775165.9181	[illegible]
O43396	10894806.96	12846333.92	8591728.852	15760250.81	15175951.73	14127036.35	25717573.44	11144789.41	14395498.39	11504386.75	11231423.35	13393884.05	16145478.73	[illegible]
O43399	15176457.55	10037556.28	15774368.82	14162691.62	21677762.18	24176091.91	28451758.28	20341211.41	17205923.77	13800368.12	13138991.86	18809460.69	16178381.1	[illegible]
O43402	0	319822.8934	120859.7189	489123.6537	739884.0087	726283.6862	1525445.351	0	1184958.845	143254.3913	196103.3113	550662.9589	1958512.422	[illegible]
O43414	1535784.823	1001425.294	533775.0225	1269508.521	975302.8706	1192292.388	780958.3338	728351.8058	0	1909606.482	577372.9918	834449.0272	926294.6875	[illegible]
O43426	53036.84446	0	0	20037.0054	21177.62883	7683.884704	43248.46358	27070.95997	25313.27832	0	0	22734.86959	8591.53407	[illegible]
O43427	407979.9734	209333.3298	254448.1967	99307.44485	35103.48935	231699.0585	83244.58373	64221.75859	118277.9804	0	501779.3918	215635.4204	92872.81135	[illegible]
O43432	47415.66172	35669.62539	35810.21906	48652.13569	18121.6926	0	32555.75374	206166.1602	194120.0281	33041.14465	53690.26726	88764.61443	93695.41994	[illegible]
O43447	1608350.668	901050.0721	0	1298962.574	422205.6805	969263.9912	1121094.847	985280.8306	1346085.739	1632709.307	1359473.965	448703.4839	216210.392	[illegible]
O43464	1227867.219	2651408.55	1691111.84	2334384.031	4355540.655	2744946.682	1504752.49	1680458.017	5078475.435	2822051.917	1681196.944	2210500.651	69421.55475	[illegible]
O43488	42091647.56	46025263.9	51076680.68	57629906.22	52630780.62	33516048.89	26600551.55	24536361.52	36180804.25	52930103.4	32582960.31	52774504.7	20978863.93	[illegible]
O43491	5338320.146	2409191.314	2524170.156	2598905.379	3856603.176	5383839.682	9257388.585	4687443.615	3530442.565	2306492.45	3189812.237	4116233.962	2532776.124	[illegible]
O43493	2249542.667	241421.4612	0	226069.8561	1435254.691	1534633.714	412316.1771	3187021.716	2708375.529	17060.80099	905007.0186	563272.2787	150395.0063	[illegible]
O43504	10350506.56	6886615.61	7634056.318	7456490.919	12451865.9	14022994.95	5893638.463	10301044.12	2020552.837	3217208.897	4544804.281	5112912.792	1172449.374	[illegible]
O43516	0	0	0	0	149668.1099	28924.60066	0	0	120904.899	278495.2553	0	0	0	[illegible]
O43520	0	24543.34335	4220.870155	48383.65661	0	0	127548.0842	109594.7512	14564.44016	0	25562.20706	0	0	[illegible]
[illegible]	[illegible]	[illegible]	[illegible]	[illegible]	[illegible]	[illegible]	[illegible]	[illegible]	[illegible]	[illegible]	[illegible]	[illegible]	[illegible]	[illegible]

O94886	87908.03862	158934.7042	294223.6867	346288.8016	304341.5813	274698.0966	516735.7277	355271.3136	127715.5752	386808.8086	276670.6893	235503.8666	530062.4744
O94887	289397.4188	300559.6743	190840.5162	293623.2592	628486.0842	195817.6964	58806.65408	152962.1327	134067.1597	363200.5297	358325.6257	397176.7888	147979.9868
O94888	108081.8201	272204.271	183953.6431	127901.8303	852300.5855	67292.22051	741033.7063	778934.9785	122758.5924	310086.4554	0	51781.49829	97421.52367
O94901	365164.2903	110342.3931	699757.7879	61019.29927	0	41093.17829	79025.0593	548843.0367	257454.3309	112041.417	213260.3988	389689.9479	103462.0937
O94903	23286507.5	16716178.61	17760123.84	16784077.65	13560426.55	19337070.93	11283397.21	15667966.11	11849127.57	18277846.82	22869785.49	32292877.24	12606445.69
O94905	69409412.7	40206856.34	34028221.81	28395868.29	24597719.61	27556130.81	18115340.34	27223132.65	30197714.38	42522994.23	43242210.74	37243887.3	25475954.96
O94906	852749.4276	532878.538	509341.1683	485003.362	637463.4427	445027.6556	795069.016	1027197.17	675653.6001	599264.6182	912212.6964	532651.0441	188014.2926
O94911	790231.3397	828319.9222	1420970.079	843817.925	1155624.605	96789.85891	231662.3721	102960.6035	687059.1273	706294.3106	794488.7949	1201356.944	220044.8497
O94915	19666.05704	12063.54396	5053.108226	47057.63859	33324.89915	0	21284.91091	5180.774657	0	23548.79833	27532.35092	14219.99905	29861.79539
O94919	1641808.293	288325.0139	446574.9453	805885.5062	1144255.771	709014.0473	1164252.613	426601.613	514736.9065	289144.8313	363774.0828	418009.8032	24194.42311
O94925	675165.4902	122098.9398	541651.6113	386452.2438	255807.1524	790513.8986	693574.5202	551780.5358	230642.6298	59255.00557	535234.7151	251122.3942	292403.6575
O94929	126675.3685	462991.6405	392914.1155	473996.3369	511708.3317	0	11494.70503	31310.8863	35324.3339	351994.8902	377200.0675	428030.0335	0
O94956	3249529.578	4139568.694	5396434.774	6578358.278	4508988.166	768808.3275	1686479.72	5063022.653	4407537.093	4678566.825	4742644.15	7632424.915	2195428.44
O94966	148283.9139	31094.76328	0	0	0	0	0	0	70456.75524	20802.51762	14363.88521	13590.66058	19006.76978
O94973	17406497.17	14975294.15	10486491.67	17277610	15289218.67	10722528.81	9889328.426	4073381.502	7518775.725	14643639.96	16070515.69	14521564.55	7925125.509
O94979	17741496.26	12545244.36	14218694.75	18230326.58	21346559.06	12808624.46	13910428.35	9209897.817	8453216.055	14868358.45	22275019.9	17947135.29	8459836.194
O94985	0	0	0	0	0	18485.22088	0	0	0	0	0	22133.32962	0
O94992	1664831.183	819742.6849	1782626.632	1949165.727	1572267.667	1585751.005	1657844.143	2279722.593	2074771.127	1120546.073	586492.94	3229785.375	2210089.287
O95049	406657.2459	79702.31527	4659252.649	289940.6128	310788.9266	56095.918	0	91855.01558	81700.88044	210635.9412	206699.4613	217003.1368	236771.8696
O95070	4021123.044	3560170.122	1606374.733	2000170.727	3636971.189	1306990.49	990357.2938	0	808058.6908	1807837.165	2786796.566	2502936.758	1064227.436
O95071	59349.13268	0	10988.41887	14541.34774	0	10117.07401	0	0	0	55182.1832	3643.581987	34047.50403	0
O95081	2376435.474	1187058.866	1949966.933	2137452.049	1842916.348	1118355.374	1116293.066	289977.5377	75127.33932	1651955.025	1670712.129	3219326.403	360640.6556
O95104	70979.61628	418042.3631	0	98614.74824	184463.7493	314525.558	640899.391	345789.8263	112463.0063	478916.2765	0	474648.1346	173689.6375
O95139	0	15263367.65	12146747.77	20881011.41	2675788.244	7094932.905	7794221.708	4084430.873	9181373.185	16237676.22	12759080.13	9963574.472	8405499.268
O95140	1285408.646	1180505.713	573230.2438	1121792.603	1452004.854	487848.3165	204372.1922	293916.9674	328125.736	1711525.168	1410455.805	1316670.286	263605.2805
O95154	241883758.2	203079690.1	217230734.2	316151205	277120551.2	172625785	127633227.8	125635385.7	168141107.6	218772538.5	242169889	296407678.1	132985304.4
O95155	60948.70513	0	56868.20833	148535.2923	227184.0294	45710.39328	13614.68056	12357.0078	0	0	66485.38105	140417.902	9746.104102
O95159	601949.5842	511843.1542	974940.3851	929787.8468	102647.6141	284306.3376	297189.3083	1115760.115	798620.116	557920.2403	1323866.426	1212204.4	420807.9444
O95163	435912.8622	451233.014	289341.1162	292605.0559	373967.9694	279967.0954	517873.2732	22435.89885	204781.6725	269944.9598	484510.7367	843682.154	182024.4424
O95166	1157714.834	2046518.022	0	0	0	1464165.51	0	0	4161544.464	5927520.642	10114647.83	10070336.71	0
O95167	4911375.778	0	5937400.946	6137929.669	18237580	4529327.852	8968402.034	7009556.904	5312130.505	15187892.86	9109463.823	10018757.88	595066.4716
O95168	12637831.6	16013601.37	15127548.54	13814517.07	20255318.54	10252712.88	8818895.007	3152559.576	14979155.08	18511755.02	15438480.59	12038616.71	12455932.52
O95169	13144408.87	14004658.91	11619865.05	23461243.83	25394878.72	6390270.677	5454978.565	1663205.407	7483681.761	21835630.07	17001315.35	8093732.049	6820471.638
O95182	1867068.336	11670416.2	8667067.022	10630525.99	2346574.121	12753914.85	8360171.928	17252219.22	12647004.23	23572566.96	18166453.25	13072003.99	835168.5957
O95183	1238589.792	0	0	0	0	0	1910991.088	413799.1725	0	2246227.905	1672452.92	2169190.644	1020457.77
O95197	165706.2335	3806563.766	4123882.466	4736747.775	1034314.336	2795247.573	3219140.425	1866970.7	3108647.507	3569759.892	2827816.054	4092604.584	3712247.639
O95202	5672511.129	4314067.029	2068133.928	3526049.493	3624103.664	2545182.436	3639904.971	3231325.17	3346879.591	4672464.197	3381229.107	4594449.151	2237217.296
O95210	24242381.91	14858843.95	25716817.62	31912677.76	40748951.58	17121956.47	15996350.65	7984352.953	12542573.8	22163718.61	25841619.98	33846360.91	10406329.34

P04921	2441564.834	2211172.403	0	1655684.601	6480707.76	2964669.297	4655546.805	12371994.8	9463524.313	1978626.567	1259556.112	1896419.423	7066334.943	8162832.122	0	0	2134274.381	0	2912843.025	2121206.022	2432227.568
P05023	24287053.26	31868164.93	32550306	39768279.87	31239504.16	26098851.49	28759269.79	29299744.81	25462618.72	30488044.65	36351557.51	38228833.31	30019387.83	25813547.71	34867596.08	45259295.13	28795360.24	29488144.77	36967969.21	31987267.15	36349820.2
P05026	23064109.11	29752097.3	22419032.32	18115282.8	40844051.72	17522082.77	17552615.13	22593190.42	18258242.56	26153947.8	43051673.95	37556660.56	18611010.5	18049325.48	36377783.6	40966160.97	28007659.25	37950962.92	26524601.52	33079237.62	26009929.55
P05062	1218272072	1455089820	1540088173	1542539933	1224086406	1211179459	1230031461	835692898.3	950866546.5	1836870577	1325613199	1762485349	1347089679	615971281.2	1241980032	21843213.82	1530351193	1847004136	1639842926	1604593179	938288154.8
P05067	348397.2738	448459.8729	176465.9903	122091.9083	462131.556	500238.4159	110556.3591	595789.3361	254403.8792	289502.8142	366324.182	220048.6538	469715.3416	458129.4557	335670.4806	668004.5653	285561.8754	78025.09376	176676.9231	666802.4113	467994.5302
P05089	839008228.6	524730893.9	866956376.8	577477403.2	448722567.8	582116728.9	317105990.6	569425103.9	542312368.1	1051741367	962259034.1	1022349286	507563238.1	286309015.5	537487429.5	22594136.47	597601188.3	589411540.6	551972203.5	543061878.7	242916055.1
P05090	59468019.34	52121238.1	50668711.47	37124507.3	31846482.58	40694080.62	48428729.25	69734463.47	62103094.59	62071316.27	56401526.79	41793292.02	58321785.94	48258911.88	47405085.7	43176701.33	47193474.42	38130884.5	40844883.32	13817695.89	71248258.97
P05091	880876017.8	1512679733	1237162191	1428781715	272341034.7	1244646669	778539506.2	1365529341	1214399650	1088616839	1450682454	1451450328	1237224794	313854687.8	512553127.5	26372977.3	294093531.4	1200884792	484461137.2	1365359149	1086617284
P05106	503170.2785	384381.2892	618453.0722	763863.1712	188593.3008	579758.0715	0	111805.3292	498236.4029	1102666.472	1925831.904	945700.8859	305779.7456	4286873.546	529496.2864	485183.9419	901652.2839	1362621.912	488599.6676	926302.2997	1115176.592
P05107	1657395.31	1994430.279	732166.6357	1991311.66	2751183.313	3555016.765	4412673.618	489381.0186	443377.3404	1274297.156	1649977.782	883078.2447	784945.5794	4633053.846	2678111.885	87142321.56	1957041.266	985635.9886	779701.1566	2468002.741	9196598.703
P05109	4952231.94	23006000.8	22989015.9	38143474.43	39627860.47	91458477.5	37971682.48	21511181.64	16494557.38	24073013.49	17108076.23	29468378.62	14021356.94	46964818.42	52654041.01	548135346.2	25853569.66	18877932.63	57859351.31	35870537.12	19235750.73
P05141	106284950.6	50251867.98	42373338.45	90549494.75	119033156.9	36060657.27	31097050.01	52985253.55	58896201.43	43039967.06	56683304.07	50945221.58	62803939.88	41710075.89	95049945.31	69370183.89	46507673.76	43075418.28	48561921.94	45409091.07	32239125.01
P05154	1098524.326	965779.2954	778415.7566	722912.9437	571505.0965	638580.2653	147154.1482	818585.6406	1120263.615	1700463.064	1622902.311	1670630.228	0	985015.2114	1261865.632	4713110.929	2031253.19	1641064.031	1763649.805	2571535.361	426273.1366
P05155	15083797.37	20794575.06	17089342.47	17937361.22	36226890.86	25614929.29	23889840.21	13243791.87	8571383.705	11278273.18	28941255.31	23546730.5	9463257.618	41524268.51	33464337.74	144303788.3	27213122.3	17212292.76	24369218.81	20592354.58	24309332.62
P05156	5422652.609	3928198.171	2514844.59	5514060.122	10815807.51	6895061.02	1800323.818	2957388.016	1703555.011	2801176.707	6217875.58	3131577.232	991409.958	7403857.638	4425663.665	19766777.67	4546558.678	2167843.642	1632895.71	2972935.723	1991901.385
P05161	0	6160011.317	3256330.942	4655188.222	663634.7006	5512710.793	11553089.51	0	2431161.089	7087544.269	4367407.75	7127554.083	1756500.892	11346853.87	958744.0117	3416584.9	5233879.469	4347227.097	7651220.323	1806922.42	18564128.2
P05164	12303460.28	5758206.731	11516094.4	17246885.09	11199252.1	31807862	11503885.45	4752854.707	2618174.168	6389305.082	6522671.13	10519892.52	2957650.405	15999652.27	20769338.07	540897676.8	10522158.08	9044777.393	8338613.581	6995745.769	5504656.398
P05165	21281294.28	23603666.05	18686929.27	27141094.49	12770024.97	20925601.78	27905895.33	15552652.98	21054952.43	37424177.65	25250026.99	31257639.22	16436255.9	24969564.67	25197149.04	6010479.596	25479579.84	23543135.93	25339972.16	22473913.51	35956274.08
P05166	45849453.48	50010409.26	33787260.3	39493023.57	23289503.78	29994741.3	36617053.71	22491330.81	40538162.21	51396445.15	29064279.78	36342787.36	16670053.39	51122149.4	28664023.33	8755952.47	50419046.14	39124491.11	39193396.46	27512427.24	63955298.91
P05177	41611464.73	69311302.79	76768770.9	54758899.78	31430257.55	63731223.71	6703270.815	41329193.14	102995336.3	64855170.03	40342890.53	69695307.88	47749830.87	22716746.2	57666058.25	0	58165794.51	35561358.34	115754312.8	88846228.11	19934202.71
P05181	51255385.38	49646207.9	62691335.57	78116148.96	203034919.8	35811090.59	20882566.84	28936819.24	57467224.76	45108172.9	85165853.15	45831736.16	28190495.24	38030111.44	52311900.57	72166.86873	122826398	66371269.24	29426005.93	31445506.72	36999500.9
P05186	10391736.87	3342912.513	10453913.78	4354905.182	6310952.895	2228131.91	3470034.16	110253.8861	488557.2616	943406.5226	3460658.817	2859942.945	1994809.829	6568782.677	2712763.375	469413.691	9670764.439	2149231.664	1085909.242	9505061.922	4885743.74
P05198	12817779.55	11541711.5	10519208.57	13063742.68	8994003.382	10021718.49	17287760.28	9237672.07	10906541.9	8301938.421	13289981.37	14921439.94	9339265.936	7074052.882	8462490.206	25739869.07	13710298.24	13485550.6	12481499.18	12221860.31	18709469.61
P05362	7911345.012	3994629.421	1560855.37	3537783.538	9687740.987	5444221.044	11453353.62	2089514.191	5185652.663	4047660.764	5121223.392	4224050.613	5039353.813	10720783.47	10375397.67	38940915.27	13380740.82	4754346.582	2993350.772	6662007.514	18469227.84
P05386	235641202.3	271737405.8	230675461	333138084.2	389065139.9	239943882.7	252409398.4	157285410.2	159865978	238720664.6	492795545.8	307987709	137894582.8	439095592.7	303551290	563588541.2	875298150.6	560386111.6	126018857.8	181920666.2	404460487.6
P05387	203512607.7	151995334	164496195	368954392.9	498758648.4	345496360	343928546.8	290788827.4	311309239.6	225474646.8	313712092.4	336944525.5	393584036.2	496011506.8	554250359.3	460557305.9	453559213.8	354277294	217594155.8	251582798.4	305236199.9
P05388	110878439.7	111692764.1	87184849.41	117704499.6	149591232.4	64076142.07	84956468.44	56798104.72	67082676.73	107744689.7	122698114	103351863.8	79458471.64	117447541.9	96729194.15	176054464.5	161456537.8	115577763.1	95671060.95	108023454.1	101259839.9
P05413	10819847.59	7378563.753	8804863.752	11526195.67	10466003.11	7919678.964	25023408.3	11556841.38	7552868.457	5747530.484	13419320.37	9713708.481	6506391.462	24925277.23	10634202.44	1194211.822	14710374.11	6723318.288	9840013.552	13382237.42	13778043.89
P05452	7339505.481	2580039.42	5317634.382	8125819.368	3093720.295	14500966.46	0	0	0	0	4885808.36	7392861.326	0	12941910.79	8172256.39	31557913.81	2285414.814	6244327.177	6794880.952	7173824.254	9278843.092
P05455	15913813.58	18944221.49	12733170.87	19151763.52	15728755.34	22463737.26	24865352.24	15910186.84	15274447.08	15096603.46	21301326.23	19587138	13070887.42	20591590.01	14023932.05	78615031.91	20923592.63	15808964.88	15874376.17	16237519.94	30149887.48
P05543	1246700.589	867740.5832	503631.5097	1723882.289	1079806.294	1222452.673	2115235.661	340173.2746	19581.99167	590193.8287	864599.2819	1643396.668	625052.2634	2629738.442	1030533.431	17521510.15	3113634.312	1010885.072	246262.7716	660380.8471	1858685.184
P05546	2272495.804	3738416.436	4332968.002	4660371.023	7084662.427	5758415.043	5811553.865	3876749.523	1497327.704	4722572.56	5602454.923	4827986.799	1917916.311	6529423.089	4068380.641	20208389.28	12382273.3	3798856.318	7579828.241	9718001.158	5767337.447
P05556	16044365.12	12199633.52	16530686.49	13496543	17888233.17	20408896.81	16817785.92	14281196.94	16756879.69	12856142.56	18924509.03	14688893.47	14749739.28	42148298.7	21984915.85	70841893.44	22931257.15	20166485.46	13572175.2	22638817.4	19866112.03
P05771	0	72329.99241	190544.2795	1017857.904	2392400.607	0	167698.3882	0	42738.21654	0	74880.32201	187575.3174	0	41979.47178	101769.0832	1022931.777	1080303.659	1066793.202	179299.7779	298896.631	1457002.199
P05783	314558834	417991646.3	442648202.8	508225541	520679178	203134617	374048323.5	153192872.7	228617823.8	198779850.6	796080618.8	485886654.2	140663762.3	444435865.5	276769013	1026526785	254833079.3	164797480.5	296682266.9	231223767.5	302217403.8
P05976	7541520.921	5241673.37	0	464531.9183	2495001.707	11175351.06	426162.8165	1222782.539	2863935.852	7884986.272	0	0	1417394.189	0	0	0	2303630.943	3676698.02	6343801.878	2796492.365	11996487.56
P05981	612232.9676	1302494.512	412334.0787	248374.3899	739081.1283	919143.3992	301405.8334	0	352239.9337	1173204.467	3300815.12	1304871.845	898618.631	209002.6281	791863.0307	0	686887.5022	966397.9713	1189900.527	3083197.644	104373.4218
P05997	0	0	9313.854598	0	0	102275.8579	0	7700.779132	0	0	185474.7812	0	78216.90392	2188915.494	95520.00449	4851074.103	416814.5941	0	0	9154.228568	54598.86097
P06132	13439114.82	13917130.13	9082166.594	17052911.3	14281866.63	7278158.627	8349294.122	1922836.652	6793356.143	13820594.5	7773885.6	15204803.62	5793888.405	11523581.62	14954271.16	8359880.093	16249093.97	10133872.29	7458580.568	6122434.865	8168742.738
P06133	96762119.84	60396254.45	83737491.63	81065326.18	93446977.44	38241434.3	94674116.96	59853315.77	64955954.08	88393316.51	79172219.6	54297799.45	57949340.26	21577230.32	83414515.37	0	42565236.92	52291161.23	82143444.59	52894334.23	33856080.91
P06213	320591.0192	214771.4558	214529.4364	303496.0391	176262.2848	147444.1096	276784.7915	89843.9964	62907.09787	361360.2244	339746.8335	205992.6858	128901.9312	530188.0559	557678.7225	47806.61935	557703.0945	222970.3497	360995.5778	511047.4777	303556.09
P06276	172886.7948	173674.7159	233088.7068	657562.2581	654086.3054	1249625.873	0	367053.2774	739554.2166	842293.0829	1326441.356	1351702.607	0	637780.0134	1133895.29	208174.6769	890304.7424	2229670.044	767928.4764	893272.8791	0
P06280	935399.901	809449.8445	79778.04801	355436.4524	476373.3206	92093.16516	1228794.661	87116.83297	90616.12231	779314.966	538651.174	0	0	3608581.222	325153.593	1422916.155	426732.076	0	386232.8282	605121.2425	0
P06310	0	0	37322762.68	84955843.88	624242.0333	51253960.92	0	65936326.71	35891209.34	28618897.37	0	0	48475315.83	0	0	0	0	0	17424923.32	26576173.09	52515496.73
P06312	6214943.636	524303.7806	0	270926.5022	4207351.054	7723562.956	5704054.984	2871204.453	1244097.479	7518089.844	5214757.413	1354598.535	2953653.826	17247071.8	9707372.93	43003308.54	3625679.43	5323769.54	3666130.085	2927125.233	11656668.27
P06331	680801.243	18709469.61	7322363.437	10931265.88	8513973.873	19538822.34	35349218.56	33050527.72	15086448.31	15375627.94	18768448.47	11197487.77	24515790.05	0	4134210.279	13134201.95	4822985.397	3893051.42	10220778.32	11521727.96	48310030.27
P06454	98596553.48	34217034.12	25657244.84	39583451.26	7815474.363	153804798.9	177548201.2	203425490.9	106984801.2	53981344.53	57404039.85	63513578.84	98374185.38	1056872.943	36950035.46	241415804.3	72468993.38	47262888.3	59449660.65	73305468.36	157159928.1
P06576	653387668.7	802150583	653406464.3	914332299.2	774713622.1	379670815.4	307581367.2	163301131.9	477406905.8	806268986.5	555343765.2	694083749.7	406611791.3	468622110.7	762305928.2	307273117.5	918194113.7	764382700.1	326624813.1	392601227.4	479483646.9
P06681	651695.0203	1189697.481	1116030.548	875435.7881	2479444.204	1360061.063	2427638.3	233223.4581	543895.9415	836268.6856	2290789.629	1243778.943	59240.53489	4322524.587	1948023.838	6321215.269	2324460.747	935647.7584	1800075.966	2460076.18	1711710.287
P06702	57938490.88	63172771.84	79962109.07	107961534.1	74209198.78	450660707.5	149844249.8	60051748.23	73301807.18	113906200.8	66855666.7	136339663.8	43080879.86	65281336.82	76970059.91	2147078573	48039467.06	21877375.72	121872340.9	72881770.45	92104729.95
P06703	17219257.63	7267265.82	7188348.069	1583885.951	1085427.431	67977391.77	42306445.95	92605811.97	6257150.181	8025959.283	12354448.93	15569098.27	99747006.04	363131.0271	0	2235625.378	0	0	20291950.03	715494.6845	45203609.97
P06727	3426412.173	4561441.922	6098882.407	8359576.016	13621774.81	23166273.47	6082065.628	22107253.44	17601344.02	6025875.312	4239860.146	6368756.195	12371809.35	17170855.49	1291873.768	118231296.2	6386510.287	4549786.651	7080784.24	5580700.225	7152224.518
P06730	6176972.618	9783281.933	8140137.381	9852199.862	11309728.32	8862577.898	9903878.921	6437987.165	7405306.918	9992011.302	9542994.022	12650892.96	5476115.043	6775510.842	7424624.96	12899697.31	8488117.269	9232061.305	12574437.95	12904297.6	10225747.49
P06733	311366952	302920422.5	449077230.7	625494521.4	538699228.8	409874841.9	388548690.9	237419029.4	353535441.9	604277835.4	521252547.9	583280477.3	341754123.6	481994558.3	346949565	1257237126	519665965.8	459696746.3	424150342.9	473141162.2	452878716.1
P06737	187581375	109274108.4	110777912.1	125726845.5	122673766.6	105287704.3	87684765.34	106373926.4	108609074.5	146619596.6	151690458.5	225890989.5	119217958.3	79665603.93	171412673.9	35114784.27	117082389	205186967.9	136386457.4	168898492.6	94609892.67

[illegible]

P12931	1090253.317	465818.1109	1917391.347	58881.69559	1473343.479	306530.757	498436.9171	0	149654.9509	354262.6016	1140082.401	1631517.537	79211.89615
P12955	19188131.05	20831855.2	13484971.78	16376137.38	21456804.45	11079468.81	14672031.23	9463973.697	7350454.1	12710036.37	20952910.74	29532029.39	6435713.824
P12956	31152349.21	19590003.43	13286454.09	22566968.04	23870130.98	23122272.63	29462330.86	17153513.65	19123791.96	20505742.35	25149114.92	27623014.69	20578222.55
P13010	17648909.69	14515273.35	12033657	18320915.2	19486859.53	14903202.85	20424076.97	11779225.11	13054770.76	13740924.27	14352195.33	16510264.93	15409458.06
P13073	68423572.87	121438024.2	53823991.05	64613698.33	37649800.13	34480713.96	41198301.07	62585605.66	60747766.25	69536593.69	128925775.9	93467848.73	36379827.66
P13196	88729.92647	250983.6163	391390.2328	190043.8553	477946.9051	394896.3729	0	51724.52841	0	937407.5612	583405.0873	732172.6456	145790.2256
P13284	2277343.799	1472482.575	0	864367.704	1741440.169	2329038.497	774126.7435	0	837395.5131	2450679.162	1091173.535	1138200.175	0
P13473	10436848.77	16885077.23	12773046.03	13997676.82	13302913.27	21189454.72	38257248.76	17549808.88	11253809.89	10918119.74	17240290.37	13509297.35	6086693.297
P13489	62110125.06	41219758.73	29105943.05	60718861.55	52896349.57	53896383.29	44102924.99	36621917.41	31574384.38	37163945.99	41987533.96	41610418.79	26606096.21
P13498	5715208.471	2082142.299	1282076.604	9562520.45	580334.6458	0	0	0	877683.7777	18792530.77	1665504.363	4508730.991	0
P13591	2471208.037	219624.2766	48013.86424	936721.7913	655511.5933	14794710.93	640217.3734	266145.9801	145736.7557	91861.21891	610555.015	123514.7548	0
P13598	376148.594	601074.6209	97565.60217	313778.8431	680775.5233	905535.7186	141474.1504	131367.9042	192187.5929	241742.4494	427251.7417	300070.4329	103903.8678
P13611	328161.9616	0	403958.4925	156056.5456	121428.079	2186226.533	2515486.813	1399465.892	260636.9735	0	41106.37071	35493.44004	3948461.874
P13612	0	67772.70407	34117.03184	0	0	0	87501.6067	107140.0597	119238.8996	93574.0297	78212.87891	87030.09793	23762.74866
P13639	117604215.4	105927482	85573040.86	119705768.5	116909052.3	91147705.28	108860816.8	86769357.54	75615920.18	118621797.4	114678911.4	122386609.9	71263661.49
P13667	189000624.5	135176557	122354640.9	163932816.8	160001898.6	139131135.3	195808105.6	124224458.7	146648743	114430194.3	165811578.7	155153801.3	117304926.1
P13671	2088898.98	2807349.1	970207.5737	2596171.723	3705472.332	2344285.325	1671399.648	1557226.118	1368609.897	2088944.158	2427372.232	2311259.555	1121750.345
P13674	20878514.48	2883705.295	8818228.048	4643957.815	16318942.56	6922031.945	2595213.565	1247894.805	2205737.561	2541274.615	1984663.544	2720659.167	1981508.247
P13686	1031752.252	633935.0612	823037.5936	0	0	0	4174518.984	0	0	0	593980.9664	0	0
P13688	764886.5818	45396.65751	714333.42	1150114.06	2772213.809	3614075.308	2349194.303	2021204.178	2427961.542	3546066.417	3750290.853	4955320.429	2175597.763
P13693	38324491.61	26198164.05	41293061.14	44097522.74	44690978.99	45535699.86	24919505.82	11407066.7	23735741.21	44348375.51	32514204.97	77957522.84	13919576.07
P13716	81341392.9	79026519.42	40868494.8	70391474.25	70997499.77	71536692.99	21424143.4	48045532.2	69087370.83	89908739.45	72867915.93	90159408.36	64949859.87
P13747	1787476.304	503535.2894	0	1638490.473	2152245.899	321212.6578	729959.1373	868630.8879	151455.3266	500643.7185	359908.9735	275725.4831	401565.8548
P13762	0	1592367.936	3202284.681	3500322.771	1512837.204	117202.0507	0	1312682.289	0	1242670.167	0	0	1213503.674
P13796	82356828.55	60050122.72	29584822.39	52873991.52	65778908.56	73966168.07	115513569.3	58399912.07	31733052.68	38015423.85	60833045.08	51189479.58	29858919.97
P13797	60611806.25	26091129.86	42592882.06	58384902.66	59657824.96	26971004.62	30129541.89	22735089.57	24263963.4	26248468.66	31574384.38	29654957.82	40590171.74
P13798	20368012.46	23100212.05	27335150.81	31378475.33	31634593.47	20589060.8	15915410.84	15609057.12	18489732.1	24026528.44	21591428.89	28274476.64	23518793.05
P13804	246093739.4	228641515.7	149310003.3	204218646.2	269722304	120881434	219872778.1	132526398.3	198874120.8	325870585.6	171812140.5	200152785.6	170756694.5
P13807	2832603.213	2826332.173	1675302.774	2718690.933	3164339.815	1553030.049	1572816.906	1629734.639	2487769.222	3950025.409	4277405.766	4175692.345	1337966.484
P13861	33298812.28	24030379.2	17656508.15	22158997.37	22356992.01	14085753.13	15835810.16	16188460.85	19544719.6	23617356.95	28272587.46	23979863.97	12182707.95
P13929	7225476.202	114874443.3	85245081.51	64951078.11	27745687.06	75549038.53	65037337.05	41673723.41	96750935.99	127468970	175869982.2	172026993.5	105045694.4
P13984	0	0	0	0	147204.9059	285391.6139	358938.9867	231759.6611	0	0	161549.8546	339717.4188	0
P13987	7870718.943	12619705.48	19817887.69	34884982.2	3301505.014	31593612.01	67921446.98	20512728.47	23413462.77	22957170.08	15106460.38	17434755.42	27976653.64
P14174	768853930.3	290993932.6	246574371.2	257040197.6	729759583.3	1073476608	116638099.6	138143163.9	49080410.43	128710337.6	176976841.3	316632928.8	53269331.2
P14207	4705308.546	6584385.595	0	9790180.96	8888437.452	10337540.09	6721522.312	2997673.01	655998.5401	2208593.917	5666121.566	5064452.412	2113660.34
P14209	12728318.95	9237176.456	2800237.775	2162634.964	118202.9198	25830980	8016377.885	38154120.12	27060957.46	18344417.5	16606889.88	17894201.81	6050438.841
P14314	173365917.1	99217060.13	80540039.92	156575140.2	124989020.4	135501016.2	134791376.7	115386396.6	133893788.7	114920856.9	144531854.5	128460634.1	110002150.2

[illegible]

P24666	10971236.38	11188872.94	16903453.79	23287679.32	32693305.2	16447950.36	21506855.86	15295456.13	17108076.23	16810731.89	14332778.75	22004145.18	18037443.21
P24752	1085433945	819518781.1	410768097.9	849121656.3	759377179.4	701855580.7	439654458.9	456977545.8	453094753.7	862416938	1121887567	829772286.2	742273523.8
P24821	2989130.344	2473234.949	4000681.003	2277173.625	5307238.356	10223745.94	5519492.875	2177514.464	2270768.499	910989.7497	1944013.5	4069609.749	9044777.393
P24844	12214549.51	3334963.122	11337261.07	11617436.84	8804674.064	21920366.78	13030056.67	7698004.201	6291984.85	4934656.167	9261019.449	9554491.491	4895736.122
P24928	309655.589	145461.0974	508089.5448	206247.2839	201173.5315	212072.1132	237163.5141	113353.6038	88483.13499	237736.9178	179808.3493	302977.4197	212639.0195
P24941	67069.56458	527435.1669	321465.5255	786988.281	218992.4804	241474.3358	133480.3711	104277.3033	269762.6368	435872.769	375449.9277	68705.06839	0
P25098	353486.6422	29686.07672	84093.24736	233256.0216	192725.7456	189509.4882	168669.4063	58955.17126	34730.85895	39598.74129	184028.3232	178170.9056	52821.13901
P25205	5516.728159	21552.21025	63131.06045	129998.7882	122619.4127	111783.883	460150.2925	196701.4975	69564.25766	11743.945	0	70838.8283	77452.40113
P25208	155937.5687	0	431895.4332	416132.6678	1934455.729	0	522799.3178	0	310270.9992	0	640026.9873	153404.2837	962596.9088
P25311	16666275.91	17088937.54	25144538.53	25569648.18	26516264.49	30385938.85	27970123.56	21241285.67	21929466.97	22911719.25	24914928.74	29951726.72	20088230.95
P25325	237633072.5	363493437.4	222889654	386478091	267155957.3	210864117.4	185154802.4	130876297.2	239948811.7	234667423.8	451690170.9	573677419.5	229264991.4
P25398	109975848	90011454.93	55510164.98	102389313.2	130586362.2	133435423.9	79172219.6	90009196.2	76706809.38	74250484.12	111289717.1	95096888.06	57404158.11
P25445	2373859.219	1588899.298	637819.5153	550299.9019	314743.1645	1257558.483	4616621.959	3397193.765	996549.7419	1865936.383	2103692.701	1029661.536	814489.3446
P25490	0	358849.281	899750.9493	880928.3384	139061.7407	529235.5296	228505.6641	809152.0138	712973.0895	465620.9242	0	529831.6059	300283.5549
P25685	4146118.829	4431368.354	2758932.878	4168359.348	6968666.999	5156963.672	4638341.936	4164544.746	2349771.596	3235856.934	4377700.751	6066642.942	3557757.662
P25686	438361.0314	158190.2067	119950.3172	450270.4217	893598.8179	229592.7488	163872.5776	990231.4981	370873.5051	422235.6192	554126.3483	360667.6001	490393.37
P25705	269030256.2	268958939.1	234996093	310187119.7	296418606.8	246348801.6	230634077.1	193618296.6	277488412.3	202915111.6	238134098.4	329759913.4	236116885.8
P25774	2110009.932	5457028.793	5954491.805	4406741.017	6037509.864	6399252.634	46950655.48	7548269.848	3223866.5	2670196.006	15895800.64	7832804.847	6151779.568
P25786	36093063.4	29291624.03	24653665.25	40035328.06	42373925.53	32148880.68	27060957.46	21640165.82	26613353.36	28220359.92	29294670.2	38930254.19	22724337.2
P25787	67318523.16	59060308	36272685.97	55852641.6	94468631.16	47693798.93	31866017.01	22993644.71	37856767.79	74918746.59	67212416.53	61242730.14	24628537.42
P25788	18477828.81	45442777.32	28184934.09	38051376.55	28154725.75	40588650.9	52687319.91	41002113.5	39028966.19	46431446.64	53063082.36	52303244.59	35696606.38
P25789	28325282.96	38297102.04	24979434.72	25269746.02	25534512.22	28852230.48	26483728.4	12789884.76	23889840.21	35196663.82	27498452.24	34050732.91	11843238.68
P25815	2751885.632	1978419.046	220707.268	2814911.115	142660.6038	10674332	0	5928507.324	0	0	2252466.041	1752892.634	1831196.546
P26006	0	0	0	1456.823836	0	22633.46125	0	0	0	0	0	0	44062.09627
P26038	39887860.02	58909730.65	47849779.36	49421540.41	52155382.46	59947397.22	94317063.37	55386877.68	37067267.27	44879397.68	65799557.1	57778844.18	30725742.89
P26196	3152154.616	2173748.488	3027974.43	3836812.996	4442978.847	2903248.427	3365115.151	1671826.92	2169275.155	5066385.148	3333733.829	4239028.013	3330190.493
P26232	1130980.145	964656.4642	681596.4868	0	1637011.515	343988.985	816344.8169	343842.239	365361.1794	1026116.294	0	1104094.97	287047.2952
P26358	2146872.027	552087.6204	0	2186412.639	127629.9676	0	910975.9314	0	0	0	298024.3533	1178311.445	13452.45569
P26368	11607955.76	10913405.57	6346690.76	11854884.77	11767408.63	3665186.652	6195319.736	5555127.357	8101834.724	5642207.681	5837908.902	9215603.942	5968779.697
P26373	79759796	27268141.07	62129563.11	67833567.29	44905764.91	33661871.71	40382442.31	23887940.28	18782078.75	40986260	40996234.41	54850193.63	39803136.35
P26440	72182228.66	79815437.58	55055644.36	71038596.13	36545311.53	52190282.21	39505410.12	30733853.68	55373453.05	75160827.44	50163398.34	75483022.43	22989674.16
P26447	25611388.97	13186040.11	12673103.46	16114529.46	52517866.1	22895223.15	19920122.58	16374234.27	8766597.247	15120143.62	14593302.29	4588996.279	2090969.689
P26572	2928512.538	2117977.235	2120065.432	3155472.04	4529269.685	2906763.593	1405983.821	1508614.004	2801209.799	3024666.25	3673846.793	2913633.104	954994.0921
P26583	0	7650270.044	12896085.81	11773724.12	24278052.88	11290198.98	28157065.57	21863389.67	16848366.07	8222726.163	19017604.92	9021174.019	3118970.103
P26599	47050835.28	41506180.88	27918763.36	32545238.64	47431701.38	21762373.85	25990087.36	12663528.38	20022490.05	35040366.74	47998381.56	36656144.9	19386138.67
P26639	10094675.71	9664135.188	8403447.317	10099996.87	11848257.65	8313856.86	11470203.45	6197246.593	5659415.732	11071457.52	14886476.3	12893885.77	4303785.472
P26640	11435673.35	9303285.589	8566566.484	9247254.857	9661629.782	6556886.278	12401074	7804712.686	6752836.715	7556737.809	11642811.45	10584271.63	7657111.511
P26641	53462535.34	61751624.66	57517987.23	59907613.08	66875810.72	47580630.23	65224360.78	23677187.69	34719629.38	59758256.99	62257465.05	53271987.11	34854543.37

[illegible]

P41226	2908065.735	742814.5395	1235995.749	2167307.529	2301536.437	899865.5635	1794248.716	764611.1237	747092.686	1998370.487	1338211.197	2094149.225	1661259.471	[illegible]
P41227	581141.9431	649085.4282	1449907.583	848241.888	1970789.317	900770.1055	2133937.498	634327.0291	789654.499	197729.2781	925171.555	1194038.622	173031.789	[illegible]
P41235	1203295.109	1216332.468	1237533.223	211860.4444	2030189.188	1674991.489	831685.5354	887172.7299	180064.2916	461431.4035	450450.8589	1468506.273	941826.0221	[illegible]
P41236	0	6704714.85	431357.4607	0	63074.06469	133064.5479	377396.7887	233555.6974	0	2281589.472	2633056.45	1282926.258	48860.24956	[illegible]
P41240	5286426.286	3366181.26	3551340.615	5573406.874	5144305.67	4927912.859	2996572.839	1367396.361	1686850.341	4404418.681	3472695.784	6177505.133	2873126.541	[illegible]
P41250	9448092.139	6128448.112	3726358.935	4923881.265	8627340.88	6150923.524	7120290.104	3648752.919	4364537.995	7160972.456	6695860.516	9678217.698	5234027.142	[illegible]
P41252	4883072.286	4029033.323	3694272.764	4575114.079	5853171.424	4172991.09	5400072.822	1577518.372	2690668.65	4085638.044	4131190.37	4621453.607	2522780.305	[illegible]
P41567	34533457.56	21755768.73	26532914.3	29896981.63	55051980.97	36148964.87	35589257.86	15639012.14	21618241.09	31608091.51	27723690.2	34410518.03	10032728.86	[illegible]
P41743	325803.1566	0	79958.10461	166360.3764	158970.5465	0	66053.30116	0	0	133950.4613	0	191816.8844	0	[illegible]
P42025	4314707.086	5869874.505	1557129.367	4793780.98	4970325.019	3261412.21	2553645.955	1058633.113	3373773.139	5368369.518	5220814.866	5236594.24	1624728.09	[illegible]
P42126	66571402.13	90333420.09	72785550.19	86913411.5	74001090.32	82842222.72	64673822.66	52106893.88	51348516.32	91164128.3	143785193.9	122852256.2	53610404.97	[illegible]
P42166	5773821.376	4009099.623	2937669.398	6311697.507	10128595.37	7941045.662	8026842.816	106891.0591	3950963.922	1193187.52	3100391.81	4248235.139	3754704.636	[illegible]
P42167	2992460.734	1865238.203	2463225.128	2064326.345	2527995.522	3268809.429	3911762.471	10939708.73	2830040.171	10459645.72	4935160.779	5170518.588	1788720.729	[illegible]
P42224	15656002.32	14226108.72	10106453.1	18145228.25	25128413.25	9068269.488	26949194.95	15381390.54	7008276.793	7794502.44	14182955.08	12840532.13	8433120.043	[illegible]
P42226	1381704.91	479692.0271	1054483.376	1133530.572	1693525.296	590339.3362	902142.4059	532191.8858	313941.6818	1088434.219	1198564.836	685040.2464	113662.7691	[illegible]
P42229	106697.4841	0	62641.30502	59872.9141	164588.6951	114064.486	1385684.169	141809.3064	0	27435.27354	0	90033.99718	19317.59394	[illegible]
P42285	647533.2509	627988.7875	356354.0509	706932.4782	557310.9453	523912.633	649036.6969	308936.4301	128931.0565	647161.8535	662529.7217	746213.0827	0	[illegible]
P42330	25050228.43	68710899.36	79402614.12	55277890.06	69134576.01	55037524.44	55696442.2	36731831.29	68392516.54	92093236.32	56529654.16	151896504.9	24683541.71	[illegible]
P42331	1338421.215	673290.589	137584.96	405596.4409	746192.5855	849431.8302	699346.3427	687802.4981	427503.5854	192528.6228	422381.8924	330134.3571	172398.0767	[illegible]
P42336	56883.77501	19926.10133	0	80006.10714	69083.48202	339569.616	31789.32928	0	0	34300.10308	67643.04212	29086.50495	0	[illegible]
P42338	170367.5008	0	129956.7341	103168.6655	65157.87875	0	48077.35637	0	0	31295.74705	16004.80724	76588.18545	7181.385989	[illegible]
P42345	391078.9238	293333.5007	283298.1717	588158.7133	636744.5485	256480.1367	220365.7611	80863.51205	188248.7116	430314.7183	274332.4896	704544.4814	78618.54613	[illegible]
P42356	348829.4939	373398.7423	173603.4254	221110.9694	648600.9393	153604.3673	247042.2767	99404.99452	274038.8551	242399.8125	759268.4818	292117.4614	130208.4194	[illegible]
P42357	15132176	14769002.63	14481957.82	19731142.66	3193869.234	9526496.745	8091710.729	4908210.16	10818270.99	23761582.38	16196184.84	20721327.91	4408898.274	[illegible]
P42566	2119132.637	1332025.774	1049373.769	2221908.51	2389881.919	2205606.095	3361256.113	1995691.574	1464422.364	1031411.078	1946011.79	1884208.303	1781739.61	[illegible]
P42574	2309898.793	1712317.161	691977.6305	2319150.608	1801488.839	2822988.249	2642684.069	226068.8909	3076095.569	2964216.944	3677844.711	2251490.894	1003783.508	[illegible]
P42677	24951002.88	37220682.16	16177178.1	9826904.885	0	16645201.83	31496585.16	38797057.92	41772857.02	32750077.78	21918026.73	43880019.94	9756145.591	[illegible]
P42685	44915.2951	450169.4692	0	0	154573.97	257752.3533	219435.7015	274961.2392	154639.1843	440303.6672	1001030.395	288197.858	0	[illegible]
P42696	0	145014.9822	0	31329.96543	57072.72103	43855.36926	0	0	0	0	87162.57478	28349.8062	0	[illegible]
P42704	16911434.84	17118938.07	14998411.02	14762750.03	7705282.384	14479747.12	13238242.21	12454124.72	8237324.755	16862547.87	19175455.22	20230102.74	12309583.23	[illegible]
P42765	367493285.5	749754535.5	918115857.9	1260007462	845787297.8	644904892.6	378997793.9	860141678.9	819801828.5	1595420218	304299071.8	1243809299	870750533.8	[illegible]
P42766	0	21284169.35	25440487.24	42805796.55	1832600.528	72863478.99	57273457.05	57500168.8	63510816.29	12691855.81	154951080.8	99608410.05	44296526.91	[illegible]
P42768	283921.6327	124578.6277	249085.9986	743921.2422	1244582.904	878609.9928	609976.0675	324122.3005	167091.9459	38072.14274	161085.3745	400676.2023	32369.1372	[illegible]
P42785	3790161.146	7109133.621	9160624.735	6940118.634	7906537.232	5803345.327	5554537.064	396745.5479	3072444.119	7204695.306	7116820.475	5457524.358	2362619.382	[illegible]
P42858	538479.1237	328993.2851	222646.1146	350051.5314	629142.6908	172662.4031	353158.894	122420.0117	106921.853	371860.8832	472326.8262	481692.4456	29415.23904	[illegible]
P42892	309259.6097	366694.4639	443269.0338	406518.1294	751081.4313	471540.9883	206080.0103	253187.31	413208.4138	473051.5022	546547.0829	615073.7037	346386.1514	[illegible]
P42898	108271.8753	0	75155.55345	64288.61711	0	105809.6407	71941.55347	46178.61525	86922.21428	52531.73588	48722.15863	112657.6214	55800.40016	[illegible]
[illegible]	[illegible]	[illegible]	[illegible]	[illegible]	[illegible]	[illegible]	[illegible]	[illegible]	[illegible]	[illegible]	[illegible]	[illegible]	[illegible]	[illegible]

P53618	14095178.5	14471745.17	11014595.21	14184211.3	23913086.47	10308639.94	13259712.65	4746158.729	8960385.43	12798940.84	13824928.83	16698782.85	10478105.18
P53621	18859652.53	15046627.79	13416008.71	18454061.52	24324773.35	15961535	17987649.67	10118891.73	9819618.038	18191023.12	21059063.23	18747658.11	9963619.568
P53634	6593985.506	6215972.838	6918870.198	12064450.12	10664217.59	10528305.59	11152186.14	10672273.99	1019841.941	4853221.726	9203609.678	8102836.201	3018532.773
P53675	0	52136.08004	0	44860.12115	0	61811.69516	51349.78424	0	9195.68927	0	77019.23703	27160.84731	0
P53680	23463118.38	20675555.93	12812357.93	17375025.48	26253324.47	14438500.17	17047614.67	12878909.33	11029371.34	16556595.75	25890038.75	13740876.68	3892257.718
P53701	3294375.759	1181779.934	1361211.502	3298755.299	3428547.713	1384234.375	2744687.59	1347826.795	1995316.91	1984963.351	2992772.912	2656817.583	2258999.006
P53814	0	0	0	0	29275.69448	0	0	0	15947.18764	0	26507.27758	0	33300.24628
P53985	8686001.932	8839536.704	6782858.692	8297772.54	4751362.763	6547603.061	9339243.235	16766518.29	14800196.12	6552095.192	5971058.396	7110447.774	13528980.81
P53990	5954848.247	10259929.11	6723949.177	9726641.257	7067434.875	8158796.224	7594544.075	5093062.778	3355739.866	7336059.467	8794411.995	7726200.353	3678190.76
P53992	7608855.758	6668744.704	5515489.595	8055762.913	7915395.131	4573063.945	6941175.873	3527646.484	4370824.834	6909629.728	8347178.222	5874667.64	5240828.555
P53999	59954101.46	34532457.37	20586852.91	29655471.41	26684466.57	30187802.02	70227631.73	57856990.83	37274794.25	27804488.3	28493012.04	42440433.09	39364146.53
P54105	3628516.307	1873808.502	2565087.418	3325020.252	5326402.559	2215367.604	2384025.342	0	1347638.652	5683133.732	6282970.202	4752047.644	0
P54136	22931519.54	15386503.83	9687225.931	13876112.19	13479979.65	11046340.76	13216511.74	6584253.669	9328580.399	13842337.8	17269193.03	13797457.48	10668098.42
P54252	278694.7557	0	0	0	0	57080.99879	0	169729.945	0	295368.7474	0	0	0
P54289	1254469.486	348746.2844	337413.3599	1598957.208	1388921.745	802002.6527	438106.1563	683647.7939	59542.61255	530333.9725	584047.4975	795330.6449	216899.3637
P54577	6744242.942	9199047.459	6108406.333	7286586.424	10755489.22	4229972.318	8081375.939	3472661.701	6662502.981	6284391.331	8940856.671	6682359.371	4551719.08
P54578	7829851.828	7211823.199	4261042.883	12897966.84	11642786.05	8765302.862	11135110.29	6370723.726	7438081.259	11022152.56	7812253.004	9457544.217	6652588.464
P54619	11783112.5	8937816.224	8515757.928	12990679.29	10255534.63	7786356.644	6932485.068	6802853.442	11404377.43	15144564.99	12536961.21	14583909.29	6673914.157
P54652	1683135.816	140752.4903	1498524.717	462903.2661	288546.5157	1663898.571	748786.0447	349280.7329	0	551645.1372	262065.759	447667.3157	97887.77721
P54709	0	0	561473.7175	576268.1188	0	270215.2133	1882408.662	47092.21142	148377.7338	289849.4688	722067.249	223119.2577	73143.99904
P54725	6346191.325	4612301.824	4759043.178	3213891.387	7587359.25	8753909.753	7006073.437	20426165.87	11308753.9	4700464.502	28601703.56	9178639.175	12223065.07
P54727	59160683.4	31501811.65	34862954.53	40695289.4	51240584.94	43898480.57	41696147.88	46058700.24	41987533.96	39754391	47110865.11	51587428.58	23202427.48
P54760	0	0	0	43465.88188	102822.0902	0	0	0	57660.5037	29943.83118	57750.9065	0	0
P54802	2189982.055	2727191.055	1935728.469	2049372.888	2249291.976	1790485.424	1790133.746	579835.3812	888473.0764	3198568.962	2664457.428	3787881.983	904263.9671
P54803	0	116071.3697	0	61468.39088	0	144746.0447	0	0	136930.4315	0	0	188856.2027	81579.86471
P54819	101715306.1	134400699.6	65659019.02	111064898.4	127848049.1	87664650.36	98419099.79	47901333.83	80060055.73	120695007.7	99835285.33	94451396.14	81651484.98
P54840	14590690.57	30212192.19	26723573.05	19941664.12	20870430.95	14858706.52	9031760.051	13807505.26	22076697.12	39021642.59	21879873.42	36574926.05	16529353.63
P54855	107717989.7	17298955.45	87883214.91	106093443.5	151330321.5	8982901.116	66954997.63	88205185.69	88062392.62	76273177.73	58419694.83	77270229.96	103559256
P54868	860209933.2	933659815.4	1015037423	1199002106	992417148.1	730330222.5	274783655.1	975432006.8	981612386.1	1160773156	812526713.8	796883388.6	995370487
P54886	2002227.717	3178181.634	2257448.969	2464700.087	3157398.333	2062641.912	1844079.328	1349512.699	1080420.133	2341941.133	3771188.686	3055427.781	1610238.246
P54920	35603899.08	30909424.92	28245100.93	40984750.69	34817921.45	25418063.76	28389741.93	26019160.26	21182246.66	27987348.48	36918800.42	30323697.67	19873200.52
P54922	792735.502	104482.0872	0	0	0	0	476373.1058	181438.381	0	0	286413.238	300787.7245	0
P55001	203436.7605	0	132058.4987	467615.5255	724995.2122	2178859.037	1418550.505	2926375.182	0	0	2748416.693	0	0
P55008	14747237.15	18271227.52	10747438.47	18894926.31	14879598.2	26806427.38	12365497.51	27498588.76	6605701.028	11166654.42	15463355.32	12380693.77	0
P55010	10714914.28	6136039.311	5381914.89	10677057.21	8045058.449	5214114.171	3749441.941	4726263.987	5171572.295	6883871.852	6624897.275	6756853.653	3225448.873
P55011	49316.72283	0	66516.47907	83984.03288	94077.16724	129004.5445	400468.7648	170023.1697	58692.21321	0	82286.94131	16372.188	139032.5342
P55036	38977128.29	9863046.173	9458556.596	16348794.15	17439437.83	16583619.09	14447677.8	5107968.732	8739650.641	14174357.88	12865654.96	14623362.8	10100270.35

P82650	825912.8059	1282661.316	457514.9716	938802.8864	585966.1754	762295.9307	585611.6649	66605.98668	146542.0425	2574721.603	1463987.687	985743.7122	540754.1294
P82663	818515.3389	4388923.632	1908449.899	1594031.015	2404811.285	1820415.151	1489838.348	93656.39249	0	3395595.046	4792510.255	1364894.742	2550173.784
P82664	0	0	1824186.968	1373455.341	398944.3862	1726979.944	0	0	0	902607.9748	434898.7988	1061446.468	0
P82673	1491661.172	1014124.611	393396.2365	905176.5545	672980.4942	754839.7148	411839.6866	112931.3068	578656.2446	1328544.685	1421608.64	411887.8226	423138.4751
P82675	254883.589	489039.1967	523937.8732	157903.5434	148801.0893	83052.69075	313158.9595	35171.94395	276477.3385	483631.6742	813047.9708	560923.4369	0
P82909	17710977.41	9050939.246	11193266	22684745.79	8502672.289	24598412.9	8844424.011	11684888.67	11697596.85	24628793.39	23123225.48	29183671.66	8473000.41
P82912	502026.2068	1704549.274	1787047.895	2224401.248	1819087.469	703598.6928	554702.0832	0	0	243391.5254	1495264.794	1373033.108	0
P82914	113706.9755	0	0	730347.1191	0	421354.3678	0	0	0	325348.9834	124452.7061	225125.4038	0
P82921	0	988483.0166	341534.7204	0	0	0	740290.9373	0	0	0	0	545006.3095	0
P82930	0	892934.4839	1212637.794	496736.2659	638909.4026	1084546.297	387948.8154	225011.9498	332264.1335	1636362.768	2687717.056	695603.3269	460161.5384
P82932	0	1507216.154	0	0	0	783617.5173	492650.7827	559921.2256	625772.4844	1549130.655	1988657.728	458572.8329	0
P82933	474328.9492	949721.4003	959736.1284	972920.8149	582745.3313	342355.7864	80897.68743	93481.42689	183135.5445	805673.5459	1135437.566	793506.6542	39228.28975
P82970	1213474.198	407379.7339	601901.1824	1455640.163	1966612.357	1945568.982	0	1859983.621	1757777.354	1837642.533	1004862.174	1898391.353	410069.3812
P82979	6153241.278	8017971.941	10357649.65	11953214.84	4222863.249	19272820.61	16217546.98	36334054.07	19694184.36	13300339.02	19204990.5	11273913.83	14694986.62
P82980	8835168.091	18613645.84	12916013.75	22854938.17	4723556.126	15254577.83	3539762.846	1998350.281	884071.0347	38739420.55	18531295.23	31769620.49	1149081.841
P83110	313200.1266	0	0	0	0	275048.6436	0	0	0	0	407928.9581	0	0
P83111	23331104.6	20755766.06	15973100.69	21503556.58	24415129.76	13262742.03	17643640.34	8747166.166	15438480.59	19954082.48	18249096.42	19557401.45	15677637.76
P83436	75929.31212	252643.2601	61561.43573	234717.8896	237582.0922	166352.5356	222206.944	132837.3392	57022.92483	114490.8949	5782.145582	143658.8576	35745.38491
P83731	27639004.64	29481388.4	14414106.55	15094430.3	19961614.19	18250360.53	20031004.64	13976359.43	7071792.71	25115194.14	36585013.66	16356514.76	7563205.999
P83876	0	501777.7752	461617.6372	1460282.5	0	0	317225.8507	165108.7768	322499.4719	922639.3696	399516.7098	1389043.366	122275.2483
P83881	15268757.25	7549940.711	0	0	932762.5934	3728080.058	0	17329416.51	8945684.071	2697241.663	12156071.25	5683701.439	5010566.123
P83916	1567605.293	1633851.488	227988.8137	1747710.922	1108907.042	3136466.211	1274749.994	865330.5457	4038382.339	1509467.937	1453830.334	1424508.607	2674883.605
P84022	0	386681.3108	132267.5105	0	124555.9929	0	24944.86231	155654.9342	0	171818.5489	108168.4826	216453.0878	520224.7639
P84077	82924009.77	187965636.1	986273.5995	3492397.321	951568.5462	73302343.9	80805931.43	155146386	93145843.03	158796306.4	248220529.5	163110254.9	159630901.4
P84085	120163272.1	3535230.747	1715799.376	3068616.041	4267659.602	3839552.861	25831216.45	0	3738217.268	5361558.098	1646801.82	5094822.66	0
P84090	21024684.76	13309664.66	22149840.66	38768985.58	46117171.9	42883642.81	39028966.19	28522441.66	22699093.98	24968181.8	33851408.79	25846543.74	23359398.89
P84095	9473529.049	9085371.454	6898901.222	9960464.859	9279818.249	8147824.337	12642820.66	4590342.641	2944942.571	4218031.472	6491887.141	6855756.065	6208044.636
P84098	64737267.98	30839100.92	24033280.68	37782381.94	26360103.36	28609404.76	31933904.71	55553666.73	31973800.68	29405618.53	32443633.6	28603254.4	21562493.57
P84103	5597457.525	4863151.149	0	4590568.726	20028672.88	6522842.972	4869155.539	0	0	16674035.07	0	0	0
P84157	1555506.54	1011918.29	552364.0835	0	875177.114	692491.0283	882239.04	2111560.744	691372.4936	476292.1781	444035.1493	229184.6079	751861.2679
P84243	298893105.1	122559127.8	37222435.68	214868087.2	10647127.86	81246005.31	116063803.2	97438274.11	54905713.41	79604580.6	49478300.57	35203151.51	13312367.04
P85037	0	0	0	54469.39909	0	0	181499.1565	0	0	0	0	85762.03456	0
P86790	745417.5025	353602.883	59287.67392	0	37849.63334	0	0	98377.96302	0	90993.92445	370402.9508	402086.0469	217577.5695
P86791	744612.9831	354096.9454	59433.63607	0	37720.89945	0	0	98169.14314	0	90828.50883	370019.7969	402499.1727	217216.8941
P98082	928319.1123	286012.6738	686333.1159	838625.1412	1390296.97	1246926.289	1238636.461	393779.4032	0	189112.8156	945999.3868	273399.7717	83467.61307
P98095	726962.8725	162643.4308	309323.3367	642186.0943	1113772.577	1676806.565	2181400.08	1029653.395	93983.31141	145642.4484	779412.0347	237638.8993	699609.0829
P98160	8084430.239	6904166.334	6704832.084	6520466.404	7597090.817	6245012.012	6640694.513	4107730.574	4678431.164	3232044.193	8232647.312	4440348.128	8315937.943

Q13418	7799885.662	8814114.654	3698616.574	6871327.434	6684156.981	3636131.715	3965261.832	3668251.26	4770063.119	8552800.289	14866738.8	12299091.84	1743250.546	[illegible]
Q13423	115400863.2	78775561.6	70363039.35	99323824.52	72721141.97	39828212.79	48547420.63	29670153.93	39559188.37	74044446.82	90039946.81	78806329.62	33865522.6	[illegible]
Q13425	0	426530.8897	538366.6693	651917.0959	853210.5641	559970.9646	225343.7533	441858.933	107837.1179	433303.8521	897771.7429	432249.1377	503088.6408	[illegible]
Q13426	658160.4517	221448.2783	0	0	36565.33931	219528.0511	474629.1248	102767.7084	380255.1774	273609.3524	765543.8113	296952.7227	118366.5991	[illegible]
Q13427	778699.0508	542105.4277	640146.9344	641536.5445	343352.2525	533510.1123	536349.6783	163461.4906	204088.3861	605258.8199	896889.7234	1066619.865	304882.2434	[illegible]
Q13428	399152.6114	220225.6097	185084.3996	230260.1003	114931.6688	396470.159	682212.708	707626.2991	420307.624	388191.8543	728575.2436	759977.3163	163837.8829	[illegible]
Q13435	3271746.538	2736887.078	2831402.783	2901628.716	2251753.936	1958871.408	3456875.574	2985699	3419511.785	2625617.469	3276112.077	2651018.297	1984005.503	[illegible]
Q13438	2666601.092	3027650.234	1901599.885	3381800.551	2954966.19	1313913.179	2124526.708	978167.3914	1518399.991	3541837.01	3028035.479	4430270.078	1140574.956	[illegible]
Q13439	302305.4522	74249.84314	48773.60883	107413.149	228467.4578	84676.48091	1546352.937	65875.07478	108504.897	98767.04661	214958.7408	99424.94591	63760.59346	[illegible]
Q13442	7619368.664	3677348.28	6695156.716	6350435.635	7521220.976	11631572.63	15390312.57	14001932.48	14540813.8	3908738.066	7986942.587	10597956.97	14308756.56	[illegible]
Q13443	51358.74989	0	0	20278.50339	0	37042.98319	0	0	0	0	0	0	60632.47065	[illegible]
Q13445	4649750.381	7733236.378	4480816.167	7000977.612	2436528.279	4255270.587	4725233.474	1822098.398	5274469.783	10205716.39	3693594.605	8254852.888	5467371.34	[illegible]
Q13451	7779591.03	4814902.316	3454210.336	4601607.864	6234935.985	5101420.579	5719827.521	5028190.827	3517216.679	2535465.493	6116586.747	6641531.835	2933654.591	[illegible]
Q13459	0	83899.72292	41876.19522	147158.5032	172922.8018	99553.06241	216396.4054	76869.69342	26592.99563	28964.10929	144746.0447	174579.6021	63580.45141	[illegible]
Q13464	454290.2423	301713.8288	154997.5881	488608.267	219891.167	6307576.138	533920.3838	106003.0698	334101.3573	9291378.699	386382.9492	492538.5741	146350.7327	[illegible]
Q13469	158546.1066	0	0	86020.53662	113058.6182	0	0	0	0	157364.8318	46441.17189	69661.96671	72959.8724	[illegible]
Q13485	634389.8525	379951.4603	0	0	0	410780.9958	0	474984.1563	1043911.636	538574.2995	304037.8148	726461.5257	397678.6946	[illegible]
Q13488	276228.5562	466889.9422	188275.3314	341103.1063	675834.4166	794953.6384	732314.6706	492685.3612	96193.14982	260101.3001	615555.4998	401594.8113	693007.7757	[illegible]
Q13492	20027340.91	13080897.01	7097908.045	8327352.369	12059417.41	7357897.172	7054584.383	4217306.614	4466922.733	7684542.615	9822471.099	9226779.103	3818451.267	[illegible]
Q13496	1265086.681	1382745.04	1295725.474	1497213.098	496555.5584	1100088.554	503128.6862	587352.9527	528191.6787	2000581.209	1444833.048	1798143.248	600617.8501	[illegible]
Q13501	2764285.822	1256116.601	1300189.568	2125943.937	1150339.213	13359600.9	5675219.122	1162627.032	2033151.606	1717256.199	2259852.468	1209809.079	2304300.866	[illegible]
Q13503	0	0	0	0	0	1046881.952	162625.4764	0	0	159038.3865	157192.4607	449717.1471	275527.8522	[illegible]
Q13505	3394619.35	2420333.52	980668.2008	1072987.579	2420684.709	2295064.605	3465766.794	869740.2245	1073561.016	2622734.312	2390273.027	1717351.507	781134.5063	[illegible]
Q13509	626465.5401	0	267994.313	0	647177.9019	3093456.324	330072.3806	419296.0497	628272.7919	280490.5001	88736.3297	786235.4582	211919.9838	[illegible]
Q13510	38238781.73	24807890.67	18400411.83	27824778.65	27340455.87	15935198.95	50051963.15	10537424.91	15300067.6	15976262.9	21197123.93	20683351.75	19831922.36	[illegible]
Q13523	21345.59792	211695.5811	112140.3561	71494.90514	230270.4933	258123.9223	53676.27146	164023.0533	163434.0019	239591.0325	432423.5867	246897.3511	220406.5828	[illegible]
Q13526	6470620.104	6626688.015	7364948.296	9918942.236	14993893.53	11028921.99	6131103.071	2741994.367	6068315.464	12098077.27	6779542.758	11259014.58	4853558.019	[illegible]
Q13542	5403275.511	0	4435537.95	7465773.986	4603686.837	6931092.963	3423375.849	0	1984888.894	3363746.262	6196061.898	1518833.674	3366400.294	[illegible]
Q13546	1304412.795	575564.0623	1005859.39	1106518.441	1027912.379	1036563.505	1420077.107	511840.1177	619414.0348	1010098.121	927935.7936	1588940.712	266052.4073	[illegible]
Q13547	81439.83773	343485.7828	693601.5301	862573.4286	1972653.294	760518.1331	1530749.139	1054798.775	1342796.415	780926.2601	211581.4633	1088272.859	0	[illegible]
Q13555	456880.3475	72992.64672	226617.8315	552215.0363	1585502.482	427092.0988	199747.6949	0	38094.02516	175983.3333	225598.0368	0	0	[illegible]
Q13557	6867375.471	4802924.572	5122116.22	6384099.464	7377663.834	6275801.397	4290320.599	2208445.224	2611611.826	4596458.17	6014588.203	8162248.768	2759314.979	[illegible]
Q13561	10336565.8	5471521.734	13845995.13	13596795.37	9647970.355	14458366.68	12606162.24	12108462.38	9776644.812	8764951.2	12486295.45	12182550.33	6005749.635	[illegible]
Q13564	1978156.358	1891409.042	1321323.032	1369053.22	2992101.554	2713943.857	1230298.165	1690606.795	1233201.4	2507520.892	2001609.408	2025700.707	2135390.883	[illegible]
Q13572	0	0	326874.9455	206825.5668	0	0	0	0	294833.657	374969.7714	551702.9832	579377.3109	379885.247	[illegible]

Q15848	0	255722.2343	960857.2887	200557.2639	0	776963.6552	0	0	0	1662774.723	906794.0736	443405.851	141902.0842
Q15904	0	0	0	233812.5066	568342.9858	346498.3016	531454.8773	0	0	0	0	387982.8007	0
Q15907	34620252.62	22176738.69	18052868.51	28286475.59	21829113.85	19105817.84	22680643.6	30421064.88	24981427.73	21915243.79	27060957.46	28001927.23	20761475.49
Q15942	3127522.924	4567083.834	5058897.399	6880002.663	4957277.583	6984284.721	8581699.456	2562084.396	4595344.596	5762593.296	9178816.943	6574230.063	6244536.733
Q16134	72851112.37	96103909.33	66284461.55	67110496.25	48830153.19	40103288.85	47676663.95	59660487.76	65371739.66	110421104.7	58703373.52	91067720.71	55414598.59
Q16181	22552541.56	21917773.04	20716425.81	20847262.43	19994409.79	18678411.42	26107211.15	13053817.83	12176533.76	15666864.71	18306812.83	20154250.02	15268223.38
Q16186	9625440.819	5182541.885	2889223.937	4277294.824	3979067.221	4119563.362	2333423.446	3555795.042	1118855.281	1737785.755	3829121.495	2919801.135	4060754.99
Q16204	765724.0282	357515.5252	0	674339.9211	424996.3551	571342.6269	1622583.147	680486.1771	1093929.238	179546.7611	108347.8772	163266.4433	649568.9947
Q16222	5780804.79	3989789.495	6849577.749	5808933.086	6166473.469	3111390.925	1802267.631	2724058.411	3364192.318	8016007.993	7947904.541	8034420.842	1472854.028
Q16270	3702609.271	950761.2298	3189306.172	2601745.107	3271673.927	6539503.718	6959581.951	5484055.339	1551036.784	1016392.193	2036608.968	3602370.618	1950723.797
Q16363	197313.61	99786.90713	235426.01	40028.50641	136683.5954	453422.2102	423198.3665	417052.6063	85232.45706	18746.8264	215245.7162	30612.53387	341031.4791
Q16401	13006863.9	11142133.16	15153429.38	19249724.6	15267176.57	8837888.186	3892016.23	3979250.049	6149154.207	14582524.23	14225537.46	17313163.03	5622991.885
Q16512	57171.78142	74081.35075	103325.3885	259579.0923	186456.781	183736.3185	130866.207	99179.43846	0	44484.51442	893967.8835	195188.258	81179.14045
Q16513	342564.424	287602.9519	245606.9806	297641.0746	303597.9024	127516.3002	19399.67213	108272.3742	95433.56382	401059.7489	229661.5988	241172.7941	80982.75513
Q16527	4448488.233	923931.2211	2986876.773	1472467.398	4555933.247	5728468.611	2052437.265	3037235.06	3784247.383	2887208.941	2353701.164	2561548.5	1179727.296
Q16531	11418255.71	13022744.9	11426680.38	10766355.49	13641789.63	10006420.81	8534359.153	5329279.586	6946168.738	14263893.96	14763673.47	13246802.02	8084921.685
Q16537	1691708.263	350486.7575	557927.8765	833437.1442	0	517922.9549	272428.8049	325073.7549	226406.7519	356692.8061	1050439.278	564961.2683	353562.5694
Q16539	3603126.671	1882327.917	3495135.981	4182435.391	3416105.866	2708012.847	2512457.099	799679.6795	862483.5608	2382861.046	4056602.16	3524886.442	1762994.495
Q16540	824128.8609	1312411.829	0	999429.4848	413723.6283	654200.0637	928281.7549	0	0	1168564.524	1576177.672	467504.0536	0
Q16543	7063328.814	5422868.245	4144990.362	9839615.184	7946289.457	6229341.232	8721999.978	2285478.734	3307151.978	6032241.626	5770179.39	8666222.768	4250812.538
Q16555	48586119.39	17961388.5	23478921.92	33370880.14	38265085.8	22676607.03	45571468.09	25230244.91	19201553.06	16700818.11	24865352.24	24334350.41	21788970.99
Q16563	6168693.725	286827.2457	1248028.975	1804186.313	5275165.307	5860274.908	11117726.49	5945393.449	1736342.039	3633058.816	6468923.13	4454722.769	2243352.102
Q16576	3102165.848	2768773.766	2842421.599	5389569.221	5575424.881	1184628.147	3246550.09	340633.5727	1712253.747	2123566.871	1781842.382	2426017.133	1863007.084
Q16595	4738291.358	7891978.765	9529880.057	11193042.94	18969142.67	8922011.246	9848171.276	3604605.757	6386521.696	9523015.346	9433477.077	12616619.24	6555414.228
Q16610	588201.8002	481272.9658	314152.7548	597285.302	1194059.793	989354.9696	40551.50236	503133.5693	516034.2475	668800.8743	534680.3376	884758.8749	116670.2143
Q16611	137362.6547	459780.7883	425486.6403	586919.7798	921185.1011	298486.3969	430232.8021	377873.1464	856144.2205	619366.8975	233660.2538	0	686804.668
Q16625	3669725.347	1007793.209	1161777.517	1640265.027	1363797.13	2009288.35	2472678.866	3682697.127	2818534.862	2121282.418	3373773.139	1902575.605	1551072.739
Q16629	11932338.89	13664571.2	13372314.36	8754666.686	16136097.75	13832977.62	27294602.35	20161090.64	13195144.64	6936145.554	18593912.31	17375682.12	13674673.47
Q16630	2697011.422	1696903.928	2231490.72	2691763.534	1976661.314	1599531.305	1812726.386	1453344.485	1759865.014	1275633.155	3236471.764	2999765.681	1470854.373
Q16643	1421763.717	52864.93444	955027.0578	878199.8001	971002.8852	2393751.591	2285557.245	1785215.398	826932.5438	517439.7192	528509.5111	402921.0459	1011130.38
Q16647	143169.2139	470744.9225	779337.1282	369177.1206	94752.77086	613256.67	2524257.071	1039937.852	213384.6668	409055.656	200628.616	0	1010027.355
Q16654	2619542.315	719859.9868	2448276.463	2096562.08	1540624.321	359834.45	649755.2635	715703.3948	0	952279.9452	1081247.238	2808195.863	230507.7858
Q16658	6727009.333	13349000	9947627.386	14481589.26	20803109.91	11742928.15	7584314.676	6554789.597	7461157.093	12322010.04	16740575.7	14837082.57	4445717.681
Q16666	1834610.937	787640.1291	1173229.145	1212385.137	884241.2728	2060469.806	3608805.378	2180312.846	572762.7382	970870.5344	784718.8645	523154.9277	1678933.245
Q16695	26836400	204039.5394	0	0	0	0	0	0	0	80120640.83	19734847.55	13837298.3	13334475.63
Q16698	252655855.2	189379496.4	163319359.8	242082261.6	159126137.1	180818383.5	192891695.7	120370942.2	205495326.7	135613187	152532202.5	166932987.2	251445373.3
Q16706	2422944.989	1947314.08	1475798.746	1853522.279	3377421.455	1770431.176	1829193.616	952391.8306	1727395.856	1891030.153	2821430.186	2500328.887	865717.362
Q16718	16360787.56	40109757.41	39406812.5	40122450.8	47316089.79	17615302.16	17707608.42	9012240.073	29176631.08	36731948.52	32033084.67	33643700.97	27635638.26

Q6IA69	331351.949	1741642.671	139987.267	1013388.823	610979.3433	176359.7912	1327413.862	296508.3278	364323.3049	556657.858	1645078.09	1374454.931	1104465.98
Q6IA86	414104.3225	279059.9856	187660.8655	279066.9458	442205.3628	35163.0672	333788.3958	21700.52196	0	451632.111	426828.0463	245103.8477	95383.50515
Q6IAA8	4418113.581	3016685.565	3164712.885	10295469.44	9944898.572	4594813.055	5127975.942	3157154.873	2388648.328	3379785.069	4880273.387	5346165.83	1734209.105
Q6IAN0	3320929.818	3634719.169	3120643.222	4101955.828	4101114.825	4611998.664	4003235.292	5344512.516	1882151.204	2405640.926	4896233.043	5605865.389	2865370.426
Q6IB77	140800960.4	258816785.2	171478949	160028923.5	166908832	130600313.1	47432856.05	110676273.6	158836525	452268263.6	206735537.3	380336549.7	104077397.3
Q6IBS0	10377781.58	8392574.976	8897562.114	11649667.11	8592949.131	9811474.192	21942791.05	5860004.54	6854167.472	9317464.16	9626941.748	12263933.09	9631153.235
Q6IC98	643912.5233	348293.7146	114209.2426	751916.9889	1379294.768	328113.1148	133041.4142	123124.6371	394464.9303	689417.0285	1092312.815	429005.0587	28350.75234
Q6ICL3	814918.6753	460871.4501	620019.2231	398193.4052	1232650.516	778808.0396	727553.6469	0	342122.5157	330886.4959	1103555.235	0	0
Q6IN85	193200.7498	188991.4358	75689.01813	281402.471	411914.146	426162.8165	385166.9066	422754.093	48707.41711	396386.5686	452417.1538	431740.4127	95971.01355
Q6IPR1	1377675.33	4463743.854	2190979.756	3109540.489	6641618.198	5429957.21	1732901.905	2745546.968	2535805.88	6944461.619	4529445.148	8580207.285	1439388.518
Q6IQ22	442469.0392	2023703.872	405037.8652	714496.4242	593128.4383	526987.7104	346098.1139	392768.284	293225.3834	1093255.204	862033.4203	567338.6282	364988.708
Q6IQ23	254206.9277	730964.704	236821.1754	280354.4042	320857.9306	327321.622	70642.5582	176053.9705	171068.7236	678811.5888	966428.4493	990759.2847	137466.7147
Q6IS14	58699564.78	123716854.6	154184604.7	189768272	279805774.3	132023541	147132810.8	49881112.35	93891487.11	183362397.7	120149324.2	143290304.8	122411761.4
Q6JBY9	37580.86868	0	126879.5711	174531.1331	249673.0501	1214421.719	2031550.202	284421.512	189745.9423	237414.5928	0	143458.8743	143424.8236
Q6JQN1	1312627.736	2898125.784	2612378.458	1711024.994	2124786.794	1687722.614	1025544.398	928941.1945	2101801.822	2238499.235	1706707.936	2176502.957	905402.2716
Q6KC79	13902.75243	425081.3742	46701.82627	368750.5359	33589.7043	724810.5764	174294.4186	50591.83943	8815.654284	15198.19219	571328.1626	749387.3616	192093.1922
Q6KCM7	710762.3992	104683.0722	0	549726.8145	244763.6045	0	0	39294.0118	103653.4601	714675.3284	611200.6905	390879.3866	0
Q6L8Q7	1684972.139	1618629.469	2150803.51	2872282.817	2787708.429	1986887.015	1777853.511	458228.4759	1236064.067	3815500.881	3134229.603	3499975.222	1088712.446
Q6NSJ0	76255.5869	630231.8894	291440.1432	314558.5306	89705.18397	246374.9544	164354.3838	304424.5752	209705.4678	483123.6108	716140.1416	687041.9234	417282.3269
Q6NSW5	2565790.771	3478159.297	875479.4497	2017074.61	786230.2125	840379.009	1317716.504	1132427.033	467449.1668	1813914.302	1371356.58	1860942.389	542871.5106
Q6NT55	169348.5346	176340.0539	23902.41907	60038.70885	74214.23227	12597.36772	0	0	13331.15658	69452.34594	10829734.11	181617.7075	12831.68808
Q6NUK1	571624.2544	323132.5066	301632.986	1045841.842	1098181.35	1764719.576	1033359.267	148260.9414	416477.1816	203016.84	1033258.432	128378.9033	522253.61
Q6NUK4	550307.3175	326939.2013	183658.6317	539858.3996	748691.913	0	0	0	0	208280.9773	141474.1504	382310.6346	0
Q6NUM9	10634996.23	5796691.357	6421283.174	4912532.1	9825021.222	2614276.891	3674274.211	3098084.225	3999116.185	11487910.51	11401132.9	5047710.795	3506253.417
Q6NUN0	13105071.97	9556645.003	520679.1326	22397464.32	7865549.43	13708856.81	4211716.372	10317068.89	11606127.27	19544138.97	6414765.697	6285719.683	7120655.047
Q6NUQ1	293486.9881	133047.3259	43641.51595	148309.8759	134269.4078	21225.30878	65744.18513	0	119467.7045	194992.0278	347809.3755	246030.3764	0
Q6NUQ4	1357886.172	1159613.214	4129320.203	1433011.258	1077481.999	757319.0862	590523.672	251298.2734	1508600.252	1276950.195	7472389.671	1857301.491	6338004.571
Q6NVY1	25876681.75	39355552.27	24601422.64	34287814.38	35577722.66	36917033.16	19220879.89	25772654.66	43051673.95	44664716.42	25055294.05	41332813.18	41757241.45
Q6NXE6	251315.3742	445816.5461	0	455241.5541	920176.3261	250820.5429	509084.414	109805.7051	78850.60234	463452.8412	656781.0117	501507.9154	627644.9967
Q6NXG1	0	0	0	0	0	0	114741.6331	0	0	0	256622.8101	0	0
Q6NXS1	0	6713984.881	430822.193	0	63217.67265	132853.3123	376969.8826	233900.1124	0	2279150.964	2630465.539	1284233.595	49021.47709
Q6NXT2	0	122031728.3	37123284.29	216619150.1	10633021.87	0	0	0	0	79857984.63	0	0	13358604.73
Q6NXT6	677157.7295	617909.9901	1092044.166	1203640.563	1269166.675	476510.2514	213935.6487	738136.5941	756430.7427	725582.2605	1367171.937	1290974.823	651231.1854
Q6NYC1	0	0	0	0	73449.28279	127943.7123	0	0	53024.80017	0	0	0	0
Q6NYC8	404733.534	97530.17072	388522.7209	141614.7392	937840.9332	1589314.11	1541094.772	953451.3066	507438.2182	174191.4621	31455.37177	498864.4005	309504.7475
Q6NZ67	350883.0085	0	0	0	1396147.66	0	0	0	0	0	0	763200.8249	0
Q6NZI2	16071622.9	15827711.48	14505274.78	16532530.54	14147926.88	15831144.74	10998123.61	12727063.13	16121375.49	11198286.35	13401066.3	10957840.45	13504489.73

Q86XE5	9409606.078	19873671.98	20266220.33	17856027.15	6718272.888	13379952.66	1407661.464	11722335.26	13683783.69	25479852.54	11611184.33	18778638.6	11094684.36
Q86XL3	0	0	29822.28411	31204.8381	22173.73067	0	0	0	0	10150.55291	0	44243.26251	0
Q86XP3	1036106.635	617207.2349	814840.1662	1666663.58	1257457.406	963966.9803	413758.1583	400012.2781	997764.119	690928.6659	1025134.986	1230977.423	982698.6679
Q86Y39	14793603.25	16857382.78	7087224.882	13456159.63	5281521.392	3844021.906	3477974.498	6360831.453	1725222.561	20869592.45	21995788.15	18383566.92	437919.2055
Q86Y56	0	0	28561.30333	54606.413	63505.10357	246960.4212	84922.11379	0	838363.0883	44880.67194	57363.94854	62932.25222	53985.50973
Q86Y82	1123534.28	3861234.071	5196705.487	4731218.826	8339679.246	7058724.022	8593990.328	5690562.114	2522688.677	4172679.157	7345635.31	5254060.136	2941182.99
Q86YB7	142983161.4	77105644.55	57856513.74	97471311.28	72054489.93	66855666.7	39984812.15	48197552.76	71534302.74	121767869.1	106890821.7	88713312.89	44549849.11
Q86YB8	373884.6133	657176.5953	213207.276	1189787.084	1267946.07	269901.0914	286788.4685	90639.75044	147282.8364	467171.6304	755460.7035	269618.0603	298931.1792
Q86YH6	45046.04775	215698.3564	417962.7076	679098.6495	574723.6069	129895.8985	53969.18103	77801.62711	0	817226.2729	684980.1236	500496.0469	0
Q86YN1	6762929.389	3125735.082	82703.82733	2521450.507	4350715.376	3326750.655	829854.6687	645575.5701	1499186.968	6874939.783	4451082.891	5463897.357	234525.5122
Q86YP4	0	65700.86378	0	0	291896.0452	0	0	0	0	0	80715.98141	0	0
Q86YQ8	164024.9506	0	69353.35079	76531.44735	212351.0133	0	59273.7527	261792.827	244068.072	223725.637	164441.9461	234910.1967	0
Q86YS6	1922914.702	760472.4282	616193.176	1256525.453	2154564.752	2130675.745	873455.3259	6711126.732	438586.8288	1448392.781	2247639.449	464525.5562	348447.7866
Q86YS7	0	175009.8863	78513.63449	202993.2296	0	76976.31355	212073.3185	151125.2745	391864.066	183973.1727	201110.6121	445768.9303	112788.5262
Q86YV0	217505.6663	44716.06139	31866.49079	91300.26745	96296.45581	106561.0622	132378.3589	2200.88011	0	94565.29043	13982.17812	12736.83949	0
Q8IU81	138868.5927	0	92367.87511	85251.77459	0	0	0	0	104332.7003	0	0	160109.8998	116210.2673
Q8IU85	493725.8178	337599.2971	1399052.708	688079.6807	914469.8983	881425.7612	200603.8203	44465.25328	130426.5412	268271.6931	2053545.299	2510649.911	372308.0521
Q8IUC4	100314.6883	0	0	100526.218	0	0	0	0	0	31540.56645	52638.22564	95263.06915	44850.13003
Q8IUD2	733776.7534	437415.5233	544205.0257	436210.3239	705129.8443	721556.7656	616919.8707	394301.2884	171360.3038	709335.5959	641911.8218	983907.0489	296751.6724
Q8IUF1	0	0	45355.03008	0	0	78592.90064	0	0	0	43849.01285	0	96053.4847	0
Q8IUI8	1558730.473	462007.2085	67201.77894	400425.4765	990562.0124	880441.4755	452644.6113	553286.723	256375.2576	659278.7015	547641.7328	477274.589	0
Q8IUR0	402845.5895	0	0	107036.0967	858666.0951	387631.2811	591200.8257	482467.8184	0	0	1019005.824	1109769.971	0
Q8IUR7	138435.2898	0	87541.54429	177016.1245	160876.6621	80923.43722	0	30143.16992	36195.19045	61856.05843	430101.2538	162581.5724	68008.17469
Q8IUX1	0	245073.9131	506180.6875	420793.55	190101.8731	248888.2489	0	299950.1822	320717.4835	0	192187.5929	557902.9679	427654.2279
Q8IUX7	458981.0784	236223.5939	1877858.588	644277.6846	598334.7129	13545093.67	1448834.482	4241765.563	1158477.606	390791.3873	362929.6605	650122.3874	7767015.734
Q8IUZ5	11000168.51	2752800.4	5359647.276	6682815.648	8168315.325	1899391.677	2793242.081	3968568.012	1937820.953	9330563.212	7073594.834	10153960.53	3128063.399
Q8IV08	3088131.539	718255.7001	2878137.355	4570000.884	4217657.013	1950098.636	15701441.79	1449580.072	0	4144136.484	1614539.938	3662104.855	183342.1127
Q8IV36	19425.11475	0	152211.9773	0	0	697913.8033	0	27347.06688	0	0	0	0	811272.0211
Q8IV38	66618.15844	206347.8174	316319.0106	67485.2153	0	87500.1056	204684.6066	66211.42371	357205.5091	0	117358.645	113874.6163	0
Q8IVD9	1165938.763	136059.993	660812.8492	607159.842	842420.9086	534102.1089	519218.4543	417556.7697	208312.7957	502254.4552	280580.7649	655477.505	694900.1628
Q8IVF2	3721.916719	13833.26414	28284.01445	2620.868537	17103.59633	18879.32247	27737.34888	1456.823836	7474.637045	0	0	2072.960254	17470.47842
Q8IVG5	0	0	0	0	16699.95643	2110.707325	11970.15108	109157.4107	0	0	0	0	7604.376842
Q8IVH4	9434890.898	6481589.193	2967892.406	7580526.345	6197193.785	4158523.712	3289871.035	2430028.052	3035912.4	8440773.493	7831351.668	6797621.822	2246608.331
Q8IVL6	106326.432	0	0	0	0	0	0	0	0	0	0	0	0
Q8IVM0	0	500663.7511	295036.6337	0	429777.5251	1922252.425	345638.7415	1869375.1	934654.8556	788330.2824	801150.6149	237931.7584	221801.5093
Q8IVM8	2540377.24	2574243.516	3008641.826	1443588.27	3149755.522	1413282.909	425201.1114	1414192.296	2290789.629	2283948.43	4465845.746	2323485.213	932126.9265
Q8IVN3	0	0	1126455.229	1815694.425	0	1658196.147	0	0	0	990568.8499	1136515.655	1764871.203	0

Q8TDH9	0	193411.0459	346455.1177	726637.1246	0	644846.5965	725316.9033	246830.5684	261810.371	429858.9225	330149.8139	1272505.846	0
Q8TDJ6	0	230193.9482	0	3370.556409	9356.154268	0	23898.82924	0	0	0	188219.4957	25314.53915	0
Q8TDN6	150948.8816	175561.7143	0	46027.70392	383960.1878	318424.7532	0	0	0	247389.2712	79300.40864	344666.59	0
Q8TDQ7	199598.7254	0	0	251791.9799	0	189247.0377	58482.21129	36915.23103	69082.97621	0	87954.77288	152758.0708	538615.4451
Q8TDW0	0	0	0	36754.85526	62932.43898	0	59706.8534	0	0	16780.97432	2736903.068	0	0
Q8TDX5	6083666.238	7089887	8691787.043	6464804.877	4545232.021	2398751.349	1616891.038	1601228.109	2443288.028	9471738.38	4857449.369	10450317.89	2744487.893
Q8TDX7	147576.1219	416157.5998	0	437680.8539	1068391.542	0	0	0	0	468810.4263	657459.3159	724388.3264	69587.43439
Q8TDY2	0	92693.1779	15912.10128	103884.7888	138620.2751	181022.9309	12272.97504	99628.00058	0	0	36146.55678	33324.81255	0
Q8TDZ2	113522.0013	0	0	52831.58575	114139.2123	879482.9914	195055.6054	0	0	305122.8751	0	0	0
Q8TE77	872258.0971	294766.1527	984074.0386	531113.6197	102143.4762	362122.3606	403429.5752	320371.8242	237084.5472	552938.0836	696268.5459	504201.7793	299375.3566
Q8TEA1	951265.7199	424587.5215	363622.5706	783688.4802	424065.9579	866340.644	389820.1096	0	291960.9299	567792.659	816432.4647	1636742.772	167344.0828
Q8TEA7	61955.40906	90565.65832	0	34167.74272	73307.7857	0	0	30742.85324	21987.36348	23899.0974	32378.23465	121068.3296	0
Q8TEA8	567816.557	0	0	0	0	986100.1287	1237177.737	596620.2465	0	1437678.363	0	587861.1931	736520.0056
Q8TEB1	2130406.221	3908650.973	4690460.871	3841252.435	2243988.136	2107644.514	1123474.073	3369303.633	2545740.285	4357006.5	4891162.498	3355691.83	2028448.364
Q8TEB9	355200.9445	1217666.754	0	1126492.622	469413.691	474800.5758	737066.5679	760693.8668	700394.2826	853152.3553	1108193.647	605521.2582	838439.1813
Q8TED0	102138.6437	43373.89226	0	111132.6718	110594.2098	36923.00068	69373.44714	0	0	0	193235.0413	0	0
Q8TEH3	0	82768.94446	0	0	6860.314641	0	0	106658.4791	0	0	0	192584.5106	0
Q8TEM1	1064159.616	927992.4603	564812.3882	734836.0753	942717.6877	513428.4891	496622.8663	121930.4355	176496.1059	621379.6214	1119476.141	998672.5725	388214.965
Q8TEQ6	329370.9998	29300.35576	113787.0482	125255.8159	178506.459	151741.5072	91550.32836	78165.79091	106685.7801	141340.5575	288160.5944	423106.5359	0
Q8TEQ8	70349.71641	103104.5088	87163.50771	96831.29499	243808.4273	66178.24554	36287.58805	0	0	92518.427	88893.59962	69944.86321	6005.275308
Q8TER5	363834.3004	146624.4496	355848.1813	193721.4423	197046.5104	58532.22318	188663.4282	9244.816339	63409.85603	186240.6548	200384.9084	312105.3601	150923.5972
Q8TEW0	42715.85849	88317.98499	34404.72984	55011.91533	61541.04965	22367.77967	17395.27684	13408.71809	83343.42675	32637.92807	67947.29579	53894.86682	10811.07768
Q8TEX9	1281261.731	504144.5733	406630.3114	359550.7817	869600.7465	620120.7504	170781.7094	102159.7801	203762.0844	770260.7637	689815.0067	1099579.762	450933.4144
Q8TF05	764069.739	236535.6638	199094.6795	529929.6856	203655.2706	224040.9879	144151.995	37686.36518	60572.88163	317460.5803	324839.1425	422683.915	59931.19405
Q8TF09	0	9159117.562	3583082.726	9599457.42	12897621.09	14417949.74	22767199.75	6242075.265	9566308.805	10707137.43	10701882.32	10168293.83	3870998.403
Q8TF65	1072790.671	1428920.306	1697147.846	2934746.236	2308927.17	847635.8563	1707208.389	1017056.75	2180835.838	5012524.821	2117100.992	2916834.003	1989079.29
Q8TF72	0	320238.7373	0	0	7739.890202	53436.57432	0	517690.9632	0	12329.10914	56960.40536	46260.9967	357599.0421
Q8TF74	0	0	284069.3589	163764.7363	0	59431.63091	39876.00535	0	0	221378.1252	47803.73015	216736.5087	0
Q8WTS6	2213705.697	1275550.945	720304.176	2076009.286	1492134.37	2741817.974	1780025.695	403311.4079	1030894.517	811898.8052	2082064.742	1943599.206	208455.0147
Q8WTT2	417297.352	275789.3811	132959.945	47446.68347	397555.3778	94182.16165	75161.58774	0	0	37962.00373	187436.7466	141023.7453	99610.98369
Q8WTV0	1150072.644	3581816.085	2714726.147	2947765.185	3385202.396	1696796.215	2565789.746	1894144.49	1695251.133	2293787.401	9566308.805	3068275.63	2512544.489
Q8WTW3	307367.6918	101453.1935	84451.37273	332361.6626	119027.264	297415.2921	293344.0299	0	1413286.696	167169.8761	94547.49833	101992.8007	22721.20327
Q8WU39	9167228.192	1880026.054	1611997.874	7046008.744	9775876.598	4114243.27	18237919.74	8887266.095	1027213.92	670276.1009	1442075.955	283689.8732	3085170.889
Q8WU76	635656.5049	183612.9586	641618.5793	979105.9493	493920.7857	474282.2236	124717.6893	341061.4622	110886.1429	239290.1998	457758.1134	648162.8196	595877.4301
Q8WU79	2748653.981	1299639.029	840870.7715	3224941.43	4139793.223	1744293.761	1597459.525	773139.8906	393905.3633	1354813.193	1411917.358	2256373.103	0
Q8WU90	1374859.764	987421.8063	866225.3867	963619.8736	1809289.341	1635113.539	723702.335	807140.0085	1312826.615	1389559.814	1058908.256	1215932.272	473638.5396
Q8WUA2	345838.2872	150806.9008	130879.6345	149607.0609	119225.0633	0	139674.9694	147157.9934	13789.57738	57224.49834	266233.908	254170.934	0
[illegible]	[illegible]	[illegible]	[illegible]	[illegible]	[illegible]	[illegible]	[illegible]	[illegible]	[illegible]	[illegible]	[illegible]	[illegible]	[illegible]

Q96EL3	1261014.725	0	718601.1384	1018972.27	684163.1518	1305508.415	0	0	0	913937.5156	1128768.8	94246.46918	0	0	0	712925.7039	1035561.112	729518.3773
Q96EM0	3549656.549	753789.6661	1309336.91	2655700.91	1788123.081	587550.3687	2322618.48	335264.5221	1455436.277	1337980.985	1036365.49	1727836.866	484508.8971	1913383.58	2186905.219	869124.5514	1118777.129	2052564.833
Q96EN8	301571.0971	508302.3299	292707.2601	1074188.996	289272.5264	254426.6789	1304679.895	380512.9681	221556.2095	2160685.771	1267194.137	939112.9016	643549.4939	0	944344.8192	0	277665.4047	463894.3923
Q96EP0	70823.77298	40965.12629	0	207370.9424	96612.30143	150432.6734	0	0	0	172855.9322	38891.5684	100593.3837	12375.18065	409347.2675	289024.581	937982.9739	146387.9451	144539.7835
Q96EP5	6453488.241	9464026.521	9757578.391	12001391.43	10378624.76	9890611.344	14469009.2	6957316.748	5297030.543	9154498.381	10573048.17	12877557.23	6161166.836	11842130.54	6370337.211	19537660.58	12972620.23	4656066.047
Q96ER9	495808.7599	1760622.648	1430875.194	1276194.138	384424.3855	439790.8117	848386.79	147707.0659	404487.53	1492088.321	1222949.891	1706581.89	317491.2869	295801.6602	948064.0472	343255.9052	1687132.524	1121859.395
Q96ES7	307783.5141	193113.0903	0	143057.2608	119615.7069	100266.0848	0	57720.33923	0	130833.5675	286040.9787	98577.12171	0	209290.1021	455021.7189	543866.942	232879.4182	174111.216
Q96EV2	0	0	0	97011.74352	0	0	0	84442.54854	0	56390.17202	0	0	0	0	97825.67624	0	37245.37371	46363.94924
Q96EY1	4888867.142	2714657.91	3023871.846	4002749.102	6189690.039	2556281.439	1359336.757	1725312.283	2063817.901	4522007.913	4347764.02	4276515.23	2893077.259	4917829.191	5930392.232	2787362.803	9494969.375	5348457.694
Q96EY5	135067.383	0	0	0	0	182964.2891	0	0	0	0	0	0	0	344460.697	0	1895146.563	417582.5487	0
Q96EY7	490405.907	1327849.93	1227176.381	1122875.464	524712.4299	875707.5124	588341.2675	326549.6665	441515.2431	1180594.782	793585.328	679850.353	190803.6606	609558.0198	579240.2825	1768090.02	1560345.117	946386.1339
Q96EY8	39521660.13	13125246.7	19519326.3	17883763.79	25284973.81	20258934.08	5406931.463	10152499.46	9687837.07	25382804.05	29654690.15	13586973.34	7142716.639	8677074.724	16326894.69	1875011.407	16866012.8	29428461.12
Q96F07	796296.0101	368934.0944	208363.2608	376067.0998	535804.7761	251805.4816	393025.4403	177530.531	213719.3371	201057.7013	528025.1781	195701.2517	86988.55182	354153.2392	439388.4824	2602563.78	488740.6599	383082.8819
Q96F10	10281325.27	21523798.83	11944973.03	13617710.54	11658125.16	11860531.38	4719650.119	13737185.42	10713558.23	19705206.27	8895989.263	14898285.52	9217941.606	8220802.494	11257075.64	837034.4895	14240582.6	15632241.67
Q96F15	944383.227	291826.6408	151694.6858	921215.8644	1122921.655	295090.8537	915028.9237	430586.6479	0	305080.9444	878879.438	732930.5042	0	684183.5543	985458.0214	103567.8559	2145221.725	528376.4924
Q96F24	140397.4637	0	668880.0793	340368.6459	214706.7365	391634.9359	0	0	0	389543.8578	0	226261.663	0	512279.7935	241431.1355	1224342.058	746000.0864	0
Q96F25	1133198.831	509479.9131	764908.9781	760548.8328	584085.4302	774581.0164	785983.6971	171167.9885	0	580473.7317	398637.5044	295885.9171	0	424867.5734	309403.4559	264439.7959	1112363.412	422349.0875
Q96F85	2043555.486	971178.1591	1168048.778	2031933.151	1697005.133	409873.9209	1330314.531	975754.0345	0	991621.7196	2055620.147	2231692.74	0	3726144.417	1459997.975	732756.6787	2069196.301	1483110.037
Q96F86	234588.3564	58976.32737	0	0	101597.396	0	58967.3527	0	0	181559.8476	180764.1737	138965.7861	0	122292.5522	185858.0348	158913.0165	86271.44957	63855.7481
Q96FF7	501486.358	370660.8037	649494.7641	425508.3498	982375.5113	298167.6681	446610.9668	155382.9225	599797.8908	1191876.229	547085.868	700104.6099	957634.8464	340458.0883	1814801.894	284477.8309	437996.9623	1315651.413
Q96FJ2	12372421.01	0	455173.8801	11133042.81	0	508260.8482	949173.5321	0	2061339.291	8127406.026	455250.4686	2089641.078	0	4074959.286	1305821.998	4386773.711	4709661.703	3688637.66
Q96FN4	0	242087.1224	48319.62738	306815.4462	567149.5211	0	130426.5412	112265.3297	217124.7143	20105.09504	344700.9341	77963.01092	0	688708.685	351378.0377	4093529.985	859188.9609	82962.34479
Q96FQ6	19744446.75	6532588.931	2182933.074	5287192.924	6052704.506	12374437.48	8918061.857	10170213.09	3908175.895	4213114.548	17214400.77	8761199.521	4649506.108	17119916.85	4179521.858	17986356.29	1873698.609	1079129.01
Q96FS4	25699.06931	0	0	95001.68096	270492.4723	164606.5923	82286.94131	0	132356.8279	96966.89998	83297.02722	0	0	135893.0272	0	636315.831	0	77401.83341
Q96FV2	25768686.91	11524314.57	18222097.56	19836675.02	12751702.39	6796839.921	10203055	11125624.84	12215929.37	9458971.444	12122955.09	19846584.73	14070600.05	8409261.244	15586063.39	6774650.593	11311126.38	17300413.18
Q96FV9	186568.6127	0	0	115065.7591	195976.823	32049.62782	24284.02616	130885.4815	52518.98328	19614.20011	33237.68358	99078.82515	0	583507.4925	50512.95951	2044501.331	159837.994	70535.8621
Q96FW1	16829014.85	15513057.91	16562071.48	20116581.79	18938083.14	16700625	18478102.82	12412247.81	13012459.6	12763536.89	17480594.03	16435719.97	12845803.4	25150416.36	17198380.24	54264063.85	21633569.9	26750347.87
Q96FX7	0	0	0	62854.50161	196782.6246	166620.9577	181774.6186	125685.2851	47136.60345	0	0	274055.9326	60124.73875	357147.9449	245700.4496	696101.1959	510127.3077	157820.8379
Q96FX8	2389230.4	1140362.842	815805.1811	716788.2488	2600446.542	1755565.215	0	0	0	602463.8568	0	709594.9293	0	655727.9548	900651.2102	5239670.694	797255.5984	515906.5139
Q96FZ2	129670.7278	0	102930.0061	105191.7741	0	0	0	85001.51191	0	0	155210.08	65957.97504	0	126887.7573	0	257712.2274	78079.30706	0
Q96FZ7	3811527.911	3898844.517	2433757.517	3310116.459	3242092.307	1738673.426	5102182.852	6772580.341	7759693.745	4847443.269	5892956	5868283.864	1945371.851	4592823.707	5027425.122	4660169.717	5372754.982	5185671.641
Q96G03	16016630.6	8650083.797	15202806.23	17343187.68	12225520.05	19137983.74	29399082.16	20073093.63	16555208.92	19021543.98	14742228.29	15860900.16	19703306.53	19728132.17	13709406.95	19675728.44	18233928.06	18405904.11
Q96G23	11656717.48	17560994.44	9271682.123	15184573.26	19679894.76	8897866.5	8675738.659	6622557.988	5684799.839	13201513.45	11111610.18	15762493.49	5427260.813	5764608.303	24455304.67	7009837.819	17990436.65	15834125.6
Q96GA7	24563165.47	22565504.97	27142624.2	24179922.07	13519405.83	8389512.869	24564229.77	14890116.83	12989376.81	27866287.57	35654773.94	28494799.37	17498886.43	4647537.192	15087804.57	1658352.31	11385980.62	23989541.59
Q96GC5	0	297146.2035	882734.4974	470243.7495	492256.087	0	0	342016.1865	286788.4685	918761.0931	819067.2884	681699.972	0	0	584723.8963	195909.0697	1360062.845	1126543.609
Q96GC9	769616.9636	0	1008161.593	1892035.031	480492.8442	682188.6489	2338358.094	0	431495.9186	644962.6213	701527.6055	1040061.507	983949.809	398649.1422	1220175.66	1594952.468	1109095.345	466492.7346
Q96GD0	6314540.699	4797292.847	3472505.188	5942714.083	2083855.264	1924116.987	1681021.975	2271130.096	5876811.617	7644014.699	4047730.854	3941571.558	1181306.364	3942695.42	6985254.194	447690.4911	3774880.26	6236594.281

Q9BRX2	696804.5233	918886.6553	0	136108.9935	437197.8319	564969.408	217279.1896	268648.4258	448027.4361	427835.1345	245026.4799	478725.2125	11002.57457
Q9BRX8	32847211.95	37036670.9	26905625.1	16590089.26	23550573.97	32524260.95	21820126.41	20469453.17	22271005.05	38376094.25	42460096.81	37792902.62	16563213.21
Q9BRZ2	858233.8061	334685.7895	172129.7947	194000.0386	315494.8091	139286.2014	16816.22295	32599.35469	0	346167.1847	332763.2811	285385.8389	89190.19963
Q9BS26	31615193.82	29620581.1	23884703.1	44712688.95	32249600.66	28671547.94	32147676.37	19738692.8	26872769.68	30554411.09	35573835.4	35348832.21	24068748.69
Q9BS40	7598922.175	1821370.319	4635312.526	12488602.4	10829342.32	8105495.338	27780271.83	15731538.16	2471663.267	1974578.639	6297899.581	2835295.116	3162469.946
Q9BSA9	920529.1551	395069.9563	967869.2704	1847441.056	860412.5755	248584.2379	284094.4781	157851.9162	2208671.473	350376.6757	1000079.151	635546.0657	445865.8954
Q9BSD7	8188408.578	4887841.729	1535238.638	5000338.259	4668237.883	3450579.659	1172707.133	644743.8209	481098.7927	2475003.533	5680632.788	3551561.567	2737194.558
Q9BSE5	41232980.82	47887588.5	36085559.23	44929511.33	26308314.19	52558570.1	15193565.48	3824722.401	56414689.75	70361645.61	43902998.98	88999034.37	32529927.35
Q9BSF4	0	878427.4479	275907.5496	793650.3569	762226.9375	0	84592.41467	493335.6993	473084.2622	1044760.019	1616094.257	1268776.484	0
Q9BSH4	1885674.885	10481519.69	8885933.208	10031112.99	4477906.283	7733871.342	4492294.133	2426768.118	6615684.113	8598866.56	8665788.797	6517752.527	3039691.549
Q9BSH5	22179553.4	44873063.29	27608743.97	36124222.33	32395852.92	33227100.74	10824637.01	12226658.25	22652044.97	43240184.53	29467911.47	33777934.15	15382740.61
Q9BSJ2	67982.05127	0	27303.07992	0	67748.62401	0	102902.2836	0	0	0	107247.5129	63339.45657	0
Q9BSJ8	12691822.77	13896960.12	10027780.05	12541084.36	12076357.34	8323551.004	12382496.35	6458553.158	4869871.591	10061668.47	16768572.63	11489121.58	6290088.19
Q9BSL1	898621.6517	1169256.219	688658.6263	961519.681	1840854.592	535893.5694	271361.3064	391694.9645	349840.6397	620708.4226	267939.2401	802751.3077	0
Q9BSQ5	0	224844.1661	0	27045.62227	698066.9013	54719.0944	0	0	0	501164.3838	61539.07499	36219.09372	0
Q9BST9	716147.7494	327313.0703	77291.65245	483867.5314	374842.1632	486201.5151	253722.3893	70839.59558	0	470346.9342	1002906.944	997594.0393	154726.8844
Q9BSY4	592829.7545	2345214.773	3289865.24	3967051.758	3511468.948	4214284.348	2148618.716	659995.7134	433308.9674	1780871.71	2705231.111	6914705.133	933426.0121
Q9BT09	2356016.82	1127929.904	3516296.484	4855343.686	4630974.007	4084242.969	2129297.983	5209190.111	3698564.265	2524174.292	2730769.333	3923201.178	3315890.181
Q9BT22	1560454.961	1938589.084	0	222816.8469	1038623.912	1369761.466	983879.1638	219800.8723	569040.6285	372761.7926	1206967.339	1227139.598	205394.5359
Q9BT30	472159.2772	936270.3336	0	854532.569	1131374.397	1075099.342	213615.8841	111366.5679	670581.5361	1620013.815	744689.0563	155370.7262	0
Q9BT40	109586.1072	152648.406	0	317206.7232	307810.9315	0	307473.1945	170904.8018	0	268630.3076	361593.255	243921.422	158403.3916
Q9BT67	163776.4257	0	120642.1171	0	0	0	0	220141.9507	0	162602.51	220508.1101	0	0
Q9BT73	1527432.391	1040124.48	1661084.081	1260410.777	1052302.537	0	705882.5054	1490046.044	918589.2249	1308216.438	1027104.568	2599110.108	1633662.708
Q9BT78	5037180.423	5416347.186	6676178.814	5729577.533	4796746.041	4672443.078	5498394.603	3110685.139	3267337.254	6822387.956	7640137.877	6889785.759	2711415.513
Q9BTA9	0	69750.47217	131585.6085	265885.1206	107053.4567	166875.1635	119702.5828	0	47292.21021	171065.1439	311711.1883	302185.295	0
Q9BTC0	62807.88657	25188.54294	0	13883.33675	77635.93507	19128.02949	33113.47527	11015.95088	0	0	26616.49877	100269.4451	0
Q9BTD8	0	0	27040.82207	107603.9565	0	288029.3992	122239.8184	110897.9844	144199.3027	227263.102	153678.7921	248704.2819	80380.68513
Q9BTE1	0	0	0	403680.8073	621592.1756	0	0	0	0	103744.8219	0	0	0
Q9BTE3	214905.1932	60192.56848	45815.70316	52141.92542	186969.7467	187387.8168	78490.31102	0	0	137022.9231	260782.1506	176277.979	0
Q9BTE6	1032853.368	1747224.347	628484.4307	879956.4306	1550351.182	579101.2484	962009.8565	0	557486.0453	1368273.697	1588916.109	1592120.281	208100.2108
Q9BTM1	550865923.9	1319398659	359118394.2	0	0	191952363.1	558720272.8	639297967	8663586.32	0	0	0	308610810.7
Q9BTM9	0	0	812857.5529	6025759.006	3804762.226	4839657.907	0	3505594.992	0	6593942.243	4402658.595	1023630.051	0
Q9BTT0	9638526.498	11373462.17	8642064.415	11367294.14	11182993.27	20482443.92	7196202.498	8858748.849	11946631.04	5252804.687	10155641.79	9445658.981	9864031.575
Q9BTT6	24972.26435	0	0	0	0	0	0	0	0	0	0	0	0
Q9BTU6	713171.1091	1026614.448	612531.3761	334687.4813	779847.0295	468274.873	679377.9581	44308.02422	224316.0233	771040.5072	1043911.636	522022.3983	109822.0949
Q9BTV4	6283287.293	4284206.436	1076469.957	3724998.617	4144302.331	5107614.852	3560604.151	2026242.667	1437569.195	3537856.083	5103264.786	2856928.032	4464253.823
Q9BTW9	185562.1701	715013.5485	258515.4696	175290.5679	172402.4399	361184.2962	473533.1741	177819.3706	99724.41222	635391.7801	791206.0529	293803.894	25972.09534

Q9H4A4	18956966.84	14582975.45	16871554.84	11664610.93	19197851.9	17797586.42	22252869.96	12069145.74	8864140.12	15544574.75	19668881.16	26702870.91	10743168.78	[illegible]
Q9H4A5	0	160691.7386	0	314973.429	176770.7155	0	0	0	351230.1149	0	134200.7933	413176.4859	280488.4372	[illegible]
Q9H4A6	5880853.175	3564543.94	2260327.784	3541708.345	4202186.678	1846991.474	3507625.812	187570.9285	860353.0148	2943580.013	3293642.851	2516126.678	1344624.184	[illegible]
Q9H4B0	0	0	559359.3574	0	167520.9824	169853.3171	0	0	0	658472.9491	445498.0042	185599.5841	0	[illegible]
Q9H4F8	641811.1164	406482.9406	837986.3873	328521.0814	284219.322	666204.2828	476957.5204	1101510.545	906306.8012	1014116.91	697692.1259	911002.2955	149042.3425	[illegible]
Q9H4G0	2218684.911	0	77623.20332	0	0	327696.5963	840891.4708	635816.2169	1151040.065	230964.5135	410209.9066	211694.6466	332744.7762	[illegible]
Q9H4G4	5080477.176	2624268.138	2636959.158	4486126.562	4135212.035	7076235.123	7615631.665	9553808.641	3258712.896	3922280.822	2708074.434	4295510.998	4743067.42	[illegible]
Q9H4I3	215208.4666	88656.95536	200937.261	184185.3689	56248.98442	0	0	0	0	279495.8468	297298.7353	132196.6159	71641.94948	[illegible]
Q9H4M3	907059.7548	0	0	451265.7323	73889.47	109111.1088	277549.1757	110673.8005	232407.4442	182400.462	152391.272	220334.5419	0	[illegible]
Q9H4M9	10758638.23	6414252.299	2282542.229	13976212.24	12296565.89	9500227.784	1763667.019	2652613.727	5208119.906	3624440.857	3182641.969	4693794.904	8419099.296	[illegible]
Q9H4Z3	243738.079	36200.79036	67701.19076	88322.69291	16569.988	19636.88794	0	33522.05095	0	126014.9111	0	28107.97946	0	[illegible]
Q9H553	4478802.481	4216272.255	2752159.545	4548433.888	5776228.26	2593944.729	1869478.982	0	1430216.932	3665890.47	4802488.411	3366275.709	861242.7186	[illegible]
Q9H583	77847.07172	45544.04576	0	186423.3823	65931.67007	477064.067	92596.93893	0	29204.20193	82995.25143	138793.9649	136116.724	31197.77461	[illegible]
Q9H5N1	393750.3408	361057.2616	108998.8751	143915.3932	420721.0084	149508.2513	394403.4024	160419.1883	65726.1049	154608.7238	187190.6708	484804.3911	74406.32331	[illegible]
Q9H5Q4	241468.0396	706269.8162	408111.3285	309511.0965	153889.7863	88509.35011	535120.3283	564285.3206	143958.775	404825.7591	321402.299	157813.2292	479427.4821	[illegible]
Q9H5X1	3521999.959	3042248.358	7536526.695	7063965.218	4519306.859	6221477.332	6357325.75	5733265.428	7249400.251	3694636.612	6247313.367	7329532.862	4137246.987	[illegible]
Q9H6A0	0	0	240284.0837	0	221355.7499	95615.14291	89750.07421	0	0	0	0	0	0	[illegible]
Q9H6E4	303525.5625	315446.808	239953.1402	241792.1854	883403.1455	0	0	0	365869.6626	0	136144.3094	790337.2433	110944.4273	[illegible]
Q9H6K4	2379123.1	3407269.488	1583568.058	1503646.055	2612026.872	0	532651.884	234265.7838	4076726.276	2432309.569	1129951.71	1654934.814	2013086.93	[illegible]
Q9H6Q4	147339.3926	102027.3403	376336.3003	949473.5915	346613.511	44370.45414	464046.6214	0	0	756687.6826	904105.357	896657.6566	119354.1461	[illegible]
Q9H6R3	26556548.24	18180961.8	17792940.62	15026184.14	17497154.71	8304084.674	16133637.61	7030796.841	11829627.14	22287397.14	16974327.98	16878086.01	7517201.432	[illegible]
Q9H6R4	144035.3192	224216.9219	0	48249.44634	62210.89572	41852.05018	0	0	48872.3329	142200.5767	152815.7959	219779.2718	0	[illegible]
Q9H6S0	70670.37279	82283.30784	52574.8897	210513.3083	61086.85186	19391.49413	0	0	0	160010.6464	118149.8992	64184.13318	0	[illegible]
Q9H6S3	6023929.084	6071247.945	8937129.848	8219489.459	9446503.304	5163325.474	3410631.285	1952817.106	3280634.466	7653091.791	5906552.255	7949209.635	4778301.262	[illegible]
Q9H6T0	569654.6464	476984.7477	364577.6222	469218.9206	0	263947.9528	0	761730.1326	308063.7271	565939.9899	500755.2885	787941.043	324146.3459	[illegible]
Q9H6T3	156416.7198	139838.525	117654.9765	343028.7068	305471.1634	27866.76664	159631.4918	0	0	81874.16584	226157.9975	237326.3519	0	[illegible]
Q9H6U6	285715.6656	249613.4092	113386.376	136502.8629	189308.7912	63851.50718	0	0	0	119203.779	250724.0548	111742.3149	0	[illegible]
Q9H6U8	420716.8619	475777.1442	0	829014.6937	340296.2828	0	71128.07942	0	0	369753.8207	490706.6223	176767.1397	0	[illegible]
Q9H6V9	105414.3123	1276925.91	761229.3386	1086758.581	1175344.62	903640.1219	726803.1548	842813.0955	0	1110945.341	1466868.589	886532.7895	1652662.064	[illegible]
Q9H6Y2	0	0	0	109297.5749	0	114481.7685	0	0	35748.54341	0	0	0	0	[illegible]
Q9H6Z4	1294903.414	2178767.249	1018842.058	943660.997	498843.1556	1294532.824	1325886.028	1100133.439	1441224.537	1454602.694	1849680.163	1747371.442	1107389.065	[illegible]
Q9H773	0	0	244591.8426	276656.9451	531738.7521	91059.81021	1859053.585	532901.6031	276926.7231	493425.8477	409797.473	208675.7988	909893.0204	[illegible]
Q9H788	0	1167985.604	1773655.963	363969.5819	1181481.248	454444.3232	1252548.875	3721500.071	406198.9326	761479.1492	1543898.382	2277955.895	0	[illegible]
Q9H7B2	0	367560.1224	109209.9368	185435.7535	0	0	471827.287	0	0	391209.5293	414200.2141	0	0	[illegible]
Q9H7C9	7054594.054	6455856.981	9742097.856	9284683.039	0	2573683.452	3419014.149	3439553.137	1643096.019	5146527.088	5429037.314	7871605.428	8624188.021	[illegible]
Q9H7D0	51926.1164	27982.16319	3898.437466	69806.20918	0	0	0	0	43179.17756	45394.98689	87644.77897	16251.47079	0	[illegible]
Q9H7D7	582334.7561	550714.8894	202812.0394	487633.4158	835477.0479	436273.9891	453167.2584	118129.3418	78656.03405	400111.4567	763237.3272	424015.9221	47384.56841	[illegible]
[illegible]	[illegible]	[illegible]	[illegible]	[illegible]	[illegible]	[illegible]	[illegible]	[illegible]	[illegible]	[illegible]	[illegible]	[illegible]	[illegible]	[illegible]

Q9NWW6	2061219.889	2791308.208	3503801.433	4325546.936	5798594.913	4850989.037	2166092.885	481832.7181	0	5098421.574	3011359.65	3766966.887	1731984.8	0	4868583.878	2290629.289	2761790.817	8902869.979	3081982.994	3079670.209	1743829.151	3482207.612
Q9NWX6	0	674852.8378	0	117121.7351	361792.2299	0	105614.6654	0	0	141781.9085	811346.7698	135332.0418	121087.4379	123754.3137	150984.0503	0	0	0	412771.0656	405875.5398	312313.0534	279350.545
Q9NWY4	120670.9152	0	0	0	224275.6667	108721.5236	152793.7473	0	0	102334.1561	69893.52753	336935.413	0	388919.8649	486908.9812	603081.561	377005.5546	146718.815	57952.35418	315171.1267	731639.5466	0
Q9NWZ3	0	88147.36116	338359.3044	124435.5466	252872.9417	0	80373.8046	0	0	0	95572.91734	117352.3014	0	511726.6047	188412.192	403978.7588	123095.5918	0	0	53157.15126	0	0
Q9NX00	238371.1621	0	585918.4148	594594.0325	805210.6352	4804184.773	0	0	0	639523.8949	1300686.583	688420.4393	0	174947.0549	105066.7146	388357.6681	647227.7745	457264.0257	0	951608.0482	0	0
Q9NX08	2473984.152	3833566.35	2195933.255	2944032.741	4882852.024	2037967.658	3172085.882	1314282.578	1649125.058	2461326.695	2397272.683	4889444.327	1629184.453	6235467.288	2428592.983	9530284.493	7197883.223	6561082.994	2571370.574	2271530.325	4585988.733	1915134.913
Q9NX14	4170156.124	3586544.452	8753247.289	8380351.49	7559350.548	1704247.286	2760804.189	6069803.401	3032065.876	4490042.869	3767116.079	3145022.931	3257059.053	1953719.207	3856422.582	1469507.513	3854111.89	5354318.646	3296172.954	5177642.15	1687507.529	2120930.417
Q9NX18	0	425567.6706	0	0	0	0	0	490101.6269	624100.3524	800371.0427	1006823.037	1118877.884	466545.5688	469171.1047	1759379.42	0	0	824965.6864	297823.9569	376953.2704	279266.8943	0
Q9NX20	0	516491.3649	682337.6594	307680.5381	611654.4097	0	0	332507.8304	292403.294	795212.0261	988190.36	880397.6835	584118.4309	0	243603.2961	419807.4427	525151.6056	0	900913.6977	93997.77101	365935.155	763856.0927
Q9NX24	4683280.454	4421353.463	2392856.934	1938731.707	7472046.053	2195723.14	5476676.619	0	2627991.976	473603.2996	3044818.818	3720708.67	1196636.071	3551962.753	3408086.142	12948895.57	5572431.287	5689757.05	1028802.909	5836092.538	2152437.087	2174698.061
Q9NX40	20617535.93	14537856.21	17151852.56	27254191.96	16530176.53	27729000.75	15071770.24	25106078.64	12793096.24	26569477.84	26040445.09	26194316.68	12121719.36	7939129.619	17752137.74	13687224.47	23658398.05	20353460.62	24610360.94	23581349.09	12232299.35	14766980.14
Q9NX46	3503078.514	3419690.45	3970368.125	5836053.105	5212504.864	4250357.958	4514623.843	2309053.199	2133646.958	3885648.166	6515138.946	5290760.581	2748064.145	5202324.226	5091833.092	11875953.33	7157279.501	5844691.405	4065813.932	1482347.656	8322770.762	2291028.207
Q9NX47	1636482.097	2269473.812	994362.9478	3113159.308	3160841.064	1312549.725	186946.9013	0	781868.3648	1634574.573	4617666.733	2352482.125	0	1971445.443	4218346.28	1017285.901	4392460.438	2338825.221	1463489.463	2285957.59	485778.4413	640740.741
Q9NX55	1980347.783	1257604.263	0	1700078.697	4238442.571	2648987.213	2586489.932	546132.0598	261435.2609	803892.2605	855048.6678	4107004.106	356044.4505	2000958.753	1244534.841	8368335.512	1920692.838	3179367.547	2350323.199	2096373.864	2481071.317	544537.2982
Q9NX57	487143.9064	167162.0379	266410.2911	599797.5273	368836.589	139719.607	0	208828.9769	0	13559703.83	511511.4568	151067.4982	0	564489.8674	71121.72917	618868.9294	807047.4449	121033.2827	150601.4606	379976.7168	537560.1867	0
Q9NX58	265644.0611	381832.551	440328.0026	433182.8664	81990.61101	723217.212	448206.3372	0	0	802101.364	160858.1565	1137015.247	242876.638	453413.1466	135999.8621	611404.3811	467970.4892	148919.4645	484146.4752	644125.8498	976930.2987	274407.2453
Q9NX61	224892.5412	0	0	0	169933.2013	76801.91368	31539.26701	0	0	0	0	468023.8551	0	70985.65547	232247.0988	85636.23334	265531.6995	314679.7974	0	0	0	0
Q9NX62	122255.2042	0	435359.2101	779036.6706	1548787.542	573990.9211	582886.9943	155108.4373	496892.7921	120011.9083	994061.1282	194171.6882	190486.18	615577.2128	560085.2471	3302946.184	1618824.852	888269.1976	0	338814.4462	194212.2498	299925.7431
Q9NX63	11576130.65	15588911.34	18475349.58	17702675.18	12953798.15	10181966.24	16191038.89	21284905.04	17459733.04	14455066.53	18109057.85	17011982.62	19233089.89	12857863.7	17250858.94	19829121.91	21095221.56	15934179.81	13422539.37	18096143.9	19666010.99	13243233.78
Q9NX95	353019.3573	78020.772	0	101614.125	140982.6912	119088.4939	0	54835.91809	111782.8756	116384.479	78850.60234	179708.5687	57138.13888	190087.1257	200345.1227	0	204146.2281	182436.4122	0	79936.81404	60115.62201	107931.6402
Q9NXA8	9871091.926	7417056.657	5225212.498	9946198.939	13225503.06	3342428.397	3591220.348	5253436.014	2361829.067	8675969.991	7142976.319	7301658.199	4750115.423	9331986.268	10648004.45	3195685.475	12026075.41	7556543.783	5603585.597	6257410.099	6904609.226	4639317.909
Q9NXC5	115672.5036	372012.2581	361572.8257	479747.841	295828.2289	108922.2338	84085.97929	212570.1378	304446.0694	393450.7774	462245.4448	838674.145	83870.18482	320289.3771	393983.671	160992.1736	834682.5217	440577.378	1339702.267	193238.5446	188420.2365	265142.4706
Q9NXD2	0	322690.3442	0	414746.1756	142848.2573	148397.8702	37619.33416	0	0	737505.4748	405333.8433	644700.7949	0	345552.9801	267743.0674	0	243361.7008	444638.3263	280292.4608	82976.1302	103636.3188	200234.8367
Q9NXE4	61234.64731	0	73124.89331	59722.58139	11688.62786	15408.21065	346908.986	17002.95177	13477.63247	86000.60891	47178.96553	89400.3391	17622.23216	128803.0815	119902.9288	159959.7583	84230.02693	48708.16982	61479.55106	83731.45194	61634.58407	27549.36776
Q9NXF1	102990.6995	46238.88844	28141.41086	168984.3127	322785.9566	106960.8422	105429.077	0	0	84094.75354	55903.96892	121984.2226	63226.76328	363539.5876	188152.185	798804.7507	303700.6854	97189.2104	23021.59071	48317.97721	226768.0316	601800.9628
Q9NXG2	2436413.175	3117802.943	2738469.612	5592727.099	2729674.415	2729272.961	1527197.813	2067326.396	1834800.07	2086670.195	4555073.816	3097638.196	2990572.285	3929971.641	2648929.542	6116459.712	3810467.51	2299393.514	2334627.837	5159980.936	4591202.008	2901034.063
Q9NXG6	35194.56055	0	0	112071.0178	73018.79298	0	0	0	0	0	0	60076.03785	65043.40047	231153.1131	426556.9292	334201.9068	554321.9871	81065.09298	64012.96306	0	0	0
Q9NXH8	0	0	0	0	0	32174.36114	344379.5268	0	0	0	0	39956.62983	0	473595.4931	118936.522	1390469.735	54938.01941	0	0	0	137845.904	0
Q9NXH9	0	114877.5423	0	0	0	0	182064.5814	0	0	0	91645.78192	11158.41698	0	0	0	0	78829.95255	0	71946.60714	0	0	0
Q9NXJ5	3525532.575	3629953.46	0	2486620.925	2351766.345	2007189.965	1995316.91	1935122.335	4883957.726	4223292.973	2215902.584	2157906.753	3477076.558	1408899.275	2191492.064	301349.6005	1662599.023	2088214.413	2279400.742	1705128.634	3769795.663	3717765.811
Q9NXN4	117176.2909	292614.5306	0	0	0	123788.8168	58010.49434	0	0	68416.88325	229380.7539	106674.0681	35591.49902	0	721003.7287	105829.6632	0	83878.8714	130240.7871	88219.73158	155678.8461	0
Q9NXR1	110506.0123	0	0	0	160423.0465	278279.0784	1133590.132	197676.1451	0	0	0	58958.74691	0	212697.0805	137805.923	237889.339	304727.4063	0	0	70856.8067	100083.7084	70352.81685
Q9NXR7	4600831.915	2055673.684	1416064.205	1736952.948	2036830.71	1784547.642	1353651.786	1195584.466	678035.6592	2504852.69	2667579.223	1939722.515	1427968.123	2508573.461	2774089.16	3676978.493	3123307.623	1350285.587	1899300.753	1725156.488	2419578.03	2755451.693
Q9NXS2	410364.3367	545815.0955	0	681197.4722	139904.3385	109307.0113	0	191255.6927	189128.3591	181300.8495	717598.9379	569099.5961	0	885192.1566	755215.8912	540346.8635	579307.2435	534697.4362	598005.5804	644808.8197	723849.0321	363128.4272
Q9NXU5	168147.5383	495887.3365	0	146744.2457	402175.8283	0	157635.73	0	0	271263.3506	0	366697.1964	0	0	322420.4719	448627.6051	483439.3289	727295.4173	250140.0189	394103.0406	0	0
Q9NXV6	408983.5086	513004.7195	885721.59	857177.3424	527131.7218	851368.6039	1010781.111	1067432.481	961518.053	747083.2537	825262.4635	988798.8003	653770.0689	278338.4797	880666.0855	957042.1342	513163.4988	203469.5731	811056.5139	1220199.869	1328602.186	1216754.538
Q9NXW2	759141.712	1422789.35	261898.1173	1523056.487	352909.6255	315692.1187	0	0	857159.517	503969.7936	645963.5985	2040062.874	0	1926210.374	2393638.963	939700.0596	2267500.48	1110106.942	1137884.78	447723.5432	0	262600.5999
Q9NY12	1111678.046	682027.2313	371821.0402	1291245.961	454402.0176	415550.222	399558.1369	237210.9075	1718915.434	1208495.497	1458120.701	780896.1464	459585.5918	1045348.328	2189288.233	5207031.322	1582090.626	228894.8828	858942.286	0	1441027.018	764731.9907
Q9NY15	1806658.226	1889193.808	489374.7266	1374957.926	1316365.577	1318199.564	443485.3332	572357.1519	948474.1676	1934042.056	1449292.088	2359792.044	739250.3828	2082188.485	1807139.628	276003.8738	3734916.634	1009981.29	1547565.912	2320489.836	1349387.726	1161273.263
Q9NY27	613523.3656	178148.721	163177.3927	374719.7187	154121.6041	1092898.421	1067082.529	1212042.274	1145301.985	584268.0613	659668.4056	485347.3567	886931.245	554252.0229	448101.6845	1485864.367	539032.9857	143043.6139	572460.3379	567690.3417	1092439.007	1167466.862
Q9NY33	7108310.519	10172367.35	8379948.995	13046940.38	10142744.01	8789851.154	12041562.23	9194090.224	9523358.625	10902198.69	11277128.94	9493590.13	13146087.01	12285877.86	13594607.36	20105292.81	15244614.51	11225199.78	6498186.701	9029537.092	15093111.08	14666157.81
Q9NY61	0	0	22945.4693	352492.2101	207529.0912	0	0	0	0	0	0	0	139307.6138	0	0	240007.9627	108124.2659	0	0	0	0	0
Q9NY93	0	166126.2613	177849.6584	54323.52505	86421.85407	369744.699	197355.7144	0	0	159514.9915	71968.99804	67273.63649	93315.09402	0	70377.35717	0	0	0	378040.0143	177879.127	188893.9237	224313.9996
Q9NYB0	86407.19303	111332.0385	0	511213.7427	906907.442	118883.4958	246738.0647	0	0	267602.17	498053.334	0	0	904429.8634	978743.6619	1242821.215	428473.0418	548010.6048	68631.73223	0	702598.2131	278619.2645
Q9NYF8	1300327.098	1017687.296	1286649.59	565064.7377	911578.9765	940305.3229	1897990.648	923239.7736	489886.0433	1070113.618	2635633.784	1298415.083	737410.6869	1455922.723	1986692.334	2365353.58	1947171.116	1525861.628	1771431.836	1978830.85	573765.475	596138.345
Q9NYH9	112432.1404	80214.12582	0	178242.4877	0	0	0	0	0	0	0	0	0	0	111908.3895	151675.8525	88680.02277	121222.7984	52782.43988	0	151157.7611	39619.54699
Q9NYJ1	0	2242569.757	3093307.031	458889.3568	1786239.085	7414666.184	0	0	0	1982823.77	2467953.413	6665837.368	0	3374181.258	6228936.125	5441787.567	4273799.438	3755438.715	0	1640114.372	0	0
Q9NYK5	165444.4444	928967.5618	181855.3605	486042.1793	828466.6984	360708.9106	239145.5417	288295.4097	175910.7041	663745.4699	810485.5152	255125.8613	0		281081.1736	847700.6127	1162800.089	532372.2243	879177.5082	571402.031	540962.0897	318424.7532
Q9NYL2	96059.4156	56153.44087	0	154910.5224	0	129464.8028	0	0	113109.1603	23176.01039	285019.339	249552.4256	0	138988.1831	421607.9297	290837.7028	290225.271	517984.8923	260423.4024	187531.8918	48717.42486	257054.4867
Q9NYL4	9805950.096	5035755.051	4586356.308	8488789.122	7433259.257	7444177.828	8782255.872	6535252.257	8807517.275	5548444.217	11080243.44	12787734.77	6445662.844	11666533.63	9289287.712	3465868.727	14199165.02	5990265.648	4766085.319	21911214.43	18954618.1	6270813.039
Q9NYL5	1192974.764	0	351122.2063	0	0	245361.5499	91723.12547	322670.1716	1114847.669	395326.818	107794.4481	213941.7132	234887.3095	197254.3632	1123516.72	0	980431.9098	576202.6926	1201925.405	1042216.244	1432143.228	562857.7172
Q9NYL9	10124872.91	12489730.57	10154625.96	8556281.462	7021321.473	12535030.44	11783875.02	7840508.737	9058241.418	8364000.648	10548000.02	9724826.286	7779214.391	19634458.13	10621004.63	28190740.27	13131681.84	14426767.57	5058733.457	8519012.583	15336898.08	12741963.41
Q9NYM9	700008.4294	0	0	0	0	504195.561	1767497.061	0	0	0	369613.4197	359853.543	0	3782704.72	0	6434070.424	2632597.757	608309.0908	869472.035	1044487.257	505210.0639	0
Q9NYP7	3241818.4	2498538.091	1689145.231	228467.9698	64840.65008	1166118.845	0	0	946213.9617	0	3669653.348	2027533.157	1077699.447	808036.1246	1195742.499	3362116.204	2448488.214	1999262.02	726754.4211	0	2556814.84	0
Q9NYQ3	3719294.098	9225116.389	5500684.062	5995919.137	5064765.44	4701014.385	659593.5234	5699146.242	4448938.871	8789028.186	10640009.61	8839992.829	5142855.148	796614.1333	2675154.323	0	3911562.961	8477881.676	2036670.01	6340794.358	234600.0079	1625053.182
Q9NYU2	12095595.94	16914849.41	13687196.68	16729291.45	20979271.53	15883331.57	11958068.37	7686980.176	12859475.38	14068418.63	17964618.42	16803663.11	9997875.709	16558025.33	13343455.54	13791562.81	14101425.35	12711257.09	10715528.87	12172482.98	17112378.14	11751951.72
Q9NYY8	145373.9733	64877.66626	0	165886.6574	85600.53706	81093.39472	0	39457.58503	0	346931.1359	208554.2782	180419.5414	23325.80996	0	158450.4894	160790.79	112570.4969	127749.0989	81534.2876	190585.8554	21031.27557	54272.18759
Q9NZ01	36340202.17	33250962.09	22652478.48	23159162.54	21162026.03	22004007	15808823.13	22348626.2	25879924.17	26682323.8	31440302.76	33911671.53	16899025.09	7537014.542	18815350.37	9093229.998	19319404.27	19102212.92	22646029.91	28879416.59	16125393.63	21269056.58
Q9NZ08	18217746.14	11836897.86	8765738.062	16037655.65	25233838.27	8095861.803	9566308.805	12087919.38	10144514.69	13902708.06	5190371.71	3301229.246	11823775.49	8049301.063	17491155.03	23967230.51	22062645.78	23902581.53	16681794.15	3053279.718	29903161.51	11495037.23
Q9NZ09	365597.3203	0	143569.0696	351608.826	137553.8275	172137.5923	0	0	0	0	69082.97621	376253.6865	0	318178.6642	212958.4833	790590.4687	299206.8036	136125.6018	236058.9014	312841.4299	78746.31304	0
Q9NZ32	2327972.046	469124.8383	32143.05958	1097105.692	1121837.004	239882.7887	778391.0823	554804.6174	210714.6875	1209772.353	1440560.244	1626241.006	164394.4368	1986587.11	1718680.78	2077801.226	1471951.94	749438.0994	976730.678	1348179.953	1140073.737	889155.3204
Q9NZ43	122860.5394	3283601.198	881693.421	922181.9637	530562.4663	331358.3306	142779.5256	0	428674.5572	982317.1091	2646962.285	865340.4285	0	0	1588074.906	311323.5722	0	650148.1989	662312.0631	1459377.942	0	
Q9NZ45	38054851.97	29036763.92	24497910.08	31707946.91	30333959.38	32992095.38	40996234.41	18110570.05	32558635.58	33494341.74	36119014.96	32510826.76	52632213.25	31648667.44	40389138.81	10449893.23	43844285.55	28945215.71	47577746.19	52160184.16	41250168.82	36521227.21
Q9NZ52	179036.5066	78515.21297	0	0	152075.7776	0	287572.0252	0	0	0	38641.42779	0	199437.4888	0	0	808176.2394	46043.15058	0	241908.4292	0	235173.1263	105842.9224
Q9NZB2	5172598.09	3651298.469	6502963.988	4045288.152	4277478.249	2704964.863	4881963.12	3280036.792	3088098.347	4738771.17	6181803.052	4861233.618	3765553.123	4444802.285	6207502.639	7518882.205	7074667.85	4576323.166	5136188.039	5010057.466	4969716.586	3000151.444
Q9NZB8	386673.4151	336356.0635	12695.9531	57703.42326	520159.4225	177117.1322	219104.4528	63016.72221	171958.9544	291788.3003	626665.7082	380937.2755	0	472093.5779	616339.501	440056.0289	701397.814	216970.4608	217284.8391	435886.6267	122110.825	139774.3667
Q9NZD2	2209036.078	1904481.461	1246560.387	1436118.148	2806175.631	815614.1999	791368.0741	0	1386906.16	635990.3778	1269554.283	1428749.727	986339.7496	2458711.521	2070516.427	6918425.374	2505111.267	1616323.698	671390.3531	1374429.883	2230478.588	397033.2106
Q9NZD8	431753.0656	473017.0167	454092.0879	448117.0101	445247.3197	496106.8544	1289159.288	153762.994	89244.02475	778494.8406	1272114.517	1271222.246	334174.3407	692122.3406	1508138.864	1562668.336	1395862.618	1312924.677	402525.2595	834064.6841	1112904.707	639313.8435
Q9NZI7	254541.7656	0	0	91642.28314	0	0	0	0	234183.2739	0	0	198084.4114	126649.0472	0	0	234085.2891	132620.0644	0	60411.81004	89511.13039	263295.8122	0
Q9NZJ6	2514888.679	3856615.281	2063363.675	1475629.941	1757743.522	1459503.673	1169061.866	1555328.431	1982404.562	3079693.854	2276553.024	3630572.724	1865545.285	2982531.304	3632131.828	0	6416712.157	5216885.653	960257.6612	1355259.271	3344550.624	3455774.143
Q9NZJ7	3533265.53	4691825.204	1610082.573	4320239.569	1635251.235	855984.0217	3126863.353	700397.3178	3606953.585	4095526.312	3547579.63	1633302.73	534326.9887	3335730.615	3334373.731	1121545.923	3453486.156	2785761.586	5071379.614	3891518.632	384677.3148	2022319.979
Q9NZJ9	0	1706486.808	0	3351226.438	945716.0639	1930736.513	628502.4361	1712213.315	822729.4017	1682832.585	1689332.082	3201534.966	1573599.012	3342716.775	0	0	761374.1307	893811.2811	1651095.382	3455883.332	2920044.484	1268655.58
Q9NZK5	177270.848	342272.8321	114446.8444	271251.5829	162052.5372	408385.9669	1886996.805	0	540525.0928	457768.5277	381463.961	247160.6911	0	173776.9465	938669.1242	677866.2254	611569.6637	104993.9538	203001.8855	635992.619	2324583.668	0

Q9ULC5	31028963.67	34927019.57	33473133.23	36287932.16	54358662.76	23209963.81	15315097.81	13712388.71	34980424.85	31147565.52	26908551.73	24685215.53	13771951.17	[illegible]
Q9ULD0	7099319.794	9074664.306	5864826.518	7926262.521	3901480.161	3430215.366	2668520.631	1506901.157	5573954.151	9252925.969	6350337.232	10327475.07	3510697.083	[illegible]
Q9ULD2	25455.09369	38811.93408	0	0	0	4858.913915	0	0	0	7076.678938	35776.70581	57952.53503	0	[illegible]
Q9ULE6	0	0	0	0	0	11174.23414	0	70470.09463	0	0	13601.78632	0	0	[illegible]
Q9ULH0	0	0	0	46689.55642	0	0	0	0	0	0	0	67996.15435	0	[illegible]
Q9ULH1	121072.3942	18645.54353	13129.18721	50904.17486	31828.04092	115479.9758	127101.0872	92847.87976	47745.00278	20678.61376	23111.23554	48539.87235	37737.76603	[illegible]
Q9ULK4	28931.70637	35034.30006	0	11105.7713	35836.10807	13639.88294	4112.479372	86908.81048	12586.61033	7444.069304	22383.37118	0	24796.59579	[illegible]
Q9ULP9	2100697.294	1100500.881	1973858.872	2060195.976	2569885.914	1147402.663	1429281.659	1886498.831	1217690.994	2095979.868	1890956.228	3574215.556	745963.8533	[illegible]
Q9ULQ1	0	30724.15764	49518.10928	152227.5058	0	0	87675.83165	0	0	92873.91097	0	222830.17	0	[illegible]
Q9ULR0	0	0	0	0	388865.4908	341995.7628	0	0	0	209469.4803	286788.4685	0	0	[illegible]
Q9ULR3	0	97156.63268	142611.623	187457.8993	146580.6459	150196.9005	0	0	91219.01173	133532.7071	0	70232.69169	136181.8022	[illegible]
Q9ULT8	327010.8376	405505.3689	206820.6916	285286.4065	372651.9901	143850.8746	146255.715	83130.52816	165062.4551	414618.7214	478971.1224	399408.5831	140856.926	[illegible]
Q9ULU4	0	0	0	0	0	0	0	0	127962.8091	0	0	172086.4167	332271.773	[illegible]
Q9ULV0	101780.6617	118528.8048	56410.92214	108925.8489	115132.6016	58065.65491	59412.36771	6170.481706	0	77816.79045	129209.6506	115196.5593	180172.2551	[illegible]
Q9ULV4	8113898.765	7588930.149	5605200.813	7619829.33	5390590.56	7241401.725	8769161.285	2878712.823	1584409.004	5876014.526	9144051.143	5013088.609	5549872.589	[illegible]
Q9ULX6	0	212611.3462	78151.74618	31447.16077	0	0	0	0	101816.1342	265145.8404	121445.7009	0	0	[illegible]
Q9ULX7	1016648.953	3143865.403	1826363.399	2148613.105	867768.9775	792241.1443	0	4345958.058	5511552.246	1421000.746	3014536.541	3107673.428	1691595.363	[illegible]
Q9ULZ3	14074521.37	7099757.039	3873211.321	10493235.64	10741760.52	19774952.15	20733677.26	9251576.572	7146008.394	3458942.228	5616136.506	7901296.664	10796631.08	[illegible]
Q9UM00	8718993.896	3765978.191	1791350.43	625026.0675	2508547.94	2277559.605	1950238.185	128391.596	0	8586190.816	8121036.769	4604749.125	482569.9891	[illegible]
Q9UM07	376637.636	0	0	312179.8447	0	547909.7427	176939.6848	0	597565.901	680369.4034	69612.4328	175568.7807	0	[illegible]
Q9UM22	4744269.276	2221338.068	0	1303037.416	2462773.67	4562270.951	177208.286	65692.55552	1357462.511	1051703.035	633491.2792	2709032.18	90195.59512	[illegible]
Q9UM47	0	20666.04932	0	3030.218686	0	82598.56357	0	0	0	0	20400.03638	15240.41183	0	[illegible]
Q9UM54	1466277.705	1723924.508	1344036.497	1179798.57	1570621.862	1416052.937	2002159.552	830539.4122	991831.0735	840598.6436	1857627.972	1471481.846	1084591.796	[illegible]
Q9UMR2	8253323.322	6369641.084	6732900.562	8230919.659	5047314.536	6012086.652	4974612.983	5436482.373	6051430.208	4393919.164	6327301.379	4285793.828	3175942.701	[illegible]
Q9UMS0	4412691.013	4073682.848	1667466.687	3313710.192	6787229.559	4296017.213	2455364.505	3139816.461	415945.4992	3035131.73	3821548.378	9665141.374	1986587.91	[illegible]
Q9UMS4	7972941.815	5839963.755	4899443.832	6366382.047	4417882.291	6506994.973	6146438.046	4376655.21	6319241.435	7373583.984	9409486.683	8054170.012	5380417.785	[illegible]
Q9UMS6	993798.2583	102379.5947	359167.3673	759776.8578	330312.5451	337407.9179	555991.656	298638.3548	186068.3415	380362.3597	290235.4946	391281.6483	603005.4779	[illegible]
Q9UMX0	16444596.63	11571171.5	11832856.6	15632473.54	26412193.17	8257700.932	19698286.12	7972195.551	11984682.98	16398029.49	20362492.66	22633394.18	4839966.794	[illegible]
Q9UMX3	2438932.985	475213.5311	2056111.976	1650463.382	383464.2778	914254.1489	0	0	290607.4628	1257074.902	1803998.306	1577768.455	188951.6934	[illegible]
Q9UMX5	5178496.381	4399925.043	2475396.579	7716561.856	3283052.212	8399893.442	5278280.411	9374231.583	9805510.074	4762441.709	1491048.965	6073801.066	6059329.428	[illegible]
Q9UMY4	0	1071326.683	2704853.297	2314070.871	1626107.108	4050220.773	4249030.764	2282557.161	2697704.821	3146729.644	2407160.334	2208259.403	3153718.521	[illegible]
Q9UMZ2	0	0	0	37656.00124	0	159069.85	0	146406.7385	58877.51275	39197.22668	23922.93975	170432.4981	0	[illegible]
Q9UN36	59627780.32	87116276.04	75979136.87	120758268.2	66523423.04	67622340.48	60023032.83	36425844.09	56952519.23	83762332.57	103605098	117348011.3	58682418.81	[illegible]
Q9UN37	1385939.497	1269846.642	524567.302	1487957.666	1520919.454	340786.3336	233887.1128	0	57994.79387	2950843.416	1469787.655	2524099.925	0	[illegible]
Q9UN86	3886617.251	2425665.455	5141506.181	5722573.269	2941393.391	3571309.252	3644022.834	3716530.197	3727572.046	4378298.985	5564058.886	5007478.145	5388700.702	[illegible]
Q9UNE7	1581153.724	2062423.519	1491665.213	1231886.91	0	440798.0412	224386.2483	332051.5548	882018.9826	2804335.17	2435183.249	2379469.495	469109.9039	[illegible]
Q9UNF0	5396685.913	4458267.35	6226411.463	7996719.267	9168878.145	6814869.232	4233385.322	8102434.163	4201208.733	4976899.518	6887507.413	4995839.949	2597352.581	[illegible]
[illegible]	[illegible]	[illegible]	[illegible]	[illegible]	[illegible]	[illegible]	[illegible]	[illegible]	[illegible]	[illegible]	[illegible]	[illegible]	[illegible]	[illegible]

Q9Y6H1	5759285.669	2926294.249	6621581.456	2643659.763	2633956.597	8537500.877	957724.871	0	2000517.226	3447591.648	3264992.735	8687119.733	0
Q9Y6I3	4345083.16	3511414.528	5266781.888	3393508.855	3652720.317	2267506.8	6803631.037	2338771.621	1326909.994	4028779.417	9055873.488	7171472.707	1491959.993
Q9Y6I9	593502.8952	939465.4429	535729.3889	792035.5089	814913.2329	499060.7509	0	0	1281103.87	1164045.988	1017926.34	992796.7012	0
Q9Y6K0	1937645.758	1770244.049	1585523.251	2640824.322	3316367.845	1587598.436	1458570.379	0	0	3213525.574	1969008.106	3208464.487	1142005.921
Q9Y6K5	388950.2384	873554.8634	320144.4052	844731.9476	2129307.076	448643.267	1699464.196	353245.991	45606.24055	617978.4863	670137.1155	232309.5068	204441.6518
Q9Y6K9	273782.3669	140532.4414	237564.7307	332750.7094	0	924114.867	630028.5492	314483.7375	83523.78373	308191.327	681643.5862	529245.6159	0
Q9Y6L6	6330160.804	10641550.27	8128051.336	8066101.105	14706535.16	3115021.734	3006388.121	5351534.026	9138786.25	3889947.33	10887262.95	11108544.13	4597195.031
Q9Y6M1	0	0	0	0	25473.69171	37586.93621	0	0	0	0	0	0	0
Q9Y6M5	1151381.753	2641963.635	1962217.047	2402073.377	2853129.443	2671038.717	2478659.264	430049.4671	641220.9841	1292214.835	3724996.537	4010998.252	1709330.861
Q9Y6M9	6780461.182	22304995.93	10611149.56	24922374.63	25847814.01	3656604.167	2879823.024	2615618.333	4514623.843	25676764.53	23848883.11	5662567.321	7207171.549
Q9Y6N1	100846.6872	1353890.894	610323.68	1582325.916	1322927.386	752356.4276	422737.5297	0	196016.5757	2004890.856	1181472.448	1653339.917	0
Q9Y6N5	13625536.76	19621742.06	20348945.73	12577021.33	35124831.79	19738615.3	25456932.51	15107340.53	24045957.5	19152318.42	24060898.92	19337410.07	16600318.23
Q9Y6Q5	0	0	0	0	0	55812.0702	0	0	0	0	0	0	577986.2276
Q9Y6R0	343344.5617	258101.4529	0	0	0	0	0	0	0	0	0	0	0
Q9Y6R1	188966.1557	435858.5917	0	210224.8898	342212.105	515184.2764	272063.4011	450368.7528	309353.4984	85526.10493	470772.943	48014.16856	43910.68886
Q9Y6W3	99431.25684	264398.6893	206168.4084	310287.6576	221069.3191	151528.925	177740.6547	176656.6975	133833.6966	216721.5368	217420.8177	426236.4816	207449.6552
Q9Y6W5	4355064.642	2347896.027	1358055.549	4054227.601	3787714.038	4304954.152	5352058.312	1906390.445	1839434.686	2151154.156	3792544.058	2553459.709	1110178.515
Q9Y6X5	317091.2249	157933.5129	0	281757.0363	252257.633	295485.6283	0	32448.39265	52699.29346	431728.1517	533015.3834	440398.1713	0
Q9Y6X8	25937.20249	0	0	93803.55264	52713.50908	21466.29121	41747.338	37528.13357	37510.98061	0	0	47498.38171	27003.05633
Q9Y6Y0	0	0	0	0	0	0	0	0	0	99114.70612	415993.2033	0	0
Q9Y6Y8	3336561.473	2711466.708	2983011.75	3405039.502	4015577.498	1478889.354	1966359.389	1410502.055	1997913.88	4526921.388	4387438.258	4111423.403	1812326.498

Q9Y6H1	11332149.9	15443825.52	10702002.14	4033751.7	10191095.29	1594927.632	2836539.388	0	3089918.602	8388548.905	6423901.692	4647598.272
Q9Y6I3	5935245.782	5162063.895	4529269.685	7238425.395	5908784.781	6940833.408	3843595.123	3757371.48	3018228.953	3935259.83	7213139.564	5713733.712
Q9Y6I9	226916.2473	1027522.593	35533.96229	1824412.71	532983.2655	889824.1908	810088.9321	1044163.417	1452999.56	900104.0371	1136264.077	1638356.931
Q9Y6K0	4031240.006	2800899.029	1854092.838	4038481.874	3260292.535	2050489.779	2883674.295	2175383.543	720172.3094	2541656.945	2327977.076	1730641.397
Q9Y6K5	4110313.889	1163123.106	1937882.42	1933779.957	1431329.576	587072.2752	742401.0315	3144914.486	254915.1337	1030082.021	1753784.283	205954.9276
Q9Y6K9	1208565.218	924386.1549	2944209.915	919759.8595	664428.7496	0	84843.98676	1293801.118	0	349182.7614	546574.9742	315546.1992
Q9Y6L6	3858412.984	8483630.789	0	19374969.08	15028594.63	4598233.88	6755338.6	6805067.037	3761483.311	4735254.722	9058675.68	5721502.073
Q9Y6M1	20390.47383	0	1966048.875	0	0	0	0	0	0	0	0	0
Q9Y6M5	2516239.491	4935341.273	222582.9211	3195931.362	2531293.652	778900.0396	1664462.143	874223.0412	1301198.844	2123555.095	2467837.164	2836630.74
Q9Y6M9	13443173.12	22656300.64	8350941.453	17560342.47	28541603.93	15402543.44	18904745.38	7537360.782	7171769.806	12456230.22	26911591.57	14958245.38
Q9Y6N1	1213230.193	1702567.949	819745.3323	1935686.905	1849247.023	1031010.336	1261686.211	279565.6347	509753.4217	669358.5685	1526454.551	324075.1247
Q9Y6N5	19607138.55	29895819.88	36873185.21	38953162.42	23684278.72	8125336.826	14339760.92	32170551.75	21433670.35	25172776.18	20013249.5	8727034.052
Q9Y6Q5	0	0	542390.259	0	54913.95556	0	15285.29867	0	0	0	0	0
Q9Y6R0	452497.7517	0	310650.2428	320561.325	0	358791.9796	292533.9994	0	0	112570.4969	244249.0972	304250.3039
Q9Y6R1	178415.5118	890476.9112	1819087.469	484402.1215	523177.1224	208206.1175	622955.3781	0	992572.125	795910.7081	365469.3568	720167.3248
Q9Y6W3	171382.5832	281426.6208	148450.4563	236468.3437	374133.9209	120010.9248	364824.8282	184213.024	120115.2399	344685.1996	443082.5513	272689.5257
Q9Y6W5	5888757.031	4929491.001	5598459.739	4776864.305	3150442.039	2905594.885	4898893.607	7161130.603	4482258.056	2061362.316	4153853.486	2615689.888
Q9Y6X5	718944.5828	501646.2815	1148372.511	538486.8212	485837.0118	0	361755.2675	315003.8022	121025.161	0	738059.4514	292734.9088
Q9Y6X8	0	0	0	70740.38211	0	24047.14171	151541.0035	39026.0826	30427.77191	0	0	0
Q9Y6Y0	164641.369	58319.67724	1184260.61	55060.18508	179073.1592	0	77564.06319	0	0	0	0	0
Q9Y6Y8	3307881.063	2817935.461	4856265.826	5408248.135	2334235.728	3347765.539	3214733.406	3509930.621	1283378.675	3276664.264	3152835.504	2509832.762

Q9Y6H1	6905336.761	3293642.851	14588861.18	12133801.97	4755458.004	4045496.921	0	14733455.42	3906219.672	3064031.39	4668146.636	8938124.782	8848324.225
Q9Y6I3	2707748.528	1503036.713	3672419.694	2972712.686	4995332.904	4016733.212	4140612.289	12749647.8	6892486.587	2593259.666	3501881.174	3111810.445	1541846.065
Q9Y6I9	348864.3027	1311888.701	989322.474	1972374.287	897909.1997	2538301.162	712781.914	2123520.965	0	1441155.461	766218.5741	0	705416.9595
Q9Y6K0	1901628.496	2897529.264	2542277.526	1157115.177	2609242.984	2752138.45	2124217.213	3260074.121	2408164.381	2189263.41	1718412.58	2352783.793	754972.0874
Q9Y6K5	403141.9377	1497917.389	425181.0303	303021.5347	726038.9696	1156994.83	434247.9315	685321.2263	1649412.648	536918.3726	392404.1072	675531.1658	901879.8603
Q9Y6K9	334909.6819	503128.6862	335467.6101	444635.9904	297301.9286	482682.1655	83236.86432	476795.2057	0	0	520654.2553	586898.9301	0
Q9Y6L6	3700083.136	6082065.628	7280393.085	9310777.651	8137531.95	11944151.62	8613051.294	2475167.122	7187617.326	11720944.21	9007605.629	7133980.554	353173.5852
Q9Y6M1	0	0	0	0	0	0	0	0	0	0	0	0	1123299.32
Q9Y6M5	1277124.709	2907970.739	1338461.111	2120370.363	2060587.646	3060260.162	986351.3262	3427017.054	2248538.698	2418763.924	1720550.836	1397631.676	879060.0551
Q9Y6M9	14338383.78	10319492.1	12577057.25	7793760.582	16542073.74	14081733.21	8925306.183	7198006.852	15112372.52	26422292.75	13553478.18	3175455.422	7892401.769
Q9Y6N1	425907.604	729162.5931	280849.1935	269761.2976	1058499.838	1458264.533	752242.9095	622353.1681	1028208.955	2039782.495	295040.4854	517013.5671	182045.8992
Q9Y6N5	14813117.65	24919505.82	12373772.1	19608219.61	15449896.53	18572661.86	8058452.244	6971158.645	19506496.42	22864827.88	5726782.772	12204078.72	30407026.9
Q9Y6Q5	66880.23296	0	0	0	0	0	0	492777.5739	0	0	0	0	2380533.799
Q9Y6R0	0	0	0	220770.0817	0	335472.5918	0	0	0	299419.3674	414288.3607	0	0
Q9Y6R1	749891.4827	0	551119.6735	1075149.774	832332.4923	74586.81073	1188373.878	544189.9967	1100375.36	551453.4399	207170.7025	765962.001	72750.07395
Q9Y6W3	201094.3107	239775.4683	122255.899	126791.5756	189542.2991	181889.882	260074.5536	89408.62807	317742.5732	165609.4275	68041.74246	91925.57737	154035.9016
Q9Y6W5	1518110.364	3078361.484	3551128.333	3125683.441	3170275.925	2660948.643	2942373.234	5525430.677	2868105.651	3313947.39	1701883.463	3204301.976	4596262.215
Q9Y6X5	110181.5968	0	113934.5508	472725.1301	0	953087.9224	632835.2677	316034.4343	281785.7995	323226.0135	333229.8983	636736.7848	553364.2561
Q9Y6X8	0	0	0	0	0	0	0	52274.30162	28091.20118	45166.54266	0	0	0
Q9Y6Y0	0	0	0	0	0	0	0	0	0	0	0	29451.89463	763294.0323
Q9Y6Y8	3423986.522	1143563.336	2500990.247	2567226.317	3123170.754	2286286.572	1618611.153	1782072.834	2421456.988	2934593.437	1722509.165	2202527.343	4102553.077

Q9Y6H1	0	8035958.409	15884994.85	17552031.48	16203294.81	364369.1973	8662947.429	8606822.736	7872185.833	2079875.527	2746920.855	2053611.899
Q9Y6I3	3572014.472	5320845.741	5577750.32	3356598.849	3236697.868	4239043.58	5754583.538	3069056.876	3363515.268	2572087.026	3466962.721	2477362.687
Q9Y6I9	96808.14454	1151444.645	536807.3441	733827.6026	619133.2606	499608.7969	1174900.36	870710.3847	436891.97	1294555.22	682551.6283	0
Q9Y6K0	3017795.134	2492721.75	2585731.29	0	1715354.148	2704164.472	3567878.661	2847816.759	42476.14343	3758949.008	3053624.197	3442059.856
Q9Y6K5	1518523.838	2069944.418	316136.9657	236845.3945	826216.4911	521454.5237	714733.1941	911907.3206	721949.6723	1793716.816	2440118.858	162927.3353
Q9Y6K9	913226.8734	755533.0726	665494.891	393535.7354	482927.2608	0	1076856.762	224139.6072	388073.1069	0	371317.3702	0
Q9Y6L6	11618877.15	8421447.397	13891592.45	15303427.36	3873603.735	14221361.5	1999840.314	4098971.93	4711646.293	6833774.378	8048077.292	6851328.242
Q9Y6M1	0	33496.03022	73133.42656	0	273092.4775	0	78385.76785	0	0	0	0	0
Q9Y6M5	2582898.568	2424243.47	1836391.527	2968845.73	2357624.591	2626898.206	1607432.083	899839.4916	2305512.091	2026388.508	2034009.65	1389234.878
Q9Y6M9	12788898.97	12050307.43	18296366.52	6021195.874	6644138.677	22736984.04	18662763.11	10791222.71	19918010.59	10557284.21	21573074.15	22085229.6
Q9Y6N1	943453.004	0	544744.7222	223405.9522	28197.83064	454251.6684	444899.6402	302057.9984	756052.6184	46571.22178	837236.2498	1539136.178
Q9Y6N5	7308651.015	12803590.31	26451027.42	9419066.126	13633699.53	19611242.24	33277387.55	4767578.455	21785067.74	22336066.96	9021951.61	13548973.2
Q9Y6Q5	0	0	65027.05298	0	94964.24813	0	0	0	0	0	0	0
Q9Y6R0	0	0	0	0	0	304399.1367	0	0	66906.39796	658409.5382	272219.8091	181447.4877
Q9Y6R1	0	945829.7388	1000559.642	743511.2194	679398.8189	195553.5771	1069776.478	690155.5421	40889.7034	11405.95673	221597.4407	422126.5878
Q9Y6W3	109045.3003	317756.5979	188584.1608	159058.1948	396679.4018	143486.0261	250397.6416	52078.18463	292911.5174	287325.2109	257596.2761	270760.358
Q9Y6W5	3471855.717	3628469.538	1404140.017	2277976.406	4947961.195	1127321.561	4132214.529	1303353.467	944725.3883	4078257.879	4046358.118	2758205.893
Q9Y6X5	0	481895.8257	204649.117	314069.5824	468020.99	0	345870.6604	231522.6232	0	255397.739	450787.3026	622332.3618
Q9Y6X8	44483.01921	0	60802.65514	22295.83377	39864.16862	15684.62135	112934.9808	0	0	0	0	0
Q9Y6Y0	48699.97899	0	13967.75286	102794.6733	0	0	0	0	0	0	73586.01832	0
Q9Y6Y8	1932864.6	2366088.913	2672521.481	1898567.025	2597582.545	4211167.883	3478431.428	896022.5039	2313907.359	2414126.606	1979206.66	2114818.062

Q9Y6H1	5914289.325	1507368.807	3760202.676	24700915.47	3153827.262	3357377.416	5501024.262	0	2718028.431	0	8843243.034	5149183.152	4892266.853
Q9Y6I3	9418331.829	1453581.255	7593437.753	6850229.562	3227861.047	7180574.319	7219526.569	6642952.644	3472736.817	2510495.877	1930082.602	3033153.197	1374123.483
Q9Y6I9	782168.2426	0	2682600.649	77712.58576	0	2289848.702	0	445876.2283	0	2177839.453	784536.972	1016194.106	678685.1939
Q9Y6K0	2141135.387	3534368.444	1872582.103	152445.8029	3145659.227	2078616.126	1597340.887	2666599.03	2123714.417	2972174.748	1126296.105	3786559.345	3164038.304
Q9Y6K5	242919.5851	1536601.745	1269450.749	8313888.874	364902.2117	1027206.781	1435683.103	294179.7072	688387.0605	623231.2251	888985.4021	620519.2032	1242948.875
Q9Y6K9	643690.3312	485732.6493	683369.626	1637895.556	0	535221.1381	479372.1566	240208.9545	356088.4921	40911.98634	726758.701	183519.1193	0
Q9Y6L6	1928156.975	9230887.184	4805195.52	0	5766594.686	3083168.532	11309926.15	8255067.632	2883417.08	9643983.292	1504164.738	3520053.913	12026320.76
Q9Y6M1	89249.42102	0	0	8590823.668	0	0	0	0	0	0	0	0	0
Q9Y6M5	2304943.498	1633803.245	5693171.904	817776.6327	1235736.133	3577719.348	2117972.228	2229673.526	812525.7439	2769963.089	1353841.141	2103610.023	1537134.614
Q9Y6M9	10217486.06	9561940.584	16043377.6	9735433.852	6974687.947	21516460.56	22508135.7	20592079.04	19619994.21	17019815.33	10804907.58	16117698.38	35652364.01
Q9Y6N1	366526.6392	384008.8065	1152450.975	188357.4445	782059.0666	633863.5723	195856.4839	960107.2056	182190.2284	562830.3922	323409.284	681005.8252	1163372.562
Q9Y6N5	21729800.97	13602203.91	54299380.77	54381276.66	6448270.174	23283141.15	19441101.42	15646026.09	6808276.582	24139363.23	11366213.84	20382423.54	31675898.87
Q9Y6Q5	0	0	31111.43753	9941346.078	0	2519286.399	2646827.227	1190285.812	2063600.604	1152933.365	312714.8055	31065.21787	0
Q9Y6R0	0	0	305057.4786	0	0	565427.344	546542.7385	0	376087.4037	363632.4149	907104.2758	0	0
Q9Y6R1	322488.5637	1199714.563	391935.4739	730346.4188	1005664.299	549643.3551	46415.25429	359187.8395	492112.679	0	614896.8384	473581.7326	538235.0417
Q9Y6W3	309293.1006	91813.18036	536167.1476	544242.0347	45124.27729	459177.3131	530913.496	658369.0908	481420.7803	320708.2797	155699.831	365619.0135	379611.8543
Q9Y6W5	2253842.581	4327658.661	5846883.837	10052449.34	1872907.073	3029668.189	2985991.753	4027894.461	2781780.568	1620742.931	2802238.788	4238259.157	2555700.938
Q9Y6X5	161948.7041	0	1079095.623	1154491.198	0	0	262740.1616	584846.9441	234435.2472	397892.2125	908040.8244	616362.1401	0
Q9Y6X8	0	0	0	205875.6654	0	0	0	50078.55253	19133.53404	20861.68919	0	79022.80793	48866.00525
Q9Y6Y0	18612.71261	0	528158.38	1190606.755	31180.04428	274238.7681	0	0	0	0	0	0	0
Q9Y6Y8	2537543.411	2423955.724	5536896.929	7581600.149	2268400.488	3461371.575	2805597.993	4829659.361	3529389.664	1941402.83	2197507.267	3614738.097	4649348.196

Q9Y6H1	5015441.069	12039355.72	0	3385398.539	0	3198445.405	4844529.033	6482574.65	4468256.303	3694519.514	6874279.476	8559408.124
Q9Y6I3	4885808.36	8618609.493	3750971.916	9616989.49	4732635.759	2262019.537	5086614.386	2513122.055	2757373.152	4941936.877	8241293.175	3144085.431
Q9Y6I9	1714386.189	1461061.895	0	1486933.472	742800.4223	615644.9772	470907.313	704530.9854	1456267.113	216953.3805	225586.8107	301679.1736
Q9Y6K0	4978819.73	839481.5775	2871071.353	2562209.092	1848193.883	1734398.366	2226529.033	2606505.646	1644589.024	3184403.873	4002088.627	3724461.356
Q9Y6K5	1007988.428	2809636.173	2390853.453	3854044.485	1746706.244	283657.9244	1125437.686	409233.8527	980196.742	984625.2517	1424179.045	1320746.349
Q9Y6K9	573230.7895	2112865.493	1405425.177	1056541.361	0	0	260133.6177	0	553240.1624	224246.6843	590447.6819	475342.583
Q9Y6L6	8889882.619	1073688.499	5037922.993	2570665.419	7389570.019	7122508.822	2430995.975	5492067.242	6815789.642	14112058.47	12485876.02	7372797.762
Q9Y6M1	0	151785.7735	235812.4561	33041.40615	0	0	0	0	0	0	0	151705.8937
Q9Y6M5	1687117.269	1458556.936	3565423.792	477214.0913	2572764.436	354538.0384	1373373.086	796250.0103	1553124.285	4123808.804	4301993.894	2922700.338
Q9Y6M9	11127329.62	31012094.01	21442969.75	12228306.55	15157477.86	8085602.039	8153123.638	15008760.31	27786236.22	21626220.32	27988387.43	11582580.51
Q9Y6N1	1453515.288	2006760.732	1083940.297	2223455.688	706219.3574	1209258.068	1230125.154	278291.2406	1115148.294	417506.1484	1707667.861	1446078.94
Q9Y6N5	20644020.28	10933998.18	15323540.6	24711857.37	18556758.98	19390860.64	19123315.96	16054440.45	14274225.52	18749544.83	18327697.6	28523681.33
Q9Y6Q5	47912.91722	327468.6344	66871.38713	0	0	0	0	0	0	0	0	39022.07486
Q9Y6R0	246928.9383	236823.9781	0	250951.5059	411946.6945	280779.2409	682121.3408	389355.0696	0	105462.6266	0	278636.9932
Q9Y6R1	760237.401	624503.1561	523369.5316	58667.39236	54509.42038	155215.8059	118761.6154	116604.9529	104199.8928	276151.1066	539930.3343	414519.9651
Q9Y6W3	221758.0509	321936.1232	198132.3876	42901.66047	467819.375	11840.3875	29096.57034	74030.16819	190021.7333	383012.4003	292703.7332	126965.7409
Q9Y6W5	7032446.394	9210215.884	7050030.587	4334613.956	2713148.139	1717231.268	4645926.27	2233035.776	3317388.915	4176067.192	5763681.828	3198286.235
Q9Y6X5	1321091.144	827946.4265	0	0	138278.8755	293354.0445	361028.2628	300700.458	494889.9289	1217147.226	1132633.139	930235.9758
Q9Y6X8	52980.06346	154230.6147	252296.2651	58064.1967	62062.82382	0	0	0	0	14109.6262	0	40308.95975
Q9Y6Y0	110057.4361	212244.409	197620.3414	0	0	0	123061.9838	0	0	182990.4754	15991.1865	0
Q9Y6Y8	4895438.746	5217855.607	3944778.735	2236104.424	3045238.388	2531715.473	2149445.147	3065499.016	2637430.56	1910710.686	3082939.658	3245934.554

Q9Y6H1	3576659.137	9723108.211	4727842.576	0	2908027.055	3183380.692	4602630.49	9767569.731	4632382.264	4888112.916	2257564.554	2999812.414
Q9Y6I3	3377226.845	6001505.063	10276152.35	7629525.263	2900972.576	4245205.024	5927885.011	2491099.62	5733177.726	4517542.162	4081761.18	5876398.836
Q9Y6I9	868053.191	925881.948	834435.4864	1483494.837	579925.1182	536027.1604	0	810268.3647	0	882142.9226	385290.7514	1743147.521
Q9Y6K0	2212848.881	2620274.155	1532839.608	3581311.824	2739699.32	2083645.312	2372543.635	1699677.199	2745091.182	1569474.52	1844538.42	2609671.783
Q9Y6K5	231440.6651	1258237.86	2264199.578	668592.6205	1950289.631	275133.5985	814666.7574	2055440.972	275028.745	565605.4798	148676.8302	537601.1852
Q9Y6K9	0	808998.3087	398795.0293	0	441519.7912	0	0	0	200811.4772	51474.58943	629823.7152	116779.4436
Q9Y6L6	12769011.64	12965016.41	7078693.22	11906679.16	8132806.844	9605380.358	7432464.145	9221603.655	3013262.899	10866677.73	5932289.902	7214213.248
Q9Y6M1	0	46328.81958	0	0	0	0	0	0	0	0	0	0
Q9Y6M5	258398.6205	5327308.838	3749813.823	3346550.173	1630900.427	2039770.256	777523.163	273302.9534	1157619.228	636294.4625	1495771.067	637063.6147
Q9Y6M9	14802343.99	16776721.43	9547925.613	29107222.36	13755616.86	11819850.08	12620336.97	12457014.54	11423641.89	18428287.69	13153250.4	12741760.47
Q9Y6N1	334723.5859	1287226.774	348696.7031	1927104.935	805073.3726	445043.6676	623067.485	883917.1092	593990.6604	258089.6878	622939.589	695103.5103
Q9Y6N5	9705768.495	22389127.72	14913792.32	22570349.62	23604253.96	12361398.33	13738743.67	6207869.828	12856225.65	4232456.658	14732011.28	19520654.17
Q9Y6Q5	0	39456.3311	0	0	0	0	0	0	0	0	0	0
Q9Y6R0	0	430521.5282	186734.2363	169492.0026	191901.1135	357606.3638	0	420730.4383	0	0	0	0
Q9Y6R1	991712.2932	409672.8531	673190.6052	229283.0791	290352.3976	781560.1359	459794.1013	1061301.838	845216.8202	980853.1643	418426.4746	348782.9168
Q9Y6W3	109454.8174	365441.9908	303582.6616	190095.1225	332314.5793	191865.1066	185909.5905	245421.981	134979.4021	0	168444.0898	386692.8414
Q9Y6W5	1692442.851	4417446.146	2349603.061	3961367.372	3102323.21	2160408.077	2584016.663	1518262.706	3233179.646	2602921.07	1683638.106	693329.4852
Q9Y6X5	118889.9248	871990.851	96121.17119	166994.5503	150787.0984	124902.7892	168000.395	254126.0892	463346.2597	185956.0079	121199.3091	105476.3456
Q9Y6X8	0	0	14673.4734	0	36789.41433	56615.88065	26290.67093	31601.11646	28532.37479	0	0	59886.35812
Q9Y6Y0	0	36242.38769	0	99835.17315	351347.9644	0	0	0	23198.8906	0	0	0
Q9Y6Y8	2896772.921	2551215.318	2954084.143	3655956.085	2467322.152	3098593.772	1703906.506	2394628.565	1579643.691	643444.2136	2748744.539	2826699.887

Q9Y6H1	3802094.015	0	3466110.069	5991182.401	5833220.307	0	2377523.087
Q9Y6I3	2493138.394	1519942.17	360317.5996	2516793.671	3798039.248	8848844.774	8965241.061
Q9Y6I9	186423.8162	0	0	0	0	2937439.463	1535105.35
Q9Y6K0	2431884.381	2062190.712	0	1255283.314	1403166.427	1330977.056	825941.2496
Q9Y6K5	770789.3239	242815.2587	0	47549.79989	132387.3319	480079.3855	471726.8906
Q9Y6K9	190130.9943	333050.0905	0	458421.4535	0	570877.5683	160978.2356
Q9Y6L6	6408551.544	4833400.497	3385467.826	5282153.628	3300702.939	13520694.38	9438901.062
Q9Y6M1	0	0	0	0	0	42634.64315	0
Q9Y6M5	1170503.845	2528022.915	0	1327638.265	1833542.207	1794104.851	1928249.941
Q9Y6M9	18149778.14	8574676.848	5383730.866	9321927.518	5857669.842	10417730.06	19280037.38
Q9Y6N1	539155.9743	0	0	0	0	1117965.156	985063.5765
Q9Y6N5	14716906.23	7703709.211	10992972.42	11742197.48	12940427.13	17159851.73	15377222.65
Q9Y6Q5	0	0	0	0	99534.88543	530396.2673	235110.5397
Q9Y6R0	0	140688.0544	170908.5518	0	181891.8777	0	0
Q9Y6R1	385977.9906	335801.5096	110835.6452	31920.09385	554872.6643	46306.22057	0
Q9Y6W3	346420.7291	181204.2055	30768.1562	132385.3809	122789.0395	221983.3231	423736.4388
Q9Y6W5	2487561.737	2604611.49	543522.5379	1264384.662	2629693.554	2294964.458	1331940.222
Q9Y6X5	100875.3518	54089.75949	0	0	256453.4835	0	110488.163
Q9Y6X8	0	37692.406	0	0	38973.39806	38459.08024	68819.96871
Q9Y6Y0	0	0	0	0	0	0	0
Q9Y6Y8	2857172.882	1863502.505	1119937.232	2229539.452	1752629.51	2464783.452	5203490.476

P196	P197	P203	P211	P214	P216	P219	P220	P222	P226	P231	P45	P46	P55	P57	P68	P71	P86	P89	P90	P95	P102
587033.1923	923371.0367	699126.8461	4551915.021	2611633.718	2613575.852	2614839.89	1321454.716	1132754.16	342085.5159	2081148.416	426496.1064	1167699.185	1485051.317	542225.4792	2292818.315	3139366.925	978477.4454	2760041.638	0	1585153.52	0
2939972.786	0	0	0	927020.6815	3362739.242	2585626.993	0	0	1316796.322	0	0	4417825.787	6043978.032	0	1861838.685	2382452.704	6218311.158	2353045.605	1152116.071	10315127.26	0
4434480.613	6713725.639	9572755.831	11029836.3	6259504.709	3070149.271	8824228.165	3824334.615	9085866.42	473111.3413	3092207.102	7107356.452	16580718.3	1344735.1	7072811.96	13611476.81	4998141.189	10333389.64	3281301.345	6464298.862	0	14820823.21
35070813.27	26702213.47	40309234.43	23464752.19	31830172.02	15803434.32	76358161.55	19611258.84	12770635.36	26106363.15	516104.8972	46081345.31	0	31671779.33	67429509.45	31258388.19	13951845.57	81936762.77	35096057.68	51901040.06	58870882.54	55008927.8
0	0	4304041.377	4814277.892	3359180.71	0	1775192.431	1467597.821	1059192.089	2066123.179	7099580.112	0	0	1510918.903	3558713.044	1827606.323	1882745.68	8395462.404	3573061.332	4955124.92	4299611.855	8124051.879
34686800.34	27250883.1	40427401.04	23604510.2	31687825.91	15859161.71	75311798.44	19472883.76	12656821.13	25984470	0	45081871.05	170984.698	32044951.74	67968876.48	31137753.53	14034718.98	0	0	0	0	0
1683817.428	3225037.432	2090579.514	5763238.626	14462040.04	1999013.174	1177556.938	1154039.599	1241114.827	2191278.981	3032208.623	6160215.809	5156704.352	5565898.996	7752022.959	0	2481523.998	0	3182812.042	2956202.369	5755046.655	3867536.184
34876416.79	27180217.97	39986006.3	23417177.03	32022514.87	15639296.09	76153507.14	19510677.86	12637786.71	26163100.17	0	45576152.76	0	32190063.87	68158911.46	31498915.02	14013319.28	0	0	0	0	55164826.25
35173188.34	26839684.62	39880595.06	23557297.98	31955678.94	15749716.88	75516410.95	19678484.06	12713609.61	26328026.97	514287.9822	45828882.37	0	31748303.55	68366597.2	31193836.91	13891748.96	82419917.66	34942311.23	52023410.52	58516541.33	54703026.42
34971980.34	27044854.84	39779578.84	23326042.62	31485668.53	15722734.75	75107525.38	19545301	12618705.99	26267065.66	0	45445697.81	170440.951	31969565.33	68569295.82	31437157.19	13971551.78	0	0	0	0	0
0	0	0	9762784.995	11216486.04	0	20526448.48	1811329.656	648810.234	9245859.98	13045197.98	9575851.447	4826071.268	6200700.4	4917012.094	11556657.05	0	21223627.99	17203318.57	14175616.62	11865161.01	24517639.1
0	686490.3244	1446750.645	1922128.254	5366232.58	725109.1887	937741.8256	0	706688.0642	684424.7933	1553020.132	2043361.057	823181.2071	431472.6419	1095744.61	1000680.041	845113.6425	1491311.218	1878756.152	0	1721775.177	931877.8066
0	0	0	1589541.291	2971899.017	0	0	0	0	0	4108465.463	1232833.233	2392729.176	422269.9051	1394499.713	0	1987936.512	0	650587.4223	1689009.845	1670312.008	0
475401.3229	364111.1307	0	0	0	0	0	0	0	0	1378771.942	1813469.003	0	0	3374398.116	0	683838.8207	0	2101442.679	0	2437134.727	0
0	0	0	2957098.299	2975178.194	0	0	0	0	0	631382.8355	1383586.363	2389878.519	3130853.777	1392940.816	7115275.307	1983780.36	7777419.451	7434959.477	3682129.617	13940568.51	6034864.455
30651655.2	102208901.9	52159655.36	102382317	88141715.31	61844099.85	91632390.29	64610501.91	91725847.74	81143284.14	95892787.33	89034965.37	90881890.64	66295477.27	72241188.65	75286831.62	67499121.67	97596006.76	65781553.82	98055788.21	62550142.61	103365502.8
0	453014.3285	1643792.73	0	1700084.804	910924.5595	1864222.461	946376.2889	937073.9716	947692.1031	0	0	1935374.379	689213.6345	3097149.293	0	1271523.324	0	0	3161171.701	682391.2096	1245461.512
2334498.22	2703951.039	1641876.919	0	1707206.289	913042.339	1869939.067	942150.7906	936039.227	945566.1013	1366272.28	0	1925751.45	690055.0052	3093674.764	0	1266487.361	4885605.005	0	3164589.876	3361585.714	0
3544446.065	3805128.408	825547.6612	2552174.213	4518712.236	785573.456	3156926.497	1026763.5	802014.009	1866941.659	3016999.138	3395295.618	1815556.676	0	3001313.023	2941057.693	2227603.268	4263254.723	1231497.091	1890201.582	1314499.246	0
0	0	0	625093.4206	8471970.203	612848.6144	0	0	1456085.602	601242.9794	3337471.028	1348673.297	856513.7847	375831.6534	5272186.204	861507.3914	1694712.004	2569641.202	679050.5321	1798086.726	941929.4851	0
15130054.52	17381340.33	15230830.16	13039023.51	0	5652273.615	27957624.49	7893923.806	8307597.663	15049348.96	0	0	22720884.63	11496483.5	13030346.65	19331240.13	5838221.606	0	6673112.002	5494569.356	0	11944822.77
8920556.592	8942072.116	0	16937676.95	810659.8023	12422030.17	29284490.78	6458547.609	0	0	2103322.272	28014821.47	12911750.35	21177778.62	26512434.78	19419190.18	11695039.42	41504537.56	12900048.89	21228293.62	22287629.89	34672771.45
1681533.586	3229643.104	2093387.743	5770867.147	14482394.08	2001316.454	1176453.457	1152644.711	1238296.551	2196118.531	258788.5647	6142508.407	5149645.673	5572978.227	7762635.028	2863123.982	2484232.848	19675705.21	3175750.354	2962865.231	5763482.791	3873098.67
0	0	4309666.098	4820546.207	3355613.379	0	1777124.682	1469176.956	1058137.611	2063893.799	7089249.743	0	0	1509078.374	3562906.131	1825873.9	1884780.595	8405278.815	3577464.954	4949259.39	4294083.589	8112273.398
1686038.596	3234298.685	2096065.678	5755385.232	14502602.76	1996585.193	1175292.154	1151319.099	1239668.411	2193706.815	0	6151274.345	5163719.07	5579812.733	7773300.357	0	2486751.566	19708537.33	3179274.972	2959657.374	5746352.621	3862589.267
6066033.673	3772296.834	2952570.996	2060589.887	17751096.1	2202413.154	3598849.052	2910536.236	1797366.27	3461743.875	8383594.252	0	5746001.546	5081437.084	10517326.18	3458146.537	6983081.173	16975692.38	6516165.56	6209930.109	9915624.766	4518132.794
6075796.535	3766933.6	2948693.531	2064967.462	17721713.04	2199831.265	3587459.672	2903282.898	1801373.57	3466619.052	8394950.519	0	5754686.139	5067187.868	10502542	3451058.343	6965602.893	16922412.67	6532770.81	6194704.107	9884110.969	4493573.958
69144735.49	50887617.77	40873835.19	28001398.03	68133862.96	27352986.2	53853426.53	35109046.94	42623614.16	34354138.8	27000284.86	28400742.07	11835044.33	14898745.45	29720233.64	28201060.3	21878621.87	26785960.52	5482614.33	17971820.94	28546576.31	56861557.82
437350.0211	918544.5634	827637.5299	1399267.143	1620497.382	1004258.491	1050465.745	1474272.758	1675474.185	760805.1849	533153.8539	264109.2437	896477.285	595357.701	1273202.298	1280831.138	986131.417	2556020.431	949579.6468	1307897.415	237869.6844	474036.4112
1278395.404	2138512.022	3364603.669	3315339.957	5796522.035	2929818.516	2692110.68	3335719.333	4410511.083	3194335.084	3587204.109	1720972.968	2335204.511	3262145.665	3771810.442	5023003.345	3469836.614	3399901.401	4606613.947	3208050.564	850722.2263	1109711.68
552206.6694	605421.7307	1049423.47	1331231.608	2786046.712	998912.5025	1533658.115	1336996.727	1173269.116	858209.5087	1786653.612	1795843.454	2171866.055	1969411.11	1730991.73	1931838.29	1963406.121	2322732.698	1535400.321	1767764.921	1135163.55	985685.2863
2821276.62	4027516.799	1923996.722	2264593.34	3378082.077	5466554.695	3471900.858	4679396.254	4224527.809	2889060.595	1577138.374	1582294.108	4119147.588	4206639.909	2387396.03	4254062.837	2839897.311	3014461.699	2396608.979	3320899.388	2816581.813	1583805.143
9722171.501	7044700.663	13241533.92	14598723.6	6029439.658	13435311.83	4414287.7	20861027.11	20549546.92	10565707.42	10764959.63	17949704.76	14131189.45	18776320.38	18446160.54	20733627.22	17205118.18	21720012.07	19774658.9	14072753.42	14401777.83	18259215.55
0	26480.52335	0	78579.06612	0	22261.70869	73053.04781	17769.9116	0	20293.8981	18515.93828	0	38164.29643	0	182691.6704	121117.7654	154211.8863	231966.0116	48620.86727	138850.6345	49466.67341	39455.56745
8069.425058	0	0	0	569763.2432	0	65509.48032	0	0	0	0	0	0	0	0	0	0	0	115102.3206	0	0	543723.9842
0	0	0	0	0	0	0	0	0	0	0	0	0	55716.78249	0	0	67775.03542	0	0	0	0	0
34592502.53	26976141.15	39563593.2	23655959.44	31556016.16	15777302.81	74876371.74	19714979.66	12751161.51	26440628.27	0	45329565.73	170725.4478	31602039.3	68800643.63	31381453.11	13992569.67	0	0	0	0	0
234695.1811	59482.54142	202691.3441	5494543.03	270263.4696	6384182.166	7865460.813	408575.8336	342979.5641	3009423.791	2417927.192	391314.5878	271542.9254	505405.2528	4328197.893	7984068.194	425472.8529	589401.2484	11128358.23	526135.7034	149842.5956	225732.7174
0	0	43932.9044	0	66764.8258	63433.52371	369280.2025	49712.35267	0	0	46521.37197	148711.3876	0	0	16665.57937	17871.44982	40573.4748	0	0	0	0	0
20277348.19	18576033.21	40646103.78	37448511.07	2565117.987	57006615.01	6705463.578	57076068.31	47221586.99	62107206.27	63356516.03	38334858.93	49651470.08	52614084.32	32029565.68	49540566.02	34928289.57	36060280.46	42678113.06	55639035.94	32025392.92	30517707.4
833810.3145	749216.7594	2944337.964	5044438.318	2422142.669	1994343.393	3271667.289	3820141.926	2305733.93	3718702.338	2138527.655	2622372.751	4015920.964	3010968.48	3971319.111	3435846.886	4126048.923	3073550.388	5040239.424	4582591.141	1734459.685	743880.3792
0	0	0	0	97246.72752	78251.95717	377910.585	0	68677.70862	0	0	0	0	71560.26702	174943.8937	207895.0749	0	177585.0988	233580.7151	97811.45934	0	0
101157.8538	337230.7163	587398.8465	486045.631	1017110.809	1133696.368	1053679.18	457608.8148	391779.011	950908.5671	729367.168	556225.3134	563093.1529	737733.4691	649226.6699	911947.4086	1049165.531	1014331.234	665972.2323	1102093.493	264481.0742	542425.7389
0	0	539324.9334	204739.4563	870180.5013	0	1542867.516	127258.7017	0	0	0	0	0	0	0	503700.2615	73000.851	745811.1934	0	251867.6149	0	0
119696.9404	51948.32211	222943.7142	418003.7948	617877.4492	320165.6386	716635.6709	310183.9151	317017.7476	290584.8106	348527.7442	227362.747	159284.9304	286596.4442	510923.8649	614547.591	428240.6335	529307.0459	511243.8236	614418.5827	249914.899	210314.2093
0	0	725939.8284	0	5739323.199	1175797.814	2176461.166	755973.1381	0	0	0	0	0	0	894308.8734	0	0	984235.17	617630.4702	848440.9793	0	2130661.957
2778267.748	2811363.134	5169174.967	5921733.114	4492822.64	4008077.453	5433915.59	5725802.111	6171154.232	2410822.649	4055756.331	2275059.641	3009313.063	3989044.151	3966606.105	5584150.247	3175441.243	1432486.089	3311701.691	3568971.709	2576821.159	3698351.518
40929918.95	23369247.13	42911278.69	30653359.26	41857186.82	28278981	42681978.51	38532090.37	40992722.33	44940227.7	37055259.93	34515504.18	37031789.45	38198681.05	34463164.17	78782288.53	77370943.81	48467618.13	76411161.23	37322826.67	35550466.39	42133303.23
5149010.835	11136732.71	4492557.569	8004777.651	8240388.725	7598341.359	10147682.18	8514041.257	6026373.294	8283886.243	3699581.602	6334585.639	5521686.145	5712801.933	7091370.866	6921454.007	5316254.866	3202616.847	10601936.43	6313377.118	3934384.719	5250355.121
1422747.172	1017031.227	1985422.555	670140.0308	2723590.407	1805532.263	3335658.285	1778639.2	1470740.427	413844.8835	82242.36263	277357.1646	873160.5901	1061202.205	1298502.869	2105737.898	276228.5562	1178237.763	1961456.846	1203527.006	1500429.564	1711539.911
440511.7821	0	0	0	1036933.396	0	934002.2159	468034.1834	1047339.929	285377.8628	968663.5455	438900.0882	353534.0837	386651.1641	851218.8566	286987.5677	587667.6771	710254.9921	395193.6342	726319.0442	0	186971.6936
0	0	0	0	152016.4862	358613.4851	0	0	306323.42	1497364.6	274970.4665	335089.6891	176783.4134	108763.6585	150198.9456	224426.3827	126075.1541	177095.4571	266390.2318	0	277326.4982	0
2608338.041	3541083.601	1544848.467	1122247.87	2320458.879	3199243.793	3862013.728	3288010.515	1451466.266	2976988.842	2850326.158	2068138.521	1161301.422	1461693.438	0	1714296.126	2258027.688	2606943.048	3843038.157	2676730.189	2297613.638	2648226.176
414684776.6	308451687	611234.853	258088747.2	303151379	756766400.9	405876715.9	579734210	528331828.2	451192807	426670584.1	285363937	693088871.4	643392076.7	597235798.9	784819612.1	550609208.6	872414233	586850740.5	478043546.1	336057474	638214371.4
5030924.623	3001993.697	4547443.02	4569193.678	14007415.38	10122251.42	14379079.41	5617383.148	3476349.851	5395672.471	4198199.92	5953012.028	7475517.327	8244856.545	1781522.094	5755577.693	4752241.892	3433975.332	4546965.786	4106716.718	5233652.487	3612783.156
2440731.363	1514671.79	0	2023651.623	2780093.358	2459675.975	1771488.832	1731642.655	1225768.91	1392148.545	1578951.123	131226.1067	0	70729.68853	1925216.729	0	1756567.575	2023119.703	1569117.302	91359.6993	1491753.779	1207653.861
0	3724.850078	28137.19707	56686.92661	150332.4502	53812.69618	133434.6717	55751.48464	38182.35819	18685.0581	26143.45305	44726.99072	29548.48511	59676.21513	33250.09111	115486.4914	91564.42627	105143.5161	57050.84744	735091.5257	32124.39096	37110.06046
427654.758	430626.1011	0	94550.18955	1659967.129	408562.7354	519558.3606	99914.46344	0	95419.18246	0	116226.6369	108530.0483	462336.671	258841.3963	841841.2451	301787.943	410944.2094	104650.6813	602552.6255	105510.4007	758685.7924
0	0	0	0	415297.6867	0	0	0	0	0	293646.9425	0	0	0	0	456107.5687	821463.272	264995.2549	362303.3106	1288267.555	164790.5473	0
32885645.69	37253306.55	62074077.9	82859294.51	121012780.7	5991208.729	98372653.42	79837203.23	74554154.55	73183747.24	84736242.45	82266642.37	63153881.09	61917727.84	20362308.08	16820571.4	17065738.78	94588748	79031421.45	100249643.4	76012750.68	69892951.27
66810.13957	35545.46377	0	60330.81372	99332.00029	0	55993.76783	99347.54297	0	0	0	0	0	62307.41069	57806.36411	122021.9492	0	51638.73713	152042.6981	114268.1312	0	0
25566571.64	25901574.68	49971593.59	25570412.52	21051789.55	25099032.42	25029991.37	39977243.28	49733442.22	35944778	31170634.94	23995953.49	21126557.74	16890079.63	50266486.73	30333721.25	33759435.54	20398193.14	15036665.75	12505109.94	12477455.88	22196192.89
93507026.12	101212411.5	64898923.74	197697322	283828228	152679588.2	298943075.5	140300286.5	78014931.49	114198575.5	126181753.7	37727038.8	149240417.7	97813271.22	44045244.08	106593506.1	116883527.3	332278487.7	103528502.1	197776982.4	170016300.2	76411390.99
128561.1145	10888.85695	6362.589723	212478.0139	0	0	0	80402.33922	0	728311.1177	0	0	14399.05579	0	325195.8696	102711.05	773081.1332	27794.28934	364531.3019	101626.8889	0	18336.35608
0	0	7941740.382	10300616.8	17028023.23	2968941.932	20969231.37	12128007.24	11238211.17	0	0	3839842.483	5052621.358	0	21423182.1	11254702.17	0	0	16586187.64	8669999.871	4548149.773	10544867.36
0	0	26839.23289	7292.952972	11947.34155	50584.40018	65210.38482	38467.16088	14500.87552	11605.04628	28694.82761	0	1456.823836	42120.16418	46521.58335	43484.17719	58128.49988	163463.8832	57197.06001	32989.23742	0	16841.78738
1007481.637	0	514430.5842	808531.5109	912008.5788	820160.1347	1891603.695	862144.6838	1068255.191	1433165.751	1284705.955	1177386.596	0	501129.6042	634921.8535	365478.1287	676244.7114	274956.6731	1138278.919	875228.6462	741501.7142	1159698.372
4112853.593	3362154.006	5155077.414	3695531.197	4503800.098	3692652.758	6178403.337	7979736.028	5622880.108	3401075.096	3492262.92	5240646.211	4742247.364	5288949.4	4408097.325	9050360.424	8889483.655	4681210.091	3457218.548	6703500.162	1271349.344	1384365.4
426465.4376	431836.339	0	94373.75264	1658197.323	408033.5366	520099.4037	99741.95578	0	95021.27035	0	116034.2469	108321.5778	461816.1165	259177.6818	841033.1345	301076.0533	410088.95	104842.8482	603934.6826	105297.6174	759572.1116
0	0	4950220.142	3367589.977	4922679.04	2330884.668	6398679.277	2282947.355	13485516.35	4217484.07	4867231.247	0	6807626.564	7651147.956	14492525.62	9999609.277	11242671.98	14946486.25	7484157.872	11431896.94	1177321.717	2004623.322
123835.5745	136346.5861	404447.8542	1051950.89	1455353.196	484042.4586	697100.5408	537042.1466	531907.9405	0	353601.5451	0	264253.0092	0	570799.1822	601160.7358	985140.5257	279545.3585	681832.3547	508870.1861	192567.9696	0
0	0	30598.24401	0	466220.3374	93079.04055	118682.6183	0	0	0	100160.2855	31062.20939	24255.89951	69273.24028	0	47042.20682	0	125409.1639	10354.07154	145750.0902	56155.92396	0
176350.0308	664988.3961	1080396.327	764215.5606	448069.9333	347812.9696	983640.6576	386742.1326	686629.8536	926098.6025	0	0	0	0	1063985.12	1647976.08	1331564.161	399884.261	1134691.577	814479.9297	169498.6469	1686622.455
152462556.7	105277250.4	197197745.4	330248178	325541439.3	127469362.1	278060536.8	186743076.7	180404649.3	206328148.1	222714164.4	121428874.4	112596890.8	162969347	150315888.8	128117544.3	140804092.3	234685354.4	114760559.6	220317179	282669185.6	171083545.7
13300463.42	5633728.239	10326448.76	8833820.27	15859604.05	10420364.07	18252754.22	4286101.795	1983598.78	12662114.8	9517744.292	8414768.906	8802101.244	4888622.766	5833437.726	9807527.241	11953518.91	5423645.757	11751453.95	4770886.034	6218368.095	8863614.708
1868493.224	3783385.161	3925367.706	4760744.765	6192479.987	4465901.343	5768169.107	4531504.056	3989497.097	3800302.874	3470201.829	3102826.315	4463666.135	5022136.214	3137372.537	7020340.44	16310248.55	5494024.47	6118536.597	4073684.654	3233582.859	3162393.173
166956.6166	0	751939.7357	614426.9288	144719.7944	890768.0198	737242.5206	564441.8032	1404898.258	478926.3049	567437.8211	0	1038558.544	739171.0162	636173.6906	938827.4195	1166544.15	537698.8079	793906.0223	1623135.716	238574.2659	200041.9674
171028385.4	179960826.2	7894859.535	640281682.1	1375905550	370632606.8	522144363.2	156551292.2	46979513.01	213069208.5	120072497.1	219239608.8	483319254	361651764.9	128202563.9	275754707.6	2494288.877	542620432.7	277710904.1	273067680.1	292284006.7	304906879.6
1006145.038	0	513079.3031	807629.4673	914798.536	823131.0015	1887596.348	861180.1322	163614.9576	1429571.303	1281760.812	1174604.937	621771.6721	500571.031	634204.5992	365061.5086	677603.021	275297.1452	1135857.375	873403.7627	743374.8284	1161082.394
119437.495	0	0	355868.6611	280180.3278	810579.1397	1724663.901	313478.0032	0	399126.5944	485260.5144	0	239815.5089	205362.3773	0	0	0	1540571.296	840764.8018	778621.7501	0	0
40300682.33	41864902.2	2030679.194	2786175.514	4277854.779	3502764.588	3264715.581	2532604.638	2282020.87	1974075.666	2314634.165	34259704.26	36356830.46	35904842.28	2686786.946	3809655.775	3729887.844	3494942.42	2643430.764	2439390.965	20646282.77	33330521.74
0	0	0	7833498.899	0	0	0	0	0	0	0	6081518.264	6759333.727	7526059.552	0	8226136.232	8003982.905	0	0	5684413.725	11541767.67	0
1599432.515	497775.4837	0	904115.3239	0	1909212.151	1821101.984	857315.9697	2088716.178	866312.106	677034.6086	2378531.099	708009.6699	518051.4613	1594191.408	1626037.806	3341864.286	1514076.943	1699467.585	0	950921.7094	516274.9534
1545298.188	2251258.151	4177173.386	1642580.462	2575740.708	1932987.16	3257754.458	2090885.618	1137747.842	1400245.42	1460203.212	3688475.146	1483096.073	2654577.28	2308174.665	2642120.153	2059057.496	3468200.972	1827952.953	3200959.134	3293026.832	2672830.885
0	0	1719132.879	5090164.901	1440591.007	1922019.71	2872127.67	2031964.141	745563.7773	438066.4217	2809083.1	2948267.302	923378.0585	2401722.257	2508489.346	2302465.473	2005546.702	1733300.37	1978231.992	2371337.1	1941419.908	4245141.925
2317865.99	1778191.319	1859973.577	1728338.278	1796363.977	3447421.587	6199638.007	3705523.394	3472237.663	2896614.118	1982329.926	2428238.182	3717728.318	2048322.364	2836471.364	2473580.633	3345514.18	4240460.73	3619772.078	2911214.001	2163231.306	317641.8862
233909.9799	0	702838.6331	622930.2774	0	768444.7762	366004.5649	784705.4056	766058.5967	1206443.66	662991.7656	403024.196	473187.9351	902388.5978	822542.0901	294182.113	335573.5765	213193.3433	0	601886.8794	452991.1482	430218.9978
0	0	0	54069.52716	33760.39427	31037.78306	24495.35521	50409.13772	0	0	0	0	0	0	0	33757.228	33614.56762	71266.66861	0	63834.20391	0	0
502717.7809	847997.0195	1310944.965	4339974.879	1836632.065	858284.0507	2028009.149	1002436.985	1187732.964	1423081.464	893541.1964	958752.1483	895320.3612	2099895.316	1195960.146	1994359.562	2573940.462	2704958.947	3674298.239	2818644.438	1140656.659	319854.2025
215169.7437	34769.90638	0	0	565603.2906	66168.46718	1871859.411	215559.2015	0	54908.50693	129856.5043	0	369629.1956	238548.903	123812.6192	528044.7331	263819.2074	74588.35356	222035.1785	189419.8632	392085.5686	0
37716282.2	49682188.84	25898065.3	43297148.97	56313159.77	47121207.36	48688663.92	34397408.75	37241403.62	38470353.68	48370142.46	21720183.17	30588030.81	26175944.82	29006902.12	45327234.08	37257478.2	55758481.08	41213048.03	36728889.16	48905343.1	43739688.14
1029334.034	632081.4347	517539.3039	1281217.556	1216378.173	1338472.366	3080243.212	1672266.988	756670.2246	292264.5135	920648.5614	789821.8175	95071.23467	597547.3805	792930.3896	1079874.624	1036912.681	2206059.076	971394.3048	1363630.144	167860.1872	0
3708660.39	3206301.542	6609253.126	9303271.083	8881196.999	8306626.453	8612835.894	10699803.08	8146009.262	5359629.775	6800745.004	7620762.448	5958991.549	18326389.91	8385480.457	10870317.78	12334226.58	9492973.718	7406606.339	9218769.209	5414890.766	4175051.903
97977.12414	374526.7412	107236.3631	135392.0882	872874.5257	540086.5059	343246.0224	393240.2213	535628.2793	141156.2187	603891.4327	1085374.933	788223.976	1005036.123	340536.7791	799665.8066	681756.1141	804383.4289	601321.3264	700215.5833	350721.7592	366749.0273
7251435.813	13696468.91	4475315.242	4181603.838	3790837.746	7423112.417	6497252.662	4321715.681	4094677.728	5743315.353	8889312.254	2102692.856	4376790.286	3617728.015	2749628.687	4337968.097	5901994.869	6030023.716	4027909.219	7515040.385	5276083.528	4317786.999
184014.3343	1325716.868	425694.6341	0	393437.3646	661876.754	404617.7591	1138634.155	789293.7787	376669.8464	848334.643	505725.0046	2343675.706	330577.8506	845597.6648	246567.4495	417000.9886	1053031.673	237678.541	594907.9003	224746.9093	1345860.918
2183235.694	1506469.774	3123384.94	3240248.186	3807062.791	2440064.284	4559381.81	3416628.233	3835990.74	4137360.933	5823168.266	1271592.535	2880831.76	3627079.928	4692886.277	2872336.062	2210657.986	5698668.54	2549783.394	3824265.142	1220514.359	1607326.048
135840.0047	401579.428	10038.08228	229359.109	1006953.588	245801.0986	1683916.1	103261.0814	489331.0831	82233.97426	643349.0862	449899.1034	159563.3765	452897.6666	341763.4833	669657.6918	316633.9989	588064.3969	451049.4416	751933.3378	301390.4824	404178.7412
1369983.26	2460384.581	950515.3655	2730732.502	5056025.447	3547805.927	4151074.813	2457915.211	1563606.83	386136.9156	3564303.394	2277937.694	2538481.164	2064629.14	883614.1385	5967819.825	2579349.197	4401759.751	1817946.443	2685676.021	2082471.77	702660.0723
563474.093	352728.6591	1672800.424	2104356.981	1339630.833	1843667.199	2473795.16	1398926.417	1509215.752	1193937.359	1494159.506	2606379.732	2025140.722	2344053.471	2297801.565	2194009.439	2412728.298	2251342.964	1788449.084	2211745.924	1236937.075	128089.608
0	0	0	147186.3573	114192.3082	110859.4081	0	245776.9907	156109.1695	122782.2957	0	556886.7529	578575.8948	309427.6075	100854.5484	0	194655.9677	59434.31035	8804.263592	102484.2661	66575.93434	299553.3016
75194.90966	144720.4836	216887.3112	586426.9874	166110.3285	279725.4412	173559.4401	653978.9135	688876.5524	154165.0262	357229.6389	481992.7004	527752.0787	668592.6205	643322.4425	566091.0384	350943.7398	518929.0345	505576.7771	744072.6976	56929.02447	68958.5405
5094127.338	10823950.53	4065252.521	7114677.408	9017898.399	10375001.9	10240245.14	6209325.805	6471964.429	8247244.088	7079538.797	945083.5965	0	2289752.65	3554665.737	3013992.989	0	3913887.555	1712608.508	1554000.2	671216.92	3256590.827
634003.8216	0	0	0	947234.336	121634.284	1167601.325	443145.1556	0	261064.9339	0	0	0	0	0	1084139.663	0	2373325.613	991250.2566	0	0	100633.7347
369994.909	940806.9083	882291.2857	1562665.682	2603087.991	1387344.247	3143110.266	1521212.419	1153234.742	1847237.128	1290863.472	1962894.334	1209926.655	1157611.596	1355212.592	2223688.42	1538034.541	1635661.983	1202673.81	1353110.162	945375.1598	1102893.592
259338.9347	346390.147	613487.7423	698372.6939	608935.0493	562163.7797	603353.258	216176.6862	0	530743.4842	481117.3196	538426.0398	727863.7889	877980.9668	379068.627	518009.245	750136.193	1775306.248	1492591.061	1546111.347	921836.4917	670641.5009
7738791.5	10241804.48	11781615.76	12536398.18	10196846	15404959.2	12624239.42	14897393.66	9805868.006	14709400.94	7537977.073	8770735.432	7486079.197	9326031.36	10851385.62	15150505.85	16232960.49	13151009.45	12376151.64	13459665.74	11160636.16	11810622.63
6303551.593	9003052.199	14479886.41	12725734.8	16866652.7	13779374.11	24705787.71	15987438.83	15399767.68	15537731.61	12150605.14	9680788.056	14770366.14	14946081.32	14514223.7	22905972.52	20163663.65	16003210.57	17291894.8	15068549.06	14967131.69	13906584.19
3901420.838	5165095.57	9054906.151	8871937.278	11557327.23	10923807.61	17236091.17	12753091.71	11156187.03	10364322.79	10292971.98	8453516.973	8015085.445	8637593.121	11256709.18	12280987.08	7784959.283	8978631.179	8027326.409	8836187.388	6270398.779	6962413.341
3943513.906	4425469.759	3872506.474	3818963.302	4969604.726	1962712.117	3321085.127	4986499.847	3396215.88	7444556.527	6071221.531	3201941.847	1802309.565	2884647.321	2511214.828	10398287.4	9017461.336	7588427.06	9450283.56	8713115.371	4584401.867	4221602.348
42292428.05	59539301.11	49272348.68	41822337.28	73843471.25	46314115.32	72654492.07	53777190.61	39015980.94	58589616.23	59052166.67	50154061.61	47102229.94	39908251.9	40781192.05	83185751.67	61357606.02	94259043.65	50909995.17	98747634.6	77643297.01	42364052.07
125375304.4	204252527.6	130300106.6	109280765.5	156177833.4	148036002.5	91070174.41	120145622.2	186713612.7	178010090.1	118431118.6	119068857	102053965.1	104109305.3	187872746.6	165956169.7	181556198.4	229353994.7	168133939.6	131057268.9	219645954.9	192585732.6
293048.457	178105.0337	240976.5127	540013.6697	858530.5785	451736.8054	708016.6338	665531.3826	448191.2592	329446.7962	449765.0875	554085.0463	54285.53968	317389.7035	608664.9469	889477.2919	525653.355	938859.7734	545906.9902	610549.1147	123688.3431	290729.3716
0	29748.63378	0	35777.51863	0	0	144136.6922	43070.9421	30922582.99	0	0	0	0	0	0	49580.31168	75552.16129	113329.3114	169200.8967	124859.8291	0	0
754543.721	1365546.491	1531561.703	2270010.584	1152753.627	1811980.062	3109930.991	1006715.521	1167154.1	1969756.192	1050685.43	699462.7425	1371017.159	1314055.627	1531839.232	2357548.003	796791.8249	2239132.103	1523645.923	1064245.944	2269705.825	582934.9761
734248.0076	499205.1665	145020.0617	736384.2983	1239301.246	369772.0472	422693.5255	322396.6172	196663.116	317787.9252	192523.0147	217991.7189	772455.5474	494542.6709	321869.6345	849421.4091	566464.2628	882397.4403	400486.7307	1002091.696	297261.4423	104830.0661
31410843.61	30865601.14	21487626.65	34466528.62	65821625.17	38897933.23	58637652.64	34007819.93	23395525.2	21400840.73	30826539.3	32096483.65	28415745.69	23945162.89	24709783.73	36674391.39	26709825.28	45270536.9	29161211.44	37235371.89	35914762.12	28521708.7
3386133.881	4762819.53	6113218.772	8689123.163	11914084.79	8901102.802	11795493.7	8262574.434	9887221.12	6897222.331	8515984.552	8329813.856	8225457.708	7673461.053	9751682.024	13457340.65	9794810.376	12722923.33	12590498.5	8744672.824	4670713.782	7425518.124
776311.5176	1138997.994	2221446.315	1393126.863	235666.8201	1548250.991	1474744.916	1540300.154	2002214.64	2114724.498	2141114.973	1035799.437	1907792.919	1303819.439	2702630.733	1244740.338	2775254.99	2519019.442	2330183.533	2348997.124	3186804.21	4940625.36
0	0	0	71337.21136	261094.9186	113650.2488	0	0	0	0	0	17612.09011	87599.9531	220616.2063	28232.57464	0	54619.13592	64495.33726	0	213114.7372	82486.06777	44185.79553
0	400366.9642	553923.2044	703437.6892	3366378.929	376833.2018	1185849.909	643637.7216	44005.23289	0	42688.57636	56405.34044	43221.68742	372952.5723	322660.601	1249646.563	466475.2468	158967.4959	78983.82347	66515.23387	236763.1916	243435.7344
732260.4204	997431.0444	0	713164.1277	2543473.014	0	1572726.789	0	0	1143248.654	1084623.146	1032119.717	1441192.218	1519838.672	1036180.694	630773.5715	635533.8689	2176917.93	809968.9497	1083899.768	203802.9384	1203304.056
159556.0011	517519.072	1587738.279	1946306.838	2562195.794	1971658.475	2845079.399	1733644.32	742931.7081	770442.8931	916384.1139	1667472.569	1191196.28	1776014.623	1863488.983	2178317.403	1263752.7	1398190.884	2845092.421	2233444.95	530469.4908	785580.6079
545187.0462	360697.6656	675572.4169	1084800.958	2031560.487	1612671.331	1951753.484	1132369.742	1012670.805	1395384.708	775911.1454	1671428.08	897541.5111	998451.4734	1468384.514	1283414.023	1403715.962	1149803.789	375561.7736	857227.2234	1725814.698	1294422.706
26362.00121	0	0	164700.5339	0	82914.24477	0	0	94787.31846	56980.91973	48683.52441	95971.72321	77960.95766	14114.46353	92347.83936	108696.8193	174913.0611	197486.6115	37088.09872	109904.6635	61185.01094	27507.62619
2953205.942	2359058.09	3667946.245	7190819.163	7610044.637	7185926.055	22490990.9	6654354.174	5809734.768	4043141.204	4129963.032	3906278.585	4297767.058	5115079.578	5900369.591	6773406.029	6432434.164	9795501.363	6302949.492	6992022.736	2440017.019	2749381.964
0	0	0	0	0	0	0	29825.22479	36572.55554	0	0	0	0	0	97529.27097	103883.7498	144752.986	83669.12232	104455.2687	0	0	0
0	0	1512365.709	1119744.081	3862100.823	1865000.747	6648372.871	2066292.267	3196024.83	2039889.335	3039827.343	2131117.732	2101345.051	2322273.896	4092929.304	3169874.695	2682521.397	3287629.23	1939769.277	3065394.468	3019140.398	2267913.169
501963.2653	564926.8768	630214.3802	91334.56907	189064.7683	359922.0961	0	491010.5326	71542.3324	389993.6294	622238.5927	389420.0833	0	392840.6298	583277.4767	402221.3733	957673.0961	833374.2121	412340.9269	1123973.895	279236.5919	240463.1826
164146.5421	0	54059.97916	151212.3949	1026486.873	0	337987.177	48578.40451	41557.77118	78668.24679	0	0	0	0	0	114562.7938	110261.959	0	248752.597	72094.02769	0	40943.41675
1143544.097	1860362.175	1876326.334	2464736.071	4374471.871	2856030.583	8685542.273	3570818.962	3965792.771	1705116.068	2336826.624	3079106.178	2541846.603	4379633.759	2474828.042	4642871.477	3994116.069	3163720.935	3269741.919	3705754.028	2311864.647	1526363.627
1590809.024	2029670.931	2130494.964	2473549.577	3558077.329	3226900.244	3981855.267	3319279.422	3690678.012	3062427.998	3095851.337	317651.4949	1602458.692	2287148.663	4397208.065	5603774.346	3398863.246	6450512.823	3369295.218	4355762.316	1649826.773	1505675.881
0	0	23025.46857	90603.37276	1005974.764	57310.9007	41473.40739	9813.600571	61742.81259	79888.95967	0	0	7722.272685	31514.79947	0	25907.51588	0	47406.72631	10651.62531	35556.02231	69127.45076	0
1008777.682	1825049.946	1820375.295	1842169.724	1811757.944	1411256.311	1550467.196	1857675.973	2057393.205	1990118.995	1947606.673	2389991.229	1050183.934	1448373.346	1178863.582	782810.1364	1074075.865	1312850.972	1105855.559	2029736.603	671994.1236	1361852.991
0	15462.47492	0	0	307283.9891	0	134594.0935	106566.1943	86791.86504	79716.97818	50880.10707	0	342230.0667	0	0	245482.4292	303326.1898	96085.69006	42730.56972	103301.0873	206981.0577	44512.64618
897688.724	672244.5259	887597.4894	2996880.413	3642910.114	947472.6375	2353966.311	1735574.35	308033.0347	802472.1244	1339337.375	322082.4389	342230.0667	1582657.396	726717.3951	3945683.522	1583942.638	4787993.518	1198910.939	1013937.572	1608350.668	858058.6347
100252.4592	95121.86088	116637.1953	890972.3213	4433870.929	131892.0651	742203.7235	230134.6352	119688.7152	0	90167.24289	55502.19862	49945.53698	34487.16118	107929.1002	180739.9225	272168.4245	715299.1952	453608.2129	97264.64197	141667.0328	124080.9142
55381.9811	50556.99852	651134.121	602971.2663	1025526.678	370640.8325	845889.98	621842.5416	367763.7633	303503.4818	907076.1468	297332.5644	543576.2246	478361.3131	605045.0446	728094.3642	366729.0106	776276.9025	400029.5789	808522.858	109157.8433	0
0	0	103117.0812	111836.9497	126932.3325	622841.1574	152665.2517	37845.32534	364972.3952	149035.5011	319270.9556	581716.3736	460625.8576	63879.4038	438072.5909	505340.9378	487444.6392	411423.8049	286298.6821	0	59866.53423	92222.24527
42030935.68	19547805.76	69921430.14	47010445.46	58784376.65	84562582.34	93721116.84	105708072.3	63891617.15	94607298.5	49530949.72	44111724.71	42674088.89	42130463.78	58865587.5	80453832.88	98096875.97	67186524.78	102705031.9	77125285.12	66391149.3	73357044.38
5261286.203	2614556.216	4327472.55	5883397.945	8891610.525	6742923.855	3634697.95	5817222.36	9428859.357	8460291.321	5284498.424	5644111.677	2548166.141	6456169.528	5635745.182	5541031.302	8970040.075	7343930.186	5625587.519	7457706.894	6979639.17	5559167.152
2140657.534	3645088.247	3094586.196	5154502.684	4003612.304	4922299.597	8706410.699	6071379.464	5672225.009	5956593.675	2370116.395	2959750.372	2488772.6	1930140.313	7194591.179	4775253.527	3453672.021	4430228.429	4318681.656	3251596.233	2033835.015	3983234.28
279935.7332	1178212.52	2204567.503	4277647.479	5948767.49	2159373.346	2278377.276	2129532.077	2446951.942	1376920.111	1844538.42	1663137.516	2022697.311	5179908.316	2452393.155	2777193.542	757218.6874	5064760.479	5645058.916	2475923.128	4174690.071	1004672.296
0	0	358982.1842	0	0	0	932981.5188	12527.80689	0	0	0	476700.1258	447772.0155	48343.64782	198604.7043	0	1006450.29	423417.5408	162192.2486	0	0	386708.7158
3282990.329	1160584.447	680535.0699	742814.0635	1873890.253	1860879.755	4225825.414	2139491.159	857876.9675	1723363.971	984480.0844	718649.6794	1140491.239	429458.8889	1571544.97	1469861.157	2189688.065	3133121.799	1121448.455	903593.7943	1715374.136	1893228.377
1247839.939	80362.24843	1282578.057	374716.2087	1687953.47	371553.6607	65939.80194	1253665.848	1836854.519	1381348.942	2915356.718	429829.0264	3615113.222	1338864.537	3712689.017	1455363.037	2796576.315	3126763.826	1970174.862	1572209.157	1748940.351	1501817.511
104697.0856	139858.4992	0	77079.99438	684936.2396	416961.1058	1675287.383	140944.6118	20596.33958	2714.652954	354569.5212	177733.8599	44099.16734	259542.1944	0	588897.0805	188392.6874	337795.9588	0	114481.247	216787.7215	0
116331.4844	78739.78131	379355.7819	477674.4098	554865.6764	125698.7161	951633.7391	187058.1786	112712.0644	126269.7083	112470.2167	675528.0237	544918.0681	313661.7341	775797.6302	987975.4684	598031.6844	351015.6547	344367.0274	555622.2458	233626.0891	157494.9558
694686.8439	3843272.104	0	432237.0276	1283090.742	834498.0834	899815.7472	1107404.468	502843.1011	1032677.435	0	338814.528	195049.3627	0	0	618297.3998	210304.4436	0	0	300104.3647	725431.39	433687.6758
1430442.934	1894213.077	1601346.804	2003065.978	6619462.726	2217834.551	2689309.094	1955378.089	1602823.015	3154146.201	1961970.294	890030.6628	1971723.589	2809058.528	2730390.43	4150348.002	1229979.923	3936570.757	719950.0873	3533971.693	4823970.552	1928269.671
0	0	0	0	104203.369	0	0	12106.16231	19206.37226	0	16955.45124	29114.95352	0	6561.412615	0	72225.40563	4778.570062	0	10796.92581	8142.160389	0	0
0	0	33913.85615	0	0	48867.22646	102881.2602	0	115733.6922	52473.23574	0	0	0	0	0	0	0	0	46997.73363	0	0	123405.7027
2644589.094	2054845.618	4698789.722	4648686.764	6986513.668	6104682.635	14015062.41	5688118.27	4838822.551	4453943.552	6038984.085	4996530.899	5393752.163	4451408.682	7015976.606	8590155.885	8042234.218	8922379.896	5611909.475	6098384.524	7019643.169	2933069.704
3215356.172	1372784.517	2860495.974	5031742.55	7358821.196	3662027.858	6941728.61	4306011.932	5354612.233	3123013.578	4736239.87	4287369.305	4858643.449	6640091.43	3776324.775	5623491.302	3077084.456	4464391.871	4968111.126	5372008.75	3428956.782	4769418.496
7549733.034	3618611.504	1422032.896	1757755.76	2033731.08	3223072.25	3032255.958	3033777.305	3180165.669	3656054.216	1733479.443	3561295.206	3371423.431	2190595.095	4143529.256	2860302.905	1895423.73	2510935.968	2261596.61	4798004.745	3831438.406	3442569.245
0	0	0	141024.0671	239891.5134	0	198336.224	69553.54871	0	0	94791.56317	0	0	0	0	0	0	0	54127.23276	0	51154.23588	369212.4135
2615755.217	3281485.657	2733806.93	3564093.372	1863954.326	4120620.327	7251609.404	4998734.665	5451830.405	9873881.339	5128755.57	2727411.273	4775165.026	2805904.494	2598568.006	3703184.715	2436281.227	4095318.338	2518331.545	7236940.275	2058812.473	5010030.075
2195276.045	859462.7506	1903241.322	1372227.329	1141035.075	1509547.678	6974256.749	1492439.971	2928557.626	345141.4159	2085707.298	948388.353	1385306.616	951688.112	3843791.533	8097822.781	6553750.329	2854080.426	1060651.74	6787973.671	2554569.468	405855.5757
0	0	145286.5543	0	0	0	0	0	0	153910.8633	151078.2184	139316.3324	161999.7055	627262.3855	137081.9532	0	311234.439	0	237986.5011	0	0	0
0	0	0	337529.4297	265664.3371	0	463701.1887	0	0	0	0	70418.36162	0	0	423854.8992	773709.1815	776952.9407	0	644860.6886	790580.3963	0	145832.9379
463048.1378	988421.0547	1046746.614	741956.9557	1439105.453	1930836.609	3133189.971	2227019.017	1088782.668	1606416.871	2345086.731	1104034.076	1345902.464	2043501.201	4966760.407	1570087.223	1477686.069	2203769.375	887900.2284	1604984.894	1288447.869	1185807.667
61910432.65	78786092.7	135046889.8	101133128.4	104078221.7	110983064.8	111887818.7	137870069.9	113812060.6	120439338.8	182217162.1	136438837.8	172865141.5	102339549.2	113559739.4	104156146.4	141473363.6	191461002	120021071.4	207574296.6	156629836.4	109476626.4
414871.2072	1175180.68	781856.8965	662495.0951	3370442.767	1279368.504	2548866.874	1087523.499	1537350.424	1385905.251	802578.3233	2971388.833	1831165.46	1723856.881	600156.9447	3176842.228	2629500.754	2884438.319	1746496.803	1739502.745	1515425.674	1771137.973
57608780.14	35970805.1	61667484.81	55830545.88	1071209.778	70231813.94	63574453.28	53895892.23	26949194.95	39274222.03	27869212.22	41401704.44	43349942.95	75866380.83	70245994.15	53079645.14	48045269.4	33691708.34	81891898.99	29285508.8	64938297.61	30145268.24
2110959.705	3092569.88	8518698.164	5629902.383	2465761.402	3438743.111	4475988.42	10648276.45	8924176.557	3111360.556	7589743.2	6639830.432	10940174.16	6134455.757	8791881.306	9092160.075	5761067.653	3538375.054	5839092.06	3354220.359	1234164.533	2943883.543
8065071.734	9757595.333	19593583.41	14998512.1	20699301.5	25048194.72	32786672.16	28226020.93	29161315.56	18210886.35	23053689.81	9505909.796	15362224.59	20393000.62	18842329.91	20100882.46	13770049.99	29478947.42	12183444.66	21194499.8	13101691.5	12075143.59
0	491544.6575	1234917.179	786354.2939	0	868037.8286	0	0	929658.9745	580099.2964	0	0	3155358.274	0	0	483496.1743	0	591289.5655	521531.7178	256618.0397	639608.5119	938907.3771
0	942006.1148	956431.3499	1668961.229	2099499.488	2341288.005	2082956.285	949446.9043	525755.8354	843383.4993	1277221.708	2234467.085	1401875.639	2087261.08	1799500.177	1505847.956	1446919.303	2739146.816	1429740.075	1626595.893	2422030.79	2618832.57
653638.6978	1379895.096	1895885.13	2306584.033	2821127.409	0	996073.4164	1427191.123	0	1288705.783	0	2467334.138	1278354.611	1919081.353	2123616.116	2535774.45	2337111.466	1240540.491	598094.9999	1265955.484	1043653.783	1336280.898
47752.62445	10516.82881	0	78937.83141	152245.6071	54985.08405	118884.3374	98434.22362	73328.12304	0	162946.8292	33315.01204	90051.60649	109764.0934	129592.6888	67655.5985	46455.70827	124897.1458	10501.20571	210627.6154	72931.86182	0
0	79944.17963	0	6476648.957	0	0	233636.3094	0	0	0	0	106145.8332	120741.5344	0	0	734568.3125	164161.6609	492887.188	214704.8335	0	602297.0044	0
130762.4182	213592.7222	749219.2077	939024.5108	1233052.2	1022254.652	1236457.738	576301.3865	255340.0313	270281.9648	311216.5742	347043.9121	278199.2389	586952.5687	863416.5603	323820.8068	412458.6931	724199.4287	345167.8553	185869.3537	468296.6483	615545.758
129701.234	78949.4252	0	0	94254.1013	75735.93598	0	0	62773.01979	123419.1701	59538.17185	0	0	0	69176.7316	160404.5103	126272.2042	252129.2436	0	128079.4295	45248.48253	109767.9363
0	0	9368411.724	8143739.145	12678429.64	11694094.94	12166347.53	22307546.18	14651348.43	17071096.93	8541568.887	9257486.85	14693446.9	10636448.03	8298466.653	17160002.81	18399996.65	12270840.9	22431756.44	9440626.866	17599782.97	9452957.385
3468670.213	4826648.077	10747086.82	4287844.029	41484482.15	6835446.602	16087016.06	5357654.203	2455291.384	3828061.499	13760078.86	1497615.603	5620477.637	10257316.62	4277359.842	7183562.143	7116517.416	13207035.11	8423463.983	7318968.11	38637354.98	13765891
25965003.43	28562902.29	33145957.2	25249011.06	21626829.42	9219146.798	41057856.86	38253276.24	32329967.43	31734758.15	23105681.9	17509409.96	17793336.12	16657459.99	22662163.54	28356731.54	46910943.27	21157950.22	33675237.81	25573737.22	29858919.97	24094847.46
0	264822.877	0	631113.5125	311354.6067	2093131.357	1093679.626	0	328260.1831	554884.4675	159598.6603	1042120.214	0	95526.372	1293104.986	891251.2671	840729.0346	1696323.19	2535339.575	928691.4332	1068618.919	0
1450505.565	0	2365155.998	7801792.432	4138726.638	4623689.894	0	1758699.639	0	966666.7521	2719166.373	5765359.937	11044110.89	5450118.34	0	9252477.674	7352397.04	14353821.8	4023444.74	4572540.944	5581270.677	0
8798654.813	7236987.132	12182811.63	18128881.19	20381456.05	23855521.29	32355142.3	19403476.82	22792440.16	14611035.41	17682551.33	17818312.32	16831774.42	19437854.66	16983131.25	12120303.07	17948131.72	20466349.63	13675535.75	22404604.56	10726051.8	17678831.82
1625912.209	6414324.313	9839937.711	14354796.46	13768077.38	9650858.306	19312707.26	15905667.79	14108803.57	6006977.199	8230343.969	10298095.04	9318514.523	12940611.05	12741339.88	11027774.76	18156056.8	21016388.67	14628606.67	22290117.06	7682352.739	9334877.769
606649.9003	653870.7807	912993.171	1183256.611	3267609.314	955954.7217	3841357.11	1257876.496	1910581.96	1126397.113	1028796.449	715122.072	993343.6245	1362664.483	1452653.509	2020846.235	1684333.815	2178983.987	1451805.499	2051259.054	581022.5201	959565.7794
4774923.246	8013754.546	8624729.27	5937144.432	6333750.374	8361571.884	9738317.269	7745005.8	9456081.822	11215951.7	7431307.441	10671486.88	7878528.552	3671275.268	5621465.46	5897544.774	7964159.347	10214808.7	4601263.404	4876598.323	4420371.222	10137081.01
296295.4896	133255.2106	113753.2365	267184.8661	130409.3026	206013.7771	105136.0779	338727.8523	494596.7487	327288.8347	632984.273	363070.8107	508934.1811	734543.4665	551491.5597	395430.7601	358460.8608	869173.7395	396769.9331	346336.1973	1459805.196	444098.0586
0	0	0	0	0	14981.99866	39904.74585	30604.3535	0	28740.14711	0	0	0	0	0	32790.87239	15946.66037	87201.1613	0	30709.7264	0	0
2189515.63	1731255.479	2409559.889	2522534.517	6236627.634	2056642.937	5754188.54	3662920.84	6033271.373	6801986.798	2161178.811	3024477.185	2032375.414	786203.6367	3326856	3212008.577	5278058.757	3534461.027	4901755.82	5644281.546	0	3707424.624
0	520491.531	538636.1018	1230772.419	1062968.933	2169309.75	1234220.047	1853340.084	1988053.194	1160888.049	2553858.476	1681807.454	1279933.636	1934566.057	1378340.161	775891.4393	634943.2797	1814203.46	1755819.035	910821.0382	1454589.617	1000074.888
950268.5618	1037333.326	738077.156	491734.914	434918.8997	1148858.962	1190491.855	1086366.926	1735505.004	1679843.19	1671766.163	984616.1146	355412.9842	681598.7629	1241792.301	2077421.028	519920.3979	519476.9355	1729478.863	2405916.718	545116.2311	619495.9651
180943.028	470020.5189	0	99930.67674	0	286117.293	860272.3374	170109.965	241858.5624	204539.5576	184608.6755	459265.2172	0	128000.1731	0	231255.4981	94901.37251	0	337787.4094	0	0	0
520253.7687	871139.3285	478073.1677	1112276.522	1179359.255	541961.5053	1191666.849	690311.5412	1037769.781	640776.757	551180.4114	1216303.877	668916.4221	1277420.464	1601195.36	1220767.236	2033036.736	2406972.986	1618277.399	1336713.552	1493426.85	1129080.453
178624.12	226839.5667	152993.154	292399.959	315923.2621	1438297.101	643724.3302	639408.7878	173784.5354	0	25477.77876	917079.4105	634512.3915	814484.1255	602151.8994	375777.7284	811593.7603	0	748070.2212	356143.1261	0	364162.6566
745212.5265	160481.1191	3194919.044	963454.1682	551141.3367	1987046.643	3509030.714	2959438.925	2736653.659	2611794.251	1167755.729	4201761.662	2121988.898	1062480.287	3186462.638	2806299.242	3519948.239	1326799.041	3915862.351	2622170.8	2203874.981	1076028.895
0	0	12979.95124	60182.59571	178013.7329	45757.3356	109378.9508	331584.2648	121610.1631	21872.96277	39880.95934	72891.48452	40766.313	238207.1249	57957.56085	141883.542	60889.28383	104633.6849	30297.09878	83307.61544	53671.32799	0
254640.1447	488115.0916	407675.7992	420037.7616	659217.1009	0	245967.7877	190600.1934	141584.7257	19299968.96	0	332428.8427	0	0	0	11512775.5	0	0	385757.0365	16405688.14	0	0
199497.0783	0	94952.19348	1461989.537	417940.5795	1325340.411	1538313.132	989493.2156	1632206.217	1178720.724	1097319.057	1576790.577	2918951.24	1356716.943	1247375.336	902188.7844	243424.1611	1364604.964	1177119.543	976185.0714	1906509.474	781020.2957
2186267.811	1349879.121	4469526.058	6513561.553	7312306.954	3631359.885	8074230.559	3938211.749	4464358.012	2214509.111	1669832.488	2211912.701	5604724.322	2964202.176	2966454.795	6357505.85	12031444.37	7117090.338	3865202.412	11106250.58	3379486.242	4114377.657
8890994.587	7379592.826	3994285.113	4131687.785	6641478.609	9622605.547	8979365.814	7477425.742	7942819.382	10409493.72	4892759.889	5430478.713	5819898.496	6421670.392	8594152.313	9651083.182	10273064.19	8565053.414	12166687.09	4742430.796	7898877.494	6531966.553
811656.9951	874573.3519	2306310.978	1369020.261	2372212.302	1151661.347	2683716.381	2680357.415	2134776.05	1531493.525	2153411.79	2133683.672	1736201.683	2989908.261	3090055.899	4342563.355	3219599.508	3505894.166	2345173.574	3150319.973	2108426.379	1587921.955
14108689.06	24812995.96	8428432.154	9248806.068	10513658.14	13480442.44	9775627.635	9449942.327	9629576.459	10378490.09	10369016.52	5886373.001	10025264.43	9533046.106	8731166.43	10956829.32	7646554.19	7108602.494	11159573.95	7252979.593	9070876.697	6115373.484
28939967.78	35473984.89	66803330.17	44366347.5	47027117.39	54978911.77	68514863.62	74902411.34	104490010.6	52810442.38	51986364.8	97641116.67	61814091.54	52365259.08	76137187.86	67028137.58	107782423.1	28899975.03	72636827.29	52254471.4	35379691.66	45077957.56
215926.9434	0	335884.1949	265338.1886	413495.0043	481785.4493	203778.9708	591787.6585	563825.0588	308111.8783	80083.54259	86960.91672	107443.1204	679411.6493	469119.2768	215056.8853	389997.6777	309507.6736	459344.4789	341236.5203	361805.2691	73313.49111
37942419.29	24460041.96	45432015.63	62986458.03	37068302.17	64988710.6	44000648.37	46600689.81	52498986.26	64396700.67	36228121.13	48531637.69	62761142.25	50971007.72	58694949.31	68252767.48	58088887.9	34304962.81	68008595.99	86939206.31	36863492.56	42705754.96
513659.9194	490193.7798	335417.5195	833236.9848	847946.3475	448350.9298	956419.9147	887383.3677	672446.3537	752935.0396	780412.7052	388903.4666	587232.9928	628046.4432	458263.7923	1140170.417	497068.1679	7635454.758	11498725.73	10601024.14	396956.4171	518119.9222
525527.0114	587209.7156	256141.0525	583714.2086	1051280.803	894818.5389	806576.8213	633634.896	347748.8651	255979.2628	543987.8199	596509.8445	497465.755	380176.7861	513288.7685	275325.9075	432093.1144	1245668.845	450526.0281	1783070.295	1085462.149	1025391.491
115875.0918	7098.996475	223640.3901	516551.2685	1137401.812	309448.4461	475141.0032	360492.6661	302729.8561	159236.3795	346629.0354	274917.7671	142057.341	192024.7677	592720.409	900454.4205	300701.042	182753.1167	237008.5134	158537.8387	94019.51414	78918.8921
249241.5139	1276399.556	658591.9835	301617.8284	2130359.77	184272.9384	593545.0892	646647.0254	388108.9201	250300.3494	458178.0856	640477.3985	121405.9565	440151.2336	1597604.954	664873.4888	157134.6083	392709.1699	784531.5987	566403.9201	229562.7345	340076.5889
0	6388.576475	0	0	0	0	17660.08779	0	83071.80871	49495.36992	41420.71466	0	18569.04564	20254.25845	0	98361.59257	80124.87863	39430.52316	132268.6476	87888.21109	6727.091464	0
0	0	17262.73685	48156.77672	35359.10118	23716.00835	83886.54867	27078.52412	0	10829.36792	10817.0724	0	18992.13139	15694.06068	34847.79537	0	27963.47672	24461.23828	124901.786	0	0	22440.43802
36301830.04	36287760.32	62653161.29	55083986.52	61265462.55	74350508.06	81195019.67	64236234.61	61352407.66	58088887.9	58050621.6	38442580.86	32934896.22	49929273.08	67216778.15	79964717.52	57200382.16	58033540.44	53665658.76	45356807.56	38437407.17	60774364.21
2475341.114	2381682.911	1603301.389	2280308.282	4297237.748	2915726.159	3730749.106	1419596.854	1715737.009	1122465.366	1807521.445	2683055.732	1290997.53	2562583.2	1824133.739	699125.399	3713017.602	2770920.386	3556953.097	2431800.369	1938967.261	1499891.966
22915442.43	1445369.225	24187470.22	24186209.42	18238552.64	15938587.28	30229379.68	27070437.15	25711013.1	27541615.74	18233000.3	21595475.86	18260614.64	19094015.09	42687218.57	30272507.74	12951646.69	25400670.56	39584996.26	18204156.2	8611073.33	3104596.796
4810905.252	4663736.166	10849622.84	8463534.28	8765111.067	7443680.429	10964403.98	9476563.797	8561469.38	8791658.754	10278882.23	6419142.473	8687564.657	7956028.499	13256972.21	14381011.63	14664544.69	14511122.76	14438675.6	12783817.14	5796761.767	7065992.198
3580896.215	2086191.636	6805698.887	7622662.213	7832936.624	6618751.201	6754164.211	9543092.504	8384926.523	11805232.92	3850596.138	5254755.517	6095570.883	8768708.314	5840519.439	11345810.38	6393401.571	7834418.653	9695420.598	8680748.455	4044487.267	4566914.029
1317119.206	4950378.795	463200.9625	1913551.99	4189820.821	4283124.152	2577319.803	1568449.62	3064295.498	1140815.402	1595255.256	727322.2781	1098670.418	1608350.668	2355778.434	4710851.602	1294321.023	3742561.079	1952647.282	4882453.597	2416226.85	1437519.035
26578504.2	15302024.48	25653164.25	32132020.54	32651200.68	31002572.37	47372022.16	46489163.95	32494355.8	44135332.43	29826559.07	44345215.04	40057143.73	37804086.64	58387304.68	47192390.75	47358321.42	49717057.93	37339618.56	58123196.87	36563231.21	28857787.58
96360.33027	243601.8304	162743.5029	454297.3103	424041.355	229363.9192	384429.2961	244804.3532	228557.4625	68073.32616	228610.7955	529685.6411	310103.6508	243963.6133	629710.3448	542426.2658	931008.0575	1011330.753	813575.3034	621590.7998	93293.48936	265470.233
6608327.615	0	0	1704817.576	5086842.417	3779080.819	1879371.409	0	1873194.946	1252220.735	0	918250.0806	1283048.324	0	169393.9223	3859081.765	1021123.419	5387290.815	3061545.882	2592113.117	2332279.814	0
0	286100.3293	83955.10688	466390.316	588295.9625	791010.3098	577867.5736	167048.3913	401744.0122	94809.47632	108983.6049	140775.2326	0	368804.2505	460349.82	782085.5692	642217.7122	680021.958	178941.801	827782.1032	211162.7786	165706.6
0	0	354660.4707	0	335308.1614	215840.9622	245064.9276	447833.4225	389063.4012	1165811.818	325317.6294	0	0	0	438605.015	1307767.154	1645356.47	828275.5814	2592237.177	955817.7058	0	367701.7308
0	14801.22553	0	79423.40991	17821.31639	0	26099.64587	145261.7432	0	230602.2863	321830.0347	105114.8757	0	21943.2475	0	566667.0964	141376.4323	225695.7395	526362.3471	402379.7889	96931.68337	0
230420.7279	251502.1104	605723.156	765086.4375	983105.8248	0	126966.9064	1260539.045	1280285.834	0	0	0	1589200.049	1231909.672	0	921992.2947	227234.818	1788402.719	1311225.893	2155956.449	748131.4272	915120.2483
310414.8832	0	0	200366.1076	105277.8885	240385.9392	125425.5397	0	0	0	0	176308.1944	0	0	0	906614.2621	93576.69496	0	54715.44122	475377.5453	0	227030.0736
3153226.987	3777793.096	2368036.716	3902942.162	9672571.7	3278796.386	5666026.21	3228898.534	4689693.422	3099685.308	5542803.005	2790127.705	3531828.958	4245507.423	4364491.67	11688189.04	7954470.216	7414234.35	3502368.894	4990777.94	6516680.428	8420326.089
428303.4038	239984.7366	594907.2589	1366091.414	1613839.417	1108483.854	1605889.29	1913597.046	912312.0292	1240671.064	949657.0628	1118911.89	907108.2548	1136468.026	746668.1725	2008632.032	2088281.539	1823687.693	1339971.661	1904408.728	1117730.8	147907.699
1084002.004	883806.5885	1759754.498	4049547.028	6080569.19	2456806.836	5821741.305	6160610.042	1611796.415	1976264.214	2692693.519	4589998.795	2544934.379	2982613.197	19281592.58	7349914.585	11543832.86	9529846.997	11624520.45	9664218.409	0	0
113620789	197226304.8	169935380	458974701.2	118656345.1	[illegible]	52741112.63	127832500.4	125728401.2	166204691.8	142622725	92569682.52	98541967.24	111877576.6	123942773.5	130741639.7	127730737.7	219663928	110247227.8	177130155.1	43743752.21	182711241.6
0	0	7348242.772	7736952.928	0	5865055.013	4670478.708	0	0	0	0	13128069.97	13714631.76	14295231.32	17186207.75	6052772.142	9757045.111	11093696.85	10058198.89	6455872.137	5649669.686	4920510.478
887768.0091	1305380.705	1260767.098	1998647.006	2662019.832	2634058.339	2129937.981	2756747.037	1756890.404	2159818.874	1549377.87	826048.9578	2258514.316	1450107.074	2371455.786	3300264.293	2532347.285	3422225.131	2526802.296	4694329.901	1269806.394	1690532.562
0	[illegible]	409361.0021	0	1028525.255	650270.0886	668725.7148	236203.225	461357.8559	494172.3086	707731.4237	0	576462.9106	0	1379775.617	2187502.616	2102049.414	585633.2071	430043.017	0	0	0
647353.1804	334679.3229	2182615.714	2783007.831	1482532.16	2437352.582	673563.9772	2441341.869	1477748.087	1655907.897	1402296.289	1970177.666	1476038.866	2380016.717	2721393.56	2845080.752	2551909.403	3643966.998	2205473.465	2650103.602	1471995.492	1411802.728
316948.7831	415684.6074	662157.3029	966774.5043	2783137.941	949541.0361	1257612.212	803742.0365	253932.8213	1488942.225	1038206.871	614667.8096	394935.7114	930174.9461	450847.2987	869325.4976	732372.0276	1725978.218	567644.1395	1404082.247	359791.8359	661497.8508
19211188.5	16871628.56	35919416.2	36444823.91	28777709.64	53371811.42	30292539.5	59622387.13	51576312.06	37778132.62	43451148.59	40429727.04	31402564.62	36805748.75	45835891.83	32861197.59	37169209.03	33000435.17	35723691.06	41779507.2	13223271.5	27726978.44
4152.201261	230344.3654	251964.1096	473123.5855	1055602.021	196319.6498	723193.5406	225395.2136	684400.7752	347838.8955	318015.4292	192781.3546	147990.4262	620577.1098	727462.2238	785088.138	531958.0623	963615.7471	419855.1078	1068318.65	243913.5192	304775.6469
3837709.035	1959584.457	2461819.126	3072341.865	4229629.846	3434503.299	7493620.745	3458703.43	4608638.22	5254947.939	5608183.873	1689505.349	2241095.128	3134505.631	4687174.049	7887421.8	4847671.821	7370569.617	3465782.386	5618840.799	3478420.482	4635038.781
3143875.13	2077647.399	4617194.9	5173718.908	9399194.033	6185107.943	12984330.23	6637849.094	7531870.323	5759687.744	6279891.767	5291258.496	5566978.187	6807046.405	5993576.382	9831619.797	7380254.719	12861750.23	8400801.908	9982357.944	3105000.207	2929386.888
0	0	52448.05844	61991.36767	134030.4881	73740.36375	39287.27939	14019.99386	54546.02094	56384.17626	9356.294388	38885.81922	74391.48905	0	35479.63425	222729.04	90535.78034	128810.4719	108815.7123	138431.4754	26669.00596	150723.076
1742997.375	1009070.53	2671136.113	4496450.269	3362646.448	2255881.353	2441056.621	4509884.275	3243432.211	2283471.286	2765639.198	3476713.405	2582876.164	3029830.949	4978817.157	3805595.084	4135752.112	2392636.76	3925304.487	3473641.563	801056.0505	2721576.705
283590.1063	265261.1339	402314.8603	1364719.53	1393852.907	803027.2522	625504.3445	702425.1657	723849.0321	696463.081	788487.2542	850262.4919	508316.2622	1293654.446	935435.6573	1152733.41	708312.7961	360205.9041	538141.1047	439549.5185	227782.1477	896673.5374
235489.3952	839070.3568	626052.8259	1008157.02	1105207.491	797378.9423	1607518.221	894331.6938	1051092.003	508821.877	736751.6728	1117700.329	741480.6347	778121.0807	933282.1887	1151543.171	1406876.936	1177138.557	1102199.795	1273622.645	874684.8933	713233.8908
124831.2493	140418.6439	460830.6954	1353261.656	1592670.081	1147548.015	1524303.057	719271.9578	635569.0652	462158.0429	653338.7191	469930.664	224991.8299	495653.0303	699658.5285	1207428.658	703133.0639	1016484.414	1132274.816	772186.6953	828560.2594	274012.4394
0	0	0	0	0	0	17775.22797	0	16881.52373	0	223043.0373	0	13361.10412	0	5124.70622	175457.2069	0	48950.03931	0	0	0	0
0	0	0	140108.1319	125162.3393	13296.6874	131904.9349	0	0	0	5936.769481	0	0	0	0	79625.43861	0	69130.14628	0	15299.86403	84960.49228	10833.64959
0	0	0	0	265967.77	18614.46685	76520.64868	0	0	0	0	17315.90803	0	0	0	109753.4969	0	0	0	0	0	0
1089935.712	945810.3751	1510317.403	1233693.825	1474937.656	1617899.896	1063878.58	1428727.744	1628301.089	1291824.658	1818421.644	998719.4957	1682451.59	1622933.828	1715534.225	2369486.106	1615515.718	1729546.591	1116828.152	1313192.546	1645994.697	904267.4896
0	0	380924.1757	228671.2532	70188.44952	63592.17964	735826.3094	170377.7588	364045.2389	150743.0931	243768.3786	179804.8923	275014.1572	194412.8514	530631.08	367164.9805	296364.555	523264.0331	355756.7929	138636.4083	0	202242.3754
0	0	16184.03567	0	24052.44495	19285.06161	88465.54779	28506.27767	15899.66802	0	13919.91426	29249.09939	0	26592.85356	11265.97281	49716.50099	0	16286.59066	19375.48105	45496.73783	0	0
76832.67085	0	535886.1873	656159.5085	166838.0976	573353.4388	425747.9427	644390.3786	231426.4947	446343.4677	146236.8759	321632.8032	214418.1658	290523.567	289982.6817	80400.0353	280612.2854	49836.8738	350277.8852	298045.7343	0	119496.5346
0	0	0	0	63483.42681	0	0	0	0	0	0	143423.2739	34185.22437	0	73282.44158	89115.36293	0	22352.19747	26551.17576	0	0	0
962160.7434	465410.3664	2098865.168	1054296.626	1496057.518	3709305.142	4096998.949	1526427.344	1211879.502	1309368.454	1952442.445	1074878.178	209517.3302	2415984.927	2073331.974	1335716.733	2613115.658	1719158.704	1386301.193	780155.1303	968426.1806	242293.6614
0	0	0	0	283962.277	172463.3991	47796.56527	0	0	101952.8143	0	0	0	32293.69051	0	380864.9205	0	258305.7296	0	382734.2651	0	0
0	0	0	0	0	0	612853.0317	0	0	0	0	0	0	0	0	99855.31036	0	110470.0768	146454.36	0	0	0
951587.957	1867837.124	2418934.555	5739673.273	587072.8507	2480496.645	2680745.051	3990114.72	2202359.298	921827.2823	284864.6823	1580549.854	1861021.723	3209714.479	3481339.634	4812155.007	5142170.378	1681376.101	3397162.383	7244969.564	578869.4241	191170.8038
0	0	0	0	0	0	1567982.135	0	0	0	0	610931.3695	0	0	0	648533.7269	0	212073.8776	0	250616.5333	0	0
1388211.963	768390.4057	377814.0662	2632367.422	2455342.606	1648280.528	4187278.074	2392074.824	783031.3116	1024551.202	1088312.628	970518.3995	4605751.925	2330711.047	0	1668407.27	1805523.803	3008192.424	1631090.85	2668101.884	1904195.429	1487289.809
1502801.314	7874388.767	2748020.947	6547520.59	6867905.137	4788825.996	13112759.72	3846798.775	7480375.89	3961738.921	5760492.486	11303581.69	5581448.118	10301418.62	7035598.072	9589384.736	6897003.446	6675711.063	2914920.045	11290295.2	13551401.7	14920928.97
875808.6712	796435.0452	0	2835481.775	2171073.485	3171569.763	2346538.907	2709022.635	680122.2286	1749632.809	1681720.657	2193001.204	3071925.331	4184538.545	0	3129129.524	2272977.105	2690534.224	3879240.556	3879904.663	1280581.822	0
25239980.07	14871090.58	13054736.91	21777665.76	36819803.58	27728741.27	28386399.62	14618717.21	10455616.33	12881205.24	17095281.92	21553383.49	15634551.41	20509877.24	16328769.83	21391306.8	20671777.06	27646590.39	19954892.11	26756232.83	21866915.15	18160033.57
16599590.03	13317825.91	29963401.58	27383188.7	51073038.75	34792626.64	38952468.11	33708763.03	22097668.95	24873734.42	27492888.91	25826330.9	28816253.11	29504088.45	26971352.6	42976087.44	36085015.36	47132350.85	32863884.57	39569178.57	35734718.05	18888252.31
10377090.34	9992002.494	5962855.703	9789230.15	14087679.3	17856090.04	10107646.91	9950526.279	11526912.7	11042102.41	8195786.097	3755998.727	15579809.95	9794374.043	11732674.82	16922486.51	15502724.94	18486646.69	15712570.82	15316315.31	8546992.862	7210213.322
0	0	10407143.09	7769965.832	13092339.3	7021835.057	14925593.13	8149130.339	5193572.495	9400303.041	6130758.517	2449223.742	0	3706505.677	3221808.092	2696629.948	4836655.989	4603362.099	0	4837021.85	1746974.671	5243017.949
399832.4721	317339.5112	154831.6951	484928.3446	491530.4249	597950.3268	398536.4886	711998.428	257375.4655	182272.182	344887.241	400351.3088	107238.1605	520046.0775	895385.3357	210677.101	362776.4797	745075.7646	566377.0182	194641.9536	518552.5307	58557.26848
38179689.33	57620893.01	43260013.88	47990675.48	29979495.91	43564433.38	68332132.43	62210698.55	26720002.97	46860759.47	28947575.86	33926699.98	50308638.81	20707904.9	31527462.76	33287937.9	42741764.99	35093160.7	36047051.83	54423666.74	25256114.47	63492871.13
0	0	0	25290.74636	111661.6846	0	17295.6972	21649.50154	49173.40966	0	0	7935.241089	0	0	100306.5265	0	67031.65879	47913.92636	0	31762.73064	0	0
1137401.059	2225334.147	2257109.552	3989970.685	4058606.987	3099117.414	3908850.55	3408411.659	848319.2025	3610630.008	1136187.116	559086.0757	1988057.931	4340927.963	936386.9454	7106720.086	5476791.132	4440221.5	828277.2646	6114131.889	3424167.303	4341237.259
0	0	0	35390.90882	112035.0492	0	63786.88307	70685.85489	0	0	0	0	0	4156.507992	23546.71579	0	0	0	0	0	0	0
295329.9132	205316.7755	672172.7476	815025.4601	548194.7841	1231585.275	1742357.851	2000453.156	1092601.872	1103026.081	1106411.332	519200.6891	1791551.964	310667.0892	585287.3323	1395318.243	1699850.547	1925616.45	822818.8587	622271.046	192885.2307	202873.4264
2119696.643	1162148.363	10510935.07	8189007.212	2196605.522	8200680.834	8160308.653	13993330.37	10470994.17	12373130.73	6197323.82	5814269.755	8976091.56	5351137.471	9540216.585	8791962.804	14094700.97	8855906.713	10784564.89	9353736.752	5393057.234	2767047.628
179979.4402	204313.077	188831.4316	801847.3609	1053442.745	276824.1865	959302.3986	954587.168	139321.1742	188219.4957	34669.76915	76015.43233	87250.60327	512133.8991	701279.4865	1981827.954	1350474.062	1285942.378	523909.7779	320307.6893	221713.6096	289444.3965
0	174944.9775	51495.32471	283480.0374	328098.2825	57892.01852	38456.36779	0	0	150498.9817	59388.61073	0	0	0	0	67955.4225	0	246738.2067	55131.74785	60074.22524	27083.01736	0
0	353900.6497	13102986.73	13227546.04	3573588.908	0	5021579.455	10379201.86	7266797.431	5607109.535	4440026.992	4366303.278	4358667.352	0	6605109.894	20067155.3	20530633.76	5551302.038	16234078.14	7783278.971	0	0
699729.8126	1130168.65	4909738.456	2540431.355	0	3219141.198	9053.871289	4968101.106	5389805.038	2510213.214	2287651.447	2975186.866	2715413.911	2565812.844	5392680.348	6666874.871	4163823.564	1869208.82	6998745.578	5272128.204	1694655.136	1850674.039
1334294.611	1529167.432	2467934.778	3208635.009	7563156.952	4013169.002	3645897.998	3209316.522	2367345.644	2286308.875	4976352.544	2634918.664	1613836.119	5501075.037	2416951.434	3254315.335	5197083.905	3546160.359	2412418.722	8483836.539	2157802.599	3330446.415
487095.738	1091131.781	1477480.498	2317132.801	2502628.441	2258593.424	2453400.662	3693181.117	1795378.31	1419993.212	3263278.32	1824785.791	4485732.945	1370085.651	3469366.215	3289796.263	3485524.381	5089305.063	3509848.012	4979163.064	3694932.141	2112652.183
7326663.367	6888962.008	13634532.25	9883507.622	6952464.248	15966243.27	15871162.27	15306320.92	12524465.35	18419401.73	7070103.136	18622719.48	23216881.73	11219245.45	11406915.92	11330386.05	15749633.42	14491220.96	15764439.03	19358341.16	10444920.1	6782764.579
12390500.24	16223293.68	42549239.03	42020765.72	30804622.24	28711253.85	30591942.01	53065544.13	48963504.95	15952259.36	27176261.71	29153662.7	24288714.79	26120723.58	31065325.39	55392186.05	48406847.73	36297979.61	53517081.47	63856059.86	34614835.87	45834709.1
259793.256	82442.99836	117308.2774	653866.7334	1172388.659	458832.702	790927.5558	783845.9592	661787.5237	828022.4383	375272.1413	226040.5946	92733.45879	540213.0603	631209.4918	123917.2216	501501.6776	206404.2145	203467.7121	734374.4704	471916.3539	67563.92194
55197.62553	44858.25979	0	48439.96544	410336.7766	219519.256	206909.8671	92229.89246	269245.527	32926.69662	401720.8241	407269.8549	129465.6292	142069.288	120728.7054	266333.1866	162319.1666	285538.823	71715.02844	491502.5956	354653.1131	797364.2499
0	59663.93251	315447.4668	211849.231	1087935.875	399653.7854	1306743.693	337020.5388	377955.8711	176391.2246	107952.2208	405201.0779	575754.3547	588324.3134	75335.38428	635410.3493	426373.3631	176864.0437	738295.141	462966.6582	0	180747.9424
3849252.171	1635844.032	9999235.84	7133558.659	13887815.74	12006877.71	7648692.606	9797933.527	12322460.32	9469846.658	11720893.51	7148828.511	7067153.21	5882971.269	7700162.004	9932481.33	7389858.958	8713271.397	11984192.94	12037768.96	10108457.72	5536675.621
0	755184.3014	126888.5801	435502.1792	308755.784	743567.7066	484595.9037	336113.8894	519602.2274	962070.3932	384772.9215	1013693.379	329608.7628	0	254029.2649	582703.8415	1214399.393	479674.5458	346412.982	637822.9279	932865.2933	434850.0942
13147811.02	0	3619807.125	6080714.791	2159784.926	2890316.682	14130357.58	9273985.778	3034868.237	2513358.905	545997.8168	5423061.961	4950189.336	3294316.729	6934327.01	8902759.8	14374784.46	13373268.52	12393433.02	8284302.854	1475645.737	8523127.145
959507.7657	829079.6085	405015.8726	69113.27625	486291.3699	267061.9845	999118.1398	2376006.551	1036669.171	3742693.771	1075799.543	405746.9861	93536.09238	278977.7011	1147562.648	814778.9132	302955.8211	1235457.984	151588.247	373893.8172	136080.7016	807683.779
0	0	22302.16715	33517.5597	174363.6418	0	21823.00949	0	26196.96721	26582.59069	25219.48164	20308.79438	137925.9046	24210.73218	65083.08635	68650.45592	169391.6659	143782.6056	32920.29144	0	111051.3412	24147.13229
996847.583	2019065.727	3433878.231	6259523.216	7303236.51	5751115.994	5319137.853	5145737.793	4844737.983	4846145.161	4817085.971	5415939.664	4469233.891	3581894.895	6870770.608	5817004.898	5530410.815	7506311.406	6919199.19	8116639.391	3433565.949	10781364.7
1318825.996	2314205.301	4027103.807	6145458.544	7153012.736	4909952.493	6991554.247	4581770.327	7174082.949	5727678.64	5720736.13	4572249.179	4673399.405	4313507.789	4108435.898	7158293.529	6626532.578	8776866.784	7572249.795	6838086.939	4402989.556	6211287.169
211371.0252	0	232737.5155	0	423545.6856	0	312922.4862	202992.8728	247859.9951	567661.7593	471896.581	919265.2604	719271.6426	0	194645.6207	720930.8534	405117.601	5193357.63	801893.1331	449487.0073	3639252.31	0
0	0	49443.12107	116425.5101	363278.7907	229967.6393	355793.6924	171654.4005	162845.2559	44578.07495	15224260.86	201846.2508	265444.4894	408011.6585	209006.3687	781361.4163	6402197.249	366156.0807	178429.2034	121221.4492	79151.26936	120686.2334
4075545.389	8235766.07	1394323.487	1081206.423	7572228.402	6592942.099	13071529.44	4040186.043	3187843.487	5562952.704	5014877.362	5614333.605	3064083.075	4574830.768	4198877.642	9723599.081	4243046.464	5557186.006	5084062.226	9378995.654	8261163.023	4465060.396
489866.6432	127196.0847	0	760694.4111	889873.3141	247091.415	448696.0381	317512.2026	0	0	0	103613.9577	0	162328.1286	[illegible]	0	313207.0712	918344.5808	162434.5876	177859.4609	252127.7764	0
4014023.011	6051546.786	6343171.997	3043304.998	0	0	3382257.027	5311364.938	8224394.663	0	9379146.804	8695488.242	9391737.958	9150658.124	7951636.611	17002295.85	21069777.64	12221355.52	20174241.75	12102700.79	3886724.609	6106826.904
469898.7685	914852.27	1242623.291	449982.4704	626492.9464	897955.7195	1421527.003	1159343.243	517080.9311	1449904.183	377258.1011	1343955.808	656477.5162	498735.0235	1012659.742	1200261.338	976206.1381	440615.332	1020787.785	780969.6643	246750.2953	412785.2747
43060.64677	0	151926.1075	0	0	0	23243.33393	348131.1067	146156.4801	144664.7565	83569.52029	0	56297.96749	286174.5092	340129.5769	329107.2877	42738.04308	0	391487.2047	159214.5232	9032.247294	0
38900.07714	0	0	334975.9038	1965232.961	103872.1469	1110239.695	58866.01334	32999.04296	0	0	0	0	0	0	69468.73088	127279.2931	215161.5032	303444.8481	51617.02845	0	0
373323.8284	462689.8349	1693414.889	1295610.366	721780.1345	343418.8237	1414916.998	376753.1469	1766444.891	1464877.112	895733.4776	1791369.414	966463.6991	1191457.423	1859506.832	2445414.704	2099854.654	1865336.064	2083016.115	1084989.809	694785.3826	1864414.985
6577571.152	2391269.296	5176047.908	3086444.848	4054167.181	3156117.486	4370426.55	6144164.302	9000292.224	5186247.798	5460700.005	1403496.112	2166631.588	6376581.202	7648252.196	11660258.46	4410945.135	3063880.263	4757255.952	3430911.52	1524722.289	4097573.64
869884.4705	306573.7554	463868.0365	1243494.734	4691269.601	1047786.798	3233135.586	858318.6668	815190.929	163642.7996	734327.9031	657230.2621	1073720.325	1557070.434	752431.5165	2324065.435	2521512.103	1708389.007	1274182.508	2862746.363	334527.7049	363674.9871
7987980.821	10028289.64	16845495.34	21147154.17	23710602.49	19422362.41	24188479.28	20626127.23	15795619.49	18872858.35	15352151.46	11194980.22	16367308.41	4107193.289	16953845.32	25796268.85	20493700.9	27744818.03	20060226.5	33994833.45	14305500.7	10203040.72
0	182011.1284	419040.8082	365532.4281	188607.6414	182920.6609	0	0	0	427197.5917	487569.6453	0	184657.4001	68461.25295	0	356013.9016	274509.1286	11914.4695	152966.3194	656895.197	34866.00018	584478.068
0	0	264215.8322	380407.7054	204943.0434	449511.1692	98556.94327	416878.9035	5361587.369	194300.2983	4730137.316	191562.2364	173610.3928	0	3780941.904	5630189.961	337596.204	244381.0272	146675.8038	5365796.875	122787.8214	270523.6158
0	53858.92129	118747.7674	86742.99732	390471.6413	211458.8797	502025.1698	279962.0956	240877.7854	0	490506.6985	0	0	1139153.086	101619.1731	501581.156	160215.6616	1091217.538	280162.6485	466113.9713	182571.6155	0
1266497.378	6301478.645	1201780.944	3351599.704	2092606.784	2231169.731	5336809.56	3037483.403	4585357.671	1155680.456	2836671.476	3008996.615	2855638.184	3217842.439	914121.0597	4705786.802	8164252.246	7561513.517	4264553.82	2774131.964	6192492.182	6692745.516
3666930.724	0	783649.1178	1038587.28	4612139.419	0	5378920.202	830307.6345	0	465003.0046	0	1147240.269	0	0	0	819919.1829	590382.2406	997847.7785	0	1199877.951	2283480.148	723866.8938
3229757.877	2684874.943	2505911.367	2971638.152	5193242.718	2095567.516	3289463.103	3059334.074	4399693.553	2642335.338	3794204.461	3087029.42	2506903.064	2752751.732	3614601.236	9288603.101	6992276.508	7959562.234	5789106.812	7579869.148	4131910.244	1655427.092
88499.69912	0	183431.1076	352757.4714	147497.5554	437084.9309	294774.9906	208829.9155	60514.02799	0	118771.1044	35058.63254	0	32169.20593	278232.2045	78555.97261	92888.36513	485221.3934	72489.63013	467111.8613	119367.7393	165409.1692
114365.0666	9196.14758	67138.64129	71500.19127	167076.9345	103104.5762	51616.64293	86804.60172	197242.5741	43610.85453	58921.41792	87351.61007	124518.5352	173957.1578	186111.5696	128157.8374	83286.01	524930.5754	120078.2918	190198.8302	83875.81815	0
0	0	0	0	55500.59442	0	24727.87022	0	0	0	0	0	0	90892.94736	0	0	265515.9485	53224.78845	0	10129.64487	0	0
0	0	0	11847.67957	64052.89414	0	230523.3652	0	0	12952.41565	12516.71183	0	0	0	9749.184944	0	38313.62773	68995.73125	0	0	0	10495.25136
100464.472	130910.6253	40363.9281	75732.09226	113826.5881	67482.33081	133046.2452	298608.0011	226608.0138	247519.6698	173084.3768	400901.5997	87440.99788	481702.9863	334183.5723	181192.3833	386499.3633	282172.8667	675527.904	379989.7477	0	169903.475
0	127731.046	349216.7688	298499.4393	293212.689	227675.403	424876.747	398825.466	312483.0773	184826.2653	245823.4565	592057.7903	255995.8968	155684.5753	305397.8283	325334.9698	392558.9799	130827.1841	371958.8539	130945.7591	213127.9669	384168.0484
58591.91638	0	62092.82265	89542.44983	138273.4409	117984.7423	0	85286.93895	0	0	78683.59475	0	21310.78157	0	0	23384.41497	0	0	50921.35652	0	0	82827.63455
37405339.65	56128598.8	35015198.79	38364195.88	50121386.76	61653883.77	106264845.7	80659734.53	52371853.4	59945343.43	65891093.13	31040756.08	31789952.29	21294110.95	22883641.67	42585802.34	56599173.47	31378046.01	25954279.15	36079624.5	26138285.12	42469398.59
28750.90147	308562.3168	643766.0542	838772.6384	949101.8192	662648.6195	1295349.875	508585.8687	634936.0001	360258.6988	842420.4241	848217.5869	677005.8087	1249073.772	1186734.182	893041.4024	633533.9929	1139681.605	1127533.169	630443.6236	413859.4911	146852.1788

84160291.76	49975528.53	120040104.6	116579364.6	109694906	91547963.28	140540232.3	149231216	102741875.1	95734106.36	112741347.2	78780062.92	78898741.15	103646675.4	138188720.2	156123110.3	101881622.9	96535636.04	91202900.96	117044223.4	78424521.62	73622865.1
0	10473563.33	8611217.56	5708819.517	10018033.04	9760313.217	16948877.36	8309348.455	12887980.64	18148632.68	10896474.77	7476994.982	8004537.676	8613551.753	6017323.562	18036101.09	20853372.46	9348177.162	12150712.89	8057517.618	12144911.26	10903093.56
0	0	91199.09728	599548.1029	0	222921.3504	250038.8827	184396.2972	349150.1615	57730.94569	130981.0984	627033.5851	337622.4744	404007.812	0	679121.0161	594133.9894	1015422.552	541140.8351	787282.0808	565305.1712	721999.5133
1407604.734	1962517.812	1113231.062	2152922.858	6023148.28	3004249.37	3895395.829	2048188.05	2096151.632	1836140.159	3158077.264	1538697.69	1374360.312	1453519.447	1723214.61	2679429.701	2823374.33	3109596.799	2471587.53	2327044.388	1950975.066	803482.2196
2867475.226	4444878.25	8441599.954	7209231.467	8375888.288	8100702.195	8034769.298	8030820.216	7230074.007	7130689.12	6772897.734	5037455.621	6468302.046	8832793.042	8938458.06	13012152.65	10814865.1	8630047.871	10017376.9	8106656.449	5162347.363	5791753.396
0	0	0	0	0	0	292515.5457	295540.338	0	0	0	384845.8651	201269.0152	237878.8258	0	122225.3541	0	374771.8407	0	530051.072	212121.4879	266227.3173
417214.9014	25249.24884	87335.60034	149339.6124	3888049.18	295042.6702	758894.22	98980.65073	264113.3242	120619.8953	87288.83456	124378.3599	149728.4422	183664.2419	283978.2167	1064005.1	510423.1239	2174526.354	472065.861	755834.3964	133768.1236	315058.9991
760710.4849	193885.3273	225031.8433	355073.6816	474958.899	1259582.777	993274.835	568285.963	578378.4512	807447.0521	709373.4482	903131.1309	832315.9039	1009459.166	900450.9571	998712.3202	1163312.036	1054097.76	1215515.933	1087251.593	147049.966	597961.4268
120765.7142	0	230982.9357	44249.49489	0	228672.0041	258277.3767	611335.5805	151318.6946	357532.5238	0	0	0	188409.1299	345473.5131	385750.2359	230293.504	127034.9906	1015117.593	0	0	0
638389.8676	815325.0865	828649.8302	779570.9253	976205.7938	1245747.163	573758.9509	970981.8183	808682.7642	774920.4348	1150641.537	851341.4447	868924.2005	1217647.186	994632.8262	1031418.96	617741.1	1467345.089	1399514.199	883292.8951	1044886.135	694527.8257
345133.2583	0	0	0	125350.5906	0	0	0	0	0	0	0	158225.8434	231233.8814	0	222474.0345	0	404304.4782	0	104814.9447	0	0
0	0	0	0	416653.6081	0	0	0	0	0	0	0	0	0	0	0	0	0	0	0	0	0
314483.6414	283417.3092	785554.0483	2766593.241	9362557.39	172751.4468	2635404.417	1394412.676	1006264.742	758201.1673	963035.7091	628625.6625	675498.8515	852169.4047	1352227.882	1700293.686	1215748.132	2467176.714	1174626.145	1838300.014	1532338.799	1257416.173
0	25704.14644	33769.65134	45083.42988	54142.89952	69989.79557	19445.31022	0	47045.73231	0	0	0	0	18925.22881	0	44938.63453	0	143995.1822	64331.62134	172373.3906	0	0
0	0	0	0	0	80152.69595	403730.7673	0	0	0	0	67545.7125	0	230272.1389	0	38029.259	129804.9246	0	0	0	95014.5212	242687.9102
0	41127.57573	0	0	0	25277.27574	0	0	67253.60108	53994.20369	0	0	0	100799.3148	84971.88045	75555.71488	0	13052.82276	76701.82948	102823.1343	0	0
771058.7544	125900.8708	830861.0856	449410.5183	254959.423	289977.6188	385698.9403	464728.5629	201055.0576	578005.4846	270536.6719	583166.2374	273452.4489	1703832.287	537330.5071	1127788.033	507624.847	428835.2416	1025064.57	795673.1651	561956.5661	1566613.253
5684.010345	75734.24186	29031.24825	0	208423.8437	159611.6614	208743.9356	192000.158	70056.35612	59805.68374	20269.96194	117546.1134	52407.94996	69882.3578	144661.7711	219710.5533	356801.2426	111378.6139	215902.7867	202957.095	0	60251.83685
142944.3364	22444.7221	41817.85811	160002.7732	0	0	97198.73977	47851.90217	127795.6634	0	92757.45483	64573.65509	41486.97537	8264.373363	32578.24503	0	65967.67797	0	180026.4806	0	65877.48941	0
1315336.995	1747470.086	3479753.305	5801842.411	6783867.55	6314609.395	9882771.468	5921835.474	6584414.744	3013048.146	4617854.808	5773249.271	3881095.23	4795406.181	5440285.895	7429468.776	4022144.768	7844353.253	4991746.835	7123433.648	2261016.748	4026417.472
5680550.708	5413508.909	7965172.044	10286359.88	12008965.85	12761902.04	7827536.428	8847983.823	4918007.276	3651450.297	4586950.173	4476900.976	9912559.196	6580270.265	5466932.552	10107079.59	12179786.59	16352088	4861121.899	16146948.32	5490916.059	7787742.689
371618.142	499934.6856	224688.5503	409238.7139	297094.7678	492717.9152	437320.4935	567014.6355	481433.07	371743.6181	656456.3444	579624.5796	269351.5147	472014.8283	432319.4227	5823595.111	263490.3867	599853.333	207412.1008	402805.9376	833646.9428	77429.35194
7136834.374	13875261.02	10165147.94	13817041.75	17370235.71	8079916.236	16333001.86	20248289.58	14495391.13	12681270.96	9725456.227	6825642.3	9190763.861	9230895.882	13152579.81	13362726.51	10577999.56	11878146.09	9916503.923	10560363.24	8469653.956	8019156.418
16184517.02	34797545.21	11064021.82	20298048.38	24727832.53	14915465.52	19807349.95	9235258.235	14154189.59	15758261.09	18035757.07	11505639.69	7729541.047	13382008.93	10882416.07	10671275.86	13142631.41	16771626.87	11671838.78	14112146.71	15782697.1	17819124.66
369449.0203	363013.2146	1119916.551	297798.0385	255929.4467	749690.2502	630196.041	1028955.152	1136476.129	741454.3572	870345.9731	366609.8938	115575.5562	0	320663.3425	156989.4039	0	0	0	0	0	595643.3636
50377.71175	111815.6188	907403.9984	150757.4292	1431493.702	903988.3677	1853136.611	262498.3776	1162418.775	681392.0817	1009091.013	694111.9937	465405.1143	854166.379	2711729.536	2832800.824	1533154.111	1495486.541	778010.2958	1697701.056	668737.2625	0
0	0	0	0	5190.708433	12201.16	0	6258.745345	10781.57318	0	0	25056.00096	0	0	0	0	0	17174.21372	0	0	0	0
158943.8594	279437.2636	75398.77419	0	61961.034	165679.4632	498391.9746	81440.83095	96585.39949	72351.62965	0	206224.8479	0	133639.2875	874056.4937	493234.0234	273827.4679	586886.1025	699805.0373	662038.3653	162674.0964	35548.41235
0	49031.04969	40647.42176	61852.78281	126139.6232	55836.43314	0	26454.31097	135278.8497	94439.82162	46807.75945	42387.97056	11526.59688	300793.4639	10359.39384	106340.0823	74761.4259	35859.66092	0	47406.74642	0	0
1486396.336	0	1041411.266	2223902.923	3419885.886	527037.153	2375450.399	1170287.773	1453079.879	1765660.62	1848968.916	343786.5006	167941.6405	659927.5489	1944852.599	1311654.092	457228.5059	1412955.65	1326017.464	586073.0963	856886.4725	488616.6311
841686.7414	1078112.738	1572106.06	2534264.981	3906153.386	2346602.721	2514455.004	3091715.1	2878788.797	1560030.561	2712334.796	1976914.363	1881564.468	2251874.05	64583772.48	3558827.935	60408631.54	4562963.156	1904561.624	52495021.73	21226588	2212401.856
14891217.83	12832589.9	35195971.93	26069227.43	32221696.73	34559294.37	28498713.48	40571553.95	45595554.04	40484398.09	42375341.75	59982265.33	26871092.07	37536237.26	44367243.93	74842135.67	64610978.77	51972750.98	46629914.91	39921397.22	32101713.74	32370171.66
1651023.526	2879805.438	2766032.765	4637410.896	3654655.185	2517661.016	2937446.031	1727526.038	2061965.468	2672324.759	3646061.359	1762752.734	1876934.775	2092227.964	2240381.844	3257837.061	3026285.412	4771428.105	2848063.652	4888058.941	2647690.249	1611130.65
2311417.882	2065477.473	0	0	139333.2666	2012692.89	423999.5072	25543.42098	576403.0207	39948.32947	232918.9341	0	188769.536	296047.9976	597521.3489	330939.3248	317384.2394	0	42112.39559	0	415448.7913	734189.7467
11805814.34	12651455.86	3525145.353	12687294.27	5404036.507	4897790.431	14053716.16	6744606.498	5508964.04	9344124.19	6247353.429	5476280.933	9992380.453	1885579.676	7709934.904	10306266.84	7026946.597	7758032.444	8695507.308	6238980.03	7010258	4590896.368
0	200176.8891	0	0	244826.0378	0	0	115537.0025	198419.0499	0	338303.2387	0	0	0	0	0	0	116201.7696	168235.4701	0	0	0
0	0	84321.64863	1863330.112	86449.8984	15956.05726	14058.20144	0	0	0	0	14910.35647	0	28866.63166	0	0	0	117672.7394	0	44726.00711	18301.2372	0
0	0	218521.7195	207899.8306	473432.7479	403584.5907	176193.4656	204455.8085	120340.4963	0	189949.3895	0	0	0	0	218023.1095	232667.9942	196961.5382	271845.9498	191193.7627	307943.0386	130509.5689
3714142.285	7355977.778	4505523.174	2490776.801	2532215.386	4692659.587	5415544.532	4929783.002	2159571.393	2474892.164	2559986.625	2772473.902	3146806.846	3138208.252	4386281.919	3268320.286	1857013.627	4557880.254	2360812.516	4336713.014	2878888.34	1605356.42
319906.4668	542628.0918	773428.6621	611647.395	1594288.982	1028955.152	631503.6837	973311.2896	888182.8289	856257.4063	827083.1328	442959.2659	343542.0584	1183328.376	1507760.029	2599331.018	1585700.441	1363290.61	1487701.337	1263227.166	367356.9409	275216.6104
14418736.24	12188232.18	25839941.99	41026071.16	51454496.96	23561001.26	31205284.81	38628789.23	54909434.13	62286660.56	43673667.21	25999634.59	37995854.33	36431241.35	29653132.27	47732838.44	36650267.43	29422273.22	34333429.21	18119645.4	31557812.35	40253353.3
1948737.392	2260944.029	3983552.93	3740173.872	3090670.471	3705151.774	5205708.604	4244929.703	3767439.482	1610040.563	1377295.987	3785878.333	1854361.586	3145972.938	3194541.115	3102061.56	3185965.251	3512860.684	2858179.012	6060485.819	1825497.177	1471018.19
467844.1057	1877973.272	2056264.197	1452344.856	2074755.762	1141416.869	2097936.741	1836200.806	1637890.201	2396704.438	1309910.559	1782501.802	1042449.146	2034044.699	1984620.98	4381374.021	2297262.089	3414510.004	3388996.042	3438497.018	1181547.773	1255785.573
834850.5211	2951279.467	4182525.084	3590461.005	4369236.504	2894032.575	4461987.81	2782830.253	2841544.662	1755849.548	2403364.218	1034550.763	1852003.424	1011536.353	2768685.015	3906257.546	4238216.196	757279.1235	5297820.399	1985530.216	0	1119285.249
0	0	0	55258.22527	350045.6458	0	151510.1348	0	190724.9198	0	0	78087.82801	0	0	0	0	0	683167.0168	0	0	55413.98259	0
0	939593.9451	962394.0561	1964865.249	2010674.136	1482644.146	5559649.641	2413710.484	2419185.649	1441587.652	2320297.27	2641575.174	1181274.697	4330216.525	2680463.012	4685104.593	3330696.033	3209491.862	3124887.815	4853509.974	2404018.276	3914784.939
0	0	258403.5183	385845.3012	560907.2867	617077.3435	768709.7362	490455.8968	337047.0049	544816.3476	568757.9111	446886.3853	622531.4123	646003.5442	698772.9816	977367.4001	1098099.768	1270381.755	1054156.755	670467.6237	443686.6144	192724.991
0	0	0	0	239021.7556	144198.2427	278680.9608	0	0	0	0	0	325130.1925	0	0	234677.6603	0	698510.2185	0	0	0	186660.8738
0	0	1280966.742	1385483.686	1860228.251	1131276.447	1403244.319	1485626.315	3711654.441	1475199.797	2213241.533	0	0	1094238.422	0	0	177414.2333	0	0	0	0	0
3316646.086	2437223.858	1968777.466	4961946.317	4878567.74	3510617.188	6737085.534	7968476.667	6283470.595	5159857.908	5291593.753	2097594.306	3497779.322	2881158.489	8803411.162	5445605.728	6666194.772	3858102.979	4723104.293	4431021.863	3902509.299	2842345.424
3872231.05	13045478.86	4578575.132	14443015.41	10223542.48	20668304.76	13278896.94	12640561.08	14886968.32	9887115.033	11653691.5	6409935.092	9478137.637	10502939.05	10835314.49	15527547.49	24495801.25	4391811.804	18147960.34	5031689.67	17801753.69	19598311.44
0	0	2003277.815	3335818.925	5073768.36	4486541.287	6535187.825	5567539.892	2129993.526	5497482.535	4558458.379	8744833.031	2267205.331	4396434.309	3287768.099	8685393.729	5222014.158	4286823.608	7235406.463	1775320.649	7415966.279	1883280.332
9886861.935	5003614.694	8650179.158	15217075.41	17460424.58	3326793.123	19252455.19	13191680.01	10157492.86	20112676.04	10978563.84	23795211.12	16741777.81	16132106.38	10899192.58	22389431.34	29028725	20910273.41	25340427.16	11121846.46	17699059.77	8575556.764
2236602.182	3940939.946	3653512.89	4360960.142	7941021.025	2083545.143	8180633.917	3860772.123	7738994.104	5577350.785	4911237.313	4231249.648	2987282.595	3501415.586	3440229.582	7697492.856	4740676.502	5252967.52	2122200.936	4831123.65	4772719.107	5683678.579
4984232.487	4303616.469	2590346.564	5592832.291	6800043.988	5206907.749	4365386.013	5539740.756	2542668.919	2781969.658	3954510.291	4935539.176	4189652.003	5167037.969	4630449.065	7868637.93	5184379.853	6699636.855	5170238.073	3897101.086	3297252.598	3196422.02
13479312.27	8115618.47	13961827.86	8210281.594	10985444.46	5376370.74	7243861.72	8397161.266	15352421.41	9038086.083	4792926.504	14133563.6	4313218.769	16218023.58	14180717.45	19484236.88	8923461.389	11486194.89	16367385.65	9206690.672	2664968.533	10658006.09
67883488.09	80051747.09	80137744.38	77816475.72	166240919.3	109034473.5	133850668.8	102651765.8	98632776.16	77904225.23	90562670.99	90422530.62	106267500.7	108863318.1	95355871.74	96473468.04	98804702.58	141626835.9	94466264.68	140644308.3	100380431.2	82697195.3
28336853	19625744.38	74405140.5	74843973.92	43022479.62	87273659.49	51433431.52	78788740.86	132599933.5	98892365.37	110113494.6	85625286.28	135595188.9	117475266.9	130404528.1	141361845.3	150935314.8	107112918.6	69198372.33	48203709.96	39269958.99	61513452.35
652734.6524	699759.5417	464490.4237	888959.9421	0	0		819687.7841	1729412.985	1500788.504	1743594.129	807723.3921	567865.4099	0	2379624.461	2125108.111	2721309.033	3057393.705	4303813.939	2008984.547	1282210.281	0
1538920.514	259332.2672	1022673.18	727614.4204	657038.2267	0	1229038.27	0	819012.2605	2047312.067	229937.5619	0	0	367393.9892	646281.84	777370.0242	641566.139	561956.5661	516364.5971	913697.0639	0	392282.5061
178267.8246	134302.6203	37561.17103	182003.5422	316322.2166	213627.233	687280.7189	186502.8042	327812.394	149512.7693	253730.4505	132185.0591	115361.6897	26713.55159	224425.7705	128538.1078	268591.0645	382538.1527	301846.2764	164501.0164	296814.1903	26954.22241
1207182.869	2749571.438	3586293.29	8983963.179	4585019.769	4345594.058	7377561.19	10503130.87	12986030.75	7920358.971	10431505.66	7510450.24	6797595.535	10331711.21	9941805.632	10701095.88	10409061.47	7653216.563	11884306.01	10878176.85	6349198.787	7660097.088
1097514.34	1673847.826	2236120.645	2557846.758	5186927.172	2387488.553	5888072.957	4212754.092	4074590.147	4068035.981	2911689.615	3679249.968	2535250.709	4272112.999	4529807.991	7395003.332	5983624.718	4956494.945	5680197.925	6074750.101	2500924.494	1515072.676
0	256809.0576	0	92572.92241	0	0	395286.4968	0	139978.2122	535206.9289	141520.5254	166051.1417	0	0	0	295221.5708	219103.9769	238121.0461	49158.59654	0	0	0
3765264.942	1003900.853	2667518.487	2417361.871	1757620.625	3604485.104	2751822.083	2009351.494	945528.7893	2504474.726	2013221.02	479655.808	2283822.72	2785995.896	2554786.978	6715857.715	7864304.346	5368442.938	4925340.853	9044777.393	3093516.954	3239455.776
2390305.052	1026505.231	942499.2901	3823322.912	4956825.279	4099531.311	6474488.682	4375416.996	4832488.909	3876605.57	2748744.539	3421986.201	2896252.477	2779349.359	4322663.014	4904615.303	2359779.929	4592935.285	4849168.788	3982144.358	3254887.411	1735252.688
2660466.111	4178387.097	1607317.106	5220152.499	2213016.045	1691844.639	1843544.325	1935115.823	2303096.91	2042375.705	1179387.666	1364437.279	1377571.578	890980.5612	1692606.428	1285214.613	0	1036899.185	1264774.097	2379011.515	1195911.25	1873852.114
12254828.03	11156427.69	39458865.29	41316663.84	26732510.01	19778500.42	40779050.28	52023116.6	38432760.38	49549393.22	29892409.69	32172630.72	30369698.84	30188745.54	55279460.09	77723356.43	58687634.42	31565152.11	98773356.16	55101398.55	35206575.42	21215705.95
7195105.712	4804973.309	8266603.232	8700780.044	10811694.27	10152685.57	15336901.77	8598226.809	11070168.23	10974697.9	8553324.039	7653155.892	8522171.892	10150469.16	7507294.292	14617170.98	11920112.55	12773986.47	10329982.92	8472991.244	6920769.873	4742366.653
4644746.788	5389404.909	7029547.354	12668973.72	7852919.07	13311516.96	9320109.21	12509093.99	13257681.29	10580933.81	11500408.05	16504713.59	12685646.82	11409767.17	13295899.29	12958222.2	13241142.06	13002656.98	11967996.02	15503804.16	12398016.13	10921157.83
0	100614.3225	574200.0804	380883.7891	767451.9096	981381.4589	1860622.316	1233039.786	783871.7364	462655.9664	641121.542	617685.6646	1006081.775	0	377688.7535	466473.3889	1320975.473	878005.4527	857103.6814	710609.5987	0	0
2437412.582	3141326.729	3644169.291	6088773.346	8157533.448	2598124.421	10000999.28	3166411.631	2808015.309	2264650.175	4328561.016	6247282.049	5335283.248	5760185.406	8535383.706	7974501.908	6049482.742	10091476.58	6742262.96	4736852.581	4955061.199	3777507.414
3770662.247	3210897.901	2976189.801	3647671.772	4012890.246	3408612.03	4337229.993	2529858.614	5301081.803	2980523.871	3595814.165	3180415.885	3277455.002	5987790.04	5047278.836	4972579.494	3782189.659	6372925.575	7044649.814	6670051.051	5385674.723	404728.2857

[illegible]	1066791.268	424884.5985	0	66933.79616	93690.43058	141245.3884	0	127307.2041	411666.1613	1078806.812	716641.0645	67008.15816	987079.6609	414603.8749	0	32630.86188	
[illegible]	197814.8308	400205.0204	263058.151	98449.79857	37228.68985	117105.3471	220327.6152	323287.1142	237697.6816	398247.1311	442065.6653	303727.4752	779719.5097	185639.7848	120994.2284	59250.44805	
[illegible]	[illegible]	0	401220.4664	0	70524.84455	105523.993	505119.0317	537032.267	379527.8279	323435.3031	253348.8428	452817.7573	572100.1615	277409.8316	1438960.672	569167.085	
[illegible]	[illegible]	139602.7913	96421.85384	51734.95349	136805.8867	138806.3737	163321.7437	340122.4791	0	389933.1585	580794.9019	642456.1188	252593.5015	170446.8811	0	0	
[illegible]	[illegible]	21481533.75	19326054.97	26922354.17	15453174.37	15052240.3	20959272.66	20939741.71	24759916.16	22042618.74	27246191.97	20779321.09	23126010.98	25480639.53	17391058.3	28321763.86	
[illegible]	[illegible]	34713135.1	36685804.37	32844870.61	33575786.05	52037534.35	37894243	40264087.51	45951350.38	46581312.36	51383896.68	53902269.66	49820107.65	45258515.09	25718085.03	33158438.71	
[illegible]	[illegible]	604883.0314	382831.8131	125168.3128	398181.7988	451659.9301	733029.4364	635594.5851	525185.4729	1017279.486	916635.0298	1449974.578	712187.3116	975137.8899	640346.1665	522149.2464	
[illegible]	[illegible]	725804.4724	1158608.217	635564.0787	896836.2206	711655.7138	304033.3725	1080548.093	685680.8459	1282192.95	850413.2512	616974.5026	1200134.709	545528.6219	389653.9153	642486.8527	
[illegible]	[illegible]	14996.72384	8172.391727	32779.72998	0	8858.384532	33475.42689	24345.44541	0	9689.34637	11927.43775	78484.74558	5529.868141	13247.74965	30043.26967	9988.980429	
[illegible]	[illegible]	608554.3256	232054.4177	215454.9281	612704.4391	196488.1945	322372.661	677790.8704	224126.6812	389540.3942	274855.6011	1231798.033	485538.6187	453996.8681	600841.075	528261.276	
[illegible]	[illegible]	745793.6707	0	52042.51936	0	68068.23042	0	106680.8213	236356.4957	560934.6081	533121.0445	748978.2339	338259.4505	50290.90826	79859.9363	0	
[illegible]	0	162741.5798	58983.05336	109634.9126	126411.1025	421702.4642	714131.9919	960492.6747	225971.2688	754630.6684	453183.1398	721902.6284	896670.5161	604596.1061	260326.5988	284846.4309	
[illegible]	[illegible]	2730873.267	2036433.947	2925975.063	2954859.914	5051390.105	7208939.148	7064159.158	3904529.456	7200206.728	6005764.174	4386639.086	4733930.724	2671108.012	2634334.242	2541239.351	
[illegible]	[illegible]	35713.8723	151089.6768	0	30862.86231	44882.52411	36723.64115	133867.2388	145367.041	96172.31324	189367.5404	0	0	46142.10586	0	0	
[illegible]	[illegible]	14852508.92	16635709.99	11408900.9	13695466.26	11610312.92	12605897.37	13948975.8	12020709.34	13818880.89	12317178.96	10469006.8	12015531.74	14389820.61	6235582.783	6634980.642	
[illegible]	[illegible]	14463297.61	16343801.06	16007481.34	14873831.2	16532794.69	13083246.33	17285113.64	14900405.13	22353800.64	19950900.55	14761503.2	17576975.61	15139479.71	12970379.13	13954570.47	
[illegible]	0	0	0	0	0	0	41191.02888	12718.49999	0	9100.122435	15569.73316	84747.39495	0	16451.97004	0	0	
[illegible]	[illegible]	2115511.513	2614070.387	2590021.101	330492.6712	81703.05028	619495.276	1243376.633	1238979.36	218838.3672	1763648.192	1203490.958	1061760.659	1484344.356	0	604019.7254	
[illegible]	0	0	199027.5088	24040.15589	2194917.835	0	225343.2721	261720.9886	989350.4916	423443.756	3473779.393	313227.0882	105738.5299	448978.6173	184621.5404	73643.75559	
[illegible]	[illegible]	3714196.794	2504124.847	1788997.802	1893776.988	0	3906915.634	4128283.255	1910777.008	2157843.823	1368117.825	1331932.797	1948162.826	2920791.607	1794288.081	0	
[illegible]	[illegible]	10896.78981	13661.03956	7006.848263	21427.12154	20694.56046	10893.55156	0	30947.02135	27511.12025	46326.33026	21513.42687	40455.99448	22683.24141	0	15926.66352	
[illegible]	[illegible]	1328605.925	2321819.633	2487044.442	1372604.612	724868.3085	1079079.072	744493.5473	2189634.259	2006663.245	2163461.236	995858.0114	2188889.864	2100139.253	844616.0441	1126123.934	

[illegible]

[illegible]

1396180833	1663406958	1447814319	622939245.5	1160934733	1514051679	958173975	1447493933	1551486291	697321459	1394210351	940841155.6	1699865722	1577175473
386570.0542	134871.73	402324.5899	353284.8158	353225.036	897053.7061	326697.133	292679.4112	378575.7012	266089.3261	136596.9486	167398.6952	336539.5228	147162.5001
252185390.4	648349734.2	667468192.1	176585110	609782452.9	226845311.1	590323700.2	1197526050	680006351.5	638071780.5	353662301	337093830.8	578161878.7	537169539.5
45349228.22	52574972.89	64326889.74	52676068.44	35865019.82	47153369.87	34868561.34	14248033.45	41940530.07	29349649.09	60182668.98	46736705.46	23079975.5	53439786.55
760257070.6	1455228669	1506001899	486620436.2	400599601.1	1252381603	415574982.8	342083818.7	1212086906	880451500.7	481247189.6	860394535.6	718937498.2	317699825.6
804138.3367	200757.6016	916079.9704	1825250.308	769232.8443	1074086.044	1112465.865	212971.5756	247892.269	355820.0271	974185.0182	350626.9499	657438.394	233790.264
2098007.922	348738.5243	1116189.549	5454627.902	1334024.603	1426948.778	778893.6168	1145349.468	339954.805	1340862.774	1072183.443	1068680.151	1562656.903	638410.7825
76742818.84	13126238.41	27139386.43	26512416	18290499.35	29340887.42	15114648.79	9752118.684	22656865.43	15587309.91	21850734.75	16009723.82	21739732.98	41865817.33
17118017.18	91305242.55	41224847.57	51975735.94	104319642.7	139859914.4	47656048.23	27552886.27	35259778.37	24432344.63	112565578.9	33439309.62	45709966.19	79576396.28
0	1165891.864	1159479.667	2513291.941	178984.1468	4732558.257	1361806.176	1165891.864	515012.1569	716025.8302	363575.9833	672173.0567	774854.6663	1026165.907
32196503.73	11527479.62	22205914.03	48167719.51	13229236.17	31579458.31	13610590.09	14726764.83	13519149.42	14707299.35	30819926.56	22166363.7	20470394.45	16840065.47
5308155.421	1691228.501	2453415.3	5885082.181	3683658.973	7484570.137	3619163.494	1990484.456	1739879.953	2656281.526	3390665.874	2132067.708	4900240.133	1954060.216
3352803.378	2291761.942	1891939.18	14321797.99	16822597.67	30786496.3	5624405.626	19190013.36	6130658.853	6374928.251	10009953.85	5313606.293	13534191.5	2036468.474
10436921.63	6957465.937	10137232.85	70477161.5	6688775.003	10623481.18	7049998.84	6291641.239	4443060.421	4103248.928	8465770.837	5191241.406	7208957.904	8352671.142
13900304.19	21574235.69	32761553.93	21697180.75	21770407.04	25893864.67	31939467.88	28709576.5	19367175.46	25946815.26	19206102.82	21841809.55	21338343.55	30111322.26
20397325.07	27332755	41414769.97	22247045.2	24995232.13	30855420.61	41976883.51	45717529.7	28233918.46	36316627.4	32675826.32	26811266.36	31174021.72	32873472.51
28345271.78	63253332.41	104547251.1	20736475.15	52544569.67	79715174.84	35843947.24	118269472.5	71755483.99	84432896.18	72871504.23	80048685.64	68068132.18	82255820.63
49074356.71	56591539.38	38077901.81	42521725.19	87603683.97	31509296.12	121862333.9	54150269.47	78153952.25	65688008.62	87621030.62	45254692.02	74629010.72	57751820.01
1940866.024	1408435.579	1138645.905	8742741.489	9270252.548	2496457.241	2621414.798	4569045.733	2962059.736	8314349.319	4408030.456	1391712.756	3111726.303	1038408.97
9611064.839	6324318.719	9721631.498	12592890.1	12819180.76	13708249.63	9935581.112	9976199.117	8856403.399	8479460.809	13625900.5	7303420.702	10932546.03	8901448.29
8398365.209	2554265.529	3686769.492	13170064.35	4350994.015	5108100.354	3654100.364	5643656.753	1882589.646	5948228.815	7824810.141	3415526.791	3314627.423	3485884.045
2909344.668	201982862.1	59671151.78	366970729.4	320930804.2	318929066.2	272755050.6	246452454.3	310071933.6	294130935	442941881.8	253307575.5	168572887.8	270791351.7
481437991.1	160055644.2	251157826.7	343107875.7	285762594.7	313577861.5	252820559.5	281474563	327896639.5	215242644.4	206312527.9	194462827.1	119161710.9	142827989.2
52347903.24	69219827.42	84822088.72	99466523.72	101741563.9	95364703.55	77110622.89	83886755.51	68588071.14	97471761.01	147437028.2	95314865.93	109875091	73836588.68
14056479	4923272.157	18861738.45	11882320.11	5030682.91	7118129.342	6937390.836	7900569.347	6811569.541	10091561.58	9709123.604	17310701.43	9981563.116	9631508.823
922182.1731	2464878.265	5273527.862	13397616.08	0	8230122.741	5177925.98	0	5388910.109	3165805.349	0	0	4035121.395	0
9104659.192	16093197.61	21984024.27	28722578.88	20899045.63	40505931.59	19132778.85	13891788.96	18976715.82	17851404.36	12459706.88	13373389.69	12646228.65	15425881.24
248719.7226	430163.4598	561271.7073	3397141.632	614297.0929	1707249.849	780539.737	770167.5057	863154.9493	717704.9483	1407125.499	800853.2941	759209.9288	0
5793826.284	6410972.471	7286410.947	14544462.29	1809855.253	5471270.404	3956880.481	3785896.912	5807478.305	3572884.462	6964214.063	4269744.901	5651969.177	7378094.308
10711665.5	10798497.79	17908483.91	28837283.08	11140496.82	18854538.57	21321999.01	17011446.98	17825585.91	18719516.16	16083210.24	17348295.92	7988882.082	17501605.18
139056.3086	0	1057830.77	1147759.133	0	0	0	0	0	0	345204.0169	0	0	0
172740430.9	131050160.1	265586659.3	549116214.9	325117941.6	326035257.8	422253332.5	576492125.3	262144694.8	376436592.3	247123071.6	459232260	234934084.6	192860161.2
0	5568468.869	8393510.4	0	5975212.538	6467024.977	5419734.639	2048126.577	69986.91857	0	9301898.135	2386944.767	897259.972	9766041.905
1272991.935	0	0	377734.148	363746.2911	473691.3556	1645967.539	1218705.226	270644.4763	593861.3347	1488813.407	1003729.63	818342.9484	1826165.703
293081.5172	0	0	349618.7108	0	0	0	0	10028.59482	10497.1226	0	56916.06362	0	0
2511572.522	5427846.121	5557943.753	3646768.713	5887968.169	3837145.008	7247043.552	10502736.71	7788745.51	9671938.626	4689690.115	5628360.127	3572567.571	10133533.78
80713452.71	48450968.5	127026136.7	87832176.1	62196038.95	116172485.9	75614544.9	73678830.52	79435170.6	81298916.36	87985318.29	37602426.86	101501951.6	120003032.9
0	61627.1224	388187.7738	83477.66055	195733.9968	319770.3571	143174.7398	142467.2397	73206.76683	331360.4593	73554.44051	156094.8685	296448.8733	283268.6436
216917.2774	708083.1473	591335.065	297841.7082	140813.1756	106671.9332	1415179.578	1588653.941	213509.0712	956277.0919	743756.0946	323766.0385	561258.1264	1197383.009
0	183727.6587	0	430968.6216	149166.3519	1591647.818	196012.6365	152727.154	178852.2367	0	89410.73584	105320.3605	0	226574.5366
27106550.62	39674077.35	23278764.37	31623871.67	15830270.23	74651347.56	19751602.41	12694830.64	26384089.21	0	45212775.46	0	32115758.49	68993791.28
1405534.793	647111.5865	5578881.156	20161044.06	4811913.567	3804853.97	1290442.147	1381671.194	5261538.896	5440130.791	6603791.36	5949673.72	3551345.474	14113014.32
17459626.99	15202487.59	13019369.8	11946583.7	5689505.772	28014496.7	7947328.674	8328012.711	15074555.88	0	0	22629800.62	11513623.63	12990197.52
175395793.5	60169882.11	77299803.33	86378216.2	113131863	102718160.3	58986280.42	39403810.06	51270979.46	68752294.47	28735196.19	34679881.33	20313247.6	43217711.75
211970736.4	403386197.1	511068982.4	424191705.5	411282757.3	391047239.6	643601457	1049337575	637184842.9	543395244.9	472835159.5	369145261.1	671685920.8	503871030.7
1132942.155	1219458.309	1827312.347	5697535.236	1523684.669	4737785.941	720066.8913	1606474.432	445806.6491	1245708.012	2011334.22	1307581.891	1206626.784	810302.3519
133881712.3	64298098.65	121051349.2	315594104.8	73905610.48	141201816.7	78565188.24	92422815.79	77134348.84	68512843.17	90068036.71	49786353.35	73490644.58	85890790.13
60165074.24	7696296.069	35264453.46	17487482.14	13888725.56	25370769.41	34554997.57	2378460.953	13782727.36	16317367.45	2154934.627	5689102.469	3497134.265	0
25573945.62	5205502.09	6695911.65	19744528.88	4928544.062	12592248.22	7578864.175	6852761.2	15895986.65	2298408.055	4390255.142	4533230.618	3586755.777	4014042.166
1564783.605	6391541.42	9344246.205	11403726.26	11989969.03	8634110.207	6399466.759	5076577.448	8030273.044	6962289.426	7127779.504	7420381.944	11288083.16	7398108.857
246702118.3	299117461.2	431749167.5	523354459.9	340230178.2	358309500.7	348721543.3	443829601.7	254866342.9	340485619.1	315842484.6	332752482.9	344117381.8	369909724
63305102.3	159032601.8	106844980.2	96210050.74	124345087.4	98706482.75	116848669.4	128909031.8	117636185.3	108619271.2	67916756.97	111468635.4	111357827.1	143567369.6
75854286.89	163475505.3	145592310.6	180171629	164367676.8	170448951.9	127241303.5	181547058	170901445.6	149561491.7	115424433.5	129397575.1	159793172.6	114611230.7
0	0	482053.912	118545.9954	0	511580.1093	0	121202.3316	579382.2299	326683.2047	318972.8959	212076.4311	92917.60505	306153.2846
111017064.9	106843436.5	112803968.8	202450653.7	106121585.3	253329630.1	112534704.4	91096705.14	106887397.3	112177204.9	91820480.03	60883082.96	85362144.55	71139025.78

[illegible]

[illegible]	107796.0508	35385.88321	642714.2575	622498.4974	98226.21351	992349.2744	1388233.891	206318.665	1829537.142	114064.7634	109801.9745	1570203.641	1306734.766	105014.5799	1728296.794	1927349.999	2483336.414	2327553.142	1871747.606	187822.856	711369.9884
[illegible]	8264197.783	9602605.225	4772833.101	8857318.18	13672500.77	21386162.75	16570244.97	12413177.24	9592999.946	11306299.96	18725472.55	13174705.32	10316045.44	15479154.28	14868403.81	10799631.37	11737601.69	18947594.06	9032752.867	13146483.65	11392944.9
[illegible]	15070407.25	15884571.69	27751281.4	37327020.45	23142941.46	36557679.04	21795777.46	22397328.71	19014153.88	20979511.84	22689262.91	19541739.44	26279577.76	19411272.24	30392380.16	31958169.6	35753497.78	18597385.88	31284207.78	25437597.31	18599257.62
[illegible]	8927595.241	13867315.59	17174542.81	30997556.78	14703996.49	34715677.77	15064211.52	15178399.48	12257598.58	15124109.98	9961105.446	10440148.4	12665910.44	14624358.25	13704981.13	13162093.76	15787415.6	10459882.74	16459333.91	13755318.45	16429739.55
[illegible]	88364407.4	46468220.76	44482043.64	79426127.85	57156022.31	55402413.78	50269864.71	34994450.09	52661980.29	34448331.64	74331715.59	68061132.26	78161336.47	43504326.74	133382989.9	119494424.9	93911700.23	169292479.3	57975871.69	122285703.6	71120942.34
0	0	154322.2184	577029.2893	279545.2044	43951.72954	595954.7561	479792.0033	170012.3839	235931.6171	609831.8648	285242.139	238436.4806	299988.4316	0	345693.9414	668506.5556	525541.8767	195104.0982	503906.4264	107221.706	65647.40848
0	0	0	575731.6848	4483015.823	0	2786420.194	1144844.91	1043827.468	0	824163.9837	712566.7566	588712.1541	0	558664.3979	2103475.324	1390441.405	2744903.615	0	0	1027431.709	497476.5005
[illegible]	8370929.571	9722268.79	5953215.877	15833250.05	21727952.13	91359072.57	12327284.26	12925118.11	17463939.32	18104857.94	14500692.61	10769357.66	11908894.97	11059153.54	16057168.19	9604421.81	18937638.65	16313272.01	23494153.69	16073728.3	15104459.24
[illegible]	20765268.33	30185655.1	35758953.48	65117008.51	35035740.74	42974216.96	40881783.08	35238357.07	21483215.78	42700466.99	26124992.63	24391749.97	31109489.4	37907914.45	43718322.66	37917377.06	46720559.22	34786142.64	40100217.13	36467579.38	21604609.63
0	351619.9481	0	2772829.879	3271173.07	1733312.326	0	0	2778855.81	0	4014532.698	934968.3401	2042244.895	704968.0722	0	4511983.384	0	0	15917705.09	0	3353155.897	2389812.752
0	0	364075.2524	491159.8181	5241985.223	501524.4173	101563.4563	516274.2805	189282.6925	0	600235.3046	166325.9787	401406.1227	276341.1285	269407.149	10454474.29	879383.725	486193.7861	420852.4376	7628081.628	843545.0059	181608.6267
0	0	75782.32342	147679.5086	87898.77983	234772.4532	22296.31076	134291.4385	82758.11688	208769.6781	113267.8534	259950.5723	23445.06011	216536.3488	0	354547.1386	147127.1342	227347.8851	0	0	117069.4992	736756.8091
[illegible]	85589.1485	111819.6228	1232276.615	14169676.62	193127.8639	2789110.826	347197.8234	0	0	53752.35096	26014.06608	52896.59682	0	0	1206230.748	484800.853	2524277.811	293017.6066	724926.3067	189281.7929	0
[illegible]	61862.63308	0	0	0	32737.31071	0	0	0	0	0	118726.3194	336748.4596	50375.78772	0	298374.4526	98520.15012	417937.8643	104051.4549	59624.86357	71343.53937	0
[illegible]	64656259.49	78180223.84	86907507.24	125370093	114215185.9	114761110	94482066	84476381.26	72480672.49	99103131.24	71873995.62	82099645.08	86921965.71	79057305.5	107942240.4	87425619.46	89177201.16	83997336.28	95908094.12	95771835.22	77812409.42
[illegible]	155366717.6	123146953	150917625.4	257393284.4	190940756.5	201240201.1	159297334.1	136181029.7	109908791.1	153373188.2	160330212.9	140488166.2	167307346.9	96392878.15	120643777.1	167345824.4	130983814.9	123856366.3	163743940.9	153662757.4	167340424.9
[illegible]	1644599.144	910686.494	2044332.71	5391770.394	1114887.046	1197834.312	1939709.976	1609983.179	1413859.765	1851225.696	1754681.198	1021669.172	1899292.917	1734929.866	1529092.247	1670035.641	1647281.867	1358086.762	2502068.742	1387922.065	2806070.335
[illegible]	4863151.149	1866837.628	6953564.449	19111053.82	10227468.9	3445661.128	1493974.441	4334518.129	4608529.786	12862067.83	9217517.778	9722723.2	4616459.034	2449601.751	1929841.617	4584162.021	10916989.26	979873.6345	8078317.267	12780990.19	16997948.52
0	0	0	0	0	0	282893.8718	0	0	467279.6057	735132.7855	0	0	0	616129.9097	899610.9695	864718.7064	0	0	709887.2086	798072.9842	0
[illegible]	2228498.923	1585641.242	2918657.019	1898202.271	3595374.477	3299845.613	1711954.454	2798303.473	1896498.728	1456772.841	1984071.991	757912.1384	985054.2231	2677322.38	2906856.573	2673477.524	3636548.813	1941848.407	1082818.868	1994726.893	3154108.935
[illegible]	23827576.05	21745594.91	19649648.25	30874322.18	36384125.88	29175008.84	41878755.19	33593643.31	27407548.83	22591806.92	16879071.92	12484553.78	17220884.96	59021038.63	30963040.2	34258556.02	46621156.12	39222125.36	25829340.4	20020709.6	44096636.85
[illegible]	60971686.76	79835519.92	63927346.82	34289460.69	65367853.68	39886885.96	89156187.52	82229321.44	47473586.16	57887981.61	40866971.11	43454063.87	38290538.85	94269416.28	89143262.16	50761827.52	50850316.02	59723877.34	50874322.82	26014760.09	50349192.62

[illegible]

13726118.94	14190909.85	16893359.01	12978781.69	13577085.16	26386405.72	18174742.16	19157637.37	16736530.75	13438675.35	7776145.822	13993596.5	9341738.678	11527780.5	26924956.63	34877895.5	22352829.91	12964495.81	[illegible]
360700054.4	576753354.9	429688637.2	553988107	652114167.1	559398611.6	386779874.8	331665883.2	482545043.5	690075932.5	425381907.5	886403018.9	510752849.5	659792983.1	452092363.7	517310135.2	548423633.3	683622733.6	[illegible]
1200311.007	12498762.62	66365987.64	2583357.488	14513622.21	3128868.759	2068854.131	2623821.062	4552362.468	5560183.695	13265357.7	1223280.623	3126933.506	7993123.989	4542367.66	19186360.75	24025929.66	11799337.88	[illegible]
6590916.288	19907222.97	8776376.563	8513550.85	212494756.9	7173422.069	7068601.322	7561496.875	5513138.613	3206088.474	1969488.786	8479205.677	8205657.52	10319209.03	8946296.414	16028167.22	6962759.772	6932368.799	[illegible]
185493.3073	243382.2479	478590.6797	208088.3538	283877.4859	130160.9797	145908.9263	76741.78199	146713.1362	155045.2577	116399.6025	227264.4857	204691.1235	190514.2562	839002.7993	864739.6842	206852.4082	880590.4738	[illegible]
0	376678.4998	313677.014	0	1576032.389	168295.3421	1049811.735	138569.2334	186789.5497	0	0	0	294529.82	411007.6124	367543.4459	232862.2667	0	37368.56912	
73280.90612	33783.9404	39994.06359	171927.088	263293.4668	59462.3406	168941.2402	171049.5951	264596.6916	162297.412	146940.5145	110369.8333	118829.3729	112614.6361	15826.75415	134359.1417	57741.38742	136544.7145	
25728.86408	29719.36455	361066.4105	171383.5225	918429.7573	473091.8141	109138.7689	60928.91984	32435.44861	0	42197.48291	85209.97438	0	383565.9966	290968.3447	124320.9909	131215.5792	195150.7466	[illegible]
0	0	1563200.784	0	0	1438690.24	299941.337	888467.4402	239640.234	0	0	0	1070646.396	673099.3053	0	168684.1562	0	0	[illegible]
24135726.93	24379215.21	38498966.41	16159862.91	25074328.62	23336839.54	24358795.88	35179914.29	23689517.02	26293836.25	20043856.06	12324513.2	18942712.01	8483430.872	19775095.14	22485300.66	16503380.14	16200318.48	[illegible]
289170598.7	299670939.6	174476545.6	240612453.3	206680927.5	314724733	350724890.8	250004199.5	300390176.2	252071760.5	316956939.4	331670287.2	286917671.8	392738318.8	259736640.9	339467068.5	353323185.7	342034993.1	[illegible]
70915877.38	74158088.55	106123463	95765065.8	132610464.7	96253192.82	81961078.52	56780511.33	74041284.69	70913245.75	76005945.93	89253734.78	87093210.65	82945522.04	60724968.62	48684769.36	70229051.33	80645303.41	[illegible]
813886.6113	327507.427	1148966.45	1717392.367	1654892.535	2136886.481	1506002.669	2871298.341	860195.0804	1038248.673	1172946.769	1162888.092	655763.6324	830334.4382	1318462.12	1024720.046	1126233.017	1210892.713	[illegible]
435405.0528	796368.6268	435907.1618	36739.33284	891509.2299	488661.4121	85920.29644	0	0	0	0	458441.8706	725987.3832	245651.8401	0	640557.8187	620382.7426	160167.4137	[illegible]
3177678.823	5572193.764	4523624.467	5011485.579	6320973.632	4911535.228	1753159.424	2929552.104	3341596.806	3504173.81	4521692.475	4807022.04	5385910.664	3789622.383	4421158.645	3079621.185	5693862.341	3901929.44	[illegible]
247510.7866	328373.5707	910109.0222	324822.6242	249155.9968	330775.6504	439136.0486	585599.0112	123142.5428	352323.0686	188166.5757	0	180238.2648	1214572.849	149998.9015	1261615.677	0	127672.5087	[illegible]
224149437.9	413193061.1	246790837.6	263946443.9	273341155.4	361555506.1	296460719.7	252366223.7	236599810.4	204648599.7	184682725.8	276172769	332358355	249221699.7	262100082.7	242488476.5	326387277.6	361818729.2	[illegible]
1475485.137	2447826.736	2411877.72	7577777.171	4717561.454	4504682.957	1511029.19	5173050.74	2893074.082	8720001.689	7799069.908	5867704.682	2925136.044	10899558.94	9856840.302	12046967.15	3834069.264	17072394.22	[illegible]
27070857.79	28981820.1	36478990.58	20437798.7	31899672.62	30276103.1	36962687.13	32665769.94	31383937.03	28274263.75	25500428.51	27405711.85	37392440.53	55953058.64	34333972.53	44342223.68	29462017.13	29403372.65	[illegible]
50375007.11	41919564.51	56734508.32	67712376.17	51302270.85	57385098.55	72268102.06	47349487.91	42476123.76	58049012.56	59774544.79	68271865.69	54731852.43	74420346.92	54903476.87	57180143.3	47757324.22	57535042.04	[illegible]
34139961.52	37705075.55	46826684.66	40357203.9	39722796.23	34473731.16	40478784.02	48503997.6	36400486.74	39178390.31	43921788.99	56837629.86	37028143.05	65301502.87	41299236.6	53029531.48	54329021.35	36554428.48	[illegible]
20422798.72	22680552.8	22602872.84	29819643.21	35355705.77	24384309.69	25013113.79	23718197.81	22143650.63	25587088.83	34002136	34902393.54	27980974.47	39607917.14	31295432.74	28346733.71	28668637.92	18790134.32	[illegible]
1806443.912	2428305.342	1958695.865	1856818.017	4660178.269	0	0	0	0	971731.398	0	0	0	0	0	6080060.482	1113234.623	1967564.754	[illegible]
0	382403.2134	602189.1441	0	0	0	0	0	0	0	0	0	0	0	10642.41952	726272.6134	0	56027.49558	
23449254.14	30515486.08	72357062.2	71145785.76	62438656.85	44527635.61	45255413.13	37976648.62	58550602.31	43202243.14	58386938.21	47119775.66	29204119.52	50208738.64	39982444.28	51463810.36	63257513.84	43807252.3	[illegible]
1462912.829	3327708.523	4810828.418	3040801.689	5217256.861	2449638.933	3927004.888	2275403.314	2301133.183	1432518.999	2314873.766	3037086.323	3880883.514	3432333.049	3615719.318	2907024.079	2468668.851	3717985.744	[illegible]
0	715473.9036	799463.8636	556146.5223	0	0	1530314.109	1306318.89	913322.0725	0	0	1308181.246	10055.11297	1124222.235	1638425.369	0	691979.3167	2209496.796	[illegible]
0	0	404444.8603	30654.76364	53831.42932	165761.8868	665542.5011	338561.7493	427237.9834	0	0	0	331356.8105	783537.9374	605895.8809	925694.7152	399610.1502	977171.4892	
4903004.636	6760788.804	11692430.41	6038461.427	17385771.36	6168668.868	8202090.69	6247471.043	10570346.87	7379585.785	5011912.406	7276653.528	10030967.74	11873244.99	8795419.688	8844560.369	4422532.457	8335331.104	[illegible]
34587299.13	39338196.28	72590371.4	41686728.52	50480895.68	50667767.37	40169920.89	53378670.72	35953820.87	50286760.06	46859433.2	51971729.54	33923286.05	66690614.86	87125249.49	49831248.65	51976042.08	55495944.32	[illegible]
43584923.4	48867744.06	54361651.32	51775404.99	65226048.46	69699785.5	62241827.08	57267926.29	76755932.44	63399499.74	64160781.92	52227929.59	47792790.64	85723961.81	82514466.94	57320854.91	82687389.16	61203817.9	[illegible]
9900492.419	4251410.603	68514437.08	10617275.3	34433997.61	20403050.86	6972887.338	8883313.496	9185050.73	13899491.32	10753152.74	17161015.3	3692112.514	37170292.69	21397419.83	58469995.85	42073399.79	27692771.32	[illegible]
1845927.171	2298762.882	3227850.932	2520724.042	6620104.887	1155522.199	1927546.309	2091682.496	1209825.773	1773375.713	2424861.473	3246025.74	2531620.12	3240249.424	3505205.248	2615192.762	2921430.646	2632961.979	[illegible]
10182155.9	4736037.806	5899429.736	11358468.79	9821720.53	3008485.279	4730046.851	13002731.89	25165543.66	6631485.224	15176482.97	14104437.82	5399054.852	25504449.56	12437143.04	27086561.24	20912198.41	13558548.34	[illegible]
22120296.38	36813225.64	56015288.56	38590679.09	94041765.59	34948073.18	34278190.04	28355047.69	37516448.94	33606034.45	27054152.26	36708598.16	31456090.83	39520686.89	34110804.03	47352880.53	28847790.48	38321496.27	[illegible]
9982740.13	5701265.316	9448749.091	11156947.53	12261583.97	10204737.96	9051017.497	9789873.128	6473732.424	9105203.412	10666436.12	13490767.25	12320484.99	12939421.68	10132976.46	7169386.753	10474701.65	11166154.95	[illegible]
6200815.986	10807215.04	9794695.944	6974742.494	10227117.67	6663916.926	7592673.276	7299777.013	9712722.969	5582536.816	10567456.45	8293523.44	6563858.166	9363239.714	8866126.362	11425276.39	8109204.717	12557213.09	[illegible]
[illegible]	[illegible]	[illegible]	[illegible]	[illegible]	[illegible]	[illegible]	[illegible]	[illegible]	[illegible]	[illegible]	[illegible]	[illegible]	[illegible]	[illegible]	[illegible]	[illegible]	[illegible]	[illegible]

649157.1892	1655497.927	1088247.727	859674.7354	2234741.089	2371151.11	1200138.458	1946130.207	1770431.176	1329982.177	1717181.513	322985.7533	757073.8625	601665.7078	1166571.855	3692097.638	2097606.187	2730287.252	2563748.903	1983449.904	686434.6351	687691.5536
0	1418727.291	767870.2211	1531283.217	1197955.069	2553312.669	2078530.356	1522961.41	1035488.689	620360.2232	1175438.367	732449.2136	491893.5038	723179.9506	1677964.051	1157496.742	945792.1493	1657063.866	268522.1213	1168444.61	275294.6056	381920.1945
1086993.343	386036.7791	690324.9749	882144.855	920403.2605	756470.8781	1091470.894	848788.9921	881370.7376	929205.7744	846435.0394	284418.5426	0	394797.2904	1170302.215	756955.0897	654749.2976	923794.222	233874.4881	1231262.861	1121654.967	1482004.006
0	573642.0239	371075.0322	0	683585.0504	463166.8303	6811052.256	381885.9118	908236.2635	430421.6317	2347866.083	613150.982	2698413.784	1443465.572	443873.1249	0	1017884.811	0	0	0	3737858.832	0
2687870.703	1360538.258	4004794.041	4069448.209	4761915.171	5446287.317	4792165.444	3132426.202	1845518.298	2828849.589	2599874.463	1597567.902	2054869.585	1801229.626	3432094.851	4351898.424	4144766.671	4991177.8	4541704.929	3950950.464	1836591.943	1406474.66
2220311.23	2149802.972	4440165.309	5423232.159	14906018.33	7302971.311	15798736.8	5771844.474	5056852.869	5990123.42	5615990.106	4843437.475	3036582.185	7557588.676	5079042.007	8579653.927	6836330.116	6275876.06	6279324.513	7300462.697	4948263.328	4394584.163
1721340.479	1752356.557	2852755.171	3831928.062	5956630.236	4241286.718	6062734.804	3623810.847	4820523.193	3866306.83	3994253.204	3637592.766	3110922.111	1996836.132	3952020.269	5508948.733	6489438.238	4547346.583	4407166.919	4776440.193	2529359.315	3092956.071
13275722.52	22763192.48	18070516.26	28125188.5	26411109.37	31912213.36	30532695.13	24879967.89	16571117.51	21234246.65	22815525.08	18084051.77	16709359.58	20191799.92	18026437.59	23750095.33	21617400.79	27965807.99	14152681.17	24743345.31	19626512.71	31418026.98
30986.47161	0	76884.1722	0	190808.5665	0	28760.87261	0	120995.2472	108823.0262	86382.72696	135442.7029	0	111392.8051	0	0	0	0	269865.878	109186.6342	84790.82802	0
5046394.891	5267315.427	5590915.988	5778164.145	9177816.02	3331035.09	2728731.679	4353278.11	4760685.004	1892036.865	2125768.241	1996658.855	2119481.529	1379403.434	7804507.955	4937870.448	3087252.149	6692100.951	3381262.734	5403713.231	1148580.784	1302234.813
89080942.57	56316366.27	71896898.13	70061187.65	76918447.14	62561898.76	89892599.96	80955068.86	59633346.65	87358782.19	86587307.16	38134241.8	68964435.58	67680524.53	63467693.43	64477801.07	113362296.3	60867227.15	62400703.64	69822761.23	77902417.21	70879372.09
228472.4443	122125.8171	7871278.623	0	4632821.827	4745586.732	9111050.978	4948046.361	5416992.183	4288264.016	5578315.266	498688.5181	4113300.201	5400297.998	440642.0242	4541626.837	5450494.932	8274762.21	3429407.983	6745059.382	187512.014	5346254.689
12211676.67	15800209.02	7859666.429	11181261.65	2985177.683	2146999.663	3215733.817	3832982.509	3502049.129	5446079.14	2833033.391	5731789.921	989847.1066	3161756.202	9211675.771	2433263.85	3619973.859	4347361.083	520948.0023	3955646.536	14378541.83	4306454.304
10561900.14	16460064.15	6075470.459	10885090.68	28107035.7	24733264.67	40143176.71	13756314.75	19016062.1	8052431.556	13218119.06	7046101.493	11948574.26	16950244.7	6086636.258	24835588.5	16416784.59	28677731.39	10519151.43	16911186.77	21358573.06	9219607.358
177647.092	142037.4837	1175440.86	543677.9338	1331583.601	1623598.109	1062877.799	721691.5317	1120170.544	714199.6783	1124460.792	986959.9105	596851.5746	1095389.279	1081412.214	1882967.536	2287714.118	2759451.937	1323365.392	1975403.028	291456.2508	370630.5678
824594.7822	98193.51021	0	0	304672.2898	223247.8498	196518.0306	29182.81892	0	0	0	883671.6488	0	85376.52184	34437.06161	1123091.027	151516.9608	110139.4258	147094.1083	102652.476	0	0
171037.1934	71865.07607	540686.4581	918233.2598	650844.7655	747127.7525	675524.251	535162.426	712362.5496	1031471.352	820126.0898	315928.555	217342.0262	764492.0033	488910.5952	1087391.401	1126237.173	1309000.849	930416.7502	1364991.933	376271.736	745733.0239
84529561.99	87175441.89	49406331.15	43821336.59	18982753.07	51051334.84	27141214.56	45905020.14	257957308.2	39965328.65	51714495.72	48933537.95	38503207.25	61723587.57	105274300.9	87666156.26	80470541.51	27852271.59	68821867.16	16046132.06	15753258.23	34926946.21
50557.11809	516843.261	108129.2381	341014.4749	1314720.281	260572.2334	207436.5135	0	133774.8545	225016.0868	449192.3715	386369.9497	614202.0859	769587.2601	35609.13418	707898.6184	615731.0649	1414423.296	1205273.734	973079.0678	761678.4463	132196.5824
0	0	0	40047.97081	0	0	44854.00566	72144.80652	24778.95809	31033.30255	63623.62718	780418.4677	0	26976.63022	105188.5742	21715.58603	0	24340.96189	13422.82112	0	0	0
0	0	30746.17722	0	64942.7492	512379.4627	166551.8882	0	85424.95852	15956.24403	18087.27408	0	76523.03942	59229.83136	34980.52338	127355.8474	57571.83437	36554.20358	144204.2188	91800.01745	0	86095.60902
89131.24166	114416.2555	180096.8357	383395.6387	370425.5103	384122.1526	606631.6205	321189.2174	424074.4322	227246.9271	105273.2016	451102.6828	358752.5457	457308.1507	501000.8598	567845.7268	866704.0458	736925.6451	512975.312	639867.3121	170917.9942	95421.29727
89343.6332	56436.04075	131033.4734	1829286.477	202266.8562	235428.1646	194989.6055	397348.3047	179439.6765	28182.50809	199127.5904	308029.3061	154792.6052	349316.0793	303446.0653	494747.1713	426829.3478	218954.031	579781.025	354613.6845	83162.95751	105643.2735
4026522.717	2068342.902	10973231.07	22637477.29	8345809.74	11919581.44	4686511.758	12829289.36	23121745.76	4476059.05	14945951.96	11827634.05	18629048.32	7736958.757	13275976.52	16303728	14601001.56	32497985.16	14353783.83	26031280.56	9260670.909	29348432.26
1541017.669	2536440.384	1250326.454	1844249.027	2473926.106	2559247.832	1640268.31	1986339.88	1849723.333	1468264.382	1500993.635	1548010.709	1037352.592	1480336.91	2469059.643	1723097.665	1751165.428	1106890.955	1663306.639	2358930.132	1309960.886	1603345.136
0	1510385.44	1038810.095	1691335.342	4249071.14	3258706.358	2632884.94	2144403.984	3031452.886	841487.5821	868223.9252	1614784.18	1585228.225	1445041.311	1795672.638	3219196.731	2322260.206	2429599.166	3649159.157	1673539.584	969644.4133	552051.6483
15492765.1	24938585.46	17342155.73	22862648.98	23355402.81	30026439.86	11005309.07	30973139.52	42842439.38	11580303.56	22633907.93	39068371.31	9780694.138	22085659.7	50906884.16	8379452.783	32972521.28	30085014.97	26875909.77	23066528.72	25950750.85	20076630.18
23527.51953	172651.3417	273267.7244	280821.4667	80386.06198	338353.8161	599652.5398	234881.2823	93014.72685	316552.1486	55422.87364	136143.3017	416176.2114	377309.0145	221660.4236	458590.2416	478040.9804	384343.233	343089.2783	438086.4916	0	0
0	0	0	0	77564.67875	0	529862.6944	134932.5713	0	49947.50872	0	0	0	0	0	0	65051.51095	0	54275.99113	34843.63276	0	0
8144071.378	5180122.897	11896499.78	10902419.21	14442847.73	13207196.2	17413342.9	16316779.25	12284285.28	17362329.12	11953536.41	11396278.63	13243163.09	15527514.15	13436427.03	18513081.15	17599212.49	15014054.38	15943752.78	16378977.75	3125793.333	2914095.867
470664746.3	324954047.8	729503812.9	1197751641	472300771.9	624728851.4	640007628.1	779351856.9	495610404.4	362025031.9	474403725.7	601302482.5	411957950.7	1217674607	965551367.9	497860633.2	600586783.2	586824679.1	637760642.2	726724169.7	565359788.7	444919325.2
44647693.61	46042893.16	50952544.79	59344485.08	83437371.38	73692044.85	96289466.02	62910472.83	30626586.57	29464296.5	32353977.21	32019286.43	38602899.25	45824119.34	7628538.879	43073418.47	82256549.58	19644130.02	59249922.26	686305.7897	65340231.05	46215962.93
0	0	283736.5035	257159.1245	614657.1282	674296.1027	116430.6598	326269.4151	380799.8427	288189.7648	444730.5071	404091.5874	342655.4266	346155.9677	0	539673.0934	473282.263	808668.7168	444768.5433	859078.0013	1035963.883	81535.93467
1301247.341	2657942.211	4635228.69	4394388.486	7920722.034	6854001.911	6794561.799	5068079.536	5207129.265	1856311.584	7010740.802	3900978.843	5255149.019	4148398.675	2718187.333	4463788.306	5636486.899	6145379.019	5336780.049	5043388.121	5860048.196	5398165.155
37251.16263	128258.2242	83761.03846	305654.9756	304309.4938	298234.1929	534298.0226	270287.8644	259531.8911	231268.3285	203038.3375	204707.8789	236996.5477	348420.171	301061.0934	411481.2101	396826.9967	311330.1466	219696.414	412807.6043	133280.212	105440.9934
190639.4594	249866.7259	638257.0236	510615.2039	737886.2722	310670.4326	506827.4566	604183.1899	601575.7838	350203.0107	485820.5739	741853.8123	231122.8318	266056.2904	945712.5304	246828.0632	607960.7121	1445534.849	760868.7393	689025.9993	539145.4003	611051.3503
0	0	0	0	0	101421.0819	0	78707.3534	0	0	0	0	0	0	91141.77937	51774.66856	0	304441.5133	52897.55282	340869.6434	0	0
170093.3177	180404.7527	0	249612.5037	122851.4947	0	0	0	0	0	0	193405.7327	0	0	0	0	252407.4728	76835.92138	0	0	0	0
0	399280.0992	236625.6286	276119.4166	426749.5455	390701.1153	62283.43299	123046.8424	0	0	126626.3612	229515.7045	0	0	0	124265.9951	122120.9712	202774.4033	83674.70414	96921.60902	76776.10512	360526.3325
4638129.153	2707734.625	4757077.702	8907734.894	10484562.98	7505981.864	9215232.104	5831703.422	6308556.933	7582107.721	6607127.587	4130686.587	4324892.929	6307667.051	7348701.055	8813414.625	4963143.506	5896123.824	4391387.29	6965703.053	4315415.598	7492377.266
73044.56937	340366.0786	0	319141.1117	646076.8689	52470.26268	1030498.023	404153.7165	130911.7155	89264.43669	290843.1675	89599.11698	0	67185.9654	67315.59396	368386.6397	252100.8148	774092.1316	102083.141	371733.7478	521146.2817	244144.008
7240801.788	6899439.916	21223727.59	22028168.08	12420694.81	20828511.28	19494276.09	22781567.33	25398333.14	16712415.4	19943130.27	13738743.67	17454984.04	26507234.36	19481443.78	24176270.62	24587023.46	18752481.37	25747668.79	21340737.44	7944406.025	7306555.089
7067196.009	4015414.484	8340725.243	12462236.01	15082801.36	11479196.54	21108374.92	11216499.71	13110282.09	10026559.87	13452812.88	8670063.098	9492066.981	10362051.27	10193577	16555606.21	13790015.82	18517638.85	9862077.361	15696747.45	6028142.177	4874418.939
173012.1952	84000.93964	70779.5069	230919.5259	485306.4734	1367695.863	5072092.966	528411.9855	429655.2176	305135.7725	1470161.72	557611.7458	233841.9256	158213.5758	664977.5694	2400786.142	475898.7868	2647231.685	1433073.557	1630195.606	2658048.662	8371.31029
156515.7444	474106.7415	2577025.218	1012377.221	714346.0168	425081.826	1695471.493	991673.5203	1303291.659	815087.119	941043.9827	1262552.132	714890.6017	1119827.769	1651717.036	1601812.034	2415443.818	2190471.77	1614892.508	757383.3709	1133784.378	1591757.794
319425.731	332702.7777	130324.2332	1004876.296	1428395.019	677123.1863	1431276.58	198042.3855	628854.379	216096.5089	279852.8389	273351.7624	207245.865	247271.615	126097.4647	745507.2436	1053692.079	1607509.018	433027.4915	950918.7826	597938.5999	148430.1573
18748227.27	24751690.56	19628596.09	35105017.53	49307373.6	48618985.03	49824030.64	30205721.88	47708560.43	42376635.64	41635065.29	23583366.84	32854911.19	33327191.9	31744406.13	26145213.64	28442982.27	26733983.29	21400732.8	29829943.5	30371215.71	47625955.38
0	0	0	0	0	0	0	0	0	81858.46459	69845.60576	0	0	0	0	0	65362.89423	183248.7863	0	40102.38458	0	0
0	0	90601.61338	95504.58446	55233.80611	0	280542.1051	75520.59728	103561.1477	99614.60725	137760.2524	21814.79007	58872.62754	0	274947.3829	359212.5261	156233.2146	194904.0948	21427.25897	336487.3336	47337.29714	0
45215.78463	314461.8109	0	27083.53629	446114.3002	93610.91395	136309.1473	86979.61649	0	0	51485.55209	0	0	283843.0786	0	284536.7949	138405.1163	674659.7884	190995.1935	410101.1126	97329.53203	0
5902419.25	10578858.36	25776517.93	25848709.5	18155009.62	26826487.46	25986972.92	21204922.43	27115137.92	27155004.69	29626608.73	14672062.4	10311056.88	5094698.808	29588768.46	11888381.55	15924519.45	21370557.01	19635308.75	15551072.84	9622180.354	16461829.4
34486466.14	64867431.01	29602095.58	63184416.16	63580956.13	54222665.52	50145912.27	35509073.07	60493951.17	28606919.12	41124598	63613586.36	37332282.48	52761269.2	39609835.71	50787464.9	50515966.73	66148926.81	39682042.12	62980400.94	44685287.85	55817859.25
1325520.444	1875604.501	1519935.246	1698954.316	7678134.229	719280.2607	2158201.291	1865827.167	1494044.209	2596047.859	977854.266	1455147.996	1806589.434	1649682.237	1658989.661	1621016.885	951600.2108	1584914.788	1471831.911	1410225.473	2187923.982	4470615.817
9995943.48	11335991.79	9751639.416	11299904.84	14028365.94	11530017.26	15584434.46	13843011.99	17983840.97	16352245.64	12319044.41	12013892.05	13967699.16	14734448.9	15581030.98	13047087.02	12001589.22	9687588.991	9745361.751	13853113.15	12578344.75	11429869.93
778539.5449	592951.8881	794462.8581	1279725.54	2359895.898	1748792.088	1246618.522	1948460.504	1435255.735	2230882.705	1747396.76	1870213.792	1239510.106	1729894.261	1813783.113	1448134.864	3043698.1	943510.357	1182095.905	3597526.332	1375365.5	564225.1374

[illegible]

7423235.873	7332317.028	7515199.858	10774601.63	16469637.72	9475382.752	15965917.1	10517971.55	13592443.03	8107831.24	11465378.34	13057522.27	8625538.503	9656397.692	11766718.62	14635828.42	12970431.45	13623570.76	12716036.02
8845031.941	7672023.896	14504042.51	15784159.36	19302092	17401862.72	22109936.63	18132412.45	20038907.59	12779660.78	18201372.53	17296857.85	14796387.73	19269314.64	12373969.7	15128375.01	17977053.97	15365765.3	15534609.69
3176945.68	4582070.291	1729741.259	3621309.54	5151598.057	4293499.982	7092717.732	4785797.575	5438097.063	7277501.244	5054278.057	9203053.618	4446335.52	4523347.379	3175365.869	12200061.65	4401011.615	8479496.841	6761099.485
0	0	56443.51829	0	94419.99389	39436.86266	45712.06222	53447.51922	115532.556	63582.4797	66814.27853	0	55246.82485	0	287028.9464	147403.7116	112868.094	65551.88553	70929.06829
4673259.99	6311255.305	14359854.3	20177048.91	23965553.52	17145764.24	21840484.72	17095259.12	22357400.65	20521166.49	20744836.07	29220437.9	23413841.11	23222703.26	18273689.56	35231134.49	19258927.11	23219009.33	24539221.58
1177716.361	1107529.318	1757681.321	2546350.589	1582761.453	2838892.719	1757732.668	2381442.349	2611040.497	3296257.127	2943508.384	1108185.426	1623606.25	1657205.311	2310831.5	3379654.918	3012635.253	2850815.968	2279234.847
0	0	0	51298.57968	161062.099	0	143334.5255	17641.29454	76092.82209	0	0	0	0	0	9592.023722	17142.54014	0	16668.42933	0
1655575.488	3329227.574	7314744.733	12258497.33	6443678.9	2656235.339	6575211.34	8679339.449	6454090.577	3350117.53	3974668.039	5393959.846	4800229.753	3564079.819	5870289.796	2530492.617	5537017.724	2835931.195	3795442.844
4007852.877	2696205.02	8834009.274	10418686.26	9881255.175	8350699.71	10121142.21	6338312.241	6300072.857	7218321.102	5752471.166	6298843.97	7389632.398	8819714.551	6253962.969	10856178.66	7597928.409	7082563.887	7330914.771
3948886.883	3975083.251	2434311.563	4615398.235	5224202.71	5273906.68	6542520.482	5433421.978	5771366.743	6097302.512	4581352.272	6648207.032	6060690.913	7462757.124	5489077.701	6467054.059	5908918.596	4598307.707	5737651.059
61679165.19	33423795.71	30956727.9	22954165.8	68726749.7	47983888.53	138386101.9	28583350.63	18612514.94	40906410.8	33497287.54	19923891.32	17725439.3	23653712.98	27313530.81	54621444.91	44966035.87	28787708.16	44848493.13
1993003.21	7849231.551	694636.3638	3236240.599	2592369.54	2619435.701	3482891.851	3375774.51	2569257.493	778540.4155	0	2366621.33	1276864.612	2849868.31	3943208.524	5358208.219	2874498.375	4205965.829	2373689.416
3954821.635	5134218.566	12403768.58	12221993.3	18891429.2	14408826.87	24146417.21	14215006.34	14678421.63	11391642.83	11583802.66	15589374	14205527	14388267.25	13416211.67	19833938.35	18184267.9	15232593.33	15388106.82
0	0	0	0	0	0	0	0	0	0	0	291079.6436	36315.25283	0	0	379589.4159	311609.2375	453786.6253	631665.9701
0	0	105487.424	509458.9456	781499.5542	313624.065	221019.906	450676.3153	237947.3849	864153.4543	959634.2606	463965.6213	605832.8881	540856.3285	717135.4637	627406.2865	657558.2873	1183019.953	478038.2689
3162609.297	3992106.517	8141105.967	7028288.483	8147053.131	5397079.854	7713597.337	7265384.088	6627228.121	5409745.087	6007289.767	8568277.775	6550406.147	5682575.548	5168003.215	11748751.95	9719247.74	7701071.036	7089607.123
3889256.694	4400036.751	10655994.61	8522564.185	16920311.5	9556203.414	11841343.67	7457103.678	8573551.139	6088067.481	4030149.779	4670373.471	7086844.417	6250033.569	7388231.765	12680883.8	8842305.006	8899803.365	9527674.982
6323086.549	2403821.732	10079281.52	8348979.175	8515530.092	13974741.62	8549147.154	10219335.77	13614694.31	8509036.943	9545037.833	7159040.398	7661993.885	7846756.594	12629538.43	13290520.75	11742203.89	8639919.808	13070173.16
357612.2231	169174.9974	296013.2444	296178.1142	3494319.344	1194472.402	1458756.183	418312.7387	0	454772.2188	1044441.661	0	408252.4341	598237.1946	0	1466974.439	504285.672	2503306.186	101335.2765
345655.0936	595379.0813	0	0	2619713.539	0	3051798.16	571627.5833	337446.0093	751303.557	499526.9649	525800.8905	249050.0075	0	142431.1742	876305.2898	343158.7264	2922971.813	379186.1136
14192485.33	5882374.975	11707202.85	9814848.969	10098705.83	8258573.716	7566328.921	6854369.454	11724109.63	6764681.065	7752172.215	3218840.824	5713292.928	6572468.644	9434644.53	5849121.684	14948245.29	9457227.974	7524198.389
47647089.77	57811867.59	29816160.99	37790490.74	35616921.41	44565264.42	40686969.86	43038006.46	45015560.79	49023046.58	37150840.86	27878368.29	40159046.24	37625091.94	24810475.97	46675605.48	32828415.83	40973057.84	37249203.2
0	0	9156.457008	30895.16676	0	23195.58797	41806.61167	154616.4106	0	0	0	0	80714.26611	0	40065.62051	22200.02466	45271.09825	35984.87204	80786.06828
453797.0983	722999.6069	1484450.603	667867.9173	765873.5759	1735238.769	1712545.567	1039190.543	1216017.605	1196748.521	1274331.6	1191615.174	1286214.844	1549958.249	1752275.08	2295120.109	1604080.025	1610598.806	1389146.384
0	165833.3726	93199.49055	0	0	0	176441.4376	0	0	16978.80883	0	0	0	0	0	117079.0513	108602.275	163246.543	199611.8248
85213688.83	64207262.96	95080797.2	113880083.5	56576798.84	65176899.71	96987054.3	130040321.1	101504168.7	122214510.9	100350362	60368538.18	86388122.2	67506938.68	97951658.08	72607758.05	88669642.87	102673973.8	89015850.15
11891735.35	9870161.395	28543490.71	14620494.34	10942603.6	9367544.69	13130807.39	25786943.84	34755114.88	29946930.14	22097507.12	13648671.51	16098425.68	24195815.01	35088415.84	28854576.95	30717128.98	9734213.234	26378411.99
29987786.06	41501877.11	108963680.7	103225167	49087015.95	125620214.8	100509764.8	142564775.3	78963761.91	90727822.69	49006790.79	69031621.72	57695144.28	54343939.77	75431609.75	113288297.4	135801204.8	95243232.92	102310354.8
606181721.1	454847642.8	1186765023	759711339.7	292055778.5	725689807.5	563678785.1	878216793	1088044059	1145822789	596385167.4	1321549619	500710357	841308220.8	1236934007	802814300.7	748135964.2	738637306.1	954448240.3
1749891.538	996121.6735	739870.7874	1627324.536	5494622.904	2491630.342	4457033.103	1920012.371	1912690.446	3358408.073	1653965.294	905230.1968	1495367.814	2877783.646	2438693.596	3718909.8	2095157.228	2147480.155	2880658.474
18253262.27	18208433.65	23970835.54	36993160.87	31057901.32	31133629.31	41764975.89	28955742.53	24700284.37	21604776.42	26882338.26	27418707.96	30442900.16	34015818.62	23381867.46	39007511.5	35301594.66	31629924.29	22858698.42
0	0	0	0	0	0	84924.15651	584992.9077	195147.7051	0	66407.20841	0	0	47352.69027	257005.5487	0	366779.6335	0	501253.7186
3292558.316	0	0	0	1090245.668	0	678756.2019	149887.4555	0	0	0	0	0	0	638584.9355	543992.854	1088409.366	0	185638.3352
10634000.18	15638140.72	5120531.764	7076282.532	26164618.41	19187094.75	4295996.511	13567576.34	10202345.61	8153503.903	11535384.65	13714927.18	6876800.309	10166682.42	4792412.041	15717727.36	12401639.74	18239068.47	15410372.39
2321276.411	4791688.53	5803863.66	6251191.03	7162276.036	5082781.12	6851296.412	8959431.615	7201517.201	6888170.22	6743532.462	5141704.976	3452112.361	5153908.622	7867303	7064441.129	7874239.864	6947073.25	5427884.623
79118.12061	62926.10038	0	70176.05231	307694.122	91439.21445	1923885.614	0	0	16978.80883	0	0	0	0	0	585768.8472	45935.54477	342780.5608	230524.8587
2834324.345	10294119.23	10016727.5	9625811.29	12217715.24	14042637.77	16694611.56	17035900.48	14065741.48	11444829.21	10000558.1	35823994.91	10990077.28	12900368.25	10293979.91	8196474.579	11838006.84	15762894.78	61885398.85

[illegible]

[illegible]	0	525735.9114	272831.0533	403524.9063	869026.9738	1563105.544	931688.0964	369589.069	1036222.89	315925.6223	1004457.744	255241.1343	794510.7152	1065078.533	1947668.263	1220871.843	350285.9919	1478598.979	223033.4464	244273.288	341884.4503
[illegible]	0	687728.9696	512459.8841	619198.3962	2743268.734	3870457.451	1535193.858	2584049.319	3165981.263	470128.8351	392338.4892	334033.4879	2205788.026	988279.8412	1996332.89	1254670.355	1985924.85	2698724.973	1670072.878	973247.4656	0
0	0	189705.1234	237744.0601	0	496104.6389	543625.9665	703254.0047	1027532.596	1154129.812	0	167740.5949	376614.7099	530056.1535	1041934.494	495722.6275	834634.4162	626696.9561	435099.5129	519897.6381	370259.0456	0
0	0	89240.62586	384898.6374	396269.7997	811532.9337	988429.5198	1334144.449	1267589.423	2247899.303	1003490.842	402029.2782	589482.6469	988446.0939	687070.6712	2350225.219	935075.5647	1507770.608	1248957.424	488785.231	1192953.615	235457.3963
0	0	117090.9932	0	0	282694.2583	479166.6183	658550.161	256398.5436	1237727.751	178076.6268	68230.13634	0	196147.3567	420876.3531	163722.9913	179430.2661	264013.3111	188422.7613	0	0	0
[illegible]	10311345.74	15737730.31	12297276.61	12488232.67	10529638.04	7202183.108	8886059.392	14517411.42	18115671	10247987.38	22598388.16	16803272.39	17813383.6	14790590.35	23833502.02	25112578.08	27358187	25491923.28	27343063.9	10690039.75	10085825.48
0	537430.4585	0	0	0	433339.0733	928360.4385	419344.4619	1021979.426	1282673.708	549141.1437	1314856.247	978294.0671	2175109.711	995678.5927	391959.0152	384300.2546	0	376517.8446	377820.3893	658719.4824	0
0	0	201358.0202	155511.6992	0	0	177369.2666	372942.2008	382313.4796	188800.2302	0	0	0	0	0	646472.2107	0	0	0	0	0	0
0	301337.6677	391920.5584	0	0	368414.2057	0	331999.0854	571698.2302	628646.9128	442530.9809	887896.3003	0	0	1080248.396	1196638.234	746089.8355	583190.7981	90971.19953	497353.2386	0	0
[illegible]	313959.0187	420130.2735	245340.0938	612724.4554	487524.7229	1060720.83	469700.5163	1346297.522	714945.6979	683164.0952	676271.6983	460025.8042	387112.7346	1372451.724	3983895.868	1660902.905	861325.7163	1657664.321	1081695.62	1723746.717	166251.8864
[illegible]	0	0	397413.8446	426293.6897	988998.2283	0	117734.5259	184127.0474	1727355.227	0	1300897.155	454789.0689	520607.6874	603627.7267	62666.00593	309340.4133	961600.0259	500475.7278	445503.6397	300115.4991	943416.905
[illegible]	56259.84291	330917.134	264970.7163	602858.0586	447233.7421	865439.963	448385.1729	0	458191.774	83240.80626	0	307033.4753	406591.2907	477466.6205	698328.882	765991.5986	624007.6341	1798144.879	311235.3391	210223.3961	106486.6482
0	2638957.732	2642657.837	2099608.066	248466.0812	1098270.001	1019665.821	1621609.41	942325.6522	332858.5248	416931.3735	243176.4218	199776.871	259908.6582	1069485.539	992887.833	318510.8236	295968.4783	509570.6287	0	220747.8939	784631.0558
[illegible]	13849871.92	12546306.86	15620005.98	7029791.124	12799604.75	18342333.88	7660789.434	9615814.27	13938694.83	17749442.69	9418900.223	6625026.623	7748408.628	8013528.1	13994181.86	10394886.36	9842673.651	8249186.594	6736464.708	12320217.67	4653580.32
[illegible]	2972507.565	5752891.718	10122541.44	5285133.292	8610399.563	6213844.935	18928717.26	7986162.894	16036861.42	0	7916115.819	6955632.281	9423126.454	30377704.27	33884682.09	18684531.09	28512613.81	22518601.8	33084822.46	7482079.256	14274353.7
0	0	0	0	66274.65677	0	0	0	0	0	0	466362.5155	0	0	0	0	335209.0321	547210.9094	851641.2664	0	0	0
[illegible]	9724896.061	15151342.33	18190156.6	9237817.402	18067779.04	14881863.93	26063602.48	28582594.05	20761536.41	16031478.53	16321346.2	18163051.97	21698109.15	26916273.2	22466831.97	19639057.46	19457683.72	22147118.98	22089797.73	14591665.53	12633266.57
0	0	72212.64425	135190.3719	775900.2054	355882.5224	710861.653	37708.66614	147294.3232	128314.6496	131907.2271	101400.3068	47203.32102	179751.5622	62310.72086	186349.6164	298519.5526	118653.3902	997430.585	436195.055	217094.3185	75966.57776
[illegible]	10206945.39	20162112.27	20874888.21	23229609.3	23900905.07	37679308.4	16719843.84	21561424.58	17494503.31	18931118.07	23084289.43	14356122.92	18847562.92	18481459.88	53457297.5	55293879.98	30265608.38	35405742.83	20486039.14	16252016.57	17157863.95
[illegible]	0	625233.4479	635534.1757	1271218.098	503302.1862	431148.8086	431402.3521	0	231915.3667	328811.8545	0	0	0	1428389.426	1097334.897	725801.2092	1605863.771	692731.4811	493127.2576	408573.14	828316.7568
[illegible]	8358699.6	0	0	0	7281316.877	5490952.888	1082772.309	1688607.989	0	0	0	4704916.65	0	0	2866223.268	3799710.04	1482286.406	6936627.716	0	5529074.98	550546.9667
0	2780645.317	972110.5691	1455382.627	3300356.805	1116202.805	3833059.079	2166288.167	1906425.209	2516439.433	1074555.496	705640.2649	1170410.274	1123566.625	905267.3224	2331415.49	1671792.711	1662335.392	2460468.314	2411044.777	0	1947692.532
0	0	112270.8997	145136.5365	0	98032.31008	174045.6552	0	0	0	0	165792.2835	0	186393.3217	601489.2566	173505.6829	0	162567.6615	588123.5219	0	0	0
[illegible]	68330679.43	81943301.43	75804881.82	102113586.4	79551309.93	74255538.73	66885475.86	200541607.9	37240257.76	44472766.41	68573711	60156853.15	151328206.5	168863704	84946193.04	67100908.97	68417474.51	67282913.96	85814071.62	3121358.566	43497322.22
[illegible]	2206931.173	668082.403	2528456.379	409471.0827	2990385.026	23969632.03	4316325.865	5831617.192	19512872.53	22451315.51	3594152.737	1041067.575	995050.8005	4024364.105	7304908.209	5106835.658	8263999.059	4493805.136	5662973.688	2782460.38	1688524.191
[illegible]	54325362.62	24399660.55	26125875.32	42622907.7	32439774.27	44391201.95	29220072.72	27421895.8	38768966.99	24581566.8	40535304.48	18595568.33	25892421.24	31000488.85	24307437.7	20125416.43	38759692.16	30043319.21	25523898.52	32945412.65	31817370.6
[illegible]	5228396.916	2321014.263	10599648.26	13208213.2	8704708.204	8321757.941	5883742.871	6724427.312	6214025.085	7622596.428	7779152.915	8371596.11	11648598.71	2395138.365	10091719.83	9180095.489	9868085.596	6410057.125	10534881.12	11396161.81	4691808.307
[illegible]	47142776.05	20295815.25	19796553.47	33796089.96	42507832.02	26840427.17	53206068.98	32255195.2	26980402.84	20897143.37	16023036.1	11601197.05	19955165.43	18055933.8	20489465.66	19290301.9	21332107.07	14196814.85	20842170.5	17281111.48	18293569.79
[illegible]	4841392.374	7248158.946	7898296.23	0	0	0	6973577.504	9227410.121	0	295192.9876	0	0	0	8913263.491	9542982.206	13026670.21	8201469.769	0	0	16778166.46	0
0	981943.7623	416873.7802	406311.8845	507745.5316	1326846.833	763499.7416	1358945.527	452652.6045	576642.2676	812266.6044	512767.1129	442686.2137	452373.7478	457182.792	301831.2513	943858.7157	363225.4265	0	758934.9419	799026.4708	294078.6488
[illegible]	61581164.22	33236477.73	49641671.59	62562969.87	56216137.01	52613061.47	95578209.64	148046655.6	57108213.56	86236386.1	54556652.85	123889772.8	60332279.22	99097172.73	51529858.41	37578897.65	119793016.7	20952683.22	35383519.6	70313926.67	89603570.97
0	0	0	0	64331.09201	51150.4241	736551.2327	0	58020.09095	0	0	12527.32615	0	101389.5199	0	178044.6779	157842.9908	115149.9382	105923.8738	0	0	0
[illegible]	0	158909.3162	651641.6514	68937.32519	546972.8919	171829.2584	228548.8962	303942.6238	0	132130.6601	110636.4591	85403.93727	46919.16479	109493.8819	419817.1419	382899.2412	473108.8507	526955.8959	144918.1805	104909.3618	0
[illegible]	0	158633.7503	650921.4834	69073.59833	546374.0502	171589.1087	228867.5059	303151.8683	0	132331.2671	110851.9512	85230.59997	47044.10514	109679.6846	420260.0389	383339.9194	473635.0699	527527.1711	144693.3806	104696.5219	0
0	89542.11616	358489.6164	886948.19	1201557.411	160408.0711	1157715.225	634415.5871	830676.723	689056.4417	952834.1993	703882.5572	162763.7599	365007.8359	587319.985	489000.3253	175179.9148	493936.5447	414195.5513	908047.1311	634990.1126	955996.2926
[illegible]	148096.5448	0	855809.1399	7999885.398	300129.8098	1688590.489	873824.6065	232714.7862	79030.41003	198204.8803	105724.6782	181374.646	129836.3738	177308.3399	1269326.668	461856.0049	966583.5688	533994.7197	434739.5065	580338.3456	0
[illegible]	4087005.407	1998391.248	7908875.011	13266881.11	3993466.647	7575474.763	8052143.546	4942524.115	3547932.139	5161569.846	3359622.887	6233805.376	6920259.866	7857024.27	10120744.25	12540001.53	14675193.62	9973075.167	11847486.4	4542341.698	4406610.472
[illegible]	0	55802.83958	83851.24533	240178.8236	177387.0488	277731.8566	179774.5544	85578.36332	103871.622	116161.6459	96163.49769	268967.7093	224415.9914	76496.46602	191270.9533	201702.9094	215995.4328	78815.7275	299760.6798	138974.3989	43613.481
[illegible]	68425.84822	16020.27477	55544.78951	673440.4902	388886.5904	210683.427	168788.7102	83382.0605	158209.8735	200646.106	76935.80262	171249.3593	448371.8749	70881.15046	372411.2405	155321.4509	73859.16024	69259.48565	180123.1096	0	0
[illegible]	69903.1155	97129.1245	639200.8643	697830.4232	260933.1655	246825.985	357874.9686	180258.3807	219867.4972	114717.0279	0	301028.5838	347918.7074	82377.18066	405319.929	127706.6916	216520.9454	269551.4905	324173.9523	406514.5019	167681.3968
[illegible]	2665650.954	692852.5517	1156817.361	21773547.96	8024813.198	56257184.57	5121380.81	2108504.968	835739.109	4611365.836	0	0	0	1887121.89	3570195.938	4490368.73	8944353.745	1916683.614	2807218.818	5348666.084	3574162.016
0	0	0	0	0	0	168579.0611	0	69296.46532	0	0	94407.84655	25644.28847	71731.51377	31445.81641	116707.7379	25799.63195	0	409996.7668	43741.73771	140193.0544	0
0	0	0	41539.5098	0	0	225165.7991	47705.61962	0	0	51024.53098	0	0	60855.21031	297367.2253	22286.16902	78036.20636	0	48063.08751	49936.86367	0	0
[illegible]	66930447.94	79542685.06	100316654.5	84207192.33	81222103.07	125893421.7	125430509.1	86565387.86	81967645.85	79588043.2	53173980.23	147266047.5	142589602.4	55729114.64	139327210.7	136466000.5	103056035.7	155453378.5	103717602.3	143312830	57371095.6
[illegible]	754161.8537	651934.5075	1018220.709	1458403.715	854442.285	1397503.294	1263303.616	1076643.438	1615407.21	1004628.961	1316381.916	1344326.628	1058953.273	1059423.656	1771994.549	1234027.186	1422413.673	1119024.1	1848305.172	568809.9798	1902877.455
[illegible]	6100315.365	5219908.17	7780604.034	10796513.89	4230266.569	8140477.085	6909669.481	4230034.418	6717041.312	3618599.053	2329457.964	2708718.85	2144631.242	3118994.069	4926711.388	7714516.182	1197572.277	6954222.067	3910772.398	3703735.067	2628505.344
[illegible]	9062686.051	7929582.309	7748182.96	7221700.569	9353309.849	15142324.82	10682790.7	10633739.87	13322580.68	11670037.04	6835136.736	10405809.26	10486410.31	9514236.657	13839379.54	7686659.495	8210788.48	7824019.307	10450586.79	6950103.61	2138992.651
[illegible]	[illegible]	[illegible]	[illegible]	[illegible]	[illegible]	[illegible]	[illegible]	[illegible]	[illegible]	[illegible]	[illegible]	[illegible]	[illegible]	[illegible]	[illegible]	[illegible]	[illegible]	[illegible]	[illegible]	[illegible]	[illegible]

[illegible]

6387152.88	4528505.583	5127366.724	7611704.71	13906407.29	5089453.255	5901807.923	5651279.414	6403806.605	5783018.209	6255684.289	4948409.349	8789537.028	6142291.368	8195494.757	14537028.14	12879433.51	12637822.98	14649130.71	11274620.05	6397403.104	4256070.001
29745460.15	24690951.61	54601579.33	84222558.74	55169592.5	63679330.29	51066680.79	69073806.36	77023269.7	75829433.92	61150548.01	41943933.2	70822945.23	100234284.4	76628543.9	71765940.13	78960184.72	56202976.12	77392547.42	93280646.89	71538548.18	26315987.94
81834.70856	23406.34326	340259.9068	313861.6493	768194.9737	513538.9358	482585.8643	544382.2736	329925.0602	334145.5517	765509.6841	461622.9928	299320.4308	633420.1624	190695.4081	579189.995	530225.3121	333663.7961	484987.4961	207261.0662	223040.1768	417019.3863
80356.08892	177809.1294	46676.15242	123628.8072	161986.1855	202543.3144	629449.0083	184054.3247	114270.812	277796.2021	205135.0618	218975.7924	154033.2324	0	0	415409.337	407369.5042	465575.3824	303051.1822	65626.67216	109375.5745	205368.1921
315960.6942	93197.26293	548827.0337	610217.9325	417467.196	228029.1431	1278696.042	742322.8793	705137.4889	713396.8702	273464.9242	487505.1744	650863.5407	623449.0271	436541.4765	729544.1366	670601.3042	618887.3256	538762.213	978152.8064	726213.0167	102892.2829
408894.364	414498.9171	684472.5389	298863.7427	328914.5225	522579.4395	609173.8651	324521.9239	274526.1891	817010.8939	462637.2011	226378.6664	425814.3468	288541.3285	76155.59332	571417.7776	192218.6604	836764.4542	494795.5638	389767.0814	862051.9734	292062.6686
3201106.732	2038055.114	2015725.087	3279829.88	4008241.916	1928443.33	2026226.3	2071355.898	2723736.877	4110601.617	1862105.606	1797865.447	1205659.788	2424276.092	2528814.925	3317883.922	2568606.799	5205624.693	2276643.899	4220981.704	2747258.036	2146839.438
329172.067	970221.1766	1576157.963	1657160.658	2450317.116	1955668.556	2356437.861	2203917.948	2098710.706	2124405.718	1926849.749	1630950.437	1082930.991	1761727.467	2705768.02	1539963.124	2124603.923	1797573.446	10160850.01	1979398.186	3732442.931	3478882.857
12348.34191	50047.85906	19192.02913	175827.6252	151583.0463	143743.228	234209.356	124674.1956	171838.8144	142104.5751	138935.6437	126932.452	128479.964	154219.3879	154623.832	273043.0557	143890.0765	69390.35993	206556.976	213979.762	38966.32296	51389.06443
5489365.118	10730969.21	3520953.176	5430118.966	4669321.63	6627124.8	11935167.32	2811561.751	5802400.132	9259040.236	7060709.798	5190468.729	6320084.055	6429570.053	5278949.949	5590459.103	8122875.674	5815376.308	7641907.253	5719058.663	8748750.334	7372156.676
0	0	0	0	0	0	34109.86515	0	21240.33319	0	0	0	0	0	0	0	0	80334.28376	0	0	0	0
3249317.535	1698457.232	1028786.241	7590778.706	2139662.018	3886154.117	7818275.747	6501532.908	8737366.742	7571549.342	8824075.511	3916513.189	8289035.321	4667888.007	7339467.109	6731091.346	9097024.601	8141257.143	6233503.242	8136886.66	8419230.961	8299009.029
3365613.914	2440447.249	4230210.704	7540667.005	3180678.618	5390598.703	5027483.297	4445479.95	4574511.443	3704998.381	4949147.332	5234262.693	4509901.411	3608932.423	5345588.592	4663553.271	5582161.478	7352614.363	2803760.507	6780171.864	3199993.92	1439339.675
0	88913.21164	72027.62641	90958.45744	321537.9365	123533.7694	84793.71532	29559.58402	67561.3183	110247.2736	310791.761	133111.015	39886.28337	101797.4544	86989.01239	193826.651	297874.5774	145052.3299	134362.7841	343122.0274	107427.6888	0
130483.6485	1850162.357	462614.7736	7962238.251	727648.4908	175248.7824	416414.4429	513939.7029	307633.4656	335387.8793	182943.356	315100.2829	5072868.956	322398.0846	724465.1107	573860.5435	431583.5891	760242.3793	858016.7397	803258.3728	411786.6403	181310.3378
0	26763.76401	0	126385.1368	0	0	0	0	0	0	30453.8727	94212.53049	0	0	0	0	0	70223.40052	0	0	28931.90606	0
34699.9601	0	403897.7603	485509.3888	493695.2924	109492.4193	264234.0202	0	0	0	0	50924.16086	106349.2931	0	138428.2414	886791.4254	475344.8299	724912.5862	635824.317	453508.5484	0	241554.2409
397473.0472	500629.0318	424615.0906	400793.2018	1377450.265	253512.8964	1142681.336	264932.4106	327416.7786	61726.87493	249519.6817	755671.8986	103216.3175	202448.1522	71852.55283	570150.7714	938998.8277	673991.8559	436597.4768	865232.9223	194458.9783	332158.5763
2802158.365	4740407.397	6420309.694	8257284.533	8365762.003	9101159.331	9668936.344	8772677.911	7438800.02	7551775.467	8651894.127	13172472.89	8421198.993	11563040.69	7267495.59	11406734.82	10977402.09	8788660.811	9501879.733	10106792.45	7426851.573	5567122.006
97475.96096	101536.5395	860771.5192	766775.1939	1081363.229	952659.2833	1003797.473	908307.7103	1083981.704	1116221.781	746133.5568	1170712.831	1076315.148	1255927.871	1589019.681	1133298.77	1070729.709	1489818.857	786206.7262	984081.0913	974443.5959	159139.0157
1182560.038	1694073.498	728466.8037	1759645.272	10304240.49	1898173.545	3393354.091	1926177.95	1126452.647	1987700.919	1544080.743	802559.0579	1224608.853	1322724.011	919091.4537	2352744.058	1016737.394	1990008.765	1739162.773	1588590.771	1792199.566	1181416.428
0	0	0	0	0	0	1183421.754	148751.6799	0	0	0	0	0	0	0	0	0	0	0	0	0	0
1289121.405	486119.3746	1304550.795	3464214.28	2079556.226	1828650.334	1642015.577	2045921.578	2607964.351	1608350.668	1489172.329	494876.9666	1484736.013	2053086.221	2505759.832	2440586.577	1931693.219	1184193.028	2117812.303	4683605.96	1376950.66	2276339.111
462428.7493	165518.6404	602501.4696	390925.8524	18459234.1	0	3606850.507	921468.3706	245224.2438	588444.2652	312517.9824	165502.9171	834296.672	445663.1616	2794307.117	410145.152	4261842.051	1935702.963	2159675.529	1020522.81	1771983.407	4180569.054
18709469.61	22196226.36	14650473.56	16769180.05	25417972.5	27230804.17	49147997.54	17942451.77	12543299.49	20362350.23	24897070.59	15027853.59	19845474.08	18133852.28	20247060.47	17187148.81	14559709.43	18845986.46	22811557.15	44004455.45	29433315.31	22964473.07
108561.6544	25400.46529	0	143715.3153	178995.5746	112689.8247	465710.0271	127493.9002	140898.2573	124481.9769	109188.7996	191855.8289	66523.42641	106286.8518	274228.8514	397380.4759	134893.0645	301872.7812	166266.1406	474381.8812	9908.978986	93805.43704
4631447.777	6659558.672	7337758.45	8784197.853	13495623.36	4454817.002	7342632.385	7508176.738	7183050.84	7614458.433	4231877.652	5919032.802	6052356.592	3310736.11	6031502.309	7004060.718	6268081.284	7683102.102	7301406.252	7842075.143	4482978.822	9067916.023
0	6291430.458	0	842399.2943	0	973735.1693	5867854.152	0	2814094.906	3493081.216	3668379.227	1673466.724	0	3748550.766	17214534.19	0	0	0	0	3175543.363	1850413.847	0
192367.4507	372283.5672	520924.2314	679195.5524	1200322.801	1444698.627	1113848.263	419839.1664	518275.3361	597753.9893	833920.5701	875324.5352	160660.3749	933507.1882	847435.8334	730269.2928	474338.5755	1156047.994	869173.3063	1132193.822	660524.9098	610276.8294
651814.9358	432502.9171	476218.9297	1625395.478	1875876.567	862160.2891	984549.5465	938075.5694	663317.7025	1657817.561	1000280.206	1881851.009	1257432.928	517448.2767	1438217.946	766104.2798	1937831.487	2434747.365	191550.2717	2312002.945	2038701.377	1574354.081
0	41606.29441	222616.3515	565073.9691	1217616.903	83730.28898	797341.8638	589048.3213	245564.1971	0	0	252115.4654	114473.8879	359720.986	426768.7734	439670.0645	364498.219	3334211.858	2975622.169	33107.99531	106752.6226	292897.5245
661280.2332	1137388.346	3047184.167	8035316.152	7639042.131	5560091.324	6481936.895	5845762.743	6049916.614	3578689.727	6668857.697	4578161.218	4917470.679	6882571.612	6434573.842	4915883.182	5234826.967	4974706.774	3719701.161	4803300.958	4288258.049	3747365.922
8768137.009	7634747.51	9041274.414	10344950.79	18523446.79	10345994.66	14437044.84	12888983.55	12246144.92	14875922.95	8863374.917	7688566.824	10472884.26	11304724.52	11955010.09	12088181.54	12522368.71	10360008.84	11466348.49	8126378.552	12699802.51	12695313.67
1203893.154	350136.4934	1647871.887	0	2644210.406	2010311.251	2481815.366	1601985.403	943426.367	1275241.543	1090951.829	788849.231	1508134.155	1581012.179	1573292.645	2202981.478	1975603.933	2033308.656	1439297.686	1737569.71	1190104.62	511753.7479
0	0	177414.5384	598188.5204	0	507842.2205	323110.9738	570221.4681	440143.2724	0	299603.7558	0	0	0	283616.2497	0	0	2622892.76	340369.9758	0	0	0
960806.4645	955806.7661	363581.6694	290190.9399	1395225.16	916014.2702	2771672.139	402239.3479	417932.7872	656109.3873	948584.1033	531756.2639	525748.3031	976367.8959	707639.1514	521328.1267	492238.2964	941563.1893	531015.6584	605267.8188	461787.5444	388280.7542
14980124.64	25511234.07	36792885.67	53783270.03	45869928.09	51188644.11	58358740.57	29743457.37	51985836.75	39180052.49	42158585.49	38236938.18	44278260.97	50053274.82	54570318.19	52367572.5	58999846.57	48578602.37	59098023.32	60881684.61	33929912.83	30369245.59
3427127.773	3561063.652	6655098.412	8293410.12	9559111.134	4213932.539	4399945.377	5779131.755	6540176.2	5283067.261	4898597.794	9092240.012	3324006.543	6552870.137	3044001.881	3949554.995	4111404.892	5187167.956	4324018.34	10406687.3	8559414.42	2487404.378
0	0	712537.1972	1147352.292	2088107.761	493303.2894	2040384.085	631383.9591	715563.5497	0	0	1095909.792	483931.5505	996142.8552	0	2045565.162	783377.4559	872790.4317	611641.3299	667656.7163	252476.9506	0
4354674.408	53305339.17	10309962.67	4035042.089	8136975.116	9665929.452	17325877.29	6272863.305	8875311.699	12525319.47	9612817.74	9896718.752	5378788.636	11135917.24	7486575.825	7849763.553	4729728.287	10441617.19	10231279.94	6149541.476	4391077.039	2830986.299
152959.0522	69012.18149	250841.4158	385391.8328	119732.9552	0	720952.7549	0	0	0	0	438284.9578	0	0	0	183005.8298	116041.4107	434991.0392	196480.8907	0	0	0
0	59846.3474	93755.03233	267560.8421	100359.8303	138509.8828	368881.4469	179482.6984	149443.643	87290.73375	76586.09749	45623.61812	90237.78848	0	295319.5394	18962.27657	202822.7165	267964.4836	70775.4842	339721.4254	175291.7015	0
62876.81065	343782.4961	412069.4887	127036.797	881811.4359	0	1055766.728	479255.4349	188219.4957	117477.2674	441950.4557	0	0	0	630495.244	677732.6601	980204.1471	643784.3576	418384.0439	216823.7074	314998.8418	133624.2958
70841.43924	0	270945.2555	223932.5487	407156.0406	77092.61553	144549.5397	93357.76112	234620.2446	241027.2262	522918.9236	45175.32755	73692.39844	77411.45487	225045.6602	530202.9389	339713.1552	135514.751	119067.2928	318424.7532	227419.3234	0
0	0	0	0	27824.57181	77264.08244	225453.2092	0	0	0	0	0	0	0	0	37811.94923	0	118262.9341	0	0	23067.60954	0
873462.5722	418556.9829	1016352.747	1019415.539	1211321.725	1312617.05	2382608.484	1878508.993	2287414.88	1130506.155	1358948.856	1386811.362	2615025.996	1640367.338	1223932.185	2060056.892	1361547.118	1258006.25	1050724.555	1624817.7	1023833.52	1277187.848
676174.9974	616328.5333	1107545.156	865451.9516	1405506.216	617794.6531	1448628.104	1108699.007	1313324.655	1393845.434	1415611.929	938360.799	422116.8999	1247650.524	1942873.364	2182913.983	1718241.848	1977921.339	1889779.681	1274919.497	1058886.238	245207.7098
1106745.999	632988.2968	1379523.746	1716723.415	2305758.552	2474307.26	2285230.773	2272706.721	1484074.072	1053562.761	1626959.638	1462224.131	1545517.969	1147983.499	2054867.166	3473829.614	2920669.112	2716221.786	2256380.657	2421424.608	1623652.551	1334715.347
543622.7515	237272.5989	2085340.16	2183331.261	2516040.098	1655591.559	1384072.089	1941875.56	889173.624	1506328.683	1909462.671	1229679.292	1071117.439	1939226.841	2211685.228	2043384.215	912910.2034	957753.9057	1599626.457	2551183.53	1464919.764	1064647.476
237123.4988	459492.0072	257231.985	351835.0581	640677.6999	414510.0598	2207601.052	657779.8266	1174540.585	1549282.224	580527.1448	150471.2048	335327.9029	573991.6756	228175.2562	1458338.762	1415752.728	1294861.882	266032.8611	890550.3445	376819.9788	138905.1788
0	0	0	0	0	0	0	316715.8948	0	0	0	0	0	0	82694.85969	77361.62283	0	86571.09035	0	0	0	0
8104118.934	11981389.54	11192194.16	14811517.93	19361461.56	13757568.96	22926363.48	11084123.5	20318536.05	9112967.097	7796594.968	12702343.09	11332991.42	24392214.45	18807056.55	10800196.25	13830493.08	9396871.319	11607700.38	9020750.227	11125617.18	12840745.87
368910.4534	0	0	823734.3807	6927317.89	650996.1958	1945529.156	774554.4885	0	296173.524	0	0	0	0	170709.068	133893.3423	0	671128.2381	173572.8176	1433374.87	499438.108	1055977.092
3239515.478	3382091.299	3298933.617	4730111.604	7803819.884	3920185.695	7210436.494	3884304.673	4502311.571	3647122.344	4025257.927	4810609.489	3251772.528	3915077.532	4148137.397	5068036.449	3889915.028	6864712.066	4706290.842	6082527.505	4039390.602	3088788.908
1391896.397	1106018.789	7638722.634	2705655.471	8440324.92	2434615.949	4034686.342	6079751.982	5608338.253	5058805.298	7884133.013	9751172.949	7982371.143	5689688.187	6708676.435	9760513.078	8205789.156	11893245.84	5590264.467	10311552.84	5071102.518	4841076.399
[illegible]																					

0	0	290494.6586	1285593.008	898337.8907	0	1281339.129	0	0	0	0	159856.3405	1002608.15	1394698.804	0	1442483.827	1515212.527	1806535.395	1019700.655	695628.7365	2357939.935	1986775.318
0	82009.72191	60438.36646	438647.3959	2406718.891	218247.8101	1046168.98	0	321936.1232	85836.21119	350362.3097	37614.26791	92144.81259	0	0	1178645.198	550515.1224	628693.7083	646326.9748	528363.1543	59228.42329	352773.2285
19895803.88	14245788.69	12882723.19	16823307.94	34507677.93	37827449.86	33946416.86	20554630.53	16010057.22	25877139.59	23295725.12	23184918.34	19504230.69	23601741.07	22402626.76	35592284.38	3953562.131	33356859.71	33090732.85	31534564.3	24746241.44	19443179
8508245.896	10224242.32	1751391.216	6178827.553	6674464.775	4611535.528	5312835.51	2683682.984	3071657.331	2562315.574	4663792.661	1988833.303	2457737.624	5675089.606	2852893.112	3098775.516	6320358.678	6489780.717	6778684.442	1637248.186	6424615.461	5957507.938
31083802.38	33050916.03	57471701.77	70277514.18	52449341.12	40251841.01	46643857.02	67666150.17	65590022.1	54740321.11	54858382.43	57706415.61	53653892.17	66881900.06	67011719.04	70966970.22	63894109.85	55217356.3	95831114.4	61504802.83	56310791.78	23885873.11
17281775.59	14004577.16	14935507.09	18827157.84	37153386.12	17498771.4	23847712.8	21011879.38	14702567.48	15355560.51	21958638.38	13241054.12	20583920.72	19839831.2	20924637.91	20557529.03	26334690.24	23343024.98	17694971.56	21448571.77	19298188.46	14221500.28
8860532.224	3629397.21	204276.5999	2638208.148	3260542.236	6192960.308	7530587.262	4311142.657	3598592.221	4765909.796	4150136.76	3495388.636	1800216.296	3000551.966	3237166.704	5565889.18	4758065.576	5671910.343	1922729.098	4241145.844	427742.6927	2247349.32
393080.2433	645011.5655	88103.01975	236705.0004	350437.5784	730611.2828	814813.4004	451726.9002	647306.541	1012649.463	381627.1932	327952.0928	164368.6944	527601.4215	618936.6524	1064999.52	699205.3314	528291.4633	242384.0413	608658.4866	225433.521	285654.7255
2324699.034	2185948.733	7008806.632	3468707.985	6603187.968	5539335.131	4544176.183	4419009.985	6742374.421	4561810.616	7718631.011	3001724.41	5506442.033	5586756.976	4546355.684	8020629.705	5556278.12	9432820.063	6927979.104	13812111.66	5155347.253	5044521.828
541408.4876	1754638.865	324022.8937	4208462.382	6389782.242	5805628.544	6634668.981	2354639.455	1966219.024	1480505.807	4171160.714	677044.4864	777947.1552	2151799.961	679987.2732	2910099.735	3067186.506	8324402.661	1836006.44	1654139.639	4114917.874	3651036.798
26667.6428	78519.66071	29741.19275	453781.1682	3706075.053	219827.9301	1518192.831	164239.8192	27600.36846	13935.75038	68103.63236	0	82385.25721	136494.0381	48717.28604	364227.8809	11481.85911	1007602.264	15700.71435	202151.5673	201281.9339	66332.74849
6997147.533	3826870.137	11579647.49	10481815.81	13926067.85	10873787.83	11981758.26	14333294.42	15901977.23	13195066.6	10495687.93	13150051.19	9681263.986	9464431.928	15604862.61	15170527.55	9731164.997	13660541.4	7936228.452	11675783.65	9963405.399	9744948.049
11408.31226	0	44079.29725	99564.59734	493150.8295	81201.64133	448163.08	107996.4672	167184.4733	123005.5706	189093.1214	0	31766.67325	50232.25381	58893.01828	131332.0929	0	32371.62319	0	121967.8587	38828.63085	20211.35367
15383.66012	16626.85851	14175.46139	416101.2168	387002.4887	345618.9093	285539.5113	258221.2038	95559.16002	241694.9484	158038.7776	62249.08825	225675.0126	98701.47728	129806.3182	280179.6686	70548.85958	244959.9324	253562.9955	380862.2385	27908.12149	172191.1386
1285575.833	1064068.465	1068416.314	3708002.678	3220828.016	2392890.734	2465879.983	1199051.388	2498335.862	1426315.255	1630977.335	1002176.62	2742855.106	3876055.544	3260468.922	4095748.656	4746636.548	4072560.629	1874859.4	1314554.186	3097310.911	1118031.583
5245429.58	5212168.558	12838235.12	11198553.74	9460943.816	9911030.787	12103188.65	12946920.44	10971246.8	9099612.963	9350744.026	8758273.184	9943900.769	10226495.56	11423122.09	13799827.7	10903716.04	10656515.43	10630197.3	10024755.88	11249946.79	8958203.212
0	113014.7482	0	224228.2692	336532.5435	527649.7932	487631.5686	390487.6818	165630.2658	667355.0216	1049398.86	0	491278.7522	363122.3192	798376.6603	424370.5499	761987.0584	541633.7157	363215.9391	1160639.96	132808.202	742881.6982
1297736.774	527899.1262	1768188.048	3581540.876	4105773.07	3140515.513	3755829.764	2702790.583	2210130.064	2419650.174	1827090.954	1643110.077	1891406.634	2080264.758	2655203.612	3264855.285	2324883.598	4127953.34	2157165.609	2844221.744	1254929.355	1041493.407
1032152.118	0	0	647258.0092	0	1286719.629	409025.9421	831239.2698	1634198.987	1046068.13	0	0	1010661.618	0	493553.9397	1510288.23	0	0	1033647.991	1861990.862	713209.9339	862262.8142
1444395.653	3602901.486	6129818.061	8121649.169	8604510.953	7810774.687	7227503.435	4808496.2	4718920.473	5269009.301	4512275.905	1919560.432	2937502.827	6816480.973	3346543.21	6996013.358	6222539.542	6459018.063	4763442.058	8418807.56	2689111.033	4125769.605
15882327.82	19379678.09	14626708.51	21939393.21	32579774.07	30299234.69	19644194.88	19975206.91	18709469.61	15564939.27	31974266.58	13921862.49	15020914.45	18610763.09	14671052.15	28032427.27	29822449.37	23637707.05	18978256.51	27907492.81	25600946.28	16215449.04
3067168.303	3794770.933	2374174.082	4701366.909	12087227.72	7363822.878	23887899.97	4961179.74	3164620.469	3713993.398	2652968.681	6474157.895	4005075.297	3599842.975	5148785.396	6045895.046	6563003.728	6124679.666	5028374.521	10325453.37	4733315.026	1857805.276
1128375.112	649503.7644	1714667.065	2111847.292	1919971.692	1318463.398	831825.5516	2769601.961	5266680.415	6403949.603	2889388.282	830244.7239	718402.1583	904382.8723	1276017.128	2583549.227	1977567.049	2475143.976	1234255.045	1240269.766	2381248.74	773564.601
13425256.23	9420624.785	16554438.99	10712430.67	3293088.742	13123941.1	15074223.72	13714661.17	11493135.89	15671931.39	12040005.94	11717579.47	6728582.951	6341440.505	4904715.594	6649896.645	8196171.332	10549857.31	6366627.378	10076865.48	7537710.562	10607955.05
419707.735	64208.32514	242448.1678	1132340.295	1927705.447	740253.6949	628791.9665	575643.7187	132644.6186	855227.9536	396629.1994	130764.1187	193202.1976	60940.29697	1040697.426	253113.9789	900840.2194	944473.5162	815382.8796	308634.4735	239280.8152	1472922.883
0	253927.9459	0	357683.8909	454129.9743	2919069.691	2862977.547	350758.8558	0	214485.4955	3047606.507	819810.1345	373027.2782	488067.904	0	1582620.515	867555.2081	1488168.047	1420859.509	989075.1562	1488030.585	364655.3793
932273.4903	1640217.732	1326395.239	2384415.816	2144210.87	1359133.129	1166477.586	1047542.691	1331794.858	2556036.399	1152036.665	1917266.2	1530633.368	710663.3169	1295822.327	1664762.507	2242724.368	3330701.765	1548635.069	4032138.005	3460182.602	1780425.843
11047431.99	16941290.82	9885107.942	17850156.98	21445271.36	20243148.48	4534129.819	13798991.15	13086883.93	15644831.78	16406454.84	16413933.78	15982201.42	19164228.45	13214123.54	22315508.2	18624177.21	13545519.9	15131424.16	9689277.657	17631976.09	10867426.85
2479278.14	1588114.32	944869.5296	1367526.095	2417244.382	1682757.392	3212306.581	1344265.121	583007.3337	537777.0725	755795.6076	3508400.005	1524966.182	1837382.512	1115735.979	2393637.889	3406455.41	1859785.642	3437373.846	3497236.676	2692495.695	1829684.681
38257.39936	1290335.465	96706.48593	1177934.975	6381693.158	1081007.296	2603904.279	627650.3949	183312.7239	159501.6579	421646.8861	347531.9292	472573.7088	292954.332	383398.0977	1218295.072	384743.9531	1187810.108	279500.0354	301556.5082	514156.1154	495526.4407
0	27375.48931	149886.8056	383923.3566	2995070.925	1555175.572	1610572.055	237546.1839	398308.483	112096.391	2268692.356	54714.49715	332256.9947	779305.9134	92908.87009	649159.9647	1020104.785	2866280.102	366821.2179	1578835.778	154176.6768	379312.6638
0	184030.0284	187284.3275	233765.9687	0	1051582.154	201440.6999	234253.1158	1321842.753	798610.455	1673704.354	717743.9638	640596.4354	624948.6833	713269.5718	0	1770981.912	430230.2486	719178.8927	2741395.093	253208.201	636166.1615
8581243.497	8482269.458	9068116.699	13583861.29	17661153.35	10184053.04	8644046.815	9370090.159	8059645.066	7476690.312	10263903.67	11143698.41	17655276.04	15269803.76	11507020.16	12230988.39	12263742.22	11272697.93	14265756.36	17328773.73	12068385.35	10639593.45
1618982.932	1369002.707	822393.3634	1341372.684	3478053.943	3282646.467	2254768.525	1236990.078	2020458.005	766199.5175	2053628.764	429273.6806	398283.1578	1195633.543	383916.3637	3043862.614	1860773.164	3027821.823	1402652.879	2077711.997	1396111.975	328583.3074
245799217.6	62225101.62	33319809.44	17054853.2	539684.9139	0	345959.4986	800026.2999	997557.9371	0	0	54883585.7	122624010	60489584.43	100285053.7	737348.4783	0	9156766.907	0	0	70083211.77	2496308.383
99994638.78	130474946.4	183921451.3	188273575.1	182335958.1	202359929.4	192254621.3	218359867.3	150825295.2	157801239.2	157152742.4	153390688.9	158079827.4	195061967.3	246801806.5	221724677	164382026.6	250569480.8	208461608.2	265642547	181337182.3	177244161
1261190.637	1134460.515	1676878.76	1522411.804	1167856.495	2425889.215	3475619.084	1591413.722	2452612.7	1838248.881	1638728.446	1905939.622	1192590.089	1636638.591	1670526.869	1878874.359	2700409.253	2097531.26	2000386.927	3284569.023	1566256.808	1213492.341
17639962.53	17081622.24	8688443.992	19407633.3	20237779.64	30434051.68	21877492.93	25896910.25	26258019.71	22074642.96	23153673.61	10634964.81	26026780.77	15688264.08	25394943.89	16975628.78	16444960.35	17002431.03	24634112.7	21126156.13	24969926.77	29921583.35
3401455.665	7622628.689	18877051.45	13289378.09	11062915.32	17086084.49	8401691.049	23996368.54	18405579.08	21036948.88	11482743.42	12069738.17	16955879.9	20233238.54	20165419.18	21961862.43	27191249.11	16481043.77	14089439.65	17508072.5	8520568.616	13812877.94
553027.813	1520792.651	2300606.336	3153953.144	4214724.773	2746335.457	3286094.554	5338201.764	3123115.281	3776098.458	3134348.997	771087.4655	2127138.605	3056032.777	1878144.084	3050306.662	3308200.975	3900935.068	3228346.178	4765446.596	3011374.341	1609230.184
214550190.9	190661236.9	385902998.1	464659086.3	331969472.6	317282843.9	386665340.9	394155259.8	813063620.3	339914221.1	438849237.4	273546153.5	299728907	385179780	374094793.8	428532980.5	212272025.8	427515670.6	418987100.9	439505630.8	174722090	133876532.9
901369.0418	396259.824	3212288.008	1506906.285	636623.6592	2215195.44	1338443.997	1512669.415	2263642.943	657684.4762	562168.9181	577455.6711	1940334.793	2114971.909	2601556.246	4032811.781	2970291.76	1101221.644	2544061.412	2135591.534	1600544.667	1254342.262
1829446.808	1073629.692	246139.9234	0	2252354.48	1353373.275	3000512.183	705634.4128	1968496.461	255252.4787	725386.1147	1222176.793	100017.8632	674162.1946	1557234.678	1468428.506	3285977.45	2762382.253	2948219.322	2104510.986	257615.6401	1643507.299
54959841.44	44269504.55	94685022.58	72570227.58	100209280.3	70009261.65	71433595.19	71928287.36	81693118.34	92452993.09	77030717.19	74569309.63	75490778.59	72789544.39	77638433.14	123157941.9	109555797.1	98996123.97	106562349	88741488.88	53434447.48	84197678.09
3312053.101	0	1062166588	92130530.58	115370801.4	1956675047	442178697	1381339138	667164053.7	552013134.7	1065812707	1038698032	1198491176	253151599.2	409644031.4	1028688967	916039593.5	1055972539	650547168.1	951799866.9	1532002883	1313045442
11090112.26	0	0	925558386.1	771552353.4	44788716.09	2943264.993	27737344.93	2386339.012	10242495.1	5249835.934	0	28094700.62	44992585.07	26140621.15	5243850.348	15040491.02	13264657.52	0	46216447.63	21692924.87	9365532.711
0	0	0	93151.4092	7327.509705	0	0	0	0	0	0	0	0	0	0	0	5865.192281	554200.3509	0	0	0	15482.01552
3437472.618	3187075.169	14706446.27	15168248.4	10236916.82	13909001.75	12949109.76	12772570.91	14934845.77	11770486.99	14655024.99	12722455.37	12445924.77	11873578.12	13814159.17	18418171.63	26385098.61	12553060.61	18397516.92	16937188.43	17183580.61	7023258.897
0	0	0	0	208699.4905	56578.83482	121522.2014	101207.162	222231.0862	196944.4995	0	0	0	0	0	41985.12276	0	0	15953.80957	0	0	0
185803160.7	122746864.6	295799268.9	293580593.8	164335965.8	297903611.4	160737083.9	344472555.6	225722040.1	335774329.4	205126890.2	295151827.8	310273589.3	239339182.8	314942906	326713652.5	327070106.3	406844691.7	408727612.9	407315104.6	313009805.1	198943911.8
341539.8057	0	120143.2553	43856.00636	429624.3902	643487.2303	0	0	0	0	102123.548	108746.4761	283669.9439	155439.7566	0	0	0	126254.2416	0	1395321.225	886111.7697	0
81801491.65	89511459.47	246746694.4	372476725.2	69675466.13	185645403.3	103879311.7	298392414.4	464447842	208048133.4	245742756	249622576.6	181878492.6	279151461.9	195730464.8	224891878.1	213768562	201689363.9	223382079.9	225092215	157597574.2	173492060.8
1821950.521	2276585.733	6031752.906	1874143.582	1879628.809	6505767.272	6249737.189	6177291.048	9306856.022	6181000.703	10217526.65	3467922.151	7378614.127	2789096.695	6979084.746	6896415.195	8673233.328	5780863.23	5781551.187	8998055.962	4384946.644	1363358.924
480056466.4	312459974.9	456267769.2	471976056.7	261818947.2	441823385.3	328868749.2	482511589.4	1017489351	412782431.7	459360923.7	254659218.3	378975886.9	749516412.1	546052067.2	551919830.9	373612583	277516857.4	476052744.7	354020298.8	349531173.4	291522989.5
5007566.36	5480650.116	7402938.333	18757829.04	9423762.004	5301388.707	8362143.471	5184422.036	12376685.16	10227152.79	12760981.23	14017227.57	11405868.81	10916655.78	12072315.33	17212934.37	21765545.14	33755779.74	15738860.18	22048983.01	12359797.7	13263403.69
2104875.841	1014185.616	915151.4628	1025041.84	0	2720855.427	229711.5747	399309.029	2236404.347	0	236898.5977	586100.837	2137741.297	1941529.057	0	2947672.227	2715149.543	2399634.295	479625.0666	2214015.622	259949.1504	258091.5152
2101934.621	4870423.101	1237923.83	3311543.943	3505557.092	6086586.12	5908400.827	5868857.7	5600713.073	6650427.187	4904728.445	5845270.764	3676291.467	3734092.653	1613384.646	3428328.653	2061138.638	3294457.107	2485701.836	4661582.87	6910502.465	2693652.883
[illegible]																					

621090.1246	243439.8374	594945.4646	1189794.062	867948.5817	1108213.67	1511965.178	473836.517	400748.4772	834528.1176	985970.9269	380305.6238	101475.6809
363244.1645	191938.1263	441732.2837	179965.7935	192320.0508	1465155.716	340448.767	235036.9534	301534.7334	151802.2048	365023.2135	342294.2103	60912.46067
4649831.49	2825540.319	7401154.711	2818738.647	5493819.688	2502803.2	4406152.623	3721566.826	1411429.939	2791157.836	1318554.571	9095368.455	3957418.903
1421581.473	1438504.511	1200386.872	2711921.547	2710176.769	2575268.767	2448022.07	5682943.473	7218546.295	6475334.088	5229748.841	2973769.692	1620863.419
192560396.7	201945162.8	228928283.2	238825522.9	214021320.7	228248587.2	304916187.1	255552640.9	238503768	255840534.6	209029830.4	146773374.7	107359289.9
8411150.557	15248575.11	6885507.663	12076548.37	7889872.397	5161792.169	11090860.89	7744470.892	22177675.15	8559548.412	15772628.2	14940347.19	6831903.348
233896.2872	40314.17209	749368.9413	129205.3279	41171.6155	1209151.73	131132.36	44504.17537	479174.8457	0	161089.0716	0	55382.86861
715776.4467	226305.3406	1133778.734	1230568.196	1771716.431	1297165.154	772886.5604	380103.9529	721158.6528	351056.6142	754241.1163	984840.8802	296190.7426
0	0	65292.6924	0	130062.2418	122145.1625	594972.0761	127084.7696	509284.5026	138803.717	378687.2188	87176.78276	63538.64901
7931773.952	5869151.8	3864792.204	4382447.35	5544831.679	4762729.169	7649868.351	8663093.241	6774338.608	7741751.276	9787525.786	3195570.567	6246200.624
849256.4422	614856.1382	147669.3446	754298.0095	1109576.622	1067208.43	2090291.922	1592421.915	2897575.891	746305.7572	1647459.087	106990.6975	223323.2598
14670.19923	152019.1468	313736.5307	455340.2533	672433.4403	641201.9736	693250.8979	333694.903	533835.5898	762635.1261	380424.7208	40440.33054	183187.5731
110887484	197422127.6	96801973.17	119069084	157885276.4	152130190	0	0	101948373.8	130777435.6	109773020.8	129748389.6	156370596.9
569013.3302	526184.5189	0	0	0	77326.25097	548522.2621	345192.2614	907377.4873	524482.1142	654073.3874	635663.239	0
1068869.191	1400632.472	1066107.877	1261767.423	1075464.722	1047822.757	1555469.248	2168085.274	1441291.296	1694170.616	1386915.027	1424890.703	325355.4694
443629.8628	18365.39584	477873.9539	453683.511	250013.8809	5802.484479	13878.03247	480567.119	47035.78249	181282.2364	23527.94796	24601.03663	4570.617046
0	0	107890.2297	234535.8131	566458.984	70066.60185	302957.1372	455131.2928	0	642730.152	342024.4392	0	0
2002266.717	2768841.684	2168337.363	1659629.341	1720066.7	2154679.027	3122258.986	2028508.31	1640654.011	3186551.11	2694429.055	1370304.087	153008.2345
211595.6855	60193.45131	307595.7539	177049.1553	388608.0229	208093.9006	464483.0332	254632.4439	203018.7438	491491.1328	221066.1149	338200.2696	0
1631021.656	0	1093367.529	624800.8239	1133945.113	1053832.981	1039645.864	1217067.179	2199163.33	1023936.174	1350142.531	603904.3322	1242337.353
39524.79095	229638.421	47702.75234	44537.40726	811616.9689	99190.71017	277668.3065	0	67425.68751	77505.11362	1694399.981	35996.27686	44785.09361
356624.3939	505030.148	560470.523	147183.6143	459544.9403	135365.8175	1443788.716	1031535.177	3054287.569	2496082.055	2088712.265	434014.3114	345544.208
211287.9847	766354.579	333326.7752	375543.8741	195851.7541	0	617034.0615	381895.9864	969523.4199	0	674113.2062	0	0
7032213.006	5768711	4436530.921	11532815.19	7161935.16	6690845.602	6605630.523	4485326.029	6058764.109	12770501.11	13054846.45	3414848.915	4239565.283
10348814.91	4412211.447	7465761.358	4247372.764	9685349.771	13543249.66	12183786.69	11178868.57	6636375.064	18627203.69	13992008.24	0	7627247.476
127393.6629	214810.9484	86213.88377	0	186106.7159	103441.5177	56581.04528	110796.948	218411.0295	137948.2244	480530.9803	0	21157.61825
938012.8704	1187665.367	1294765.674	666476.7443	1118575.92	789526.6518	2112402.43	1127505.996	1914277.758	785368.4367	1717618.535	855859.6839	462967.9998
24110838.43	38376306.68	29784580.21	31630371.65	29576952.91	33185267.15	36763925.33	28385463.46	34734637.21	38559568.45	30454990.07	22100699.91	15237323.33
68549.72731	0	42074.52362	172119.4392	0	0	431132.4777	165567.7586	38003.67149	0	215136.1621	313632.7152	79643.40259
0	0	0	0	499345.9558	0	0	0	0	0	0	0	0
430938.6266	2350648.381	612424.2261	2694985.741	1441893.884	443386.4059	0	1018996.791	0	0	0	3742796.674	0
0	0	54732716.23	123247735.4	60151782.79	99490626.51	736656.1783	0	9134386.461	0	0	70552149.92	89269464.76
1078922.005	704430.3894	806696.1779	1583277.339	905397.7739	1084934.078	935928.3667	902700.9334	855272.9212	934636.292	276679.169	1430018.404	486218.7116
51427.94174	0	0	0	0	0	293544.0806	0	0	0	214840.5654	0	0
139760.3018	591110.4187	306624.8441	239494.1755	1382260.253	0	657152.0745	303341.139	229152.6499	393735.9893	244848.452	231566.2502	456478.765
0	0	0	0	0	0	396436.9364	0	0	284833.427	338061.3872	0	866613.5143
13653164.25	12338131.01	6204139.135	6513831.663	7598420.416	10409622.96	14034549.51	14621464.48	19809140.24	11270856.5	19950431.15	13190801.57	7833819.847
228628.7041	316755.0204	37911.59374	48723.97885	22224.71699	260574.3788	132691.1289	120367.6428	405694.1905	88617.69573	285859.4396	59536.90471	0
0	0	0	0	0	0	0	0	0	0	13750.28716	0	0
1962735.058	1902909.386	0	768849.6839	0	1152896.776	0	1026299.975	919190.8396	1067175.439	715805.6355	2491932.762	2802565.038
1621168.596	113458.8515	0	596056.5862	876942.9604	1960756.98	11093.62794	646414.4971	3259088.686	467740.3083	0	655406.3099	0
8097488.162	14404648.56	18187715.65	14820216.41	19022467.24	5892986.623	14477278.01	10524815.24	12306081.38	6203053.439	13772554.14	5030629.231	4108844.228
15096.60607	14498.11013	301464.4637	226341.1145	345677.4433	285450.2506	414524.3663	333301.1291	350643.2403	180532.3896	494188.2409	0	0
1691178.892	2260378.223	0	857461.642	0	1214363.973	1925656.655	2766295.176	1802969.021	1338514.863	1027644.766	1241568.424	1178485.408
246239.7731	114272.6894	221014.3075	256709.941	218998.8926	524522.1723	179797.14	400446.8008	604928.032	587497.598	893266.1425	162937.3542	71729.39771
229334.7823	505656.223	440542.543	410911.1694	142285.0109	856846.2543	1355529.477	693800.0267	965560.1332	1141992.114	645887.7713	12663.93911	65283.18767
214168.6107	99588.63928	2555045.716	290664.5444	706549.984	277532.9834	1333233.136	415669.4787	898576.5293	761758.8708	1039185.855	138725.7301	327200.7494
1853996.6	1157534.863	385379.0297	1744575.522	1389849.79	2619360.991	4497190.407	3356629.463	2890972.09	1533746.034	3853757.415	835571.2351	1228443.436
475401.0711	386686.8241	0	0	650560.0038	437050.7432	623400.1333	555536.2587	0	69825.08063	198906.8959	0	47960.81659
7519883.071	10187853.08	11846189.88	11423548.69	13556581.09	14342353.73	12630132.18	10695802.45	11692487.08	11173442.64	11831237.42	6498440.514	5856035.45
792899.5018	724557.3353	458660.7979	361148.6178	308615.8118	462042.2281	408343.451	688702.5671	1172580.95	573415.2437	532314.5335	400074.7064	245956.1349
17697.69356	31778.35558	49625761.83	56187587.65	77914541	12576.67696	30352.82475	0	70747002.59	79795021.66	67578359.24	84236.36422	0
1198243.455	2276659.1	1293346.278	1390062.316	1906158.832	1828057.741	2839028.883	2446453.066	3251955.185	2174218.739	3119172.285	1823218.418	1992001.045
486433.6765	120547.324	520463.0051	430175.4122	683856.7707	474631.8997	779091.4111	403694.3987	334765.4544	723958.9196	961849.532	303989.0998	179245.5176
358925.3577	163729.5521	0	0	4977.028626	156285.8834	0	129602.5003	35277.06866	0	30828.12653	0	57551.82194
0	140105.8208	582402.1932	793860.8812	219669.8041	1373924.986	1026405.045	1629601.846	741195.5524	798365.4622	846734.431	232609.4983	0
157686.5647	0	501828.3423	724598.8728	340545.5755	2132816.536	760643.4195	449107.6862	568239.0515	0	615007.0149	1408364.403	623317.8648
524990.9789	509981.5546	932501.329	183497.4189	514562.4895	127159.3494	231814.7403	725075.7886	536573.6357	442747.0688	273963.6507	588253.6079	199046.6032

[illegible]

[illegible]	5891943.584	15417725.47	30173428.01	14106685.65	10939120.04	10053302.91	18693780.32	9550078.855	7508704.988	9584812.956	11681793.7	6302849.208	7715330.701	11636455.64	10785543.51	27994417.04	25630167.81	21246491.23	9290025.602	13665205.72	15132494.3
0	0	0	0	168683.1725	21853.58348	0	0	0	42898.87255	0	0	0	91271.72946	15839.86426	13385.87802	36099.46402	52553.81519	62003.91516	38360.38151	0	0
[illegible]	167977.6717	893060.3461	690428.0803	1144337.104	805887.2685	1376342.418	684261.1902	504025.142	568316.6034	834790.0026	224026.4977	553890.6423	493387.6043	1443028.841	1195406.93	810712.5351	895770.335	872991.9621	1003033.933	366384.4404	668956.474
[illegible]	2726422.304	7381181.727	3047106.928	0	3400555.322	9858283.016	8138135.113	7164932.861	8019458.032	5418256.95	5903093.613	6588663.926	10899286.17	10119244.35	13495866.86	10711623.72	11830061.99	16287566.85	4688985.467	22376959.18	10283474.44
[illegible]	49195.5334	32732.25824	103875.274	197395.4512	0	425329.6256	0	24914.93694	0	0	0	0	55143.97052	58573.07807	28227.58007	0	33717.53363	24444.32682	0	552701.1857	0
[illegible]	8868946.391	1910185.941	2593860.469	6707989.056	4492149.218	4706976.186	3015544.847	3941957.602	5907961.337	3677490.863	8134511.515	3393700.919	2815223.688	2839828.146	2245482.898	5562840.677	6095109.58	3666059.592	3977911.002	7614260.897	5375784.25
[illegible]	27699180.82	69684287.41	105415467.8	43216934.47	50386518.08	38866897.53	89467582.53	66573652.3	91084419.42	79863466.99	62226734.78	84495479.82	67865748.22	62568570.41	83649479.51	85973002.42	83663533.6	93109851.05	74618826.39	84532843.89	50079707.62
0	0	79463.24671	216221.389	178512.881	361327.7495	576697.5825	77684.00212	166389.4639	106308.7443	53302.80432	1370778.981	387903.0515	301591.7198	289255.0438	361253.9368	512655.6606	283538.5099	227088.7015	343505.4696	246434.3096	0
0	0	196553.1741	59694.84045	0	176607.6883	409911.6738	143397.9822	118849.5785	177753.7166	0	811641.15	130876.8772	607649.2214	197460.4139	670359.1004	29938.07823	194416.706	329051.8732	0	30751.95763	0
0	0	2587221.934	3303490.331	860255.9315	5342239.519	0	4120655.512	6444614.104	3304643.42	2034341.07	3386189.982	2971857.022	4557698.879	4732467.797	3511018.304	4309964.448	4249527.966	1034717.601	3461433.008	2355228.243	2576352.82
0	48181.33498	0	114829.0181	0	0	0	0	0	0	0	265691.4294	0	0	0	77224.50899	0	224557.6878	0	0	0	0
[illegible]	336161.2975	0	242333.2936	245100.0245	99534.06269	201983.5239	354840.4648	62163.59675	120149.2641	160413.5461	185084.5633	329180.7337	99263.12694	143753.4197	166209.51	104350.5274	78810.04451	229352.5054	297686.8292	147566.4563	77796.75606
0	747193.4956	251588.9945	3134855.921	2671122.106	6143654.43	2143322.175	1610868.548	957184.9431	570336.9559	2409062.185	1683813.305	980586.7499	2262922.734	1562620.28	1652865.166	965800.0872	1941805.661	575337.7202	1803753.254	1664047.841	1724347.792
0	45859.37719	108997.7311	248349.7634	337666.031	92303.08462	692117.3772	175758.8347	168202.342	115833.2326	145988.131	146420.8748	28132.874	189780.2523	0	217763.2945	166530.4701	149663.3722	165529.9972	270059.8914	226096.0561	61749.34095
0	0	0	18387.12125	51594.59895	106251.129	0	0	12579.52311	25253.30282	86739.8264	14757.53195	29972.82298	0	0	38375.95442	16341.60197	96151.93285	118228.8621	17234.39946	0	0
0	11599.38346	0	0	0	0	181673.7638	0	0	45431.93567	91479.30008	0	0	0	0	107619.7988	52565.97914	0	172234.5441	83170.57974	0	124528.1172
[illegible]	295304.1121	138276.9041	369239.1201	473974.489	4422619.653	1048255.747	622542.6353	0	1907877.597	315521.2577	296886.9717	427924.6928	634950.7021	864359.4121	3597970.82	1253361.85	172466.3683	531630.689	132749.2163	727035.4412	106062.4042
0	0	0	58138.15562	107286.2065	0	326163.9512	22719.2917	32434.54727	75019.0559	18222.33966	48181.24141	0	66878.25053	53260.66125	167614.167	32458.63969	27925.04772	0	0	0	0
[illegible]	277338.4647	342940.1885	1032890.886	1332871.77	606491.3405	1121457.188	510922.8759	607426.182	480606.5463	504401.5086	471144.0483	406236.1454	671682.9819	613397.2567	1396630.02	982150.6102	967569.472	1425183.883	1278817.757	152367.5545	467255.7576
0	0	0	0	851492.389	85590.10825	186039.2295	0	0	0	0	0	139396.1984	88027.85664	0	0	114126.1438	123596.2307	145133.9998	0	0	0
[illegible]	297694.8891	168866.633	222955.4493	294886.8308	564677.991	282209.4169	247702.3098	168685.9954	51893.93983	233244.6657	85314.1087	194751.0087	223445.0246	300273.1433	549060.8856	370476.9606	317976.0407	0	350046.6115	388649.8155	329464.6919
0	392100.2066	114191.6854	0	0	593760.1517	169503.8151	574995.4489	658510.9299	769590.3923	627502.6732	114049.9545	728746.1881	207494.6178	647044.1287	1171881.178	231754.5989	489160.641	177680.1533	888687.5358	492538.1575	78726.80877
[illegible]	15123.69751	22438.95099	251337.5717	232723.1193	198089.9635	394334.3695	52875.29708	254620.7875	253803.728	216100.4869	52493.87788	194147.1592	23820.9038	171983.307	184204.7458	418361.2719	356657.2665	213800.3716	201049.9211	10246.2447	40052.24123
0	113454.4492	0	0	149877.2052	252496.6783	594137.3162	465850.6611	259893.5527	0	422699.557	526506.6261	142513.8301	217144.2049	436057.4975	476025.8319	0	0	528109.7143	0	145799.9655	83173.043
[illegible]	1527065.336	217859.9654	1724401.146	18063214.13	770876.9073	6014436.351	1365995.254	124731.4624	364661.4923	668516.4964	138319.3526	253739.5182	437516.5438	29662.54933	2771104.176	1213198.618	2513699.217	2089603.864	1521938.291	1131206.632	24287.60265
[illegible]	2248065.366	3761501.329	4785292.391	5703812.208	2845660.413	4235765.638	3942801.737	4292860.08	3596964.41	3431060.681	1840455.576	4630712.265	6722781.323	5142365.181	8836474.49	5609563.587	6282820.748	4105401.981	5810996.786	3699353.834	4312071.446
[illegible]	623982.593	2937032.518	2946387.133	2991551.258	3343101.239	2558951.459	3291813.4	1574412.24	3088215.509	1354573.676	1056391.74	3048714.636	1270128.293	2856569.258	3142299.941	6132326.756	2422048.783	4443077.016	13638254.48	3851549.659	89905.48764
[illegible]	0	0	0	901076.6207	0	499460.9088	0	0	0	262462.3963	0	0	159488.0047	0	226119.2816	0	44670.28105	0	0	0	0
[illegible]	0	140016.6178	199165.0702	267006.2725	0	0	219809.5557	398820.9364	269606.3538	0	199688.2115	209862.3229	163074.8168	326053.513	700660.4778	511518.7216	257632.0036	610274.0597	517534.6222	124113.3051	0
0	522652.3153	0	195934.5677	330022.1293	659450.5405	882851.8321	480305.1746	347302.0465	429409.5551	29508.18346	0	34618.41728	121886.893	361229.9765	244494.382	361399.4468	538285.8611	594099.4912	671847.8568	0	359538.6447
0	16124.82704	0	82559.25733	212045.0136	0	26327.28236	26187.45916	3805.589483	19885.40506	0	0	100598.8261	13686.54191	0	236728.9586	0	258638.5292	0	27076.9236	0	23321.3897
0	194256.204	0	0	76967.15713	14410.96124	13558.27908	0	0	0	0	0	0	0	0	21013.32649	43996.61304	0	29802.03452	38850.68731	37096.6894	0
[illegible]	2089044.364	4957339.675	7560735.802	2880823.918	7897129.665	5740212.512	9649826.154	7717729.462	6973114.452	5527991.323	9389871.173	7169845.673	8187062.28	7110381.802	8389825.469	8463038.642	6650458.536	6458729.276	7309891.679	4379225.323	3108987.509
0	0	0	190769.9439	806439.6023	0	0	0	0	0	0	0	0	0	0	0	0	228549.7335	0	0	0	0
0	964920.046	581422.1927	1093468.742	1091443.17	1729113.561	768033.8099	0	448793.2974	915428.7596	302006.2356	701252.6755	544220.0157	347450.4018	269736.7843	1270565.526	638947.9822	956767.3104	0	655470.5262	931756.4464	284020.8115
[illegible]	863987.8085	3268428.431	3039537.637	2489795.663	1982510.497	2426072.919	4856901.375	4246116.862	2881585.264	2025000.58	2599813.682	2956327.496	4190345.571	3839235.402	7840072.196	2180151.143	2052062.798	4739478.112	2658991.046	1268449.711	794403.0765
0	0	1218027.581	1347121.86	669872.2181	957101.8753	761949.7025	535802.6248	0	0	0	0	0	0	0	610803.8317	529577.0192	687135.6913	481152.7945	427424.0746	1702729.022	347929.8631
0	0	0	0	336090.0667	146091.6097	2522369.665	967772.1381	509429.7018	0	96470.27008	0	632097.0784	666210.9465	0	339300.5606	0	369047.6135	474759.9695	804991.3488	395198.6387	0
[illegible]	116103.8746	330466.22	437059.621	422687.3231	379621.3124	363935.9838	963403.0853	216995.8616	354420.6462	427767.4385	485689.703	349705.0967	833073.9853	671732.6626	175701.76	772202.9585	450913.1221	427515.9603	917411.9838	796156.2528	612549.434
[illegible]	7597150.359	19519132.26	20530137	16416162	15084677.63	12361452.05	22003619.5	30216845.87	15813268.24	20137763.74	11558165.53	19805436.26	17620052.35	20742285.95	15596324.44	21918009.63	31954847.64	18882783.94	19012079.86	22053361.18	15827624.73
0	0	0	0	0	0	298915.8159	42243.22573	0	0	0	0	0	0	0	116172.6096	0	0	0	0	0	0
[illegible]	6757604.263	17873004.53	11718387	11355780.25	9516123.5	25795575.25	11334752.95	17383433.76	13497388.65	13476223.65	10571186.2	11100058.77	14852508.92	16302046.55	16129646.13	21726359.01	12756357.65	12200884.67	16831323.29	13849017.97	14027117
0	0	0	64273.62397	141616.691	46035.55308	364344.586	47159.86547	0	61575.60269	0	41924.73231	134613.5366	32708.83455	48297.08126	55914.63335	112117.9673	66731.99601	148931.7939	0	0	64742.25623
0	0	0	337706.6342	253677.4521	258038.3787	373712.4476	309777.0894	53370.96465	70838.88088	88193.82983	122136.5646	39747.63941	178600.633	100669.3981	107987.4934	221715.1103	512518.8482	188928.6853	0	315819.2219	179587.2522
0	0	143488.4561	387210.0181	495707.2692	300537.1934	418554.9834	119040.7611	73789.17333	176114.5342	46230.77971	627851.9125	240497.0729	256348.295	0	189065.0279	935979.0422	154554.2624	101898.1308	728464.525	585329.8501	1094676.582
[illegible]	609699.9879	1092441.633	906107.7194	1622274.654	962562.1701	1090312.865	277520.9484	584429.2938	414349.9225	598158.9532	1062468.154	851384.9239	489159.43	620369.0181	1452668.288	2309692.183	1947687.583	1868625.609	1755528.297	663824.7782	0
0	0	0	40867.14958	44726.4269	0	29920.41909	40235.43305	25174.30016	0	0	0	25784.02062	0	44325.21112	64180.79268	27823.38274	0	12391.63713	9248.332271	0	0
[illegible]																					

0	0	171162.1184	272126.3919	343933.4411	193430.4548	574385.2961	0	0	571069.2992	0	0	265819.9892	632694.78	0	931176.806	878537.4349	697756.917	1173399.007	828624.2218	489364.4366	643296.3942
0	0	157244.161	6496.907866	84891.62839	0	20894.19823	3453.626132	11385.16416	0	3142.666709	0	0	0	0	16136.34601	9469.759191	23545.52054	5300.276809	45234.06591	0	0
96782.87321	36789.861	305544.7552	180385.1477	1292560.611	103292.8318	2020193.523	0	649515.3856	239677.4465	321394.0909	93286.52306	79064.84319	292182.1486	540394.2804	643109.7477	321211.0354	352215.3699	0	298351.7284	121215.9344	116073.1
53399.37364	0	37231.69725	104908.6972	671941.0154	226703.3904	314022.5714	40773.73187	21362.53965	0	26418.17532	0	0	140686.4265	0	0	0	0	0	23291.68532	453575.052	0
0	0	0	0	86922.44033	10195.27388	33403.31142	12665.9173	0	0	0	0	10062.09582	14398.95305	0	7554.576208	0	55328.90352	0	17619.54551	0	0
794606.8814	2564769.206	4777375.717	7255476.763	679638.3421	4686805.591	1696959.84	4408212.913	7953787.326	10612122.14	3627981.895	1626765.086	5184105.14	2398807.686	11456995.15	6168327.963	3773421.218	1318118.707	6830509.91	3058774.58	3652788.891	4847368.005
0	0	0	0	663552.873	0	55638.83801	70022.1458	164349.2349	0	0	0	399834.4814	522458.9751	0	999714.5135	505971.2694	383422.8374	0	156239.5286	484554.7051	147097.0646
0	0	0	34040.80289	65245.09899	0	597848.3213	30738.55002	36701.68077	0	31909.52442	0	0	224743.0615	0	0	11348.21494	712439.5106	0	154462.9055	0	0
0	0	0	18113.50692	859426.7831	55976.2548	279910.5179	60990.25157	0	0	69689.85207	0	0	56889.29316	6217.727921	55564.69108	48005.30565	338239.9918	80277.75153	94262.83958	0	38149.81557
140512.9356	372853.1215	329556.8754	752250.9183	1223973.872	200206.5913	758113.7303	635733.4502	415550.222	920771.5144	695914.9614	123500.2905	465990.5431	272631.6125	397648.9775	1194207.531	1451675.325	1559145.598	655555.9198	1354515.547	299261.7615	320793.743
0	0	286245.7192	1246277.2	383076.614	605709.3798	334598.8644	581537.2778	667114.6538	375629.1014	98608.59724	171048.3146	385906.6021	474333.8096	0	870307.2982	774651.3667	837575.7477	331775.5781	447399.6027	0	750444.8483
0	0	0	31910.63886	80868.78859	0	176893.6139	0	36319.46534	0	0	0	70404.99026	65868.66472	30696.54733	24410.09814	38545.45961	0	56905.52763	89324.63141	0	0
0	0	234821.4254	423129.0801	524275.3651	1157051.439	243017.4534	312648.9807	522585.4805	371259.7075	1545950.185	758372.5861	0	0	112805.902	291491.6013	217962.3913	141371.0747	315176.9396	2336652.918	243204.3358	155895.3806
1871071.378	556388.9431	3303316.427	4302885.417	2526868.427	3443042.24	2903105.982	4553951.96	3020200.04	2296995.722	2735484.097	2861792.269	1746598.143	2514236.682	4898867.581	4521990.227	3125885.687	3227186.809	3598680.833	2507497.365	1783258.004	2890530.27
0	0	0	492325.3526	533394.0632	1980365.711	394833.4277	0	0	291427.2192	424730.2067	319836.923	261644.4302	362643.0862	732971.9906	1113290.325	432611.2798	958708.7155	1293469.792	0	418565.2758	1528302.184
0	0	0	0	1187953.292	310284.6263	607911.9774	35203.77472	0	0	0	298528.4457	0	89089.32421	292388.3283	271449.0841	83590.97489	312060.1149	219381.5795	70278.30519	54290.96394	0
0	0	0	0	214339.6447	109297.5049	0	63547.0877	0	0	0	92868.61101	114910.4276	91458.39609	209637.3513	177103.3706	4528.888518	0	183979.9484	0	87561.59589	21868.95511
363808.7534	219459.4411	732870.2723	1450679.577	1086922.343	751398.727	1214319.197	802801.8205	730136.1061	688264.5446	851331.2396	571723.6999	780787.0746	780142.1288	1191914.947	1381759.944	1059031.866	1560798.402	1623693.945	1205988.357	165650.7937	616323.1728
116102.6412	46523.8724	94731.06809	75060.41465	389649.6886	148934.8231	372886.7432	214940.9023	100356.1212	113706.055	36043.76849	60966.979	73365.09899	10432.22809	123386.8527	277355.4848	270511.4318	474612.6522	304202.0651	313948.3414	73426.83958	155099.3313
0	0	53421.80066	0	5425.105925	0	87381.67747	20876.80524	296859.8357	160820.8631	66265.90436	59803.68002	50999.81087	11224.3142	127603.4508	175942.5911	104152.941	200918.2797	52186.46459	168506.6229	101392.7014	0
16057.55195	42753.12192	131523.2916	183174.9653	90632.37528	41083.06465	183216.5573	161032.3549	212344.0109	167069.0051	333469.0801	70595.71477	188475.5908	139297.6821	297281.6737	93900.2186	37292.36021	183483.2747	141417.4159	219032.9653	208868.8891	38424.00882
0	0	0	46901.3378	22139.67103	45903.50684	42432.66722	32511.29315	24519.5517	14959.2737	0	0	74718.49741	49944.96423	69460.36906	146172.0396	50799.44127	138120.5523	137498.7599	175927.1128	50088.60304	22743.89575
242800.0794	92280.86246	527113.1701	519014.4818	1235459.208	299752.5043	1370863.783	525379.3219	383835.6334	604963.0867	326236.2237	114728.1531	496819.6883	319868.0033	1097027.773	1055836.857	929990.4228	1425127.698	343908.0704	1458714.363	150866.6277	52670.97955
35829.15393	21977.04307	140538.1038	255462.2237	747179.4668	319748.0417	749941.1293	409101.3662	229232.6393	235570.1885	114508.6499	123271.7294	69711.06609	477268.3724	148139.2692	278302.3841	147580.6729	348278.5818	234493.0234	217348.2664	295550.6728	143332.0861
8421758.898	4003411.993	8558409.952	6234767.529	11293817.4	13632217.02	14824039.4	11957060.49	10683728.49	7854939.076	6733792.177	3556854.334	9808591.875	6167252.52	9897970.234	2546486.863	10190381.25	11302617.91	7359438.324	5844196.097	6107115.089	6018925.988
480867.4823	803019.5979	4010430.137	4563548.164	5711068.413	1474337.299	4942526.987	1713938.965	2449784.457	423629.1048	606913.9765	910496.7964	1976242.43	1604626.775	3338838.463	3001722.667	2781567.179	4196644.095	3781831.869	3739394.011	1426601.858	2019428.586
0	0	0	0	71860.89436	350123.5174	4668.331216	0	0	87107.57779	2149.514908	0	61969.35234	0	227832.1569	9249.477487	0	12923.73991	51351.47145	45112.32487	0	5118.839498
183675.0489	0	0	60480.0883	132403.6619	0	173042.6729	165997.5399	220061.4356	195446.3815	171122.3019	0	0	0	0	0	226110.8854	0	0	105676.2003	0	0
151501.7541	0	791478.2284	770046.551	1117948.158	1507895.895	1590060.21	324115.7042	1317780.631	1387510.831	844462.7863	299786.8781	756154.7139	1493659.354	2007443.655	847760.9523	776185.4934	1292533.22	1483003.399	332619.1586	880962.3017	1541613.562
0	0	0	247984.8205	274511.2721	162636.1234	986427.368	13073.55676	40603.28279	68726.91302	0	121475.8064	0	205971.5725	122581.4249	279265.2107	94047.13813	361061.1592	35355.56233	242352.4974	120059.3925	0
411919.3009	2161758.593	1482739.62	2657363.398	171322.9568	2035589.256	1964104.581	1907370.981	2421873.234	2407987.818	1571533.341	5121991.159	2017810.132	1788722.278	1889404.356	3398231.895	3349060.866	1757647.954	3412793.464	3862247.581	2291916.468	4572869.918
0	0	52294.0552	125951.0991	355346.1483	3416951.232	705197.7705	109388.8681	35523.48299	391361.7936	11922.11116	18777.42834	1519377.006	30596.29438	0	84181.87509	193565.7643	55808.64244	73555.6498	335634.2908	0	63367.14156
4760101.127	4260473.152	895356.6784	0	17338691.92	2876262.468	4069614.273	0	0	455879.5323	3969673.48	170515.4031	0	4838341.561	860574.0018	3664896.505	481587.7711	4639186.2	177925.1842	2376409.198	5851277.785	402085.8822
0	71276.84176	283353.1281	722031.22	882716.6978	558814.2588	702319.5809	410075.0313	356249.2585	589970.4435	609143.1364	715991.6393	272691.6627	568454.3188	447488.4463	545791.3537	872217.8984	700104.3674	555800.0562	899993.8884	120537.7243	349415.353
846347.6671	1128612.683	774338.577	889953.6073	1559650.429	1449684.018	1791882.474	816788.545	1703868.431	1367374.953	1002422.141	2776193.963	2093462.599	2703847.337	932168.8843	3699538.826	3073967.608	2878471.076	2907834.291	2798265.731	1265460.437	1170372.657
271121.5349	1143381.98	814945.8529	1975713.45	1885893.576	1610903.172	2287784.674	1375172.079	740506.7624	868238.0925	1153455.746	645989.5888	913591.494	1630725.013	736847.5024	1150312.833	1107535.916	1537581.424	878609.3754	1212118.791	1386302.991	1328669.056
0	0	0	229670.3454	137639.6111	157331.4164	360539.8587	0	79156.27923	58040.24265	62830.98626	0	96768.88355	63561.08692	237010.4877	427424.3708	159486.6351	27569.68854	0	255996.4356	50390.52053	0
0	0	0	0	0	0	0	0	58653.89686	0	0	0	0	0	0	0	41817.00836	0	0	0	0	0
0	0	190889.372	0	784536.1129	39157.44781	491671.1561	197145.9923	0	47298.21478	0	0	0	160543.7856	0	0	0	200676.6862	0	0	0	190865.8192
0	0	0	581042.8366	181000.193	159351.4012	280869.7422	0	208395.1613	0	0	391830.1157	322842.4831	866024.9798	192648.8348	495239.6781	724331.4055	401664.6897	311148.6422	811029.7142	0	255847.7285
0	454888.0636	0	288443.8578	708371.9506	232532.0338	198583.4122	632178.4956	143141.0211	662654.092	0	54871.94794	148499.162	0	0	386174.8886	0	265656.5063	0	0	0	144313.4889
0	0	0	0	0	0	245349.9129	36885.57116	0	64725.50826	0	0	0	0	0	0	115682.5051	121162.8144	0	0	0	0
0	0	0	46058.18826	159903.3192	99133.69557	375387.7267	185952.026	0	0	752259.7301	0	413020.2146	0	0	350102.5457	419799.9513	0	128650.8461	0	577427.538	0
1019859.095	1449264.902	0	1896276.747	5857000.722	1989239.317	4866650.711	546943.4528	1709783.153	3032129.13	1123145.471	1518109.4	0	2260181.317	1173811.379	233542.4508	1485027.06	1322996.944	1245034.742	1306578.86	1048756.964	1672251.939
212865.7946	50214.43867	433026.4799	1066785.207	785339.348	144919.207	1585180.621	1249716.674	514825.8672	717452.9054	629823.7152	442347.3598	928841.2771	110761.3135	716367.8666	459557.0824	583229.9787	652468.2386	693452.3086	748139.9631	184330.5264	307907.3907
148950.7454	30521.06526	200148.9655	573736.0499	1093726.521	722588.8194	1104738.146	320794.273	598900.3957	448589.1797	447998.6453	344770.1325	330937.4509	414076.4989	604344.6519	1034484.009	735353.2107	1276796.783	464058.7198	900853.7782	288095.9526	187906.0945
6771849.711	8454639.448	9617149.656	11437516.7	14403311.03	13165402.81	19586108.01	12023491.8	12868930.79	10712782.35	11934952.62	10376673.3	11205611.47	14342220.07	11143348.44	17877306.9	14821506.43	15883055.26	13370789.65	15025557.84	11734430.22	9511634.626
0	0	0	0	1535912.093	0	391018.1314	188757.4572	0	0	0	0	0	0	0	0	77900.22055	608887.4033	0	0	576032.3567	0
4665883.118	4009439.388	2135895.281	1260019.728	4799901.734	2301097.885	4394979.147	3143504.111	1353927.366	1967478.554	2466728.362	2252162.264	3502212.633	2605068.586	2566815.316	3625786.421	4359411.424	4482966.207	3549116.823	5079009.169	3054913.357	2886675.426
0	86628.0216	236274.3134	260726.697	446601.1097	548806.4172	252744.4963	349465.4126	275272.3672	118841.4199	181440.317	178006.5378	169581.6033	143197.7919	248741.2048	0	431128.712	87678.77863	0	179888.1468	115799.499	168743.3213
4045018.713	2433971.122	6776115.358	8199959.186	3516408.948	4733272.777	10780375.33	7218945.075	10560077.08	5868592.245	8850376.265	2986856.243	1725439.507	4977186.611	6095096.116	6739536.736	11369044.5	7432296.414	6567545.318	8295596.634	6166155.13	3506824.774
0	0	0	110433.4397	271544.2357	95794.72327	282541.3607	0	0	0	125304.7322	0	0	53057.72014	0	224990.7905	120975.3951	0	258568.8966	140325.5885	45700.97189	0
0	0	0	0	153783.2616	185856.7972	169980.1248	133867.2388	87329.83144	89810.45334	80446.50624	0	0	0	151339.376	114172.0187	0	216805.3549	115689.5959	147479.1217	0	58387.71986
1108352.596	671388.7035	1933809.155	412198.2231	2442565.101	3700972.871	2499058.02	4191207.419	1214564.241	2436997.786	2063026.349	2332333.298	2528954.683	3254012.666	2642637.138	3233018.711	1211903.053	2558686.511	2726006.325	3678333.442	1970681.908	545115.0628
2535610.461	873385.2512	981693.9837	1848556.336	3892176.779	3114310.799	2745876.779	2270209.342	3168660.049	6774120.232	4301457.433	210142.8672	1423736.319	1669218.457	3933672.391	2207631.826	1754867.307	2869378.427	2883793.767	3197107.594	1502375.005	3226498.079
0	0	0	0	0	8021.507082	33166.61196	1456.823836	0	0	49410.75954	0	0	0	115772.4104	37053.41816	228786.6964	347426.1581	86271.44957	25777.91488	123227.8143	36973.92654
2464710.27	2051970.416	3443125.993	4485163.214	6484401.718	5602885.472	8251083.903	5412816.073	6010735.204	3501754.462	3989110.534	7554950.437	4302390.545	3935768.158	3584585.49	4623944.466	6215150.955	6405949.665	6040542.395	7448183.231	2079811.606	2868059.332
0	0	0	0	390055.1992	87708.04651	304238.3078	0	0	0	0	0	0	0	0	82998.46599	0	0	0	89651.26047	0	134098.9785
0	141767.7643	110088.3655	79599.31136	276917.1645	0	126391.1314	94742.25826	89768.64115	607246.2112	115364.6563	250276.4157	244838.8516	165085.0767	0	65502.80799	361857.7116	371417.8359	0	219642.0805	0	37249.77509
161738.3186	0	0	0	170388.7038	0	1505893.842	0	0	0	0	0	0	0	0	132487.5656	0	97123.27968	0	0	0	0
131289.4222	48003.53567	24159.84159	516023.9691	866609.1913	179268.3805	108661.0074	184130.5309	128017.9043	0	0	280375.4051	99009.66227	54850.03181	187752.2223	328745.6326	73288.7369	328481.8407	503237.7089	468702.4849	332741.7496	0
398675.5539	0	292702.5123	415104.2483	1446429.975	905985.3071	1292962.419	777901.829	768560.7964	302180.8259	2133008.975	782241.9367	1011730.812	290090.7083	380463.4342	526960.0757	706806.4286	2043679.517	800147.6793	1608350.668	352627.9532	586675.7602
2347310.782	3686375.357	3534079.754	2352157.802	2337549.123	3095439.845	3537647.062	1633020.276	1817958.187	804412.2099	3107187.174	1190197.19	1414202.843	1839480.271	1543666.512	1341094.322	2601618.406	1079251.27	2452472.833	1936055.317	1554566.162	1639409.428
24249120.15	31178764.07	63675679.05	53650348	51317565.82	63290809.49	49598831.3	54795529.94	50872156	73675958.46	56743177	40758268.2	42443471.29	61200765.08	53593791.69	51022015.06	36001036.82	59628849.08	70885888.86	23624689.2	36291590.69	18563330.71
333710.1372	90377.73786	1301438.094	3054339.419	3883848.194	1625505.869	1250340.193	1984099.332	2345742.216	4415332.204	1262649.773	2571164.253	2090714.421	3103737.876	2787840.924	2878375.253	3583030.699	3592330.033	3786242.871	2304723.722	2118751.661	843506.3432
1280189.563	2371893.252	1074933.731	2005539.145	2283900.605	1537855.812	1905320.436	1609155.28	1970696.971	2152037.931	1999221.605	1718835.409	2064463.19	2393534.78	1748163.76	2242950.928	2394802.473	2534922.443	2220232.854	2065902.221	1763523.619	1914299.12
641840.681	460759.2279	948239.8177	235384.935	814087.5695	676396.6514	1013316.136	556624.3332	1052284.177	892556.4748	1033513.297	792628.2171	999227.2485	1040656.027	676188.4486	942474.8527	1208178.324	806114.775	1115616.797	1467608.057	404332.3041	1150961.768
165362.0237	104504.2484	64805.45292	225182.7266	290307.8002	143980.8091	284219.6499	210086.0048	249184.3556	446875.3417	226607.7145	395490.4384	103629.0729	238880.2072	238012.5105	1279530.23	728670.9509	807028.7993	1100985.267	492586.0981	278460.7617	660658.0494
281276.8854	212851.2282	618906.2429	637671.7814	2168850.553	0	293813.8314	950452.3924	1062684.635	613780.5389	38220.55564	0	540983.1008	423244.8702	682708.9325	1323033.847	578905.1869	1157052.355	928488.9882	1128893.887	506426.0252	0
485054.083	769322.326	500047.8853	641980.5716	1052355.841	817192.9512	519018.6592	676880.3785	825803.4639	455301.6947	438653.9291	849208.1829	591008.7954	997262.1615	356392.1706	579800.3694	896108.3789	889379.7371	1186005.012	1219602.452	381846.6344	685261.8872
0	0	0	0	10255.8394	183454.3493	31531.44233	0	0	0	0	0	0	0	0	0	9624.20957	0	0	0	0	0
510713.7171	494966.9769	408216.5065	490599.2249	140582.7221	178732.2855	260175.3033	522863.1254	628166.2355	114773.558	271280.3022	949516.8865	517746.2075	555179.4338	592070.0434	393224.402	964738.365	787145.5722	824697.63	374336.3538	370715.6994	86290.8809
231189.0707	159855.7651	536536.9669	1484879.639	198736.4632	1991889.4	1103593.448	1435568.423	1290639.32	749553.8164	792138.3834	301872.0287	1451693.525	1478797.759	1923184.486	2847851.888	2086048.706	823165.0257	1047166.547	2169680.155	0	539059.0873
632191.802	236456.3981	0	0	12386441.49	272046.1171	6041887.787	0	278240.9958	210996.8845	1027649.903	0	0	0	0	0	271497.6924	304079.5192	0	718834.7706	177575.0882	0
599659.0009	610543.1509	113537.615	1218224.803	765081.0163	466037.6917	2149357.892	801846.3	1060310.095	202109.1214	1035858.703	1572857.654	593139.1809	1038277.466	872238.2246	1278198.335	903615.4108	1731452.214	1069395.807	1361103.33	1619634.004	1503744.834
43883.73548	654812.3374	276475.4107	1095817.937	214910.8632	2488900.01	63368.0085	480852.9553	318237.2209	140705.9104	889434.2141	1345581.582	691485.8927	563234.8925	0	111705.5573	737556.8566	252421.4688	796634.6746	1378806.926	1015178.67	1281980.815
366695.695	0	220592.6971	108869.971	703966.301	373428.2522	1038656.4	81264.60075	103176.8957	845395.1238	151763.1759	72715.85292	325580.8938	408547.9424	389038.6723	132311.102	513764.6378	638652.0869	429052.9573	332244.8311	432790.9449	251732.5831
385136.8609	54553.19947	0	1043491.265	3111061.169	259142.8326	533210.2704	125523.976	284109.3436	45009.65033	140353.3554	50318.95333	364682.6157	293700.6225	0	820854.5581	233836.6329	788004.8823	374216.8465	615638.5322	332297.8292	118555.3854
372769.3465	0	413648.8991	0	354205.9306	679483.1744	1580652.995	0	471940.0189	0	0	110435.7185	0	504800.0852	1135314.324	0	655416.2819	883243.6963	0	0	0	297948.9763
0	0	28727.88234	0	53344.00524	0	113563.2188	39263.61105	629617.1927	52950.12241	33708.03536	0	0	84513.64051	211383.1018	0	59310.89504	107277.0722	575036.6853	0	0	0
739058.1851	1076545.673	624394.3668	208520.1073	844513.3022	1239978.474	754369.4694	1074222.821	154423.9564	552096.3778	526846.8408	2165756.72	614887.1326	630361.2967	754955.1092	1298757.092	1570908.639	1692843.828	1018560.478	1182504.07	1109522.085	1174298.814
0	0	24021.74887	0	170636.7605	0	0	0	0	0	0	0	0	0	0	25116.54604	0	55191.47944	41609.57396	0	75708.75904	41374.16737
0	0	0	8678.820524	45232.97366	9059.394049	16413.63945	9658.825326	0	0	14068.00105	0	0	0	3436.643649	9392.946059	7207.717309	13650.93409	10954.63571	0	0	0
8290.008117	65583.87884	52625.95524	162010.6278	189304.656	220137.1085	328755.521	94028.21977	92632.81332	28046.51222	145726.5327	251739.4612	75631.89036	244309.0834	169661.1035	383124.0311	342394.83	441611.7036	164097.5009	352861.1294	58201.89254	65992.25498
0	0	0	0	0	40535.01139	94304.66017	379984.3213	0	0	75376.16554	265294.2043	99411.46016	263863.3457	0	155032.3374	0	683831.4371	27887.19686	0	90942.28831	0
135031.9764	443368.404	576359.9707	817852.5125	1952534.988	1237078.996	1491035.876	841280.5451	991076.3613	566993.5964	1343675.941	211716.0415	0	0	692424.9543	606369.1626	786626.5233	1217993.471	394158.9344	884105.0876	754360.1073	667303.7632
1573454.06	809709.2849	1829442.446	1253074.625	2166639.975	1001082.795	1283857.109	1876535.585	2557008.368	672075.8573	1524338.411	1986495.85	1382112.173	2547680.224	1575111.427	1759780.974	1326153.245	1929809.639	1448506.761	1657498.059	3023058.461	1163758.682
0	0	1689185.595	1102145.302	94741.91528	59454.58902	80899.10961	1070659.567	534380.186	0	100525.0731	0	1233679.996	99856.62231	0	2347806.072	2745018.455	2488240.858	2306830.292	678043.5705	479771.2422	594169.2808
86140.4728	496300.815	340706.8743	475935.9621	479113.4728	311493.3314	1244223.573	583562.8262	877437.4628	740682.7721	380648.8365	523085.489	419870.3038	756674.9255	470728.2873	1682023.195	373482.6867	708792.2932	476446.4102	463503.3557	439616.7236	137170.2502
332683.6202	390975.365	1987852.705	831397.953	417060.4695	2465415.777	2853969.606	3299611.373	1532141.944	1136746.887	445815.4484	544358.656	1775945.54	1325508.972	2534529.398	3962974.081	3591214.983	1320515.158	3273834.844	3233320.101	1200282.118	308738.3788
12507.13753	27529.26495	8409.759349	9847.454539	52394.33115	0	50213.6001	45794.28082	62615.14704	134246.9322	33423.46221	16101.4362	0	0	68387.74918	116529.8991	96413.33381	47283.63409	50114.12529	14391.08626	0	0
0	0	3347371.33	4490565.588	12626030.93	15587221.26	12540809.75	15719914.98	5544959.311	14926069.27	3860007.642	1211846.449	6284934.218	1340396.133	8023764.832	0	611894.5562	1830892.554	0	2556241.75	0	2059603.597
46928.22747	73578.67607	0	24641.37471	47272.51146	4416.203023	7367.439608	8323.533604	18937.29232	25644.65833	25745.12193	22380.5353	47955.21085	20376.48251	3773.646162	5560.68424	16219.00299	3929.845102	8145.168601	0	79488.30371	4273.81699
770054.04	445259.8517	316758.6146	333333.8068	533959.6048	0	113737.9797	657025.9357	483158.1885	273212.711	901991.3307	290638.6834	1025280.754	332674.9831	793795.6532	3053921.526	1393464.476	763438.9422	1316737.166	825184.59	1061251.546	549890.8024
2018115.765	4298418.041	3677573.464	6723280.691	4409739.326	8068435.484	9787021.936	4905214.062	4512870.157	10085475.94	7040825.612	1931258.045	1796000.832	1780456.403	5945374.004	3311097.241	2187288.495	4517821.826	1290896.708	2810175.31	2823673.645	7350343.198
1063752.848	872328.9725	3552858.465	2939289.031	6611498.578	2842340.723	4101716.064	1784765.375	3634519.11	3895689.85	3299978.499	1652939.311	1645876.205	2089717.308	3866567.812	4376635.225	2154117.138	2808981.612	3408767.422	2068125.189	2286435.036	2328502.714
432084.0671	0	0	0	287525.7036	0	619106.7884	362435.4641	566371.6012	734941.3055	132781.7266	413639.9665	871140.3685	141133.2533	333365.8778	653748.688	253991.3567	1148536.194	0	1069402.758	398026.0962	336722.2684
68728.70954	270822.8868	471850.0631	0	269351.5782	0	0	263509.22	360231.0213	499002.4713	223699.5103	173994.0325	221001.5339	509036.1161	299896.6076	175210.1403	694615.7086	637271.3003	106864.9935	1509043.183	2028673.708	439967.7277
5344914.044	4273490.638	4809500.259	8547184.97	8822342.676	8166654.064	13956452.07	6983138.766	7582434.744	6264574.687	7907061.93	8008322.411	7821688.526	8208857.255	6580345.48	9772811.595	7196539.313	8933627.585	8548421.616	8027763.751	7041519.009	4329821.38
12947564.52	18366179.24	26201498.15	20414214.66	20451194.85	29353540.52	27357198.36	26791047.36	27484097.09	32338658.47	35105144.23	30657986.27	38097030.17	35229631.04	23064192.38	42117586.51	36483093.69	58755221.12	41120894.25	40380319.12	26803029.74	46598658.66
0	0	0	53787.46622	27962.46366	0	9208.430711	0	0	0	0	0	0	0	0	0	0	8643.171317	0	15814.12029	0	0
2180142.381	1383308.546	1382621.631	2234536.852	5163579.083	2494311.06	3813090.835	1338553.091	1411486.802	913420.8073	3051156.069	3521265.427	1179793.726	2243431.056	1917054.003	2756746.517	2184782.156	8500311.563	1629241.518	3446412.055	4683718.081	1707248.581
6693458.618	3744776.545	6297602.171	3897775.376	9064154.234	4599570.624	6021532.161	3256413.321	1601061.911	4063043.279	3999280.718	3669629.533	973603.8378	2355234.668	5953592.822	6822213.302	4552861.841	4532387.373	5109212.798	2914528.273	4902126.447	2959838.043
4695066.739	3951696.846	10832068.94	11232515.66	5175121.093	6331276.504	6313818.883	9850329.323	6435949.86	10442848.79	15782141.09	9243896.763	9233093.836	9848071.403	12911582.89	16229468.82	14134882.17	11395320.17	12048099.61	12216997.43	6489067.065	5279808.358
237549.5548	171672.8548	559727.0328	386736.6126	923232.3216	540720.4489	713110.5829	452290.3242	636212.3805	545539.5861	447426.8591	419468.5516	291104.4415	303678.4749	460919.0549	1070037.072	886632.3166	786309.1867	508420.1764	768376.0927	176149.1826	177209.6223
94369.36825	215098.4382	310001.3836	469176.6478	514899.1912	762496.9063	552114.2426	538916.825	410612.1019	305562.3146	381146.1105	894324.4618	433568.0092	151063.0049	119242.4744	591403.1685	578283.0275	358655.0507	293717.4362	551324.3009	612341.5312	191492.8656
0	0	42894.05745	0	164912.3274	74558.24127	0	0	64464.24143	38538.63836	0	17161.88673	222316.5478	297273.1232	94310.55636	422612.2194	228500.7974	68004.80663	371520.2039	233028.6071	531803.6542	0
848614.8699	750195.9655	499370.2459	533168.1876	861148.4882	1031221.019	1382725.585	251053.6982	594660.0217	632620.0968	564744.2969	269956.0844	579294.8911	649079.2801	418861.5367	1887110.732	1364164.438	1523372.194	789737.5235	1281565.597	331427.8738	859171.1553
998212.6721	1286773.641	663013.0497	813144.061	2057174.736	1705446.877	4211508.433	1044048.957	579726.6436	536480.2571	967585.6357	0	889886.24	1046856.668	1744574.791	1710748.932	3497675.656	1992351.016	906205.4618	1815134.797	327395.846	825255.7559
491277.6539	331703.5345	447626.9827	148418.1035	600950.5404	1291240.197	1795514.841	916333.3619	786494.3956	622580.5389	639694.0415	1249370.088	443289.3529	549625.0553	1536939.127	733134.6424	529022.9384	771651.5913	1172276.735	685613.0493	428888.9098	0
315012.2724	142547.394	1345470.936	593350.7169	590325.2684	1459488.697	1269823.483	807593.3645	860771.5192	1188377.576	1272796.383	824027.4539	929919.5851	1156412.042	743211.1486	1449627.969	1195664.967	1298649.934	1077518.701	1018346.982	693015.75	448779.7129
1968476.559	555609.257	387336.8805	2295789.321	1794766.567	1904981.503	1985438.61	351169.9791	1747120.269	301392.6953	826094.1209	927022.3523	1393490.042	1117425.086	0	2127256.751	1310453.426	2215090.501	2181568.985	1478690.568	1238533.195	763404.5597
3907412.415	5701601.472	3456580.375	1742345.876	2409298.365	4432876.471	4505317.083	5036726.824	4405224.643	3362301.416	2303830.433	4184337.97	2463937.416	5522764.947	4803293.325	5190289.999	3931545.285	5349939.705	3365044.405	5942813.701	1929388.638	1289908.77
36308.68606	11250.88511	0	54368.23974	11123.0728	0	0	11640.26428	0	0	0	0	4535.987366	0	38342.62894	10945.51626	21409.45859	41728.58706	16358.73615	38722.78618	14584.67625	95214.51306
11543480.71	10561649.76	20807537.5	21617809.48	34947987.67	16937592.33	34079648.88	32865502.08	24751431.61	21981932.58	13282480.96	9548715.286	11565949.5	16275201.97	21853095.8	27864897.4	28675473.47	23260036.44	24217085.99	23239052.68	6871103.145	9568393.289
1697294.821	2167689.82	633499.3702	866540.3744	2347388.417	2026383.557	2095598.844	1421119.443	1464263.835	1332806.131	399695.0808	1896362.354	1684484.42	2300540.974	1779512.63	2493899.738	2966713.296	1998662.867	2574968.579	1385478.546	1334233.931	1367998.637
913575.7193	1420550.313	84510.42087	1835673.505	4838988.74	2601196.834	5617040.637	1566684.183	2820463.019	1059453.588	3949465.068	773999.8392	1543588.385	2104755.382	917075.1735	910097.2749	1657301.857	1478115.763	1165233.514	1557137.544	3841407.101	777127.992
375633.7525	92730.40294	0	395246.863	768898.4793	737794.2413	802314.2398	263855.6379	563213.2936	0	348034.7642	572435.9226	314540.7078	362138.548	596833.5355	723187.9056	559253.2847	1586643.869	272545.0376	592427.317	219042.0617	0
793398.6137	1370901.562	9292257.665	2753535.065	3466934.976	5355159.286	3760474.622	2766520.201	4826384.638	2519516.33	3214488.359	2552083.811	4167610.883	4153441.975	3318583.712	2248013.226	2956571.412	3779887.314	3602868.918	4495307.618	2789147.979	624094.4628
0	66761.13508	20191.2567	58307.91467	691264.3842	189459.9871	101206.6181	104038.9261	129605.1258	31298.50444	139182.5717	49096.51884	109788.5333	134731.3829	43007.09827	546886.8837	500457.1768	858769.9313	209136.7159	949000.6083	72756.25016	98808.16362
0	110167.5317	0	402785.4642	384898.8061	242426.1647	298287.8163	0	59428.49814	34725.72565	149143.3076	18177.75505	274231.3076	166151.9086	215651.9959	282515.1655	298211.2948	80019.81103	0	81784.88643	221073.4603	135269.4142
1132846.695	1624281.559	973358.0191	3003332.014	1721785.556	2406274.901	2185195.434	2349113.006	2548577.716	1757762.484	1771475.887	2028260.578	2075189.102	2832741.879	2849481.585	2814457.753	2883946.885	2989179.229	2423045.038	2600093.043	1306767.161	1410073.787
62689.16277	39337.05739	0	185324.9703	29427.44799	137376.8718	560321.714	0	120781.7683	0	0	88659.49742	97374.43982	45066.0239	39246.54257	0	239672.0778	157615.4051	0	67112.26923	143580.5023	127611.7044
987608.5125	771278.8828	1360070.432	2030289.337	1867908.507	2344036.709	2883971.157	2585999.472	2162019.274	2185956.301	1989208.448	2004071.007	1842785.302	2338422.557	2491749.616	2751106.485	2621240.22	2545714.837	2572195.93	2144958.102	1263919.836	1446290.811
137712.7449	259775.5274	85787.93905	44947.19281	642629.7942	238454.8618	124471.0145	16381.67946	119916.2259	0	310395.915	298923.8656	163849.2507	332256.8379	0	261579.493	207705.8676	396324.4157	115497.2974	180859.8976	200701.7721	333967.1756
89771.83098	82633.73964	92768.15917	163997.1677	190551.6537	207788.4355	154382.1221	142922.5034	91391.32555	101348.6758	153262.6539	102025.7398	0	81250.97277	211678.6915	83138.87361	148921.7124	140771.2593	170658.7729	86434.60745	93832.2771	16542.88852
9123.889987	88706.54615	141022.7644	371441.2753	413042.2962	156600.2005	789409.2249	318333.5039	513004.1041	138118.7805	216713.8433	235672.6154	267007.7496	129370.1261	268706.9651	837788.4593	616394.5276	1074742.647	700605.5886	1134515.282	36539.68105	126639.1128
73602.25002	44359.65307	57082.90826	82869.41647	70956.01508	0	64466.73562	0	32856.04196	208138.3912	296018.9131	45030.74357	39150.71011	67845.53926	48013.03772	172744.0369	58287.49144	203519.222	232350.4403	172131.6508	52572.05208	0
44207.04692	170767.1332	15725.04328	1267095.678	2279260.695	545063.1153	824264.3521	335270.0656	0	55216.54383	192230.529	40209.06849	144971.1048	158978.7442	44874.04179	64756.4684	48812.48775	500755.9925	25318.04366	352015.3405	50847.95891	185513.8722
0	0	263840.2909	370966.2607	1595834.339	296244.3979	685330.9001	0	0	767983.095	1370961.091	0	0	600288.5051	190431.0722	244786.925	496497.9601	108709.2676	0	0	946495.7659	209983.1348
183030.0923	190490.4124	0	0	475454.88	274944.0364	329113.7122	232675.9187	215172.6049	285002.7601	0	302775.5622	60352.69482	295647.3505	0	113994.0996	181432.6128	72886.46389	181811.646	0	191358.1972	0
218487.0553	111097.0121	810817.44	474799.1346	370037.8412	571289.3768	242713.0322	1300165.411	186514.8755	869240.1893	102490.2724	234955.7203	91206.63449	542744.9791	555419.359	605023.0533	2247987.935	962612.7019	795811.9679	461545.9356	450705.5835	337198.212
1690349.324	1092627.168	1802054.431	1823217.181	1612299.535	1422071.644	2535482.038	1855638.53	1652615.938	2316127.353	1506509.163	962243.7668	1655617.543	1349179.616	1074164.357	1818245.878	1780888.765	1616811.565	2078230.806	1443745.14	1351474.493	1834250.028
74209.27945	63659.80912	124745.3835	89891.21119	151787.5282	37712.02782	167023.0075	116111.9025	355779.8786	331997.4647	135713.2387	511463.4503	90816.16976	275222.5549	348983.5921	88030.6296	102724.721	118860.5269	176157.0638	394430.636	42915.38941	116270.7285
0	191507.0342	0	0	0	324390.3953	477705.0964	423368.2122	307175.5852	559632.8337	147944.9738	0	341351.8615	449546.2845	0	372839.5462	161152.7773	291143.5163	625184.2644	0	0	197101.3462
6861423.684	4393446.77	8305475.063	11419106.34	21809059.55	9706589.356	13261741.22	9356239.367	4683852.613	13804835.49	11046801.39	7056841.091	8336075.01	10182445.43	14742575.91	5285937.619	8441570.739	13169786.7	3172012.067	5547148.492	11413623.1	7159625.152
986171.1848	675091.0376	324952.9085	1440909.98	1569914.784	900062.728	582520.1554	670158.8408	1134005.075	2198793.29	9005237.046	786844.2481	1621678.328	1897027.394	1307003.45	1318166.21	938002.8706	801830.1316	1027149.165	805866.9707	2121234.459	971508.8504
1043647.454	687327.9918	1097831.842	959061.6882	1827064.967	1431626.01	1593169.167	2219217.866	2622722.62	1573304.654	2452306.412	1822469.689	1333475.807	1593646.169	2480904.089	2954006.183	1698049.598	1073646.245	1829875.066	2006973.728	1511632.662	1081429.22
0	269994.1467	63421.33694	468638.2019	301693.6289	393943.4641	1477954.519	216772.2373	289839.6497	259221.7961	235535.6314	0	0	0	408738.2037	394008.2934	263145.9353	0	136023.8145	0	0	0
4582116.377	25000294.18	16247052.72	31477825.14	27047095.71	20118149.44	28124736.04	29093799.76	23167296.62	22385086.69	4478416.426	15906366.42	18767531.48	13360489.24	21656520.27	14400264.82	10858206.12	10511591.68	15176048.61	20945593.96	4327276.096	32983608.06
0	0	0	0	0	132307.5905	0	0	35015.11668	0	28550.79248	40498.6588	41329.73525	0	14325.07725	48182.29502	40705.16731	109600.4425	75756.7152	0	0	0
0	0	0	105653.6966	9075245.424	0	583791.0398	106978.9309	0	0	0	126233.7336	0	0	0	130752.3862	572051.2646	0	0	150756.5778	0	114068.5116
4227163.569	3023021.902	7207052.805	6010044.32	8700796.942	7587919.016	11516099.95	7406684.18	10036789.17	9178677.229	6829264.05	6465025.297	8250868.354	9368808.159	12722811.82	12299150.01	13181174.06	14650815.63	15607958.97	11815403.7	4696184.357	6731355.651
483606.1591	187559.2909	0	478215.8176	654237.7449	218553.4577	564905.7795	332409.3454	267057.0048	218562.8311	730202.5236	0	320641.9906	1828758.933	482878.9457	3372263.969	157615.3641	530494.9134	2480256.746	725657.0899	0	148171.7958
780603.8616	702747.1818	248304.6312	1203404.228	1442127.47	733822.3814	3045539.418	1729524.316	861786.9536	1260687.267	960727.4099	0	668201.967	339673.3447	679138.0255	1069012.153	669898.7896	1293638.603	920680.5247	767657.8295	598682.056	8666063.519
61617.4408	428804.034	104196.9224	155991.8847	179805.7011	126734.6884	0	45361.29411	0	0	0	128360.358	0	88908.31063	119874.3571	202461.2837	298860.2142	285893.7926	164768.8203	138030.0449	57403.39544	151692.9258
0	0	0	86396.7276	186010.4356	115109.3431	133640.7483	327454.7456	0	0	95755.50163	71475.52343	13220.24701	591898.9886	247376.5188	235550.5775	182242.4166	187103.7495	216466.7958	128672.949	22925.56672	380346.6835
0	0	296873.5135	0	207066.7349	0	0	0	107733.2096	87656.71637	35362.44257	294742.1128	0	0	0	0	0	43941.82164	0	498474.3785	0	0
0	78112.37547	142494.9145	274643.7262	437809.7201	304176.5508	949758.702	131916.3827	301889.246	178015.3506	196979.6085	127168.9883	144201.6464	336981.3976	291996.0972	388697.186	180936.7851	414903.6325	271509.5499	189690.2418	172330.0326	244529.1049
1595075.135	2859716.14	1850593.758	1468375.288	2342517.387	1715341.892	3061515.97	2224381.934	1253584.227	592721.2169	1395721.071	1213332.778	813857.434	2325142.607	2819264.499	2428065.547	1720159.545	3584697.748	2600551.569	1277501.092	947588.5362	2665780.083
58202.88099	39678.75633	85602.54763	74707.92864	141183.159	79288.78291	78447.51555	79713.77197	127340.0692	169152.479	230579.6215	109395.7971	159025.307	85877.97874	135166.4264	147184.9378	198163.9119	138312.8338	199315.8542	206153.7983	98385.55499	92045.24883
7511739.246	27552795.4	19364577.58	19721818.74	27550226.58	28960657.59	45925035.37	17855919.86	19704036.26	48118558.57	53508466.51	35205776.64	39137689.81	22748950.63	33484058.04	36591887.22	39089237.74	25296126.07	21065109.4	46000339.97	14543121.43	32897246.68
0	0	0	0	0	0	74321.48363	674077.1775	124313.6008	187335.2098	0	0	0	0	0	74093.90662	34352.49839	131406.3285	120714.9694	47784.70686	0	0
30508.77812	137148.2936	8586.231961	42058.54284	233319.5528	70486.58828	0	0	69149.95976	0	48835.61174	0	7139.233324	75354.12916	19468.42417	52690.94626	48410.71011	44925.66101	0	224205.9923	42623.54408	0
8226199.183	7101272.451	8584260.051	13269397.31	14971303.13	11955468.45	19840593.32	11367892.24	9375697.368	10678111.26	13672892.21	11884647.24	10906571.66	13600210.69	8975427.911	18154239.75	15799579.65	17879482.78	16181777.17	18452820.72	10913291.81	11137198.06
138276.4481	89747.42213	0	0	92604.24787	0	0	0	0	0	0	48331.55842	81260.48619	43244.83263	163893.9472	0	0	126065.7511	0	0	204791.9921	0
78259.68498	0	498712.3757	74561.43389	175350.7653	374394.1483	756678.3978	111349.7884	540626.2403	286181.9686	454234.2359	0	0	43393.71665	78461.87255	344940.8921	1666509.836	158527.2517	486051.3092	306351.6811	104478.3261	162950.4618
344076.8583	373965.2593	420727.898	445611.7455	989054.5271	488576.8687	1077406.312	718465.4498	515398.0811	505133.9222	577743.0426	803572.1053	287390.189	735315.9322	435523.6879	1145707.663	910008.7858	1076975.151	403815.0824	1079496.608	963735.0906	554985.5435
553831.9411	663201.2548	1813547.306	3146115.591	1881756.96	1835020.709	2997758.983	1418029.766	1387668.114	992665.3671	985520.1732	1077596.757	2297987.763	2441445.643	1836334.326	6575996.191	4986646.79	3089680.007	7464650.409	4135287.645	1926842.532	1337945.662
8185558.922	10417087.16	8058204.291	7307572.025	13966626.62	10001147.57	10558171.79	6362801.722	7750250.584	9164825.469	6403527.62	10026372.35	8870045.42	11101121.1	8442548.874	15551140.42	11805778.87	16716736.58	13616725.34	6306317.068	6752398.137	8247065.321
594554.1889	484045.747	1210571.814	988611.9525	363710.4479	1669311.109	907562.6434	1498916.915	1785067.527	1761681.297	875339.2823	1616645.348	2409966.794	1198511.369	1963126.756	1383118.053	2135815.244	1216818.182	2113256.478	945211.9649	1021473.86	1016636.42
650043.5374	780595.3216	389934.3113	713978.0997	1623885.847	1514981.046	1574304.057	913386.9303	734130.923	693897.3343	1165245.661	856492.5349	617914.7828	1606439.547	623831.9172	1350090.987	856700.8737	1875071.171	1401135.379	1642409.663	859983.9531	369686.6794
1432596.209	1281605.528	1299858.572	1700989.26	2518744.701	1577004.043	2387380.244	1973239.141	3818447.248	3665221.113	3357916.722	664016.85	2454735.012	1923272.127	2616599.544	3988103.329	3182340.948	3403605.893	4125094.167	3511854.601	1120313.513	2386822.299
459741.7101	1317563.841	238112.5671	884991.4672	3206256.636	607203.5916	1511957.95	891329.6142	850216.1917	185895.1367	683929.3982	963458.6136	601198.8942	1043267.726	700489.4023	929287.1346	1007490.182	1251851.663	912906.3001	1250672.356	837665.7792	930708.5882
1811754.574	876849.45	1613050.288	3068632.28	2945314.569	3522769.135	5264047.907	2848191.646	2279458.604	1776155.382	2493510.036	1820145.656	2395417.348	2026949.955	2472034.938	2623431.683	2799639.652	3796848.279	2806747.407	3014800.565	2160613.931	1539660.152
4315799.208	1685333.139	2755223.639	1533041.076	4091079.009	4261561.498	4796904.034	2481317.033	3950920.023	2629951.358	2118493.202	3485983.621	3870909.644	3900301.954	3167635.188	7132150.229	3908433.82	4727864.815	4873000.084	5650202.627	4332985.398	1285091.075
234306.252	468743.224	0	378521.9729	82308.8722	173879.0224	792503.3824	322835.884	0	366649.869	0	221977.6832	0	64849.37027	125874.7723	141679.4186	264465.9673	133179.3855	0	127252.3961	116004.0451	0
126952.6723	185308.021	257585.4901	137148.4458	204109.1016	81888.24072	39418.56549	0	53815.95168	0	0	135222.4686	101206.9433	125986.9808	0	256528.3202	137352.2728	230542.3907	92509.61437	100554.8268	96721.7822	57249.95526
1105255.972	355416.3449	0	923288.3182	1732717.597	0	133840.4915	132324.8178	178868.9978	561014.2501	958537.737	279994.9005	1080350.275	1754054.098	1160671.967	232389.4471	0	1388558.628	758462.3755	312384.8973	378788.076	1171652.271
2095935.961	258492.7658	793393.4629	4518788.852	1166711.416	2604249.214	4106333.272	171126.4598	1090057.604	682880.8572	607670.7335	0	0	982925.4385	2363548.578	2233198.73	2441328.769	2243906.114	2147765.174	2044859.128	0	802455.0455
10124465.91	9484505.294	8805941.387	14789399.36	14147930.52	9732163.116	18436271.78	9260320.025	6927424.33	12642307.91	10479546.47	5870172.081	8408311.4	9218406.487	10236319.68	12921221.84	12680834.27	16152561.93	12081827.67	12168170.51	7133109.693	10747964.4
13661754.12	20159437.56	38190710.04	53343976.37	22565035.98	17234217.85	23216432.4	34641059.53	48832447.91	54255726.84	39572589.66	37362355.15	51006726.38	25401620.69	53735157.37	22701820.3	58541385.32	66320845.08	50403407.75	26482996.09	47697636.02	53432188.62
14173.23487	3312.647632	90799.31887	113041.7104	121687.0342	266658.5009	32679.72773	313066.7995	70392.61104	301787.1279	0	194357.7324	446070.0879	460623.8145	294916.1941	413645.7386	8674.398302	199371.0619	185263.245	155115.1585	224079.9952	205082.5629
1995780.652	1253079.556	1706053.781	1769300.528	3351855.359	2936592.144	4192375.5	2168677.903	2992291.099	2442561.608	1907285.784	3139055.633	2223713.786	3107698.314	2121249.884	4081935.699	2724042.287	4014060.914	3469890.935	3701880.767	2212054.472	1733169.088
41555.1377	52113.73663	529036.8983	0	680950.0622	0	69056.73046	31497.60284	71873.91905	48625.18669	21561.49037	43127.90683	0	68154.87509	12708.5428	85535.71202	97262.91141	77889.05169	32022.28216	290111.3893	95223.97415	41226.45825
0	41453.2727	0	197972.4344	0	436557.0497	1256271.179	428342.4181	0	0	0	0	0	0	0	0	0	0	0	0	0	0
0	0	168318.3949	641294.6597	691956.8712	332797.2855	272194.5923	844097.9221	905331.2925	706613.4409	966445.4397	1480095.947	1177055.655	2216106.471	0	984087.0587	931960.1457	1920098.285	0	2635579.583	679317.8281	1425070.629
0	473418.1833	0	52037.51114	79903.30653	55705.08464	106159.7836	91187.31584	81731.47128	108210.8128	119421.2077	31750.73617	65126.4237	91646.11397	173342.1858	56184.2376	46592.01706	40532.00005	39007.12794	75215.10495	41723.62911	30095.23729
1021210.529	867561.0093	1194359.908	1139983.957	1288489.908	1224527.264	1665166.51	1461215.672	1731536.972	1340429.758	1183528.311	722166.4949	1579744.25	1460014.569	1919150.294	2340560.177	1685998.301	2183585.42	2339971.927	1858263.287	784495.4427	571349.8744
866353.7901	1045890.477	1095034.991	1816686.484	1269894.274	1573316.962	2332685.921	1677878.414	767791.382	542305.2197	674878.4413	1412405.981	722796.5849	2003844.792	1381144.621	1547809.25	1677023.474	2171902.94	1382018.567	1965430.627	720082.4922	1048059.101
567411.2338	416833.8645	1371251.466	1894189.522	1817331.009	1686406.012	1912725.103	1489146.28	1673636.49	1223312.911	1324379.929	1325329.703	1458488.558	1765658.435	1480084.259	1705531.093	1063363.566	1248136.701	1553595.733	1826855.375	1477629.176	1035169.107
307474.3675	235275.6344	491031.8924	1058872.853	1419977.039	1433408.257	1658176.826	1075414.042	605947.9897	1558204.009	553950.3136	775737.3866	917950.9902	958327.1975	951831.5654	1556993.72	853090.6389	1501580.837	628080.6836	2515562.582	474933.2832	532951.3876
13349238.7	8778973.431	7917615.986	17465839.31	16185504.16	11831282.54	13746362.89	11350921.99	11255307.68	11060435.59	8627951.922	5378939.744	10871032.19	14126830.66	10616784.23	15236529.79	19065689.61	19741313.57	12065224.06	13578007.54	8673642.525	14073732.22
0	578376.3507	635131.7029	1667051.553	1319924.032	1532613.726	1676962.746	421924.1446	613739.2034	625573.5579	626725.0829	1043499.528	62768.59264	743590.8816	748404.836	1685396.888	2069812.78	1124845.409	755234.7815	638536.269	846661.3088	868642.455
0	0	10208.63526	9529.667671	30388.234	0	18973.77829	7791.220464	8523.039444	4743.849042	6999.960718	0	0	0	0	12164.57098	27115.47268	39056.94906	40709.58613	16835.87822	0	0
1461580039	1140826387	845447206.3	92813877.01	114435624.7	1707063287	453608628.1	1451632407	713705683.8	572096120	1353987795	1121704896	1270487247	248421114.4	414349452.5	1129801432	830968925	1091505373	735055519.3	1008826592	1674092780	1191226320
747338958.4	668701028.7	545472290.8	11470906.62	147712423.8	508905701.4	923789311.8	41440.90741	315846950.3	933549409	743747149.1	857787022.3	567703299.7	970039259.5	863506733.5	873852840.9	1169698169	90176.34326	1045606413	1268499918	0	411792957.9
45730.3114	34117.67344	0	34865.84301	159195.5544	34628.69018	44604.27592	52729.70639	0	0	119639.2885	56092.38625	53210.35611	23168.21414	0	88498.75216	35841.98933	222587.7583	32264.70528	80836.74123	0	0
430196753.8	241416969.5	414588171.2	495833605.1	374954616.4	394945181.9	366123247.6	702102250.5	852742243	1030016649	451959528.8	489753396.5	546550106.7	479004660.3	1062312220	599811106.5	524414878.1	455456536.5	442576042.1	285667777.3	709150710.9	248955662.6
0	532236.6841	1416899.407	2290749.756	442735.4186	2139781.978	5096262.847	2503555.885	2194798.341	904112.2205	3145928.487	2050891.823	2255625.924	4686378.813	4596422.22	2018825.655	1526501.896	2201371.042	2552343.601	2834724.355	695653.4203	0
71848037.75	63074963.28	124484683.7	137694308.2	49787880.74	163259870	71621968.98	167633602.4	140039702.1	168618051.2	154321371.8	101987737	134011696.8	137781659	174256194.9	146305487.7	106819762.6	122799849	128655536	112535333.8	110548778.4	114517607.1
1244523.772	1101480.184	3639622.718	3204820.685	2419674.932	2682204.897	2956166.901	3493164.117	3647155.753	2465778.838	2962267.33	1544231.324	3358337.77	4212508.276	5174227.641	4304607.046	3101069.212	2224547.392	3296329.462	2523687.794	1249161.169	1919024.894
0	294814.7048	326855.462	70348.57876	0	394459.0852	665304.1322	720887.8308	718031.2529	785511.5908	387676.365	1197587.798	1165106.127	1121124.846	175223.6857	2281290.474	1227310.605	513560.215	2311931.096	2614219.341	1934102.513	1572416.147
0	0	1401203.153	1575408.035	1823293.716	2451313.638	3038775.955	2088498.146	0	0	0	0	1903247.558	0	0	2297674.919	2914318.79	1497014.367	3838556.424	2324532.968	4104036.709	6862304.135
0	0	269442.1466	287322.1166	241110.7868	673469.4278	1808018.083	0	797351.6223	238330.2302	649539.5314	499332.8192	0	1096744.919	0	384034.1239	243720.2661	1113845.997	945633.4579	198100.3455	490058.2981	510447.7403
0	331260.942	228529.0807	0	842683.1128	82598.7144	636605.8115	102345.9012	310426.4294	107785.8228	292518.5661	255442.7511	419339.5494	335194.8623	167667.3722	162812.7668	0	116041.5991	445304.1912	0	186900.9121	229585.7732
731287.5285	383128.3771	1328081.075	935811.0569	5849759.57	1799399.141	3074174.693	370642.3949	231735.2824	1929996.672	1812042.476	1639025.959	826211.7896	2651312.166	185086.7056	2919565.84	3665103.216	3701948.88	2671390.498	5572485.253	2654588.484	3096791.433
0	0	0	152384.8854	0	0	0	735963.5205	167947.4992	0	153755.2102	0	0	0	294175.1356	629366.0657	751715.7747	693399.7865	254216.7877	1139204.104	190183.1634	167386.8871
14391781.28	21266731.3	34408059.02	48256683.48	32510589.48	36214870.6	51706995.7	37604069.46	27006632.83	29747021.66	32275092.28	26790343.77	23016364.21	46423531.2	43314042.43	18322642.69	27078928.88	32020065.49	32248900.64	44515043.68	14866025.8	19075820.88
9087748.538	14898778.52	21310296.32	22334343.69	10331564.43	21571760.73	13898720.61	29675032.18	11408328.72	9315694.622	12624269.64	12221434.6	18129852.58	12961878.81	562592.4582	26384275.32	15698396.98	12093777.91	16812401.5	14196425.67	10930153.19	8148249.485
4402203.885	2776653.438	2040843.093	1434493.962	1238070.465	3573038.988	4182575.294	3688681.853	2985310.986	1003769.566	1575292.381	3490790.093	3424892.736	3804941.781	8698176.093	4581824.918	2410014.239	1949795.89	2507158.688	2059831.304	1326569.235	3037532.266
0	337689.0995	0	419042.6075	0	0	1105880.355	867074.4774	126044.5549	348823.4306	0	0	1174266.2	357038.7138	325611.0121	380440.0761	274171.0278	304800.5884	422362.1459	303068.82	297700.3631	525588.1734
3519153.578	10993736.78	7685390.739	7437039.613	10169921.66	6919758.805	15606088.77	8116258.817	3916870.446	14313987.24	0	8731698.996	9274643.088	11749296.4	0	10227549.37	10176657.3	2887862.724	11222448.86	5824547.464	0	8849877.28
8479319.004	4051126.127	9736461.297	9107038.5	10928705.34	17820882.88	12844650.47	8561762.212	9239580.989	12449423.46	15173171.67	22783122.7	12746469.1	15855857.27	2589991.933	0	14688824.57	11139374.75	9930339.309	8047115.833	12258940.08	8794966.558
0	303278.22	0	0	0	707881.1868	0	597289.6093	774517.7289	948752.74	655643.578	0	0	225710.2682	896448.5502	590814.7337	0	315451.7328	291916.6247	0	0	769737.3724
0	0	136523.3924	177403.2492	643997.1902	217330.9096	466680.3357	357422.207	221312.2691	172999.2631	239951.4344	124159.3476	250111.5154	384751.0762	234741.3046	587601.1611	671332.9732	661527.2835	565768.8243	639224.9471	493169.4206	0
1014006.943	1059913.231	1203242.676	430190.7348	985004.0822	996603.8925	668125.7542	1332732.196	1696255.588	1331365.487	1527927.039	378522.7804	639862.5851	1415836.711	1893785.951	1348800.107	2438750.966	780217.3096	890781.5954	1788723.524	1661985.739	1317960.57
16423660.97	8345342.791	0	0	0	7292182.696	5497232.129	1081558.884	1690458.219	0	0	0	4698683.967	0	0	2869309.915	3803568.417	1480918.923	6945042.086	0	5536735.164	551318.9062
0	0	0	320373.8535	0	704666.0804	0	173590.2485	145664.1689	0	207839.5035	304076.202	0	251756.8349	121339.6699	396915.9693	719667.1907	418873.3207	410902.2251	393149.2657	0	0
52150.15346	33489.35583	95574.80342	122143.4654	146443.4027	107679.2769	422249.8199	62478.52067	81385.58107	0	39027.65569	0	80147.19401	245296.4001	184036.7561	74653.24933	189635.7469	82662.93544	0	76752.79237	0	0
0	284293.5206	118033.1478	87453.4222	200904.8914	130541.6264	198857.867	484809.7913	0	164901.8843	0	0	140358.1069	0	367681.2422	587025.7108	232991.2557	0	270857.4103	0	176729.5462	0
0	0	190580.3476	78403.63745	127749.0989	19141.70001	93540.30898	333656.3748	418974.8	90542.89093	346230.5855	0	100806.7521	110966.9646	191540.7682	290019.0557	509303.8534	21635.65237	93244.93474	538648.8761	0	80782.83574
0	0	0	1534731.859	3554306.047	443045.2827	647779.3347	886381.1163	1791241.015	1699044.378	3784277.402	419990.403	157403.0123	2736292.076	1641041.836	2823155.46	3274911.322	3767520.637	166517.6054	1043712.59	3043034.103	493095.5989
0	0	959993.2318	956909.3758	959926.7731	940124.2263	1442992.138	766151.2064	1048586.214	1066514.635	585327.2386	721261.5182	680357.5403	968354.2591	870268.4496	3060013.031	1262373.28	1415620.74	1956913.405	1902450.681	496163.5518	397802.913
4062879.817	1783094.972	4724362.376	7918556.594	6435781.254	4171079.751	6892976.501	5610061.758	4307647.71	3230931.449	4748845.48	3547979.721	3375939.843	7092914.545	4607535.006	7038871.425	8063043.593	5158444.745	9346695.861	7665518.364	4006275.15	6298456.564
1362877.297	5589700.102	1834007.913	2051310.735	2978390.407	0	0	3637366.129	1079205.701	3288309.016	0	2880038.67	2892207.275	3846950.532	0	2314218.686	1505535.434	1492725.621	1654169.888	1258210.577	3975527.258	1741716.84
20982329.8	22559618.63	27616366.66	42983145.39	42336855.41	31069084.75	35498193.94	26960384.92	33743637.63	44253071.23	31448594.11	47597044.57	41566995.66	50836746.69	26746983.56	42019876.59	43331480.94	33621126.33	42377186.99	35084974.87	36197391.22	29418968.61
0	0	0	42904.42929	44347.86036	0	410313.1195	0	0	0	0	0	33325.20665	82820.7811	43256.14994	122991.984	60598.29905	180966.5727	50794.1934	264393.1843	0	0
0	0	138770.6582	589965.8031	102278.2844	385107.8072	181182.5083	0	375104.4575	0	249873.8965	56870.28242	122055.4248	433733.0862	296488.8357	125793.3513	40837.75966	374371.5272	471534.137	0	36268.62419	0
456418.1508	108281.9863	434265.1814	972291.8721	1128361.017	528795.457	546998.2408	1533486.653	1401663.957	1673823.424	760989.0715	365061.3381	234199.5981	1068172.275	1251454.349	1698543.417	1375437.012	1210676.03	1562026.161	820723.6158	76243.09951	368206.5145
25902291.32	19418549.07	37977747.78	27873683.73	29308711.66	26032811.36	32860886.07	63675893.27	55049045.83	29069801	43038519.49	20880217.34	38821351.08	46062797.27	51297329.26	63116928.72	56182908.42	63765770.73	49933798.43	51654807.72	19261723.42	17221728.2
42163959.08	37768691.96	24894183.97	36280645.71	19142559.21	22503219.9	48473630.38	36711744.35	23875733.95	34928802.35	25453134.75	11629342.42	19320445.66	15195136.57	17880239.22	20659070.2	72728957.85	34519284.59	51167330.1	36242999.06	24224403.23	39516958.66
1332567.871	3114694.336	7824448.43	7940596.749	9142520.091	6062356.954	8758271.157	8656911.131	10246556.8	8693222.637	7420825.141	9913239.707	7336309.012	7141836.877	9804597.028	12247044.75	15646870.99	11441551.56	14005847.65	14092885.16	6772667.578	9041305.224
446431.7629	794167.3185	293900.5236	523241.0472	234465.7555	422600.1216	236807.7392	0	464122.5759	871379.5825	0	0	0	371030.6583	704503.7142	407457.4413	153516.4835	165937.7604	522118.5471	749657.0408	253937.2131	537660.9027
1341460.737	263962.3308	2882528.083	5537310.478	3427961.25	2675638.257	5686496.858	3387847.254	6610134.191	3837510.355	2991879.31	4493950.241	2204462.239	5407311.304	4839285.372	4090887.728	3828915.002	4132795.732	4056361.446	2434432.925	1861822.307	4441652.411
0	0	1790952.573	0	0	1521891.374	1987431.581	2460790.715	1030868.277	879367.8025	0	2422241.499	1784919.653	3389011.55	0	2442958.829	1511936.949	0	0	0	0	1286691.162
381438.1603	202594.9886	184323.228	226121.9119	700230.8657	859293.8174	1100270.617	470256.7838	682138.7617	4255877.754	464975.4057	210791.2969	748949.4931	796413.7658	1558891.932	2000583.897	1379528.5	1038910.206	404777.4385	623514.795	519924.582	193659.7603
1895490.713	2427056.591	2688476.368	3630296.959	5816787.586	6427238.615	9581776.631	235534.5321	4856858.79	2154583.486	4518141.935	410996.4925	2674395.819	1921267.429	2886494.335	0	2020288.156	7361550.755	7126452.332	9515010.469	7405866.742	2176944.509
0	84604.20892	0	0	176813.5877	181728.9182	265853.6227	168060.3491	156366.4158	231588.4178	142435.3278	26155.71687	404041.5424	133402.0101	109871.4797	683052.9113	187325.2244	39298.15205	702184.6588	228589.1942	454696.2564	29820.04733
24426348.6	19055564.89	79255047.81	111728486.9	27259154.95	61472955.87	27588843.03	65922965.23	65784905.59	57433389.16	88893378.43	74099022.5	81242901.83	62992377.29	59333157.89	56107733.18	44028923.45	67558078.76	46950840.03	52986206.97	73509526.85	41151874.23
4554833.395	3691706.584	15675700.67	16389339.79	18704679.27	13414633.49	14474545.08	16861639.39	14775137.09	11860389.65	15947404.08	11790312.81	11689355.8	10211763.32	19683879.83	21779745.16	16635092.36	22294569.09	13330520.56	18549822.92	8023515.09	11005569
8639607.433	8853838.984	5944251.522	7601114.513	9510524.664	9311782.204	9295930.007	8896970.779	7677179.649	10212247.8	9503990.601	3431631.392	5612992.998	6282119.844	4947031.672	9577295.852	8878321.734	12078815.77	7172801.46	8627702.047	9797589.766	6902170.262
0	257218.3217	641384.0674	536239.5107	210704.3457	151966.4879	87847.33008	820722.6099	300729.2802	385650.0875	289226.1003	809605.5655	451354.4328	746233.3599	0	430669.5183	714375.4971	0	954530.1248	632555.4978	670395.965	357163.5864
24786946.79	40062880.46	22497229.45	27935410.38	41770387.86	46080025.02	35943826.21	34786221.7	34043470.96	33255919.48	28820388.59	38863234.42	36079688.4	43217373.49	32626818.19	33553255.37	43827056.43	37353200.25	37926958.17	37716488.44	23909797.72	46716844.75
189998.6101	287538.4552	0	225804.0659	0	1058580.612	535383.7056	0	204296.5461	0	0	74591.04906	0	144119.5363	0	570774.8968	87924.53713	490290.3854	447258.5814	257235.0718	617585.713	419748.2034
0	0	288772.2145	302399.6302	357781.8589	376319.3195	316903.6711	181886.4848	221606.0734	399610.6336	176989.966	183933.2878	0	73066.97549	332545.0555	401288.9719	573332.2581	369438.4466	101720.9968	176155.8848	125700.8786	227414.0972
2531878.629	1927336.413	1815630.183	1855202.329	5920071.882	1266691.655	3891133.373	1995752.932	2625779.714	1685705.664	1397264.474	1339410.122	2264320.349	3100186.583	2326827.237	1324242.048	2428296.115	1140820.77	3054750.904	1401159.168	1686545.522	2481332.399
2240004.346	4966665.879	6551747.868	3413202.638	7170953.491	3322640.712	9547875.174	8227183.223	10763418.12	11717811.72	7473897.197	3938353.246	5170482.984	5829780.11	10179467.65	10147478.26	8154098.941	4300661.056	5121751.231	3734829.886	4989814.841	9582512.983
29238.14545	156581.0055	67848.80294	0	341680.845	235094.2726	767289.0123	224440.7378	314179.8702	116214.1531	377731.0368	116878.3923	43373.53698	147192.587	116139.0133	277994.7201	435141.2128	96952.78099	155730.9716	645171.3444	196586.5748	30236.97905
0	68070.18885	0	372372.0346	4263086.339	193709.0928	2048815.732	259288.8704	0	118377.1558	336514.0278	236038.4479	155580.1312	255260.5943	0	249452.2004	175944.7947	1250663.475	125322.8891	307556.7957	324680.6205	205682.6748
0	0	0	0	0	0	0	0	0	0	131426.9173	0	0	0	0	309439.3799	0	97454.90719	0	0	0	0
0	0	244308.6453	496922.8426	328525.6642	273837.0236	175249.6595	767005.1613	636948.9629	143248.5066	308329.3984	714314.4457	507653.6784	226347.1756	182979.2366	171053.8561	138202.3652	890295.4433	583591.9477	440021.7298	491899.4141	0
4568792.776	82232.89766	623594.728	769233.5609	477607.2375	0	2294387.227	0	1886359.342	2157115.92	470701.9444	761104.506	456564.7144	435352.0199	3538109.106	1356735.455	877591.589	1154763.631	726191.8279	840647.6431	0	865437.8835
0	0	0	0	482126.9503	0	1029508.4	0	0	0	0	0	598321.9257	0	0	0	0	295613.4586	0	0	0	984583.0698
5126284.898	0	1740773.075	0	6036601.417	9077917.743	8083852.954	9035067.185	8082052.868	5139647.002	6172427.206	0	2289600.824	2374408.42	4721488.736	5377240.845	0	4749309.605	0	0	938675.0153	0
1679113.231	0	0	928401.6292	701718.378	589650.7457	724665.6023	732847.1783	1274589.277	2428198.292	715190.1318	2033425.441	654010.0742	800147.3326	456076.8208	1456805.694	640265.2731	1838516.655	0	0	817155.9759	0
97019.78262	285200.6343	4054309.944	53344.32628	208140.9966	380579.7528	658351.656	322014.289	264468.2455	663430.5748	373321.7689	137347.557	0	171424.4905	110263.0624	430237.8953	93421.04265	297035.1828	221462.0907	278066.7477	155462.974	17469.91952
327130.5279	444604.1309	3705592.369	1859280.172	6977490.619	2024162.312	4650058.399	3121110.986	3980126.642	5830138.099	3259248.941	881629.3895	4839315.148	4255761.22	1332139.051	2722351.213	2194096.188	4054409.982	3110285.118	3343420.029	4414426.624	3084837.126
0	0	648766.6847	1405858.144	928851.9212	666463.8651	602682.4138	758458.0992	744725.738	0	0	672190.9203	0	0	0	1142401.804	0	548302.2423	323383.8704	896191.2384	680210.7403	0
185629.5269	222759.7598	603323.7178	231227.6286	780712.0281	287332.4241	1061755.929	1057195.426	766905.6482	747855.5088	289993.4972	551928.7202	831373.2909	1216264.227	812169.6285	1419483.047	506509.7627	791166.2726	1454757.021	1359774.413	619121.0615	800453.0108
24627.23901	0	0	0	57728.2115	0	116606.7589	0	48319.90031	0	0	0	0	0	0	69600.26363	149782.425	113490.7292	26774.90487	27540.4924	0	0
13844125.47	13647677.86	15176135.9	1402556.627	22438279.25	15256042.2	20897679.27	15163148.8	19889373.39	6437172.376	16913261.44	2936894.009	1029963.832	7431112.628	11305532.69	28304209.88	30785825.81	35457628.46	23979924.61	5578651.796	15663463.86	2125522.928
313461.2644	0	287963.7657	0	214055.3636	225788.8564	173800.2486	215821.4297	565075.6345	665733.2159	109785.5724	311464.1469	113395.3823	410086.9713	339696.9601	877231.1457	794171.775	716027.0585	760051.1759	89165.01985	14740.05486	0
143794.0078	26008.00287	38710.34259	157967.3169	293881.4399	33694.10146	157840.7754	0	90839.26361	0	0	63399.02137	32902.84046	412583.5149	154840.4604	245053.7831	240245.6141	297400.7979	353230.5514	260513.5599	0	0
37616.04421	107312.4174	375198.8155	386315.5558	281539.6764	220779.8316	452615.0899	0	602996.6035	407214.5252	327470.1458	255800.7031	298909.3427	304075.9503	263692.6641	436405.5607	287380.8804	870138.014	0	436638.2414	0	72978.79418
67764.81993	0	261545.1863	0	0	182017.1072	300313.8947	0	238929.7952	0	85837.04276	342885.8482	186056.3113	93311.6481	278930.4333	234391.4084	270861.3574	297768.4037	241410.6391	0	0	0
0	0	0	0	0	0	0	0	0	0	0	0	0	0	0	0	261858.837	289003.5967	0	0	0	0
761842.3558	1131506.909	5653189.201	4993446.102	6740681.947	4053414.769	7420906.992	7416062.386	2949393.453	6255817.96	6163629.284	2504544.096	8484030.653	5260897.633	6544925.476	7576151.83	3833147.928	4473715.249	4510139.605	5210945.841	3891563.533	3798046.121
41219.78724	0	0	186096.662	115723.7622	0	281203.8449	0	0	0	0	0	186934.8602	0	412607.2171	0	0	147104.5694	0	55768.5183	52738.52757	0
0	0	0	0	0	0	0	0	0	0	178653.509	0	141079.4264	0	0	0	0	373093.7702	0	0	81172.99585	919545.7622
0	0	0	0	4975.048894	78603.59192	5110.060792	93685.83583	7648.663979	0	22938.28907	0	68560.80375	1456.823836	17363.40854	10383.8493	0	0	17449.08531	16586.28665	23495.90037	0
0	0	0	543091.8392	424947.8263	0	2906354.912	0	591349.9609	436463.1407	450363.6486	443519.3223	924500.7952	884875.0591	606519.1634	0	96914.79288	229722.9154	378288.1951	484129.4951	1033522.082	0
3866074.906	5775028.441	4166090.493	3859829.285	3057547.197	3136606.179	6055945.815	4145427.759	1616937.72	5884084.85	5447319.242	3810364.929	2608535.53	1848388.332	3390525.585	2554107.217	2634819.262	4201112.061	4289467.694	4615266.344	3605782.267	7107222.305
5980034.12	3587511.079	7777799.561	5622428.771	5649781.324	6863870.376	5659458.56	6296716.311	7613737.906	9273212.643	7611290.164	8645057.981	10136714.8	13707532.29	9871682.643	13196833.33	9518422.905	7516300.492	11318494.55	10244514.03	6535139.674	3679605.844
10830198.86	7067389.022	17765123.69	21694431.41	18953602.83	13102442.22	24055155.27	12870217.05	10827041.73	18908647.56	13025133.45	7927695.941	14256883.13	12626709.71	13941657.63	15369337.63	26870343.62	27470082.47	17174780.11	21850207.79	7784045.233	18487146.63
21510304.45	12147039.61	28341774.19	15288510.45	21884795.45	30867219.89	26475964.43	40074043.35	41794520.99	36308366	63162785.37	24900805.44	32251976.64	23176232.74	53852510.53	39266269.06	31891510.7	62908119.35	33317778.52	52599509.84	39375582.11	42014786.87
127500.2398	0	0	398387.7145	135616.9365	249100.3991	63102.63744	0	0	0	0	466933.9495	27560.19984	269784.8317	0	0	0	344347.8861	0	732896.2629	256902.9307	141886.6381
0	0	0	0	1725210.395	0	2131906.704	0	0	0	80631.06912	0	0	79980.15508	0	194620.9697	0	174694.0795	586183.6674	0	0	0
2149205.913	3196772.826	6271313.282	4712811.639	7126380.633	4224722.57	8991064.672	3994945.216	3404461.159	4037977.139	6115063.361	2220274.234	1833381.946	1164085.469	4428789.578	5617217.582	2203491.657	9746035.91	3958080.176	3237093.908	4001093.768	2875332.507
1192271.836	5738309.459	1181189.939	664770.8539	6421705.57	2879803.215	4976992.267	923487.8055	0	1625006.398	0	0	0	127092.6632	1840521.747	1970315.495	185311.5375	4104850.127	1513705.383	2537410.444	2182431.075	1760529.757
0	0	0	0	98994.93314	0	84183.67653	106184.122	0	181658.6238	0	0	0	0	115176.1147	274660.1335	0	298404.4518	114703.1906	106748.8568	0	0
0	58378.2865	492308.9071	0	0	179862.7044	94778.01112	85597.39482	409168.5889	633359.2669	0	331124.6263	253021.8046	0	468578.2364	425266.9872	304072.6747	439248.253	0	256331.2166	0	0
0	275532.726	220245.7347	0	242016.6491	506329.5316	3797252.465	255815.4356	369157.9231	642232.5777	661375.6975	0	0	888929.1258	1244720.997	1527522.721	784175.9067	949088.6196	570772.4874	454477.3352	935271.0604	1552886.15
0	0	0	97870.60605	975161.3158	0	207169.0081	0	0	0	0	0	0	0	0	0	131424.3139	339036.1609	0	158990.1256	0	0
9923103.581	6910475.481	11671498.46	16556981.26	7230832.541	13544802.01	15751192.61	14808597.44	15155632.73	21725172.65	17063502.99	16351804.72	14404202.98	16861019.84	10337584.58	26234656.92	18498825.58	15295282.71	22990650.41	17182630.13	6061941.693	4969100.839
28263564.86	28712298.43	48997028.88	42342615.12	10552590.33	37735380.86	24842105.82	48522538.01	49097892.78	34686443.58	58712811.61	32334808.17	27708120.91	39282590.06	39235733.23	60446261.11	42845103.27	53277553.76	40620309.07	43498980.99	30300862.62	25598231.13
0	0	0	0	50797.58311	0	0	0	158090.7546	50534.64999	35896.99281	204998.7954	0	58743.82259	0	76148.04493	108050.2295	80810.33374	0	35804.96305	11548.00391	0
124315.5054	0	439435.1327	720378.061	193958.8518	336127.5641	224599.624	222861.7563	0	0	0	0	244492.714	0	0	0	0	0	178856.2993	0	341427.9013	132642.5807
1168558.803	984515.8931	1236272.297	2425410.86	2392085.657	1426702.91	3676811.423	2113035.298	3808632.408	832920.5213	1833305.697	486862.9615	1132318.443	1883333.423	2746268.423	2198415.395	2726952.187	2839012.803	3099506.951	1790629.035	1108237.413	735869.6828
3719667.069	3192031.16	1295177.367	879184.235	1281829.736	2859441.549	1516689.135	1617774.039	1923227.463	2349537.389	988982.4085	1241764.956	1178379.578	1999291.612	657273.8175	1896974.348	1942069.035	976562.1949	4684172.374	2329566.165	1924497.029	1791574.661
209554.4159	168270.4223	1093805.027	1275462.144	375544.684	1248527.952	898905.8441	1547307.625	206632.4413	294935.3448	805490.1211	0	372017.0173	778413.1529	1046676.893	603045.1286	391668.8264	458167.1226	2401870.074	1024599.509	180772.3215	244875.3785
417811.7517	153679.6258	306823.0734	570984.3371	495211.3302	879846.6288	1359264.815	513315.4657	532491.4581	954074.2453	721940.268	165215.9294	354548.9246	325030.7898	568210.7129	750924.6172	364931.6918	910961.3434	614920.0504	361104.7959	131894.7708	98221.11271
4618212.307	3911250.879	5902397.219	7509627.048	8925452.561	7323332.633	7296890.598	4369728.347	7329794.259	4840131.923	7391242.003	12498991.89	11873896.93	15096803.89	8570507.151	16613172.09	11557592.98	10772961.72	15106734.47	11493837.82	6628131.239	3378591.88
0	21342.71305	61935.32791	0	143871.0926	168252.8833	303537.9214	9494.753826	0	74351.20484	0	0	0	0	250693.6724	148909.4516	134293.7123	322377.0897	58950.69929	138230.902	0	55219.23302
5541525.089	7924837.938	6665413.784	8534990.878	18587619.84	8338587.295	17158301.66	6416499.388	4863151.149	3243221.537	8663924.54	3854921.51	4394394.497	7266071.864	3268034.841	8452381.556	6618762.394	11410089.39	5988019.607	10909594.62	10672606.29	4400691.561
128306.6026	621465.2976	494823.7493	554666.9823	3875388.771	1234464.056	886224.2886	680009.9892	738077.156	653753.105	975620.4086	113422.4322	262020.0207	387622.5977	345899.369	666917.2748	197047.7353	1429374.915	238295.055	1568950.372	1074981.591	190286.4479
126158.5882	158368.7879	615770.2602	898143.7322	1058727.363	645748.5402	686648.8988	567655.6562	1146567.426	782866.2195	403203.3128	489933.1897	427367.7249	254175.037	684186.088	1695344.011	1087678.719	768019.7852	891656.3777	327639.3304	505165.4277	614817.2353
0	0	0	0	0	0	240017.7446	0	0	49801.13238	0	0	0	98516.37885	0	91982.71004	0	97820.82514	19618.85692	0	0	0
0	0	397466.7389	0	40251.39131	151502.8856	262717.1863	214627.9868	91579.04808	281881.5999	151535.9595	300191.9294	390397.5686	307840.0518	0	505879.1058	626497.0359	288001.9311	847070.5453	401940.8889	267663.1475	470898.7013
0	0	0	933689.3621	428122.1969	95452.71869	0	0	0	0	0	0	276199.2698	502308.09	0	718747.3788	0	406975.4774	0	1676965.551	913106.6045	0
0	22142.86143	639852.1561	1617837.82	831710.9306	0	723950.9502	0	0	1715272.727	997921.7117	1247856.066	560979.8074	1113634.513	0	1319347.97	1921509.437	2004817.117	2704977.483	1055844.497	907618.4422	1026546.39
1129990.399	424042.2235	1461061.895	2470604.342	791110.5564	2468294.586	7143853.029	2240542.454	3480194.526	1478956.578	1267150.721	1058769.852	1696313.107	2580719.679	2061522.343	3842458.176	2615931.899	438775.8127	4334402.224	6384911.875	1776526.723	3005992.074
2734535.014	12764131.43	31244904.78	40200166.14	19584737.16	13039446.69	25419563.8	49525500.91	27677898.9	17429690.52	34197763.93	56863666.46	30663805.72	45944551.39	24965072.51	27003852.11	5975794.376	30447649.07	33602239.19	53882566.23	27363616.32	11062301.42
1377523.773	799667.8144	6218198.839	5607872.422	3538809.035	6781111.929	5270471.524	4719867.502	6162713.825	5459476.644	6063356.839	7337084.243	2794974.359	6233311.118	10728998.65	5011660.817	5797337.819	4654809.831	5133212.194	5409943.934	5283210.306	5057922.072
100485623.6	66703545.06	161165999.3	82553225.24	78712059.19	113652712.8	109279520.4	165536451.8	142302081.3	143932489.7	96679723.22	79057144.49	105253269	99818132.67	240274235	210742769.5	157782821.6	112429578.4	150216932.3	92674428.4	62162514.2	82088919.81
0	0	0	259662.7643	362412.5047	1663375.893	2561668.951	2034272.149	708303.6887	294156.082	325773.3078	0	1090714.243	2311268.143	0	689803.1274	1384930.315	1168147.517	1481518.677	543185.9605	366899.7921	0
781647.9065	533002.756	703681.6327	2075099.689	5501160.165	995495.5119	4620696.345	2416570.337	2071465.09	1493985.112	2499403.814	1802198.787	2003107.981	3169722.436	1550422.766	3590207.128	3708893.147	4377013.91	3545095.257	2641337.191	2002076.273	1288305.16
0	0	59097.0316	149568.4667	0	83383.05319	406836.9107	209147.7357	181313.3834	0	0	0	156369.3712	116267.9045	263325.1943	152888.7057	37956.832	163008.3364	31042.11742	318063.0991	123907.9434	0
65189.79357	147201.395	430695.6084	205263.9755	186755.5735	46181.93445	249428.8531	1214939.727	265591.6034	639355.9778	187980.3004	668200.9857	307532.5234	401496.7761	585947.0954	371987.223	940886.9874	233686.8812	76057.31249	178331.0268	137010.4306	203474.2895
376241.6	217971.5053	397996.1211	3165388.632	327703.4809	224521.3342	919261.8434	3066444.535	701861.477	516216.6917	2246378.139	427006.1997	762570.2037	0	2107139.434	2235636.139	1345091.568	4371888.869	3999274.101	2021605.471	1621723.868	1135719.373
117325.6427	247907.2683	417339.6483	665484.154	1317422.541	724304.9186	1023586.208	613348.9562	402256.9772	115421.0519	430454.9655	535049.4672	678596.1403	1027821.91	539185.4503	1487708.304	1349138.233	950955.9285	1184671.995	919263.9712	873602.6627	637798.3311
471986.6174	897020.1361	393472.4344	661728.0693	0	0	127954.9792	527862.1851	683647.7939	346475.6219	0	0	0	0	600830.1501	195613.407	167027.0874	1135084.501	132456.7642	1159534.187	0	314134.3717
93924.19453	0	213147.4213	325871.1101	345140.4184	417907.1476	238306.632	359182.5368	252244.5916	177484.2497	313395.8316	145643.4071	0	330153.1916	0	508539.2188	0	557729.1179	0	0	262979.1082	175488.7154
137157.0879	0	789583.0395	953470.5948	231248.0297	259491.7187	174747.3557	559919.1512	796509.7373	1033848.57	620738.283	291955.5399	130640.2428	460050.2491	1325071.295	895861.0481	1167720.774	464564.8046	1044909.4	1202301.157	50994.44709	374674.1366
0	0	64269.32383	130382.732	78032.45113	197484.0029	197023.5979	258955.3969	83230.8374	31161.87935	27067.2211	0	64950.9985	0	227150.7184	180258.046	74021.51626	21760.22744	344783.2764	85190.30206	0	87248.2694
0	0	0	210406.0943	0	0	0	0	662554.849	273949.018	876269.697	0	0	213382.1329	0	873743.8493	906372.6768	1122602.951	722319.178	1346150.665	510886.8483	0
0	0	0	895021.4647	430503.5047	68124.51329	405551.4222	284420.2115	277891.3711	14222.55419	134370.181	0	0	31902.12232	217724.2522	316794.9765	324739.6226	121526.8528	325412.7299	80517.20101	123451.1616	24010.19241
430221.4178	376241.9982	985178.3462	205848.6992	1306765.543	807869.1619	462687.2402	952538.5781	1025364.822	719175.4327	796846.9272	663212.7121	825252.8724	841530.4442	1154271.511	1900985.338	1971660.744	594854.7644	1006787.358	1045888.119	134679.5431	463608.4703
51610.53298	99521.57521	0	0	0	244130.3719	58638.74627	661803.0258	425066.8167	92377.08031	133704.0332	0	293974.0056	410560.6681	509191.5245	31959.7225	34992.90825	143584.5081	293366.8741	35208.01707	28059.85631	72619.9368
1161232.454	902997.7498	646258.3606	555304.8249	938636.9173	323115.4507	516797.2115	483635.6065	143818.8452	777484.4229	0	19885.32326	0	82659.53542	135587.4556	935060.6722	260870.231	353940.9671	122919.5422	205336.5239	95974.16218	166537.9183
0	52639.62252	0	96010.26801	34118.11903	108298.399	27682.21811	101607.097	374634.9121	170539.3006	131682.5479	102810.6777	0	179139.2085	133237.5516	107807.0967	108978.8619	66253.5611	24819.58154	1752004.917	0	0
24067091.47	15274191.39	36989879.11	31917258.06	16235347.48	37208327.12	22301250.21	45097882.74	56746564.85	33950786.76	54073221.77	21765228.76	26557629.72	28530913.7	64937860.07	46976020.01	43515315.4	40234033.75	31098027.06	49463304.56	28025202.29	33895232.25
0	0	0	27588.82375	29904.43951	78961.41668	180494.3425	32652.34706	37520.75648	0	0	12676.28264	6505.784368	28173.81154	22768.63455	145953.3987	70081.17878	162770.6453	25197.01039	77839.4305	28346.25887	0
0	0	1025181.422	1605589.568	885307.4164	466591.6065	923825.7648	1099885.426	970327.7509	2492639.955	1345262.944	549078.5143	258199.4468	203588.4138	1299958.256	898660.4159	962733.5199	1396795.102	772255.6993	681502.963	0	0

0	0	0	1061066.147	453147.376	0	512165.3281	640772.8757	297277.4808	592037.9445	0	832177.6767	0	0	416923.1303	540206.2113	781863.3442	0	835397.7249	0	0	0
1195669.328	185904.0355	622851.2649	542487.8863	919433.0318	1399690.597	318343.7069	1416462.028	2149474.281	1551121.592	2356454.989	1184464.237	1959540.834	1234771.951	1353873.002	2504230.323	2637554.766	2593646.548	581046.0081	1023530.724	990714.2574	575043.0625
204091.9124	30838.43462	585139.3973	0	0	809623.7782	329464.8316	1093731.544	926627.791	687471.4314	520415.7612	446332.7348	287804.1138	496876.2014	419852.6005	1074313.92	490094.4858	0	388451.496	322213.0524	538466.4897	472151.0168
0	95961.13913	0	59545.93781	166595.7097	277588.8209	0	0	0	0	0	0	0	0	119037.7096	168272.8105	76173.00522	164553.1598	62424.65606	34335.15598	0	48743.37512
7598406.706	7192029.44	7145441.102	10075167.7	11450722.72	10168660.17	17894696.56	5318374.682	5824423.539	3149893.93	8978626.81	9173923.925	7707433.636	8685184.022	6816382.943	14457555.18	11496159.43	11014971.8	4942895.641	10298362.15	9711457.767	5108649.328
1635408.182	1228071.812	693683.4471	2053734.585	2254850.274	548194.5821	805653.6524	1016579.898	2174871.086	2035200.195	1317319.837	1101334.66	1492073.752	1244894.739	1207813.519	1639648.807	1897341.53	1185468.868	961926.5533	2381433.441	2419149.943	1007035.735
0	0	82869.23028	203516.3901	122268.6097	54546.88726	226785.694	128797.3478	0	0	0	266856.1772	180243.4688	169288.6352	219217.5308	367556.1424	87606.27718	101883.5039	89112.55392	95785.15559	0	178941.8982
0	199822.6967	0	0	77417.26232	0	0	0	1273136.539	0	86197.8254	19752.5569	0	0	0	0	0	0	0	0	37556.40958	1663825.333
1462382.244	2144412.731	1936257.688	3534399.35	2753136.87	2759529.109	3695939.154	6509941.891	4546163.129	4465296.352	3333297.299	2152275.682	3226438.632	4223477.258	4786748.064	4073065.126	6185280.964	3738462.571	7311056.832	6336762.68	2174212.234	3688989.302
0	0	0	0	668505.0175	0	681414.7433	0	0	0	0	0	0	0	0	0	0	0	0	57108.04742	0	0
182319.2121	119607.1788	285387.163	298169.0652	177042.442	644981.1218	952536.7313	383950.0363	546246.3396	1061802.526	224351.0309	795688.7648	545659.5637	655117.0385	1349247.799	800550.3664	996303.9461	653788.5374	1111821.857	534612.7054	241017.4795	141132.132
14606939.23	19097037.34	15096513.62	3932600.366	16684396.71	20159183.26	24890986.67	19238060.66	23350401.52	22842619.65	24281486.62	18324130.75	20085231.18	11392824.55	17997387.6	21003874.14	15312274.11	16528258.8	25693632.09	17683406.27	17245775.8	23987125.74
0	0	195289.4758	244034.2245	466714.0004	413026.7328	263928.8666	31240.1997	197838.2885	352912.9428	418972.1689	394339.4714	143969.9884	356588.3541	157238.4616	333520.1939	346408.0516	435960.7413	369404.4329	470705.3117	41019.89509	189992.3483
3559583.026	7295259.63	8000403.074	8885145.852	0	5849122.864	903281.4555	10365568.64	15032800.67	9578858.777	15667358.55	4442625.224	17273465.6	3185258.505	8309389.846	18095470.45	15338244.48	8755291.463	10085816.65	12626546.04	12948629.83	10219049.49
0	0	159453.1933	185824.2991	582634.2558	1112264.336	70432.41312	182778.5289	98327.30019	61409.20255	536316.0227	0	566452.0647	381636.3894	257732.4003	293876.3813	803715.4781	0	348122.3536	1197435.395	271422.4552	129047.9336
0	217246.7417	0	151432.9753	403069.6806	0	0	0	108148.9653	0	251298.7967	0	0	0	0	459996.3975	0	0	899654.4212	0	0	515015.9339
0	115648.0222	301549.4941	674082.3364	186514.6333	289230.4017	90010.81081	808491.333	426535.5905	244159.9291	147419.5903	0	306150.2745	0	365406.7324	595611.9744	713701.8813	501770.8336	498747.7556	1194833.243	524459.6908	29392.02926
1299493.097	1642509.646	1031253.069	2852195.758	1998267.115	1946342.007	653630.6952	1096098.319	0	1019958.978	1364854.252	1784708.176	729596.7109	2169536.147	763385.2805	738758.8498	1613930.918	2596085.638	944656.9455	1051514.326	885076.0306	1371454.481
0	0	29877.68888	83186.42792	0	91800.73052	395777.8726	73728.96391	0	0	55564.37951	0	0	88383.79455	154387.474	104072.3826	150459.1014	0	45392.97775	0	82298.77927	0
817391.1884	0	0	0	0	264102.4702	264550.4631	0	470778.8332	193075.865	0	258010.432	191970.2107	864902.6383	239616.1495	195367.3443	110987.4957	0	0	134372.2147	606270.5073	0
6175823.335	15392843.96	1306105.117	1379699.615	2023307.376	0	2012084.402	2633036.663	4084355.22	1310872.941	1308303.83	0	862623.4409	0	1649908.695	8825256.757	16362714.04	1779236.96	3967216.66	4996385.474	3237990.75	0
557017.3011	296712.7321	0	163736.9354	0	100276.9777	199951.0553	0	0	279268.8393	0	75477.74871	286099.2022	306498.2615	476930.4645	421222.477	454179.773	241926.0226	536954.3949	147250.3323	306992.0002	0
18550643.22	4819650.021	1982946.148	5565157.915	22929485.59	11594572.84	23257089.57	11552776.5	6341705.001	6055221.445	5382828.347	11413758.5	8749661.844	5421282.927	3276247.803	8310463.712	2986944.96	10802074.18	5639129.9	10148420.11	18458563.54	10849703.33
0	0	0	40187.68865	191333.3738	191972.8542	54872.30827	134503.0634	0	0	28005.13914	58830.25091	152496.2103	178300.8583	0	62008.58636	0	21815.05072	0	0	0	70834.70239
6748541.888	12124675.02	11973671.78	8110717.687	10113845.14	10499809.89	10835565.95	19031771.89	9845862.45	30302187.65	16175058.68	6400873.76	8815338.576	5631968.564	13857875.69	22664103.7	10061700.76	11546962.02	13696608.19	16224100.36	3797759.436	11540698.72
95926.38456	0	93943.70993	186379.8145	267695.9757	23965.96495	1178685.633	70350.54934	0	21323.91311	0	197740.9493	92326.08846	0	230049.8742	150382.8467	200394.6487	109251.8301	204030.2899	102310.6177	42161.60456	0
8682367.237	13595576.47	9456516.741	11856246.64	23839227.48	18975523.19	21991020.26	15017996.97	14958676.94	10891644.11	14030097.22	14087501.13	14719000.82	11118145.2	15356687.66	18991430.46	15724699.75	19278075.24	16617656.99	18699859.09	17733663.6	9925209.527
29547.88547	45340.71878	0	212162.4944	196859.5927	202233.3609	781625.4172	112735.1524	0	192183.2914	81866.47421	0	0	103487.4714	161955.0169	398706.8154	290204.4462	796115.8388	53312.86381	221888.1866	76970.26626	103294.6479
0	0	0	1131187.657	0	0	0	1192154.108	1023058.416	1945915.38	782984.156	2737936.221	0	0	788616.2356	1260600.981	860247.7168	1002730.878	2680335.985	3384445.685	0	0
0	0	0	133226.3186	0	0	0	112941.5198	0	0	0	22953.52713	0	100235.06	57658.45396	57358.52522	137127.1914	260871.2902	45941.21125	0	269587.0273	0
0	3411493.11	1784165.652	2575754.884	3348205.428	675698.2589	1571148.13	2254019.724	4394157.93	3543518.04	3944980.348	4102798.766	3634728.308	1176641.406	4168169.428	4128292.245	2760151.503	3237876.595	4120375.565	1485834.878	1296103.91	3100851.439
15426622.94	9774033.641	26949194.95	30378851.34	14522540.15	20711083.15	20037628.8	10393938.83	17669159.66	23416020.51	22235269.76	5894947.996	24340738.19	13023356.84	20550174.7	13955032.38	21031592.92	18295133.21	16674611.38	33782739.24	13124653.16	11317189.58
13374904.42	12167266.51	10780696.96	14144389.1	11929638.42	12330404.3	13038177.35	13588568.1	11658780.66	11355399.59	9836251.543	6984373.251	12287452.63	15501927.32	14249823.99	18291231.01	21176578.57	13754187.57	13015337.81	18056019.9	10742784.98	11410940.32
2871707.461	2343000.8	12753553.41	6032509.006	3593956.395	14937215.93	7161217.244	15140648.36	13761203.62	14948420	5312836.634	10876220.16	16862046.64	14710063.82	12211656.59	11599797.35	19739998.09	10065029.22	15584496.05	13306358.22	8883753.358	14247305.79
405299.2472	0	0	294582.6514	0	565976.8809	0	0	406243.124	643761.0112	379670.3563	0	690683.3398	0	626778.7674	1235691.942	1335487.009	0	0	0	0	312777.8291
0	0	401761.8723	612324.9107	268348.506	1094511.387	677411.2158	1607416.219	1671753.905	1326913.7	938964.9534	0	184369.3684	0	678330.6044	741615.2454	1471155.066	0	1166349.636	1512419.948	0	530921.1465
1488526.663	1566901.891	6307038.283	5167366.891	2709074.475	2912145.437	2443379.032	5824572.078	5953947.249	1903485.808	3686288.61	3106778.268	2404018.276	2917732.124	4513706.209	5034295.861	5069071.317	1722583.61	2322269.056	2241103.862	3310271.827	5391145.345
3331888.693	1855536.516	1239428.273	1977829.146	5180970.798	4083796.599	3926200.309	3030384.473	6706494.418	1294521.819	4119302.839	8824890.423	3447862.967	4563497.443	5699038.16	3593848.353	3816706.479	3996410.794	3292612.448	5132771.897	6693868.747	3498073.543
73823574.17	44530555.61	55392909.2	34707476.43	43581436.4	48117872.19	60562013.47	61168866.09	72972199.6	55174416.16	54540262.52	49475460.46	56350542.04	77668779.63	64005000.01	100725591.4	73588431.88	70947305.38	79539249.06	67730368.47	43077337.98	47221586.99
551374.3969	0	0	0	43978.36025	0	997058.8202	1009971.108	986678.377	0	740865.321	0	0	34354.40699	2034196.88	0	0	0	0	0	0	0
0	273343.2621	1263890.433	1105957.423	2859267.311	654766.1084	913893.8853	1975384.562	1733569.584	1743839.091	1394210.374	398305.5469	1242622.378	547191.7512	2279827.437	885043.7204	1809521.312	1280667.448	1017405.042	1938120.816	0	326788.6188
0	0	59927.95465	2929316.167	28571.87038	154868.6282	575530.4096	106757.9316	72352.3087	0	69234.48309	0	0	103680.5984	93290.30774	121479.5185	128323.2081	406578.8263	0	41220.56544	21084.08635	162160.3137
236303.2088	146398.7388	247906.5283	465836.2453	855872.3287	328666.3024	1253849.988	18460659.56	0	21936248.43	0	985705.4187	6579488.358	523742.3435	298390.7192	409275.7352	726474.3559	953665.7266	18565821.14	3811377.35	1113768.471	93994.10989
269006.5439	206375.9818	457256.1151	374262.632	0	486925.5892	89544.71373	856436.131	656980.5915	1027974.098	240624.8875	703070.4699	580745.8898	860021.0606	866432.5724	263443.7117	372173.8284	389829.125	747216.4761	448425.6298	771602.473	225399.4895
0	0	550234.9159	621509.8142	795373.1115	0	645040.8787	0	1074202.599	0	0	0	0	0	833729.5438	0	269828.8775	543805.9909	0	288019.7267	0	0
0	0	940260.8682	659302.346	633424.6689	796530.7699	386514.7275	1025693.401	1626369.425	339503.6309	0	1418927.936	806968.2543	442984.4126	1646434.711	1385744.774	1459097.965	2156253.279	963012.3459	472283.0116	816133.7233	0
391329.7508	310486.8881	231325.8272	226712.6846	257467.3239	253864.6002	714465.5937	714431.9463	201642.3415	258870.2547	314691.5616	0	407720.9452	937852.4988	374428.6114	243292.619	212223.611	305151.1989	238925.894	309001.6927	261856.1354	0
1070654.105	0	765042.2668	1882783.538	1399471.38	2502971.74	358201.2702	1103606.611	115332.4305	857194.7301	1133614.534	836211.3181	2381316.918	1579255.032	1578618.448	3706998.643	2430992.107	2847860.16	1428153.542	1916016.557	591325.9294	2152037.931
352130.4201	0	0	322036.4104	579032.2722	226418.9989	194741.068	14869.66885	0	50964.38397	0	0	0	0	0	0	0	0	0	0	0	112597.3534
2423611.926	774451.3855	3164751.022	2581809.04	656343.3752	2943183.186	2187629.282	3529010.543	2907114.107	3271685.525	2446440.576	2225861.387	3192678.143	2352322.66	4163513.762	2724982.806	3208373.863	4449773.49	4229531.689	3204532.746	2223145.619	2141723.33
20080151.64	16257562.6	14154961.56	29122821.08	32438147.39	22330488.75	17667181.89	20440374.54	30489417.11	20678505.99	34771170.24	19387581.08	19390317.96	22174268.31	24087248.28	25215605.32	31166012.62	33959499.89	24124120.61	17045233.58	29580160.09	19832986.53
346215.427	300397.5408	366682.0193	444547.2546	1154041.849	1230128.798	820839.4368	744973.7235	847372.6392	972372.7545	173676.6782	1456862.863	402982.4406	1495208.719	925164.3404	575640.8058	1189396.302	1876910.233	1800172.758	752661.4361	2089824.786	1994622.972
2948921.356	3899697.13	8876208.891	6750415.398	7212895.357	5872414.061	6721551.115	7191565.952	7277203.488	5735662.967	6500291.093	2309010.959	3577341.59	3680364.368	8476655.167	15742060.15	11055036.72	15980291.51	6249216.166	6846346.689	5197334.473	3532732.95
5360819.804	12696915	16689178.31	23756650.83	18328621.47	13394178.43	17027058.15	20208617.54	18844896.05	7821782.995	13850343.58	16383563.07	13578929.12	12820116.01	15976872.61	16896631.01	18714013.01	31076387.66	16528092.87	13419235.9	17054989.31	16154168.43
105155.2751	240774.6509	103323.912	3485492.031	84115.26259	2365661.546	278996.109	79216.24353	73953.30146	38678.12429	349431.446	1154556.159	1886451.658	131389.5962	156533.7238	453730.6322	283767.6435	170350.5463	439611.9388	490459.8433	51474.64902	171595.3941
0	0	0	1456.823836	33643.63612	0	51892.52669	0	2131.19231	0	35091.86311	0	0	9302.093187	0	8020.434893	33855.98465	131828.3389	11665.0981	10998.95215	0	7207.831023
0	0	21716.89726	0	995960.7689	545742.8976	3661398.736	35453.29188	451548.9422	197545.8235	304136.3105	103813.2156	224655.471	219970.2409	544588.3954	596266.7121	532503.8762	681235.3506	0	0	153131.7429	66151.1837
58589832.17	42610625.24	29173144.78	37889507.75	8971132.243	43458251.95	16161009.91	52407141.61	37952418.55	28481774.29	51562651.54	51274081.44	52324725.47	69775103.39	65683168.37	60753230.52	43145253.94	37268930.82	55590245.53	51240178.7	78667942.03	62685503.2
2373850.087	1700828.948	8889598.87	7530026.019	5753703.574	3954649.555	4258351.116	3201703.313	2679056.175	1509656.188	2606161.87	2352261.333	3652774.205	2641573.375	5558191.645	1481901.123	3693465.248	4649525.659	8368272.83	6695184.897	5093071.102	1310122.335
604818.2952	294359.6968	0	0	704736.9129	739408.7768	778625.1291	0	453178.8761	1044811.656	587452.7828	0	439756.6936	782715.6477	516733.4841	299022.4583	1168875.697	707360.4863	633137.8556	1268467.353	776219.4663	882537.9455
0	0	19621.50839	131935.6764	57592.56002	39579.72133	95920.29926	35580.95095	39102.22928	61872.20925	29784.46837	67214.04702	73527.70992	150596.2893	38083.34634	58868.51752	106156.0425	35393.60687	35719.07903	45890.17099	18156.8614	0
11785102.69	19340206.51	14010283.11	23709717.09	23613377.5	27416232.57	22685502.12	22350532.16	23444630.09	25589643.12	19498663.81	18589528.47	16248672.25	12437136.72	14559497.53	14192634.61	16689327.04	23814207.55	12608175.09	22445066.88	14663974.56	17254153.45
5069223.223	5251506.471	7673265.113	15366018.23	2429880.699	12092484.36	7657589.17	8386395.314	11186974.4	12740333.27	6641287.548	8256398.339	4612655.126	6562974.358	6851966.777	19968143.64	18212121.82	11181269.79	17606707.84	12319973.51	2939174.357	3782635.736
7262752.571	5937264.231	20465354.59	20565158.52	7648091.423	13501415.23	8422723.713	13171368.06	17217652.24	12019998.18	12781075.8	14597836.73	15923571.65	12840891.49	20436838.41	18840557.02	16496006.93	26027536.28	18084089	12232932.59	26308182.1	12330733.43
375049.5837	732442.6668	1878504.166	2692681.05	523712.9378	1436660.862	1036665.681	3073690.69	2354093.97	1584115.753	2699319.368	1945378.783	1062679.004	919732.6338	1494404.159	1030367.285	593512.2195	580268.482	866478.2253	0	2709656.68	3608156.189
2626492.306	2835347.785	2271891.039	3228327.054	4623192.487	2939834.48	3411443.674	2851518.613	2383600.388	3823804.301	3234699.859	3651001.002	3757463.995	3393525.106	4750382.206	7098344.39	5483764.562	4664748.092	5825376.514	3369066.37	4361117.713	4069559.797
34526.41958	0	459641.4669	421637.8381	521998.3014	602752.7773	612261.4311	881615.0271	265927.4367	861196.4324	396053.7767	679568.0879	273837.5192	693989.7889	139343.779	216695.0473	225238.5016	778737.8841	948579.6641	387589.2027	304856.394	415455.9282
85520.29936	0	304225.9186	600886.926	0	102365.3369	0	162280.6855	147063.0381	326899.5017	74860.15436	252819.7378	276593.3801	0	277888.6305	218566.3736	491687.8891	389000.5888	267494.9686	232398.1831	255449.0618	0
0	0	1413830.708	1670758.98	845365.6822	1598237.769	1659884.346	2025275.341	887165.9607	1597053.876	1330392.081	1277725.616	1149886.08	1744099.738	1564539.336	1631258.663	1582379.875	1645589.001	2274176.375	2126747.828	420203.1755	563532.2431
31243918.11	23882602.7	43468308.55	56952423.77	47593787.37	60105855.72	53484421.36	77607603.22	64472134.85	64767099.9	50714391.94	43309788.5	51911062.88	49402518.4	19181226.51	47081070.6	60256276.14	39972129.67	58633461.6	55228580	45808036.78	34062129.12
0	0	0	83335.78856	71101.87293	95620.94726	0	0	27341.06367	0	0	15804.5621	0	0	938567.1975	91182.39564	973057.2551	66110.7196	119463.4405	855487.2596	0	36270.43017
4428103.741	5380804.195	9933287.093	9438977.554	9215303.117	9003117.164	8665157.703	7609598.302	12228271.17	5221021.768	8137733.471	9766854.864	6293770.697	10042622.3	9578040.524	16745173.51	12368220.59	9999140.909	13797612.14	9477735.251	3923150.161	6391603.62
94396101.98	139235146.8	166979708.9	173213597.7	78287787.86	129586832.4	177474412.7	179222909.7	206615439.1	190073743.2	167693355.4	158314980	175235032.9	223226560.3	191606692.3	215236712.8	146668894.8	150728725.3	142924658.2	121138338.1	197067505.6	125644743.1
0	0	95379.50864	0	0	315682.5524	0	190880.8984	321510.8778	268118.042	143596.2001	192154.1243	0	146511.5884	356828.3078	278623.8581	66250.10855	288340.7383	0	430313.8113	58543.57305	219632.6729
83814804.13	50583880.12	95507404	96760434.2	106575287.9	97687853.18	118254134.5	103955250.6	112731810.7	93833880.44	90224267.41	70180340.24	73254606.58	95504022.87	121681966.1	72381997.53	78077171.53	90919339.66	109344130.6	82400154.62	106424976.6	94366939.35
385701.4898	356482.7611	0	606687.0011	2106184.177	0	138719.9969	79878.45931	0	152662.6395	264227.3005	1194551.268	76691.98908	0	680659.8067	713764.4445	907242.1818	705943.3087	0	280995.3013	439090.7933	504611.3799
2060340.725	3377185.174	916254.1787	1786037.906	2812486.977	3666429.926	3456398.424	2362589.747	1993062.006	762710.1142	570802.2939	594248.714	1947276.824	2404362.519	1229330.899	1937729.279	1448449.883	4717780.354	676188.6826	2179296.029	2570676.473	1308425.809
0	0	0	0	496812.4738	283822.513	783058.6507	0	610956.8315	1176112.359	208473.7488	0	441475.8009	328478.5575	220459.3893	0	479588.7699	0	973621.0262	0	0	0
66168.0423	41908.8967	332725.4273	612995.8477	456090.9339	731385.0973	728794.1444	956900.6282	621544.4278	459338.0113	376280.2166	879477.544	367678.1828	1150797.614	1255340.436	944425.2267	712083.4577	814478.937	1153042.219	609895.9174	157018.9104	213590.2519
266290.7328	469363.9676	488454.6098	839690.1566	236272.3657	343861.7058	411170.48756	679151.0779	290657.929	786422.4461	483451.0118	764855.8315	481522.8651	589836.1149	622485.1189	722416.3037	664893.9717	425672.2703	430504.9023	756636.3046	239944.012	778169.8671
0	0	0	0	0	0	0	0	0	990359.6448	1665857.08	0	0	23950.73531	0	0	0	29363.71322	641372.0824	0	41158.62668	0
[illegible]																					

0	94228.28158	520310.6495	336246.9047	890820.2755	793657.8988	1424154.03	342916.2523	513595.7783	826031.3007	350773.7295	335978.255	324660.6288	168499.6512	766700.1034	914677.2673	883063.6504	1334453.865	503816.4064	853745.8977	258012.3702	124754.4664
8240651.558	10749243.19	42667893.24	25470222.79	16262003.5	30640116.67	34218085.85	50147670.38	35815156.91	34843164.26	36960685.1	24312749.15	19966336.18	16627233.43	20285371.77	39699082.25	32229013.21	24246197.34	34105326.4	26809653.79	18709469.61	23266295.91
466517.3986	60784.3827	107900.6704	202080.5259	235982.7108	95961.63841	556782.1319	181336.6418	463508.4166	225991.8978	269851.2426	208126.117	237741.5925	1409504.285	214430.0532	537409.425	638252.2898	380728.1352	698948.8669	258550.425	0	186075.3345
20843466.62	16321944.04	18657268.28	22161196.62	31366298.11	26884045.95	26141883.81	30476459.29	33055833.8	42702624.5	27802095.99	26913076.01	17177064.31	21500914.37	28164524.23	30453405.58	18905136.81	30140608.05	32107561.55	26136151.52	27702394.15	48311390.67
6783281.262	4699039.096	2452527.266	2499177.638	23923315.66	7012965.389	6567752.133	3787890.585	1741448.282	3185859.707	14972312.95	6568355.508	1201352.165	1595384.088	2164546.81	9004811.101	4547412.293	24067651.98	2747508.105	7831900.237	17967437.72	2534513.931
1218695.279	0	99417.88243	120481.9668	322666.2796	338775.5956	741465.2518	413960.2839	1008445.623	720856.5845	210717.5487	0	0	0	454987.4855	462473.2702	0	97285.84716	329816.9688	389357.3192	0	621814.6778
2782348.084	1631235.398	1931479.075	4095086.472	3666802.104	1578770.333	3649544.29	2686758.36	3538166.696	4126745.134	3672816.363	1957730.975	5018407.018	5054382.383	3365849.755	7964342.539	6260792.51	6152984.265	5064222.503	4111224.679	4721251.541	818857.8535
35876592.5	12342256.93	60360818.97	56165515.97	48843817.94	40978190.56	38343813.17	50025827.95	64285224.4	91418509.57	72863325.76	22453050.81	29434555.17	54180384.18	48044702.3	87371105.25	55722010.06	62123741.35	119010886	79151562.54	40633475.53	98037788
144641.2072	0	323086.6295	0	114777.2706	723470.7628	0	0	533152.6687	121476.6642	659732.1924	825096.0531	872205.0864	702490.8005	181813.8081	968395.9112	1250967.192	1096732.676	728407.6042	503345.852	576736.0707	239775.2394
3196039.747	1592297.068	7081953.032	6365788.426	4508693.867	6789614.303	8190527.17	9995384.426	10112079.65	8765526.145	5884631.24	5877851.831	4140882.216	10532890.83	7794301.057	9398913.676	9782037.175	7334957.646	9398681.535	7637423.596	2149774.577	3252244.655
10105942.65	15515519.21	23767965.69	34225798.12	16766109.68	19323991.44	24796466.49	27430717.95	24909365.69	21644075.95	17782401.11	23482928.57	21504590.75	20148003.11	21813705.46	33956202.56	30266017.08	28293337.52	24400311.95	27457871.62	12496871.53	26142899.12
0	0	106327.976	110819.7409	271871.5771	203379.588	366878.8198	108790.4715	64974.36581	58534.7275	104901.1393	49553.37383	60995.1718	172253.0778	0	65794.44249	233279.1956	58460.22044	197026.8486	264734.8606	165914.2208	0
2766329.903	2215908.577	9093258.881	9132667.915	13749194.49	9054095.88	9428489.559	10810509.37	9673385.157	8778479.495	8184241.987	10329926.64	11655760.6	10833101.3	10280573.58	9747215.319	10380961.76	15593188.87	9475915.595	14306707.47	9769272.542	6597942.544
0	283838.1104	186960.2653	257829.0483	789537.8745	725881.9239	426180.8923	914379.2684	528218.5596	121057.2931	122033.8315	1185972.866	398830.6878	334380.9519	440135.6119	759899.7717	498204.619	322766.3678	461971.0591	593706.1489	384073.6646	1859968.683
0	0	0	0	0	130334.3058	30264.80012	30077.71394	0	133331.5239	19344.21546	331984.6687	157944.6938	0	95233.55129	0	114531.9809	75639.72663	0	0	0	0
232764.3489	37099.96832	501914.3786	244721.8794	63338.22317	222322.5481	648453.6132	295135.041	386669.4843	69817.06386	68760.59476	29385.46174	611949.6368	149852.7728	437601.7864	1005425.492	548741.2279	222016.2572	311922.3649	250303.3293	0	0
1779130.349	1233289.451	1143332.849	1505142.815	1634594.316	496740.6756	2677904.584	2103117.985	1811809.715	851313.5266	68257.20584	205908.406	2725540.708	1160278.639	1705869.072	3152444.747	2826622.527	3066970.077	4090102.659	7402201.953	185743.4317	2735301.479
1047943.512	2593102.561	945993.1572	1469981.055	1691315.393	1457830.274	3003488.257	4105080.6	4602843.603	3819384.624	4706385.741	1428807.138	1112184.98	1861424.798	2147481.141	209005.4445	2079586.989	3931955.684	3160825.834	2597306.147	1065026.239	4975882.908
1154910.537	318787.0526	0	183704.357	1191582.968	233499.8714	588650.541	245101.5578	581669.7144	251359.4245	280210.5592	1677505.655	734744.4752	953707.3308	619706.544	558553.1071	262198.7557	1310379.53	0	506670.9736	676849.0589	103709.6078
0	0	562672.4115	791770.7285	0	813414.6012	109545.6693	729664.0571	718853.5164	543533.7149	0	81146.46991	381629.4181	252781.53	0	243587.9898	416576.9341	582550.1033	748830.3227	1701361.553	0	187291.7198
0	43079.79561	238475.9357	0	402223.6401	0	0	122831.7959	484887.6567	0	201198.5998	0	169872.3915	187519.7437	264364.9208	1264409.501	502606.5096	172232.2525	203208.4789	0	259569.8136	118096.5458
0	0	0	0	444677.3533	0	1909033.124	0	148529.6991	0	228957.3421	154286.4793	0	110180.3716	0	104632.9939	0	121342.882	198712.2043	0	102214.844	122735.6106
2279290.564	2010829.99	0	0	607604.2287	0	2857249.356	0	0	1256391.005	0	0	2209970.214	894001.4746	0	1615999.412	1673547.488	1713734.249	2800557.052	1227317.295	944269.4534	0
2770101.509	2788185.795	4398737.927	5147692.607	6310925.492	4246416.352	4920695.287	3276369.18	4323632.978	4283160.258	4215056.626	4804139.901	3911918.273	6543609.603	5015695.59	6937749.66	5022034.552	5651955.499	5461123.837	5091326.789	5544489.548	5037351.158
163536.6205	32205.57489	60114.81269	49577.24132	152904.8566	45056.19646	270952.5602	203844.0673	148981.23	202437.3316	115975.255	78008.63831	106770.1313	255619.6111	62461.89428	110125.2824	148275.28	263063.2319	247768.6366	64709.61339	169211.6023	197747.944
0	0	57881.29259	20355.3183	78491.40393	6078.402435	80593.23528	19107.50039	44141.4964	16254.34436	60967.79761	49251.95658	7915.443125	17416.69838	53970.13586	25564.46091	23402.1142	66877.18428	41978.66732	38967.79996	26940.35777	18187.4506
0	98617.72455	0	55674.37696	53476.50715	78423.07878	524955.739	406664.4824	516530.9503	236935.9177	390555.7617	217682.7707	0	0	0	0	0	217315.0344	0	52157.93362	0	159982.4872
0	0	546164.4492	0	0	0	0	263141.6231	0	0	0	0	0	213689.8446	615428.2631	0	0	939764.3914	483324.4362	0	0	0
33557.33995	50886.64606	59266.28844	187212.4078	346701.0252	138973.9013	278052.7513	0	84755.8035	162373.6983	274593.2554	0	0	74201.05013	95590.64604	202178.8611	222309.8027	180024.682	91332.16062	171134.6234	0	0
404650.0554	133522.4137	627714.784	688050.4046	1072263.658	1280835.724	661260.3767	369346.818	1159861.626	342910.3779	1236801.805	1220685.454	1122810.321	163330.8605	1258170.694	1477619.258	843282.1071	946326.4927	895637.1234	58344.52856	829648.5265	109996.2928
1818801527	1259404157	924213156.3	91146059.19	118034808.8	1260293708	472908611.8	1267047349	759452928.1	522921280	1488931002	968238516.4	1469702308	260271033.6	427090892.5	1216872205	813712058.9	1027068798	721961258.8	878129253.2	1322932322	1384843811
4368229.33	3871299.848	2215659.599	0	4258234.858	4536918.895	3699601.492	4730929.309	5037934.7	3687471.371	3681757.867	0	6541171.743	4983213.368	0	0	5010367.778	4503281.748	3350024.126	0	4596975.136	5637204.995
5086488.761	7733910.937	7718414.846	5341429.929	14255757.03	6762607.997	12675183.79	10248748.87	10649082.88	13717104.34	10383410.44	7390639.452	7753048.236	8503178.865	11716683.87	11151943.77	11103304.31	15931164.72	8347790.887	13518356.44	14567533.98	6812106.509
0	0	0	0	74069.77424	0	2100063.915	0	0	0	0	0	0	0	0	0	0	20596.68741	0	0	0	0
57303.1429	272918.6286	242841.6174	247275.1814	676125.908	550732.8647	1389324.462	710397.9491	234935.9952	382600.292	199448.4661	243539.6615	364198.7652	946400.5816	215961.5558	742363.2334	507083.7451	170593.3821	322539.4805	714369.523	195976.1702	157774.0355
2703643.485	1254722.326	2058855.578	3855222.693	6595681.126	4219161.694	5252563.54	5277571.895	2244595.966	1823270.4	2846952.289	3064016.897	3591638.73	2620401.183	3632425.637	4477756.34	7017791.706	3659583.636	3304054.136	2142776.085	3397328.588	505913.1117
0	36951.51029	75043.54066	109065.353	637324.965	136237.4908	895173.5622	121368.9881	176213.0451	121266.6504	290431.8166	179165.4156	378593.3149	608395.1979	116526.996	170376.6494	57442.10077	590688.9513	289954.4878	392720.907	425642.5641	5865.269292
0	305168.6131	0	181443.6828	128154.4058	31163.21754	145576.9297	324958.3452	222832.8164	1091464.987	125966.5909	0	77074.14328	0	183763.6428	240247.6121	71622.00774	0	90455.29857	0	0	0
0	879199.4927	1334250.97	480932.7383	0	1217554.185	160548.4796	1126086.906	1854191.705	744176.27	135259.9322	2425140.258	1750708.288	2775894.675	2400446.293	1742602.175	1425704.628	0	4552461.37	839797.8462	174988.515	418069.0107
0	583956.8089	1122621.238	770867.5805	610826.401	1366267.112	1041844.001	1224605.865	2013600.388	1177415.607	735909.6746	2317534.194	853403.6244	1846227.843	1363601.127	1085214.395	1160984.919	765737.8048	615567.2863	1421569.919	563294.4502	658829.039
486388.2917	339348.2916	671386.2078	628817.3468	638735.4073	476850.4067	1558124.359	1148549.334	1459448.033	671336.5205	953881.2887	578899.4986	542302.5869	891930.0866	859695.1201	1838770.557	1453172.296	1006597.962	888847.4452	965918.0035	310503.1605	1068249.866
19628043.47	31768194.12	35735339.62	26009869.68	15038492.33	30936610.08	37753955.41	39882221.56	39307124.92	33485561.85	35189685.43	25067252.53	32763099.78	42445824.61	50659671.36	60905501.51	40927153.48	45993987.5	3902078.38	46641585.68	29295448.6	9147708.138
169440.1581	514123.4189	920804.3775	213415.3584	1388154.165	664927.5199	446735.0313	660227.1711	440659.3563	941187.5744	1024211.719	388389.1279	820019.2304	433141.6965	1956188.071	73246.97418	518750.9903	863011.561	229651.4528	574453.0336	665415.9608	473401.0433
970256.1709	983278.8179	1808901.373	3224474.218	3156139.803	4674758.058	3921504.292	3412586.237	4458831.672	2201329.505	2815313.765	3780909.755	1824796.355	1312609.917	3533825.313	4894169.153	3661069.381	4492530.872	2986005.775	5115672.555	3162031.841	1659501.439
411304.1652	0	1797656.116	1180628.079	1022403.012	0	2380358.776	411030.8093	782237.0167	139534.9012	1211172.077	0	0	1180645.263	1132828.537	993786.0815	2538056.763	991054.6232	2183909.828	1914063.328	0	1397549.239
110477.4638	569574.0445	69702.3812	521375.2123	1164565.659	354230.7306	261132.8878	557268.9777	0	0	0	0	80893.20172	0	405389.955	248001.527	443594.2347	62287.23155	0	197820.904	0	0
1411552.593	726785.7984	0	3392700.047	1519830.984	1077336.107	5170456.774	2500618.67	923526.716	2385724.273	864147.4274	2887551.956	494964.9862	2097418.067	1831979.94	1507329.741	1439468.001	553019.3603	1604855.791	1638850.725	994184.0492	501348.3839
0	0	2906222.498	1121018.659	835963.38	269612.6849	123196.7667	793557.762	0	0	0	1243359.246	1513832.691	3193646.63	1256754.16	1287363.808	673538.9759	935951.3003	0	0	0	0
5698372.634	3087973.653	1535341.52	4863151.149	26360238.01	2869569.76	28180147.41	6280495.383	2177417.402	1360090.148	3874886.261	12888882.87	3896009.866	4346056.979	1627899.396	4114223.192	6862729.289	9505803.881	1634828.805	4279411.491	2942733.722	1339623.324
2901474.867	1763614.784	1627180.027	1144898.678	2150534.458	0	2024256.7	1840424.878	1843331.089	2498474.731	0	0	0	0	1061639.202	2191937.773	1721907.433	1908699.614	1319436.447	1510697.606	0	0
6918053.312	6042050.743	4133326.122	2479655.663	5328995.938	5335444.672	4268095.027	1692716.474	3239556.654	5146050.359	4406898.467	2306175.415	2363858.627	4996088.133	2441404.634	7715404.277	6586759.823	2713248.892	4364669.074	7026151.331	1774194.739	2728228.658
2461246.156	1814850.203	1343794.636	3104664.481	4844410.028	3246880.006	4615534.363	2335868.18	1929949.427	2027909.271	3452523.103	2320550.447	1398342.114	2895924.225	2578026.868	4638182.916	3417986.161	7397653.48	3741985.324	3171943.819	2977655.814	2428552.387
1184179.631	1362178.542	1570190.518	2362976.751	4833202.523	1235684.957	3668860.329	2124773.526	2031783.205	2204083.427	2364586.807	2542748.048	2191196.637	3781633.675	1436630.881	1627765.977	4082564.852	2954291.753	2383855.029	2361519.847	3331931.653	1442899.916
1033553.301	338769.9083	3026678.344	4256432.836	2759076.324	5004782.551	6365838.645	4713808.466	2861818.524	2167495.716	2423441.798	6766791.975	4625250.375	4633200.52	1797567.63	6442273.666	6289729.457	4245045.745	5442130.72	4820210.583	2522953.803	2704254.075
1292732.583	2607255.997	1153416.132	7265138.748	1894316.873	77576.01871	2471194.466	769419.3188	3016616.896	278896.4516	234533.2356	354382.2816	3563523.498	3241959.761	836508.7762	0	2200992.489	6790236.114	1120248.79	6262150.911	630166.8423	4698043.748
2546589.639	4451170.428	2371111.164	3763666.596	5559244.893	6069937.736	8200181.277	4796948.34	6963831.919	12128467.32	8149142.891	3963920.903	5342520.633	7999262.479	7730411.908	6846165.863	5642565.469	8521853.695	4728413.601	8848072.788	5713526.884	4542056.976
224581.6591	261477.5617	329120.034	644941.2709	571680.9496	350539.3046	266492.7154	636391.6017	575084.6677	331568.2212	731004.1482	146899.576	514718.8624	624187.5498	779281.1794	428368.5996	518086.5782	258966.3696	171706.3097	215420.9065	558345.5879	442358.1192
865015.9938	0	5034391.526	4585491.881	5448901.284	4634345.873	4500322.08	2892690.749	3335151.344	258485.8251	2689465.277	4583967.525	2822188.131	2608308.073	8087775.407	5274049.93	5252749.212	3879549.243	5151737.654	6915321.384	6079001.26	4352525.81
15039243.52	8168514.617	15973918.66	12763158.29	17001993.29	5213572.167	18792097.69	17212658.55	13636566.19	17920286.75	12279287.7	11903292.92	9736573.997	7171591.604	23717015.36	11209034.44	18743632.85	6748626.149	14966309.77	651349.3233	5260847.681	5257130.043
0	0	57400.86995	63344.3529	126528.6981	0	363119.7039	0	323627.1112	0	272728.9699	422792.7616	0	0	498690.5835	598148.102	577658.3243	346627.3618	120284.0599	0	0	0
275049.5665	961001.5157	1961217.596	1011256	2108498.017	601349.1821	1489535.029	1307295.844	496889.2549	2018778.566	1751611.943	835102.7864	2034817.617	322850.1741	3510317.157	1486177.374	2685507.054	1193835.53	1735299.933	1049207.069	2190606.447	1676273.699
1235693.937	1662422.17	3600406.313	2205873.594	3928827.072	3761061.65	4313260.645	4617006.467	3339190.852	3588166.97	3884427.036	1375785.06	5234730.428	3343640.851	3670411.378	5062246.377	4511449.644	3315785.056	7243831.32	1910226.082	3767683.518	4021013.374
402272.7892	509106.0725	1065988.733	973433.4041	412177.2009	1212015.603	402446.5914	1638554.177	2515733.974	1195320.279	1799134.225	2030844.29	1417180.476	512708.1713	3061804.494	2481654.359	2763284.208	480614.7465	2119945.528	2391289.651	503203.7558	453551.6931
832675.0898	148376.1829	0	0	0	335271.9557	400205.6079	73275.34362	0	0	0	326692.4997	148243.2045	194101.594	337910.7488	143472.7842	0	177340.026	267128.4213	214271.1294	236411.519	0
1464260.07	5018771.452	4380560.969	3827412.738	2050778.608	8234920.121	6860093.471	1719968.493	10700957.39	17395194.97	12920566.88	1716712.581	370106.5745	3221975.93	4619908.162	5528241.372	7854559.732	3667317.262	3078361.484	2386216.721	5820430.483	355679.3559
0	45517.84825	0	394169.7359	91954.94247	126308.5564	342890.2676	39821.64483	239583.6165	262887.0735	110204.0455	0	0	0	0	212809.1477	142219.2332	0	0	0	0	0
8753782.142	9852860.283	45314017.41	61387053.01	30352468.35	26146603.55	42878806.9	38444710.65	29547276.02	40378152.17	33346626.5	18121533.61	35361894.1	17389867.66	30511615.53	38228965.78	21321319.94	51209367.34	20616349.34	24338548.04	28411484.43	24677035.29
3302199.886	1719633.367	776132.6195	783736.4647	862887.3791	903019.4023	776935.0744	1176450.179	867643.9368	1362968.191	852272.1759	1235649.13	1631874.076	147963.2871	2294998.366	924744.8468	692372.9613	1221709.776	417403.093	1628373.613	2615696.138	0
0	841266.0208	1003099.61	353741.0538	1510170.584	340140.5142	262076.8266	374349.2437	1100045.969	1495580.039	764594.3251	587568.6843	452542.0765	379701.7648	1198724.079	1657753.88	1544409.599	560105.2813	2136076.78	1285568.396	0	1764715.623
0	0	0	0	0	0	38261399.14	0	0	0	241164297.4	15315015.77	0	0	33707025.36	22990030	20241472.18	0	16948625.69	0	16340052.69	29145338.14
[illegible]																					

12802854.78	7773013.093	20117245.1	16081622.28	14644976.46	9581513.746	19399635.51	17007699.59	24854352.59	16382755.41	14540194.42	15878804.89	20465734.77	22844392.98	20819115.64	27371688.57	12663597.27	15501573.41	18750077.17	17570239.09	10012309.02	10955081.51
171675.6901	448399.9577	0	188892.5624	0	0	1124719.212	161800.8749	0	0	0	125522.1651	0	200372.1216	0	287329.3943	0	255819.1775	0	237823.3369	112998.1452	0
448402.2292	968859.4858	857586.6283	2135748.386	5353294.481	3000876.426	4883563.784	1898598.671	2029322.816	1073739.608	1448441.043	2337696.444	1617560.774	2388143.544	1590728.335	2262495.142	1981744.841	3113067.898	918745.1484	3068787.555	2489020.063	2910051.246
0	37609.26375	0	178173.0033	146657.7657	250447.8465	0	141628.3852	0	481171.711	168444.0898	0	474978.1963	574694.573	0	611448.6648	537176.4288	550554.2967	812698.7939	591831.0909	0	0
263214.0111	216545.871	447028.4422	392330.1524	326243.9427	506943.8149	266825.3152	281801.5408	355379.8279	629393.5881	0	31886.76966	717488.9683	542116.887	411162.071	340077.0916	695394.4574	369839.5301	335714.6204	370877.811	478574.1911	1320862.761
97728.97018	211054.2489	0	96187.63017	61267.68238	98766.67066	721669.5426	118596.7654	0	319886.1817	115778.2564	399374.6745	0	131864.6336	0	520299.0825	0	825719.4493	47928.30516	211149.8706	152620.9635	0
7148978.127	2200696.402	2902188.1	5875478.425	7687743.278	5024328.804	5828346.126	2568307.396	1349214.364	3981305.279	4076821.223	3274038.142	1981009.274	3693820.739	2256384.305	226397.3643	6439995.441	6211442.302	606190.1307	5754520.488	1727929.781	6371449.232
0	234510.0592	0	171977.1578	0	186415.9709	0	0	95190.25654	221159.3001	0	198070.0963	0	83999.44661	223816.8445	408819.8622	368840.5256	0	0	157601.7561	227040.2502	220296.3384
0	0	109853.8684	71834.41015	161534.3823	280417.9098	130721.7206	265663.5377	291068.528	157416.6121	241317.4261	193083.0805	132069.5764	285010.405	231913.6427	465978.572	127915.6904	0	258923.5777	135977.0685	0	281970.361
1336020.78	1938043.918	2286101.906	12744291.35	11617161	9191554.019	9763799.686	3063036.443	3255313.796	11302651.64	4886805.397	2603059.851	8190020.811	7034718.272	3350386.013	16666803.47	2999905.26	13114393.32	4065943.308	10734516.49	4243871.848	11773859.77
0	0	0	0	22864.4768	28394.21752	301366.3528	0	0	119708.9458	0	0	0	0	80838.08613	11606.59703	11776.10742	166647.819	0	86106.35691	0	0
796828.3285	1303769.255	1529551.315	2069866.537	2029302.191	2368321.119	1833923.63	4196641.789	4287567.455	2253412.204	3695331.041	7229769.288	3123000.4	3092809.445	3758489.176	3470037.986	2793582.546	6312341.057	2154828.118	4371182.703	882016.4586	934300.5187
5421.17649	19537.01114	36386.79372	26577.88595	114967.0586	169545.2912	482075.5989	126181.8932	54400.9607	62470.66615	61282.23277	78177.76913	78125.78608	63244.85009	66867.19811	210933.2411	890394.2562	206944.9841	154126.0409	2202310.041	31575.18807	33088.7784
67556.73515	178767.9911	187581.9651	167058.6321	254002.9172	104084.8534	0	0	96244.81719	447993.348	0	164930.4041	334904.2026	81431.04235	123189.9084	295585.2527	26529.24663	504471.5386	320992.1639	348397.567	527870.9244	108922.6434
0	43732.50536	354191.6939	864426.1957	362848.2351	0	478185.9002	114512.1798	356709.8676	712483.1059	595968.0581	494289.7686	0	513937.2819	179138.591	2002478.946	471695.7835	475597.4844	547078.5788	792225.4865	0	6560.864151
1665032.633	379597.4595	5466209.589	8025083.979	6155368.505	9041574.859	7741908.626	5482976.975	5154661.269	7529862.598	6393899.964	2994567.325	6328824.903	9996949.272	4353759.502	7897775.115	9806522.024	9985751.026	6769455.254	7950447.639	1362489.119	1296065.406
0	69190.98797	0	0	102125.3924	0	64623.54063	98607.19842	113668.7134	48027.25798	89987.01403	943963.1418	0	52439.53835	59200.4145	0	66580.65083	0	0	72707.36393	0	149684.2088
0	0	0	367403.2305	0	230297.1357	109021.052	176555.6427	0	0	0	0	150240.4682	0	0	0	0	0	0	288369.0813	0	278823.949
522425.9448	267474.3685	2744591.277	3443216.584	630654.6369	983563.1098	0	2627480.165	2413600.666	0	2323164.283	951770.9989	742375.6441	952703.2959	2727362.036	2065954.865	554953.8942	1019710.324	1372195.8	1470583.87	774461.16	1219383.318
109530.5016	29131.80291	182840.6417	204108.8287	672643.8234	129195.0667	550303.6659	392784.0693	135489.0882	228316.899	175362.5204	0	0	484611.6954	723656.2479	790710.4734	534830.173	734103.3909	448320.2796	310134.8147	160014.0064	107383.2415
6047359.682	5616473.4	13148622.7	15437725.02	7367963.271	14106952.32	8293458.411	10666394.49	13297335.81	9830753.365	23835554.13	10056707.24	14867079.4	20106482.47	14386716.31	12529059.96	12841422.85	18814026.1	14875195.47	24887937.93	19592193.33	8383287.083
33064.48942	0	0	63042.90344	76639.74589	254200.8669	317263.7031	24002.33828	100537.3278	39807.93382	50738.47207	0	40910.04212	35989.87208	46659.47924	382211.4012	103592.6007	415359.5108	211440.2211	39978.85206	0	17314.6917
15554.24619	9776.978331	0	18246.01302	108947.6647	10351.47397	120787.2151	52575.05396	56565.70752	62629.11118	31387.35766	0	57241.32245	55588.22968	29531.59794	66961.60788	235343.3358	37022.04112	113356.7735	75837.8706	20942.77419	0
1669799.219	2071453.78	2619873.036	5929764.818	4161945.17	5315118.161	4197121.755	4135844.172	5930374.398	9151934.365	3741358.68	2804339.395	4330703.621	5186573.344	4973133.205	6262939.697	7343396.316	3471897.2	4308692.308	7723399.158	3505994.774	4554403.966
30179.96842	17939.15174	271685.151	407218.2027	411725.7242	596555.8484	315822.2439	700775.8535	946579.2913	483506.3039	501320.2273	369518.2908	637467.8977	275604.451	0	1057927.89	378349.26	820551.5054	1931217.264	933570.1426	498144.0287	282800.3072
0	92083.33673	0	0	0	0	0	247058.8989	39378.66379	93177.40451	77979.98678	0	0	62937.42054	36507.92625	321931.6375	116582.7308	224843.8719	173299.5062	397870.0204	35150.66145	63205.9709
0	0	41680.1309	126165.8851	87093.94176	38614.82684	66828.675	110353.5015	42515.91772	0	96642.38534	57347.65157	117055.2791	393762.4795	157738.5676	203212.18	116395.7672	296314.459	152518.3286	196218.9529	0	0
255517.3159	521917.1751	0	0	0	0	0	59337.14043	812141.2932	478707.2353	0	0	0	0	0	0	0	0	152609.4006	0	230947.7781	0
784694.0509	666809.6399	1036322.883	496297.9427	802907.7486	1495832.442	1160047.209	1373572.057	1396787.8	247182.0278	1225434.676	779519.9477	953435.4904	2902957.419	1321006.848	988938.4309	1840818.407	749737.67	1470201.654	589981.2439	806132.6182	1248448.037
127767.6113	65182.50786	0	0	74367.26369	0	73731.21626	81944.7493	72523.32998	0	0	28524.4816	0	0	0	0	0	41377.5649	143343.4698	0	28631.56153	56774.75589
0	390470.6425	612733.4254	1518997.682	1627418.91	909917.4559	3829033.19	847832.95	2120432.964	1533285.465	1535183.014	1521713.545	867883.9411	1591871.253	1469960.31	1686852.773	1466592.577	1852207.36	1130076.374	931596.7355	1975154.473	1141316.961
837265.5659	227630.2098	0	0	703271.6086	0	0	0	213592.1543	421039.3435	278778.8771	0	0	267971.6834	534869.4475	1166339.286	121927.3243	1198776.287	215600.5619	531203.2971	1073674.711	0
759687.3857	131423.9195	1253190.485	1188598.044	1542299.602	453901.1676	541481.4786	1038028.995	1016167.335	1737737.006	1715051.227	0	836340.1974	637795.1036	690921.6112	1589141.292	489490.8945	854447.9583	691256.8729	2034136.828	1547222.243	373141.1408
105389.0785	0	61099.6828	239693.1675	339365.3842	51452.06738	3460068.951	193716.6017	0	175853.7204	0	638969.9663	306574.8853	836913.2396	0	257114.3561	146065.684	324679.274	694240.4065	0	1016461.116	0
1140642.129	3676224.513	8022804.325	5327941.374	5631933.419	4514721.811	4494823.46	2642097.316	4975167.139	2947333.612	9641239.374	101179.5908	5673512.113	4765027.069	4225535.135	10293203.57	5575922.689	6643079.392	7978069.199	8550119.738	5588689.233	7549496.68
0	0	0	32984.33727	9968.034832	6301.749089	15800.48524	15276.59847	0	0	42829.75781	0	13805.18161	26468.26134	6436.613746	40442.5387	140954.6828	19360.74394	30803.78248	79548.88004	42325.12642	3585.43257
90642.40426	75134.88609	144486.4629	432791.5818	761070.328	663406.4991	1342215.076	306905.8614	160393.1282	0	0	145189.8347	0	66346.0896	169924.2186	791505.4031	619800.7419	1032775.241	716849.4978	507228.4875	108308.3074	303036.1369
0	0	0	291187.1702	378595.9152	0	0	412046.3583	0	0	0	0	0	217763.9285	282495.0061	0	236885.2854	0	0	232101.9529	0	271664.6394
0	0	0	0	0	42728.61905	0	0	72696.07339	0	33279.01016	0	29413.42056	105112.6963	0	0	0	20937.71522	0	42477.28871	17141.79862	0
0	0	0	0	0	0	0	0	0	0	0	0	0	0	0	180498.2952	199280.0553	261191.6966	0	76922.2845	322857.8886	0
0	29433.00527	0	0	33879.84212	21987.44818	132100.1571	34125.27534	26706.41781	40511.36892	51798.51895	13115.73623	0	0	0	55175.60831	47597.24331	25962.29218	52612.65414	27771.27032	39709.8256	19074.00734
0	0	0	0	25017.17826	0	0	0	178611.3229	169985.0759	330915.8406	30507.50581	114690.6769	0	106705.53	149736.9754	0	30418.17995	0	209791.2782	41453.68668	0
1424590.038	1433914.667	3771082.497	2821238.43	1858388.705	2308997.403	2899976.952	4661344.333	3551037.17	3951220.737	3755604.032	2730720.217	2688143.156	4402262.482	2775007.812	3329236.202	2443749.599	3763714.198	3498436.172	3136418.419	3912761.756	3731798.046
0	76716.82386	0	174995.3985	0	163645.4763	0	200684.0789	0	0	0	121039.1213	0	271421.3519	204810.6265	246528.5324	0	0	0	0	313185.3772	269802.7107
301971.2225	63283.83064	865008.736	1236245.604	1991768.376	967033.5817	1216862.868	687181.5823	559906.3582	136677.6219	153502.7286	236375.0417	436412.1106	464038.8769	768356.8764	1898964.283	1678927.454	2037425.457	1786570.166	2138149.761	382992.5561	543084.3549
1597217.391	1048738.137	4143946.436	3331826.124	2510480.915	2733285.013	4712056.322	4175571.599	5031832.116	3337960.77	2505406.491	4772567.777	3328528.789	3702185.367	3735314.573	3892586.426	8619277.257	5008285.222	4558123.492	3942739.326	4238328.93	1217966.866
0	0	1554326.19	47160.01467	0	114052.7769	214470.0702	67879.22813	89421.70194	0	0	0	36184.93756	65367.45021	65732.74507	129778.4508	172602.8306	121711.7876	0	418639.9757	0	0
0	0	218853.9187	272493.3084	310971.36	0	0	153642.3873	0	0	0	147949.9935	0	0	0	0	0	104450.1666	0	0	126179.2773	0
0	0	0	0	0	5183.619001	350319.4001	0	0	34301.87392	59082.6525	0	0	25281.08387	79463.71224	105824.2518	0	93271.69673	0	40982.1754	126874.3518	0
122414224.8	107606047.9	173189947.2	242163348.5	191869713	156394630.3	181925687.6	257421000	159122857.1	104074055.2	145119453	196745444.4	176510583	146662499.3	163395003.6	155202851.4	178063265.3	195116679.6	153764961.9	172895304.6	217610107.8	169909862.2
53577.19718	152258.8905	98204.08756	602239.8924	380761.3638	440763.1572	165639.9629	304149.2442	163368.4795	287426.5081	433173.5468	98768.51907	198777.3773	391861.7221	494648.7837	267691.3905	181687.9613	130625.9034	96146.26008	365882.3869	364834.7841	207630.4974
1498622.67	1394180.951	2815396.993	2431195.817	2298558.948	2105458.454	1553599.131	1815283.044	1293373.28	1438188.105	2535720.891	1710245.217	865829.1908	2611429.221	1988957.378	3485051.718	2041588.994	7066092.87	1192252.178	2726571.96	2596101.239	1053403.833
257173.2365	272508.8215	0	716320.13	658440.1034	400588.7201	911134.6752	323262.1768	567053.6432	171591.3678	557324.5613	301054.2881	785275.4803	692415.8205	208695.0057	603733.7105	285516.2038	906561.5105	377838.2963	166153.6782	641834.7956	0
0	165199.9492	0	180118.738	0	0	226541.9516	0	0	0	0	0	0	0	0	0	0	0	0	0	0	354363.5573
0	0	0	37501.73339	44602.19365	0	0	0	0	0	0	0	0	66525.51698	0	0	0	0	0	54038.14558	0	164057.6744

[illegible]

0	38074.37847	0	0	50018.16394	0	351040.4803	0	0	0	0	58131.06091	21179.71845	101589.3776	0	0	[illegible]
556256.5408	1057358.058	755655.7648	1326915.375	958938.3239	2078640.451	878538.1256	2043492.039	3127108.025	1635048.272	973319.0376	1334571.741	7856010.031	2511091.685	2059342.026	3390714.509	[illegible]
151235.2394	0	0	0	122053.3171	16671.39455	137102.3517		0	0	0		0	26197.20248	35051.08644	0	[illegible]
0	0	0	0	156002.3744	0	0	91904.81796	75605.1686	84106.2053	0	86592.68996	0	81949.39519	0	0	[illegible]
613488.7255	846905.6919	2242100.794	806668.6356	1979147.472	2068121.028	2792117.946	1943937.779	1392176.015	1992798.628	1584161.325	2045984.199	1412433.181	2254621.288	2014032.438	2457974.865	[illegible]
0	0	713499.9564	726106.6536	750261.3288	125013.5543	676775.708	572975.2413	775333.9149	0	638181.5084	417792.8083	493159.996	530698.455	355916.7735	1766639.193	[illegible]
0	0	0	0	0	444072.5155	662648.7465	0	216386.7985	183162.8547	282930.8901	190941.5402	0	291770.2902	575478.5196	215607.4116	[illegible]
915964.467	354946.9101	500676.2964	708220.7448	1103977.257	1103337.71	1071840.445	832084.3467	464658.8988	370352.1441	538274.7333	233269.8585	494380.9639	1116194.501	472986.2702	1167475.737	[illegible]
0	359609.2926	403341.8605	1199450.075	125726.0747	274207.6716	146634.4802	380927.2625	634206.3215	0	417897.7534	852323.0035	1086807.502	1045673.966	783351.9698	1199124.138	[illegible]
1055357.135	0	519662.334	1086043.666	1114540.244	473379.4953	1311860.743	1251092.396	476524.2921	702504.2473	1360486.842	0	347262.3568	423755.3687	0	774428.0036	[illegible]
389714.0766	1332589.755	616550.6664	1861338.433	951012.1601	1174465.104	634861.9772	728052.2749	983500.6451	613037.1205	1831173.239	1858975.98	1488412.867	1503960.445	1416301.816	1225832.654	[illegible]
262824.0822	1086750.662	284560.5233	159478.3116	0	452819.3673	434068.6566	543787.608	548672.617	391859.7151	748041.5203	0	309215.2444	0	98121.84564	224712.0633	[illegible]
5883367.298	6404408.024	13194030.95	6731670.858	10565071.55	12057659.52	12524077.47	11266864.34	10334705.13	7910123.379	10463571.64	5756782.549	6936038.946	10980099.75	9314784.703	11904070.36	[illegible]
0	0	0	0	0	0	943318.7387	254411.693	0	0	0	0	0	0	0	144081.7094	[illegible]
23830.14551	0	169449.6793	220188.1047	86287.83235	106047.9721	178058.2832	166259.473	190124.4797	211895.0857	55109.53766	140279.0007	0	0	140217.0484	87088.52987	[illegible]
148677.0355	0	0	401827.9498	1343465.081	43003.75408	60163.31	0	0	188521.3463	211607.4729	67724.98204	88560.14168	267608.8929	208399.9242	959574.4239	[illegible]
612648.3357	324251.3177	1366238.162	811265.1362	2090311.547	1193077.842	552734.6854	1182942.431	1311919.225	1410438.691	1320139.774	719530.4195	1295364.763	1194261.165	1357950.428	1758099.133	[illegible]
0	0	0	0	0	223557.5785	105812.2179	0	0	0	0	0	0	0	49837.18715	0	[illegible]
0	0	2830392.732	3447579.681	0	2382114.879	0	1465968.778	1319107.973	0	2526485.495	3820825.122	1258913.411	1090421.207	5509728.234	1816240.799	[illegible]
256377.1846	0	184621.0514	323295.002	619900.4067	438684.5658	969009.087	408052.9083	968117.2474	337220.0377	456470.4888	276501.3466	318862.127	311500.9069	393104.5154	956681.589	[illegible]
228856.4683	0	92565.84284	92948.96637	56804.20533	92687.30239	258605.7826	121573.6119	68506.62031	0	286455.3904	0	45883.37349	147443.0699	139564.7401	248898.6468	[illegible]
6827567.947	14410101.32	4457742.885	8859319.698	12120088.66	5781724.971	6235085.383	6835985.712	8621987.031	4431451.572	8503727.264	11451207.32	3171821.679	6779743.285	3601650.509	8959450.425	[illegible]
186290.8697	59136.48898	668837.6934	308512.5016	93303.39243	81031.01126	175737.2795	964440.7211	640626.9821	82416.05635	197283.9879	27248.43456	308383.046	472591.1794	921122.0007	500060.905	[illegible]
4937834.155	6932857.521	4937007.296	10513506.59	12169029.43	9139511.861	12056336.71	9422980.424	8715041.931	7383158.18	4682554.959	6100160.141	8496392.61	9190302.143	8709585.782	10201528.92	[illegible]
0	0	0	0	1545479.85	0	0	0	1193048.466	1273783.783	0	423284.474	0	817363.6937	0	1022425.995	[illegible]
816224.6644	987122.0882	0	0	1002035.045	3518604.459	2517067.987	1911535.79	1569082.906	2068673.488	1540487.706	1601486.676	3183907.578	2109871.649	0	2693732.994	[illegible]
3089204.45	1259715.479	3595440.509	1290005.908	1615400.698	5532246.329	8923801.159	8478790.767	9588666.136	8906593.279	2951359.865	2529782.576	7923839.604	4819677.968	5565284.686	5463374.942	[illegible]
4952703.846	9548567.732	11303105.84	15264980.77	17923221.79	14063666.49	12708738.28	14664540.14	15554896.27	6868799.531	10879090.51	15506811.47	12464954.03	16103171.04	12537068.18	11935044.07	[illegible]
53053.34687	34936.58993	0	0	0	0	0	0	0	137632.1358	39747.53928	0	109384.8814	166693.8284	102182.2882	259332.5175	[illegible]
12922222.41	15736642.03	18003665.67	12186741.88	10210233.01	19740665.79	20697251.39	18253870.73	12505469.26	17194921.54	18371793.62	20802921.34	29581103.92	23504766.18	20703027.95	32148790.52	[illegible]
3635474.648	9973779.886	8190661.429	14833499.03	18182758.61	9015352.121	10520436.35	17885244.16	15686027.76	4661564.501	9365435.495	27747646.94	3699207.411	5228139.179	3662220.839	14597857.88	[illegible]
32387.16339	33963.97493	0	63502.09507	170140.9023	0	30500.92827	56471.44152	0	72528.3355	0	162794.3135	0	274093.1393	39120.09155	28834.15364	[illegible]
106327.0901	306100.2425	854494.3679	1254459.584	787868.9826	161154.2266	656328.314	530865.069	677705.1306	122347.4189	197898.6747	306200.682	764358.9925	309021.2648	848367.4068	1688416.201	[illegible]
0	224646.7384	240594.6066	0	300251.4481	331545.2707	0	548754.299	132030.4054	0	149623.9963	897715.4314	281649.0749	263507.7434	386687.1124	386947.7601	[illegible]
38639933.33	31502745.16	25368780.4	59025559.46	45975826.45	38305088.13	37838364.53	41476118.54	30287911.11	36952236.29	35605424.75	17260347.66	31551777.51	28909156.33	24661498.91	21814021.72	[illegible]
0	0	0	92037.28006	102449.949	134719.6665	71597.97705	86464.52803	0	0	84109.88564	181202.8823	0	94732.79146	0	137805.9584	[illegible]
2652536.502	2317368.19	3181718.482	5293952.225	4399779.73	7394320.946	4221050.294	5497294.621	5717300.928	5688488.051	3909449.326	4219394.425	3289964.523	5002394.694	2973507.445	7208258.639	[illegible]
0	85788.70439	211773.1154	304457.6195	52012.52562	280061.7068	107958.0305	137091.4272	315410.7705	234889.6505	71370.80449	370916.0719	434734.7392	316988.5954	382424.7462	203716.7529	[illegible]
492711.6085	506262.2219	719647.6948	880129.3023	1768159.11	1216181.3	1008627.362	192570.4182	189850.7425	852257.0082	2027150.319	680406.3287	850317.1282	984023.0882	641876.5821	635975.502	[illegible]
382706.8976	275966.956	585873.1594	876194.5252	1041157.643	249409.7383	1764505.289	1664577.78	1034332.693	1205102.312	904036.8482	634349.4048	88190.34839	798255.352	1098249.723	2033061.428	[illegible]
165987.3205	0	0	195349.7596	109127.6307	0	560943.0808	0	0	272835.1828	212872.3493	0	0	0	486209.978	0	[illegible]
1162745.803	1012851.925	2277456.025	3489971.836	562759.2676	3144330.863	1970089.244	1484064.773	3776568.078	2405065.827	1602661.965	2203713.133	2066853.489	2467149.927	3542246.69	2030889.973	[illegible]
0	419186.3344	2377176.056	2859355.536	896467.6411	1299615.157	1094681.597	3213326.935	5978991.761	3901182.572	1973351.106	664830.7607	1666038.019	2221491.031	3291524.022	3695846.972	[illegible]
586171.8084	924619.0287	698159.7316	4546306.27	2614405.977	2610505.356	2612160.022	1320108.648	1131540.134	341678.5528	2078383.461	425965.3845	1166348.719	1486817.343	541612.8639	2290639.111	[illegible]
0	285631.2606	0	0	70347.13492	1195840.256	0	196296.0547	77442.65646	0	671628.1958	0	136928.6248	0	334588.6193	241159.7911	[illegible]
250059.7866	231468.3758	486044.6696	782104.2514	331187.078	884729.0432	1883447.764	433017.9651	308799.1443	0	486371.0341	561956.5661	489366.5197	829209.9531	1006370.688	1393992.675	[illegible]
12996173.65	8078497.705	18210616.87	18693675.18	15059139.63	18227942.39	20932557.12	12528070.93	17158694.72	16237625.21	14828823.64	11377454.59	10836304.94	12174534.03	16470812.34	24349776.75	[illegible]
84907.23605	31132.43918	21144.79264	130595.1666	2468417.413	279371.1315	779374.1078	167298.5239	33127.91341	49355.06886	61618.26775	34614.75957	56456.2742	86768.36183	34707.76718	270499.8359	[illegible]
0	0	0	70514.64284	0	267811.4282	163849.3828	63390.38786	0	304319.2363	108775.2212	0	194467.2685	120160.4365	29033.79506	82714.51663	[illegible]
0	0	301961.3582	119392.6132	0	137136.1447	382650.8504	149432.7731	133546.9166	0	269510.8461	0	53357.05351	0	42329.5227	69738.80559	[illegible]
10579574.07	9339748.135	20253203.42	10584484.25	3836368.674	3412845.013	3055236.529	18565506.41	13549943.29	6446227.071	12507641.96	18692443.25	3079102.507	4801017.371	19517950.5	8769043.661	[illegible]
2722970.425	2192031.638	5375424.268	9802452.193	10083418.43	3974313.43	9144853.416	4009524.826	4772786.198	5482577.238	4467755.008	3111085.024	3142900.828	5859888.648	5481788.473	5553327.613	[illegible]
119970.8715	190192.7127	132459.4	633394.0868	697109.0424	611489.6997	460259.261	285924.6983	305143.6964	76570.35526	477756.6307	292385.374	610433.5281	343899.1719	529340.6617	542975.7353	[illegible]
0	0	245021.1461	173362.1861	115901.8036	186672.5887	272517.8696	0	215791.853	300994.3752	182341.5926	0	0	184498.762	0	1368311.112	[illegible]
0	0	0	0	45111.56918	260207.1284	0	0	0	0	0	585370.2729	302260.1764	322005.2222	378610.0111	0	[illegible]
0	0	58396.87806	246036.8636	54692.17842	255275.1555	65362.79643	63240.03258	157591.3597	65412.86838	95927.71104	269227.6914	44239.08002	27626.3172	321058.1596	150593.7183	[illegible]
497653.5054	1392244.038	1062234.041	1712687.705	1779744.283	1089522.155	884473.265	1392828.179	1641696.423	1434965.835	1252734.753	1789071.239	1302996.001	1201150.936	1733065.27	2730575.266	[illegible]
177316.9374	212484.7274	540003.3365	724567.4728	2000420.254	705474.8037	1363034.072	595829.5939	644987.8109	550028.4336	404242.8064	0	124991.1185	186652.3358	0	1165293.657	[illegible]
0	92959.60777	241687.7011	0	236563.9386	341816.1932	994181.7473	262828.4866	236917.8098	1025723.441	114929.3897	1136445.541	416701.4982	193502.7236	197165.2446	1120957.591	[illegible]
1174585.977	2463859.643	2570740.801	4024977.44	6288004.793	7080155.633	8433493.347	2322580.208	2220399.799	4089029.845	3118749.852	1193068.7	1738324.177	906416.0876	6572873.276	5202858.243	[illegible]
971580.8162	4184139.875	3156613.754	2314479.572	5033050.132	3033254.144	8736167.097	2158773.518	2091121.383	1793363.483	2633597.631	6281182.138	9591292.248	8118527.248	5233547.869	7907498.591	[illegible]
1099049.373	3150205.055	4463831.988	14530295.88	19081477.37	11123150.92	20286224.71	9120008.407	15657923.01	9635177.022	9475967.154	4146414.66	2516248.618	16477406.57	12037139.61	0	[illegible]
0	0	0	0	1316025.734	0	739379.032	516797.0429	0	0	0	0	0	0	0	0	[illegible]
0	230706.4426	197838.2885	541869.4215	102789.9304	580736.0977	717308.0848	1092493.713	824914.1184	425206.5881	235191.3905	909468.9808	0	498115.6213	1301369.926	582092.1747	[illegible]
286884.3485	0	1761845.186	1761550.052	3942347.25	1753006.465	1486385.172	1340064.596	1222887.839	1795479.049	2460961.231	1656850.205	1910022.637	2139604.095	2025307.939	4220833.291	[illegible]
[illegible]																

[illegible]

6906114.974	4412778.908	4410672.841	3295472.293	0	2811713.414	4775999.01	10471565.23	4282633.63	3405290.783	10830135.81	0	2922713.833	2553390.615	4875397.301	2998834.744	7314404.658	5443128.079	8513616.287	3258889.305	0	7231198.538
581989.2751	2753391.28	2458575.856	6824310.404	5128536.04	1600101.139	3077146.66	3842277.091	2289928.411	6465501.869	5689283.79	2755205.383	2948917.992	3335442.913	1474982.819	2479030.613	4212737.15	2452115.302	4505168.262	3694334.975	1253440.918	3883264.984
145765.3987	748238.6271	823443.6893	940042.9016	623750.9658	1350113.53	1334484.551	764446.705	1228378.372	497118.6058	73856.94259	518463.5558	1045014.824	917723.2074	1422360.551	974411.578	350181.0456	455652.2445	819051.0332	901738.9019	0	861219.2618
1438596.407	1437692.733	1772623.778	0	1354541.197	2187538.54	618454.8512	2463688.336	3145451.5	1538657.131	2449371.293	4069417.873	1858835.919	2327903.792	2149738.781	142270.5987	999495.7341	3478952.8	2304368	2280493.734	2097933.883	2707710.424
592922.9154	1256340.985	89438.6525	585066.4384	2597538.294	1567942.697	2073915.148	940141.1695	1963959.422	345584.1883	741654.5693	2266754.767	418234.0901	1341741.687	324766.6172	3504042.972	770583.5453	2404472.043	1183399.476	1613078.645	1257987.854	263503.9705
530768.0353	105874.4984	0	0	305408.9452	430658.5234	232810.8197	92604.85598	67093.34009	458758.9464	364342.2079	0	168731.44	51407.14911	409746.8277	0	0	171737.459	109005.4061	94752.83004	752520.5553	106278.7809
5480293.125	2550763.598	12163725.68	960134.0499	1327684.559	3890704.923	2619949.558	9771699.711	6945318.01	5629908.014	8528378.821	6925036.622	9666335.838	10346650.2	13480529.65	6559808.526	6465780.631	3709386.1	7543987.826	6754192.495	2726590.162	6324893.706
0	0	0	0	406752.1668	0	107242.3247	0	21626.07224	0	27852.08071	0	0	0	0	0	0	235420.4762	0	0	0	0
1375703.439	1200584.182	2787380.304	2769693.332	2988396.301	2662719.876	1134860.001	2100803.852	965914.3258	900086.2172	1837818.652	3334076.339	1341282.672	1228876.561	1891592.454	1003455.042	1474387.596	2727527.854	9231245.88	4609187.056	2375371.118	3857501.441
9959292.005	9033109.295	14205478.27	7000047.533	8721586.715	7843172.915	10533467.29	15041532.99	10079281.52	5113828.311	5041322.527	14975250.18	25211804.39	11926015.65	8275058.356	9855826.026	10104945.6	18608466.69	10754548.45	15364463.49	22704001.05	2894576.909
0	0	201692.2988	1142467.774	97416.61274	684607.6926	887110.7277	629890.7091	673292.475	343776.081	432033.2831	1578671.437	931083.758	1022164.264	1226652.318	798033.3586	1092368.316	847559.0983	1355391.639	649346.5842	747178.1538	717522.0295
5636990.176	7257942.816	17303260.03	16447416.83	13730507.59	17657836.19	14319229.72	29158264.55	22747982.56	7943225.937	9599133.041	19130538.39	18484909.1	21782893.36	4704236.911	16529295.66	20422073.41	18722638	25445511.67	16532711.48	16464859.36	6401792.042
0	0	0	0	1770025.73	0	2022246.623	0	0	0	101523.5359	0	0	0	0	0	0	246171.7466	0	0	0	0
0	0	0	410682.4199	354600.1629	281933.0105	304587.0963	0	511825.4092	325116.2361	497028.5235	168000.395	0	0	333783.523	321551.4523	310487.9528	0	0	342747.1458	207587.1893	212232.8911
301043.5638	184984.5216	845069.4816	411706.0788	0	86989.41723	0	274904.4253	431617.7588	501416.9068	390093.6479	493058.5871	0	294063.6744	0	1314195.837	954466.0563	1083562.869	622447.1049	70891.50666	46761.07037	0
46072.17345	0	178262.5754	192764.785	280850.2533	157590.0747	215854.4596	231080.9842	146838.5121	175286.5816	282521.8028	283234.4334	331392.5585	232540.589	316272.0173	474503.5977	216019.4698	193607.1653	179783.1105	262807.0723	0	42095.00922
533047.3748	2913821.715	1975746.694	2218668.044	2447727.21	4973806.024	5781034.945	2011603.026	3049850.786	2431184.924	2065204.447	3404132.041	2621164.651	2444105.339	2360979.478	3443502.511	1958865.254	5973818.221	4244974.398	3553590.902	2890103.39	3303999.987
0	74355.37476	556784.9428	100120.454	365407.0155	0	98886.79476	360975.221	456972.6384	353412.1698	309545.8578	0	289406.3275	587614.9484	276392.8363	675195.6346	387812.6182	577843.6728	176909.2726	149867.5451	265282.5266	0
0	0	0	0	0	0	0	19248.50782	31796.83723	0	0	0	0	0	0	33267.02147	46969.88567	74877.79564	0	67260.0742	0	0
0	0	0	104507.6525	609533.4015	0	233374.0282	0	0	0	0	278882.5008	0	0	179729.7324	262211.2163	152415.3073	0	36968.75185	0	0	0
1273218.352	2792146.806	1292266.509	2126115.154	2257357.153	3179231.182	3129985.342	3425428.356	2914470.643	2805454.64	3177890.277	2082139.74	1470563.072	2341265.859	2321383.004	2682380.518	3668907.131	3143145.801	3051084.647	2248708.151	1053968.208	852848.1706
